# Supplementary material for: Diversity of Clinically Relevant Outcomes Resulting from Hypofractionated Radiation in Human Glioma Stem Cells Mirrors Distinct Patterns of Transcriptomic Changes
Source: Cancers (Basel). 2020 Mar 1;12(3):570. doi: 10.3390/cancers12030570 (PMC7139840; doi:10.3390/cancers12030570)
Supplement: Supplementary file 1 [file cancers-12-00570-s001.pdf]

Figure S1

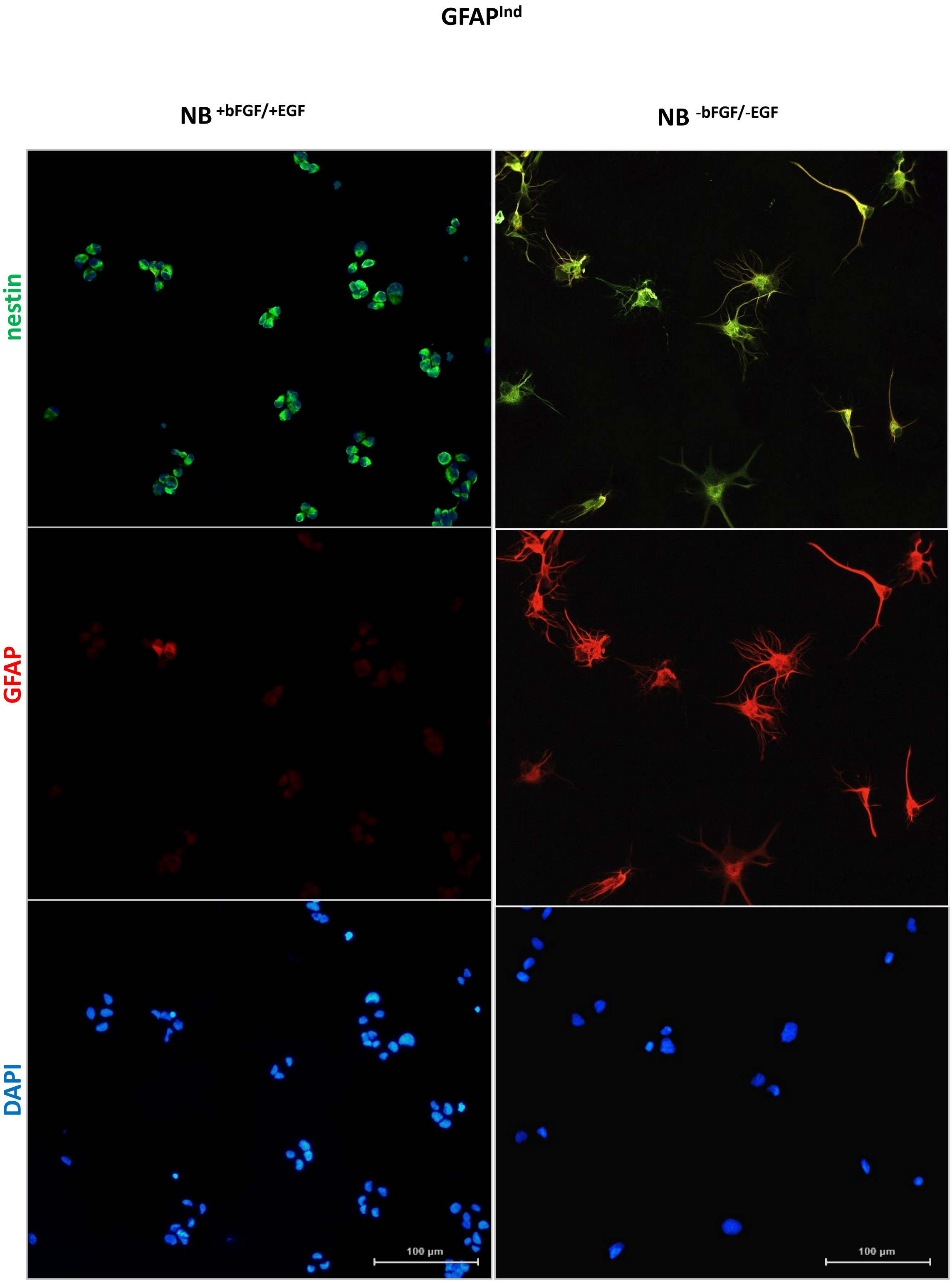

(a)

Figure S1

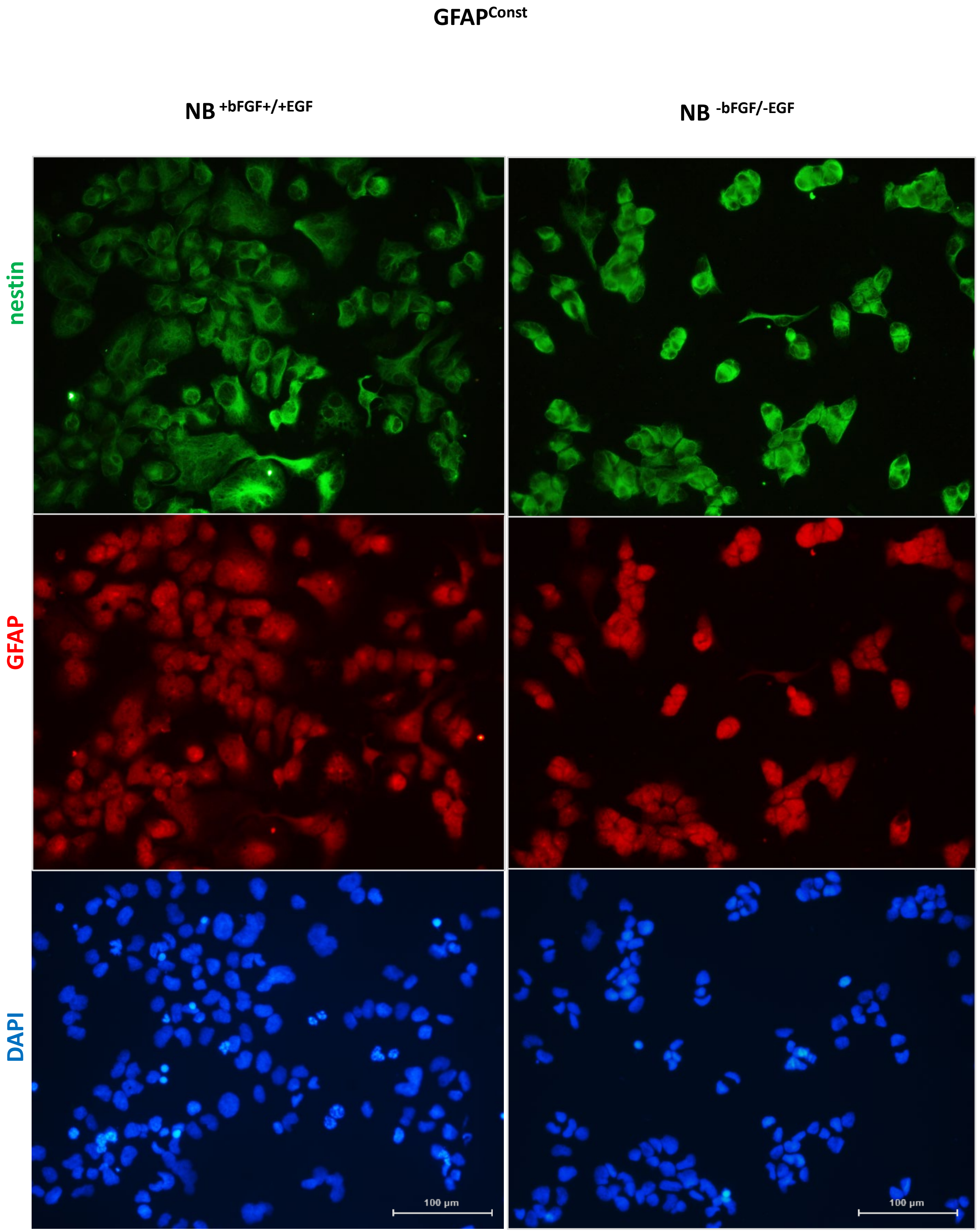
GFAPConst
NB +bFGF+/+EGF
NB -bFGF/-EGF
nestin
GFAP
DAPI
100 µm
100 µm

(b)

Figure S2

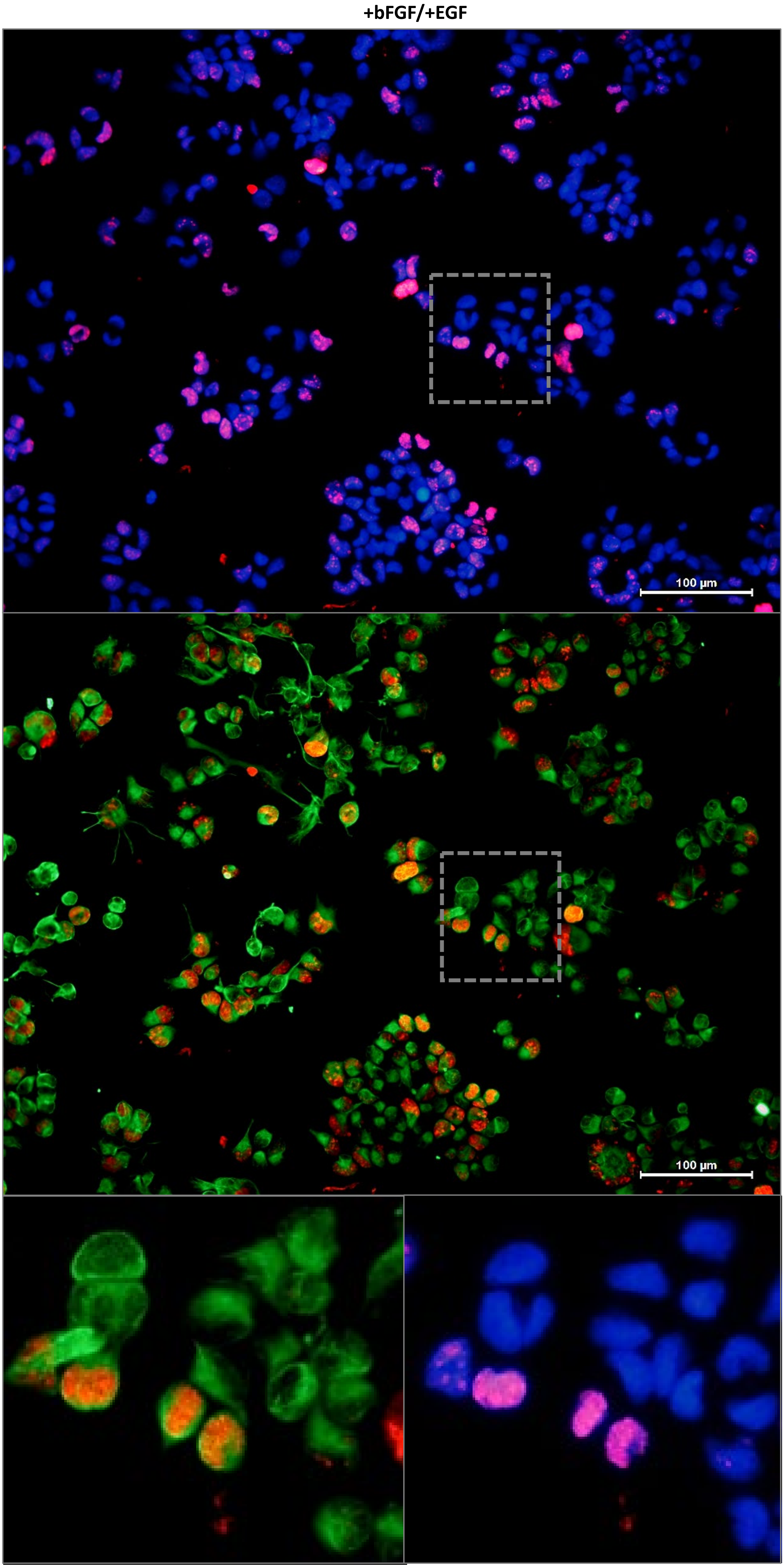

(a)

Figure S2

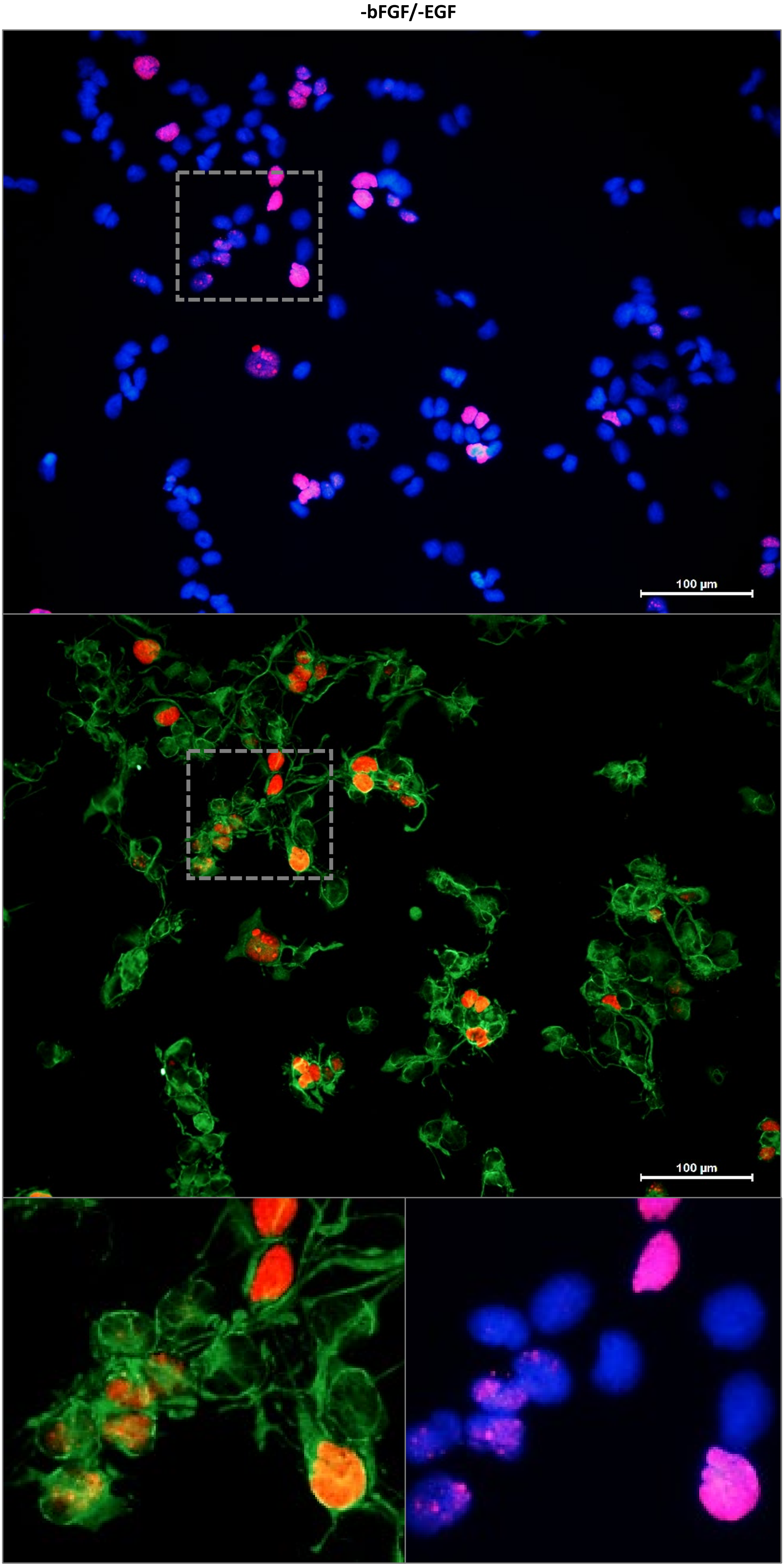

(b)

Figure S3

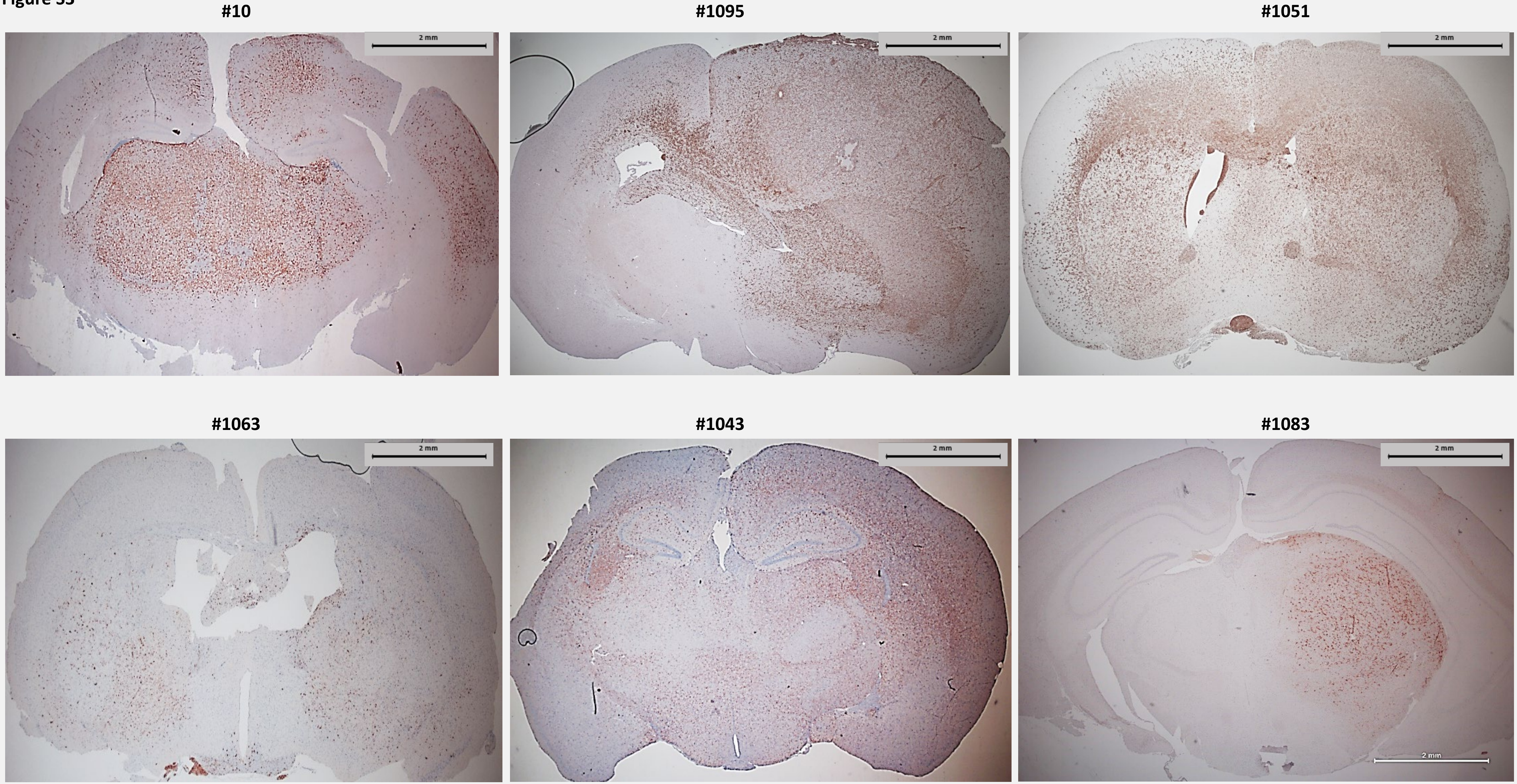

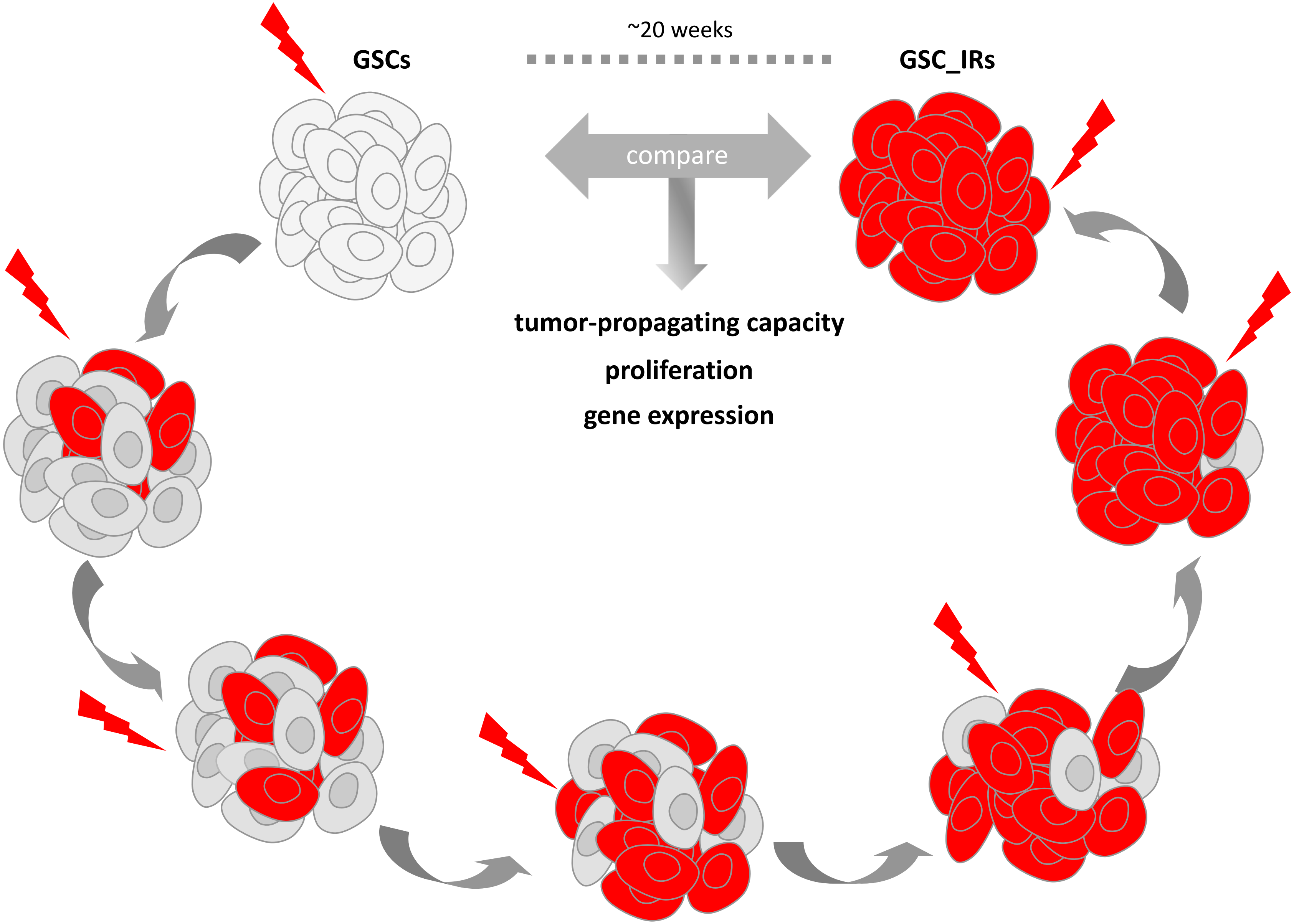
~20 weeks
GSCs
GSC_IRs
compare
tumor-propagating capacity
proliferation
gene expression

Supplemental Table S1. Expression patterns of nestin and GFAP in GSCs self-renewing *in vitro*.

GSC line	nestin (%)	GFAP (%)
#10	90 ± 1,9	26 ± 5,8
#1095	92 ± 5,4	1,45 ± 0,1
#1063	74,4 ± 27,9	3,14 ± 1,8
#1051	92 ± 1,3	< 1
#1043	96,7 ± 1,4	96,7 ± 1,4
#1080	99 ± 0,6	99 ± 0,6
#1083	64,5 ± 14,1	64,5 ± 14,1
G112-NB	99 ± 1,6	99 ± 1,6

Immunofluorescence staining of GSCs cultured under self-renewal promoting condition

Supplemental Table S2. Gene expression analysis by Gene Ontology terms.

Names	total	elements
#1051IR #1063IR #1083IR #1095IR #10IR	144	GO:0006810: transport
		GO:0006915: apoptosis
		GO:0005515: protein binding
		GO:0005789: endoplasmic reticulum membrane
		GO:0050896: response to stimulus
		GO:0006355: regulation of transcription, DNA-dependent
		GO:0007601: visual perception
		GO:0043565: sequence-specific DNA binding
		GO:0003676: nucleic acid binding
		GO:0007264: small GTPase mediated signal transduction
		GO:0006461: protein complex assembly
		GO:0007605: sensory perception of sound
		GO:0045202: synapse
		GO:0005730: nucleolus
		GO:0042475: odontogenesis of dentine-containing tooth
		GO:0043234: protein complex
		GO:0042734: presynaptic membrane
		GO:0006813: potassium ion transport
		GO:0005200: structural constituent of cytoskeleton
		GO:0007420: brain development
		GO:0030173: integral to Golgi membrane
		GO:0042803: protein homodimerization activity
		GO:0042127: regulation of cell proliferation
		GO:0016787: hydrolase activity
		GO:0030659: cytoplasmic vesicle membrane
		GO:0016757: transferase activity, transferring glycosyl groups
		GO:0005518: collagen binding
		GO:0001503: ossification
		GO:0040008: regulation of growth
		GO:0008134: transcription factor binding
		GO:0009611: response to wounding
		GO:0015293: symporter activity
		GO:0004872: receptor activity
		GO:0008380: RNA splicing
		GO:0005216: ion channel activity
		GO:0048306: calcium-dependent protein binding
		GO:0007268: synaptic transmission
		GO:0005575: cellular_component
		GO:0005622: intracellular
		GO:0016491: oxidoreductase activity
		GO:0016021: integral to membrane
		GO:0009055: electron carrier activity
		GO:0003779: actin binding
		GO:0008270: zinc ion binding
		GO:0017124: SH3 domain binding
		GO:0016324: apical plasma membrane
		GO:0006816: calcium ion transport
		GO:0005525: GTP binding
		GO:0045087: innate immune response
		GO:0009968: negative regulation of signal transduction
		GO:0008283: cell proliferation
		GO:0008083: growth factor activity
		GO:0005604: basement membrane
		GO:0046872: metal ion binding
		GO:0005516: calmodulin binding
		GO:0007169: transmembrane receptor protein tyrosine kinase signaling pathway
		GO:0005509: calcium ion binding
		GO:0008015: blood circulation
		GO:0003677: DNA binding
		GO:0019901: protein kinase binding
		GO:0007275: multicellular organismal development
		GO:0005578: proteinaceous extracellular matrix
		GO:0006886: intracellular protein transport
		GO:0005829: cytosol
		GO:0016563: transcription activator activity
		GO:0007186: G-protein coupled receptor protein signaling pathway
		GO:0006334: nucleosome assembly
		GO:0006357: regulation of transcription from RNA polymerase II promoter
		GO:0030054: cell junction
		GO:0005488: binding
		GO:0006811: ion transport
		GO:0031410: cytoplasmic vesicle
		GO:0005215: transporter activity
		GO:0001558: regulation of cell growth
		GO:0000287: magnesium ion binding
		GO:0045211: postsynaptic membrane
		GO:0016740: transferase activity
		GO:0005576: extracellular region
		GO:0008150: biological_process
		GO:0005769: early endosome
		GO:0000122: negative regulation of transcription from RNA polymerase II promoter
		GO:0004930: G-protein coupled receptor activity
		GO:0005886: plasma membrane
		GO:0003674: molecular_function
		GO:0030154: cell differentiation
		GO:0050501: hyaluronan synthase activity
		GO:0007179: transforming growth factor beta receptor signaling pathway
		GO:0019717: synaptosome
		GO:0008285: negative regulation of cell proliferation
		GO:0012505: endomembrane system
		GO:0007267: cell-cell signaling
		GO:0005783: endoplasmic reticulum
		GO:0000139: Golgi membrane
		GO:0003924: GTPase activity
		GO:0009986: cell surface
		GO:0008152: metabolic process
		GO:0007417: central nervous system development
		GO:0016020: membrane
		GO:0004674: protein serine/threonine kinase activity
		GO:0005615: extracellular space
		GO:0031093: platelet alpha granule lumen
		GO:0006916: anti-apoptosis
		GO:0016337: cell-cell adhesion
		GO:0006457: protein folding
		GO:0006814: sodium ion transport
		GO:0005856: cytoskeleton
		GO:0030335: positive regulation of cell migration
		GO:0007528: neuromuscular junction development
		GO:0030199: collagen fibril organization
		GO:0003824: catalytic activity
		GO:0005624: membrane fraction
		GO:0007156: homophilic cell adhesion
		GO:0005524: ATP binding
		GO:0005540: hyaluronic acid binding
		GO:0005737: cytoplasm
		GO:0000166: nucleotide binding
		GO:0007399: nervous system development
		GO:0001726: ruffle
		GO:0004871: signal transducer activity
		GO:0005125: cytokine activity
		GO:0005882: intermediate filament
		GO:0030426: growth cone
		GO:0005529: sugar binding
		GO:0007165: signal transduction
		GO:0015031: protein transport
		GO:0045944: positive regulation of transcription from RNA polymerase II promoter

		GO:0005634: nucleus
		GO:0007507: heart development
		GO:0005244: voltage-gated ion channel activity
		GO:0007155: cell adhesion
		GO:0003723: RNA binding
		GO:0005887: integral to plasma membrane
		GO:0005254: chloride channel activity
		GO:0005096: GTPase activator activity
		GO:0043123: positive regulation of I-kappaB kinase/NF-kappaB cascade
		GO:0006954: inflammatory response
		GO:0009887: organ morphogenesis
		GO:0008378: galactosyltransferase activity
		GO:0005201: extracellular matrix structural constituent
		GO:0005794: Golgi apparatus
		GO:0048471: perinuclear region of cytoplasm
		GO:0046982: protein heterodimerization activity
		GO:0005874: microtubule
		GO:0005507: copper ion binding
#1051IR #1083IR #1095IR #10IR	6	GO:0045786: negative regulation of cell cycle
		GO:0008307: structural constituent of muscle
		GO:0051082: unfolded protein binding
		GO:0006397: mRNA processing
		GO:0000086: G2/M transition of mitotic cell cycle
		GO:0030145: manganese ion binding
#1063IR #1083IR #1095IR #10IR	87	GO:0006950: response to stress
		GO:0044419: interspecies interaction between organisms
		GO:0004888: transmembrane receptor activity
		GO:0051092: positive regulation of NF-kappaB transcription factor activity
		GO:0001764: neuron migration
		GO:0042169: SH2 domain binding
		GO:0030879: mammary gland development
		GO:0042995: cell projection
		GO:0016607: nuclear speck
		GO:0007411: axon guidance
		GO:0005543: phospholipid binding
		GO:0003009: skeletal muscle contraction
		GO:0042476: odontogenesis
		GO:0008277: regulation of G-protein coupled receptor protein signaling pathway
		GO:0043197: dendritic spine
		GO:0004842: ubiquitin-protein ligase activity
		GO:0006511: ubiquitin-dependent protein catabolic process
		GO:0042493: response to drug
		GO:0007413: axonal fasciculation
		GO:0050806: positive regulation of synaptic transmission
		GO:0004725: protein tyrosine phosphatase activity
		GO:0030036: actin cytoskeleton organization
		GO:0031623: receptor internalization
		GO:0016042: lipid catabolic process
		GO:0051966: regulation of synaptic transmission, glutamatergic
		GO:0007218: neuropeptide signaling pathway
		GO:0006887: exocytosis
		GO:0015629: actin cytoskeleton
		GO:0030326: embryonic limb morphogenesis
		GO:0008092: cytoskeletal protein binding
		GO:0042472: inner ear morphogenesis
		GO:0005234: extracellular-glutamate-gated ion channel activity
		GO:0007596: blood coagulation
		GO:0006260: DNA replication
		GO:0005102: receptor binding
		GO:0043065: positive regulation of apoptosis
		GO:0008022: protein C-terminus binding
		GO:0005792: microsome
		GO:0045941: positive regulation of transcription
		GO:0015277: kainate selective glutamate receptor activity
		GO:0007018: microtubule-based movement
		GO:0045177: apical part of cell
		GO:0005654: nucleoplasm
		GO:0004437: inositol or phosphatidylinositol phosphatase activity
		GO:0001501: skeletal system development
		GO:0042802: identical protein binding
		GO:0016788: hydrolase activity, acting on ester bonds
		GO:0016363: nuclear matrix
		GO:0006936: muscle contraction
		GO:0003713: transcription coactivator activity
		GO:0051015: actin filament binding
		GO:0051260: protein homooligomerization
		GO:0009116: nucleoside metabolic process
		GO:0030018: Z disc
		GO:0030971: receptor tyrosine kinase binding
		GO:0003682: chromatin binding
		GO:0008104: protein localization
		GO:0007067: mitosis
		GO:0017111: nucleoside-triphosphatase activity
		GO:0051056: regulation of small GTPase mediated signal transduction
		GO:0016874: ligase activity
		GO:0030111: regulation of Wnt receptor signaling pathway
		GO:0001525: angiogenesis
		GO:0043005: neuron projection
		GO:0030424: axon
		GO:0003714: transcription corepressor activity
		GO:0042470: melanosome
		GO:0043687: post-translational protein modification
		GO:0007160: cell-matrix adhesion
		GO:0005667: transcription factor complex
		GO:0020037: heme binding
		GO:0007049: cell cycle
		GO:0005694: chromosome
		GO:0005544: calcium-dependent phospholipid binding
		GO:0008016: regulation of heart contraction
		GO:0019899: enzyme binding
		GO:0004859: phospholipase inhibitor activity
		GO:0019904: protein domain specific binding
		GO:0009897: external side of plasma membrane
		GO:0030139: endocytic vesicle
		GO:0051301: cell division
		GO:0009408: response to heat
		GO:0005625: soluble fraction
		GO:0030660: Golgi-associated vesicle membrane
		GO:0016459: myosin complex
		GO:0005975: carbohydrate metabolic process
		GO:0016055: Wnt receptor signaling pathway
#1051IR #1063IR #1095IR #10IR	98	GO:0030674: protein binding, bridging
		GO:0007409: axonogenesis
		GO:0016481: negative regulation of transcription
		GO:0042310: vasoconstriction
		GO:0048246: macrophage chemotaxis
		GO:0031225: anchored to membrane
		GO:0006641: triglyceride metabolic process
		GO:0042632: cholesterol homeostasis
		GO:0030539: male genitalia development
		GO:0016323: basolateral plasma membrane
		GO:0008284: positive regulation of cell proliferation
		GO:0006958: complement activation, classical pathway
		GO:0005161: platelet-derived growth factor receptor binding
		GO:0004528: phosphodiesterase I activity
		GO:0007584: response to nutrient
		GO:0050839: cell adhesion molecule binding

		GO:0008233: peptidase activity
		GO:0006812: cation transport
		GO:0006869: lipid transport
		GO:0004867: serine-type endopeptidase inhibitor activity
		GO:0050656: 3'-phosphoadenosine 5'-phosphosulfate binding
		GO:0016477: cell migration
		GO:0006836: neurotransmitter transport
		GO:0030346: protein phosphatase 2B binding
		GO:0006979: response to oxidative stress
		GO:0006897: endocytosis
		GO:0008201: heparin binding
		GO:0030324: lung development
		GO:0005923: tight junction
		GO:0005813: centrosome
		GO:0019838: growth factor binding
		GO:0005085: guanyl-nucleotide exchange factor activity
		GO:0016023: cytoplasmic membrane-bounded vesicle
		GO:0043193: positive regulation of gene-specific transcription
		GO:0004714: transmembrane receptor protein tyrosine kinase activity
		GO:0005021: vascular endothelial growth factor receptor activity
		GO:0004176: ATP-dependent peptidase activity
		GO:0030505: inorganic diphosphate transport
		GO:0048008: platelet-derived growth factor receptor signaling pathway
		GO:0004252: serine-type endopeptidase activity
		GO:0030027: lamellipodium
		GO:0009653: anatomical structure morphogenesis
		GO:0007586: digestion
		GO:0016942: insulin-like growth factor binding protein complex
		GO:0008239: dipeptidyl-peptidase activity
		GO:0006952: defense response
		GO:0016192: vesicle-mediated transport
		GO:0016887: ATPase activity
		GO:0000278: mitotic cell cycle
		GO:0032869: cellular response to insulin stimulus
		GO:0045121: membrane raft
		GO:0006508: proteolysis
		GO:0042594: response to starvation
		GO:0030334: regulation of cell migration
		GO:0009725: response to hormone stimulus
		GO:0022900: electron transport chain
		GO:0001570: vasculogenesis
		GO:0031994: insulin-like growth factor I binding
		GO:0005230: extracellular ligand-gated ion channel activity
		GO:0007259: JAK-STAT cascade
		GO:0007194: negative regulation of adenylate cyclase activity
		GO:0001755: neural crest cell migration
		GO:0048662: negative regulation of smooth muscle cell proliferation
		GO:0008289: lipid binding
		GO:0045449: regulation of transcription
		GO:0051020: GTPase binding
		GO:0008219: cell death
		GO:0030521: androgen receptor signaling pathway
		GO:0006629: lipid metabolic process
		GO:0042981: regulation of apoptosis
		GO:0014912: negative regulation of smooth muscle cell migration
		GO:0030198: extracellular matrix organization
		GO:0005506: iron ion binding
		GO:0030500: regulation of bone mineralization
		GO:0030162: regulation of proteolysis
		GO:0007229: integrin-mediated signaling pathway
		GO:0005057: receptor signaling protein activity
		GO:0003774: motor activity
		GO:0004222: metalloendopeptidase activity
		GO:0007200: activation of phospholipase C activity by G-protein coupled receptor protein signaling pathway coupled to IP3 second messenger
		GO:0006796: phosphate metabolic process
		GO:0008168: methyltransferase activity
		GO:0006826: iron ion transport
		GO:0019722: calcium-mediated signaling
		GO:0006874: cellular calcium ion homeostasis
		GO:0042391: regulation of membrane potential
		GO:0005739: mitochondrion
		GO:0006955: immune response
		GO:0004551: nucleotide diphosphatase activity
		GO:0008217: regulation of blood pressure
		GO:0030528: transcription regulator activity
		GO:0005158: insulin receptor binding
		GO:0030308: negative regulation of cell growth
		GO:0007171: activation of transmembrane receptor protein tyrosine kinase activity
		GO:0005911: cell-cell junction
		GO:0010008: endosome membrane
		GO:0045599: negative regulation of fat cell differentiation
		GO:0006091: generation of precursor metabolites and energy
#1051IR #1063IR #1083IR #10IR	2	GO:0030165: PDZ domain binding
		GO:0016339: calcium-dependent cell-cell adhesion
#1051IR #1063IR #1083IR #1095IR	7	GO:0006917: induction of apoptosis
		GO:0005875: microtubule associated complex
		GO:0005891: voltage-gated calcium channel complex
		GO:0007626: locomotory behavior
		GO:0004896: cytokine receptor activity
		GO:0016301: kinase activity
		GO:0005245: voltage-gated calcium channel activity
#1083IR #1095IR #10IR	14	GO:0003712: transcription cofactor activity
		GO:0008301: DNA bending activity
		GO:0007015: actin filament organization
		GO:0017153: sodium:dicarboxylate symporter activity
		GO:0006835: dicarboxylic acid transport
		GO:0005070: SH3/SH2 adaptor activity
		GO:0003777: microtubule motor activity
		GO:0015813: L-glutamate transport
		GO:0040007: growth
		GO:0007126: meiosis
		GO:0048468: cell development
		GO:0007286: spermatid development
		GO:0003785: actin monomer binding
		GO:0031145: anaphase-promoting complex-dependent proteasomal ubiquitin-dependent protein catabolic process
#1051IR #1095IR #10IR	24	GO:0046627: negative regulation of insulin receptor signaling pathway
		GO:0016820: hydrolase activity, acting on acid anhydrides, catalyzing transmembrane movement of substances
		GO:0014826: vein smooth muscle contraction
		GO:0003707: steroid hormone receptor activity
		GO:0006754: ATP biosynthetic process
		GO:0045719: negative regulation of glycogen biosynthetic process
		GO:0047429: nucleoside-triphosphate diphosphatase activity
		GO:0043034: costamere
		GO:0045722: positive regulation of gluconeogenesis
		GO:0050427: 3'-phosphoadenosine 5'-phosphosulfate metabolic process
		GO:0009143: nucleoside triphosphate catabolic process
		GO:0050909: sensory perception of taste
		GO:0030730: sequestering of triglyceride
		GO:0001600: endothelin-B receptor activity
		GO:0009187: cyclic nucleotide metabolic process
		GO:0006885: regulation of pH
		GO:0006367: transcription initiation from RNA polymerase II promoter
		GO:0030643: cellular phosphate ion homeostasis
		GO:0050681: androgen receptor binding
		GO:0030374: ligand-dependent nuclear receptor transcription coactivator activity
		GO:0007187: G-protein signaling, coupled to cyclic nucleotide second messenger
		GO:0007368: determination of left/right symmetry

		GO:0016455: RNA polymerase II transcription mediator activity
		GO:0046325: negative regulation of glucose import
#1063IR #1095IR #10IR	304	GO:0048705: skeletal system morphogenesis
		GO:0043184: vascular endothelial growth factor receptor 2 binding
		GO:0045787: positive regulation of cell cycle
		GO:0032587: ruffle membrane
		GO:0001942: hair follicle development
		GO:0001540: beta-amyloid binding
		GO:0005770: late endosome
		GO:0043536: positive regulation of blood vessel endothelial cell migration
		GO:0043204: perikaryon
		GO:0019221: cytokine-mediated signaling pathway
		GO:0032355: response to estradiol stimulus
		GO:0005159: insulin-like growth factor receptor binding
		GO:0051291: protein heterooligomerization
		GO:0043627: response to estrogen stimulus
		GO:0042626: ATPase activity, coupled to transmembrane movement of substances
		GO:0032874: positive regulation of stress-activated MAPK cascade
		GO:0016032: viral reproduction
		GO:0000902: cell morphogenesis
		GO:0046983: protein dimerization activity
		GO:0008624: induction of apoptosis by extracellular signals
		GO:0005922: connexon complex
		GO:0006688: glycosphingolipid biosynthetic process
		GO:0031902: late endosome membrane
		GO:0005759: mitochondrial matrix
		GO:0008236: serine-type peptidase activity
		GO:0004197: cysteine-type endopeptidase activity
		GO:0043231: intracellular membrane-bounded organelle
		GO:0006911: phagocytosis, engulfment
		GO:0008009: chemokine activity
		GO:0008415: acyltransferase activity
		GO:0005778: peroxisomal membrane
		GO:0006805: xenobiotic metabolic process
		GO:0005952: cAMP-dependent protein kinase complex
		GO:0030425: dendrite
		GO:0043120: tumor necrosis factor binding
		GO:0006695: cholesterol biosynthetic process
		GO:0008603: cAMP-dependent protein kinase regulator activity
		GO:0042517: positive regulation of tyrosine phosphorylation of Stat3 protein
		GO:0008147: structural constituent of bone
		GO:0048741: skeletal muscle fiber development
		GO:0001937: negative regulation of endothelial cell proliferation
		GO:0016702: oxidoreductase activity, acting on single donors with incorporation of molecular oxygen, incorporation of two atoms of oxygen
		GO:0007173: epidermal growth factor receptor signaling pathway
		GO:0015333: peptide:hydrogen symporter activity
		GO:0043434: response to peptide hormone stimulus
		GO:0006112: energy reserve metabolic process
		GO:0050905: neuromuscular process
		GO:0000776: kinetochore
		GO:0031557: induction of programmed cell death in response to chemical stimulus
		GO:0043542: endothelial cell migration
		GO:0044262: cellular carbohydrate metabolic process
		GO:0042755: eating behavior
		GO:0009617: response to bacterium
		GO:0046330: positive regulation of JNK cascade
		GO:0001837: epithelial to mesenchymal transition
		GO:0007435: salivary gland morphogenesis
		GO:0030307: positive regulation of cell growth
		GO:0048011: nerve growth factor receptor signaling pathway
		GO:0006919: activation of caspase activity
		GO:0001889: liver development
		GO:0031290: retinal ganglion cell axon guidance
		GO:0050900: leukocyte migration
		GO:0004623: phospholipase A2 activity
		GO:0008017: microtubule binding
		GO:0001541: ovarian follicle development
		GO:0006605: protein targeting
		GO:0001654: eye development
		GO:0019903: protein phosphatase binding
		GO:0005768: endosome
		GO:0043524: negative regulation of neuron apoptosis
		GO:0004568: chitinase activity
		GO:0031069: hair follicle morphogenesis
		GO:0019228: regulation of action potential in neuron
		GO:0008305: integrin complex
		GO:0004672: protein kinase activity
		GO:0008286: insulin receptor signaling pathway
		GO:0007190: activation of adenylate cyclase activity
		GO:0007204: elevation of cytosolic calcium ion concentration
		GO:0005771: multivesicular body
		GO:0005160: transforming growth factor beta receptor binding
		GO:0017046: peptide hormone binding
		GO:0016050: vesicle organization
		GO:0001968: fibronectin binding
		GO:0051726: regulation of cell cycle
		GO:0048168: regulation of neuronal synaptic plasticity
		GO:0042325: regulation of phosphorylation
		GO:0015175: neutral amino acid transmembrane transporter activity
		GO:0005089: Rho guanyl-nucleotide exchange factor activity
		GO:0005758: mitochondrial intermembrane space
		GO:0015297: antiporter activity
		GO:0030900: forebrain development
		GO:0045892: negative regulation of transcription, DNA-dependent
		GO:0016504: peptidase activator activity
		GO:0035094: response to nicotine
		GO:0016264: gap junction assembly
		GO:0016500: protein-hormone receptor activity
		GO:0006935: chemotaxis
		GO:0042110: T cell activation
		GO:0043525: positive regulation of neuron apoptosis
		GO:0006633: fatty acid biosynthetic process
		GO:0051384: response to glucocorticoid stimulus
		GO:0006643: membrane lipid metabolic process
		GO:0003702: RNA polymerase II transcription factor activity
		GO:0006663: platelet activating factor biosynthetic process
		GO:0042605: peptide antigen binding
		GO:0010718: positive regulation of epithelial to mesenchymal transition
		GO:0050731: positive regulation of peptidyl-tyrosine phosphorylation
		GO:0004497: monooxygenase activity
		GO:0045726: positive regulation of integrin biosynthetic process
		GO:0006220: pyrimidine nucleotide metabolic process
		GO:0047555: 3',5'-cyclic-GMP phosphodiesterase activity
		GO:0030170: pyridoxal phosphate binding
		GO:0031418: L-ascorbic acid binding
		GO:0006809: nitric oxide biosynthetic process
		GO:0001502: cartilage condensation
		GO:0006974: response to DNA damage stimulus
		GO:0060271: cilium morphogenesis
		GO:0050778: positive regulation of immune response
		GO:0019959: interleukin-8 binding
		GO:0006892: post-Golgi vesicle-mediated transport
		GO:0031295: T cell costimulation
		GO:0031966: mitochondrial membrane
		GO:0010693: negative regulation of alkaline phosphatase activity
		GO:0019966: interleukin-1 binding
		GO:0009409: response to cold

GO:0050777: negative regulation of immune response
GO:0016209: antioxidant activity
GO:0042542: response to hydrogen peroxide
GO:0060389: pathway-restricted SMAD protein phosphorylation
GO:0015334: high affinity oligopeptide transporter activity
GO:0051091: positive regulation of transcription factor activity
GO:0043169: cation binding
GO:0005868: cytoplasmic dynein complex
GO:0019216: regulation of lipid metabolic process
GO:0007214: gamma-aminobutyric acid signaling pathway
GO:0030315: T-tubule
GO:0007215: glutamate signaling pathway
GO:0030182: neuron differentiation
GO:0051891: positive regulation of cardioblast differentiation
GO:0000075: cell cycle checkpoint
GO:0007283: spermatogenesis
GO:0005802: trans-Golgi network
GO:0004089: carbonate dehydratase activity
GO:0022601: menstrual cycle phase
GO:0051018: protein kinase A binding
GO:0016829: lyase activity
GO:0043531: ADP binding
GO:0001938: positive regulation of endothelial cell proliferation
GO:0019430: removal of superoxide radicals
GO:0048678: response to axon injury
GO:0005520: insulin-like growth factor binding
GO:0010575: positive regulation vascular endothelial growth factor production
GO:0048663: neuron fate commitment
GO:0008430: selenium binding
GO:0019233: sensory perception of pain
GO:0043066: negative regulation of apoptosis
GO:0006107: oxaloacetate metabolic process
GO:0009615: response to virus
GO:0005916: fascia adherens
GO:0051258: protein polymerization
GO:0006879: cellular iron ion homeostasis
GO:0005003: ephrin receptor activity
GO:0016798: hydrolase activity, acting on glycosyl bonds
GO:0048103: somatic stem cell division
GO:0043499: eukaryotic cell surface binding
GO:0008146: sulfotransferase activity
GO:0007585: respiratory gaseous exchange
GO:0016568: chromatin modification
GO:0045296: cadherin binding
GO:0006032: chitin catabolic process
GO:0009636: response to toxin
GO:0051402: neuron apoptosis
GO:0007623: circadian rhythm
GO:0015893: drug transport
GO:0005777: peroxisome
GO:0009966: regulation of signal transduction
GO:0008021: synaptic vesicle
GO:0045669: positive regulation of osteoblast differentiation
GO:0048172: regulation of short-term neuronal synaptic plasticity
GO:0000187: activation of MAPK activity
GO:0043195: terminal button
GO:0030552: cAMP binding
GO:0005925: focal adhesion
GO:0005938: cell cortex
GO:0004965: GABA-B receptor activity
GO:0043027: caspase inhibitor activity
GO:0032570: response to progesterone stimulus
GO:0031012: extracellular matrix
GO:0048545: response to steroid hormone stimulus
GO:0005743: mitochondrial inner membrane
GO:0051084: 'de novo' posttranslational protein folding
GO:0003828: alpha-N-acetylneuraminate alpha-2,8-sialyltransferase activity
GO:0050729: positive regulation of inflammatory response
GO:0043154: negative regulation of caspase activity
GO:0005765: lysosomal membrane
GO:0045893: positive regulation of transcription, DNA-dependent
GO:0051592: response to calcium ion
GO:0006006: glucose metabolic process
GO:0045732: positive regulation of protein catabolic process
GO:0005381: iron ion transmembrane transporter activity
GO:0030097: hemopoiesis
GO:0008144: drug binding
GO:0019953: sexual reproduction
GO:0005114: type II transforming growth factor beta receptor binding
GO:0033630: positive regulation of cell adhesion mediated by integrin
GO:0004143: diacylglycerol kinase activity
GO:0006464: protein modification process
GO:0031965: nuclear membrane
GO:0016922: ligand-dependent nuclear receptor binding
GO:0004046: aminoacylase activity
GO:0015467: G-protein activated inward rectifier potassium channel activity
GO:0015075: ion transmembrane transporter activity
GO:0006469: negative regulation of protein kinase activity
GO:0001947: heart looping
GO:0008076: voltage-gated potassium channel complex
GO:0045454: cell redox homeostasis
GO:0010634: positive regulation of epithelial cell migration
GO:0005080: protein kinase C binding
GO:0034097: response to cytokine stimulus
GO:0007010: cytoskeleton organization
GO:0000785: chromatin
GO:0050680: negative regulation of epithelial cell proliferation
GO:0007265: Ras protein signal transduction
GO:0001666: response to hypoxia
GO:0009395: phospholipid catabolic process
GO:0042416: dopamine biosynthetic process
GO:0005905: coated pit
GO:0070064: proline-rich region binding
GO:0009749: response to glucose stimulus
GO:0006396: RNA processing
GO:0006749: glutathione metabolic process
GO:0060317: cardiac epithelial to mesenchymal transition
GO:0008028: monocarboxylic acid transmembrane transporter activity
GO:0045778: positive regulation of ossification
GO:0006096: glycolysis
GO:0048666: neuron development
GO:0004702: receptor signaling protein serine/threonine kinase activity
GO:0008237: metallopeptidase activity
GO:0031016: pancreas development
GO:0060038: cardiac muscle cell proliferation
GO:0048754: branching morphogenesis of a tube
GO:0000165: MAPKKK cascade
GO:0010038: response to metal ion
GO:0050796: regulation of insulin secretion
GO:0040014: regulation of multicellular organism growth
GO:0030593: neutrophil chemotaxis
GO:0048813: dendrite morphogenesis
GO:0005198: structural molecule activity
GO:0030496: midbody
GO:0004177: aminopeptidase activity
GO:0043043: peptide biosynthetic process
GO:0000079: regulation of cyclin-dependent protein kinase activity

		GO:0005635: nuclear envelope
		GO:0032909: regulation of transforming growth factor-beta2 production
		GO:0006281: DNA repair
		GO:0060021: palate development
		GO:0035023: regulation of Rho protein signal transduction
		GO:0032147: activation of protein kinase activity
		GO:0015804: neutral amino acid transport
		GO:0000084: S phase of mitotic cell cycle
		GO:0042060: wound healing
		GO:0007154: cell communication
		GO:0006909: phagocytosis
		GO:0007050: cell cycle arrest
		GO:0005741: mitochondrial outer membrane
		GO:0051219: phosphoprotein binding
		GO:0005764: lysosome
		GO:0031901: early endosome membrane
		GO:0043200: response to amino acid stimulus
		GO:0050770: regulation of axonogenesis
		GO:0046320: regulation of fatty acid oxidation
		GO:0016567: protein ubiquitination
		GO:0045766: positive regulation of angiogenesis
		GO:0006366: transcription from RNA polymerase II promoter
		GO:0048469: cell maturation
		GO:0008373: sialyltransferase activity
		GO:0022857: transmembrane transporter activity
		GO:0008203: cholesterol metabolic process
		GO:0008509: anion transmembrane transporter activity
		GO:0030949: positive regulation of vascular endothelial growth factor receptor signaling pathway
		GO:0030168: platelet activation
		GO:0008656: caspase activator activity
		GO:0044267: cellular protein metabolic process
		GO:0045471: response to ethanol
		GO:0009612: response to mechanical stimulus
		GO:0030100: regulation of endocytosis
		GO:0016564: transcription repressor activity
		GO:0007588: excretion
		GO:0006631: fatty acid metabolic process
		GO:0045823: positive regulation of heart contraction
		GO:0007040: lysosome organization
		GO:0006139: nucleobase, nucleoside, nucleotide and nucleic acid metabolic process
		GO:0030890: positive regulation of B cell proliferation
		GO:0008347: glial cell migration
		GO:0007389: pattern specification process
		GO:0019825: oxygen binding
		GO:0000777: condensed chromosome kinetochore
		GO:0004857: enzyme inhibitor activity
		GO:0048699: generation of neurons
		GO:0051795: positive regulation of catagen
		GO:0042593: glucose homeostasis
		GO:0000082: G1/S transition of mitotic cell cycle
		GO:0007205: activation of protein kinase C activity by G-protein coupled receptor protein signaling pathway
		GO:0051899: membrane depolarization
#1063IR #1083IR #10IR	21	GO:0030672: synaptic vesicle membrane
		GO:0017157: regulation of exocytosis
		GO:0005606: laminin-1 complex
		GO:0042130: negative regulation of T cell proliferation
		GO:0006898: receptor-mediated endocytosis
		GO:0005902: microvillus
		GO:0022891: substrate-specific transmembrane transporter activity
		GO:0045184: establishment of protein localization
		GO:0017075: syntaxin-1 binding
		GO:0005626: insoluble fraction
		GO:0000786: nucleosome
		GO:0042393: histone binding
		GO:0055085: transmembrane transport
		GO:0048843: negative regulation of axon extension involved in axon guidance
		GO:0004115: 3',5'-cyclic-AMP phosphodiesterase activity
		GO:0050679: positive regulation of epithelial cell proliferation
		GO:0014069: postsynaptic density
		GO:0007398: ectoderm development
		GO:0005546: phosphatidylinositol-4,5-bisphosphate binding
		GO:0043010: camera-type eye development
		GO:0042383: sarcolemma
#1051IR #1063IR #10IR	25	GO:0005319: lipid transporter activity
		GO:0004889: nicotinic acetylcholine-activated cation-selective channel activity
		GO:0050821: protein stabilization
		GO:0008013: beta-catenin binding
		GO:0006940: regulation of smooth muscle contraction
		GO:0005892: nicotinic acetylcholine-gated receptor-channel complex
		GO:0019898: extrinsic to membrane
		GO:0030178: negative regulation of Wnt receptor signaling pathway
		GO:0034394: protein localization at cell surface
		GO:0005178: integrin binding
		GO:0030863: cortical cytoskeleton
		GO:0015171: amino acid transmembrane transporter activity
		GO:0015464: acetylcholine receptor activity
		GO:0045597: positive regulation of cell differentiation
		GO:0005912: adherens junction
		GO:0006865: amino acid transport
		GO:0006953: acute-phase response
		GO:0003730: mRNA 3'-UTR binding
		GO:0030336: negative regulation of cell migration
		GO:0005581: collagen
		GO:0060079: regulation of excitatory postsynaptic membrane potential
		GO:0007611: learning or memory
		GO:0007005: mitochondrion organization
		GO:0009312: oligosaccharide biosynthetic process
		GO:0042157: lipoprotein metabolic process
#1051IR #1083IR #1095IR	13	GO:0055114: oxidation reduction
		GO:0003700: transcription factor activity
		GO:0005545: phosphatidylinositol binding
		GO:0006486: protein amino acid glycosylation
		GO:0007517: muscle development
		GO:0006468: protein amino acid phosphorylation
		GO:0007242: intracellular signaling cascade
		GO:0006350: transcription
		GO:0006470: protein amino acid dephosphorylation
		GO:0030955: potassium ion binding
		GO:0007416: synaptogenesis
		GO:0019941: modification-dependent protein catabolic process
		GO:0004653: polypeptide N-acetylgalactosaminyltransferase activity
#1063IR #1083IR #1095IR	26	GO:0016327: apicolateral plasma membrane
		GO:0050860: negative regulation of T cell receptor signaling pathway
		GO:0006690: icosanoid metabolic process
		GO:0005042: netrin receptor activity
		GO:0006310: DNA recombination
		GO:0051233: spindle midzone
		GO:0008585: female gonad development
		GO:0005100: Rho GTPase activator activity
		GO:0001890: placenta development
		GO:0009566: fertilization
		GO:0001756: somitogenesis
		GO:0005095: GTPase inhibitor activity
		GO:0006914: autophagy
		GO:0018298: protein-chromophore linkage
		GO:0007269: neurotransmitter secretion
		GO:0005884: actin filament

		GO:0003950: NAD+ ADP-ribosyltransferase activity
		GO:0008218: bioluminescence
		GO:0019896: axon transport of mitochondrion
		GO:0042813: Wnt receptor activity
		GO:0030509: BMP signaling pathway
		GO:0019079: viral genome replication
		GO:0050885: neuromuscular process controlling balance
		GO:0009311: oligosaccharide metabolic process
		GO:0001504: neurotransmitter uptake
		GO:0043205: fibril
#1051IR #1063IR #1095IR	28	GO:0007219: Notch signaling pathway
		GO:0001775: cell activation
		GO:0016310: phosphorylation
		GO:0043569: negative regulation of insulin-like growth factor receptor signaling pathway
		GO:0018108: peptidyl-tyrosine phosphorylation
		GO:0008499: UDP-galactose:beta-N-acetylglucosamine beta-1,3-galactosyltransferase activity
		GO:0017166: vinculin binding
		GO:0006559: L-phenylalanine catabolic process
		GO:0045028: purinergic nucleotide receptor activity, G-protein coupled
		GO:0045165: cell fate commitment
		GO:0007271: synaptic transmission, cholinergic
		GO:0005793: ER-Golgi intermediate compartment
		GO:0048407: platelet-derived growth factor binding
		GO:0030318: melanocyte differentiation
		GO:0001542: ovulation from ovarian follicle
		GO:0005249: voltage-gated potassium channel activity
		GO:0007422: peripheral nervous system development
		GO:0000155: two-component sensor activity
		GO:0045740: positive regulation of DNA replication
		GO:0008360: regulation of cell shape
		GO:0005179: hormone activity
		GO:0005018: platelet-derived growth factor alpha-receptor activity
		GO:0017148: negative regulation of translation
		GO:0004890: GABA-A receptor activity
		GO:0048146: positive regulation of fibroblast proliferation
		GO:0005328: neurotransmitter:sodium symporter activity
		GO:0006572: tyrosine catabolic process
		GO:0015269: calcium-activated potassium channel activity
#1095IR #10IR	137	GO:0031227: intrinsic to endoplasmic reticulum membrane
		GO:0006656: phosphatidylcholine biosynthetic process
		GO:0015078: hydrogen ion transmembrane transporter activity
		GO:0048538: thymus development
		GO:0045860: positive regulation of protein kinase activity
		GO:0006108: malate metabolic process
		GO:0030330: DNA damage response, signal transduction by p53 class mediator
		GO:0004540: ribonuclease activity
		GO:0006094: gluconeogenesis
		GO:0019001: guanyl nucleotide binding
		GO:0009650: UV protection
		GO:0005388: calcium-transporting ATPase activity
		GO:0007252: I-kappaB phosphorylation
		GO:0004004: ATP-dependent RNA helicase activity
		GO:0007188: G-protein signaling, coupled to cAMP nucleotide second messenger
		GO:0032963: collagen metabolic process
		GO:0004386: helicase activity
		GO:0030101: natural killer cell activation
		GO:0045859: regulation of protein kinase activity
		GO:0030176: integral to endoplasmic reticulum membrane
		GO:0004322: ferroxidase activity
		GO:0042612: MHC class I protein complex
		GO:0019439: aromatic compound catabolic process
		GO:0030553: cGMP binding
		GO:0006222: UMP biosynthetic process
		GO:0004364: glutathione transferase activity
		GO:0016070: RNA metabolic process
		GO:0002474: antigen processing and presentation of peptide antigen via MHC class I
		GO:0005834: heterotrimeric G-protein complex
		GO:0008544: epidermis development
		GO:0015992: proton transport
		GO:0008320: protein transmembrane transporter activity
		GO:0000186: activation of MAPKK activity
		GO:0005097: Rab GTPase activator activity
		GO:0042645: mitochondrial nucleoid
		GO:0032313: regulation of Rab GTPase activity
		GO:0016461: unconventional myosin complex
		GO:0030867: rough endoplasmic reticulum membrane
		GO:0005391: sodium:potassium-exchanging ATPase activity
		GO:0005641: nuclear envelope lumen
		GO:0006164: purine nucleotide biosynthetic process
		GO:0048873: homeostasis of number of cells within a tissue
		GO:0001935: endothelial cell proliferation
		GO:0030216: keratinocyte differentiation
		GO:0045088: regulation of innate immune response
		GO:0000502: proteasome complex
		GO:0006509: membrane protein ectodomain proteolysis
		GO:0031941: filamentous actin
		GO:0000303: response to superoxide
		GO:0004104: cholinesterase activity
		GO:0043457: regulation of cellular respiration
		GO:0007565: female pregnancy
		GO:0016779: nucleotidyltransferase activity
		GO:0019861: flagellum
		GO:0031424: keratinization
		GO:0060001: minus-end directed microfilament motor activity
		GO:0007405: neuroblast proliferation
		GO:0051216: cartilage development
		GO:0006210: thymine catabolic process
		GO:0015701: bicarbonate transport
		GO:0006212: uracil catabolic process
		GO:0006825: copper ion transport
		GO:0014850: response to muscle activity
		GO:0015718: monocarboxylic acid transport
		GO:0004180: carboxypeptidase activity
		GO:0005452: inorganic anion exchanger activity
		GO:0048477: oogenesis
		GO:0014003: oligodendrocyte development
		GO:0043085: positive regulation of catalytic activity
		GO:0046920: alpha(1,3)-fucosyltransferase activity
		GO:0005247: voltage-gated chloride channel activity
		GO:0006986: response to unfolded protein
		GO:0019370: leukotriene biosynthetic process
		GO:0006214: thymidine catabolic process
		GO:0042254: ribosome biogenesis
		GO:0031638: zymogen activation
		GO:0045334: clathrin-coated endocytic vesicle
		GO:0016881: acid-amino acid ligase activity
		GO:0006417: regulation of translation
		GO:0005788: endoplasmic reticulum lumen
		GO:0010181: FMN binding
		GO:0006821: chloride transport
		GO:0004385: guanylate kinase activity
		GO:0016747: transferase activity, transferring acyl groups other than amino-acyl groups
		GO:0046870: cadmium ion binding
		GO:0008199: ferric iron binding
		GO:0016493: C-C chemokine receptor activity
		GO:0005504: fatty acid binding
		GO:0051539: 4 iron, 4 sulfur cluster binding

		GO:0005929: cilium
		GO:0004181: metallocarboxypeptidase activity
		GO:0032722: positive regulation of chemokine production
		GO:0006635: fatty acid beta-oxidation
		GO:0051046: regulation of secretion
		GO:0060087: relaxation of vascular smooth muscle
		GO:0046688: response to copper ion
		GO:0043588: skin development
		GO:0046873: metal ion transmembrane transporter activity
		GO:0008026: ATP-dependent helicase activity
		GO:0007341: penetration of zona pellucida
		GO:0032393: MHC class I receptor activity
		GO:0004866: endopeptidase inhibitor activity
		GO:0006207: 'de novo' pyrimidine base biosynthetic process
		GO:0009607: response to biotic stimulus
		GO:0006145: purine base catabolic process
		GO:0060173: limb development
		GO:0007254: JNK cascade
		GO:0019695: choline metabolic process
		GO:0006875: cellular metal ion homeostasis
		GO:0007612: learning
		GO:0042355: L-fucose catabolic process
		GO:0004693: cyclin-dependent protein kinase activity
		GO:0006099: tricarboxylic acid cycle
		GO:0030048: actin filament-based movement
		GO:0030520: estrogen receptor signaling pathway
		GO:0016591: DNA-directed RNA polymerase II, holoenzyme
		GO:0017113: dihydropyrimidine dehydrogenase (NADP+) activity
		GO:0007598: blood coagulation, extrinsic pathway
		GO:0045919: positive regulation of cytolysis
		GO:0050783: cocaine metabolic process
		GO:0006880: intracellular sequestering of iron ion
		GO:0002526: acute inflammatory response
		GO:0035035: histone acetyltransferase binding
		GO:0000302: response to reactive oxygen species
		GO:0051607: defense response to virus
		GO:0004185: serine-type carboxypeptidase activity
		GO:0005375: copper ion transmembrane transporter activity
		GO:0042102: positive regulation of T cell proliferation
		GO:0015501: glutamate:sodium symporter activity
		GO:0001533: cornified envelope
		GO:0006541: glutamine metabolic process
		GO:0008510: sodium:bicarbonate symporter activity
		GO:0003945: N-acetyllactosamine synthase activity
		GO:0019882: antigen processing and presentation
		GO:0008043: intracellular ferritin complex
		GO:0006783: heme biosynthetic process
		GO:0004158: dihydroorotate oxidase activity
#1083IR #10IR	21	GO:0030957: Tat protein binding
		GO:0032420: stereocilium
		GO:0016595: glutamate binding
		GO:0030274: LIM domain binding
		GO:0016524: latrotoxin receptor activity
		GO:0016316: phosphatidylinositol-3,4-bisphosphate 4-phosphatase activity
		GO:0042742: defense response to bacterium
		GO:0045109: intermediate filament organization
		GO:0005903: brush border
		GO:0015180: L-alanine transmembrane transporter activity
		GO:0048808: male genitalia morphogenesis
		GO:0005871: kinesin complex
		GO:0050890: cognition
		GO:0032279: asymmetric synapse
		GO:0042113: B cell activation
		GO:0015808: L-alanine transport
		GO:0005212: structural constituent of eye lens
		GO:0008093: cytoskeletal adaptor activity
		GO:0032387: negative regulation of intracellular transport
		GO:0005314: high-affinity glutamate transmembrane transporter activity
		GO:0005876: spindle microtubule
#1051IR #10IR	15	GO:0008527: taste receptor activity
		GO:0001678: cellular glucose homeostasis
		GO:0035066: positive regulation of histone acetylation
		GO:0046321: positive regulation of fatty acid oxidation
		GO:0045333: cellular respiration
		GO:0050873: brown fat cell differentiation
		GO:0005665: DNA-directed RNA polymerase II, core complex
		GO:0001659: temperature homeostasis
		GO:0005001: transmembrane receptor protein tyrosine phosphatase activity
		GO:0022904: respiratory electron transport chain
		GO:0004114: 3',5'-cyclic-nucleotide phosphodiesterase activity
		GO:0045295: gamma-catenin binding
		GO:0004949: cannabinoid receptor activity
		GO:0007610: behavior
		GO:0018149: peptide cross-linking
#1063IR #10IR	506	GO:0048812: neuron projection morphogenesis
		GO:0070374: positive regulation of ERK1 and ERK2 cascade
		GO:0010759: positive regulation of macrophage chemotaxis
		GO:0060070: canonical Wnt receptor signaling pathway
		GO:0032094: response to food
		GO:0030247: polysaccharide binding
		GO:0018279: protein N-linked glycosylation via asparagine
		GO:0019807: aspartoacylase activity
		GO:0006939: smooth muscle contraction
		GO:0008398: sterol 14-demethylase activity
		GO:0019432: triglyceride biosynthetic process
		GO:0070195: growth hormone receptor complex
		GO:0004903: growth hormone receptor activity
		GO:0002634: regulation of germinal center formation
		GO:0006928: cellular component movement
		GO:0006906: vesicle fusion
		GO:0060325: face morphogenesis
		GO:0048539: bone marrow development
		GO:0006325: chromatin organization
		GO:0017016: Ras GTPase binding
		GO:0035307: positive regulation of protein dephosphorylation
		GO:0046449: creatinine metabolic process
		GO:0008198: ferrous iron binding
		GO:0043101: purine-containing compound salvage
		GO:0042578: phosphoric ester hydrolase activity
		GO:0010553: negative regulation of gene-specific transcription from RNA polymerase II promoter
		GO:0071062: alphav-beta3 integrin-vitronectin complex
		GO:0005980: glycogen catabolic process
		GO:0046581: intercellular canaliculus
		GO:0007568: aging
		GO:0004833: tryptophan 2,3-dioxygenase activity
		GO:0001656: metanephros development
		GO:0021506: anterior neuropore closure
		GO:0050479: glyceryl-ether monooxygenase activity
		GO:0006569: tryptophan catabolic process
		GO:0050998: nitric-oxide synthase binding
		GO:0048514: blood vessel morphogenesis
		GO:0007517: muscle organ development
		GO:0050768: negative regulation of neurogenesis
		GO:0005901: caveola
		GO:0022409: positive regulation of cell-cell adhesion
		GO:0070836: caveola assembly
		GO:0002051: osteoblast fate commitment

GO:0043966: histone H3 acetylation
GO:0030177: positive regulation of Wnt receptor signaling pathway
GO:0043403: skeletal muscle tissue regeneration
GO:0070172: positive regulation of tooth mineralization
GO:0002709: regulation of T cell mediated immunity
GO:0002020: protease binding
GO:0017154: semaphorin receptor activity
GO:0010843: promoter binding
GO:0004459: L-lactate dehydrogenase activity
GO:0021766: hippocampus development
GO:0004445: inositol-polyphosphate 5-phosphatase activity
GO:0080025: phosphatidylinositol-3,5-bisphosphate binding
GO:0006776: vitamin A metabolic process
GO:0010596: negative regulation of endothelial cell migration
GO:0044236: multicellular organismal metabolic process
GO:0001676: long-chain fatty acid metabolic process
GO:0010951: negative regulation of endopeptidase activity
GO:0030147: natriuresis
GO:0045879: negative regulation of smoothened signaling pathway
GO:0042936: dipeptide transporter activity
GO:0045329: carnitine biosynthetic process
GO:0034755: iron ion transmembrane transport
GO:0004000: adenosine deaminase activity
GO:0010936: negative regulation of macrophage cytokine production
GO:0051591: response to cAMP
GO:0014049: positive regulation of glutamate secretion
GO:0004435: phosphatidylinositol phospholipase C activity
GO:0005773: vacuole
GO:0048312: intracellular distribution of mitochondria
GO:0007274: neuromuscular synaptic transmission
GO:0009168: purine ribonucleoside monophosphate biosynthetic process
GO:0030902: hindbrain development
GO:0050748: negative regulation of lipoprotein metabolic process
GO:0090090: negative regulation of canonical Wnt receptor signaling pathway
GO:0006351: transcription, DNA-dependent
GO:0007044: cell-substrate junction assembly
GO:0030133: transport vesicle
GO:0000216: M/G1 transition of mitotic cell cycle
GO:0032809: neuronal cell body membrane
GO:0004336: galactosylceramidase activity
GO:0070050: neuron homeostasis
GO:0071320: cellular response to cAMP
GO:0045124: regulation of bone resorption
GO:0010524: positive regulation of calcium ion transport into cytosol
GO:0006105: succinate metabolic process
GO:0043235: receptor complex
GO:0043278: response to morphine
GO:0006767: water-soluble vitamin metabolic process
GO:0043097: pyrimidine nucleoside salvage
GO:0033300: dehydroascorbic acid transporter activity
GO:0021879: forebrain neuron differentiation
GO:0032496: response to lipopolysaccharide
GO:0016010: dystrophin-associated glycoprotein complex
GO:0005262: calcium channel activity
GO:0015267: channel activity
GO:0090023: positive regulation of neutrophil chemotaxis
GO:0030818: negative regulation of cAMP biosynthetic process
GO:0070555: response to interleukin-1
GO:0003700: sequence-specific DNA binding transcription factor activity
GO:0002090: regulation of receptor internalization
GO:0051789: response to protein stimulus
GO:0048661: positive regulation of smooth muscle cell proliferation
GO:0032868: response to insulin stimulus
GO:0046638: positive regulation of alpha-beta T cell differentiation
GO:0050728: negative regulation of inflammatory response
GO:0002021: response to dietary excess
GO:0043679: axon terminus
GO:0034138: toll-like receptor 3 signaling pathway
GO:0060484: lung-associated mesenchyme development
GO:0019442: tryptophan catabolic process to acetyl-CoA
GO:0005154: epidermal growth factor receptor binding
GO:0030667: secretory granule membrane
GO:0031234: extrinsic to internal side of plasma membrane
GO:0006066: alcohol metabolic process
GO:0001934: positive regulation of protein phosphorylation
GO:0006521: regulation of cellular amino acid metabolic process
GO:0031995: insulin-like growth factor II binding
GO:0006520: cellular amino acid metabolic process
GO:0009166: nucleotide catabolic process
GO:0032369: negative regulation of lipid transport
GO:0051545: negative regulation of elastin biosynthetic process
GO:0033555: multicellular organismal response to stress
GO:0032026: response to magnesium ion
GO:0010888: negative regulation of lipid storage
GO:0055056: D-glucose transmembrane transporter activity
GO:0016566: specific transcriptional repressor activity
GO:0004943: C3a anaphylatoxin receptor activity
GO:0000904: cell morphogenesis involved in differentiation
GO:0007266: Rho protein signal transduction
GO:0001958: endochondral ossification
GO:0046545: development of primary female sexual characteristics
GO:0016525: negative regulation of angiogenesis
GO:0042166: acetylcholine binding
GO:0043394: proteoglycan binding
GO:0045596: negative regulation of cell differentiation
GO:0005044: scavenger receptor activity
GO:0048167: regulation of synaptic plasticity
GO:0032755: positive regulation of interleukin-6 production
GO:0055037: recycling endosome
GO:0048487: beta-tubulin binding
GO:0043526: neuroprotection
GO:0090082: positive regulation of heart induction by negative regulation of canonical Wnt receptor signaling pathway
GO:0070491: repressing transcription factor binding
GO:0005088: Ras guanyl-nucleotide exchange factor activity
GO:0034372: very-low-density lipoprotein particle remodeling
GO:0008645: hexose transport
GO:0031258: lamellipodium membrane
GO:0032526: response to retinoic acid
GO:0051289: protein homotetramerization
GO:0048786: presynaptic active zone
GO:0034976: response to endoplasmic reticulum stress
GO:0042923: neuropeptide binding
GO:0043409: negative regulation of MAPKKK cascade
GO:0033391: chromatoid body
GO:0034599: cellular response to oxidative stress
GO:0015250: water channel activity
GO:0030901: midbrain development
GO:0006090: pyruvate metabolic process
GO:0060732: positive regulation of inositol phosphate biosynthetic process
GO:0004876: complement component C3a receptor activity
GO:0050918: positive chemotaxis
GO:0071377: cellular response to glucagon stimulus
GO:0031340: positive regulation of vesicle fusion
GO:0044130: negative regulation of growth of symbiont in host
GO:0051149: positive regulation of muscle cell differentiation
GO:0006184: GTP catabolic process
GO:0008336: gamma-butyrobetaine dioxygenase activity

GO:0007416: synapse assembly
GO:0006766: vitamin metabolic process
GO:0032983: kainate selective glutamate receptor complex
GO:0048630: skeletal muscle tissue growth
GO:0007492: endoderm development
GO:0055086: nucleobase, nucleoside and nucleotide metabolic process
GO:0002430: complement receptor mediated signaling pathway
GO:0001850: complement component C3a binding
GO:0042692: muscle cell differentiation
GO:0051897: positive regulation of protein kinase B signaling cascade
GO:0032870: cellular response to hormone stimulus
GO:0014842: regulation of satellite cell proliferation
GO:0006644: phospholipid metabolic process
GO:0010002: cardioblast differentiation
GO:0015732: prostaglandin transport
GO:0001664: G-protein-coupled receptor binding
GO:0003680: AT DNA binding
GO:0042568: insulin-like growth factor binary complex
GO:0006101: citrate metabolic process
GO:0016043: cellular component organization
GO:0051259: protein oligomerization
GO:0016311: dephosphorylation
GO:0031781: type 3 melanocortin receptor binding
GO:0051403: stress-activated MAPK cascade
GO:0060055: angiogenesis involved in wound healing
GO:0046898: response to cycloheximide
GO:0048278: vesicle docking
GO:0045332: phospholipid translocation
GO:0050714: positive regulation of protein secretion
GO:0030819: positive regulation of cAMP biosynthetic process
GO:0030301: cholesterol transport
GO:0005502: 11-cis retinal binding
GO:0048755: branching morphogenesis of a nerve
GO:0006103: 2-oxoglutarate metabolic process
GO:0006533: aspartate catabolic process
GO:0006573: valine metabolic process
GO:0014824: artery smooth muscle contraction
GO:0001869: negative regulation of complement activation, lectin pathway
GO:0034186: apolipoprotein A-I binding
GO:0004806: triglyceride lipase activity
GO:0015186: L-glutamine transmembrane transporter activity
GO:0051234: establishment of localization
GO:0045542: positive regulation of cholesterol biosynthetic process
GO:0006730: one-carbon metabolic process
GO:0004850: uridine phosphorylase activity
GO:0060161: positive regulation of dopamine receptor signaling pathway
GO:0030855: epithelial cell differentiation
GO:0090050: positive regulation of cell migration involved in sprouting angiogenesis
GO:0035583: negative regulation of transforming growth factor beta receptor signaling pathway by extracellular sequestering of TGFbeta
GO:0004620: phospholipase activity
GO:0050776: regulation of immune response
GO:0034329: cell junction assembly
GO:0007613: memory
GO:0051593: response to folic acid
GO:0015101: organic cation transmembrane transporter activity
GO:0016044: cellular membrane organization
GO:0050660: flavin adenine dinucleotide binding
GO:0008081: phosphoric diester hydrolase activity
GO:0045715: negative regulation of low-density lipoprotein particle receptor biosynthetic process
GO:0060463: lung lobe morphogenesis
GO:0042136: neurotransmitter biosynthetic process
GO:0032760: positive regulation of tumor necrosis factor production
GO:0030073: insulin secretion
GO:0015132: prostaglandin transmembrane transporter activity
GO:0006853: carnitine shuttle
GO:0048566: embryonic digestive tract development
GO:0046485: ether lipid metabolic process
GO:0050804: regulation of synaptic transmission
GO:0015035: protein disulfide oxidoreductase activity
GO:0031333: negative regulation of protein complex assembly
GO:0005614: interstitial matrix
GO:0060337: type I interferon-mediated signaling pathway
GO:0008643: carbohydrate transport
GO:0048839: inner ear development
GO:0005545: 1-phosphatidylinositol binding
GO:0008543: fibroblast growth factor receptor signaling pathway
GO:0060346: bone trabecula formation
GO:0031224: intrinsic to membrane
GO:0060396: growth hormone receptor signaling pathway
GO:0055114: oxidation-reduction process
GO:0001933: negative regulation of protein phosphorylation
GO:0007088: regulation of mitosis
GO:0002092: positive regulation of receptor internalization
GO:0050891: multicellular organismal water homeostasis
GO:0043014: alpha-tubulin binding
GO:0042567: insulin-like growth factor ternary complex
GO:0001569: patterning of blood vessels
GO:0016597: amino acid binding
GO:0090303: positive regulation of wound healing
GO:0006486: protein glycosylation
GO:0010552: positive regulation of gene-specific transcription from RNA polymerase II promoter
GO:0035115: embryonic forelimb morphogenesis
GO:0009755: hormone-mediated signaling pathway
GO:0006069: ethanol oxidation
GO:0003987: acetate-CoA ligase activity
GO:0002040: sprouting angiogenesis
GO:0033267: axon part
GO:0042637: catagen
GO:0042895: antibiotic transporter activity
GO:0035116: embryonic hindlimb morphogenesis
GO:0007193: inhibition of adenylate cyclase activity by G-protein signaling pathway
GO:0001527: microfibril
GO:0030673: axolemma
GO:0051482: elevation of cytosolic calcium ion concentration involved in G-protein signaling coupled to IP3 second messenger
GO:0060174: limb bud formation
GO:0009117: nucleotide metabolic process
GO:0046135: pyrimidine nucleoside catabolic process
GO:0046427: positive regulation of JAK-STAT cascade
GO:0007512: adult heart development
GO:0014068: positive regulation of phosphatidylinositol 3-kinase cascade
GO:0047498: calcium-dependent phospholipase A2 activity
GO:0031398: positive regulation of protein ubiquitination
GO:0060445: branching involved in salivary gland morphogenesis
GO:0046426: negative regulation of JAK-STAT cascade
GO:0045671: negative regulation of osteoclast differentiation
GO:0051781: positive regulation of cell division
GO:0035313: wound healing, spreading of epidermal cells
GO:0032582: negative regulation of gene-specific transcription
GO:0046209: nitric oxide metabolic process
GO:0005791: rough endoplasmic reticulum
GO:0070935: 3'-UTR-mediated mRNA stabilization
GO:0019841: retinol binding
GO:0008202: steroid metabolic process
GO:0009952: anterior/posterior pattern formation
GO:0006468: protein phosphorylation
GO:0034220: ion transmembrane transport
GO:0001701: in utero embryonic development

GO:0031115: negative regulation of microtubule polymerization
GO:0008063: Toll signaling pathway
GO:0014070: response to organic cyclic compound
GO:0031782: type 4 melanocortin receptor binding
GO:0051205: protein insertion into membrane
GO:0010243: response to organic nitrogen
GO:0000209: protein polyubiquitination
GO:0005921: gap junction
GO:0016079: synaptic vesicle exocytosis
GO:0045885: positive regulation of survival gene product expression
GO:0005242: inward rectifier potassium channel activity
GO:0030278: regulation of ossification
GO:0034707: chloride channel complex
GO:0019433: triglyceride catabolic process
GO:0043084: penile erection
GO:0050830: defense response to Gram-positive bacterium
GO:0008528: peptide receptor activity, G-protein coupled
GO:0055065: metal ion homeostasis
GO:0010037: response to carbon dioxide
GO:0070588: calcium ion transmembrane transport
GO:0007597: blood coagulation, intrinsic pathway
GO:0070837: dehydroascorbic acid transport
GO:0004999: vasoactive intestinal polypeptide receptor activity
GO:0034134: toll-like receptor 2 signaling pathway
GO:0002686: negative regulation of leukocyte migration
GO:0046855: inositol phosphate dephosphorylation
GO:0043691: reverse cholesterol transport
GO:0060664: epithelial cell proliferation involved in salivary gland morphogenesis
GO:0001944: vasculature development
GO:0002224: toll-like receptor signaling pathway
GO:0008160: protein tyrosine phosphatase activator activity
GO:0034332: adherens junction organization
GO:0015030: Cajal body
GO:0048706: embryonic skeletal system development
GO:0030665: clathrin coated vesicle membrane
GO:0009268: response to pH
GO:0016941: natriuretic peptide receptor activity
GO:0004383: guanylate cyclase activity
GO:0006600: creatine metabolic process
GO:0033137: negative regulation of peptidyl-serine phosphorylation
GO:0031315: extrinsic to mitochondrial outer membrane
GO:0006154: adenosine catabolic process
GO:0006921: cellular component disassembly involved in apoptosis
GO:0004029: aldehyde dehydrogenase (NAD) activity
GO:0045765: regulation of angiogenesis
GO:0009790: embryo development
GO:0034142: toll-like receptor 4 signaling pathway
GO:0070256: negative regulation of mucus secretion
GO:0090263: positive regulation of canonical Wnt receptor signaling pathway
GO:0031783: type 5 melanocortin receptor binding
GO:0010842: retina layer formation
GO:0017091: AU-rich element binding
GO:0030514: negative regulation of BMP signaling pathway
GO:0006527: arginine catabolic process
GO:0000255: allantoin metabolic process
GO:0010745: negative regulation of macrophage derived foam cell differentiation
GO:0016529: sarcoplasmic reticulum
GO:0042220: response to cocaine
GO:0070365: hepatocyte differentiation
GO:0016328: lateral plasma membrane
GO:0034375: high-density lipoprotein particle remodeling
GO:0046847: filopodium assembly
GO:0005372: water transmembrane transporter activity
GO:0060666: dichotomous subdivision of terminal units involved in salivary gland branching
GO:0050678: regulation of epithelial cell proliferation
GO:0051549: positive regulation of keratinocyte migration
GO:0042977: activation of JAK2 kinase activity
GO:0045663: positive regulation of myoblast differentiation
GO:0033144: negative regulation of steroid hormone receptor signaling pathway
GO:0051924: regulation of calcium ion transport
GO:0005881: cytoplasmic microtubule
GO:0006182: cGMP biosynthetic process
GO:0007184: SMAD protein import into nucleus
GO:0051928: positive regulation of calcium ion transport
GO:0035050: embryonic heart tube development
GO:0002576: platelet degranulation
GO:0048701: embryonic cranial skeleton morphogenesis
GO:0004030: aldehyde dehydrogenase [NAD(P)+] activity
GO:0048014: Tie receptor signaling pathway
GO:0019534: toxin transporter activity
GO:0045844: positive regulation of striated muscle tissue development
GO:0006206: pyrimidine base metabolic process
GO:0000083: regulation of transcription involved in G1/S phase of mitotic cell cycle
GO:0002070: epithelial cell maturation
GO:0005083: small GTPase regulator activity
GO:0055091: phospholipid homeostasis
GO:0015867: ATP transport
GO:0014032: neural crest cell development
GO:0031748: D1 dopamine receptor binding
GO:0032580: Golgi cisterna membrane
GO:0015758: glucose transport
GO:0070207: protein homotrimerization
GO:0032332: positive regulation of chondrocyte differentiation
GO:0019441: tryptophan catabolic process to kynurenine
GO:0030850: prostate gland development
GO:0042487: regulation of odontogenesis of dentine-containing tooth
GO:0030136: clathrin-coated vesicle
GO:0003333: amino acid transmembrane transport
GO:0007168: receptor guanylyl cyclase signaling pathway
GO:0070742: C2H2 zinc finger domain binding
GO:0060916: mesenchymal cell proliferation involved in lung development
GO:0008179: adenylate cyclase binding
GO:0007029: endoplasmic reticulum organization
GO:0031622: positive regulation of fever generation
GO:0019530: taurine metabolic process
GO:0060548: negative regulation of cell death
GO:0014909: smooth muscle cell migration
GO:0060351: cartilage development involved in endochondral bone morphogenesis
GO:0033632: regulation of cell-cell adhesion mediated by integrin
GO:0030414: peptidase inhibitor activity
GO:0034641: cellular nitrogen compound metabolic process
GO:0010232: vascular transport
GO:0000922: spindle pole
GO:0060348: bone development
GO:0001599: endothelin-A receptor activity
GO:0035295: tube development
GO:0046108: uridine metabolic process
GO:0002053: positive regulation of mesenchymal cell proliferation
GO:0070295: renal water absorption
GO:0019905: syntaxin binding
GO:0048010: vascular endothelial growth factor receptor signaling pathway
GO:0007166: cell surface receptor linked signaling pathway
GO:0001649: osteoblast differentiation
GO:0070083: clathrin sculpted monoamine transport vesicle membrane
GO:0048557: embryonic digestive tract morphogenesis
GO:0031960: response to corticosteroid stimulus
GO:0060416: response to growth hormone stimulus

GO:0040036: regulation of fibroblast growth factor receptor signaling pathway
GO:0002755: MyD88-dependent toll-like receptor signaling pathway
GO:0030141: stored secretory granule
GO:0007162: negative regulation of cell adhesion
GO:0001883: purine nucleoside binding
GO:0006144: purine base metabolic process
GO:0055093: response to hyperoxia
GO:0030057: desmosome
GO:0046716: muscle cell homeostasis
GO:0010629: negative regulation of gene expression
GO:0031625: ubiquitin protein ligase binding
GO:0043237: laminin-1 binding
GO:0071438: invadopodium membrane
GO:0050750: low-density lipoprotein particle receptor binding
GO:0048568: embryonic organ development
GO:0010595: positive regulation of endothelial cell migration
GO:0005184: neuropeptide hormone activity
GO:0071398: cellular response to fatty acid
GO:0034199: activation of protein kinase A activity
GO:0010466: negative regulation of peptidase activity
GO:0006833: water transport
GO:0010628: positive regulation of gene expression
GO:0042277: peptide binding
GO:0010811: positive regulation of cell-substrate adhesion
GO:0010716: negative regulation of extracellular matrix disassembly
GO:0006549: isoleucine metabolic process
GO:0033574: response to testosterone stimulus
GO:0015695: organic cation transport
GO:0001657: ureteric bud development
GO:0033089: positive regulation of T cell differentiation in thymus
GO:0044255: cellular lipid metabolic process
GO:0007202: activation of phospholipase C activity
GO:0030210: heparin biosynthetic process
GO:0031594: neuromuscular junction
GO:0040018: positive regulation of multicellular organism growth
GO:0030325: adrenal gland development
GO:0004439: phosphatidylinositol-4,5-bisphosphate 5-phosphatase activity
GO:0002756: MyD88-independent toll-like receptor signaling pathway
GO:0003007: heart morphogenesis
GO:0042787: protein ubiquitination involved in ubiquitin-dependent protein catabolic process
GO:0060333: interferon-gamma-mediated signaling pathway
GO:0030501: positive regulation of bone mineralization
GO:0046856: phosphatidylinositol dephosphorylation
GO:0034704: calcium channel complex
GO:0005811: lipid particle
GO:0019800: peptide cross-linking via chondroitin 4-sulfate glycosaminoglycan
GO:0019933: cAMP-mediated signaling
GO:0035556: intracellular signal transduction
GO:0009954: proximal/distal pattern formation
GO:0005539: glycosaminoglycan binding
GO:0004175: endopeptidase activity
GO:0050881: musculoskeletal movement
GO:0002063: chondrocyte development
GO:0031092: platelet alpha granule membrane
GO:0048019: receptor antagonist activity
GO:0017137: Rab GTPase binding
GO:0043190: ATP-binding cassette (ABC) transporter complex
GO:0043116: negative regulation of vascular permeability
GO:0004465: lipoprotein lipase activity
GO:0045216: cell-cell junction organization
GO:0031175: neuron projection development
GO:0071346: cellular response to interferon-gamma
GO:0042562: hormone binding
GO:0003705: sequence-specific enhancer binding RNA polymerase II transcription factor activity
GO:0048870: cell motility
GO:0060523: prostate epithelial cord elongation
GO:0031780: adrenocorticotropin hormone receptor binding
GO:0006683: galactosylceramide catabolic process
GO:0005243: gap junction channel activity
GO:0090244: Wnt receptor signaling pathway involved in somitogenesis
GO:0005355: glucose transmembrane transporter activity
GO:0070996: type 1 melanocortin receptor binding
GO:0004962: endothelin receptor activity
GO:0034130: toll-like receptor 1 signaling pathway
GO:0048009: insulin-like growth factor receptor signaling pathway
GO:0051281: positive regulation of release of sequestered calcium ion into cytosol
GO:0042523: positive regulation of tyrosine phosphorylation of Stat5 protein
GO:0050955: thermoception
GO:0006012: galactose metabolic process
GO:0043025: neuronal cell body
GO:0051602: response to electrical stimulus
GO:0043252: sodium-independent organic anion transport
#1083IR #1095IR 49 GO:0006959: humoral immune response
GO:0021965: spinal cord ventral commissure morphogenesis
GO:0005840: ribosome
GO:0006414: translational elongation
GO:0030071: regulation of mitotic metaphase/anaphase transition
GO:0045843: negative regulation of striated muscle development
GO:0042255: ribosome assembly
GO:0032281: alpha-amino-3-hydroxy-5-methyl-4-isoxazolepropionic acid selective glutamate receptor complex
GO:0003705: RNA polymerase II transcription factor activity, enhancer binding
GO:0006228: UTP biosynthetic process
GO:0070330: aromatase activity
GO:0004065: arylsulfatase activity
GO:0043025: cell soma
GO:0007093: mitotic cell cycle checkpoint
GO:0004550: nucleoside diphosphate kinase activity
GO:0043198: dendritic shaft
GO:0030141: secretory granule
GO:0031014: troponin T binding
GO:0000090: mitotic anaphase
GO:0006493: protein amino acid O-linked glycosylation
GO:0007051: spindle organization
GO:0045494: photoreceptor cell maintenance
GO:0006183: GTP biosynthetic process
GO:0003735: structural constituent of ribosome
GO:0051017: actin filament bundle formation
GO:0030833: regulation of actin filament polymerization
GO:0051246: regulation of protein metabolic process
GO:0007163: establishment or maintenance of cell polarity
GO:0006928: cell motion
GO:0019843: rRNA binding
GO:0007569: cell aging
GO:0008089: anterograde axon cargo transport
GO:0045095: keratin filament
GO:0005680: anaphase-promoting complex
GO:0051437: positive regulation of ubiquitin-protein ligase activity during mitotic cell cycle
GO:0001919: regulation of receptor recycling
GO:0051436: negative regulation of ubiquitin-protein ligase activity during mitotic cell cycle
GO:0048341: paraxial mesoderm formation
GO:0005681: spliceosome
GO:0005861: troponin complex
GO:0006241: CTP biosynthetic process
GO:0021542: dentate gyrus development
GO:0009416: response to light stimulus
GO:0001891: phagocytic cup
GO:0051059: NF-kappaB binding

		GO:0022627: cytosolic small ribosomal subunit
		GO:0031175: neurite development
		GO:0006364: rRNA processing
		GO:0048015: phosphoinositide-mediated signaling
#1051IR #1095IR	30	GO:0048154: S100 beta binding
		GO:0008109: N-acetyllactosaminide beta-1,6-N-acetylglucosaminyltransferase activity
		GO:0042801: polo kinase kinase activity
		GO:0006944: membrane fusion
		GO:0008037: cell recognition
		GO:0050660: FAD binding
		GO:0034614: cellular response to reactive oxygen species
		GO:0034644: cellular response to UV
		GO:0007338: single fertilization
		GO:0006497: protein amino acid lipidation
		GO:0048066: pigmentation during development
		GO:0031402: sodium ion binding
		GO:0006957: complement activation, alternative pathway
		GO:0007497: posterior midgut development
		GO:0030594: neurotransmitter receptor activity
		GO:0030286: dynein complex
		GO:0042953: lipoprotein transport
		GO:0015662: ATPase activity, coupled to transmembrane movement of ions, phosphorylative mechanism
		GO:0031953: negative regulation of protein amino acid autophosphorylation
		GO:0046777: protein amino acid autophosphorylation
		GO:0005005: transmembrane-ephrin receptor activity
		GO:0000775: chromosome, centromeric region
		GO:0031404: chloride ion binding
		GO:0030866: cortical actin cytoskeleton organization
		GO:0001816: cytokine production
		GO:0042598: vesicular fraction
		GO:0007166: cell surface receptor linked signal transduction
		GO:0004864: phosphoprotein phosphatase inhibitor activity
		GO:0004411: homogentisate 1,2-dioxygenase activity
		GO:0006024: glycosaminoglycan biosynthetic process
#1063IR #1095IR	179	GO:0007089: traversing start control point of mitotic cell cycle
		GO:0003697: single-stranded DNA binding
		GO:0048245: eosinophil chemotaxis
		GO:0010033: response to organic substance
		GO:0019900: kinase binding
		GO:0009987: cellular process
		GO:0005796: Golgi lumen
		GO:0070309: lens fiber cell morphogenesis
		GO:0003690: double-stranded DNA binding
		GO:0004860: protein kinase inhibitor activity
		GO:0008242: omega peptidase activity
		GO:0021952: central nervous system projection neuron axonogenesis
		GO:0014059: regulation of dopamine secretion
		GO:0007176: regulation of epidermal growth factor receptor activity
		GO:0017147: Wnt-protein binding
		GO:0008584: male gonad development
		GO:0048845: venous blood vessel morphogenesis
		GO:0050662: coenzyme binding
		GO:0008467: [heparan sulfate]-glucosamine 3-sulfotransferase 1 activity
		GO:0051537: 2 iron, 2 sulfur cluster binding
		GO:0045182: translation regulator activity
		GO:0043130: ubiquitin binding
		GO:0021680: cerebellar Purkinje cell layer development
		GO:0035162: embryonic hemopoiesis
		GO:0050659: N-acetylgalactosamine 4-sulfate 6-O-sulfotransferase activity
		GO:0008195: phosphatidate phosphatase activity
		GO:0007566: embryo implantation
		GO:0021987: cerebral cortex development
		GO:0019915: lipid storage
		GO:0004879: ligand-dependent nuclear receptor activity
		GO:0001508: regulation of action potential
		GO:0032839: dendrite cytoplasm
		GO:0042246: tissue regeneration
		GO:0042989: sequestering of actin monomers
		GO:0030968: endoplasmic reticulum unfolded protein response
		GO:0008234: cysteine-type peptidase activity
		GO:0005740: mitochondrial envelope
		GO:0007595: lactation
		GO:0015914: phospholipid transport
		GO:0060298: positive regulation of sarcomere organization
		GO:0000718: nucleotide-excision repair, DNA damage removal
		GO:0048037: cofactor binding
		GO:0045737: positive regulation of cyclin-dependent protein kinase activity
		GO:0009313: oligosaccharide catabolic process
		GO:0050699: WW domain binding
		GO:0009306: protein secretion
		GO:0045429: positive regulation of nitric oxide biosynthetic process
		GO:0001974: blood vessel remodeling
		GO:0050730: regulation of peptidyl-tyrosine phosphorylation
		GO:0016853: isomerase activity
		GO:0007599: hemostasis
		GO:0032312: regulation of ARF GTPase activity
		GO:0043122: regulation of I-kappaB kinase/NF-kappaB cascade
		GO:0006302: double-strand break repair
		GO:0043407: negative regulation of MAP kinase activity
		GO:0006412: translation
		GO:0048027: mRNA 5'-UTR binding
		GO:0001725: stress fiber
		GO:0016049: cell growth
		GO:0031667: response to nutrient levels
		GO:0060042: retina morphogenesis in camera-type eye
		GO:0050811: GABA receptor binding
		GO:0060214: endocardium formation
		GO:0015026: coreceptor activity
		GO:0015807: L-amino acid transport
		GO:0009791: post-embryonic development
		GO:0030246: carbohydrate binding
		GO:0043406: positive regulation of MAP kinase activity
		GO:0008466: glycogenin glucosyltransferase activity
		GO:0006297: nucleotide-excision repair, DNA gap filling
		GO:0046620: regulation of organ growth
		GO:0048012: hepatocyte growth factor receptor signaling pathway
		GO:0005484: SNAP receptor activity
		GO:0021516: dorsal spinal cord development
		GO:0009410: response to xenobiotic stimulus
		GO:0008156: negative regulation of DNA replication
		GO:0031072: heat shock protein binding
		GO:0060009: Sertoli cell development
		GO:0007628: adult walking behavior
		GO:0005782: peroxisomal matrix
		GO:0006491: N-glycan processing
		GO:0032947: protein complex scaffold
		GO:0045768: positive regulation of anti-apoptosis
		GO:0004527: exonuclease activity
		GO:0008060: ARF GTPase activator activity
		GO:0042789: mRNA transcription from RNA polymerase II promoter
		GO:0008654: phospholipid biosynthetic process
		GO:0047485: protein N-terminus binding
		GO:0009437: carnitine metabolic process
		GO:0005008: hepatocyte growth factor receptor activity
		GO:0004221: ubiquitin thiolesterase activity
		GO:0030276: clathrin binding
		GO:0000036: acyl carrier activity

		GO:0030504: inorganic diphosphate transmembrane transporter activity
		GO:0006820: anion transport
		GO:0015015: heparan sulfate proteoglycan biosynthetic process, enzymatic modification
		GO:0004519: endonuclease activity
		GO:0031201: SNARE complex
		GO:0015271: outward rectifier potassium channel activity
		GO:0005978: glycogen biosynthetic process
		GO:0045351: type I interferon biosynthetic process
		GO:0030910: olfactory placode formation
		GO:0016758: transferase activity, transferring hexosyl groups
		GO:0019674: NAD metabolic process
		GO:0000723: telomere maintenance
		GO:0015459: potassium channel regulator activity
		GO:0016579: protein deubiquitination
		GO:0009058: biosynthetic process
		GO:0042326: negative regulation of phosphorylation
		GO:0004571: mannosyl-oligosaccharide 1,2-alpha-mannosidase activity
		GO:0042133: neurotransmitter metabolic process
		GO:0043049: otic placode formation
		GO:0001709: cell fate determination
		GO:0016790: thiolester hydrolase activity
		GO:0008210: estrogen metabolic process
		GO:0003955: NAD(P)H dehydrogenase (quinone) activity
		GO:0050930: induction of positive chemotaxis
		GO:0015923: mannosidase activity
		GO:0001568: blood vessel development
		GO:0030532: small nuclear ribonucleoprotein complex
		GO:0042056: chemoattractant activity
		GO:0033872: [heparan sulfate]-glucosamine 3-sulfotransferase 3 activity
		GO:0060041: retina development in camera-type eye
		GO:0008191: metalloendopeptidase inhibitor activity
		GO:0005819: spindle
		GO:0016538: cyclin-dependent protein kinase regulator activity
		GO:0046329: negative regulation of JNK cascade
		GO:0021522: spinal cord motor neuron differentiation
		GO:0006516: glycoprotein catabolic process
		GO:0016605: PML body
		GO:0019319: hexose biosynthetic process
		GO:0004869: cysteine-type endopeptidase inhibitor activity
		GO:0032940: secretion by cell
		GO:0021702: cerebellar Purkinje cell differentiation
		GO:0051879: Hsp90 protein binding
		GO:0019864: IgG binding
		GO:0004128: cytochrome-b5 reductase activity
		GO:0000076: DNA replication checkpoint
		GO:0005801: cis-Golgi network
		GO:0008503: benzodiazepine receptor activity
		GO:0003993: acid phosphatase activity
		GO:0006665: sphingolipid metabolic process
		GO:0047391: alkylglycerophosphoethanolamine phosphodiesterase activity
		GO:0000118: histone deacetylase complex
		GO:0005519: cytoskeletal regulatory protein binding
		GO:0021915: neural tube development
		GO:0016126: sterol biosynthetic process
		GO:0004553: hydrolase activity, hydrolyzing O-glycosyl compounds
		GO:0045807: positive regulation of endocytosis
		GO:0015179: L-amino acid transmembrane transporter activity
		GO:0060076: excitatory synapse
		GO:0042035: regulation of cytokine biosynthetic process
		GO:0008565: protein transporter activity
		GO:0035264: multicellular organism growth
		GO:0032769: negative regulation of monooxygenase activity
		GO:0019956: chemokine binding
		GO:0034341: response to interferon-gamma
		GO:0046928: regulation of neurotransmitter secretion
		GO:0017053: transcriptional repressor complex
		GO:0005315: inorganic phosphate transmembrane transporter activity
		GO:0048511: rhythmic process
		GO:0016197: endosome transport
		GO:0004683: calmodulin-dependent protein kinase activity
		GO:0000151: ubiquitin ligase complex
		GO:0004721: phosphoprotein phosphatase activity
		GO:0055005: ventricular cardiac myofibril development
		GO:0007412: axon target recognition
		GO:0006289: nucleotide-excision repair
		GO:0008379: thioredoxin peroxidase activity
		GO:0004622: lysophospholipase activity
		GO:0006044: N-acetylglucosamine metabolic process
		GO:0048247: lymphocyte chemotaxis
		GO:0051045: negative regulation of membrane protein ectodomain proteolysis
		GO:0008354: germ cell migration
		GO:0004091: carboxylesterase activity
		GO:0042744: hydrogen peroxide catabolic process
		GO:0060048: cardiac muscle contraction
		GO:0019867: outer membrane
		GO:0009925: basal plasma membrane
#1051IR #1083IR	1	GO:0005499: vitamin D binding
#1063IR #1083IR	25	GO:0043259: laminin-10 complex
		GO:0042730: fibrinolysis
		GO:0045103: intermediate filament-based process
		GO:0005099: Ras GTPase activator activity
		GO:0005513: detection of calcium ion
		GO:0043257: laminin-8 complex
		GO:0030666: endocytic vesicle membrane
		GO:0030889: negative regulation of B cell proliferation
		GO:0050919: negative chemotaxis
		GO:0045666: positive regulation of neuron differentiation
		GO:0045499: chemorepellent activity
		GO:0021545: cranial nerve development
		GO:0007140: male meiosis
		GO:0043292: contractile fiber
		GO:0006968: cellular defense response
		GO:0035249: synaptic transmission, glutamatergic
		GO:0002027: regulation of heart rate
		GO:0007499: ectoderm and mesoderm interaction
		GO:0042986: positive regulation of amyloid precursor protein biosynthetic process
		GO:0005607: laminin-2 complex
		GO:0001843: neural tube closure
		GO:0007128: meiotic prophase I
		GO:0001736: establishment of planar polarity
		GO:0009314: response to radiation
		GO:0042094: interleukin-2 biosynthetic process
#1051IR #1063IR	10	GO:0016308: 1-phosphatidylinositol-4-phosphate 5-kinase activity
		GO:0048156: tau protein binding
		GO:0031018: endocrine pancreas development
		GO:0005898: interleukin-13 receptor complex
		GO:0032092: positive regulation of protein binding
		GO:0005596: collagen type XIV
		GO:0048814: regulation of dendrite morphogenesis
		GO:0014056: regulation of acetylcholine secretion
		GO:0045210: FasL biosynthetic process
		GO:0001822: kidney development
#10IR	552	GO:0008475: procollagen-lysine 5-dioxygenase activity
		GO:0032318: regulation of Ras GTPase activity
		GO:0006208: pyrimidine base catabolic process
		GO:0045995: regulation of embryonic development
		GO:0031849: olfactory receptor binding

GO:0001841: neural tube formation
GO:0032757: positive regulation of interleukin-8 production
GO:0005913: cell-cell adherens junction
GO:0016071: mRNA metabolic process
GO:0032714: negative regulation of interleukin-5 production
GO:0071565: nBAF complex
GO:0045909: positive regulation of vasodilation
GO:0032376: positive regulation of cholesterol transport
GO:0007502: digestive tract mesoderm development
GO:0045785: positive regulation of cell adhesion
GO:0034993: SUN-KASH complex
GO:0071285: cellular response to lithium ion
GO:0005220: inositol 1,4,5-trisphosphate-sensitive calcium-release channel activity
GO:0035590: purinergic nucleotide receptor signaling pathway
GO:0010976: positive regulation of neuron projection development
GO:0048625: myoblast cell fate commitment
GO:0031683: G-protein beta/gamma-subunit complex binding
GO:0070826: paraferritin complex
GO:0016051: carbohydrate biosynthetic process
GO:0051495: positive regulation of cytoskeleton organization
GO:0007250: activation of NF-kappaB-inducing kinase activity
GO:2000062: negative regulation of ureter smooth muscle cell differentiation
GO:0031313: extrinsic to endosome membrane
GO:0050434: positive regulation of viral transcription
GO:0015238: drug transmembrane transporter activity
GO:0060046: regulation of acrosome reaction
GO:0031095: platelet dense tubular network membrane
GO:0031090: organelle membrane
GO:0016540: protein autoprocessing
GO:0060662: salivary gland cavitation
GO:0001574: ganglioside biosynthetic process
GO:0060405: regulation of penile erection
GO:0043410: positive regulation of MAPKKK cascade
GO:0043132: NAD transport
GO:0021882: regulation of transcription from RNA polymerase II promoter involved in forebrain neuron fate commitment
GO:0019985: translesion synthesis
GO:0048844: artery morphogenesis
GO:0030151: molybdenum ion binding
GO:0048859: formation of anatomical boundary
GO:0031094: platelet dense tubular network
GO:0021940: positive regulation of cerebellar granule cell precursor proliferation
GO:0004461: lactose synthase activity
GO:0051209: release of sequestered calcium ion into cytosol
GO:0009101: glycoprotein biosynthetic process
GO:0060782: regulation of mesenchymal cell proliferation involved in prostate gland development
GO:0010288: response to lead ion
GO:0022602: ovulation cycle process
GO:0001916: positive regulation of T cell mediated cytotoxicity
GO:0014902: myotube differentiation
GO:0008269: JAK pathway signal transduction adaptor activity
GO:0043369: CD4-positive or CD8-positive, alpha-beta T cell lineage commitment
GO:0005989: lactose biosynthetic process
GO:0003214: cardiac left ventricle morphogenesis
GO:0071421: manganese ion transmembrane transport
GO:0070447: positive regulation of oligodendrocyte progenitor proliferation
GO:0030331: estrogen receptor binding
GO:0030854: positive regulation of granulocyte differentiation
GO:0015295: solute:hydrogen symporter activity
GO:0070682: proteasome regulatory particle assembly
GO:2000114: regulation of establishment of cell polarity
GO:0051901: positive regulation of mitochondrial depolarization
GO:0007340: acrosome reaction
GO:0034141: positive regulation of toll-like receptor 3 signaling pathway
GO:0034364: high-density lipoprotein particle
GO:0045741: positive regulation of epidermal growth factor receptor activity
GO:0046578: regulation of Ras protein signal transduction
GO:0030332: cyclin binding
GO:0021893: cerebral cortex GABAergic interneuron fate commitment
GO:0045727: positive regulation of translation
GO:0070574: cadmium ion transmembrane transport
GO:0046686: response to cadmium ion
GO:0031572: G2/M transition DNA damage checkpoint
GO:0042733: embryonic digit morphogenesis
GO:0005814: centriole
GO:0004087: carbamoyl-phosphate synthase (ammonia) activity
GO:0032000: positive regulation of fatty acid beta-oxidation
GO:0032229: negative regulation of synaptic transmission, GABAergic
GO:0051001: negative regulation of nitric-oxide synthase activity
GO:0042489: negative regulation of odontogenesis of dentine-containing tooth
GO:0033017: sarcoplasmic reticulum membrane
GO:0031214: biomineral tissue development
GO:0006801: superoxide metabolic process
GO:0010042: response to manganese ion
GO:0016925: protein sumoylation
GO:0032648: regulation of interferon-beta production
GO:0051938: L-glutamate import
GO:0009331: glycerol-3-phosphate dehydrogenase complex
GO:0070053: thrombospondin receptor activity
GO:0006196: AMP catabolic process
GO:0071223: cellular response to lipoteichoic acid
GO:0055072: iron ion homeostasis
GO:0003727: single-stranded RNA binding
GO:0031999: negative regulation of fatty acid beta-oxidation
GO:0035253: ciliary rootlet
GO:0051496: positive regulation of stress fiber assembly
GO:0071864: positive regulation of cell proliferation in bone marrow
GO:0051894: positive regulation of focal adhesion assembly
GO:0005639: integral to nuclear inner membrane
GO:0045759: negative regulation of action potential
GO:0050715: positive regulation of cytokine secretion
GO:0071866: negative regulation of apoptosis in bone marrow
GO:0006349: regulation of gene expression by genetic imprinting
GO:0045136: development of secondary sexual characteristics
GO:0040038: polar body extrusion after meiotic divisions
GO:0005313: L-glutamate transmembrane transporter activity
GO:0030388: fructose 1,6-bisphosphate metabolic process
GO:0015085: calcium ion transmembrane transporter activity
GO:0050829: defense response to Gram-negative bacterium
GO:2000343: positive regulation of chemokine (C-X-C motif) ligand 2 production
GO:0070409: carbamoyl phosphate biosynthetic process
GO:0005113: patched binding
GO:0005351: sugar:hydrogen symporter activity
GO:0018119: peptidyl-cysteine S-nitrosylation
GO:0034618: arginine binding
GO:0042153: RPTP-like protein binding
GO:0030155: regulation of cell adhesion
GO:0004368: glycerol-3-phosphate dehydrogenase activity
GO:0005795: Golgi stack
GO:0008253: 5'-nucleotidase activity
GO:0005094: Rho GDP-dissociation inhibitor activity
GO:0016528: sarcoplasm
GO:0015676: vanadium ion transport
GO:0042098: T cell proliferation
GO:0015631: tubulin binding
GO:0004499: flavin-containing monooxygenase activity
GO:0060439: trachea morphogenesis
GO:0015100: vanadium ion transmembrane transporter activity

GO:0015299: solute:hydrogen antiporter activity
GO:0007228: positive regulation of hh target transcription factor activity
GO:0043923: positive regulation by host of viral transcription
GO:0035434: copper ion transmembrane transport
GO:0031953: negative regulation of protein autophosphorylation
GO:0046685: response to arsenic-containing substance
GO:0048715: negative regulation of oligodendrocyte differentiation
GO:0004835: tubulin-tyrosine ligase activity
GO:0006004: fucose metabolic process
GO:0006956: complement activation
GO:0071548: response to dexamethasone stimulus
GO:0016151: nickel ion binding
GO:0045880: positive regulation of smoothened signaling pathway
GO:0090022: regulation of neutrophil chemotaxis
GO:0006651: diacylglycerol biosynthetic process
GO:0005104: fibroblast growth factor receptor binding
GO:0031981: nuclear lumen
GO:0070679: inositol 1,4,5 trisphosphate binding
GO:0046168: glycerol-3-phosphate catabolic process
GO:0042178: xenobiotic catabolic process
GO:0001660: fever generation
GO:0007494: midgut development
GO:0014043: negative regulation of neuron maturation
GO:0035444: nickel ion transmembrane transport
GO:0030131: clathrin adaptor complex
GO:0050482: arachidonic acid secretion
GO:0032993: protein-DNA complex
GO:0061056: sclerotome development
GO:0048592: eye morphogenesis
GO:0090068: positive regulation of cell cycle process
GO:0060783: mesenchymal smoothened signaling pathway involved in prostate gland development
GO:2000357: negative regulation of kidney smooth muscle cell differentiation
GO:0002793: positive regulation of peptide secretion
GO:0035663: Toll-like receptor 2 binding
GO:0007224: smoothened signaling pathway
GO:0007132: meiotic metaphase I
GO:0060406: positive regulation of penile erection
GO:0015884: folic acid transport
GO:0006972: hyperosmotic response
GO:0045178: basal part of cell
GO:0050717: positive regulation of interleukin-1 alpha secretion
GO:0006649: phospholipid transfer to membrane
GO:0046888: negative regulation of hormone secretion
GO:0006868: glutamine transport
GO:0004607: phosphatidylcholine-sterol O-acyltransferase activity
GO:0060259: regulation of feeding behavior
GO:0034501: protein localization to kinetochore
GO:0032060: bleb assembly
GO:0032348: negative regulation of aldosterone biosynthetic process
GO:0043032: positive regulation of macrophage activation
GO:0032224: positive regulation of synaptic transmission, cholinergic
GO:0035326: enhancer binding
GO:0060025: regulation of synaptic activity
GO:0001658: branching involved in ureteric bud morphogenesis
GO:0060428: lung epithelium development
GO:0051155: positive regulation of striated muscle cell differentiation
GO:0016628: oxidoreductase activity, acting on the CH-CH group of donors, NAD or NADP as acceptor
GO:0004560: alpha-L-fucosidase activity
GO:0009411: response to UV
GO:0005610: laminin-5 complex
GO:0043236: laminin binding
GO:0010039: response to iron ion
GO:0060458: right lung development
GO:0060135: maternal process involved in female pregnancy
GO:0007097: nuclear migration
GO:0008417: fucosyltransferase activity
GO:0006930: substrate-dependent cell migration, cell extension
GO:0033004: negative regulation of mast cell activation
GO:0048738: cardiac muscle tissue development
GO:0048714: positive regulation of oligodendrocyte differentiation
GO:0022408: negative regulation of cell-cell adhesion
GO:0046639: negative regulation of alpha-beta T cell differentiation
GO:0072568: protein kinase C delta binding
GO:0035091: phosphatidylinositol binding
GO:0032738: positive regulation of interleukin-15 production
GO:0030049: muscle filament sliding
GO:0001759: organ induction
GO:0003990: acetylcholinesterase activity
GO:0003032: detection of oxygen
GO:0003093: regulation of glomerular filtration
GO:0016344: meiotic chromosome movement towards spindle pole
GO:0015116: sulfate transmembrane transporter activity
GO:0050031: L-pipecolate oxidase activity
GO:0050995: negative regulation of lipid catabolic process
GO:0045652: regulation of megakaryocyte differentiation
GO:0003407: neural retina development
GO:0008272: sulfate transport
GO:0016539: intein-mediated protein splicing
GO:0007009: plasma membrane organization
GO:0030030: cell projection organization
GO:0031668: cellular response to extracellular stimulus
GO:0006828: manganese ion transport
GO:0042323: negative regulation of circadian sleep/wake cycle, non-REM sleep
GO:0004950: chemokine receptor activity
GO:0006884: cell volume homeostasis
GO:0030284: estrogen receptor activity
GO:0033198: response to ATP
GO:0045059: positive thymic T cell selection
GO:0015093: ferrous iron transmembrane transporter activity
GO:0045822: negative regulation of heart contraction
GO:0016514: SWI/SNF complex
GO:0051712: positive regulation of killing of cells of other organism
GO:0045777: positive regulation of blood pressure
GO:0033077: T cell differentiation in thymus
GO:0044429: mitochondrial part
GO:0051439: regulation of ubiquitin-protein ligase activity involved in mitotic cell cycle
GO:0014732: skeletal muscle atrophy
GO:0005640: nuclear outer membrane
GO:0005588: collagen type V
GO:0004367: glycerol-3-phosphate dehydrogenase [NAD+] activity
GO:0031022: nuclear migration along microfilament
GO:0045776: negative regulation of blood pressure
GO:0009953: dorsal/ventral pattern formation
GO:0042474: middle ear morphogenesis
GO:0030126: COPI vesicle coat
GO:0005384: manganese ion transmembrane transporter activity
GO:0048617: embryonic foregut morphogenesis
GO:0008209: androgen metabolic process
GO:0001955: blood vessel maturation
GO:0000185: activation of MAPKKK activity
GO:0046534: positive regulation of photoreceptor cell differentiation
GO:0034123: positive regulation of toll-like receptor signaling pathway
GO:0006839: mitochondrial transport
GO:0048771: tissue remodeling
GO:0004332: fructose-bisphosphate aldolase activity
GO:0033265: choline binding
GO:0050718: positive regulation of interleukin-1 beta secretion

GO:0001614: purinergic nucleotide receptor activity
GO:0060738: epithelial-mesenchymal signaling involved in prostate gland development
GO:0008353: RNA polymerase II carboxy-terminal domain kinase activity
GO:0045779: negative regulation of bone resorption
GO:0006900: membrane budding
GO:0007217: tachykinin receptor signaling pathway
GO:0004931: extracellular ATP-gated cation channel activity
GO:0032308: positive regulation of prostaglandin secretion
GO:0006259: DNA metabolic process
GO:0070097: delta-catenin binding
GO:0090073: positive regulation of protein homodimerization activity
GO:0001708: cell fate specification
GO:0008504: monoamine transmembrane transporter activity
GO:0060586: multicellular organismal iron ion homeostasis
GO:0051967: negative regulation of synaptic transmission, glutamatergic
GO:0005148: prolactin receptor binding
GO:0047496: vesicle transport along microtubule
GO:0032728: positive regulation of interferon-beta production
GO:0048645: organ formation
GO:0045359: positive regulation of interferon-beta biosynthetic process
GO:0046486: glycerolipid metabolic process
GO:0030658: transport vesicle membrane
GO:0015385: sodium:hydrogen antiporter activity
GO:0004301: epoxide hydrolase activity
GO:0051287: NAD binding
GO:0021978: telencephalon regionalization
GO:0010467: gene expression
GO:0001845: phagolysosome assembly
GO:0042301: phosphate binding
GO:0007520: myoblast fusion
GO:0045410: positive regulation of interleukin-6 biosynthetic process
GO:0030035: microspike assembly
GO:0021772: olfactory bulb development
GO:0048016: inositol phosphate-mediated signaling
GO:0032228: regulation of synaptic transmission, GABAergic
GO:0060020: Bergmann glial cell differentiation
GO:0060054: positive regulation of epithelial cell proliferation involved in wound healing
GO:0021521: ventral spinal cord interneuron specification
GO:0034339: regulation of transcription from RNA polymerase II promoter by nuclear hormone receptor
GO:0044325: ion channel binding
GO:0090197: positive regulation of chemokine secretion
GO:0055038: recycling endosome membrane
GO:0005367: myo-inositol:sodium symporter activity
GO:0045356: positive regulation of interferon-alpha biosynthetic process
GO:0071221: cellular response to bacterial lipopeptide
GO:0008970: phospholipase A1 activity
GO:0050897: cobalt ion binding
GO:0007032: endosome organization
GO:0035662: Toll-like receptor 4 binding
GO:0071353: cellular response to interleukin-4
GO:0034145: positive regulation of toll-like receptor 4 signaling pathway
GO:0007339: binding of sperm to zona pellucida
GO:0045908: negative regulation of vasodilation
GO:0051437: positive regulation of ubiquitin-protein ligase activity involved in mitotic cell cycle
GO:0072136: metanephric mesenchymal cell proliferation involved in metanephros development
GO:0019532: oxalate transport
GO:0045294: alpha-catenin binding
GO:0009629: response to gravity
GO:0051709: regulation of killing of cells of other organism
GO:0006937: regulation of muscle contraction
GO:0005290: L-histidine transmembrane transporter activity
GO:0010983: positive regulation of high-density lipoprotein particle clearance
GO:0009922: fatty acid elongase activity
GO:0033602: negative regulation of dopamine secretion
GO:0002866: positive regulation of acute inflammatory response to antigenic stimulus
GO:0005605: basal lamina
GO:0090041: negative regulation of gene-specific transcription elongation from RNA polymerase II promoter
GO:0030223: neutrophil differentiation
GO:0051295: establishment of meiotic spindle localization
GO:0050661: NADP binding
GO:0034617: tetrahydrobiopterin binding
GO:0015099: nickel ion transmembrane transporter activity
GO:0007418: ventral midline development
GO:0010977: negative regulation of neuron projection development
GO:0000050: urea cycle
GO:0017034: Rap guanyl-nucleotide exchange factor activity
GO:0022890: inorganic cation transmembrane transporter activity
GO:0031526: brush border membrane
GO:0019531: oxalate transmembrane transporter activity
GO:0019290: siderophore biosynthetic process
GO:0035250: UDP-galactosyltransferase activity
GO:0048484: enteric nervous system development
GO:0002028: regulation of sodium ion transport
GO:0001530: lipopolysaccharide binding
GO:0032696: negative regulation of interleukin-13 production
GO:0016578: histone deubiquitination
GO:0001609: adenosine receptor activity, G-protein coupled
GO:0046086: adenosine biosynthetic process
GO:0051436: negative regulation of ubiquitin-protein ligase activity involved in mitotic cell cycle
GO:0090286: cytoskeletal anchoring at nuclear membrane
GO:0051270: regulation of cellular component movement
GO:0034230: enkephalin processing
GO:2000065: negative regulation of cortisol biosynthetic process
GO:0046931: pore complex assembly
GO:0042761: very long-chain fatty acid biosynthetic process
GO:0032900: negative regulation of neurotrophin production
GO:0046653: tetrahydrofolate metabolic process
GO:0016986: transcription initiation factor activity
GO:0030183: B cell differentiation
GO:0004517: nitric-oxide synthase activity
GO:0006195: purine nucleotide catabolic process
GO:0003725: double-stranded RNA binding
GO:0006829: zinc ion transport
GO:0014741: negative regulation of muscle hypertrophy
GO:0045060: negative thymic T cell selection
GO:0048589: developmental growth
GO:0035665: TIRAP-dependent toll-like receptor 4 signaling pathway
GO:0014050: negative regulation of glutamate secretion
GO:0019240: citrulline biosynthetic process
GO:0071400: cellular response to oleic acid
GO:0030502: negative regulation of bone mineralization
GO:0002076: osteoblast development
GO:0010043: response to zinc ion
GO:0008095: inositol-1,4,5-trisphosphate receptor activity
GO:0033276: transcription factor TFTC complex
GO:0015817: histidine transport
GO:0072199: regulation of mesenchymal cell proliferation involved in ureter development
GO:0015182: L-asparagine transmembrane transporter activity
GO:0002087: regulation of respiratory gaseous exchange by neurological system process
GO:0004513: neolactotetraosylceramide alpha-2,3-sialyltransferase activity
GO:0031821: metabotropic serotonin receptor binding
GO:0005218: intracellular ligand-gated calcium channel activity
GO:0014054: positive regulation of gamma-aminobutyric acid secretion
GO:0035338: long-chain fatty-acyl-CoA biosynthetic process
GO:0032835: glomerulus development
GO:0034405: response to fluid shear stress
GO:0021513: spinal cord dorsal/ventral patterning

GO:0030856: regulation of epithelial cell differentiation
GO:0043271: negative regulation of ion transport
GO:0045078: positive regulation of interferon-gamma biosynthetic process
GO:0051290: protein heterotetramerization
GO:0071899: negative regulation of estrogen receptor binding
GO:0005131: growth hormone receptor binding
GO:0071577: zinc ion transmembrane transport
GO:2000063: positive regulation of ureter smooth muscle cell differentiation
GO:2000358: positive regulation of kidney smooth muscle cell differentiation
GO:0031527: filopodium membrane
GO:0002227: innate immune response in mucosa
GO:0006116: NADH oxidation
GO:0048864: stem cell development
GO:0050543: icosatetraenoic acid binding
GO:0031685: adenosine receptor binding
GO:0002320: lymphoid progenitor cell differentiation
GO:0015094: lead ion transmembrane transporter activity
GO:0060326: cell chemotaxis
GO:0070627: ferrous iron import
GO:0030574: collagen catabolic process
GO:0004864: protein phosphatase inhibitor activity
GO:0031117: positive regulation of microtubule depolymerization
GO:0046923: ER retention sequence binding
GO:0006513: protein monoubiquitination
GO:0031088: platelet dense granule membrane
GO:0045063: T-helper 1 cell differentiation
GO:0006977: DNA damage response, signal transduction by p53 class mediator resulting in cell cycle arrest
GO:0030010: establishment of cell polarity
GO:0071436: sodium ion export
GO:0003858: 3-hydroxybutyrate dehydrogenase activity
GO:0042589: zymogen granule membrane
GO:0044212: transcription regulatory region DNA binding
GO:0043967: histone H4 acetylation
GO:0034597: phosphatidylinositol-4,5-bisphosphate 4-phosphatase activity
GO:0016358: dendrite development
GO:0034605: cellular response to heat
GO:0051642: centrosome localization
GO:0004095: carnitine O-palmitoyltransferase activity
GO:0006612: protein targeting to membrane
GO:0042307: positive regulation of protein import into nucleus
GO:0060170: cilium membrane
GO:0070542: response to fatty acid
GO:0032059: bleb
GO:0032403: protein complex binding
GO:0015844: monoamine transport
GO:0042100: B cell proliferation
GO:0050805: negative regulation of synaptic transmission
GO:0000293: ferric-chelate reductase activity
GO:0016791: phosphatase activity
GO:0060058: positive regulation of apoptosis involved in mammary gland involution
GO:0060685: regulation of prostatic bud formation
GO:0048643: positive regulation of skeletal muscle tissue development
GO:0032435: negative regulation of proteasomal ubiquitin-dependent protein catabolic process
GO:0030099: myeloid cell differentiation
GO:0031581: hemidesmosome assembly
GO:0016485: protein processing
GO:0003831: beta-N-acetylglucosaminylglycopeptide beta-1,4-galactosyltransferase activity
GO:0008020: G-protein coupled photoreceptor activity
GO:0055118: negative regulation of cardiac muscle contraction
GO:0006406: mRNA export from nucleus
GO:0008540: proteasome regulatory particle, base subcomplex
GO:0002064: epithelial cell development
GO:0046513: ceramide biosynthetic process
GO:0002162: dystroglycan binding
GO:0034231: islet amyloid polypeptide processing
GO:0006621: protein retention in ER lumen
GO:0015106: bicarbonate transmembrane transporter activity
GO:0009584: detection of visible light
GO:0047291: lactosylceramide alpha-2,3-sialyltransferase activity
GO:0016998: cell wall macromolecule catabolic process
GO:0034626: fatty acid elongation, polyunsaturated fatty acid
GO:0060447: bud outgrowth involved in lung branching
GO:0060840: artery development
GO:0060516: primary prostatic bud elongation
GO:0042346: positive regulation of NF-kappaB import into nucleus
GO:0046632: alpha-beta T cell differentiation
GO:0001912: positive regulation of leukocyte mediated cytotoxicity
GO:0046332: SMAD binding
GO:0043615: astrocyte cell migration
GO:0031100: organ regeneration
GO:0033092: positive regulation of immature T cell proliferation in thymus
GO:0042168: heme metabolic process
GO:0001578: microtubule bundle formation
GO:0000138: Golgi trans cisterna
GO:2000340: positive regulation of chemokine (C-X-C motif) ligand 1 production
GO:0014858: positive regulation of skeletal muscle cell proliferation
GO:0030112: glycocalyx
GO:0051895: negative regulation of focal adhesion assembly
GO:0044344: cellular response to fibroblast growth factor stimulus
GO:0048646: anatomical structure formation involved in morphogenesis
GO:0007026: negative regulation of microtubule depolymerization
GO:0007442: hindgut morphogenesis
GO:0006470: protein dephosphorylation
GO:0043401: steroid hormone mediated signaling pathway
GO:0048632: negative regulation of skeletal muscle tissue growth
GO:0016615: malate dehydrogenase activity
GO:0045445: myoblast differentiation
GO:0001948: glycoprotein binding
GO:0034137: positive regulation of toll-like receptor 2 signaling pathway
GO:0015684: ferrous iron transport
GO:0006867: asparagine transport
GO:0048821: erythrocyte development
GO:0030070: insulin processing
GO:0014706: striated muscle tissue development
GO:0015301: anion:anion antiporter activity
GO:0043268: positive regulation of potassium ion transport
GO:0060459: left lung development
GO:0050746: regulation of lipoprotein metabolic process
GO:0021938: smoothened signaling pathway involved in regulation of cerebellar granule cell precursor cell proliferation
GO:0015086: cadmium ion transmembrane transporter activity
GO:0004086: carbamoyl-phosphate synthase activity
GO:0043279: response to alkaloid
GO:0009597: detection of virus
GO:0042308: negative regulation of protein import into nucleus
GO:0007257: activation of JUN kinase activity
GO:0006000: fructose metabolic process
GO:0031464: Cul4A-RING ubiquitin ligase complex
GO:0004629: phospholipase C activity
GO:0010740: positive regulation of intracellular protein kinase cascade
GO:0019834: phospholipase A2 inhibitor activity
GO:0015081: sodium ion transmembrane transporter activity
GO:0030878: thyroid gland development
GO:0021904: dorsal/ventral neural tube patterning
GO:0060769: positive regulation of epithelial cell proliferation involved in prostate gland development
GO:0031465: Cul4B-RING ubiquitin ligase complex
GO:0019395: fatty acid oxidation
GO:0034435: cholesterol esterification

GO:0019367: fatty acid elongation, saturated fatty acid
GO:0043392: negative regulation of DNA binding
GO:0043587: tongue morphogenesis
GO:0008486: diphosphoinositol-polyphosphate diphosphatase activity
GO:0015087: cobalt ion transmembrane transporter activity
GO:0017144: drug metabolic process
GO:0032244: positive regulation of nucleoside transport
GO:0032795: heterotrimeric G-protein binding
GO:0004402: histone acetyltransferase activity
GO:0043029: T cell homeostasis
GO:0003920: GMP reductase activity
GO:0030914: STAGA complex
GO:0015675: nickel ion transport
GO:0006824: cobalt ion transport
GO:0005932: microtubule basal body
GO:0005385: zinc ion transmembrane transporter activity
GO:0008328: ionotropic glutamate receptor complex
GO:0006606: protein import into nucleus
GO:0032735: positive regulation of interleukin-12 production
GO:0032713: negative regulation of interleukin-4 production
GO:0005272: sodium channel activity
GO:0002052: positive regulation of neuroblast proliferation
GO:0080125: multicellular structure septum development
GO:0044254: multicellular organismal protein catabolic process
GO:0032591: dendritic spine membrane
GO:0048155: S100 alpha binding
GO:0016705: oxidoreductase activity, acting on paired donors, with incorporation or reduction of molecular oxygen
GO:0030742: GTP-dependent protein binding
GO:0008115: sarcosine oxidase activity
GO:0015692: lead ion transport
GO:0030217: T cell differentiation
GO:0070779: D-aspartate import
GO:0005542: folic acid binding
GO:0050965: detection of temperature stimulus involved in sensory perception of pain
GO:0070016: armadillo repeat domain binding
GO:0055081: anion homeostasis
GO:0042158: lipoprotein biosynthetic process
GO:0048314: embryo sac morphogenesis
GO:0005324: long-chain fatty acid transporter activity
#1095IR 716 GO:0048676: axon extension involved in development
GO:0032052: bile acid binding
GO:0009790: embryonic development
GO:0006807: nitrogen compound metabolic process
GO:0007519: skeletal muscle development
GO:0007212: dopamine receptor signaling pathway
GO:0009434: microtubule-based flagellum
GO:0010766: negative regulation of sodium ion transport
GO:0005761: mitochondrial ribosome
GO:0050665: hydrogen peroxide biosynthetic process
GO:0004828: serine-tRNA ligase activity
GO:0004017: adenylate kinase activity
GO:0032781: positive regulation of ATPase activity
GO:0004813: alanine-tRNA ligase activity
GO:0043754: dihydrolipoyllysine-residue (2-methylpropanoyl)transferase activity
GO:0031105: septin complex
GO:0006413: translational initiation
GO:0019911: structural constituent of myelin sheath
GO:0046933: hydrogen ion transporting ATP synthase activity, rotational mechanism
GO:0004579: dolichyl-diphosphooligosaccharide-protein glycotransferase activity
GO:0042384: cilium assembly
GO:0014068: positive regulation of phosphoinositide 3-kinase cascade
GO:0045747: positive regulation of Notch signaling pathway
GO:0019835: cytolysis
GO:0043507: positive regulation of JUN kinase activity
GO:0005885: Arp2/3 protein complex
GO:0051603: proteolysis involved in cellular protein catabolic process
GO:0009755: hormone-mediated signaling
GO:0006857: oligopeptide transport
GO:0051538: 3 iron, 4 sulfur cluster binding
GO:0004825: methionine-tRNA ligase activity
GO:0033179: proton-transporting V-type ATPase, V0 domain
GO:0004347: glucose-6-phosphate isomerase activity
GO:0043498: cell surface binding
GO:0009086: methionine biosynthetic process
GO:0003983: UTP:glucose-1-phosphate uridylyltransferase activity
GO:0006694: steroid biosynthetic process
GO:0008494: translation activator activity
GO:0018193: peptidyl-amino acid modification
GO:0047042: 3-alpha-hydroxysteroid dehydrogenase (B-specific) activity
GO:0031571: G1 DNA damage checkpoint
GO:0003954: NADH dehydrogenase activity
GO:0042511: positive regulation of tyrosine phosphorylation of Stat1 protein
GO:0015520: tetracycline:hydrogen antiporter activity
GO:0051346: negative regulation of hydrolase activity
GO:0004800: thyroxine 5'-deiodinase activity
GO:0003980: UDP-glucose:glycoprotein glucosyltransferase activity
GO:0006590: thyroid hormone generation
GO:0045636: positive regulation of melanocyte differentiation
GO:0001601: peptide YY receptor activity
GO:0031669: cellular response to nutrient levels
GO:0051016: barbed-end actin filament capping
GO:0048269: methionine adenosyltransferase complex
GO:0005151: interleukin-1, Type II receptor binding
GO:0035091: phosphoinositide binding
GO:0004337: geranyltranstransferase activity
GO:0000224: peptide-N4-(N-acetyl-beta-glucosaminyl)asparagine amidase activity
GO:0004984: olfactory receptor activity
GO:0046902: regulation of mitochondrial membrane permeability
GO:0005762: mitochondrial large ribosomal subunit
GO:0016229: steroid dehydrogenase activity
GO:0050815: phosphoserine binding
GO:0018879: biphenyl metabolic process
GO:0007369: gastrulation
GO:0019902: phosphatase binding
GO:0045298: tubulin complex
GO:0005000: vasopressin receptor activity
GO:0017183: peptidyl-diphthamide biosynthetic process from peptidyl-histidine
GO:0005839: proteasome core complex
GO:0005763: mitochondrial small ribosomal subunit
GO:0005859: muscle myosin complex
GO:0006563: L-serine metabolic process
GO:0015457: auxiliary transport protein activity
GO:0015272: ATP-activated inward rectifier potassium channel activity
GO:0005643: nuclear pore
GO:0046934: phosphatidylinositol-4,5-bisphosphate 3-kinase activity
GO:0001945: lymph vessel development
GO:0021954: central nervous system neuron development
GO:0046961: proton-transporting ATPase activity, rotational mechanism
GO:0032287: myelin maintenance in the peripheral nervous system
GO:0008061: chitin binding
GO:0008449: N-acetylglucosamine-6-sulfatase activity
GO:0045815: positive regulation of gene expression, epigenetic
GO:0052171: growth or development during symbiotic interaction
GO:0015450: P-P-bond-hydrolysis-driven protein transmembrane transporter activity
GO:0016786: selenotransferase activity
GO:0019008: molybdopterin synthase complex
GO:0006368: RNA elongation from RNA polymerase II promoter

GO:0045475: locomotor rhythm
GO:0004697: protein kinase C activity
GO:0006519: cellular amino acid and derivative metabolic process
GO:0045840: positive regulation of mitosis
GO:0001895: retina homeostasis
GO:0005942: phosphoinositide 3-kinase complex
GO:0005744: mitochondrial inner membrane presequence translocase complex
GO:0004905: type I interferon receptor activity
GO:0006431: methionyl-tRNA aminoacylation
GO:0051146: striated muscle cell differentiation
GO:0002318: myeloid progenitor cell differentiation
GO:0006027: glycosaminoglycan catabolic process
GO:0048861: leukemia inhibitory factor signaling pathway
GO:0004035: alkaline phosphatase activity
GO:0035254: glutamate receptor binding
GO:0004743: pyruvate kinase activity
GO:0008479: queuine tRNA-ribosyltransferase activity
GO:0004174: electron-transferring-flavoprotein dehydrogenase activity
GO:0015904: tetracycline transport
GO:0032729: positive regulation of interferon-gamma production
GO:0018345: protein palmitoylation
GO:0016846: carbon-sulfur lyase activity
GO:0003729: mRNA binding
GO:0043209: myelin sheath
GO:0005851: eukaryotic translation initiation factor 2B complex
GO:0050661: NADP or NADPH binding
GO:0000275: mitochondrial proton-transporting ATP synthase complex, catalytic core F(1)
GO:0000119: mediator complex
GO:0005746: mitochondrial respiratory chain
GO:0004656: procollagen-proline 4-dioxygenase activity
GO:0030515: snoRNA binding
GO:0042423: catecholamine biosynthetic process
GO:0001817: regulation of cytokine production
GO:0032038: myosin II heavy chain binding
GO:0004771: sterol esterase activity
GO:0015248: sterol transporter activity
GO:0004569: glycoprotein endo-alpha-1,2-mannosidase activity
GO:0008616: queuosine biosynthetic process
GO:0006817: phosphate transport
GO:0005139: interleukin-7 receptor binding
GO:0051450: myoblast proliferation
GO:0000247: C-8 sterol isomerase activity
GO:0006122: mitochondrial electron transport, ubiquinol to cytochrome c
GO:0030060: L-malate dehydrogenase activity
GO:0033119: negative regulation of RNA splicing
GO:0009303: rRNA transcription
GO:0045648: positive regulation of erythrocyte differentiation
GO:0008177: succinate dehydrogenase (ubiquinone) activity
GO:0004134: 4-alpha-glucanotransferase activity
GO:0045899: positive regulation of transcriptional preinitiation complex assembly
GO:0019206: nucleoside kinase activity
GO:0000254: C-4 methylsterol oxidase activity
GO:0004504: peptidylglycine monooxygenase activity
GO:0051287: NAD or NADH binding
GO:0050840: extracellular matrix binding
GO:0042491: auditory receptor cell differentiation
GO:0008353: RNA polymerase subunit kinase activity
GO:0005173: stem cell factor receptor binding
GO:0006904: vesicle docking during exocytosis
GO:0004587: ornithine-oxo-acid transaminase activity
GO:0034464: BBSome
GO:0044237: cellular metabolic process
GO:0004897: ciliary neurotrophic factor receptor activity
GO:0032801: receptor catabolic process
GO:0006298: mismatch repair
GO:0042273: ribosomal large subunit biogenesis
GO:0006544: glycine metabolic process
GO:0045446: endothelial cell differentiation
GO:0008190: eukaryotic initiation factor 4E binding
GO:0004165: dodecenoyl-CoA delta-isomerase activity
GO:0003906: DNA-(apurinic or apyrimidinic site) lyase activity
GO:0004008: copper-exporting ATPase activity
GO:0045261: proton-transporting ATP synthase complex, catalytic core F(1)
GO:0002262: myeloid cell homeostasis
GO:0004487: methylenetetrahydrofolate dehydrogenase (NAD+) activity
GO:0045794: negative regulation of cell volume
GO:0018279: protein amino acid N-linked glycosylation via asparagine
GO:0006338: chromatin remodeling
GO:0060163: subpallium neuron fate commitment
GO:0046677: response to antibiotic
GO:0043353: enucleate erythrocyte differentiation
GO:0003684: damaged DNA binding
GO:0051208: sequestering of calcium ion
GO:0051087: chaperone binding
GO:0003887: DNA-directed DNA polymerase activity
GO:0043218: compact myelin
GO:0043033: isoamylase complex
GO:0045947: negative regulation of translational initiation
GO:0000387: spliceosomal snRNP biogenesis
GO:0047021: 15-hydroxyprostaglandin dehydrogenase (NADP+) activity
GO:0005126: hematopoietin/interferon-class (D200-domain) cytokine receptor binding
GO:0008299: isoprenoid biosynthetic process
GO:0045239: tricarboxylic acid cycle enzyme complex
GO:0020027: hemoglobin metabolic process
GO:0030534: adult behavior
GO:0002687: positive regulation of leukocyte migration
GO:0042921: glucocorticoid receptor signaling pathway
GO:0016251: general RNA polymerase II transcription factor activity
GO:0008137: NADH dehydrogenase (ubiquinone) activity
GO:0000380: alternative nuclear mRNA splicing, via spliceosome
GO:0065002: intracellular protein transmembrane transport
GO:0003706: ligand-regulated transcription factor activity
GO:0019789: SUMO ligase activity
GO:0019682: glyceraldehyde-3-phosphate metabolic process
GO:0004521: endoribonuclease activity
GO:0046854: phosphoinositide phosphorylation
GO:0004921: interleukin-11 receptor activity
GO:0016531: copper chaperone activity
GO:0031403: lithium ion binding
GO:0006924: activation-induced cell death of T cells
GO:0047750: cholestenol delta-isomerase activity
GO:0019209: kinase activator activity
GO:0022625: cytosolic large ribosomal subunit
GO:0032364: oxygen homeostasis
GO:0046326: positive regulation of glucose import
GO:0060052: neurofilament cytoskeleton organization
GO:0043249: erythrocyte maturation
GO:0004135: amylo-alpha-1,6-glucosidase activity
GO:0008599: protein phosphatase type 1 regulator activity
GO:0042552: myelination
GO:0001932: regulation of protein amino acid phosphorylation
GO:0016878: acid-thiol ligase activity
GO:0055010: ventricular cardiac muscle morphogenesis
GO:0002821: positive regulation of adaptive immune response
GO:0060072: large conductance calcium-activated potassium channel activity
GO:0005669: transcription factor TFIID complex
GO:0001892: embryonic placenta development

GO:0015721: bile acid and bile salt transport
GO:0005784: translocon complex
GO:0030641: regulation of cellular pH
GO:0042759: long-chain fatty acid biosynthetic process
GO:0001952: regulation of cell-matrix adhesion
GO:0051920: peroxiredoxin activity
GO:0006800: oxygen and reactive oxygen species metabolic process
GO:0050221: prostaglandin-E2 9-reductase activity
GO:0004526: ribonuclease P activity
GO:0042769: DNA damage response, detection of DNA damage
GO:0043039: tRNA aminoacylation
GO:0030169: low-density lipoprotein binding
GO:0007213: muscarinic acetylcholine receptor signaling pathway
GO:0030371: translation repressor activity
GO:0042541: hemoglobin biosynthetic process
GO:0016081: synaptic vesicle docking during exocytosis
GO:0004300: enoyl-CoA hydratase activity
GO:0051318: G1 phase
GO:0004634: phosphopyruvate hydratase activity
GO:0004576: oligosaccharyl transferase activity
GO:0004161: dimethylallyltranstransferase activity
GO:0006301: postreplication repair
GO:0005977: glycogen metabolic process
GO:0003923: GPI-anchor transamidase activity
GO:0042719: mitochondrial intermembrane space protein transporter complex
GO:0005372: water transporter activity
GO:0005853: eukaryotic translation elongation factor 1 complex
GO:0006654: phosphatidic acid biosynthetic process
GO:0033081: regulation of T cell differentiation in the thymus
GO:0004064: arylesterase activity
GO:0042835: BRE binding
GO:0000827: inositol 1,3,4,5,6-pentakisphosphate kinase activity
GO:0008324: cation transmembrane transporter activity
GO:0006221: pyrimidine nucleotide biosynthetic process
GO:0060088: auditory receptor cell stereocilium organization
GO:0008333: endosome to lysosome transport
GO:0006082: organic acid metabolic process
GO:0007309: oocyte axis specification
GO:0004769: steroid delta-isomerase activity
GO:0006984: ER-nuclear signaling pathway
GO:0031401: positive regulation of protein modification process
GO:0006309: DNA fragmentation during apoptosis
GO:0004924: oncostatin-M receptor activity
GO:0004740: pyruvate dehydrogenase (acetyl-transferring) kinase activity
GO:0043162: ubiquitin-dependent protein catabolic process via the multivesicular body sorting pathway
GO:0004058: aromatic-L-amino-acid decarboxylase activity
GO:0000381: regulation of alternative nuclear mRNA splicing, via spliceosome
GO:0000015: phosphopyruvate hydratase complex
GO:0003988: acetyl-CoA C-acyltransferase activity
GO:0016254: preassembly of GPI anchor in ER membrane
GO:0000389: nuclear mRNA 3'-splice site recognition
GO:0032259: methylation
GO:0004159: dihydrouracil dehydrogenase (NAD+) activity
GO:0045738: negative regulation of DNA repair
GO:0051262: protein tetramerization
GO:0000210: NAD+ diphosphatase activity
GO:0000781: chromosome, telomeric region
GO:0006518: peptide metabolic process
GO:0004137: deoxycytidine kinase activity
GO:0006011: UDP-glucose metabolic process
GO:0033814: propanoyl-CoA C-acyltransferase activity
GO:0005072: transforming growth factor beta receptor, cytoplasmic mediator activity
GO:0006789: bilirubin conjugation
GO:0006529: asparagine biosynthetic process
GO:0031508: centromeric heterochromatin formation
GO:0031119: tRNA pseudouridine synthesis
GO:0031032: actomyosin structure organization
GO:0008121: ubiquinol-cytochrome-c reductase activity
GO:0001844: protein insertion into mitochondrial membrane during induction of apoptosis
GO:0006777: Mo-molybdopterin cofactor biosynthetic process
GO:0010768: negative regulation of transcription from RNA polymerase II promoter in response to UV-induced DNA damage
GO:0000105: histidine biosynthetic process
GO:0006120: mitochondrial electron transport, NADH to ubiquinone
GO:0005732: small nucleolar ribonucleoprotein complex
GO:0019226: transmission of nerve impulse
GO:0005086: ARF guanyl-nucleotide exchange factor activity
GO:0019089: transmission of virus
GO:0032236: positive regulation of calcium ion transport via store-operated calcium channel activity
GO:0004096: catalase activity
GO:0004470: malic enzyme activity
GO:0051541: elastin metabolic process
GO:0046685: response to arsenic
GO:0045926: negative regulation of growth
GO:0006467: protein thiol-disulfide exchange
GO:0060083: smooth muscle contraction involved in micturition
GO:0004691: cAMP-dependent protein kinase activity
GO:0010613: positive regulation of cardiac muscle hypertrophy
GO:0016219: GDP-dissociation stimulator activity
GO:0004715: non-membrane spanning protein tyrosine kinase activity
GO:0042809: vitamin D receptor binding
GO:0055009: atrial cardiac muscle morphogenesis
GO:0032790: ribosome disassembly
GO:0015917: aminophospholipid transport
GO:0008633: activation of pro-apoptotic gene products
GO:0019276: UDP-N-acetylgalactosamine metabolic process
GO:0043550: regulation of lipid kinase activity
GO:0032862: activation of Rho GTPase activity
GO:0000900: translation repressor activity, nucleic acid binding
GO:0008180: signalosome
GO:0070063: RNA polymerase binding
GO:0003746: translation elongation factor activity
GO:0001514: selenocysteine incorporation
GO:0033014: tetrapyrrole biosynthetic process
GO:0015321: sodium-dependent phosphate transmembrane transporter activity
GO:0050847: progesterone receptor signaling pathway
GO:0031674: I band
GO:0016018: cyclosporin A binding
GO:0004784: superoxide dismutase activity
GO:0019059: initiation of viral infection
GO:0008536: Ran GTPase binding
GO:0010458: exit from mitosis
GO:0005112: Notch binding
GO:0008641: small protein activating enzyme activity
GO:0046755: non-lytic virus budding
GO:0006710: androgen catabolic process
GO:0000049: tRNA binding
GO:0005850: eukaryotic translation initiation factor 2 complex
GO:0005138: interleukin-6 receptor binding
GO:0050819: negative regulation of coagulation
GO:0004590: orotidine-5'-phosphate decarboxylase activity
GO:0017076: purine nucleotide binding
GO:0006418: tRNA aminoacylation for protein translation
GO:0002675: positive regulation of acute inflammatory response
GO:0009249: protein lipoylation
GO:0030203: glycosaminoglycan metabolic process
GO:0060166: olfactory pit development
GO:0004598: peptidylamidoglycolate lyase activity

GO:0004647: phosphoserine phosphatase activity
GO:0007220: Notch receptor processing
GO:0047115: trans-1,2-dihydrobenzene-1,2-diol dehydrogenase activity
GO:0015991: ATP hydrolysis coupled proton transport
GO:0045453: bone resorption
GO:0006520: amino acid metabolic process
GO:0001887: selenium metabolic process
GO:0006471: protein amino acid ADP-ribosylation
GO:0033857: diphosphoinositol-pentakisphosphate kinase activity
GO:0048806: genitalia development
GO:0004915: interleukin-6 receptor activity
GO:0006106: fumarate metabolic process
GO:0004861: cyclin-dependent protein kinase inhibitor activity
GO:0051877: pigment granule aggregation in cell center
GO:0046716: muscle maintenance
GO:0004563: beta-N-acetylhexosaminidase activity
GO:0000178: exosome (RNase complex)
GO:0045931: positive regulation of mitotic cell cycle
GO:0047804: cysteine-S-conjugate beta-lyase activity
GO:0001934: positive regulation of protein amino acid phosphorylation
GO:0006434: seryl-tRNA aminoacylation
GO:0019871: sodium channel inhibitor activity
GO:0047006: 20-alpha-hydroxysteroid dehydrogenase activity
GO:0000832: inositol hexakisphosphate 5-kinase activity
GO:0019752: carboxylic acid metabolic process
GO:0000077: DNA damage checkpoint
GO:0005498: sterol carrier activity
GO:0045116: protein neddylation
GO:0016407: acetyltransferase activity
GO:0008831: dTDP-4-dehydrorhamnose reductase activity
GO:0009982: pseudouridine synthase activity
GO:0001757: somite specification
GO:0033989: 3alpha,7alpha,12alpha-trihydroxy-5beta-cholest-24-enoyl-CoA hydratase activity
GO:0019787: small conjugating protein ligase activity
GO:0000027: ribosomal large subunit assembly
GO:0006110: regulation of glycolysis
GO:0009165: nucleotide biosynthetic process
GO:0015934: large ribosomal subunit
GO:0033044: regulation of chromosome organization
GO:0001888: glucuronyl-galactosyl-proteoglycan 4-alpha-N-acetylglucosaminyltransferase activity
GO:0043024: ribosomal small subunit binding
GO:0051716: cellular response to stimulus
GO:0032020: ISG15-protein conjugation
GO:0004333: fumarate hydratase activity
GO:0015077: monovalent inorganic cation transmembrane transporter activity
GO:0006730: one-carbon compound metabolic process
GO:0004556: alpha-amylase activity
GO:0006778: porphyrin metabolic process
GO:0017119: Golgi transport complex
GO:0004190: aspartic-type endopeptidase activity
GO:0046886: positive regulation of hormone biosynthetic process
GO:0003874: 6-pyruvoyltetrahydropterin synthase activity
GO:0004981: muscarinic acetylcholine receptor activity
GO:0031118: rRNA pseudouridine synthesis
GO:0006689: ganglioside catabolic process
GO:0034263: autophagy in response to ER overload
GO:0055007: cardiac muscle cell differentiation
GO:0003743: translation initiation factor activity
GO:0031698: beta-2 adrenergic receptor binding
GO:0008441: 3'(2'),5'-bisphosphate nucleotidase activity
GO:0008652: amino acid biosynthetic process
GO:0031575: G1/S transition checkpoint
GO:0031362: anchored to external side of plasma membrane
GO:0000279: M phase
GO:0042780: tRNA 3'-end processing
GO:0051000: positive regulation of nitric-oxide synthase activity
GO:0004843: ubiquitin-specific protease activity
GO:0016773: phosphotransferase activity, alcohol group as acceptor
GO:0006583: melanin biosynthetic process from tyrosine
GO:0005678: chromatin assembly complex
GO:0006970: response to osmotic stress
GO:0043666: regulation of phosphoprotein phosphatase activity
GO:0042787: protein ubiquitination during ubiquitin-dependent protein catabolic process
GO:0008389: coumarin 7-hydroxylase activity
GO:0004105: choline-phosphate cytidylyltransferase activity
GO:0043015: gamma-tubulin binding
GO:0043560: insulin receptor substrate binding
GO:0005751: mitochondrial respiratory chain complex IV
GO:0004581: dolichyl-phosphate beta-glucosyltransferase activity
GO:0008250: oligosaccharyltransferase complex
GO:0000276: mitochondrial proton-transporting ATP synthase complex, coupling factor F(o)
GO:0032473: external side of mitochondrial outer membrane
GO:0048547: gut morphogenesis
GO:0005010: insulin-like growth factor receptor activity
GO:0008889: glycerophosphodiester phosphodiesterase activity
GO:0015020: glucuronosyltransferase activity
GO:0004132: dCMP deaminase activity
GO:0032344: regulation of aldosterone metabolic process
GO:0043297: apical junction assembly
GO:0007184: SMAD protein nuclear translocation
GO:0005534: galactose binding
GO:0045039: protein import into mitochondrial inner membrane
GO:0005153: interleukin-8 receptor binding
GO:0016255: attachment of GPI anchor to protein
GO:0004969: histamine receptor activity
GO:0000307: cyclin-dependent protein kinase holoenzyme complex
GO:0042171: lysophosphatidic acid acyltransferase activity
GO:0004485: methylcrotonoyl-CoA carboxylase activity
GO:0033180: proton-transporting V-type ATPase, V1 domain
GO:0016494: C-X-C chemokine receptor activity
GO:0004970: ionotropic glutamate receptor activity
GO:0015446: arsenite transmembrane-transporting ATPase activity
GO:0002009: morphogenesis of an epithelium
GO:0004948: calcitonin receptor activity
GO:0033269: internode region of axon
GO:0009841: mitochondrial endopeptidase Clp complex
GO:0005246: calcium channel regulator activity
GO:0005686: snRNP U2
GO:0042765: GPI-anchor transamidase complex
GO:0008634: negative regulation of survival gene product expression
GO:0035248: alpha-1,4-N-acetylgalactosaminyltransferase activity
GO:0005068: transmembrane receptor protein tyrosine kinase adaptor protein activity
GO:0046839: phospholipid dephosphorylation
GO:0008157: protein phosphatase 1 binding
GO:0005753: mitochondrial proton-transporting ATP synthase complex
GO:0042771: DNA damage response, signal transduction by p53 class mediator resulting in induction of apoptosis
GO:0000792: heterochromatin
GO:0017101: aminoacyl-tRNA synthetase multienzyme complex
GO:0030586: [methionine synthase] reductase activity
GO:0045837: negative regulation of membrane potential
GO:0001573: ganglioside metabolic process
GO:0051028: mRNA transport
GO:0005579: membrane attack complex
GO:0006626: protein targeting to mitochondrion
GO:0008206: bile acid metabolic process
GO:0004865: protein serine/threonine phosphatase inhibitor activity
GO:0000398: nuclear mRNA splicing, via spliceosome

GO:0021537: telencephalon development
GO:0008049: male courtship behavior
GO:0060081: membrane hyperpolarization
GO:0006556: S-adenosylmethionine biosynthetic process
GO:0015319: sodium:inorganic phosphate symporter activity
GO:0006744: ubiquinone biosynthetic process
GO:0021554: optic nerve development
GO:0006400: tRNA modification
GO:0032767: copper-dependent protein binding
GO:0032467: positive regulation of cytokinesis
GO:0051536: iron-sulfur cluster binding
GO:0000386: second spliceosomal transesterification activity
GO:0006790: sulfur metabolic process
GO:0048675: axon extension
GO:0006104: succinyl-CoA metabolic process
GO:0000070: mitotic sister chromatid segregation
GO:0012506: vesicle membrane
GO:0019885: antigen processing and presentation of endogenous peptide antigen via MHC class I
GO:0042587: glycogen granule
GO:0003823: antigen binding
GO:0035267: NuA4 histone acetyltransferase complex
GO:0007158: neuron adhesion
GO:0030299: cholesterol absorption
GO:0004012: phospholipid-translocating ATPase activity
GO:0007281: germ cell development
GO:0042296: ISG15 ligase activity
GO:0048270: methionine adenosyltransferase regulator activity
GO:0007400: neuroblast fate determination
GO:0051881: regulation of mitochondrial membrane potential
GO:0019992: diacylglycerol binding
GO:0030001: metal ion transport
GO:0002763: positive regulation of myeloid leukocyte differentiation
GO:0000272: polysaccharide catabolic process
GO:0050816: phosphothreonine binding
GO:0009060: aerobic respiration
GO:0007157: heterophilic cell adhesion
GO:0006552: leucine catabolic process
GO:0005487: nucleocytoplasmic transporter activity
GO:0045668: negative regulation of osteoblast differentiation
GO:0045010: actin nucleation
GO:0042776: mitochondrial ATP synthesis coupled proton transport
GO:0004477: methenyltetrahydrofolate cyclohydrolase activity
GO:0051823: regulation of synapse structural plasticity
GO:0060165: regulation of timing of subpallium neuron differentiation
GO:0005900: oncostatin-M receptor complex
GO:0045651: positive regulation of macrophage differentiation
GO:0042221: response to chemical stimulus
GO:0006360: transcription from RNA polymerase I promoter
GO:0021779: oligodendrocyte cell fate commitment
GO:0035173: histone kinase activity
GO:0004066: asparagine synthase (glutamine-hydrolyzing) activity
GO:0001516: prostaglandin biosynthetic process
GO:0009396: folic acid and derivative biosynthetic process
GO:0016409: palmitoyltransferase activity
GO:0004311: farnesyltranstransferase activity
GO:0032446: protein modification by small protein conjugation
GO:0016272: prefoldin complex
GO:0006020: inositol metabolic process
GO:0003755: peptidyl-prolyl cis-trans isomerase activity
GO:0043183: vascular endothelial growth factor receptor 1 binding
GO:0018106: peptidyl-histidine phosphorylation
GO:0005672: transcription factor TFIIA complex
GO:0045226: extracellular polysaccharide biosynthetic process
GO:0006401: RNA catabolic process
GO:0006098: pentose-phosphate shunt
GO:0004372: glycine hydroxymethyltransferase activity
GO:0008033: tRNA processing
GO:0000184: nuclear-transcribed mRNA catabolic process, nonsense-mediated decay
GO:0007193: inhibition of adenylate cyclase activity by G-protein signaling
GO:0016706: oxidoreductase activity, acting on paired donors, with incorporation or reduction of molecular oxygen, 2-oxoglutarate as one donor, and incorporation of one atom each of oxygen into both donors
GO:0005663: DNA replication factor C complex
GO:0048147: negative regulation of fibroblast proliferation
GO:0033178: proton-transporting two-sector ATPase complex, catalytic domain
GO:0031323: regulation of cellular metabolic process
GO:0030628: pre-mRNA 3'-splice site binding
GO:0005947: mitochondrial alpha-ketoglutarate dehydrogenase complex
GO:0045930: negative regulation of mitotic cell cycle
GO:0008235: metalloexopeptidase activity
GO:0008047: enzyme activator activity
GO:0046966: thyroid hormone receptor binding
GO:0000120: RNA polymerase I transcription factor complex
GO:0006611: protein export from nucleus
GO:0004830: tryptophan-tRNA ligase activity
GO:0005674: transcription factor TFIIF complex
GO:0005655: nucleolar ribonuclease P complex
GO:0050790: regulation of catalytic activity
GO:0004129: cytochrome-c oxidase activity
GO:0004331: fructose-2,6-bisphosphate 2-phosphatase activity
GO:0006265: DNA topological change
GO:0060082: eye blink reflex
GO:0030218: erythrocyte differentiation
GO:0032391: photoreceptor connecting cilium
GO:0035110: leg morphogenesis
GO:0001669: acrosome
GO:0016458: gene silencing
GO:0030433: ER-associated protein catabolic process
GO:0046541: saliva secretion
GO:0004810: tRNA adenylyltransferase activity
GO:0004060: arylamine N-acetyltransferase activity
GO:0032402: melanosome transport
GO:0003918: DNA topoisomerase (ATP-hydrolyzing) activity
GO:0000038: very-long-chain fatty acid metabolic process
GO:0060047: heart contraction
GO:0009405: pathogenesis
GO:0044445: cytosolic part
GO:0006089: lactate metabolic process
GO:0004588: orotate phosphoribosyltransferase activity
GO:0003870: 5-aminolevulinate synthase activity
GO:0004090: carbonyl reductase (NADPH) activity
GO:0032027: myosin light chain binding
GO:0015270: dihydropyridine-sensitive calcium channel activity
GO:0002248: connective tissue replacement during inflammatory response
GO:0048496: maintenance of organ identity
GO:0007206: activation of phospholipase C activity by metabotropic glutamate receptor signaling pathway
GO:0050220: prostaglandin-E synthase activity
GO:0015986: ATP synthesis coupled proton transport
GO:0006878: cellular copper ion homeostasis
GO:0003844: 1,4-alpha-glucan branching enzyme activity
GO:0045582: positive regulation of T cell differentiation
GO:0016805: dipeptidase activity
GO:0015105: arsenite transmembrane transporter activity
GO:0004597: peptide-aspartate beta-dioxygenase activity
GO:0004063: aryldialkylphosphatase activity
GO:0016769: transferase activity, transferring nitrogenous groups
GO:0050658: RNA transport
GO:0042554: superoxide release
GO:0008593: regulation of Notch signaling pathway

GO:0010259: multicellular organismal aging
GO:0030317: sperm motility
GO:0046034: ATP metabolic process
GO:0018738: S-formylglutathione hydrolase activity
GO:0004923: leukemia inhibitory factor receptor activity
GO:0000245: spliceosome assembly
GO:0004823: leucine-tRNA ligase activity
GO:0042826: histone deacetylase binding
GO:0004033: aldo-keto reductase activity
GO:0046824: positive regulation of nucleocytoplasmic transport
GO:0005890: sodium:potassium-exchanging ATPase complex
GO:0000059: protein import into nucleus, docking
GO:0006474: N-terminal protein amino acid acetylation
GO:0008080: N-acetyltransferase activity
GO:0004423: iduronate-2-sulfatase activity
GO:0032012: regulation of ARF protein signal transduction
GO:0042137: sequestering of neurotransmitter
GO:0001786: phosphatidylserine binding
GO:0005896: interleukin-6 receptor complex
GO:0002026: regulation of the force of heart contraction
GO:0051044: positive regulation of membrane protein ectodomain proteolysis
GO:0015677: copper ion import
GO:0045509: interleukin-27 receptor activity
GO:0006275: regulation of DNA replication
GO:0009399: nitrogen fixation
GO:0004303: estradiol 17-beta-dehydrogenase activity
GO:0006687: glycosphingolipid metabolic process
GO:0004618: phosphoglycerate kinase activity
GO:0040016: embryonic cleavage
GO:0007127: meiosis I
GO:0033981: D-dopachrome decarboxylase activity
GO:0045541: negative regulation of cholesterol biosynthetic process
GO:0004298: threonine-type endopeptidase activity
GO:0005031: tumor necrosis factor receptor activity
GO:0045444: fat cell differentiation
GO:0032088: negative regulation of NF-kappaB transcription factor activity
GO:0006449: regulation of translational termination
GO:0046619: optic placode formation involved in camera-type eye
GO:0006610: ribosomal protein import into nucleus
GO:0003689: DNA clamp loader activity
GO:0045263: proton-transporting ATP synthase complex, coupling factor F(o)
GO:0004807: triose-phosphate isomerase activity
GO:0004831: tyrosine-tRNA ligase activity
GO:0006200: ATP catabolic process
GO:0016998: cell wall catabolic process
GO:0006729: tetrahydrobiopterin biosynthetic process
GO:0000153: cytoplasmic ubiquitin ligase complex
GO:0007608: sensory perception of smell
GO:0006429: leucyl-tRNA aminoacylation
GO:0046579: positive regulation of Ras protein signal transduction
GO:0021530: spinal cord oligodendrocyte cell fate specification
GO:0006622: protein targeting to lysosome
GO:0043619: regulation of transcription from RNA polymerase II promoter in response to oxidative stress
GO:0016212: kynurenine-oxoglutarate transaminase activity
GO:0016149: translation release factor activity, codon specific
GO:0003720: telomerase activity
GO:0034465: response to carbon monoxide
GO:0007243: protein kinase cascade
GO:0005673: transcription factor TFIIE complex
GO:0015238: drug transporter activity
GO:0006436: tryptophanyl-tRNA aminoacylation
GO:0000175: 3'-5'-exoribonuclease activity
GO:0015074: DNA integration
GO:0030007: cellular potassium ion homeostasis
GO:0031307: integral to mitochondrial outer membrane
GO:0031672: A band
GO:0001819: positive regulation of cytokine production
GO:0006446: regulation of translational initiation
GO:0016198: axon choice point recognition
GO:0010001: glial cell differentiation
GO:0003995: acyl-CoA dehydrogenase activity
GO:0008038: neuron recognition
GO:0004003: ATP-dependent DNA helicase activity
GO:0042446: hormone biosynthetic process
GO:0019829: cation-transporting ATPase activity
GO:0046949: acyl-CoA biosynthetic process
GO:0003701: RNA polymerase I transcription factor activity
GO:0030529: ribonucleoprotein complex
GO:0016226: iron-sulfur cluster assembly
GO:0022010: myelination in the central nervous system
GO:0004776: succinate-CoA ligase (GDP-forming) activity
GO:0031252: cell leading edge
GO:0004428: inositol or phosphatidylinositol kinase activity
GO:0007292: female gamete generation
GO:0004998: transferrin receptor activity
GO:0030041: actin filament polymerization
GO:0006437: tyrosyl-tRNA aminoacylation
GO:0004164: diphthine synthase activity
GO:0003678: DNA helicase activity
GO:0005747: mitochondrial respiratory chain complex I
GO:0004167: dopachrome isomerase activity
GO:0006325: establishment or maintenance of chromatin architecture
GO:0016303: 1-phosphatidylinositol-3-kinase activity
GO:0016281: eukaryotic translation initiation factor 4F complex
GO:0016810: hydrolase activity, acting on carbon-nitrogen (but not peptide) bonds
GO:0030240: muscle thin filament assembly
GO:0031202: RNA splicing factor activity, transesterification mechanism
GO:0043022: ribosome binding
GO:0045821: positive regulation of glycolysis
GO:0048102: autophagic cell death
GO:0006487: protein amino acid N-linked glycosylation
GO:0034235: GPI anchor binding
GO:0030983: mismatched DNA binding
GO:0007017: microtubule-based process
GO:0045730: respiratory burst
GO:0019538: protein metabolic process
GO:0042404: thyroid hormone catabolic process
GO:0019048: virus-host interaction
GO:0006942: regulation of striated muscle contraction
GO:0009628: response to abiotic stimulus
GO:0042149: cellular response to glucose starvation
GO:0031405: lipoic acid binding
GO:0003883: CTP synthase activity
GO:0042623: ATPase activity, coupled
GO:0005697: telomerase holoenzyme complex
GO:0003857: 3-hydroxyacyl-CoA dehydrogenase activity
GO:0050576: 3-keto-steroid reductase activity
GO:0016818: hydrolase activity, acting on acid anhydrides, in phosphorus-containing anhydrides
GO:0021527: spinal cord association neuron differentiation
GO:0006419: alanyl-tRNA aminoacylation
#1083IR 51 GO:0015194: L-serine transmembrane transporter activity
GO:0033596: TSC1-TSC2 complex
GO:0047131: saccharopine dehydrogenase (NAD+, L-glutamate-forming) activity
GO:0015184: L-cystine transmembrane transporter activity
GO:0015195: L-threonine transmembrane transporter activity
GO:0034590: L-hydroxyproline transmembrane transporter activity
GO:0015193: L-proline transmembrane transporter activity

		GO:0050957: equilibrioception
		GO:0008392: arachidonic acid epoxygenase activity
		GO:0034597: phosphatidyl-inositol-4,5-bisphosphate 4-phosphatase activity
		GO:0005883: neurofilament
		GO:0006166: purine ribonucleoside salvage
		GO:0008090: retrograde axon cargo transport
		GO:0008143: poly(A) binding
		GO:0004422: hypoxanthine phosphoribosyltransferase activity
		GO:0015824: proline transport
		GO:0015825: L-serine transport
		GO:0008266: poly(U) binding
		GO:0016814: hydrolase activity, acting on carbon-nitrogen (but not peptide) bonds, in cyclic amidines
		GO:0046847: filopodium formation
		GO:0033693: neurofilament bundle assembly
		GO:0030201: heparan sulfate proteoglycan metabolic process
		GO:0031616: spindle pole centrosome
		GO:0003835: beta-galactoside alpha-2,6-sialyltransferase activity
		GO:0030516: regulation of axon extension
		GO:0046907: intracellular transport
		GO:0031223: auditory behavior
		GO:0000800: lateral element
		GO:0007098: centrosome cycle
		GO:0030506: ankyrin binding
		GO:0006913: nucleocytoplasmic transport
		GO:0001750: photoreceptor outer segment
		GO:0045162: clustering of voltage-gated sodium channels
		GO:0007130: synaptonemal complex assembly
		GO:0015826: threonine transport
		GO:0006536: glutamate metabolic process
		GO:0006996: organelle organization
		GO:0015811: L-cystine transport
		GO:0000940: outer kinetochore of condensed chromosome
		GO:0004926: non-G-protein coupled 7TM receptor activity
		GO:0007143: female meiosis
		GO:0005267: potassium channel activity
		GO:0010226: response to lithium ion
		GO:0010826: negative regulation of centrosome duplication
		GO:0005844: polysome
		GO:0050750: low-density lipoprotein receptor binding
		GO:0004971: alpha-amino-3-hydroxy-5-methyl-4-isoxazole propionate selective glutamate receptor activity
		GO:0034589: hydroxyproline transport
		GO:0048667: cell morphogenesis involved in neuron differentiation
		GO:0005041: low-density lipoprotein receptor activity
		GO:0004908: interleukin-1 receptor activity
#1051IR	38	GO:0006891: intra-Golgi vesicle-mediated transport
		GO:0045104: intermediate filament cytoskeleton organization
		GO:0005523: tropomyosin binding
		GO:0002891: positive regulation of immunoglobulin mediated immune response
		GO:0014731: spectrin-associated cytoskeleton
		GO:0006893: Golgi to plasma membrane transport
		GO:0031256: leading edge membrane
		GO:0015327: cystine:glutamate antiporter activity
		GO:0045055: regulated secretory pathway
		GO:0030507: spectrin binding
		GO:0042462: eye photoreceptor cell development
		GO:0030239: myofibril assembly
		GO:0004435: phosphoinositide phospholipase C activity
		GO:0005496: steroid binding
		GO:0000156: two-component response regulator activity
		GO:0008091: spectrin
		GO:0060084: synaptic transmission involved in micturition
		GO:0010839: negative regulation of keratinocyte proliferation
		GO:0030016: myofibril
		GO:0007352: zygotic determination of dorsal/ventral axis
		GO:0019215: intermediate filament binding
		GO:0030259: lipid glycosylation
		GO:0033005: positive regulation of mast cell activation
		GO:0016180: snRNA processing
		GO:0005577: fibrinogen complex
		GO:0016235: aggresome
		GO:0032039: integrator complex
		GO:0035095: behavioral response to nicotine
		GO:0006198: cAMP catabolic process
		GO:0030140: trans-Golgi network transport vesicle
		GO:0005024: transforming growth factor beta receptor activity
		GO:0000160: two-component signal transduction system (phosphorelay)
		GO:0060053: neurofilament cytoskeleton
		GO:0060369: positive regulation of Fc receptor mediated stimulatory signaling pathway
		GO:0046488: phosphatidylinositol metabolic process
		GO:0048812: neurite morphogenesis
		GO:0048041: focal adhesion formation
		GO:0002860: positive regulation of natural killer cell mediated cytotoxicity directed against tumor cell target
#1063IR	694	GO:0009100: glycoprotein metabolic process
		GO:0034446: substrate adhesion-dependent cell spreading
		GO:0001921: positive regulation of receptor recycling
		GO:0043067: regulation of programmed cell death
		GO:0051707: response to other organism
		GO:0008652: cellular amino acid biosynthetic process
		GO:0001702: gastrulation with mouth forming second
		GO:0070244: negative regulation of thymocyte apoptosis
		GO:0007631: feeding behavior
		GO:0002001: renin secretion into blood stream
		GO:0046111: xanthine biosynthetic process
		GO:0019229: regulation of vasoconstriction
		GO:0044430: cytoskeletal part
		GO:0000724: double-strand break repair via homologous recombination
		GO:0035385: Roundabout signaling pathway
		GO:0008589: regulation of smoothened signaling pathway
		GO:0005451: monovalent cation:hydrogen antiporter activity
		GO:0019863: IgE binding
		GO:0019067: viral assembly, maturation, egress, and release
		GO:0042581: specific granule
		GO:0009267: cellular response to starvation
		GO:0030169: low-density lipoprotein particle binding
		GO:0001932: regulation of protein phosphorylation
		GO:0060347: heart trabecula formation
		GO:0009258: 10-formyltetrahydrofolate catabolic process
		GO:0090024: negative regulation of neutrophil chemotaxis
		GO:0060512: prostate gland morphogenesis
		GO:0042588: zymogen granule
		GO:0006270: DNA-dependent DNA replication initiation
		GO:0060421: positive regulation of heart growth
		GO:0007129: synapsis
		GO:0007249: I-kappaB kinase/NF-kappaB cascade
		GO:0015879: carnitine transport
		GO:0007263: nitric oxide mediated signal transduction
		GO:0043518: negative regulation of DNA damage response, signal transduction by p53 class mediator
		GO:0034188: apolipoprotein A-I receptor activity
		GO:0034371: chylomicron remodeling
		GO:0009743: response to carbohydrate stimulus
		GO:0042405: nuclear inclusion body
		GO:0031663: lipopolysaccharide-mediated signaling pathway
		GO:0006379: mRNA cleavage
		GO:0016064: immunoglobulin mediated immune response
		GO:0035329: hippo signaling cascade
		GO:0002314: germinal center B cell differentiation
		GO:0001946: lymphangiogenesis

GO:0071333: cellular response to glucose stimulus
GO:0090103: cochlea morphogenesis
GO:0045071: negative regulation of viral genome replication
GO:0032494: response to peptidoglycan
GO:0050772: positive regulation of axonogenesis
GO:0019894: kinesin binding
GO:0000123: histone acetyltransferase complex
GO:0021510: spinal cord development
GO:0035602: fibroblast growth factor receptor signaling pathway involved in negative regulation of apoptosis in bone marrow
GO:0051930: regulation of sensory perception of pain
GO:0046950: cellular ketone body metabolic process
GO:0015174: basic amino acid transmembrane transporter activity
GO:0002790: peptide secretion
GO:0022008: neurogenesis
GO:0000299: integral to membrane of membrane fraction
GO:0050925: negative regulation of negative chemotaxis
GO:0060527: prostate epithelial cord arborization involved in prostate glandular acinus morphogenesis
GO:0030837: negative regulation of actin filament polymerization
GO:0030676: Rac guanyl-nucleotide exchange factor activity
GO:0040017: positive regulation of locomotion
GO:0071385: cellular response to glucocorticoid stimulus
GO:0035024: negative regulation of Rho protein signal transduction
GO:0060412: ventricular septum morphogenesis
GO:0016594: glycine binding
GO:0050999: regulation of nitric-oxide synthase activity
GO:0070141: response to UV-A
GO:0009888: tissue development
GO:0046696: lipopolysaccharide receptor complex
GO:0060169: negative regulation of adenosine receptor signaling pathway
GO:0007501: mesodermal cell fate specification
GO:0035235: ionotropic glutamate receptor signaling pathway
GO:0043046: DNA methylation involved in gamete generation
GO:0008331: high voltage-gated calcium channel activity
GO:0006497: protein lipidation
GO:0043129: surfactant homeostasis
GO:0021769: orbitofrontal cortex development
GO:0046697: decidualization
GO:0004123: cystathionine gamma-lyase activity
GO:0015038: glutathione disulfide oxidoreductase activity
GO:0042165: neurotransmitter binding
GO:0048562: embryonic organ morphogenesis
GO:0000062: fatty-acyl-CoA binding
GO:0034752: cytosolic aryl hydrocarbon receptor complex
GO:0033280: response to vitamin D
GO:0045130: keratan sulfotransferase activity
GO:0050727: regulation of inflammatory response
GO:0004117: calmodulin-dependent cyclic-nucleotide phosphodiesterase activity
GO:0051168: nuclear export
GO:0060501: positive regulation of epithelial cell proliferation involved in lung morphogenesis
GO:0051225: spindle assembly
GO:0060688: regulation of morphogenesis of a branching structure
GO:0033691: sialic acid binding
GO:0005283: sodium:amino acid symporter activity
GO:0060024: rhythmic synaptic transmission
GO:0034361: very-low-density lipoprotein particle
GO:0070189: kynurenine metabolic process
GO:0001954: positive regulation of cell-matrix adhesion
GO:0001505: regulation of neurotransmitter levels
GO:0016199: axon midline choice point recognition
GO:0001931: uropod
GO:0004500: dopamine beta-monooxygenase activity
GO:0045502: dynein binding
GO:0070814: hydrogen sulfide biosynthetic process
GO:0047273: galactosylgalactosylglucosylceramide beta-D-acetylgalactosaminyltransferase activity
GO:0060687: regulation of branching involved in prostate gland morphogenesis
GO:2000124: regulation of endocannabinoid signaling pathway
GO:0008489: UDP-galactose:glucosylceramide beta-1,4-galactosyltransferase activity
GO:0042135: neurotransmitter catabolic process
GO:0046875: ephrin receptor binding
GO:0008611: ether lipid biosynthetic process
GO:0045907: positive regulation of vasoconstriction
GO:0030544: Hsp70 protein binding
GO:0046103: inosine biosynthetic process
GO:0005776: autophagic vacuole
GO:0009880: embryonic pattern specification
GO:0045187: regulation of circadian sleep/wake cycle, sleep
GO:0045190: isotype switching
GO:0051497: negative regulation of stress fiber assembly
GO:0032455: nerve growth factor processing
GO:0000981: sequence-specific DNA binding RNA polymerase II transcription factor activity
GO:0003149: membranous septum morphogenesis
GO:0001553: luteinization
GO:0051041: positive regulation of calcium-independent cell-cell adhesion
GO:0070493: thrombin receptor signaling pathway
GO:0050667: homocysteine metabolic process
GO:0010744: positive regulation of macrophage derived foam cell differentiation
GO:0042339: keratan sulfate metabolic process
GO:0032902: nerve growth factor production
GO:0004601: peroxidase activity
GO:0000722: telomere maintenance via recombination
GO:0050613: delta14-sterol reductase activity
GO:0000038: very long-chain fatty acid metabolic process
GO:0006534: cysteine metabolic process
GO:0050880: regulation of blood vessel size
GO:0022028: tangential migration from the subventricular zone to the olfactory bulb
GO:0052547: regulation of peptidase activity
GO:0045899: positive regulation of RNA polymerase II transcriptional preinitiation complex assembly
GO:0045806: negative regulation of endocytosis
GO:0071222: cellular response to lipopolysaccharide
GO:0016601: Rac protein signal transduction
GO:0003986: acetyl-CoA hydrolase activity
GO:0032436: positive regulation of proteasomal ubiquitin-dependent protein catabolic process
GO:0048406: nerve growth factor binding
GO:0031982: vesicle
GO:0030277: maintenance of gastrointestinal epithelium
GO:0048185: activin binding
GO:0005976: polysaccharide metabolic process
GO:0001875: lipopolysaccharide receptor activity
GO:0060907: positive regulation of macrophage cytokine production
GO:0016585: chromatin remodeling complex
GO:0021847: ventricular zone neuroblast division
GO:0009605: response to external stimulus
GO:0048244: phytanoyl-CoA dioxygenase activity
GO:0001518: voltage-gated sodium channel complex
GO:0032507: maintenance of protein location in cell
GO:0080146: L-cysteine desulfhydrase activity
GO:0021696: cerebellar cortex morphogenesis
GO:0055010: ventricular cardiac muscle tissue morphogenesis
GO:0001741: XY body
GO:0031749: D2 dopamine receptor binding
GO:0048853: forebrain morphogenesis
GO:0060203: clathrin sculpted glutamate transport vesicle membrane
GO:0051925: regulation of calcium ion transport via voltage-gated calcium channel activity
GO:0060059: embryonic retina morphogenesis in camera-type eye
GO:0033688: regulation of osteoblast proliferation
GO:0001662: behavioral fear response
GO:0060836: lymphatic endothelial cell differentiation

GO:0032589: neuron projection membrane
GO:0034374: low-density lipoprotein particle remodeling
GO:0046101: hypoxanthine biosynthetic process
GO:0003326: pancreatic A cell fate commitment
GO:0000188: inactivation of MAPK activity
GO:0071504: cellular response to heparin
GO:0034587: piRNA metabolic process
GO:0061364: apoptosis involved in luteolysis
GO:0004602: glutathione peroxidase activity
GO:0002636: positive regulation of germinal center formation
GO:0017059: serine C-palmitoyltransferase complex
GO:0045335: phagocytic vesicle
GO:0043395: heparan sulfate proteoglycan binding
GO:0043621: protein self-association
GO:0048554: positive regulation of metalloenzyme activity
GO:0034185: apolipoprotein binding
GO:0034046: poly(G) RNA binding
GO:0051963: regulation of synaptogenesis
GO:0021520: spinal cord motor neuron cell fate specification
GO:0043522: leucine zipper domain binding
GO:0015485: cholesterol binding
GO:0007423: sensory organ development
GO:0050910: detection of mechanical stimulus involved in sensory perception of sound
GO:0003350: pulmonary myocardium development
GO:0016236: macroautophagy
GO:0006637: acyl-CoA metabolic process
GO:0034109: homotypic cell-cell adhesion
GO:0045750: positive regulation of S phase of mitotic cell cycle
GO:0016772: transferase activity, transferring phosphorus-containing groups
GO:0071672: negative regulation of smooth muscle cell chemotaxis
GO:0030857: negative regulation of epithelial cell differentiation
GO:0030240: skeletal muscle thin filament assembly
GO:0004321: fatty-acyl-CoA synthase activity
GO:0033631: cell-cell adhesion mediated by integrin
GO:0051973: positive regulation of telomerase activity
GO:0019369: arachidonic acid metabolic process
GO:0042392: sphingosine-1-phosphate phosphatase activity
GO:0006848: pyruvate transport
GO:0048013: ephrin receptor signaling pathway
GO:0043083: synaptic cleft
GO:0001964: startle response
GO:0010035: response to inorganic substance
GO:0031513: nonmotile primary cilium
GO:0048565: digestive tract development
GO:0046777: protein autophosphorylation
GO:0043616: keratinocyte proliferation
GO:0035265: organ growth
GO:0055074: calcium ion homeostasis
GO:0060595: fibroblast growth factor receptor signaling pathway involved in mammary gland specification
GO:0071229: cellular response to acid
GO:0030285: integral to synaptic vesicle membrane
GO:0032488: Cdc42 protein signal transduction
GO:0006471: protein ADP-ribosylation
GO:0005006: epidermal growth factor receptor activity
GO:0030323: respiratory tube development
GO:0005548: phospholipid transporter activity
GO:0060667: branch elongation involved in salivary gland morphogenesis
GO:0008273: calcium, potassium:sodium antiporter activity
GO:0048791: calcium ion-dependent exocytosis of neurotransmitter
GO:0004441: inositol-1,4-bisphosphate 1-phosphatase activity
GO:0061001: regulation of dendritic spine morphogenesis
GO:0019217: regulation of fatty acid metabolic process
GO:0030675: Rac GTPase activator activity
GO:0003847: 1-alkyl-2-acetylglycerophosphocholine esterase activity
GO:0048268: clathrin coat assembly
GO:0010942: positive regulation of cell death
GO:0060414: aorta smooth muscle tissue morphogenesis
GO:0035604: fibroblast growth factor receptor signaling pathway involved in positive regulation of cell proliferation in bone marrow
GO:0045987: positive regulation of smooth muscle contraction
GO:0004522: pancreatic ribonuclease activity
GO:0008046: axon guidance receptor activity
GO:0004716: receptor signaling protein tyrosine kinase activity
GO:0034436: glycoprotein transport
GO:0002042: cell migration involved in sprouting angiogenesis
GO:0051414: response to cortisol stimulus
GO:0045569: TRAIL binding
GO:0042577: lipid phosphatase activity
GO:0007519: skeletal muscle tissue development
GO:0002903: negative regulation of B cell apoptosis
GO:0043088: regulation of Cdc42 GTPase activity
GO:0033484: nitric oxide homeostasis
GO:0021860: pyramidal neuron development
GO:0021549: cerebellum development
GO:0044330: canonical Wnt receptor signaling pathway involved in positive regulation of wound healing
GO:0030510: regulation of BMP signaling pathway
GO:0031116: positive regulation of microtubule polymerization
GO:0060349: bone morphogenesis
GO:0048593: camera-type eye morphogenesis
GO:0070301: cellular response to hydrogen peroxide
GO:0045749: negative regulation of S phase of mitotic cell cycle
GO:0090072: positive regulation of sodium ion transport via voltage-gated sodium channel activity
GO:0003215: cardiac right ventricle morphogenesis
GO:0015307: drug:hydrogen antiporter activity
GO:0021631: optic nerve morphogenesis
GO:0030849: autosome
GO:0035255: ionotropic glutamate receptor binding
GO:0009072: aromatic amino acid family metabolic process
GO:0050966: detection of mechanical stimulus involved in sensory perception of pain
GO:0051297: centrosome organization
GO:0051984: positive regulation of chromosome segregation
GO:0016167: glial cell line-derived neurotrophic factor receptor activity
GO:0010827: regulation of glucose transport
GO:0030828: positive regulation of cGMP biosynthetic process
GO:0060442: branching involved in prostate gland morphogenesis
GO:0030148: sphingolipid biosynthetic process
GO:0032024: positive regulation of insulin secretion
GO:0001960: negative regulation of cytokine-mediated signaling pathway
GO:0043121: neurotrophin binding
GO:0010593: negative regulation of lamellipodium assembly
GO:0048730: epidermis morphogenesis
GO:0060992: response to fungicide
GO:0032956: regulation of actin cytoskeleton organization
GO:0015227: acyl carnitine transporter activity
GO:0071230: cellular response to amino acid stimulus
GO:0060201: clathrin sculpted acetylcholine transport vesicle membrane
GO:0045576: mast cell activation
GO:0021575: hindbrain morphogenesis
GO:0005681: spliceosomal complex
GO:0032201: telomere maintenance via semi-conservative replication
GO:0010468: regulation of gene expression
GO:0008542: visual learning
GO:0005369: taurine:sodium symporter activity
GO:0005815: microtubule organizing center
GO:0001975: response to amphetamine
GO:0016290: palmitoyl-CoA hydrolase activity
GO:0071445: cellular response to protein stimulus
GO:0016444: somatic cell DNA recombination

GO:0005477: pyruvate secondary active transmembrane transporter activity
GO:0050062: long-chain-fatty-acyl-CoA reductase activity
GO:0008188: neuropeptide receptor activity
GO:0007589: body fluid secretion
GO:0042627: chylomicron
GO:0043006: activation of phospholipase A2 activity by calcium-mediated signaling
GO:0048489: synaptic vesicle transport
GO:0034380: high-density lipoprotein particle assembly
GO:0051412: response to corticosterone stimulus
GO:0042584: chromaffin granule membrane
GO:0032367: intracellular cholesterol transport
GO:2000096: positive regulation of Wnt receptor signaling pathway, planar cell polarity pathway
GO:0021836: chemorepulsion involved in postnatal olfactory bulb interneuron migration
GO:0048169: regulation of long-term neuronal synaptic plasticity
GO:0004713: protein tyrosine kinase activity
GO:0070367: negative regulation of hepatocyte differentiation
GO:0071363: cellular response to growth factor stimulus
GO:0000979: RNA polymerase II core promoter sequence-specific DNA binding
GO:0043433: negative regulation of transcription factor activity
GO:0007346: regulation of mitotic cell cycle
GO:0017134: fibroblast growth factor binding
GO:0017156: calcium ion-dependent exocytosis
GO:0050690: regulation of defense response to virus by virus
GO:0004972: N-methyl-D-aspartate selective glutamate receptor activity
GO:0050929: induction of negative chemotaxis
GO:0016155: formyltetrahydrofolate dehydrogenase activity
GO:0017129: triglyceride binding
GO:0045861: negative regulation of proteolysis
GO:0044328: canonical Wnt receptor signaling pathway involved in positive regulation of endothelial cell migration
GO:0014063: negative regulation of serotonin secretion
GO:0021972: corticospinal neuron axon guidance through spinal cord
GO:0004502: kynurenine 3-monooxygenase activity
GO:0030345: structural constituent of tooth enamel
GO:0033993: response to lipid
GO:0018272: protein-pyridoxal-5-phosphate linkage via peptidyl-N6-pyridoxal phosphate-L-lysine
GO:0015630: microtubule cytoskeleton
GO:0046061: dATP catabolic process
GO:0031146: SCF-dependent proteasomal ubiquitin-dependent protein catabolic process
GO:0071359: cellular response to dsRNA
GO:0002158: osteoclast proliferation
GO:0007189: activation of adenylate cyclase activity by G-protein signaling pathway
GO:0048266: behavioral response to pain
GO:0033700: phospholipid efflux
GO:0019005: SCF ubiquitin ligase complex
GO:0021891: olfactory bulb interneuron development
GO:0008344: adult locomotory behavior
GO:0010872: regulation of cholesterol esterification
GO:0051480: cytosolic calcium ion homeostasis
GO:0009720: detection of hormone stimulus
GO:0040023: establishment of nucleus localization
GO:0061003: positive regulation of dendritic spine morphogenesis
GO:0046464: acylglycerol catabolic process
GO:0005547: phosphatidylinositol-3,4,5-trisphosphate binding
GO:0043186: P granule
GO:0034041: sterol-transporting ATPase activity
GO:0060056: mammary gland involution
GO:0060915: mesenchymal cell differentiation involved in lung development
GO:0005250: A-type (transient outward) potassium channel activity
GO:0051668: localization within membrane
GO:0048488: synaptic vesicle endocytosis
GO:0042417: dopamine metabolic process
GO:0060029: convergent extension involved in organogenesis
GO:0042089: cytokine biosynthetic process
GO:0016174: NAD(P)H oxidase activity
GO:0060311: negative regulation of elastin catabolic process
GO:0010332: response to gamma radiation
GO:0042058: regulation of epidermal growth factor receptor signaling pathway
GO:0042445: hormone metabolic process
GO:0030235: nitric-oxide synthase regulator activity
GO:0035555: initiation of Roundabout signal transduction
GO:0019344: cysteine biosynthetic process
GO:0090007: regulation of mitotic anaphase
GO:0000096: sulfur amino acid metabolic process
GO:0004121: cystathionine beta-lyase activity
GO:0051150: regulation of smooth muscle cell differentiation
GO:0010875: positive regulation of cholesterol efflux
GO:0060045: positive regulation of cardiac muscle cell proliferation
GO:0030595: leukocyte chemotaxis
GO:0016331: morphogenesis of embryonic epithelium
GO:0042059: negative regulation of epidermal growth factor receptor signaling pathway
GO:0030512: negative regulation of transforming growth factor beta receptor signaling pathway
GO:0000730: DNA recombinase assembly
GO:0015355: secondary active monocarboxylate transmembrane transporter activity
GO:0043274: phospholipase binding
GO:0071805: potassium ion transmembrane transport
GO:0017146: N-methyl-D-aspartate selective glutamate receptor complex
GO:0051017: actin filament bundle assembly
GO:0032410: negative regulation of transporter activity
GO:0015068: glycine amidinotransferase activity
GO:0071300: cellular response to retinoic acid
GO:0005248: voltage-gated sodium channel activity
GO:0008074: guanylate cyclase complex, soluble
GO:0050928: negative regulation of positive chemotaxis
GO:0015226: carnitine transporter activity
GO:0014047: glutamate secretion
GO:0006975: DNA damage induced protein phosphorylation
GO:0043408: regulation of MAPKKK cascade
GO:0070976: TIR domain binding
GO:0046952: ketone body catabolic process
GO:0032091: negative regulation of protein binding
GO:0045920: negative regulation of exocytosis
GO:0017127: cholesterol transporter activity
GO:0008815: citrate (pro-3S)-lyase activity
GO:0010952: positive regulation of peptidase activity
GO:0007157: heterophilic cell-cell adhesion
GO:0051605: protein maturation by peptide bond cleavage
GO:0004677: DNA-dependent protein kinase activity
GO:0042063: gliogenesis
GO:0010044: response to aluminum ion
GO:0071777: positive regulation of cell cycle cytokinesis
GO:0014051: gamma-aminobutyric acid secretion
GO:0035108: limb morphogenesis
GO:0050544: arachidonic acid binding
GO:0043113: receptor clustering
GO:0030888: regulation of B cell proliferation
GO:0090260: negative regulation of retinal ganglion cell axon guidance
GO:0004016: adenylate cyclase activity
GO:0047372: acylglycerol lipase activity
GO:0031659: positive regulation of cyclin-dependent protein kinase activity involved in G1/S
GO:0090288: negative regulation of cellular response to growth factor stimulus
GO:0032497: detection of lipopolysaccharide
GO:0030644: cellular chloride ion homeostasis
GO:0050693: LBD domain binding
GO:0031532: actin cytoskeleton reorganization
GO:0032808: lacrimal gland development
GO:0043981: histone H4-K5 acetylation
GO:0033278: cell proliferation in midbrain

GO:0051058: negative regulation of small GTPase mediated signal transduction
GO:0017158: regulation of calcium ion-dependent exocytosis
GO:0007431: salivary gland development
GO:0048608: reproductive structure development
GO:0046619: optic placode formation involved in camera-type eye formation
GO:0010212: response to ionizing radiation
GO:0051585: negative regulation of dopamine uptake
GO:0008131: primary amine oxidase activity
GO:0019363: pyridine nucleotide biosynthetic process
GO:0007276: gamete generation
GO:0051597: response to methylmercury
GO:0005958: DNA-dependent protein kinase-DNA ligase 4 complex
GO:0003329: pancreatic PP cell fate commitment
GO:0010886: positive regulation of cholesterol storage
GO:0060039: pericardium development
GO:0055100: adiponectin binding
GO:0016600: flotillin complex
GO:0006907: pinocytosis
GO:0071547: piP-body
GO:0004710: MAP/ERK kinase kinase activity
GO:0003956: NAD(P)+-protein-arginine ADP-ribosyltransferase activity
GO:0042524: negative regulation of tyrosine phosphorylation of Stat5 protein
GO:0033327: Leydig cell differentiation
GO:0021754: facial nucleus development
GO:0015057: thrombin receptor activity
GO:0005121: Toll binding
GO:0006465: signal peptide processing
GO:0009651: response to salt stress
GO:0047961: glycine N-acyltransferase activity
GO:0015491: cation:cation antiporter activity
GO:0060449: bud elongation involved in lung branching
GO:0051622: negative regulation of norepinephrine uptake
GO:0008406: gonad development
GO:0060612: adipose tissue development
GO:0001517: N-acetylglucosamine 6-O-sulfotransferase activity
GO:0001561: fatty acid alpha-oxidation
GO:2000016: negative regulation of determination of dorsal identity
GO:0006904: vesicle docking involved in exocytosis
GO:0030122: AP-2 adaptor complex
GO:0016742: hydroxymethyl-, formyl- and related transferase activity
GO:0050902: leukocyte adhesive activation
GO:0031047: gene silencing by RNA
GO:0006261: DNA-dependent DNA replication
GO:0071456: cellular response to hypoxia
GO:0050692: DBD domain binding
GO:0070876: SOSS complex
GO:0006584: catecholamine metabolic process
GO:0001669: acrosomal vesicle
GO:0021679: cerebellar molecular layer development
GO:0032569: gene-specific transcription from RNA polymerase II promoter
GO:0045773: positive regulation of axon extension
GO:0010269: response to selenium ion
GO:0010721: negative regulation of cell development
GO:0006725: cellular aromatic compound metabolic process
GO:0030273: melanin-concentrating hormone receptor activity
GO:0070328: triglyceride homeostasis
GO:0007387: anterior compartment pattern formation
GO:0032314: regulation of Rac GTPase activity
GO:0030349: syntaxin-13 binding
GO:0010551: regulation of gene-specific transcription from RNA polymerase II promoter
GO:0060205: cytoplasmic membrane-bounded vesicle lumen
GO:0010887: negative regulation of cholesterol storage
GO:0030117: membrane coat
GO:0050671: positive regulation of lymphocyte proliferation
GO:0048541: Peyer's patch development
GO:0051612: negative regulation of serotonin uptake
GO:0043069: negative regulation of programmed cell death
GO:0030146: diuresis
GO:0050927: positive regulation of positive chemotaxis
GO:0034103: regulation of tissue remodeling
GO:0050775: positive regulation of dendrite morphogenesis
GO:0033563: dorsal/ventral axon guidance
GO:0010890: positive regulation of sequestering of triglyceride
GO:0080008: CUL4 RING ubiquitin ligase complex
GO:0060313: negative regulation of blood vessel remodeling
GO:0016830: carbon-carbon lyase activity
GO:0045019: negative regulation of nitric oxide biosynthetic process
GO:0043548: phosphatidylinositol 3-kinase binding
GO:0021750: vestibular nucleus development
GO:0007616: long-term memory
GO:0060849: regulation of transcription involved in lymphatic endothelial cell fate commitment
GO:0043206: fibril organization
GO:0048846: axon extension involved in axon guidance
GO:0015697: quaternary ammonium group transport
GO:0018146: keratan sulfate biosynthetic process
GO:0030193: regulation of blood coagulation
GO:0051964: negative regulation of synaptogenesis
GO:0006983: ER overload response
GO:0006790: sulfur compound metabolic process
GO:0006283: transcription-coupled nucleotide-excision repair
GO:0060615: mammary gland bud formation
GO:0003322: pancreatic A cell development
GO:0061037: negative regulation of cartilage development
GO:0032747: positive regulation of interleukin-23 production
GO:0009346: citrate lyase complex
GO:0015347: sodium-independent organic anion transmembrane transporter activity
GO:0015375: glycine:sodium symporter activity
GO:0034437: glycoprotein transporter activity
GO:0045080: positive regulation of chemokine biosynthetic process
GO:0071902: positive regulation of protein serine/threonine kinase activity
GO:0031694: alpha-2A adrenergic receptor binding
GO:0016486: peptide hormone processing
GO:0042327: positive regulation of phosphorylation
GO:0042997: negative regulation of Golgi to plasma membrane protein transport
GO:0043983: histone H4-K12 acetylation
GO:0035603: fibroblast growth factor receptor signaling pathway involved in hemopoiesis
GO:0033138: positive regulation of peptidyl-serine phosphorylation
GO:0008610: lipid biosynthetic process
GO:0008266: poly(U) RNA binding
GO:0005123: death receptor binding
GO:0033344: cholesterol efflux
GO:0045717: negative regulation of fatty acid biosynthetic process
GO:0070652: HAUS complex
GO:0035607: fibroblast growth factor receptor signaling pathway involved in orbitofrontal cortex development
GO:0070100: negative regulation of chemokine-mediated signaling pathway
GO:0006844: acyl carnitine transport
GO:0070858: negative regulation of bile acid biosynthetic process
GO:0043114: regulation of vascular permeability
GO:0048495: Roundabout binding
GO:0043982: histone H4-K8 acetylation
GO:0030215: semaphorin receptor binding
GO:0005030: neurotrophin receptor activity
GO:0006271: DNA strand elongation involved in DNA replication
GO:0008306: associative learning
GO:0010350: cellular response to magnesium starvation
GO:0030288: outer membrane-bounded periplasmic space
GO:0045600: positive regulation of fat cell differentiation

GO:0090136: epithelial cell-cell adhesion
GO:0007256: activation of JNKK activity
GO:0021590: cerebellum maturation
GO:0090083: regulation of inclusion body assembly
GO:0005326: neurotransmitter transporter activity
GO:0001101: response to acid
GO:0032914: positive regulation of transforming growth factor-beta1 production
GO:0004758: serine C-palmitoyltransferase activity
GO:0001950: plasma membrane enriched fraction
GO:0000790: nuclear chromatin
GO:0008184: glycogen phosphorylase activity
GO:0071425: hemopoietic stem cell proliferation
GO:0046618: drug export
GO:0030032: lamellipodium assembly
GO:0048066: developmental pigmentation
GO:0042640: anagen
GO:0044329: canonical Wnt receptor signaling pathway involved in positive regulation of cell-cell adhesion
GO:0003148: outflow tract septum morphogenesis
GO:0032261: purine nucleotide salvage
GO:0014823: response to activity
GO:0002238: response to molecule of fungal origin
GO:0007158: neuron cell-cell adhesion
GO:0010040: response to iron(II) ion
GO:0060407: negative regulation of penile erection
GO:0010642: negative regulation of platelet-derived growth factor receptor signaling pathway
GO:0051902: negative regulation of mitochondrial depolarization
GO:0044424: intracellular part
GO:0031177: phosphopantetheine binding
GO:0042582: azurophil granule
GO:0016291: acyl-CoA thioesterase activity
GO:0004708: MAP kinase kinase activity
GO:0006601: creatine biosynthetic process
GO:0060670: branching involved in embryonic placenta morphogenesis
GO:0007159: leukocyte cell-cell adhesion
GO:0010711: negative regulation of collagen catabolic process
GO:0003151: outflow tract morphogenesis
GO:0042312: regulation of vasodilation
GO:0045197: establishment or maintenance of epithelial cell apical/basal polarity
GO:0000060: protein import into nucleus, translocation
GO:0003851: 2-hydroxyacylsphingosine 1-beta-galactosyltransferase activity
GO:0070435: Shc-EGFR complex
GO:0031576: G2/M transition checkpoint
GO:0001836: release of cytochrome c from mitochondria
GO:0030977: taurine binding
GO:0071813: lipoprotein particle binding
GO:0031394: positive regulation of prostaglandin biosynthetic process
GO:0043405: regulation of MAP kinase activity
GO:0030119: AP-type membrane coat adaptor complex
GO:0043208: glycosphingolipid binding
GO:0071407: cellular response to organic cyclic compound
GO:0030916: otic vesicle formation
GO:0008294: calcium- and calmodulin-responsive adenylate cyclase activity
GO:0061202: clathrin sculpted gamma-aminobutyric acid transport vesicle membrane
GO:0034765: regulation of ion transmembrane transport
GO:0070372: regulation of ERK1 and ERK2 cascade
GO:0032792: negative regulation of CREB transcription factor activity
GO:0001967: suckling behavior
GO:0008513: secondary active organic cation transmembrane transporter activity
GO:0032926: negative regulation of activin receptor signaling pathway
GO:0060529: squamous basal epithelial stem cell differentiation involved in prostate gland acinus development
GO:0032353: negative regulation of hormone biosynthetic process
GO:0007199: G-protein signaling, coupled to cGMP nucleotide second messenger
GO:0002906: negative regulation of mature B cell apoptosis
GO:0048015: phosphatidylinositol-mediated signaling
GO:0060429: epithelium development
GO:0042747: circadian sleep/wake cycle, REM sleep
GO:0050702: interleukin-1 beta secretion
GO:0015949: nucleobase, nucleoside and nucleotide interconversion
GO:0071679: commissural neuron axon guidance
GO:0055009: atrial cardiac muscle tissue morphogenesis
GO:0055096: low-density lipoprotein particle mediated signaling
GO:0002689: negative regulation of leukocyte chemotaxis
GO:0010703: negative regulation of histolysis
GO:0006944: cellular membrane fusion
GO:0043620: regulation of transcription in response to stress
GO:0071676: negative regulation of mononuclear cell migration
GO:0046882: negative regulation of follicle-stimulating hormone secretion
GO:0016576: histone dephosphorylation
GO:0005152: interleukin-1 receptor antagonist activity
GO:0008260: 3-oxoacid CoA-transferase activity
GO:0048762: mesenchymal cell differentiation
GO:0048841: regulation of axon extension involved in axon guidance
GO:0010517: regulation of phospholipase activity
GO:0055106: ubiquitin-protein ligase regulator activity
GO:0009967: positive regulation of signal transduction
GO:0005251: delayed rectifier potassium channel activity
GO:0070830: tight junction assembly
GO:0005109: frizzled binding
GO:0070495: negative regulation of thrombin receptor signaling pathway
GO:0032740: positive regulation of interleukin-17 production
GO:0005522: profilin binding
GO:0045964: positive regulation of dopamine metabolic process
GO:0042805: actinin binding
GO:0042987: amyloid precursor protein catabolic process
GO:0050883: musculoskeletal movement, spinal reflex action
GO:0004334: fumarylacetoacetase activity
GO:0005007: fibroblast growth factor receptor activity
GO:0007243: intracellular protein kinase cascade
GO:0030282: bone mineralization
GO:0050432: catecholamine secretion
GO:0035067: negative regulation of histone acetylation
GO:0071287: cellular response to manganese ion
GO:0005657: replication fork
GO:0001554: luteolysis
GO:0030118: clathrin coat
GO:0031045: dense core granule
GO:0035413: positive regulation of catenin import into nucleus
GO:0006157: deoxyadenosine catabolic process
GO:0071396: cellular response to lipid
GO:0032584: growth cone membrane
GO:0050850: positive regulation of calcium-mediated signaling
GO:0010460: positive regulation of heart rate
GO:0043199: sulfate binding
GO:0010453: regulation of cell fate commitment
GO:0031267: small GTPase binding
GO:0007388: posterior compartment specification
GO:0006303: double-strand break repair via nonhomologous end joining
GO:0022612: gland morphogenesis
GO:0051798: positive regulation of hair follicle development
GO:0043537: negative regulation of blood vessel endothelial cell migration
GO:0001829: trophectodermal cell differentiation
GO:0032277: negative regulation of gonadotropin secretion
GO:0015651: quaternary ammonium group transmembrane transporter activity
GO:0005068: transmembrane receptor protein tyrosine kinase adaptor activity
GO:0015012: heparan sulfate proteoglycan biosynthetic process
GO:0051443: positive regulation of ubiquitin-protein ligase activity
GO:0042272: nuclear RNA export factor complex

GO:0048286: lung alveolus development
GO:0010633: negative regulation of epithelial cell migration
GO:0060601: lateral sprouting from an epithelium
GO:0002244: hemopoietic progenitor cell differentiation
GO:0060155: platelet dense granule organization
GO:0006171: cAMP biosynthetic process
GO:0015802: basic amino acid transport
GO:0019907: cyclin-dependent protein kinase activating kinase holoenzyme complex
GO:0048041: focal adhesion assembly
GO:0002088: lens development in camera-type eye
GO:0005662: DNA replication factor A complex
GO:0001780: neutrophil homeostasis
GO:0008045: motor axon guidance
GO:0050862: positive regulation of T cell receptor signaling pathway

Names	total	elements
#1043IR #1051IR #1063IR #1095IR #10IR	70	GO:0006810: transport
		GO:0005515: protein binding
		GO:0005789: endoplasmic reticulum membrane
		GO:0007264: small GTPase mediated signal transduction
		GO:0045202: synapse
		GO:0005730: nucleolus
		GO:0006813: potassium ion transport
		GO:0007420: brain development
		GO:0042803: protein homodimerization activity
		GO:0016787: hydrolase activity
		GO:0007584: response to nutrient
		GO:0008233: peptidase activity
		GO:0001503: ossification
		GO:0004872: receptor activity
		GO:0005216: ion channel activity
		GO:0006979: response to oxidative stress
		GO:0006897: endocytosis
		GO:0007268: synaptic transmission
		GO:0005622: intracellular
		GO:0016021: integral to membrane
		GO:0004252: serine-type endopeptidase activity
		GO:0008270: zinc ion binding
		GO:0017124: SH3 domain binding
		GO:0005525: GTP binding
		GO:0008239: dipeptidyl-peptidase activity
		GO:0008083: growth factor activity
		GO:0046872: metal ion binding
		GO:0005516: calmodulin binding
		GO:0005509: calcium ion binding
		GO:0019901: protein kinase binding
		GO:0006508: proteolysis
		GO:0007186: G-protein coupled receptor protein signaling pathway
		GO:0030054: cell junction
		GO:0006811: ion transport
		GO:0005215: transporter activity
		GO:0000287: magnesium ion binding
		GO:0045211: postsynaptic membrane
		GO:0007259: JAK-STAT cascade
		GO:0016740: transferase activity
		GO:0005576: extracellular region
		GO:0008150: biological_process
		GO:0004930: G-protein coupled receptor activity
		GO:0005886: plasma membrane
		GO:0003674: molecular_function
		GO:0030154: cell differentiation
		GO:0007267: cell-cell signaling
		GO:0008289: lipid binding
		GO:0005783: endoplasmic reticulum
		GO:0003924: GTPase activity
		GO:0009986: cell surface
		GO:0008152: metabolic process
		GO:0016020: membrane
		GO:0005615: extracellular space
		GO:0006814: sodium ion transport
		GO:0005624: membrane fraction
		GO:0007156: homophilic cell adhesion
		GO:0005524: ATP binding
		GO:0005737: cytoplasm
		GO:0000166: nucleotide binding
		GO:0007399: nervous system development
		GO:0006874: cellular calcium ion homeostasis
		GO:0005125: cytokine activity
		GO:0007165: signal transduction
		GO:0005634: nucleus
		GO:0007155: cell adhesion
		GO:0005739: mitochondrion
		GO:0005887: integral to plasma membrane
		GO:0005254: chloride channel activity
		GO:0043123: positive regulation of I-kappaB kinase/NF-kappaB cascade
		GO:0005794: Golgi apparatus
#1043IR #1051IR #1095IR #10IR	3	GO:0016820: hydrolase activity, acting on acid anhydrides, catalyzing transmembrane movement of substances
		GO:0045786: negative regulation of cell cycle
		GO:0006754: ATP biosynthetic process
#1043IR #1063IR #1095IR #10IR	22	GO:0042517: positive regulation of tyrosine phosphorylation of Stat3 protein
		GO:0001837: epithelial to mesenchymal transition
		GO:0007204: elevation of cytosolic calcium ion concentration
		GO:0016042: lipid catabolic process
		GO:0017046: peptide hormone binding
		GO:0042110: T cell activation
		GO:0050731: positive regulation of peptidyl-tyrosine phosphorylation
		GO:0005234: extracellular-glutamate-gated ion channel activity
		GO:0015277: kainate selective glutamate receptor activity
		GO:0008430: selenium binding
		GO:0006107: oxaloacetate metabolic process
		GO:0042802: identical protein binding
		GO:0000187: activation of MAPK activity
		GO:0019953: sexual reproduction
		GO:0008076: voltage-gated potassium channel complex
		GO:0001666: response to hypoxia
		GO:0070064: proline-rich region binding
		GO:0004177: aminopeptidase activity
		GO:0007588: excretion
		GO:0006631: fatty acid metabolic process
		GO:0005625: soluble fraction
		GO:0007389: pattern specification process
#1051IR #1063IR #1095IR #10IR	172	GO:0030674: protein binding, bridging
		GO:0006915: apoptosis
		GO:0007409: axonogenesis
		GO:0016481: negative regulation of transcription
		GO:0042310: vasoconstriction
		GO:0048246: macrophage chemotaxis
		GO:0050896: response to stimulus
		GO:0006355: regulation of transcription, DNA-dependent
		GO:0007601: visual perception
		GO:0031225: anchored to membrane
		GO:0006641: triglyceride metabolic process
		GO:0043565: sequence-specific DNA binding
		GO:0042632: cholesterol homeostasis
		GO:0003676: nucleic acid binding
		GO:0006461: protein complex assembly
		GO:0030539: male genitalia development
		GO:0007605: sensory perception of sound
		GO:0016323: basolateral plasma membrane
		GO:0042475: odontogenesis of dentine-containing tooth
		GO:0043234: protein complex
		GO:0008284: positive regulation of cell proliferation
		GO:0042734: presynaptic membrane
		GO:0006958: complement activation, classical pathway
		GO:0005200: structural constituent of cytoskeleton
		GO:0005161: platelet-derived growth factor receptor binding
		GO:0030173: integral to Golgi membrane
		GO:0042127: regulation of cell proliferation
		GO:0004528: phosphodiesterase I activity
		GO:0030659: cytoplasmic vesicle membrane
		GO:0016757: transferase activity, transferring glycosyl groups
		GO:0050839: cell adhesion molecule binding
		GO:0005518: collagen binding
		GO:0006812: cation transport
		GO:0040008: regulation of growth

GO:0006869: lipid transport
GO:0004867: serine-type endopeptidase inhibitor activity
GO:0008134: transcription factor binding
GO:0009611: response to wounding
GO:0015293: symporter activity
GO:0050656: 3'-phosphoadenosine 5'-phosphosulfate binding
GO:0008380: RNA splicing
GO:0016477: cell migration
GO:0006836: neurotransmitter transport
GO:0030346: protein phosphatase 2B binding
GO:0048306: calcium-dependent protein binding
GO:0008201: heparin binding
GO:0030324: lung development
GO:0005923: tight junction
GO:0005575: cellular_component
GO:0005813: centrosome
GO:0019838: growth factor binding
GO:0005085: guanyl-nucleotide exchange factor activity
GO:0016023: cytoplasmic membrane-bounded vesicle
GO:0016491: oxidoreductase activity
GO:0043193: positive regulation of gene-specific transcription
GO:0004714: transmembrane receptor protein tyrosine kinase activity
GO:0009055: electron carrier activity
GO:0005021: vascular endothelial growth factor receptor activity
GO:0004176: ATP-dependent peptidase activity
GO:0030505: inorganic diphosphate transport
GO:0048008: platelet-derived growth factor receptor signaling pathway
GO:0003779: actin binding
GO:0030027: lamellipodium
GO:0016324: apical plasma membrane
GO:0009653: anatomical structure morphogenesis
GO:0006816: calcium ion transport
GO:0007586: digestion
GO:0016942: insulin-like growth factor binding protein complex
GO:0045087: innate immune response
GO:0009968: negative regulation of signal transduction
GO:0008283: cell proliferation
GO:0006952: defense response
GO:0005604: basement membrane
GO:0016192: vesicle-mediated transport
GO:0007169: transmembrane receptor protein tyrosine kinase signaling pathway
GO:0016887: ATPase activity
GO:0000278: mitotic cell cycle
GO:0032869: cellular response to insulin stimulus
GO:0008015: blood circulation
GO:0045121: membrane raft
GO:0003677: DNA binding
GO:0007275: multicellular organismal development
GO:0005578: proteinaceous extracellular matrix
GO:0006886: intracellular protein transport
GO:0005829: cytosol
GO:0042594: response to starvation
GO:0030334: regulation of cell migration
GO:0009725: response to hormone stimulus
GO:0022900: electron transport chain
GO:0001570: vasculogenesis
GO:0016563: transcription activator activity
GO:0006334: nucleosome assembly
GO:0006357: regulation of transcription from RNA polymerase II promoter
GO:0031994: insulin-like growth factor I binding
GO:0005488: binding
GO:0031410: cytoplasmic vesicle
GO:0005230: extracellular ligand-gated ion channel activity
GO:0001558: regulation of cell growth
GO:0007194: negative regulation of adenylate cyclase activity
GO:0005769: early endosome
GO:0001755: neural crest cell migration
GO:0048662: negative regulation of smooth muscle cell proliferation
GO:0000122: negative regulation of transcription from RNA polymerase II promoter
GO:0050501: hyaluronan synthase activity
GO:0007179: transforming growth factor beta receptor signaling pathway
GO:0019717: synaptosome
GO:0008285: negative regulation of cell proliferation
GO:0012505: endomembrane system
GO:0045449: regulation of transcription
GO:0051020: GTPase binding
GO:0008219: cell death
GO:0000139: Golgi membrane
GO:0030521: androgen receptor signaling pathway
GO:0006629: lipid metabolic process
GO:0007417: central nervous system development
GO:0042981: regulation of apoptosis
GO:0004674: protein serine/threonine kinase activity
GO:0014912: negative regulation of smooth muscle cell migration
GO:0030198: extracellular matrix organization
GO:0031093: platelet alpha granule lumen
GO:0006916: anti-apoptosis
GO:0016337: cell-cell adhesion
GO:0006457: protein folding
GO:0005506: iron ion binding
GO:0030500: regulation of bone mineralization
GO:0030162: regulation of proteolysis
GO:0007229: integrin-mediated signaling pathway
GO:0005057: receptor signaling protein activity
GO:0005856: cytoskeleton
GO:0030335: positive regulation of cell migration
GO:0007528: neuromuscular junction development
GO:0030199: collagen fibril organization
GO:0003774: motor activity
GO:0003824: catalytic activity
GO:0004222: metalloendopeptidase activity
GO:0007200: activation of phospholipase C activity by G-protein coupled receptor protein signaling pathway coupled to IP3 second messenger
GO:0005540: hyaluronic acid binding
GO:0006796: phosphate metabolic process
GO:0008168: methyltransferase activity
GO:0006826: iron ion transport
GO:0019722: calcium-mediated signaling
GO:0001726: ruffle
GO:0004871: signal transducer activity
GO:0005882: intermediate filament
GO:0030426: growth cone
GO:0042391: regulation of membrane potential
GO:0005529: sugar binding
GO:0015031: protein transport
GO:0045944: positive regulation of transcription from RNA polymerase II promoter
GO:0007507: heart development
GO:0005244: voltage-gated ion channel activity
GO:0003723: RNA binding
GO:0006955: immune response
GO:0004551: nucleotide diphosphatase activity
GO:0008217: regulation of blood pressure
GO:0030528: transcription regulator activity
GO:0005096: GTPase activator activity
GO:0005158: insulin receptor binding
GO:0006954: inflammatory response
GO:0009887: organ morphogenesis
GO:0008378: galactosyltransferase activity
GO:0030308: negative regulation of cell growth
GO:0005201: extracellular matrix structural constituent
GO:0007171: activation of transmembrane receptor protein tyrosine kinase activity

		GO:0005911: cell-cell junction
		GO:0010008: endosome membrane
		GO:0048471: perinuclear region of cytoplasm
		GO:0046982: protein heterodimerization activity
		GO:0005874: microtubule
		GO:0005507: copper ion binding
		GO:0045599: negative regulation of fat cell differentiation
		GO:0006091: generation of precursor metabolites and energy
#1043IR #1051IR #1063IR #10IR	1	GO:0030165: PDZ domain binding
#1043IR #1051IR #1063IR #1095IR	4	GO:0007626: locomotory behavior
		GO:0005249: voltage-gated potassium channel activity
		GO:0004896: cytokine receptor activity
		GO:0005179: hormone activity
#1043IR #1095IR #10IR	6	GO:0005391: sodium:potassium-exchanging ATPase activity
		GO:0019861: flagellum
		GO:0051216: cartilage development
		GO:0005929: cilium
		GO:0019695: choline metabolic process
		GO:0040007: growth
#1051IR #1095IR #10IR	27	GO:0046627: negative regulation of insulin receptor signaling pathway
		GO:0014826: vein smooth muscle contraction
		GO:0003707: steroid hormone receptor activity
		GO:0045719: negative regulation of glycogen biosynthetic process
		GO:0047429: nucleoside-triphosphate diphosphatase activity
		GO:0043034: costamere
		GO:0045722: positive regulation of gluconeogenesis
		GO:0050427: 3'-phosphoadenosine 5'-phosphosulfate metabolic process
		GO:0008307: structural constituent of muscle
		GO:0009143: nucleoside triphosphate catabolic process
		GO:0050909: sensory perception of taste
		GO:0051082: unfolded protein binding
		GO:0030730: sequestering of triglyceride
		GO:0001600: endothelin-B receptor activity
		GO:0009187: cyclic nucleotide metabolic process
		GO:0006885: regulation of pH
		GO:0006397: mRNA processing
		GO:0006367: transcription initiation from RNA polymerase II promoter
		GO:0030643: cellular phosphate ion homeostasis
		GO:0050681: androgen receptor binding
		GO:0030374: ligand-dependent nuclear receptor transcription coactivator activity
		GO:0007187: G-protein signaling, coupled to cyclic nucleotide second messenger
		GO:0000086: G2/M transition of mitotic cell cycle
		GO:0007368: determination of left/right symmetry
		GO:0030145: manganese ion binding
		GO:0016455: RNA polymerase II transcription mediator activity
		GO:0046325: negative regulation of glucose import
#1063IR #1095IR #10IR	369	GO:0048705: skeletal system morphogenesis
		GO:0043184: vascular endothelial growth factor receptor 2 binding
		GO:0045787: positive regulation of cell cycle
		GO:0032587: ruffle membrane
		GO:0001942: hair follicle development
		GO:0001540: beta-amyloid binding
		GO:0005770: late endosome
		GO:0043536: positive regulation of blood vessel endothelial cell migration
		GO:0006950: response to stress
		GO:0043204: perikaryon
		GO:0044419: interspecies interaction between organisms
		GO:0004888: transmembrane receptor activity
		GO:0019221: cytokine-mediated signaling pathway
		GO:0051092: positive regulation of NF-kappaB transcription factor activity
		GO:0032355: response to estradiol stimulus
		GO:0001764: neuron migration
		GO:0005159: insulin-like growth factor receptor binding
		GO:0051291: protein heterooligomerization
		GO:0043627: response to estrogen stimulus
		GO:0042626: ATPase activity, coupled to transmembrane movement of substances
		GO:0042169: SH2 domain binding
		GO:0032874: positive regulation of stress-activated MAPK cascade
		GO:0016032: viral reproduction
		GO:0000902: cell morphogenesis
		GO:0046983: protein dimerization activity
		GO:0008624: induction of apoptosis by extracellular signals
		GO:0005922: connexon complex
		GO:0006688: glycosphingolipid biosynthetic process
		GO:0031902: late endosome membrane
		GO:0005759: mitochondrial matrix
		GO:0008236: serine-type peptidase activity
		GO:0004197: cysteine-type endopeptidase activity
		GO:0043231: intracellular membrane-bounded organelle
		GO:0006911: phagocytosis, engulfment
		GO:0008009: chemokine activity
		GO:0008415: acyltransferase activity
		GO:0005778: peroxisomal membrane
		GO:0006805: xenobiotic metabolic process
		GO:0030879: mammary gland development
		GO:0005952: cAMP-dependent protein kinase complex
		GO:0030425: dendrite
		GO:0043120: tumor necrosis factor binding
		GO:0006695: cholesterol biosynthetic process
		GO:0042995: cell projection
		GO:0008603: cAMP-dependent protein kinase regulator activity
		GO:0016607: nuclear speck
		GO:0008147: structural constituent of bone
		GO:0048741: skeletal muscle fiber development
		GO:0001937: negative regulation of endothelial cell proliferation
		GO:0016702: oxidoreductase activity, acting on single donors with incorporation of molecular oxygen, incorporation of two atoms of oxygen
		GO:0007411: axon guidance
		GO:0005543: phospholipid binding
		GO:0007173: epidermal growth factor receptor signaling pathway
		GO:0015333: peptide:hydrogen symporter activity
		GO:0043434: response to peptide hormone stimulus
		GO:0006112: energy reserve metabolic process
		GO:0050905: neuromuscular process
		GO:0000776: kinetochore
		GO:0003009: skeletal muscle contraction
		GO:0042476: odontogenesis
		GO:0008277: regulation of G-protein coupled receptor protein signaling pathway
		GO:0031557: induction of programmed cell death in response to chemical stimulus
		GO:0043197: dendritic spine
		GO:0043542: endothelial cell migration
		GO:0044262: cellular carbohydrate metabolic process
		GO:0042755: eating behavior
		GO:0009617: response to bacterium
		GO:0004842: ubiquitin-protein ligase activity
		GO:0046330: positive regulation of JNK cascade
		GO:0007435: salivary gland morphogenesis
		GO:0030307: positive regulation of cell growth
		GO:0048011: nerve growth factor receptor signaling pathway
		GO:0006919: activation of caspase activity
		GO:0001889: liver development
		GO:0031290: retinal ganglion cell axon guidance
		GO:0050900: leukocyte migration
		GO:0004623: phospholipase A2 activity
		GO:0008017: microtubule binding
		GO:0006511: ubiquitin-dependent protein catabolic process
		GO:0042493: response to drug
		GO:0007413: axonal fasciculation
		GO:0001541: ovarian follicle development
		GO:0006605: protein targeting
		GO:0001654: eye development

GO:0019903: protein phosphatase binding
GO:0005768: endosome
GO:0043524: negative regulation of neuron apoptosis
GO:0050806: positive regulation of synaptic transmission
GO:0004568: chitinase activity
GO:0031069: hair follicle morphogenesis
GO:0019228: regulation of action potential in neuron
GO:0008305: integrin complex
GO:0004672: protein kinase activity
GO:0004725: protein tyrosine phosphatase activity
GO:0030036: actin cytoskeleton organization
GO:0008286: insulin receptor signaling pathway
GO:0007190: activation of adenylate cyclase activity
GO:0031623: receptor internalization
GO:0005771: multivesicular body
GO:0005160: transforming growth factor beta receptor binding
GO:0016050: vesicle organization
GO:0001968: fibronectin binding
GO:0051726: regulation of cell cycle
GO:0048168: regulation of neuronal synaptic plasticity
GO:0042325: regulation of phosphorylation
GO:0015175: neutral amino acid transmembrane transporter activity
GO:0005089: Rho guanyl-nucleotide exchange factor activity
GO:0005758: mitochondrial intermembrane space
GO:0015297: antiporter activity
GO:0030900: forebrain development
GO:0045892: negative regulation of transcription, DNA-dependent
GO:0051966: regulation of synaptic transmission, glutamatergic
GO:0016504: peptidase activator activity
GO:0035094: response to nicotine
GO:0016264: gap junction assembly
GO:0016500: protein-hormone receptor activity
GO:0006935: chemotaxis
GO:0043525: positive regulation of neuron apoptosis
GO:0006633: fatty acid biosynthetic process
GO:0051384: response to glucocorticoid stimulus
GO:0007218: neuropeptide signaling pathway
GO:0006643: membrane lipid metabolic process
GO:0003702: RNA polymerase II transcription factor activity
GO:0006663: platelet activating factor biosynthetic process
GO:0042605: peptide antigen binding
GO:0006887: exocytosis
GO:0010718: positive regulation of epithelial to mesenchymal transition
GO:0004497: monooxygenase activity
GO:0015629: actin cytoskeleton
GO:0045726: positive regulation of integrin biosynthetic process
GO:0006220: pyrimidine nucleotide metabolic process
GO:0030326: embryonic limb morphogenesis
GO:0047555: 3',5'-cyclic-GMP phosphodiesterase activity
GO:0030170: pyridoxal phosphate binding
GO:0031418: L-ascorbic acid binding
GO:0006809: nitric oxide biosynthetic process
GO:0001502: cartilage condensation
GO:0008092: cytoskeletal protein binding
GO:0006974: response to DNA damage stimulus
GO:0060271: cilium morphogenesis
GO:0050778: positive regulation of immune response
GO:0019959: interleukin-8 binding
GO:0042472: inner ear morphogenesis
GO:0006892: post-Golgi vesicle-mediated transport
GO:0007596: blood coagulation
GO:0031295: T cell costimulation
GO:0031966: mitochondrial membrane
GO:0010693: negative regulation of alkaline phosphatase activity
GO:0019966: interleukin-1 binding
GO:0009409: response to cold
GO:0006260: DNA replication
GO:0005102: receptor binding
GO:0050777: negative regulation of immune response
GO:0016209: antioxidant activity
GO:0042542: response to hydrogen peroxide
GO:0060389: pathway-restricted SMAD protein phosphorylation
GO:0015334: high affinity oligopeptide transporter activity
GO:0043065: positive regulation of apoptosis
GO:0008022: protein C-terminus binding
GO:0051091: positive regulation of transcription factor activity
GO:0043169: cation binding
GO:0005868: cytoplasmic dynein complex
GO:0019216: regulation of lipid metabolic process
GO:0007214: gamma-aminobutyric acid signaling pathway
GO:0030315: T-tubule
GO:0007215: glutamate signaling pathway
GO:0030182: neuron differentiation
GO:0051891: positive regulation of cardioblast differentiation
GO:0000075: cell cycle checkpoint
GO:0007283: spermatogenesis
GO:0005802: trans-Golgi network
GO:0004089: carbonate dehydratase activity
GO:0005792: microsome
GO:0045941: positive regulation of transcription
GO:0022601: menstrual cycle phase
GO:0051018: protein kinase A binding
GO:0016829: lyase activity
GO:0007018: microtubule-based movement
GO:0045177: apical part of cell
GO:0005654: nucleoplasm
GO:0043531: ADP binding
GO:0001938: positive regulation of endothelial cell proliferation
GO:0019430: removal of superoxide radicals
GO:0048678: response to axon injury
GO:0005520: insulin-like growth factor binding
GO:0010575: positive regulation vascular endothelial growth factor production
GO:0048663: neuron fate commitment
GO:0019233: sensory perception of pain
GO:0043066: negative regulation of apoptosis
GO:0009615: response to virus
GO:0005916: fascia adherens
GO:0004437: inositol or phosphatidylinositol phosphatase activity
GO:0051258: protein polymerization
GO:0006879: cellular iron ion homeostasis
GO:0005003: ephrin receptor activity
GO:0001501: skeletal system development
GO:0016798: hydrolase activity, acting on glycosyl bonds
GO:0048103: somatic stem cell division
GO:0043499: eukaryotic cell surface binding
GO:0008146: sulfotransferase activity
GO:0007585: respiratory gaseous exchange
GO:0016568: chromatin modification
GO:0045296: cadherin binding
GO:0006032: chitin catabolic process
GO:0009636: response to toxin
GO:0051402: neuron apoptosis
GO:0007623: circadian rhythm
GO:0015893: drug transport
GO:0016788: hydrolase activity, acting on ester bonds
GO:0005777: peroxisome
GO:0009966: regulation of signal transduction
GO:0008021: synaptic vesicle
GO:0045669: positive regulation of osteoblast differentiation
GO:0048172: regulation of short-term neuronal synaptic plasticity

GO:0043195: terminal button
GO:0016363: nuclear matrix
GO:0030552: cAMP binding
GO:0005925: focal adhesion
GO:0006936: muscle contraction
GO:0005938: cell cortex
GO:0004965: GABA-B receptor activity
GO:0043027: caspase inhibitor activity
GO:0003713: transcription coactivator activity
GO:0032570: response to progesterone stimulus
GO:0031012: extracellular matrix
GO:0051015: actin filament binding
GO:0051260: protein homooligomerization
GO:0048545: response to steroid hormone stimulus
GO:0005743: mitochondrial inner membrane
GO:0051084: 'de novo' posttranslational protein folding
GO:0009116: nucleoside metabolic process
GO:0003828: alpha-N-acetylneuraminate alpha-2,8-sialyltransferase activity
GO:0050729: positive regulation of inflammatory response
GO:0043154: negative regulation of caspase activity
GO:0005765: lysosomal membrane
GO:0045893: positive regulation of transcription, DNA-dependent
GO:0051592: response to calcium ion
GO:0006006: glucose metabolic process
GO:0030018: Z disc
GO:0030971: receptor tyrosine kinase binding
GO:0045732: positive regulation of protein catabolic process
GO:0005381: iron ion transmembrane transporter activity
GO:0003682: chromatin binding
GO:0008104: protein localization
GO:0007067: mitosis
GO:0030097: hemopoiesis
GO:0008144: drug binding
GO:0005114: type II transforming growth factor beta receptor binding
GO:0017111: nucleoside-triphosphatase activity
GO:0033630: positive regulation of cell adhesion mediated by integrin
GO:0004143: diacylglycerol kinase activity
GO:0006464: protein modification process
GO:0031965: nuclear membrane
GO:0016922: ligand-dependent nuclear receptor binding
GO:0004046: aminoacylase activity
GO:0051056: regulation of small GTPase mediated signal transduction
GO:0015467: G-protein activated inward rectifier potassium channel activity
GO:0015075: ion transmembrane transporter activity
GO:0006469: negative regulation of protein kinase activity
GO:0016874: ligase activity
GO:0001947: heart looping
GO:0045454: cell redox homeostasis
GO:0010634: positive regulation of epithelial cell migration
GO:0005080: protein kinase C binding
GO:0030111: regulation of Wnt receptor signaling pathway
GO:0034097: response to cytokine stimulus
GO:0007010: cytoskeleton organization
GO:0000785: chromatin
GO:0050680: negative regulation of epithelial cell proliferation
GO:0001525: angiogenesis
GO:0007265: Ras protein signal transduction
GO:0043005: neuron projection
GO:0009395: phospholipid catabolic process
GO:0042416: dopamine biosynthetic process
GO:0005905: coated pit
GO:0030424: axon
GO:0009749: response to glucose stimulus
GO:0003714: transcription corepressor activity
GO:0006396: RNA processing
GO:0006749: glutathione metabolic process
GO:0060317: cardiac epithelial to mesenchymal transition
GO:0042470: melanosome
GO:0008028: monocarboxylic acid transmembrane transporter activity
GO:0045778: positive regulation of ossification
GO:0006096: glycolysis
GO:0048666: neuron development
GO:0004702: receptor signaling protein serine/threonine kinase activity
GO:0043687: post-translational protein modification
GO:0007160: cell-matrix adhesion
GO:0008237: metallopeptidase activity
GO:0031016: pancreas development
GO:0060038: cardiac muscle cell proliferation
GO:0048754: branching morphogenesis of a tube
GO:0000165: MAPKKK cascade
GO:0010038: response to metal ion
GO:0050796: regulation of insulin secretion
GO:0005667: transcription factor complex
GO:0040014: regulation of multicellular organism growth
GO:0030593: neutrophil chemotaxis
GO:0048813: dendrite morphogenesis
GO:0005198: structural molecule activity
GO:0030496: midbody
GO:0043043: peptide biosynthetic process
GO:0020037: heme binding
GO:0007049: cell cycle
GO:0005694: chromosome
GO:0000079: regulation of cyclin-dependent protein kinase activity
GO:0005544: calcium-dependent phospholipid binding
GO:0008016: regulation of heart contraction
GO:0005635: nuclear envelope
GO:0032909: regulation of transforming growth factor-beta2 production
GO:0006281: DNA repair
GO:0060021: palate development
GO:0035023: regulation of Rho protein signal transduction
GO:0019899: enzyme binding
GO:0032147: activation of protein kinase activity
GO:0015804: neutral amino acid transport
GO:0000084: S phase of mitotic cell cycle
GO:0042060: wound healing
GO:0007154: cell communication
GO:0004859: phospholipase inhibitor activity
GO:0019904: protein domain specific binding
GO:0009897: external side of plasma membrane
GO:0006909: phagocytosis
GO:0030139: endocytic vesicle
GO:0051301: cell division
GO:0007050: cell cycle arrest
GO:0005741: mitochondrial outer membrane
GO:0051219: phosphoprotein binding
GO:0005764: lysosome
GO:0031901: early endosome membrane
GO:0043200: response to amino acid stimulus
GO:0050770: regulation of axonogenesis
GO:0046320: regulation of fatty acid oxidation
GO:0016567: protein ubiquitination
GO:0045766: positive regulation of angiogenesis
GO:0006366: transcription from RNA polymerase II promoter
GO:0048469: cell maturation
GO:0008373: sialyltransferase activity
GO:0022857: transmembrane transporter activity
GO:0008203: cholesterol metabolic process
GO:0008509: anion transmembrane transporter activity
GO:0030949: positive regulation of vascular endothelial growth factor receptor signaling pathway
GO:0030168: platelet activation

		GO:0008656: caspase activator activity
		GO:0044267: cellular protein metabolic process
		GO:0045471: response to ethanol
		GO:0009612: response to mechanical stimulus
		GO:0009408: response to heat
		GO:0030100: regulation of endocytosis
		GO:0016564: transcription repressor activity
		GO:0045823: positive regulation of heart contraction
		GO:0007040: lysosome organization
		GO:0006139: nucleobase, nucleoside, nucleotide and nucleic acid metabolic process
		GO:0030890: positive regulation of B cell proliferation
		GO:0030660: Golgi-associated vesicle membrane
		GO:0016459: myosin complex
		GO:0005975: carbohydrate metabolic process
		GO:0016055: Wnt receptor signaling pathway
		GO:0008347: glial cell migration
		GO:0019825: oxygen binding
		GO:0000777: condensed chromosome kinetochore
		GO:0004857: enzyme inhibitor activity
		GO:0048699: generation of neurons
		GO:0051795: positive regulation of catagen
		GO:0042593: glucose homeostasis
		GO:0000082: G1/S transition of mitotic cell cycle
		GO:0007205: activation of protein kinase C activity by G-protein coupled receptor protein signaling pathway
		GO:0051899: membrane depolarization
#1043IR #1063IR #10IR	21	GO:0070195: growth hormone receptor complex
		GO:0046449: creatinine metabolic process
		GO:0046581: intercellular canaliculus
		GO:0044236: multicellular organismal metabolic process
		GO:0006105: succinate metabolic process
		GO:0032870: cellular response to hormone stimulus
		GO:0006101: citrate metabolic process
		GO:0006103: 2-oxoglutarate metabolic process
		GO:0006573: valine metabolic process
		GO:0060396: growth hormone receptor signaling pathway
		GO:0045184: establishment of protein localization
		GO:0006600: creatine metabolic process
		GO:0000255: allantoin metabolic process
		GO:0042977: activation of JAK2 kinase activity
		GO:0019530: taurine metabolic process
		GO:0033632: regulation of cell-cell adhesion mediated by integrin
		GO:0006549: isoleucine metabolic process
		GO:0007202: activation of phospholipase C activity
		GO:0040018: positive regulation of multicellular organism growth
		GO:0048009: insulin-like growth factor receptor signaling pathway
		GO:0042523: positive regulation of tyrosine phosphorylation of Stat5 protein
#1051IR #1063IR #10IR	26	GO:0005319: lipid transporter activity
		GO:0004889: nicotinic acetylcholine-activated cation-selective channel activity
		GO:0050821: protein stabilization
		GO:0008013: beta-catenin binding
		GO:0006940: regulation of smooth muscle contraction
		GO:0005892: nicotinic acetylcholine-gated receptor-channel complex
		GO:0019898: extrinsic to membrane
		GO:0030178: negative regulation of Wnt receptor signaling pathway
		GO:0034394: protein localization at cell surface
		GO:0005178: integrin binding
		GO:0030863: cortical cytoskeleton
		GO:0015171: amino acid transmembrane transporter activity
		GO:0015464: acetylcholine receptor activity
		GO:0045597: positive regulation of cell differentiation
		GO:0005912: adherens junction
		GO:0006865: amino acid transport
		GO:0006953: acute-phase response
		GO:0003730: mRNA 3'-UTR binding
		GO:0030336: negative regulation of cell migration
		GO:0005581: collagen
		GO:0016339: calcium-dependent cell-cell adhesion
		GO:0060079: regulation of excitatory postsynaptic membrane potential
		GO:0007611: learning or memory
		GO:0007005: mitochondrion organization
		GO:0009312: oligosaccharide biosynthetic process
		GO:0042157: lipoprotein metabolic process
#1043IR #1051IR #1095IR	5	GO:0031402: sodium ion binding
		GO:0007242: intracellular signaling cascade
		GO:0030955: potassium ion binding
		GO:0031404: chloride ion binding
		GO:0007166: cell surface receptor linked signal transduction
#1043IR #1063IR #1095IR	3	GO:0043130: ubiquitin binding
		GO:0008195: phosphatidate phosphatase activity
		GO:0009791: post-embryonic development
#1051IR #1063IR #1095IR	31	GO:0007219: Notch signaling pathway
		GO:0006917: induction of apoptosis
		GO:0001775: cell activation
		GO:0016310: phosphorylation
		GO:0043569: negative regulation of insulin-like growth factor receptor signaling pathway
		GO:0018108: peptidyl-tyrosine phosphorylation
		GO:0008499: UDP-galactose:beta-N-acetylglucosamine beta-1,3-galactosyltransferase activity
		GO:0017166: vinculin binding
		GO:0006559: L-phenylalanine catabolic process
		GO:0045028: purinergic nucleotide receptor activity, G-protein coupled
		GO:0045165: cell fate commitment
		GO:0007271: synaptic transmission, cholinergic
		GO:0005875: microtubule associated complex
		GO:0005793: ER-Golgi intermediate compartment
		GO:0048407: platelet-derived growth factor binding
		GO:0005891: voltage-gated calcium channel complex
		GO:0030318: melanocyte differentiation
		GO:0001542: ovulation from ovarian follicle
		GO:0007422: peripheral nervous system development
		GO:0000155: two-component sensor activity
		GO:0045740: positive regulation of DNA replication
		GO:0008360: regulation of cell shape
		GO:0016301: kinase activity
		GO:0005018: platelet-derived growth factor alpha-receptor activity
		GO:0017148: negative regulation of translation
		GO:0004890: GABA-A receptor activity
		GO:0048146: positive regulation of fibroblast proliferation
		GO:0005328: neurotransmitter:sodium symporter activity
		GO:0005245: voltage-gated calcium channel activity
		GO:0006572: tyrosine catabolic process
		GO:0015269: calcium-activated potassium channel activity
#1095IR #10IR	145	GO:0031227: intrinsic to endoplasmic reticulum membrane
		GO:0006656: phosphatidylcholine biosynthetic process
		GO:0015078: hydrogen ion transmembrane transporter activity
		GO:0048538: thymus development
		GO:0045860: positive regulation of protein kinase activity
		GO:0003712: transcription cofactor activity
		GO:0006108: malate metabolic process
		GO:0030330: DNA damage response, signal transduction by p53 class mediator
		GO:0004540: ribonuclease activity
		GO:0006094: gluconeogenesis
		GO:0008301: DNA bending activity
		GO:0019001: guanyl nucleotide binding
		GO:0009650: UV protection
		GO:0005388: calcium-transporting ATPase activity
		GO:0007252: I-kappaB phosphorylation
		GO:0004004: ATP-dependent RNA helicase activity
		GO:0007188: G-protein signaling, coupled to cAMP nucleotide second messenger
		GO:0032963: collagen metabolic process
		GO:0004386: helicase activity

		GO:0030101: natural killer cell activation
		GO:0045859: regulation of protein kinase activity
		GO:0030176: integral to endoplasmic reticulum membrane
		GO:0004322: ferroxidase activity
		GO:0042612: MHC class I protein complex
		GO:0019439: aromatic compound catabolic process
		GO:0030553: cGMP binding
		GO:0007015: actin filament organization
		GO:0006222: UMP biosynthetic process
		GO:0004364: glutathione transferase activity
		GO:0017153: sodium:dicarboxylate symporter activity
		GO:0016070: RNA metabolic process
		GO:0002474: antigen processing and presentation of peptide antigen via MHC class I
		GO:0005834: heterotrimeric G-protein complex
		GO:0008544: epidermis development
		GO:0015992: proton transport
		GO:0008320: protein transmembrane transporter activity
		GO:0000186: activation of MAPKK activity
		GO:0006835: dicarboxylic acid transport
		GO:0005097: Rab GTPase activator activity
		GO:0042645: mitochondrial nucleoid
		GO:0032313: regulation of Rab GTPase activity
		GO:0016461: unconventional myosin complex
		GO:0030867: rough endoplasmic reticulum membrane
		GO:0005641: nuclear envelope lumen
		GO:0006164: purine nucleotide biosynthetic process
		GO:0048873: homeostasis of number of cells within a tissue
		GO:0001935: endothelial cell proliferation
		GO:0030216: keratinocyte differentiation
		GO:0045088: regulation of innate immune response
		GO:0000502: proteasome complex
		GO:0006509: membrane protein ectodomain proteolysis
		GO:0031941: filamentous actin
		GO:0000303: response to superoxide
		GO:0004104: cholinesterase activity
		GO:0043457: regulation of cellular respiration
		GO:0007565: female pregnancy
		GO:0016779: nucleotidyltransferase activity
		GO:0031424: keratinization
		GO:0060001: minus-end directed microfilament motor activity
		GO:0007405: neuroblast proliferation
		GO:0006210: thymine catabolic process
		GO:0015701: bicarbonate transport
		GO:0006212: uracil catabolic process
		GO:0006825: copper ion transport
		GO:0014850: response to muscle activity
		GO:0005070: SH3/SH2 adaptor activity
		GO:0015718: monocarboxylic acid transport
		GO:0004180: carboxypeptidase activity
		GO:0005452: inorganic anion exchanger activity
		GO:0048477: oogenesis
		GO:0014003: oligodendrocyte development
		GO:0043085: positive regulation of catalytic activity
		GO:0046920: alpha(1,3)-fucosyltransferase activity
		GO:0005247: voltage-gated chloride channel activity
		GO:0006986: response to unfolded protein
		GO:0019370: leukotriene biosynthetic process
		GO:0006214: thymidine catabolic process
		GO:0042254: ribosome biogenesis
		GO:0031638: zymogen activation
		GO:0045334: clathrin-coated endocytic vesicle
		GO:0003777: microtubule motor activity
		GO:0016881: acid-amino acid ligase activity
		GO:0006417: regulation of translation
		GO:0005788: endoplasmic reticulum lumen
		GO:0010181: FMN binding
		GO:0006821: chloride transport
		GO:0004385: guanylate kinase activity
		GO:0016747: transferase activity, transferring acyl groups other than amino-acyl groups
		GO:0046870: cadmium ion binding
		GO:0008199: ferric iron binding
		GO:0016493: C-C chemokine receptor activity
		GO:0015813: L-glutamate transport
		GO:0005504: fatty acid binding
		GO:0051539: 4 iron, 4 sulfur cluster binding
		GO:0004181: metallocarboxypeptidase activity
		GO:0032722: positive regulation of chemokine production
		GO:0006635: fatty acid beta-oxidation
		GO:0051046: regulation of secretion
		GO:0060087: relaxation of vascular smooth muscle
		GO:0046688: response to copper ion
		GO:0043588: skin development
		GO:0046873: metal ion transmembrane transporter activity
		GO:0008026: ATP-dependent helicase activity
		GO:0007341: penetration of zona pellucida
		GO:0032393: MHC class I receptor activity
		GO:0004866: endopeptidase inhibitor activity
		GO:0006207: 'de novo' pyrimidine base biosynthetic process
		GO:0009607: response to biotic stimulus
		GO:0006145: purine base catabolic process
		GO:0060173: limb development
		GO:0007254: JNK cascade
		GO:0006875: cellular metal ion homeostasis
		GO:0007612: learning
		GO:0007126: meiosis
		GO:0042355: L-fucose catabolic process
		GO:0004693: cyclin-dependent protein kinase activity
		GO:0006099: tricarboxylic acid cycle
		GO:0030048: actin filament-based movement
		GO:0030520: estrogen receptor signaling pathway
		GO:0016591: DNA-directed RNA polymerase II, holoenzyme
		GO:0017113: dihydropyrimidine dehydrogenase (NADP+) activity
		GO:0007598: blood coagulation, extrinsic pathway
		GO:0045919: positive regulation of cytolysis
		GO:0050783: cocaine metabolic process
		GO:0006880: intracellular sequestering of iron ion
		GO:0002526: acute inflammatory response
		GO:0035035: histone acetyltransferase binding
		GO:0048468: cell development
		GO:0000302: response to reactive oxygen species
		GO:0051607: defense response to virus
		GO:0007286: spermatid development
		GO:0004185: serine-type carboxypeptidase activity
		GO:0005375: copper ion transmembrane transporter activity
		GO:0042102: positive regulation of T cell proliferation
		GO:0015501: glutamate:sodium symporter activity
		GO:0001533: cornified envelope
		GO:0006541: glutamine metabolic process
		GO:0003785: actin monomer binding
		GO:0008510: sodium:bicarbonate symporter activity
		GO:0031145: anaphase-promoting complex-dependent proteasomal ubiquitin-dependent protein catabolic process
		GO:0003945: N-acetyllactosamine synthase activity
		GO:0019882: antigen processing and presentation
		GO:0008043: intracellular ferritin complex
		GO:0006783: heme biosynthetic process
		GO:0004158: dihydroorotate oxidase activity
#1043IR #10IR	2	GO:0043410: positive regulation of MAPKKK cascade
		GO:0042301: phosphate binding
#1051IR #10IR	15	GO:0008527: taste receptor activity
		GO:0001678: cellular glucose homeostasis

GO:0035066: positive regulation of histone acetylation
GO:0046321: positive regulation of fatty acid oxidation
GO:0045333: cellular respiration
GO:0050873: brown fat cell differentiation
GO:0005665: DNA-directed RNA polymerase II, core complex
GO:0001659: temperature homeostasis
GO:0005001: transmembrane receptor protein tyrosine phosphatase activity
GO:0022904: respiratory electron transport chain
GO:0004114: 3',5'-cyclic-nucleotide phosphodiesterase activity
GO:0045295: gamma-catenin binding
GO:0004949: cannabinoid receptor activity
GO:0007610: behavior
GO:0018149: peptide cross-linking
#1063IR #10IR 506 GO:0048812: neuron projection morphogenesis
GO:0070374: positive regulation of ERK1 and ERK2 cascade
GO:0010759: positive regulation of macrophage chemotaxis
GO:0060070: canonical Wnt receptor signaling pathway
GO:0032094: response to food
GO:0030247: polysaccharide binding
GO:0018279: protein N-linked glycosylation via asparagine
GO:0019807: aspartoacylase activity
GO:0006939: smooth muscle contraction
GO:0008398: sterol 14-demethylase activity
GO:0030672: synaptic vesicle membrane
GO:0019432: triglyceride biosynthetic process
GO:0004903: growth hormone receptor activity
GO:0002634: regulation of germinal center formation
GO:0006928: cellular component movement
GO:0006906: vesicle fusion
GO:0060325: face morphogenesis
GO:0048539: bone marrow development
GO:0006325: chromatin organization
GO:0017016: Ras GTPase binding
GO:0035307: positive regulation of protein dephosphorylation
GO:0008198: ferrous iron binding
GO:0043101: purine-containing compound salvage
GO:0042578: phosphoric ester hydrolase activity
GO:0010553: negative regulation of gene-specific transcription from RNA polymerase II promoter
GO:0071062: alphav-beta3 integrin-vitronectin complex
GO:0005980: glycogen catabolic process
GO:0007568: aging
GO:0004833: tryptophan 2,3-dioxygenase activity
GO:0001656: metanephros development
GO:0021506: anterior neuropore closure
GO:0050479: glyceryl-ether monooxygenase activity
GO:0006569: tryptophan catabolic process
GO:0050998: nitric-oxide synthase binding
GO:0048514: blood vessel morphogenesis
GO:0007517: muscle organ development
GO:0050768: negative regulation of neurogenesis
GO:0005901: caveola
GO:0022409: positive regulation of cell-cell adhesion
GO:0070836: caveola assembly
GO:0002051: osteoblast fate commitment
GO:0043966: histone H3 acetylation
GO:0030177: positive regulation of Wnt receptor signaling pathway
GO:0043403: skeletal muscle tissue regeneration
GO:0070172: positive regulation of tooth mineralization
GO:0002709: regulation of T cell mediated immunity
GO:0002020: protease binding
GO:0017154: semaphorin receptor activity
GO:0010843: promoter binding
GO:0004459: L-lactate dehydrogenase activity
GO:0021766: hippocampus development
GO:0004445: inositol-polyphosphate 5-phosphatase activity
GO:0080025: phosphatidylinositol-3,5-bisphosphate binding
GO:0017157: regulation of exocytosis
GO:0006776: vitamin A metabolic process
GO:0010596: negative regulation of endothelial cell migration
GO:0001676: long-chain fatty acid metabolic process
GO:0010951: negative regulation of endopeptidase activity
GO:0030147: natriuresis
GO:0045879: negative regulation of smoothened signaling pathway
GO:0042936: dipeptide transporter activity
GO:0045329: carnitine biosynthetic process
GO:0034755: iron ion transmembrane transport
GO:0004000: adenosine deaminase activity
GO:0010936: negative regulation of macrophage cytokine production
GO:0051591: response to cAMP
GO:0014049: positive regulation of glutamate secretion
GO:0004435: phosphatidylinositol phospholipase C activity
GO:0005773: vacuole
GO:0048312: intracellular distribution of mitochondria
GO:0007274: neuromuscular synaptic transmission
GO:0009168: purine ribonucleoside monophosphate biosynthetic process
GO:0030902: hindbrain development
GO:0050748: negative regulation of lipoprotein metabolic process
GO:0090090: negative regulation of canonical Wnt receptor signaling pathway
GO:0006351: transcription, DNA-dependent
GO:0007044: cell-substrate junction assembly
GO:0030133: transport vesicle
GO:0000216: M/G1 transition of mitotic cell cycle
GO:0032809: neuronal cell body membrane
GO:0004336: galactosylceramidase activity
GO:0070050: neuron homeostasis
GO:0071320: cellular response to cAMP
GO:0045124: regulation of bone resorption
GO:0010524: positive regulation of calcium ion transport into cytosol
GO:0043235: receptor complex
GO:0043278: response to morphine
GO:0006767: water-soluble vitamin metabolic process
GO:0043097: pyrimidine nucleoside salvage
GO:0033300: dehydroascorbic acid transporter activity
GO:0021879: forebrain neuron differentiation
GO:0032496: response to lipopolysaccharide
GO:0016010: dystrophin-associated glycoprotein complex
GO:0005262: calcium channel activity
GO:0015267: channel activity
GO:0090023: positive regulation of neutrophil chemotaxis
GO:0030818: negative regulation of cAMP biosynthetic process
GO:0070555: response to interleukin-1
GO:0003700: sequence-specific DNA binding transcription factor activity
GO:0002090: regulation of receptor internalization
GO:0051789: response to protein stimulus
GO:0048661: positive regulation of smooth muscle cell proliferation
GO:0032868: response to insulin stimulus
GO:0046638: positive regulation of alpha-beta T cell differentiation
GO:0050728: negative regulation of inflammatory response
GO:0002021: response to dietary excess
GO:0043679: axon terminus
GO:0034138: toll-like receptor 3 signaling pathway
GO:0060484: lung-associated mesenchyme development
GO:0019442: tryptophan catabolic process to acetyl-CoA
GO:0005154: epidermal growth factor receptor binding
GO:0030667: secretory granule membrane
GO:0031234: extrinsic to internal side of plasma membrane
GO:0006066: alcohol metabolic process
GO:0001934: positive regulation of protein phosphorylation
GO:0006521: regulation of cellular amino acid metabolic process
GO:0031995: insulin-like growth factor II binding

GO:0006520: cellular amino acid metabolic process
GO:0009166: nucleotide catabolic process
GO:0032369: negative regulation of lipid transport
GO:0051545: negative regulation of elastin biosynthetic process
GO:0033555: multicellular organismal response to stress
GO:0032026: response to magnesium ion
GO:0010888: negative regulation of lipid storage
GO:0055056: D-glucose transmembrane transporter activity
GO:0016566: specific transcriptional repressor activity
GO:0004943: C3a anaphylatoxin receptor activity
GO:0000904: cell morphogenesis involved in differentiation
GO:0007266: Rho protein signal transduction
GO:0001958: endochondral ossification
GO:0046545: development of primary female sexual characteristics
GO:0016525: negative regulation of angiogenesis
GO:0042166: acetylcholine binding
GO:0043394: proteoglycan binding
GO:0045596: negative regulation of cell differentiation
GO:0005044: scavenger receptor activity
GO:0048167: regulation of synaptic plasticity
GO:0032755: positive regulation of interleukin-6 production
GO:0055037: recycling endosome
GO:0048487: beta-tubulin binding
GO:0043526: neuroprotection
GO:0090082: positive regulation of heart induction by negative regulation of canonical Wnt receptor signaling pathway
GO:0005606: laminin-1 complex
GO:0070491: repressing transcription factor binding
GO:0005088: Ras guanyl-nucleotide exchange factor activity
GO:0034372: very-low-density lipoprotein particle remodeling
GO:0008645: hexose transport
GO:0031258: lamellipodium membrane
GO:0032526: response to retinoic acid
GO:0051289: protein homotetramerization
GO:0048786: presynaptic active zone
GO:0034976: response to endoplasmic reticulum stress
GO:0042923: neuropeptide binding
GO:0043409: negative regulation of MAPKKK cascade
GO:0033391: chromatoid body
GO:0034599: cellular response to oxidative stress
GO:0015250: water channel activity
GO:0030901: midbrain development
GO:0006090: pyruvate metabolic process
GO:0060732: positive regulation of inositol phosphate biosynthetic process
GO:0004876: complement component C3a receptor activity
GO:0050918: positive chemotaxis
GO:0071377: cellular response to glucagon stimulus
GO:0031340: positive regulation of vesicle fusion
GO:0044130: negative regulation of growth of symbiont in host
GO:0042130: negative regulation of T cell proliferation
GO:0051149: positive regulation of muscle cell differentiation
GO:0006184: GTP catabolic process
GO:0008336: gamma-butyrobetaine dioxygenase activity
GO:0007416: synapse assembly
GO:0006766: vitamin metabolic process
GO:0032983: kainate selective glutamate receptor complex
GO:0048630: skeletal muscle tissue growth
GO:0007492: endoderm development
GO:0055086: nucleobase, nucleoside and nucleotide metabolic process
GO:0002430: complement receptor mediated signaling pathway
GO:0001850: complement component C3a binding
GO:0042692: muscle cell differentiation
GO:0051897: positive regulation of protein kinase B signaling cascade
GO:0014842: regulation of satellite cell proliferation
GO:0006644: phospholipid metabolic process
GO:0010002: cardioblast differentiation
GO:0015732: prostaglandin transport
GO:0001664: G-protein-coupled receptor binding
GO:0003680: AT DNA binding
GO:0042568: insulin-like growth factor binary complex
GO:0016043: cellular component organization
GO:0051259: protein oligomerization
GO:0016311: dephosphorylation
GO:0031781: type 3 melanocortin receptor binding
GO:0051403: stress-activated MAPK cascade
GO:0060055: angiogenesis involved in wound healing
GO:0046898: response to cycloheximide
GO:0048278: vesicle docking
GO:0045332: phospholipid translocation
GO:0050714: positive regulation of protein secretion
GO:0006898: receptor-mediated endocytosis
GO:0030819: positive regulation of cAMP biosynthetic process
GO:0030301: cholesterol transport
GO:0005502: 11-cis retinal binding
GO:0048755: branching morphogenesis of a nerve
GO:0006533: aspartate catabolic process
GO:0014824: artery smooth muscle contraction
GO:0001869: negative regulation of complement activation, lectin pathway
GO:0034186: apolipoprotein A-I binding
GO:0004806: triglyceride lipase activity
GO:0015186: L-glutamine transmembrane transporter activity
GO:0051234: establishment of localization
GO:0045542: positive regulation of cholesterol biosynthetic process
GO:0006730: one-carbon metabolic process
GO:0004850: uridine phosphorylase activity
GO:0060161: positive regulation of dopamine receptor signaling pathway
GO:0030855: epithelial cell differentiation
GO:0090050: positive regulation of cell migration involved in sprouting angiogenesis
GO:0035583: negative regulation of transforming growth factor beta receptor signaling pathway by extracellular sequestering of TGFbeta
GO:0004620: phospholipase activity
GO:0005902: microvillus
GO:0050776: regulation of immune response
GO:0034329: cell junction assembly
GO:0007613: memory
GO:0051593: response to folic acid
GO:0015101: organic cation transmembrane transporter activity
GO:0016044: cellular membrane organization
GO:0050660: flavin adenine dinucleotide binding
GO:0008081: phosphoric diester hydrolase activity
GO:0045715: negative regulation of low-density lipoprotein particle receptor biosynthetic process
GO:0060463: lung lobe morphogenesis
GO:0042136: neurotransmitter biosynthetic process
GO:0032760: positive regulation of tumor necrosis factor production
GO:0030073: insulin secretion
GO:0015132: prostaglandin transmembrane transporter activity
GO:0006853: carnitine shuttle
GO:0048566: embryonic digestive tract development
GO:0046485: ether lipid metabolic process
GO:0050804: regulation of synaptic transmission
GO:0015035: protein disulfide oxidoreductase activity
GO:0031333: negative regulation of protein complex assembly
GO:0005614: interstitial matrix
GO:0060337: type I interferon-mediated signaling pathway
GO:0008643: carbohydrate transport
GO:0048839: inner ear development
GO:0022891: substrate-specific transmembrane transporter activity
GO:0005545: 1-phosphatidylinositol binding
GO:0008543: fibroblast growth factor receptor signaling pathway
GO:0060346: bone trabecula formation
GO:0031224: intrinsic to membrane
GO:0055114: oxidation-reduction process

GO:0001933: negative regulation of protein phosphorylation
GO:0007088: regulation of mitosis
GO:0002092: positive regulation of receptor internalization
GO:0050891: multicellular organismal water homeostasis
GO:0043014: alpha-tubulin binding
GO:0042567: insulin-like growth factor ternary complex
GO:0001569: patterning of blood vessels
GO:0016597: amino acid binding
GO:0090303: positive regulation of wound healing
GO:0006486: protein glycosylation
GO:0010552: positive regulation of gene-specific transcription from RNA polymerase II promoter
GO:0035115: embryonic forelimb morphogenesis
GO:0009755: hormone-mediated signaling pathway
GO:0006069: ethanol oxidation
GO:0003987: acetate-CoA ligase activity
GO:0002040: sprouting angiogenesis
GO:0033267: axon part
GO:0042637: catagen
GO:0042895: antibiotic transporter activity
GO:0035116: embryonic hindlimb morphogenesis
GO:0007193: inhibition of adenylate cyclase activity by G-protein signaling pathway
GO:0001527: microfibril
GO:0030673: axolemma
GO:0051482: elevation of cytosolic calcium ion concentration involved in G-protein signaling coupled to IP3 second messenger
GO:0060174: limb bud formation
GO:0009117: nucleotide metabolic process
GO:0046135: pyrimidine nucleoside catabolic process
GO:0046427: positive regulation of JAK-STAT cascade
GO:0007512: adult heart development
GO:0014068: positive regulation of phosphatidylinositol 3-kinase cascade
GO:0047498: calcium-dependent phospholipase A2 activity
GO:0031398: positive regulation of protein ubiquitination
GO:0060445: branching involved in salivary gland morphogenesis
GO:0046426: negative regulation of JAK-STAT cascade
GO:0045671: negative regulation of osteoclast differentiation
GO:0051781: positive regulation of cell division
GO:0035313: wound healing, spreading of epidermal cells
GO:0032582: negative regulation of gene-specific transcription
GO:0046209: nitric oxide metabolic process
GO:0005791: rough endoplasmic reticulum
GO:0070935: 3'-UTR-mediated mRNA stabilization
GO:0019841: retinol binding
GO:0008202: steroid metabolic process
GO:0009952: anterior/posterior pattern formation
GO:0006468: protein phosphorylation
GO:0017075: syntaxin-1 binding
GO:0034220: ion transmembrane transport
GO:0001701: in utero embryonic development
GO:0031115: negative regulation of microtubule polymerization
GO:0008063: Toll signaling pathway
GO:0005626: insoluble fraction
GO:0014070: response to organic cyclic compound
GO:0031782: type 4 melanocortin receptor binding
GO:0051205: protein insertion into membrane
GO:0010243: response to organic nitrogen
GO:0000209: protein polyubiquitination
GO:0005921: gap junction
GO:0016079: synaptic vesicle exocytosis
GO:0045885: positive regulation of survival gene product expression
GO:0005242: inward rectifier potassium channel activity
GO:0030278: regulation of ossification
GO:0034707: chloride channel complex
GO:0019433: triglyceride catabolic process
GO:0043084: penile erection
GO:0050830: defense response to Gram-positive bacterium
GO:0008528: peptide receptor activity, G-protein coupled
GO:0055065: metal ion homeostasis
GO:0010037: response to carbon dioxide
GO:0070588: calcium ion transmembrane transport
GO:0007597: blood coagulation, intrinsic pathway
GO:0070837: dehydroascorbic acid transport
GO:0004999: vasoactive intestinal polypeptide receptor activity
GO:0034134: toll-like receptor 2 signaling pathway
GO:0002686: negative regulation of leukocyte migration
GO:0046855: inositol phosphate dephosphorylation
GO:0043691: reverse cholesterol transport
GO:0000786: nucleosome
GO:0060664: epithelial cell proliferation involved in salivary gland morphogenesis
GO:0001944: vasculature development
GO:0002224: toll-like receptor signaling pathway
GO:0008160: protein tyrosine phosphatase activator activity
GO:0034332: adherens junction organization
GO:0015030: Cajal body
GO:0048706: embryonic skeletal system development
GO:0030665: clathrin coated vesicle membrane
GO:0009268: response to pH
GO:0016941: natriuretic peptide receptor activity
GO:0004383: guanylate cyclase activity
GO:0033137: negative regulation of peptidyl-serine phosphorylation
GO:0031315: extrinsic to mitochondrial outer membrane
GO:0006154: adenosine catabolic process
GO:0006921: cellular component disassembly involved in apoptosis
GO:0004029: aldehyde dehydrogenase (NAD) activity
GO:0045765: regulation of angiogenesis
GO:0009790: embryo development
GO:0034142: toll-like receptor 4 signaling pathway
GO:0070256: negative regulation of mucus secretion
GO:0090263: positive regulation of canonical Wnt receptor signaling pathway
GO:0031783: type 5 melanocortin receptor binding
GO:0010842: retina layer formation
GO:0042393: histone binding
GO:0017091: AU-rich element binding
GO:0030514: negative regulation of BMP signaling pathway
GO:0006527: arginine catabolic process
GO:0010745: negative regulation of macrophage derived foam cell differentiation
GO:0016529: sarcoplasmic reticulum
GO:0042220: response to cocaine
GO:0070365: hepatocyte differentiation
GO:0016328: lateral plasma membrane
GO:0034375: high-density lipoprotein particle remodeling
GO:0046847: filopodium assembly
GO:0005372: water transmembrane transporter activity
GO:0060666: dichotomous subdivision of terminal units involved in salivary gland branching
GO:0050678: regulation of epithelial cell proliferation
GO:0051549: positive regulation of keratinocyte migration
GO:0045663: positive regulation of myoblast differentiation
GO:0033144: negative regulation of steroid hormone receptor signaling pathway
GO:0051924: regulation of calcium ion transport
GO:0005881: cytoplasmic microtubule
GO:0006182: cGMP biosynthetic process
GO:0007184: SMAD protein import into nucleus
GO:0051928: positive regulation of calcium ion transport
GO:0035050: embryonic heart tube development
GO:0002576: platelet degranulation
GO:0055085: transmembrane transport
GO:0048701: embryonic cranial skeleton morphogenesis
GO:0048843: negative regulation of axon extension involved in axon guidance
GO:0004030: aldehyde dehydrogenase [NAD(P)+] activity
GO:0048014: Tie receptor signaling pathway
GO:0019534: toxin transporter activity

GO:0045844: positive regulation of striated muscle tissue development
GO:0006206: pyrimidine base metabolic process
GO:0000083: regulation of transcription involved in G1/S phase of mitotic cell cycle
GO:0002070: epithelial cell maturation
GO:0004115: 3',5'-cyclic-AMP phosphodiesterase activity
GO:0005083: small GTPase regulator activity
GO:0055091: phospholipid homeostasis
GO:0015867: ATP transport
GO:0014032: neural crest cell development
GO:0031748: D1 dopamine receptor binding
GO:0032580: Golgi cisterna membrane
GO:0015758: glucose transport
GO:0070207: protein homotrimerization
GO:0032332: positive regulation of chondrocyte differentiation
GO:0019441: tryptophan catabolic process to kynurenine
GO:0050679: positive regulation of epithelial cell proliferation
GO:0030850: prostate gland development
GO:0042487: regulation of odontogenesis of dentine-containing tooth
GO:0030136: clathrin-coated vesicle
GO:0003333: amino acid transmembrane transport
GO:0014069: postsynaptic density
GO:0007168: receptor guanylyl cyclase signaling pathway
GO:0070742: C2H2 zinc finger domain binding
GO:0060916: mesenchymal cell proliferation involved in lung development
GO:0008179: adenylate cyclase binding
GO:0007029: endoplasmic reticulum organization
GO:0031622: positive regulation of fever generation
GO:0060548: negative regulation of cell death
GO:0014909: smooth muscle cell migration
GO:0060351: cartilage development involved in endochondral bone morphogenesis
GO:0030414: peptidase inhibitor activity
GO:0034641: cellular nitrogen compound metabolic process
GO:0010232: vascular transport
GO:0000922: spindle pole
GO:0060348: bone development
GO:0001599: endothelin-A receptor activity
GO:0035295: tube development
GO:0046108: uridine metabolic process
GO:0002053: positive regulation of mesenchymal cell proliferation
GO:0070295: renal water absorption
GO:0019905: syntaxin binding
GO:0048010: vascular endothelial growth factor receptor signaling pathway
GO:0007166: cell surface receptor linked signaling pathway
GO:0001649: osteoblast differentiation
GO:0070083: clathrin sculpted monoamine transport vesicle membrane
GO:0048557: embryonic digestive tract morphogenesis
GO:0031960: response to corticosteroid stimulus
GO:0060416: response to growth hormone stimulus
GO:0040036: regulation of fibroblast growth factor receptor signaling pathway
GO:0002755: MyD88-dependent toll-like receptor signaling pathway
GO:0030141: stored secretory granule
GO:0007162: negative regulation of cell adhesion
GO:0001883: purine nucleoside binding
GO:0006144: purine base metabolic process
GO:0055093: response to hyperoxia
GO:0030057: desmosome
GO:0046716: muscle cell homeostasis
GO:0010629: negative regulation of gene expression
GO:0031625: ubiquitin protein ligase binding
GO:0007398: ectoderm development
GO:0043237: laminin-1 binding
GO:0071438: invadopodium membrane
GO:0050750: low-density lipoprotein particle receptor binding
GO:0048568: embryonic organ development
GO:0010595: positive regulation of endothelial cell migration
GO:0005184: neuropeptide hormone activity
GO:0071398: cellular response to fatty acid
GO:0034199: activation of protein kinase A activity
GO:0010466: negative regulation of peptidase activity
GO:0006833: water transport
GO:0010628: positive regulation of gene expression
GO:0042277: peptide binding
GO:0010811: positive regulation of cell-substrate adhesion
GO:0010716: negative regulation of extracellular matrix disassembly
GO:0033574: response to testosterone stimulus
GO:0015695: organic cation transport
GO:0001657: ureteric bud development
GO:0033089: positive regulation of T cell differentiation in thymus
GO:0044255: cellular lipid metabolic process
GO:0030210: heparin biosynthetic process
GO:0031594: neuromuscular junction
GO:0030325: adrenal gland development
GO:0004439: phosphatidylinositol-4,5-bisphosphate 5-phosphatase activity
GO:0002756: MyD88-independent toll-like receptor signaling pathway
GO:0003007: heart morphogenesis
GO:0042787: protein ubiquitination involved in ubiquitin-dependent protein catabolic process
GO:0060333: interferon-gamma-mediated signaling pathway
GO:0030501: positive regulation of bone mineralization
GO:0046856: phosphatidylinositol dephosphorylation
GO:0005546: phosphatidylinositol-4,5-bisphosphate binding
GO:0034704: calcium channel complex
GO:0005811: lipid particle
GO:0019800: peptide cross-linking via chondroitin 4-sulfate glycosaminoglycan
GO:0043010: camera-type eye development
GO:0019933: cAMP-mediated signaling
GO:0035556: intracellular signal transduction
GO:0009954: proximal/distal pattern formation
GO:0005539: glycosaminoglycan binding
GO:0004175: endopeptidase activity
GO:0050881: musculoskeletal movement
GO:0002063: chondrocyte development
GO:0031092: platelet alpha granule membrane
GO:0048019: receptor antagonist activity
GO:0017137: Rab GTPase binding
GO:0043190: ATP-binding cassette (ABC) transporter complex
GO:0043116: negative regulation of vascular permeability
GO:0004465: lipoprotein lipase activity
GO:0045216: cell-cell junction organization
GO:0031175: neuron projection development
GO:0071346: cellular response to interferon-gamma
GO:0042562: hormone binding
GO:0003705: sequence-specific enhancer binding RNA polymerase II transcription factor activity
GO:0048870: cell motility
GO:0060523: prostate epithelial cord elongation
GO:0031780: adrenocorticotropin hormone receptor binding
GO:0006683: galactosylceramide catabolic process
GO:0005243: gap junction channel activity
GO:0090244: Wnt receptor signaling pathway involved in somitogenesis
GO:0005355: glucose transmembrane transporter activity
GO:0070996: type 1 melanocortin receptor binding
GO:0004962: endothelin receptor activity
GO:0034130: toll-like receptor 1 signaling pathway
GO:0051281: positive regulation of release of sequestered calcium ion into cytosol
GO:0050955: thermoception
GO:0006012: galactose metabolic process
GO:0043025: neuronal cell body
GO:0051602: response to electrical stimulus
GO:0042383: sarcolemma
GO:0043252: sodium-independent organic anion transport
#1043IR #1095IR 12 GO:0018345: protein palmitoylation

		GO:0032281: alpha-amino-3-hydroxy-5-methyl-4-isoxazolepropionic acid selective glutamate receptor complex
		GO:0030641: regulation of cellular pH
		GO:0005372: water transporter activity
		GO:0001887: selenium metabolic process
		GO:0015077: monovalent inorganic cation transmembrane transporter activity
		GO:0008889: glycerophosphodiester phosphodiesterase activity
		GO:0019992: diacylglycerol binding
		GO:0016409: palmitoyltransferase activity
		GO:0030317: sperm motility
		GO:0005890: sodium:potassium-exchanging ATPase complex
		GO:0006942: regulation of striated muscle contraction
#1051IR #1095IR	38	GO:0048154: S100 beta binding
		GO:0055114: oxidation reduction
		GO:0003700: transcription factor activity
		GO:0008109: N-acetyllactosaminide beta-1,6-N-acetylglucosaminyltransferase activity
		GO:0042801: polo kinase kinase activity
		GO:0006944: membrane fusion
		GO:0008037: cell recognition
		GO:0050660: FAD binding
		GO:0034614: cellular response to reactive oxygen species
		GO:0034644: cellular response to UV
		GO:0007338: single fertilization
		GO:0006497: protein amino acid lipidation
		GO:0005545: phosphatidylinositol binding
		GO:0048066: pigmentation during development
		GO:0006957: complement activation, alternative pathway
		GO:0007497: posterior midgut development
		GO:0006486: protein amino acid glycosylation
		GO:0007517: muscle development
		GO:0006468: protein amino acid phosphorylation
		GO:0030594: neurotransmitter receptor activity
		GO:0030286: dynein complex
		GO:0042953: lipoprotein transport
		GO:0015662: ATPase activity, coupled to transmembrane movement of ions, phosphorylative mechanism
		GO:0031953: negative regulation of protein amino acid autophosphorylation
		GO:0006350: transcription
		GO:0046777: protein amino acid autophosphorylation
		GO:0006470: protein amino acid dephosphorylation
		GO:0005005: transmembrane-ephrin receptor activity
		GO:0000775: chromosome, centromeric region
		GO:0007416: synaptogenesis
		GO:0019941: modification-dependent protein catabolic process
		GO:0030866: cortical actin cytoskeleton organization
		GO:0004653: polypeptide N-acetylgalactosaminyltransferase activity
		GO:0001816: cytokine production
		GO:0042598: vesicular fraction
		GO:0004864: phosphoprotein phosphatase inhibitor activity
		GO:0004411: homogentisate 1,2-dioxygenase activity
		GO:0006024: glycosaminoglycan biosynthetic process
#1063IR #1095IR	202	GO:0007089: traversing start control point of mitotic cell cycle
		GO:0003697: single-stranded DNA binding
		GO:0048245: eosinophil chemotaxis
		GO:0010033: response to organic substance
		GO:0019900: kinase binding
		GO:0009987: cellular process
		GO:0005796: Golgi lumen
		GO:0070309: lens fiber cell morphogenesis
		GO:0003690: double-stranded DNA binding
		GO:0004860: protein kinase inhibitor activity
		GO:0008242: omega peptidase activity
		GO:0021952: central nervous system projection neuron axonogenesis
		GO:0014059: regulation of dopamine secretion
		GO:0007176: regulation of epidermal growth factor receptor activity
		GO:0017147: Wnt-protein binding
		GO:0008584: male gonad development
		GO:0016327: apicolateral plasma membrane
		GO:0050860: negative regulation of T cell receptor signaling pathway
		GO:0048845: venous blood vessel morphogenesis
		GO:0050662: coenzyme binding
		GO:0008467: [heparan sulfate]-glucosamine 3-sulfotransferase 1 activity
		GO:0051537: 2 iron, 2 sulfur cluster binding
		GO:0045182: translation regulator activity
		GO:0021680: cerebellar Purkinje cell layer development
		GO:0006690: icosanoid metabolic process
		GO:0035162: embryonic hemopoiesis
		GO:0050659: N-acetylgalactosamine 4-sulfate 6-O-sulfotransferase activity
		GO:0007566: embryo implantation
		GO:0021987: cerebral cortex development
		GO:0019915: lipid storage
		GO:0004879: ligand-dependent nuclear receptor activity
		GO:0001508: regulation of action potential
		GO:0032839: dendrite cytoplasm
		GO:0005042: netrin receptor activity
		GO:0042246: tissue regeneration
		GO:0042989: sequestering of actin monomers
		GO:0030968: endoplasmic reticulum unfolded protein response
		GO:0008234: cysteine-type peptidase activity
		GO:0005740: mitochondrial envelope
		GO:0006310: DNA recombination
		GO:0007595: lactation
		GO:0015914: phospholipid transport
		GO:0060298: positive regulation of sarcomere organization
		GO:0000718: nucleotide-excision repair, DNA damage removal
		GO:0048037: cofactor binding
		GO:0045737: positive regulation of cyclin-dependent protein kinase activity
		GO:0009313: oligosaccharide catabolic process
		GO:0051233: spindle midzone
		GO:0050699: WW domain binding
		GO:0009306: protein secretion
		GO:0045429: positive regulation of nitric oxide biosynthetic process
		GO:0001974: blood vessel remodeling
		GO:0050730: regulation of peptidyl-tyrosine phosphorylation
		GO:0008585: female gonad development
		GO:0005100: Rho GTPase activator activity
		GO:0016853: isomerase activity
		GO:0007599: hemostasis
		GO:0032312: regulation of ARF GTPase activity
		GO:0043122: regulation of I-kappaB kinase/NF-kappaB cascade
		GO:0006302: double-strand break repair
		GO:0043407: negative regulation of MAP kinase activity
		GO:0001890: placenta development
		GO:0006412: translation
		GO:0048027: mRNA 5'-UTR binding
		GO:0001725: stress fiber
		GO:0016049: cell growth
		GO:0031667: response to nutrient levels
		GO:0060042: retina morphogenesis in camera-type eye
		GO:0050811: GABA receptor binding
		GO:0009566: fertilization
		GO:0060214: endocardium formation
		GO:0015026: coreceptor activity
		GO:0015807: L-amino acid transport
		GO:0001756: somitogenesis
		GO:0005095: GTPase inhibitor activity
		GO:0030246: carbohydrate binding
		GO:0043406: positive regulation of MAP kinase activity
		GO:0008466: glycogenin glucosyltransferase activity
		GO:0006297: nucleotide-excision repair, DNA gap filling
		GO:0046620: regulation of organ growth
		GO:0048012: hepatocyte growth factor receptor signaling pathway

		GO:0005484: SNAP receptor activity
		GO:0021516: dorsal spinal cord development
		GO:0009410: response to xenobiotic stimulus
		GO:0008156: negative regulation of DNA replication
		GO:0031072: heat shock protein binding
		GO:0060009: Sertoli cell development
		GO:0007628: adult walking behavior
		GO:0005782: peroxisomal matrix
		GO:0006491: N-glycan processing
		GO:0032947: protein complex scaffold
		GO:0045768: positive regulation of anti-apoptosis
		GO:0004527: exonuclease activity
		GO:0006914: autophagy
		GO:0008060: ARF GTPase activator activity
		GO:0042789: mRNA transcription from RNA polymerase II promoter
		GO:0008654: phospholipid biosynthetic process
		GO:0047485: protein N-terminus binding
		GO:0009437: carnitine metabolic process
		GO:0005008: hepatocyte growth factor receptor activity
		GO:0004221: ubiquitin thiolesterase activity
		GO:0030276: clathrin binding
		GO:0018298: protein-chromophore linkage
		GO:0000036: acyl carrier activity
		GO:0030504: inorganic diphosphate transmembrane transporter activity
		GO:0006820: anion transport
		GO:0015015: heparan sulfate proteoglycan biosynthetic process, enzymatic modification
		GO:0004519: endonuclease activity
		GO:0031201: SNARE complex
		GO:0015271: outward rectifier potassium channel activity
		GO:0005978: glycogen biosynthetic process
		GO:0045351: type I interferon biosynthetic process
		GO:0030910: olfactory placode formation
		GO:0016758: transferase activity, transferring hexosyl groups
		GO:0019674: NAD metabolic process
		GO:0000723: telomere maintenance
		GO:0015459: potassium channel regulator activity
		GO:0016579: protein deubiquitination
		GO:0009058: biosynthetic process
		GO:0042326: negative regulation of phosphorylation
		GO:0004571: mannosyl-oligosaccharide 1,2-alpha-mannosidase activity
		GO:0042133: neurotransmitter metabolic process
		GO:0043049: otic placode formation
		GO:0001709: cell fate determination
		GO:0016790: thiolester hydrolase activity
		GO:0008210: estrogen metabolic process
		GO:0003955: NAD(P)H dehydrogenase (quinone) activity
		GO:0050930: induction of positive chemotaxis
		GO:0015923: mannosidase activity
		GO:0001568: blood vessel development
		GO:0030532: small nuclear ribonucleoprotein complex
		GO:0042056: chemoattractant activity
		GO:0033872: [heparan sulfate]-glucosamine 3-sulfotransferase 3 activity
		GO:0060041: retina development in camera-type eye
		GO:0008191: metalloendopeptidase inhibitor activity
		GO:0005819: spindle
		GO:0016538: cyclin-dependent protein kinase regulator activity
		GO:0046329: negative regulation of JNK cascade
		GO:0021522: spinal cord motor neuron differentiation
		GO:0007269: neurotransmitter secretion
		GO:0006516: glycoprotein catabolic process
		GO:0005884: actin filament
		GO:0016605: PML body
		GO:0019319: hexose biosynthetic process
		GO:0004869: cysteine-type endopeptidase inhibitor activity
		GO:0032940: secretion by cell
		GO:0021702: cerebellar Purkinje cell differentiation
		GO:0051879: Hsp90 protein binding
		GO:0019864: IgG binding
		GO:0004128: cytochrome-b5 reductase activity
		GO:0000076: DNA replication checkpoint
		GO:0003950: NAD+ ADP-ribosyltransferase activity
		GO:0005801: cis-Golgi network
		GO:0008218: bioluminescence
		GO:0008503: benzodiazepine receptor activity
		GO:0003993: acid phosphatase activity
		GO:0019896: axon transport of mitochondrion
		GO:0006665: sphingolipid metabolic process
		GO:0047391: alkylglycerophosphoethanolamine phosphodiesterase activity
		GO:0042813: Wnt receptor activity
		GO:0030509: BMP signaling pathway
		GO:0000118: histone deacetylase complex
		GO:0005519: cytoskeletal regulatory protein binding
		GO:0021915: neural tube development
		GO:0019079: viral genome replication
		GO:0016126: sterol biosynthetic process
		GO:0004553: hydrolase activity, hydrolyzing O-glycosyl compounds
		GO:0045807: positive regulation of endocytosis
		GO:0015179: L-amino acid transmembrane transporter activity
		GO:0060076: excitatory synapse
		GO:0042035: regulation of cytokine biosynthetic process
		GO:0050885: neuromuscular process controlling balance
		GO:0008565: protein transporter activity
		GO:0035264: multicellular organism growth
		GO:0032769: negative regulation of monooxygenase activity
		GO:0019956: chemokine binding
		GO:0034341: response to interferon-gamma
		GO:0046928: regulation of neurotransmitter secretion
		GO:0017053: transcriptional repressor complex
		GO:0005315: inorganic phosphate transmembrane transporter activity
		GO:0009311: oligosaccharide metabolic process
		GO:0048511: rhythmic process
		GO:0016197: endosome transport
		GO:0004683: calmodulin-dependent protein kinase activity
		GO:0000151: ubiquitin ligase complex
		GO:0004721: phosphoprotein phosphatase activity
		GO:0055005: ventricular cardiac myofibril development
		GO:0007412: axon target recognition
		GO:0006289: nucleotide-excision repair
		GO:0008379: thioredoxin peroxidase activity
		GO:0004622: lysophospholipase activity
		GO:0006044: N-acetylglucosamine metabolic process
		GO:0001504: neurotransmitter uptake
		GO:0048247: lymphocyte chemotaxis
		GO:0051045: negative regulation of membrane protein ectodomain proteolysis
		GO:0008354: germ cell migration
		GO:0004091: carboxylesterase activity
		GO:0042744: hydrogen peroxide catabolic process
		GO:0060048: cardiac muscle contraction
		GO:0019867: outer membrane
		GO:0043205: fibril
		GO:0009925: basal plasma membrane
#1051IR #1063IR	10	GO:0016308: 1-phosphatidylinositol-4-phosphate 5-kinase activity
		GO:0048156: tau protein binding
		GO:0031018: endocrine pancreas development
		GO:0005898: interleukin-13 receptor complex
		GO:0032092: positive regulation of protein binding
		GO:0005596: collagen type XIV
		GO:0048814: regulation of dendrite morphogenesis
		GO:0014056: regulation of acetylcholine secretion
		GO:0045210: FasL biosynthetic process

		GO:0001822: kidney development
#10IR	571	GO:0008475: procollagen-lysine 5-dioxygenase activity
		GO:0032318: regulation of Ras GTPase activity
		GO:0006208: pyrimidine base catabolic process
		GO:0045995: regulation of embryonic development
		GO:0031849: olfactory receptor binding
		GO:0001841: neural tube formation
		GO:0032757: positive regulation of interleukin-8 production
		GO:0005913: cell-cell adherens junction
		GO:0016071: mRNA metabolic process
		GO:0032714: negative regulation of interleukin-5 production
		GO:0071565: nBAF complex
		GO:0045909: positive regulation of vasodilation
		GO:0032376: positive regulation of cholesterol transport
		GO:0030957: Tat protein binding
		GO:0007502: digestive tract mesoderm development
		GO:0045785: positive regulation of cell adhesion
		GO:0032420: stereocilium
		GO:0034993: SUN-KASH complex
		GO:0071285: cellular response to lithium ion
		GO:0005220: inositol 1,4,5-trisphosphate-sensitive calcium-release channel activity
		GO:0035590: purinergic nucleotide receptor signaling pathway
		GO:0010976: positive regulation of neuron projection development
		GO:0048625: myoblast cell fate commitment
		GO:0031683: G-protein beta/gamma-subunit complex binding
		GO:0070826: paraferritin complex
		GO:0016051: carbohydrate biosynthetic process
		GO:0051495: positive regulation of cytoskeleton organization
		GO:0007250: activation of NF-kappaB-inducing kinase activity
		GO:2000062: negative regulation of ureter smooth muscle cell differentiation
		GO:0031313: extrinsic to endosome membrane
		GO:0050434: positive regulation of viral transcription
		GO:0016595: glutamate binding
		GO:0015238: drug transmembrane transporter activity
		GO:0060046: regulation of acrosome reaction
		GO:0031095: platelet dense tubular network membrane
		GO:0031090: organelle membrane
		GO:0016540: protein autoprocessing
		GO:0060662: salivary gland cavitation
		GO:0001574: ganglioside biosynthetic process
		GO:0060405: regulation of penile erection
		GO:0043132: NAD transport
		GO:0021882: regulation of transcription from RNA polymerase II promoter involved in forebrain neuron fate commitment
		GO:0019985: translesion synthesis
		GO:0048844: artery morphogenesis
		GO:0030151: molybdenum ion binding
		GO:0048859: formation of anatomical boundary
		GO:0031094: platelet dense tubular network
		GO:0021940: positive regulation of cerebellar granule cell precursor proliferation
		GO:0004461: lactose synthase activity
		GO:0051209: release of sequestered calcium ion into cytosol
		GO:0009101: glycoprotein biosynthetic process
		GO:0060782: regulation of mesenchymal cell proliferation involved in prostate gland development
		GO:0010288: response to lead ion
		GO:0022602: ovulation cycle process
		GO:0001916: positive regulation of T cell mediated cytotoxicity
		GO:0014902: myotube differentiation
		GO:0008269: JAK pathway signal transduction adaptor activity
		GO:0043369: CD4-positive or CD8-positive, alpha-beta T cell lineage commitment
		GO:0005989: lactose biosynthetic process
		GO:0003214: cardiac left ventricle morphogenesis
		GO:0071421: manganese ion transmembrane transport
		GO:0070447: positive regulation of oligodendrocyte progenitor proliferation
		GO:0030331: estrogen receptor binding
		GO:0030854: positive regulation of granulocyte differentiation
		GO:0015295: solute:hydrogen symporter activity
		GO:0070682: proteasome regulatory particle assembly
		GO:2000114: regulation of establishment of cell polarity
		GO:0051901: positive regulation of mitochondrial depolarization
		GO:0007340: acrosome reaction
		GO:0034141: positive regulation of toll-like receptor 3 signaling pathway
		GO:0034364: high-density lipoprotein particle
		GO:0045741: positive regulation of epidermal growth factor receptor activity
		GO:0046578: regulation of Ras protein signal transduction
		GO:0030332: cyclin binding
		GO:0021893: cerebral cortex GABAergic interneuron fate commitment
		GO:0045727: positive regulation of translation
		GO:0070574: cadmium ion transmembrane transport
		GO:0046686: response to cadmium ion
		GO:0031572: G2/M transition DNA damage checkpoint
		GO:0042733: embryonic digit morphogenesis
		GO:0005814: centriole
		GO:0004087: carbamoyl-phosphate synthase (ammonia) activity
		GO:0032000: positive regulation of fatty acid beta-oxidation
		GO:0032229: negative regulation of synaptic transmission, GABAergic
		GO:0051001: negative regulation of nitric-oxide synthase activity
		GO:0042489: negative regulation of odontogenesis of dentine-containing tooth
		GO:0030274: LIM domain binding
		GO:0033017: sarcoplasmic reticulum membrane
		GO:0031214: biomineral tissue development
		GO:0006801: superoxide metabolic process
		GO:0010042: response to manganese ion
		GO:0016925: protein sumoylation
		GO:0032648: regulation of interferon-beta production
		GO:0051938: L-glutamate import
		GO:0009331: glycerol-3-phosphate dehydrogenase complex
		GO:0070053: thrombospondin receptor activity
		GO:0006196: AMP catabolic process
		GO:0071223: cellular response to lipoteichoic acid
		GO:0055072: iron ion homeostasis
		GO:0003727: single-stranded RNA binding
		GO:0031999: negative regulation of fatty acid beta-oxidation
		GO:0016524: latrotoxin receptor activity
		GO:0035253: ciliary rootlet
		GO:0051496: positive regulation of stress fiber assembly
		GO:0071864: positive regulation of cell proliferation in bone marrow
		GO:0016316: phosphatidylinositol-3,4-bisphosphate 4-phosphatase activity
		GO:0051894: positive regulation of focal adhesion assembly
		GO:0005639: integral to nuclear inner membrane
		GO:0045759: negative regulation of action potential
		GO:0050715: positive regulation of cytokine secretion
		GO:0071866: negative regulation of apoptosis in bone marrow
		GO:0006349: regulation of gene expression by genetic imprinting
		GO:0045136: development of secondary sexual characteristics
		GO:0040038: polar body extrusion after meiotic divisions
		GO:0005313: L-glutamate transmembrane transporter activity
		GO:0030388: fructose 1,6-bisphosphate metabolic process
		GO:0015085: calcium ion transmembrane transporter activity
		GO:0050829: defense response to Gram-negative bacterium
		GO:2000343: positive regulation of chemokine (C-X-C motif) ligand 2 production
		GO:0070409: carbamoyl phosphate biosynthetic process
		GO:0005113: patched binding
		GO:0005351: sugar:hydrogen symporter activity
		GO:0018119: peptidyl-cysteine S-nitrosylation
		GO:0034618: arginine binding
		GO:0042153: RPTP-like protein binding
		GO:0030155: regulation of cell adhesion
		GO:0004368: glycerol-3-phosphate dehydrogenase activity
		GO:0005795: Golgi stack
		GO:0008253: 5'-nucleotidase activity

GO:0005094: Rho GDP-dissociation inhibitor activity
GO:0016528: sarcoplasm
GO:0015676: vanadium ion transport
GO:0042098: T cell proliferation
GO:0015631: tubulin binding
GO:0004499: flavin-containing monooxygenase activity
GO:0060439: trachea morphogenesis
GO:0015100: vanadium ion transmembrane transporter activity
GO:0015299: solute:hydrogen antiporter activity
GO:0007228: positive regulation of hh target transcription factor activity
GO:0043923: positive regulation by host of viral transcription
GO:0035434: copper ion transmembrane transport
GO:0031953: negative regulation of protein autophosphorylation
GO:0046685: response to arsenic-containing substance
GO:0048715: negative regulation of oligodendrocyte differentiation
GO:0004835: tubulin-tyrosine ligase activity
GO:0006004: fucose metabolic process
GO:0006956: complement activation
GO:0071548: response to dexamethasone stimulus
GO:0016151: nickel ion binding
GO:0045880: positive regulation of smoothened signaling pathway
GO:0090022: regulation of neutrophil chemotaxis
GO:0006651: diacylglycerol biosynthetic process
GO:0005104: fibroblast growth factor receptor binding
GO:0031981: nuclear lumen
GO:0070679: inositol 1,4,5 trisphosphate binding
GO:0046168: glycerol-3-phosphate catabolic process
GO:0042178: xenobiotic catabolic process
GO:0001660: fever generation
GO:0007494: midgut development
GO:0014043: negative regulation of neuron maturation
GO:0035444: nickel ion transmembrane transport
GO:0030131: clathrin adaptor complex
GO:0050482: arachidonic acid secretion
GO:0032993: protein-DNA complex
GO:0061056: sclerotome development
GO:0048592: eye morphogenesis
GO:0090068: positive regulation of cell cycle process
GO:0060783: mesenchymal smoothened signaling pathway involved in prostate gland development
GO:2000357: negative regulation of kidney smooth muscle cell differentiation
GO:0002793: positive regulation of peptide secretion
GO:0035663: Toll-like receptor 2 binding
GO:0007224: smoothened signaling pathway
GO:0007132: meiotic metaphase I
GO:0060406: positive regulation of penile erection
GO:0015884: folic acid transport
GO:0006972: hyperosmotic response
GO:0045178: basal part of cell
GO:0050717: positive regulation of interleukin-1 alpha secretion
GO:0006649: phospholipid transfer to membrane
GO:0046888: negative regulation of hormone secretion
GO:0006868: glutamine transport
GO:0004607: phosphatidylcholine-sterol O-acyltransferase activity
GO:0060259: regulation of feeding behavior
GO:0034501: protein localization to kinetochore
GO:0032060: bleb assembly
GO:0032348: negative regulation of aldosterone biosynthetic process
GO:0043032: positive regulation of macrophage activation
GO:0032224: positive regulation of synaptic transmission, cholinergic
GO:0035326: enhancer binding
GO:0060025: regulation of synaptic activity
GO:0001658: branching involved in ureteric bud morphogenesis
GO:0060428: lung epithelium development
GO:0051155: positive regulation of striated muscle cell differentiation
GO:0016628: oxidoreductase activity, acting on the CH-CH group of donors, NAD or NADP as acceptor
GO:0004560: alpha-L-fucosidase activity
GO:0009411: response to UV
GO:0005610: laminin-5 complex
GO:0043236: laminin binding
GO:0010039: response to iron ion
GO:0060458: right lung development
GO:0060135: maternal process involved in female pregnancy
GO:0007097: nuclear migration
GO:0008417: fucosyltransferase activity
GO:0006930: substrate-dependent cell migration, cell extension
GO:0033004: negative regulation of mast cell activation
GO:0048738: cardiac muscle tissue development
GO:0048714: positive regulation of oligodendrocyte differentiation
GO:0022408: negative regulation of cell-cell adhesion
GO:0046639: negative regulation of alpha-beta T cell differentiation
GO:0072568: protein kinase C delta binding
GO:0035091: phosphatidylinositol binding
GO:0032738: positive regulation of interleukin-15 production
GO:0030049: muscle filament sliding
GO:0001759: organ induction
GO:0003990: acetylcholinesterase activity
GO:0003032: detection of oxygen
GO:0003093: regulation of glomerular filtration
GO:0016344: meiotic chromosome movement towards spindle pole
GO:0015116: sulfate transmembrane transporter activity
GO:0050031: L-pipecolate oxidase activity
GO:0050995: negative regulation of lipid catabolic process
GO:0045652: regulation of megakaryocyte differentiation
GO:0003407: neural retina development
GO:0008272: sulfate transport
GO:0016539: intein-mediated protein splicing
GO:0007009: plasma membrane organization
GO:0030030: cell projection organization
GO:0031668: cellular response to extracellular stimulus
GO:0006828: manganese ion transport
GO:0042323: negative regulation of circadian sleep/wake cycle, non-REM sleep
GO:0004950: chemokine receptor activity
GO:0006884: cell volume homeostasis
GO:0030284: estrogen receptor activity
GO:0033198: response to ATP
GO:0045059: positive thymic T cell selection
GO:0015093: ferrous iron transmembrane transporter activity
GO:0045822: negative regulation of heart contraction
GO:0016514: SWI/SNF complex
GO:0051712: positive regulation of killing of cells of other organism
GO:0045777: positive regulation of blood pressure
GO:0033077: T cell differentiation in thymus
GO:0044429: mitochondrial part
GO:0042742: defense response to bacterium
GO:0051439: regulation of ubiquitin-protein ligase activity involved in mitotic cell cycle
GO:0014732: skeletal muscle atrophy
GO:0005640: nuclear outer membrane
GO:0005588: collagen type V
GO:0004367: glycerol-3-phosphate dehydrogenase [NAD+] activity
GO:0031022: nuclear migration along microfilament
GO:0045776: negative regulation of blood pressure
GO:0009953: dorsal/ventral pattern formation
GO:0042474: middle ear morphogenesis
GO:0030126: COPI vesicle coat
GO:0005384: manganese ion transmembrane transporter activity
GO:0048617: embryonic foregut morphogenesis
GO:0008209: androgen metabolic process
GO:0001955: blood vessel maturation
GO:0000185: activation of MAPKKK activity
GO:0046534: positive regulation of photoreceptor cell differentiation

GO:0034123: positive regulation of toll-like receptor signaling pathway
GO:0006839: mitochondrial transport
GO:0048771: tissue remodeling
GO:0004332: fructose-bisphosphate aldolase activity
GO:0033265: choline binding
GO:0050718: positive regulation of interleukin-1 beta secretion
GO:0001614: purinergic nucleotide receptor activity
GO:0060738: epithelial-mesenchymal signaling involved in prostate gland development
GO:0008353: RNA polymerase II carboxy-terminal domain kinase activity
GO:0045779: negative regulation of bone resorption
GO:0006900: membrane budding
GO:0007217: tachykinin receptor signaling pathway
GO:0004931: extracellular ATP-gated cation channel activity
GO:0032308: positive regulation of prostaglandin secretion
GO:0045109: intermediate filament organization
GO:0006259: DNA metabolic process
GO:0070097: delta-catenin binding
GO:0090073: positive regulation of protein homodimerization activity
GO:0001708: cell fate specification
GO:0008504: monoamine transmembrane transporter activity
GO:0060586: multicellular organismal iron ion homeostasis
GO:0051967: negative regulation of synaptic transmission, glutamatergic
GO:0005148: prolactin receptor binding
GO:0047496: vesicle transport along microtubule
GO:0032728: positive regulation of interferon-beta production
GO:0048645: organ formation
GO:0045359: positive regulation of interferon-beta biosynthetic process
GO:0046486: glycerolipid metabolic process
GO:0030658: transport vesicle membrane
GO:0015385: sodium:hydrogen antiporter activity
GO:0004301: epoxide hydrolase activity
GO:0051287: NAD binding
GO:0021978: telencephalon regionalization
GO:0010467: gene expression
GO:0001845: phagolysosome assembly
GO:0005903: brush border
GO:0007520: myoblast fusion
GO:0045410: positive regulation of interleukin-6 biosynthetic process
GO:0030035: microspike assembly
GO:0021772: olfactory bulb development
GO:0048016: inositol phosphate-mediated signaling
GO:0032228: regulation of synaptic transmission, GABAergic
GO:0060020: Bergmann glial cell differentiation
GO:0060054: positive regulation of epithelial cell proliferation involved in wound healing
GO:0021521: ventral spinal cord interneuron specification
GO:0034339: regulation of transcription from RNA polymerase II promoter by nuclear hormone receptor
GO:0044325: ion channel binding
GO:0015180: L-alanine transmembrane transporter activity
GO:0090197: positive regulation of chemokine secretion
GO:0055038: recycling endosome membrane
GO:0005367: myo-inositol:sodium symporter activity
GO:0045356: positive regulation of interferon-alpha biosynthetic process
GO:0071221: cellular response to bacterial lipopeptide
GO:0008970: phospholipase A1 activity
GO:0050897: cobalt ion binding
GO:0007032: endosome organization
GO:0035662: Toll-like receptor 4 binding
GO:0071353: cellular response to interleukin-4
GO:0034145: positive regulation of toll-like receptor 4 signaling pathway
GO:0007339: binding of sperm to zona pellucida
GO:0045908: negative regulation of vasodilation
GO:0051437: positive regulation of ubiquitin-protein ligase activity involved in mitotic cell cycle
GO:0072136: metanephric mesenchymal cell proliferation involved in metanephros development
GO:0019532: oxalate transport
GO:0045294: alpha-catenin binding
GO:0009629: response to gravity
GO:0051709: regulation of killing of cells of other organism
GO:0006937: regulation of muscle contraction
GO:0005290: L-histidine transmembrane transporter activity
GO:0010983: positive regulation of high-density lipoprotein particle clearance
GO:0009922: fatty acid elongase activity
GO:0033602: negative regulation of dopamine secretion
GO:0002866: positive regulation of acute inflammatory response to antigenic stimulus
GO:0005605: basal lamina
GO:0090041: negative regulation of gene-specific transcription elongation from RNA polymerase II promoter
GO:0048808: male genitalia morphogenesis
GO:0030223: neutrophil differentiation
GO:0051295: establishment of meiotic spindle localization
GO:0050661: NADP binding
GO:0034617: tetrahydrobiopterin binding
GO:0015099: nickel ion transmembrane transporter activity
GO:0007418: ventral midline development
GO:0010977: negative regulation of neuron projection development
GO:0000050: urea cycle
GO:0017034: Rap guanyl-nucleotide exchange factor activity
GO:0022890: inorganic cation transmembrane transporter activity
GO:0031526: brush border membrane
GO:0019531: oxalate transmembrane transporter activity
GO:0019290: siderophore biosynthetic process
GO:0035250: UDP-galactosyltransferase activity
GO:0048484: enteric nervous system development
GO:0002028: regulation of sodium ion transport
GO:0001530: lipopolysaccharide binding
GO:0032696: negative regulation of interleukin-13 production
GO:0016578: histone deubiquitination
GO:0001609: adenosine receptor activity, G-protein coupled
GO:0046086: adenosine biosynthetic process
GO:0051436: negative regulation of ubiquitin-protein ligase activity involved in mitotic cell cycle
GO:0090286: cytoskeletal anchoring at nuclear membrane
GO:0051270: regulation of cellular component movement
GO:0034230: enkephalin processing
GO:2000065: negative regulation of cortisol biosynthetic process
GO:0046931: pore complex assembly
GO:0042761: very long-chain fatty acid biosynthetic process
GO:0032900: negative regulation of neurotrophin production
GO:0046653: tetrahydrofolate metabolic process
GO:0016986: transcription initiation factor activity
GO:0030183: B cell differentiation
GO:0004517: nitric-oxide synthase activity
GO:0006195: purine nucleotide catabolic process
GO:0005871: kinesin complex
GO:0003725: double-stranded RNA binding
GO:0006829: zinc ion transport
GO:0014741: negative regulation of muscle hypertrophy
GO:0045060: negative thymic T cell selection
GO:0050890: cognition
GO:0048589: developmental growth
GO:0035665: TIRAP-dependent toll-like receptor 4 signaling pathway
GO:0014050: negative regulation of glutamate secretion
GO:0019240: citrulline biosynthetic process
GO:0071400: cellular response to oleic acid
GO:0030502: negative regulation of bone mineralization
GO:0002076: osteoblast development
GO:0010043: response to zinc ion
GO:0008095: inositol-1,4,5-trisphosphate receptor activity
GO:0032279: asymmetric synapse
GO:0033276: transcription factor TFTC complex
GO:0015817: histidine transport
GO:0072199: regulation of mesenchymal cell proliferation involved in ureter development
GO:0015182: L-asparagine transmembrane transporter activity

GO:0002087: regulation of respiratory gaseous exchange by neurological system process
GO:0004513: neolactotetraosylceramide alpha-2,3-sialyltransferase activity
GO:0031821: metabotropic serotonin receptor binding
GO:0005218: intracellular ligand-gated calcium channel activity
GO:0014054: positive regulation of gamma-aminobutyric acid secretion
GO:0035338: long-chain fatty-acyl-CoA biosynthetic process
GO:0032835: glomerulus development
GO:0034405: response to fluid shear stress
GO:0021513: spinal cord dorsal/ventral patterning
GO:0030856: regulation of epithelial cell differentiation
GO:0043271: negative regulation of ion transport
GO:0045078: positive regulation of interferon-gamma biosynthetic process
GO:0051290: protein heterotetramerization
GO:0071899: negative regulation of estrogen receptor binding
GO:0005131: growth hormone receptor binding
GO:0071577: zinc ion transmembrane transport
GO:2000063: positive regulation of ureter smooth muscle cell differentiation
GO:2000358: positive regulation of kidney smooth muscle cell differentiation
GO:0031527: filopodium membrane
GO:0002227: innate immune response in mucosa
GO:0006116: NADH oxidation
GO:0048864: stem cell development
GO:0050543: icosatetraenoic acid binding
GO:0031685: adenosine receptor binding
GO:0002320: lymphoid progenitor cell differentiation
GO:0015094: lead ion transmembrane transporter activity
GO:0060326: cell chemotaxis
GO:0070627: ferrous iron import
GO:0030574: collagen catabolic process
GO:0042113: B cell activation
GO:0004864: protein phosphatase inhibitor activity
GO:0031117: positive regulation of microtubule depolymerization
GO:0046923: ER retention sequence binding
GO:0006513: protein monoubiquitination
GO:0031088: platelet dense granule membrane
GO:0045063: T-helper 1 cell differentiation
GO:0006977: DNA damage response, signal transduction by p53 class mediator resulting in cell cycle arrest
GO:0030010: establishment of cell polarity
GO:0071436: sodium ion export
GO:0003858: 3-hydroxybutyrate dehydrogenase activity
GO:0042589: zymogen granule membrane
GO:0044212: transcription regulatory region DNA binding
GO:0043967: histone H4 acetylation
GO:0034597: phosphatidylinositol-4,5-bisphosphate 4-phosphatase activity
GO:0016358: dendrite development
GO:0034605: cellular response to heat
GO:0051642: centrosome localization
GO:0004095: carnitine O-palmitoyltransferase activity
GO:0006612: protein targeting to membrane
GO:0042307: positive regulation of protein import into nucleus
GO:0060170: cilium membrane
GO:0015808: L-alanine transport
GO:0070542: response to fatty acid
GO:0032059: bleb
GO:0032403: protein complex binding
GO:0015844: monoamine transport
GO:0042100: B cell proliferation
GO:0050805: negative regulation of synaptic transmission
GO:0000293: ferric-chelate reductase activity
GO:0016791: phosphatase activity
GO:0005212: structural constituent of eye lens
GO:0060058: positive regulation of apoptosis involved in mammary gland involution
GO:0060685: regulation of prostatic bud formation
GO:0048643: positive regulation of skeletal muscle tissue development
GO:0032435: negative regulation of proteasomal ubiquitin-dependent protein catabolic process
GO:0030099: myeloid cell differentiation
GO:0031581: hemidesmosome assembly
GO:0016485: protein processing
GO:0003831: beta-N-acetylglucosaminylglycopeptide beta-1,4-galactosyltransferase activity
GO:0008020: G-protein coupled photoreceptor activity
GO:0055118: negative regulation of cardiac muscle contraction
GO:0006406: mRNA export from nucleus
GO:0008540: proteasome regulatory particle, base subcomplex
GO:0002064: epithelial cell development
GO:0046513: ceramide biosynthetic process
GO:0002162: dystroglycan binding
GO:0034231: islet amyloid polypeptide processing
GO:0008093: cytoskeletal adaptor activity
GO:0006621: protein retention in ER lumen
GO:0015106: bicarbonate transmembrane transporter activity
GO:0009584: detection of visible light
GO:0047291: lactosylceramide alpha-2,3-sialyltransferase activity
GO:0016998: cell wall macromolecule catabolic process
GO:0034626: fatty acid elongation, polyunsaturated fatty acid
GO:0032387: negative regulation of intracellular transport
GO:0060447: bud outgrowth involved in lung branching
GO:0060840: artery development
GO:0060516: primary prostatic bud elongation
GO:0042346: positive regulation of NF-kappaB import into nucleus
GO:0046632: alpha-beta T cell differentiation
GO:0001912: positive regulation of leukocyte mediated cytotoxicity
GO:0046332: SMAD binding
GO:0043615: astrocyte cell migration
GO:0031100: organ regeneration
GO:0033092: positive regulation of immature T cell proliferation in thymus
GO:0042168: heme metabolic process
GO:0001578: microtubule bundle formation
GO:0000138: Golgi trans cisterna
GO:2000340: positive regulation of chemokine (C-X-C motif) ligand 1 production
GO:0014858: positive regulation of skeletal muscle cell proliferation
GO:0030112: glycocalyx
GO:0051895: negative regulation of focal adhesion assembly
GO:0044344: cellular response to fibroblast growth factor stimulus
GO:0048646: anatomical structure formation involved in morphogenesis
GO:0007026: negative regulation of microtubule depolymerization
GO:0007442: hindgut morphogenesis
GO:0006470: protein dephosphorylation
GO:0043401: steroid hormone mediated signaling pathway
GO:0048632: negative regulation of skeletal muscle tissue growth
GO:0016615: malate dehydrogenase activity
GO:0045445: myoblast differentiation
GO:0001948: glycoprotein binding
GO:0034137: positive regulation of toll-like receptor 2 signaling pathway
GO:0015684: ferrous iron transport
GO:0006867: asparagine transport
GO:0048821: erythrocyte development
GO:0030070: insulin processing
GO:0014706: striated muscle tissue development
GO:0015301: anion:anion antiporter activity
GO:0043268: positive regulation of potassium ion transport
GO:0060459: left lung development
GO:0050746: regulation of lipoprotein metabolic process
GO:0021938: smoothened signaling pathway involved in regulation of cerebellar granule cell precursor cell proliferation
GO:0015086: cadmium ion transmembrane transporter activity
GO:0004086: carbamoyl-phosphate synthase activity
GO:0043279: response to alkaloid
GO:0009597: detection of virus
GO:0042308: negative regulation of protein import into nucleus
GO:0007257: activation of JUN kinase activity
GO:0006000: fructose metabolic process

GO:0031464: Cul4A-RING ubiquitin ligase complex
GO:0004629: phospholipase C activity
GO:0010740: positive regulation of intracellular protein kinase cascade
GO:0019834: phospholipase A2 inhibitor activity
GO:0015081: sodium ion transmembrane transporter activity
GO:0030878: thyroid gland development
GO:0021904: dorsal/ventral neural tube patterning
GO:0060769: positive regulation of epithelial cell proliferation involved in prostate gland development
GO:0031465: Cul4B-RING ubiquitin ligase complex
GO:0019395: fatty acid oxidation
GO:0005314: high-affinity glutamate transmembrane transporter activity
GO:0034435: cholesterol esterification
GO:0019367: fatty acid elongation, saturated fatty acid
GO:0043392: negative regulation of DNA binding
GO:0043587: tongue morphogenesis
GO:0008486: diphosphoinositol-polyphosphate diphosphatase activity
GO:0015087: cobalt ion transmembrane transporter activity
GO:0017144: drug metabolic process
GO:0032244: positive regulation of nucleoside transport
GO:0032795: heterotrimeric G-protein binding
GO:0004402: histone acetyltransferase activity
GO:0043029: T cell homeostasis
GO:0003920: GMP reductase activity
GO:0005876: spindle microtubule
GO:0030914: STAGA complex
GO:0015675: nickel ion transport
GO:0006824: cobalt ion transport
GO:0005932: microtubule basal body
GO:0005385: zinc ion transmembrane transporter activity
GO:0008328: ionotropic glutamate receptor complex
GO:0006606: protein import into nucleus
GO:0032735: positive regulation of interleukin-12 production
GO:0032713: negative regulation of interleukin-4 production
GO:0005272: sodium channel activity
GO:0002052: positive regulation of neuroblast proliferation
GO:0080125: multicellular structure septum development
GO:0044254: multicellular organismal protein catabolic process
GO:0032591: dendritic spine membrane
GO:0048155: S100 alpha binding
GO:0016705: oxidoreductase activity, acting on paired donors, with incorporation or reduction of molecular oxygen
GO:0030742: GTP-dependent protein binding
GO:0008115: sarcosine oxidase activity
GO:0015692: lead ion transport
GO:0030217: T cell differentiation
GO:0070779: D-aspartate import
GO:0005542: folic acid binding
GO:0050965: detection of temperature stimulus involved in sensory perception of pain
GO:0070016: armadillo repeat domain binding
GO:0055081: anion homeostasis
GO:0042158: lipoprotein biosynthetic process
GO:0048314: embryo sac morphogenesis
GO:0005324: long-chain fatty acid transporter activity
#1095IR 753 GO:0048676: axon extension involved in development
GO:0032052: bile acid binding
GO:0009790: embryonic development
GO:0006807: nitrogen compound metabolic process
GO:0007519: skeletal muscle development
GO:0007212: dopamine receptor signaling pathway
GO:0009434: microtubule-based flagellum
GO:0010766: negative regulation of sodium ion transport
GO:0005761: mitochondrial ribosome
GO:0050665: hydrogen peroxide biosynthetic process
GO:0004828: serine-tRNA ligase activity
GO:0004017: adenylate kinase activity
GO:0032781: positive regulation of ATPase activity
GO:0004813: alanine-tRNA ligase activity
GO:0043754: dihydrolipoyllysine-residue (2-methylpropanoyl)transferase activity
GO:0031105: septin complex
GO:0006413: translational initiation
GO:0019911: structural constituent of myelin sheath
GO:0046933: hydrogen ion transporting ATP synthase activity, rotational mechanism
GO:0004579: dolichyl-diphosphooligosaccharide-protein glycotransferase activity
GO:0042384: cilium assembly
GO:0014068: positive regulation of phosphoinositide 3-kinase cascade
GO:0045747: positive regulation of Notch signaling pathway
GO:0019835: cytolysis
GO:0043507: positive regulation of JUN kinase activity
GO:0005885: Arp2/3 protein complex
GO:0051603: proteolysis involved in cellular protein catabolic process
GO:0009755: hormone-mediated signaling
GO:0006857: oligopeptide transport
GO:0051538: 3 iron, 4 sulfur cluster binding
GO:0004825: methionine-tRNA ligase activity
GO:0033179: proton-transporting V-type ATPase, V0 domain
GO:0004347: glucose-6-phosphate isomerase activity
GO:0043498: cell surface binding
GO:0009086: methionine biosynthetic process
GO:0003983: UTP:glucose-1-phosphate uridylyltransferase activity
GO:0006694: steroid biosynthetic process
GO:0008494: translation activator activity
GO:0018193: peptidyl-amino acid modification
GO:0047042: 3-alpha-hydroxysteroid dehydrogenase (B-specific) activity
GO:0031571: G1 DNA damage checkpoint
GO:0003954: NADH dehydrogenase activity
GO:0042511: positive regulation of tyrosine phosphorylation of Stat1 protein
GO:0015520: tetracycline:hydrogen antiporter activity
GO:0051346: negative regulation of hydrolase activity
GO:0006959: humoral immune response
GO:0004800: thyroxine 5'-deiodinase activity
GO:0003980: UDP-glucose:glycoprotein glucosyltransferase activity
GO:0006590: thyroid hormone generation
GO:0045636: positive regulation of melanocyte differentiation
GO:0001601: peptide YY receptor activity
GO:0031669: cellular response to nutrient levels
GO:0051016: barbed-end actin filament capping
GO:0048269: methionine adenosyltransferase complex
GO:0005151: interleukin-1, Type II receptor binding
GO:0035091: phosphoinositide binding
GO:0004337: geranyltranstransferase activity
GO:0000224: peptide-N4-(N-acetyl-beta-glucosaminyl)asparagine amidase activity
GO:0004984: olfactory receptor activity
GO:0046902: regulation of mitochondrial membrane permeability
GO:0005762: mitochondrial large ribosomal subunit
GO:0016229: steroid dehydrogenase activity
GO:0050815: phosphoserine binding
GO:0018879: biphenyl metabolic process
GO:0007369: gastrulation
GO:0019902: phosphatase binding
GO:0045298: tubulin complex
GO:0005000: vasopressin receptor activity
GO:0017183: peptidyl-diphthamide biosynthetic process from peptidyl-histidine
GO:0005839: proteasome core complex
GO:0005763: mitochondrial small ribosomal subunit
GO:0005859: muscle myosin complex
GO:0006563: L-serine metabolic process
GO:0015457: auxiliary transport protein activity
GO:0015272: ATP-activated inward rectifier potassium channel activity
GO:0005643: nuclear pore
GO:0046934: phosphatidylinositol-4,5-bisphosphate 3-kinase activity
GO:0001945: lymph vessel development

GO:0021954: central nervous system neuron development
GO:0046961: proton-transporting ATPase activity, rotational mechanism
GO:0032287: myelin maintenance in the peripheral nervous system
GO:0008061: chitin binding
GO:0008449: N-acetylglucosamine-6-sulfatase activity
GO:0045815: positive regulation of gene expression, epigenetic
GO:0052171: growth or development during symbiotic interaction
GO:0015450: P-P-bond-hydrolysis-driven protein transmembrane transporter activity
GO:0021965: spinal cord ventral commissure morphogenesis
GO:0016786: selenotransferase activity
GO:0019008: molybdopterin synthase complex
GO:0005840: ribosome
GO:0006368: RNA elongation from RNA polymerase II promoter
GO:0045475: locomotor rhythm
GO:0004697: protein kinase C activity
GO:0006519: cellular amino acid and derivative metabolic process
GO:0045840: positive regulation of mitosis
GO:0001895: retina homeostasis
GO:0005942: phosphoinositide 3-kinase complex
GO:0005744: mitochondrial inner membrane presequence translocase complex
GO:0004905: type I interferon receptor activity
GO:0006431: methionyl-tRNA aminoacylation
GO:0051146: striated muscle cell differentiation
GO:0002318: myeloid progenitor cell differentiation
GO:0006027: glycosaminoglycan catabolic process
GO:0048861: leukemia inhibitory factor signaling pathway
GO:0004035: alkaline phosphatase activity
GO:0035254: glutamate receptor binding
GO:0004743: pyruvate kinase activity
GO:0008479: queuine tRNA-ribosyltransferase activity
GO:0004174: electron-transferring-flavoprotein dehydrogenase activity
GO:0015904: tetracycline transport
GO:0032729: positive regulation of interferon-gamma production
GO:0016846: carbon-sulfur lyase activity
GO:0003729: mRNA binding
GO:0043209: myelin sheath
GO:0005851: eukaryotic translation initiation factor 2B complex
GO:0050661: NADP or NADPH binding
GO:0000275: mitochondrial proton-transporting ATP synthase complex, catalytic core F(1)
GO:0000119: mediator complex
GO:0005746: mitochondrial respiratory chain
GO:0004656: procollagen-proline 4-dioxygenase activity
GO:0030515: snoRNA binding
GO:0042423: catecholamine biosynthetic process
GO:0001817: regulation of cytokine production
GO:0032038: myosin II heavy chain binding
GO:0004771: sterol esterase activity
GO:0015248: sterol transporter activity
GO:0004569: glycoprotein endo-alpha-1,2-mannosidase activity
GO:0006414: translational elongation
GO:0030071: regulation of mitotic metaphase/anaphase transition
GO:0008616: queuosine biosynthetic process
GO:0006817: phosphate transport
GO:0005139: interleukin-7 receptor binding
GO:0051450: myoblast proliferation
GO:0000247: C-8 sterol isomerase activity
GO:0006122: mitochondrial electron transport, ubiquinol to cytochrome c
GO:0030060: L-malate dehydrogenase activity
GO:0033119: negative regulation of RNA splicing
GO:0009303: rRNA transcription
GO:0045648: positive regulation of erythrocyte differentiation
GO:0008177: succinate dehydrogenase (ubiquinone) activity
GO:0004134: 4-alpha-glucanotransferase activity
GO:0045899: positive regulation of transcriptional preinitiation complex assembly
GO:0019206: nucleoside kinase activity
GO:0045843: negative regulation of striated muscle development
GO:0000254: C-4 methylsterol oxidase activity
GO:0004504: peptidylglycine monooxygenase activity
GO:0051287: NAD or NADH binding
GO:0050840: extracellular matrix binding
GO:0042491: auditory receptor cell differentiation
GO:0008353: RNA polymerase subunit kinase activity
GO:0005173: stem cell factor receptor binding
GO:0006904: vesicle docking during exocytosis
GO:0004587: ornithine-oxo-acid transaminase activity
GO:0034464: BBSome
GO:0044237: cellular metabolic process
GO:0004897: ciliary neurotrophic factor receptor activity
GO:0032801: receptor catabolic process
GO:0006298: mismatch repair
GO:0042255: ribosome assembly
GO:0042273: ribosomal large subunit biogenesis
GO:0006544: glycine metabolic process
GO:0045446: endothelial cell differentiation
GO:0008190: eukaryotic initiation factor 4E binding
GO:0004165: dodecenoyl-CoA delta-isomerase activity
GO:0003906: DNA-(apurinic or apyrimidinic site) lyase activity
GO:0004008: copper-exporting ATPase activity
GO:0045261: proton-transporting ATP synthase complex, catalytic core F(1)
GO:0002262: myeloid cell homeostasis
GO:0004487: methylenetetrahydrofolate dehydrogenase (NAD+) activity
GO:0045794: negative regulation of cell volume
GO:0018279: protein amino acid N-linked glycosylation via asparagine
GO:0006338: chromatin remodeling
GO:0060163: subpallium neuron fate commitment
GO:0046677: response to antibiotic
GO:0043353: enucleate erythrocyte differentiation
GO:0003684: damaged DNA binding
GO:0051208: sequestering of calcium ion
GO:0051087: chaperone binding
GO:0003887: DNA-directed DNA polymerase activity
GO:0043218: compact myelin
GO:0043033: isoamylase complex
GO:0045947: negative regulation of translational initiation
GO:0000387: spliceosomal snRNP biogenesis
GO:0047021: 15-hydroxyprostaglandin dehydrogenase (NADP+) activity
GO:0005126: hematopoietin/interferon-class (D200-domain) cytokine receptor binding
GO:0008299: isoprenoid biosynthetic process
GO:0045239: tricarboxylic acid cycle enzyme complex
GO:0020027: hemoglobin metabolic process
GO:0030534: adult behavior
GO:0002687: positive regulation of leukocyte migration
GO:0042921: glucocorticoid receptor signaling pathway
GO:0016251: general RNA polymerase II transcription factor activity
GO:0008137: NADH dehydrogenase (ubiquinone) activity
GO:0000380: alternative nuclear mRNA splicing, via spliceosome
GO:0003705: RNA polymerase II transcription factor activity, enhancer binding
GO:0065002: intracellular protein transmembrane transport
GO:0003706: ligand-regulated transcription factor activity
GO:0019789: SUMO ligase activity
GO:0019682: glyceraldehyde-3-phosphate metabolic process
GO:0004521: endoribonuclease activity
GO:0046854: phosphoinositide phosphorylation
GO:0004921: interleukin-11 receptor activity
GO:0016531: copper chaperone activity
GO:0031403: lithium ion binding
GO:0006924: activation-induced cell death of T cells
GO:0047750: cholestenol delta-isomerase activity
GO:0019209: kinase activator activity
GO:0022625: cytosolic large ribosomal subunit

GO:0032364: oxygen homeostasis
GO:0046326: positive regulation of glucose import
GO:0060052: neurofilament cytoskeleton organization
GO:0043249: erythrocyte maturation
GO:0004135: amylo-alpha-1,6-glucosidase activity
GO:0008599: protein phosphatase type 1 regulator activity
GO:0042552: myelination
GO:0001932: regulation of protein amino acid phosphorylation
GO:0016878: acid-thiol ligase activity
GO:0055010: ventricular cardiac muscle morphogenesis
GO:0002821: positive regulation of adaptive immune response
GO:0060072: large conductance calcium-activated potassium channel activity
GO:0005669: transcription factor TFIID complex
GO:0001892: embryonic placenta development
GO:0015721: bile acid and bile salt transport
GO:0005784: translocon complex
GO:0042759: long-chain fatty acid biosynthetic process
GO:0001952: regulation of cell-matrix adhesion
GO:0051920: peroxiredoxin activity
GO:0006800: oxygen and reactive oxygen species metabolic process
GO:0050221: prostaglandin-E2 9-reductase activity
GO:0004526: ribonuclease P activity
GO:0042769: DNA damage response, detection of DNA damage
GO:0043039: tRNA aminoacylation
GO:0030169: low-density lipoprotein binding
GO:0007213: muscarinic acetylcholine receptor signaling pathway
GO:0030371: translation repressor activity
GO:0042541: hemoglobin biosynthetic process
GO:0016081: synaptic vesicle docking during exocytosis
GO:0004300: enoyl-CoA hydratase activity
GO:0051318: G1 phase
GO:0004634: phosphopyruvate hydratase activity
GO:0004576: oligosaccharyl transferase activity
GO:0006228: UTP biosynthetic process
GO:0004161: dimethylallyltranstransferase activity
GO:0006301: postreplication repair
GO:0005977: glycogen metabolic process
GO:0003923: GPI-anchor transamidase activity
GO:0042719: mitochondrial intermembrane space protein transporter complex
GO:0005853: eukaryotic translation elongation factor 1 complex
GO:0006654: phosphatidic acid biosynthetic process
GO:0033081: regulation of T cell differentiation in the thymus
GO:0004064: arylesterase activity
GO:0070330: aromatase activity
GO:0042835: BRE binding
GO:0000827: inositol 1,3,4,5,6-pentakisphosphate kinase activity
GO:0008324: cation transmembrane transporter activity
GO:0004065: arylsulfatase activity
GO:0006221: pyrimidine nucleotide biosynthetic process
GO:0060088: auditory receptor cell stereocilium organization
GO:0008333: endosome to lysosome transport
GO:0006082: organic acid metabolic process
GO:0007309: oocyte axis specification
GO:0004769: steroid delta-isomerase activity
GO:0043025: cell soma
GO:0006984: ER-nuclear signaling pathway
GO:0031401: positive regulation of protein modification process
GO:0007093: mitotic cell cycle checkpoint
GO:0006309: DNA fragmentation during apoptosis
GO:0004924: oncostatin-M receptor activity
GO:0004550: nucleoside diphosphate kinase activity
GO:0004740: pyruvate dehydrogenase (acetyl-transferring) kinase activity
GO:0043162: ubiquitin-dependent protein catabolic process via the multivesicular body sorting pathway
GO:0004058: aromatic-L-amino-acid decarboxylase activity
GO:0000381: regulation of alternative nuclear mRNA splicing, via spliceosome
GO:0000015: phosphopyruvate hydratase complex
GO:0003988: acetyl-CoA C-acyltransferase activity
GO:0016254: preassembly of GPI anchor in ER membrane
GO:0000389: nuclear mRNA 3'-splice site recognition
GO:0032259: methylation
GO:0004159: dihydrouracil dehydrogenase (NAD+) activity
GO:0045738: negative regulation of DNA repair
GO:0051262: protein tetramerization
GO:0000210: NAD+ diphosphatase activity
GO:0000781: chromosome, telomeric region
GO:0006518: peptide metabolic process
GO:0004137: deoxycytidine kinase activity
GO:0006011: UDP-glucose metabolic process
GO:0033814: propanoyl-CoA C-acyltransferase activity
GO:0005072: transforming growth factor beta receptor, cytoplasmic mediator activity
GO:0006789: bilirubin conjugation
GO:0006529: asparagine biosynthetic process
GO:0031508: centromeric heterochromatin formation
GO:0031119: tRNA pseudouridine synthesis
GO:0031032: actomyosin structure organization
GO:0008121: ubiquinol-cytochrome-c reductase activity
GO:0043198: dendritic shaft
GO:0001844: protein insertion into mitochondrial membrane during induction of apoptosis
GO:0006777: Mo-molybdopterin cofactor biosynthetic process
GO:0010768: negative regulation of transcription from RNA polymerase II promoter in response to UV-induced DNA damage
GO:0030141: secretory granule
GO:0000105: histidine biosynthetic process
GO:0006120: mitochondrial electron transport, NADH to ubiquinone
GO:0031014: troponin T binding
GO:0005732: small nucleolar ribonucleoprotein complex
GO:0019226: transmission of nerve impulse
GO:0005086: ARF guanyl-nucleotide exchange factor activity
GO:0019089: transmission of virus
GO:0032236: positive regulation of calcium ion transport via store-operated calcium channel activity
GO:0000090: mitotic anaphase
GO:0004096: catalase activity
GO:0004470: malic enzyme activity
GO:0051541: elastin metabolic process
GO:0046685: response to arsenic
GO:0045926: negative regulation of growth
GO:0006467: protein thiol-disulfide exchange
GO:0060083: smooth muscle contraction involved in micturition
GO:0004691: cAMP-dependent protein kinase activity
GO:0010613: positive regulation of cardiac muscle hypertrophy
GO:0016219: GDP-dissociation stimulator activity
GO:0004715: non-membrane spanning protein tyrosine kinase activity
GO:0042809: vitamin D receptor binding
GO:0055009: atrial cardiac muscle morphogenesis
GO:0032790: ribosome disassembly
GO:0015917: aminophospholipid transport
GO:0008633: activation of pro-apoptotic gene products
GO:0019276: UDP-N-acetylgalactosamine metabolic process
GO:0043550: regulation of lipid kinase activity
GO:0032862: activation of Rho GTPase activity
GO:0000900: translation repressor activity, nucleic acid binding
GO:0008180: signalosome
GO:0070063: RNA polymerase binding
GO:0003746: translation elongation factor activity
GO:0001514: selenocysteine incorporation
GO:0033014: tetrapyrrole biosynthetic process
GO:0015321: sodium-dependent phosphate transmembrane transporter activity
GO:0006493: protein amino acid O-linked glycosylation
GO:0050847: progesterone receptor signaling pathway
GO:0031674: I band
GO:0016018: cyclosporin A binding

GO:0004784: superoxide dismutase activity
GO:0019059: initiation of viral infection
GO:0008536: Ran GTPase binding
GO:0010458: exit from mitosis
GO:0005112: Notch binding
GO:0008641: small protein activating enzyme activity
GO:0007051: spindle organization
GO:0046755: non-lytic virus budding
GO:0006710: androgen catabolic process
GO:0000049: tRNA binding
GO:0005850: eukaryotic translation initiation factor 2 complex
GO:0005138: interleukin-6 receptor binding
GO:0050819: negative regulation of coagulation
GO:0004590: orotidine-5'-phosphate decarboxylase activity
GO:0017076: purine nucleotide binding
GO:0006418: tRNA aminoacylation for protein translation
GO:0002675: positive regulation of acute inflammatory response
GO:0009249: protein lipoylation
GO:0030203: glycosaminoglycan metabolic process
GO:0060166: olfactory pit development
GO:0004598: peptidylamidoglycolate lyase activity
GO:0004647: phosphoserine phosphatase activity
GO:0007220: Notch receptor processing
GO:0047115: trans-1,2-dihydrobenzene-1,2-diol dehydrogenase activity
GO:0015991: ATP hydrolysis coupled proton transport
GO:0045453: bone resorption
GO:0006520: amino acid metabolic process
GO:0006471: protein amino acid ADP-ribosylation
GO:0033857: diphosphoinositol-pentakisphosphate kinase activity
GO:0048806: genitalia development
GO:0004915: interleukin-6 receptor activity
GO:0006106: fumarate metabolic process
GO:0004861: cyclin-dependent protein kinase inhibitor activity
GO:0051877: pigment granule aggregation in cell center
GO:0046716: muscle maintenance
GO:0004563: beta-N-acetylhexosaminidase activity
GO:0045494: photoreceptor cell maintenance
GO:0000178: exosome (RNase complex)
GO:0045931: positive regulation of mitotic cell cycle
GO:0047804: cysteine-S-conjugate beta-lyase activity
GO:0001934: positive regulation of protein amino acid phosphorylation
GO:0006434: seryl-tRNA aminoacylation
GO:0019871: sodium channel inhibitor activity
GO:0047006: 20-alpha-hydroxysteroid dehydrogenase activity
GO:0000832: inositol hexakisphosphate 5-kinase activity
GO:0019752: carboxylic acid metabolic process
GO:0000077: DNA damage checkpoint
GO:0005498: sterol carrier activity
GO:0045116: protein neddylation
GO:0016407: acetyltransferase activity
GO:0008831: dTDP-4-dehydrorhamnose reductase activity
GO:0006183: GTP biosynthetic process
GO:0009982: pseudouridine synthase activity
GO:0001757: somite specification
GO:0033989: 3alpha,7alpha,12alpha-trihydroxy-5beta-cholest-24-enoyl-CoA hydratase activity
GO:0003735: structural constituent of ribosome
GO:0019787: small conjugating protein ligase activity
GO:0000027: ribosomal large subunit assembly
GO:0006110: regulation of glycolysis
GO:0009165: nucleotide biosynthetic process
GO:0015934: large ribosomal subunit
GO:0033044: regulation of chromosome organization
GO:0001888: glucuronyl-galactosyl-proteoglycan 4-alpha-N-acetylglucosaminyltransferase activity
GO:0043024: ribosomal small subunit binding
GO:0051716: cellular response to stimulus
GO:0032020: ISG15-protein conjugation
GO:0004333: fumarate hydratase activity
GO:0006730: one-carbon compound metabolic process
GO:0004556: alpha-amylase activity
GO:0006778: porphyrin metabolic process
GO:0017119: Golgi transport complex
GO:0004190: aspartic-type endopeptidase activity
GO:0046886: positive regulation of hormone biosynthetic process
GO:0051017: actin filament bundle formation
GO:0003874: 6-pyruvoyltetrahydropterin synthase activity
GO:0004981: muscarinic acetylcholine receptor activity
GO:0031118: rRNA pseudouridine synthesis
GO:0006689: ganglioside catabolic process
GO:0034263: autophagy in response to ER overload
GO:0055007: cardiac muscle cell differentiation
GO:0003743: translation initiation factor activity
GO:0031698: beta-2 adrenergic receptor binding
GO:0008441: 3'(2'),5'-bisphosphate nucleotidase activity
GO:0008652: amino acid biosynthetic process
GO:0031575: G1/S transition checkpoint
GO:0031362: anchored to external side of plasma membrane
GO:0000279: M phase
GO:0042780: tRNA 3'-end processing
GO:0051000: positive regulation of nitric-oxide synthase activity
GO:0004843: ubiquitin-specific protease activity
GO:0016773: phosphotransferase activity, alcohol group as acceptor
GO:0006583: melanin biosynthetic process from tyrosine
GO:0005678: chromatin assembly complex
GO:0006970: response to osmotic stress
GO:0043666: regulation of phosphoprotein phosphatase activity
GO:0042787: protein ubiquitination during ubiquitin-dependent protein catabolic process
GO:0008389: coumarin 7-hydroxylase activity
GO:0004105: choline-phosphate cytidylyltransferase activity
GO:0030833: regulation of actin filament polymerization
GO:0043015: gamma-tubulin binding
GO:0043560: insulin receptor substrate binding
GO:0005751: mitochondrial respiratory chain complex IV
GO:0004581: dolichyl-phosphate beta-glucosyltransferase activity
GO:0008250: oligosaccharyltransferase complex
GO:0000276: mitochondrial proton-transporting ATP synthase complex, coupling factor F(o)
GO:0032473: external side of mitochondrial outer membrane
GO:0051246: regulation of protein metabolic process
GO:0048547: gut morphogenesis
GO:0005010: insulin-like growth factor receptor activity
GO:0015020: glucuronosyltransferase activity
GO:0004132: dCMP deaminase activity
GO:0032344: regulation of aldosterone metabolic process
GO:0043297: apical junction assembly
GO:0007184: SMAD protein nuclear translocation
GO:0005534: galactose binding
GO:0045039: protein import into mitochondrial inner membrane
GO:0005153: interleukin-8 receptor binding
GO:0016255: attachment of GPI anchor to protein
GO:0004969: histamine receptor activity
GO:0000307: cyclin-dependent protein kinase holoenzyme complex
GO:0042171: lysophosphatidic acid acyltransferase activity
GO:0007163: establishment or maintenance of cell polarity
GO:0004485: methylcrotonoyl-CoA carboxylase activity
GO:0033180: proton-transporting V-type ATPase, V1 domain
GO:0016494: C-X-C chemokine receptor activity
GO:0006928: cell motion
GO:0004970: ionotropic glutamate receptor activity
GO:0015446: arsenite transmembrane-transporting ATPase activity
GO:0002009: morphogenesis of an epithelium
GO:0004948: calcitonin receptor activity

GO:0033269: internode region of axon
GO:0019843: rRNA binding
GO:0009841: mitochondrial endopeptidase Clp complex
GO:0005246: calcium channel regulator activity
GO:0005686: snRNP U2
GO:0042765: GPI-anchor transamidase complex
GO:0008634: negative regulation of survival gene product expression
GO:0035248: alpha-1,4-N-acetylgalactosaminyltransferase activity
GO:0005068: transmembrane receptor protein tyrosine kinase adaptor protein activity
GO:0007569: cell aging
GO:0046839: phospholipid dephosphorylation
GO:0008157: protein phosphatase 1 binding
GO:0005753: mitochondrial proton-transporting ATP synthase complex
GO:0042771: DNA damage response, signal transduction by p53 class mediator resulting in induction of apoptosis
GO:0000792: heterochromatin
GO:0017101: aminoacyl-tRNA synthetase multienzyme complex
GO:0030586: [methionine synthase] reductase activity
GO:0045837: negative regulation of membrane potential
GO:0001573: ganglioside metabolic process
GO:0051028: mRNA transport
GO:0005579: membrane attack complex
GO:0006626: protein targeting to mitochondrion
GO:0008206: bile acid metabolic process
GO:0004865: protein serine/threonine phosphatase inhibitor activity
GO:0000398: nuclear mRNA splicing, via spliceosome
GO:0021537: telencephalon development
GO:0008049: male courtship behavior
GO:0060081: membrane hyperpolarization
GO:0006556: S-adenosylmethionine biosynthetic process
GO:0015319: sodium:inorganic phosphate symporter activity
GO:0006744: ubiquinone biosynthetic process
GO:0021554: optic nerve development
GO:0006400: tRNA modification
GO:0032767: copper-dependent protein binding
GO:0032467: positive regulation of cytokinesis
GO:0051536: iron-sulfur cluster binding
GO:0000386: second spliceosomal transesterification activity
GO:0006790: sulfur metabolic process
GO:0048675: axon extension
GO:0006104: succinyl-CoA metabolic process
GO:0008089: anterograde axon cargo transport
GO:0000070: mitotic sister chromatid segregation
GO:0012506: vesicle membrane
GO:0019885: antigen processing and presentation of endogenous peptide antigen via MHC class I
GO:0042587: glycogen granule
GO:0003823: antigen binding
GO:0035267: NuA4 histone acetyltransferase complex
GO:0007158: neuron adhesion
GO:0030299: cholesterol absorption
GO:0004012: phospholipid-translocating ATPase activity
GO:0007281: germ cell development
GO:0042296: ISG15 ligase activity
GO:0048270: methionine adenosyltransferase regulator activity
GO:0007400: neuroblast fate determination
GO:0051881: regulation of mitochondrial membrane potential
GO:0030001: metal ion transport
GO:0002763: positive regulation of myeloid leukocyte differentiation
GO:0000272: polysaccharide catabolic process
GO:0050816: phosphothreonine binding
GO:0009060: aerobic respiration
GO:0007157: heterophilic cell adhesion
GO:0006552: leucine catabolic process
GO:0005487: nucleocytoplasmic transporter activity
GO:0045668: negative regulation of osteoblast differentiation
GO:0045010: actin nucleation
GO:0042776: mitochondrial ATP synthesis coupled proton transport
GO:0004477: methenyltetrahydrofolate cyclohydrolase activity
GO:0051823: regulation of synapse structural plasticity
GO:0060165: regulation of timing of subpallium neuron differentiation
GO:0005900: oncostatin-M receptor complex
GO:0045651: positive regulation of macrophage differentiation
GO:0042221: response to chemical stimulus
GO:0006360: transcription from RNA polymerase I promoter
GO:0021779: oligodendrocyte cell fate commitment
GO:0035173: histone kinase activity
GO:0004066: asparagine synthase (glutamine-hydrolyzing) activity
GO:0001516: prostaglandin biosynthetic process
GO:0009396: folic acid and derivative biosynthetic process
GO:0004311: farnesyltranstransferase activity
GO:0032446: protein modification by small protein conjugation
GO:0016272: prefoldin complex
GO:0006020: inositol metabolic process
GO:0003755: peptidyl-prolyl cis-trans isomerase activity
GO:0043183: vascular endothelial growth factor receptor 1 binding
GO:0018106: peptidyl-histidine phosphorylation
GO:0005672: transcription factor TFIIA complex
GO:0045226: extracellular polysaccharide biosynthetic process
GO:0006401: RNA catabolic process
GO:0006098: pentose-phosphate shunt
GO:0045095: keratin filament
GO:0004372: glycine hydroxymethyltransferase activity
GO:0008033: tRNA processing
GO:0000184: nuclear-transcribed mRNA catabolic process, nonsense-mediated decay
GO:0007193: inhibition of adenylate cyclase activity by G-protein signaling
GO:0005680: anaphase-promoting complex
GO:0016706: oxidoreductase activity, acting on paired donors, with incorporation or reduction of molecular oxygen, 2-oxoglutarate as one donor, and incorporation of one atom each of oxygen into both donors
GO:0005663: DNA replication factor C complex
GO:0048147: negative regulation of fibroblast proliferation
GO:0033178: proton-transporting two-sector ATPase complex, catalytic domain
GO:0031323: regulation of cellular metabolic process
GO:0030628: pre-mRNA 3'-splice site binding
GO:0005947: mitochondrial alpha-ketoglutarate dehydrogenase complex
GO:0045930: negative regulation of mitotic cell cycle
GO:0008235: metalloexopeptidase activity
GO:0008047: enzyme activator activity
GO:0046966: thyroid hormone receptor binding
GO:0000120: RNA polymerase I transcription factor complex
GO:0006611: protein export from nucleus
GO:0004830: tryptophan-tRNA ligase activity
GO:0005674: transcription factor TFIIF complex
GO:0005655: nucleolar ribonuclease P complex
GO:0050790: regulation of catalytic activity
GO:0004129: cytochrome-c oxidase activity
GO:0004331: fructose-2,6-bisphosphate 2-phosphatase activity
GO:0006265: DNA topological change
GO:0060082: eye blink reflex
GO:0030218: erythrocyte differentiation
GO:0051437: positive regulation of ubiquitin-protein ligase activity during mitotic cell cycle
GO:0001919: regulation of receptor recycling
GO:0032391: photoreceptor connecting cilium
GO:0035110: leg morphogenesis
GO:0001669: acrosome
GO:0016458: gene silencing
GO:0030433: ER-associated protein catabolic process
GO:0046541: saliva secretion
GO:0004810: tRNA adenylyltransferase activity
GO:0051436: negative regulation of ubiquitin-protein ligase activity during mitotic cell cycle
GO:0004060: arylamine N-acetyltransferase activity
GO:0032402: melanosome transport
GO:0003918: DNA topoisomerase (ATP-hydrolyzing) activity

GO:0000038: very-long-chain fatty acid metabolic process
GO:0060047: heart contraction
GO:0009405: pathogenesis
GO:0048341: paraxial mesoderm formation
GO:0044445: cytosolic part
GO:0006089: lactate metabolic process
GO:0004588: orotate phosphoribosyltransferase activity
GO:0003870: 5-aminolevulinate synthase activity
GO:0004090: carbonyl reductase (NADPH) activity
GO:0032027: myosin light chain binding
GO:0015270: dihydropyridine-sensitive calcium channel activity
GO:0002248: connective tissue replacement during inflammatory response
GO:0048496: maintenance of organ identity
GO:0007206: activation of phospholipase C activity by metabotropic glutamate receptor signaling pathway
GO:0050220: prostaglandin-E synthase activity
GO:0015986: ATP synthesis coupled proton transport
GO:0006878: cellular copper ion homeostasis
GO:0003844: 1,4-alpha-glucan branching enzyme activity
GO:0005681: spliceosome
GO:0045582: positive regulation of T cell differentiation
GO:0016805: dipeptidase activity
GO:0015105: arsenite transmembrane transporter activity
GO:0004597: peptide-aspartate beta-dioxygenase activity
GO:0004063: aryldialkylphosphatase activity
GO:0016769: transferase activity, transferring nitrogenous groups
GO:0050658: RNA transport
GO:0042554: superoxide release
GO:0008593: regulation of Notch signaling pathway
GO:0010259: multicellular organismal aging
GO:0005861: troponin complex
GO:0046034: ATP metabolic process
GO:0018738: S-formylglutathione hydrolase activity
GO:0004923: leukemia inhibitory factor receptor activity
GO:0000245: spliceosome assembly
GO:0004823: leucine-tRNA ligase activity
GO:0006241: CTP biosynthetic process
GO:0021542: dentate gyrus development
GO:0042826: histone deacetylase binding
GO:0009416: response to light stimulus
GO:0004033: aldo-keto reductase activity
GO:0046824: positive regulation of nucleocytoplasmic transport
GO:0000059: protein import into nucleus, docking
GO:0006474: N-terminal protein amino acid acetylation
GO:0008080: N-acetyltransferase activity
GO:0004423: iduronate-2-sulfatase activity
GO:0032012: regulation of ARF protein signal transduction
GO:0042137: sequestering of neurotransmitter
GO:0001786: phosphatidylserine binding
GO:0005896: interleukin-6 receptor complex
GO:0001891: phagocytic cup
GO:0002026: regulation of the force of heart contraction
GO:0051044: positive regulation of membrane protein ectodomain proteolysis
GO:0015677: copper ion import
GO:0045509: interleukin-27 receptor activity
GO:0006275: regulation of DNA replication
GO:0009399: nitrogen fixation
GO:0004303: estradiol 17-beta-dehydrogenase activity
GO:0051059: NF-kappaB binding
GO:0006687: glycosphingolipid metabolic process
GO:0004618: phosphoglycerate kinase activity
GO:0040016: embryonic cleavage
GO:0007127: meiosis I
GO:0033981: D-dopachrome decarboxylase activity
GO:0045541: negative regulation of cholesterol biosynthetic process
GO:0004298: threonine-type endopeptidase activity
GO:0005031: tumor necrosis factor receptor activity
GO:0045444: fat cell differentiation
GO:0032088: negative regulation of NF-kappaB transcription factor activity
GO:0022627: cytosolic small ribosomal subunit
GO:0006449: regulation of translational termination
GO:0046619: optic placode formation involved in camera-type eye
GO:0006610: ribosomal protein import into nucleus
GO:0003689: DNA clamp loader activity
GO:0045263: proton-transporting ATP synthase complex, coupling factor F(o)
GO:0004807: triose-phosphate isomerase activity
GO:0004831: tyrosine-tRNA ligase activity
GO:0006200: ATP catabolic process
GO:0016998: cell wall catabolic process
GO:0006729: tetrahydrobiopterin biosynthetic process
GO:0000153: cytoplasmic ubiquitin ligase complex
GO:0007608: sensory perception of smell
GO:0006429: leucyl-tRNA aminoacylation
GO:0046579: positive regulation of Ras protein signal transduction
GO:0021530: spinal cord oligodendrocyte cell fate specification
GO:0006622: protein targeting to lysosome
GO:0043619: regulation of transcription from RNA polymerase II promoter in response to oxidative stress
GO:0016212: kynurenine-oxoglutarate transaminase activity
GO:0016149: translation release factor activity, codon specific
GO:0003720: telomerase activity
GO:0034465: response to carbon monoxide
GO:0007243: protein kinase cascade
GO:0005673: transcription factor TFIIE complex
GO:0015238: drug transporter activity
GO:0006436: tryptophanyl-tRNA aminoacylation
GO:0000175: 3'-5'-exoribonuclease activity
GO:0015074: DNA integration
GO:0030007: cellular potassium ion homeostasis
GO:0031307: integral to mitochondrial outer membrane
GO:0031672: A band
GO:0001819: positive regulation of cytokine production
GO:0006446: regulation of translational initiation
GO:0016198: axon choice point recognition
GO:0010001: glial cell differentiation
GO:0031175: neurite development
GO:0003995: acyl-CoA dehydrogenase activity
GO:0008038: neuron recognition
GO:0004003: ATP-dependent DNA helicase activity
GO:0042446: hormone biosynthetic process
GO:0019829: cation-transporting ATPase activity
GO:0046949: acyl-CoA biosynthetic process
GO:0003701: RNA polymerase I transcription factor activity
GO:0030529: ribonucleoprotein complex
GO:0016226: iron-sulfur cluster assembly
GO:0022010: myelination in the central nervous system
GO:0004776: succinate-CoA ligase (GDP-forming) activity
GO:0031252: cell leading edge
GO:0004428: inositol or phosphatidylinositol kinase activity
GO:0007292: female gamete generation
GO:0004998: transferrin receptor activity
GO:0030041: actin filament polymerization
GO:0006437: tyrosyl-tRNA aminoacylation
GO:0004164: diphthine synthase activity
GO:0003678: DNA helicase activity
GO:0005747: mitochondrial respiratory chain complex I
GO:0004167: dopachrome isomerase activity
GO:0006325: establishment or maintenance of chromatin architecture
GO:0016303: 1-phosphatidylinositol-3-kinase activity
GO:0016281: eukaryotic translation initiation factor 4F complex
GO:0016810: hydrolase activity, acting on carbon-nitrogen (but not peptide) bonds
GO:0030240: muscle thin filament assembly

		GO:0031202: RNA splicing factor activity, transesterification mechanism
		GO:0043022: ribosome binding
		GO:0045821: positive regulation of glycolysis
		GO:0006364: rRNA processing
		GO:0048102: autophagic cell death
		GO:0006487: protein amino acid N-linked glycosylation
		GO:0034235: GPI anchor binding
		GO:0030983: mismatched DNA binding
		GO:0007017: microtubule-based process
		GO:0048015: phosphoinositide-mediated signaling
		GO:0045730: respiratory burst
		GO:0019538: protein metabolic process
		GO:0042404: thyroid hormone catabolic process
		GO:0019048: virus-host interaction
		GO:0009628: response to abiotic stimulus
		GO:0042149: cellular response to glucose starvation
		GO:0031405: lipoic acid binding
		GO:0003883: CTP synthase activity
		GO:0042623: ATPase activity, coupled
		GO:0005697: telomerase holoenzyme complex
		GO:0003857: 3-hydroxyacyl-CoA dehydrogenase activity
		GO:0050576: 3-keto-steroid reductase activity
		GO:0016818: hydrolase activity, acting on acid anhydrides, in phosphorus-containing anhydrides
		GO:0021527: spinal cord association neuron differentiation
		GO:0006419: alanyl-tRNA aminoacylation
#1043IR	4	GO:0032060: bleb formation
		GO:0035025: positive regulation of Rho protein signal transduction
		GO:0016188: synaptic vesicle maturation
		GO:0001619: lysosphingolipid and lysophosphatidic acid receptor activity
#1051IR	39	GO:0006891: intra-Golgi vesicle-mediated transport
		GO:0045104: intermediate filament cytoskeleton organization
		GO:0005523: tropomyosin binding
		GO:0002891: positive regulation of immunoglobulin mediated immune response
		GO:0014731: spectrin-associated cytoskeleton
		GO:0006893: Golgi to plasma membrane transport
		GO:0031256: leading edge membrane
		GO:0005499: vitamin D binding
		GO:0015327: cystine:glutamate antiporter activity
		GO:0045055: regulated secretory pathway
		GO:0030507: spectrin binding
		GO:0042462: eye photoreceptor cell development
		GO:0030239: myofibril assembly
		GO:0004435: phosphoinositide phospholipase C activity
		GO:0005496: steroid binding
		GO:0000156: two-component response regulator activity
		GO:0008091: spectrin
		GO:0060084: synaptic transmission involved in micturition
		GO:0010839: negative regulation of keratinocyte proliferation
		GO:0030016: myofibril
		GO:0007352: zygotic determination of dorsal/ventral axis
		GO:0019215: intermediate filament binding
		GO:0030259: lipid glycosylation
		GO:0033005: positive regulation of mast cell activation
		GO:0016180: snRNA processing
		GO:0005577: fibrinogen complex
		GO:0016235: aggresome
		GO:0032039: integrator complex
		GO:0035095: behavioral response to nicotine
		GO:0006198: cAMP catabolic process
		GO:0030140: trans-Golgi network transport vesicle
		GO:0005024: transforming growth factor beta receptor activity
		GO:0000160: two-component signal transduction system (phosphorelay)
		GO:0060053: neurofilament cytoskeleton
		GO:0060369: positive regulation of Fc receptor mediated stimulatory signaling pathway
		GO:0046488: phosphatidylinositol metabolic process
		GO:0048812: neurite morphogenesis
		GO:0048041: focal adhesion formation
		GO:0002860: positive regulation of natural killer cell mediated cytotoxicity directed against tumor cell target
#1063IR	719	GO:0009100: glycoprotein metabolic process
		GO:0034446: substrate adhesion-dependent cell spreading
		GO:0001921: positive regulation of receptor recycling
		GO:0043067: regulation of programmed cell death
		GO:0051707: response to other organism
		GO:0008652: cellular amino acid biosynthetic process
		GO:0001702: gastrulation with mouth forming second
		GO:0070244: negative regulation of thymocyte apoptosis
		GO:0007631: feeding behavior
		GO:0002001: renin secretion into blood stream
		GO:0046111: xanthine biosynthetic process
		GO:0019229: regulation of vasoconstriction
		GO:0043259: laminin-10 complex
		GO:0044430: cytoskeletal part
		GO:0000724: double-strand break repair via homologous recombination
		GO:0035385: Roundabout signaling pathway
		GO:0008589: regulation of smoothened signaling pathway
		GO:0005451: monovalent cation:hydrogen antiporter activity
		GO:0019863: IgE binding
		GO:0019067: viral assembly, maturation, egress, and release
		GO:0042581: specific granule
		GO:0009267: cellular response to starvation
		GO:0030169: low-density lipoprotein particle binding
		GO:0001932: regulation of protein phosphorylation
		GO:0060347: heart trabecula formation
		GO:0009258: 10-formyltetrahydrofolate catabolic process
		GO:0090024: negative regulation of neutrophil chemotaxis
		GO:0060512: prostate gland morphogenesis
		GO:0042588: zymogen granule
		GO:0006270: DNA-dependent DNA replication initiation
		GO:0060421: positive regulation of heart growth
		GO:0007129: synapsis
		GO:0007249: I-kappaB kinase/NF-kappaB cascade
		GO:0015879: carnitine transport
		GO:0007263: nitric oxide mediated signal transduction
		GO:0043518: negative regulation of DNA damage response, signal transduction by p53 class mediator
		GO:0034188: apolipoprotein A-I receptor activity
		GO:0034371: chylomicron remodeling
		GO:0009743: response to carbohydrate stimulus
		GO:0042405: nuclear inclusion body
		GO:0031663: lipopolysaccharide-mediated signaling pathway
		GO:0006379: mRNA cleavage
		GO:0016064: immunoglobulin mediated immune response
		GO:0035329: hippo signaling cascade
		GO:0002314: germinal center B cell differentiation
		GO:0001946: lymphangiogenesis
		GO:0071333: cellular response to glucose stimulus
		GO:0090103: cochlea morphogenesis
		GO:0045071: negative regulation of viral genome replication
		GO:0032494: response to peptidoglycan
		GO:0050772: positive regulation of axonogenesis
		GO:0019894: kinesin binding
		GO:0000123: histone acetyltransferase complex
		GO:0021510: spinal cord development
		GO:0035602: fibroblast growth factor receptor signaling pathway involved in negative regulation of apoptosis in bone marrow
		GO:0051930: regulation of sensory perception of pain
		GO:0046950: cellular ketone body metabolic process
		GO:0015174: basic amino acid transmembrane transporter activity
		GO:0002790: peptide secretion
		GO:0022008: neurogenesis
		GO:0000299: integral to membrane of membrane fraction
		GO:0050925: negative regulation of negative chemotaxis

GO:0060527: prostate epithelial cord arborization involved in prostate glandular acinus morphogenesis
GO:0030837: negative regulation of actin filament polymerization
GO:0030676: Rac guanyl-nucleotide exchange factor activity
GO:0040017: positive regulation of locomotion
GO:0071385: cellular response to glucocorticoid stimulus
GO:0035024: negative regulation of Rho protein signal transduction
GO:0060412: ventricular septum morphogenesis
GO:0016594: glycine binding
GO:0050999: regulation of nitric-oxide synthase activity
GO:0070141: response to UV-A
GO:0009888: tissue development
GO:0042730: fibrinolysis
GO:0046696: lipopolysaccharide receptor complex
GO:0060169: negative regulation of adenosine receptor signaling pathway
GO:0007501: mesodermal cell fate specification
GO:0035235: ionotropic glutamate receptor signaling pathway
GO:0043046: DNA methylation involved in gamete generation
GO:0008331: high voltage-gated calcium channel activity
GO:0006497: protein lipidation
GO:0043129: surfactant homeostasis
GO:0021769: orbitofrontal cortex development
GO:0046697: decidualization
GO:0004123: cystathionine gamma-lyase activity
GO:0015038: glutathione disulfide oxidoreductase activity
GO:0042165: neurotransmitter binding
GO:0048562: embryonic organ morphogenesis
GO:0000062: fatty-acyl-CoA binding
GO:0034752: cytosolic aryl hydrocarbon receptor complex
GO:0033280: response to vitamin D
GO:0045130: keratan sulfotransferase activity
GO:0050727: regulation of inflammatory response
GO:0004117: calmodulin-dependent cyclic-nucleotide phosphodiesterase activity
GO:0051168: nuclear export
GO:0060501: positive regulation of epithelial cell proliferation involved in lung morphogenesis
GO:0051225: spindle assembly
GO:0060688: regulation of morphogenesis of a branching structure
GO:0033691: sialic acid binding
GO:0045103: intermediate filament-based process
GO:0005283: sodium:amino acid symporter activity
GO:0060024: rhythmic synaptic transmission
GO:0034361: very-low-density lipoprotein particle
GO:0070189: kynurenine metabolic process
GO:0001954: positive regulation of cell-matrix adhesion
GO:0001505: regulation of neurotransmitter levels
GO:0016199: axon midline choice point recognition
GO:0001931: uropod
GO:0004500: dopamine beta-monooxygenase activity
GO:0045502: dynein binding
GO:0070814: hydrogen sulfide biosynthetic process
GO:0047273: galactosylgalactosylglucosylceramide beta-D-acetylgalactosaminyltransferase activity
GO:0060687: regulation of branching involved in prostate gland morphogenesis
GO:2000124: regulation of endocannabinoid signaling pathway
GO:0008489: UDP-galactose:glucosylceramide beta-1,4-galactosyltransferase activity
GO:0042135: neurotransmitter catabolic process
GO:0046875: ephrin receptor binding
GO:0008611: ether lipid biosynthetic process
GO:0045907: positive regulation of vasoconstriction
GO:0030544: Hsp70 protein binding
GO:0046103: inosine biosynthetic process
GO:0005776: autophagic vacuole
GO:0009880: embryonic pattern specification
GO:0045187: regulation of circadian sleep/wake cycle, sleep
GO:0045190: isotype switching
GO:0051497: negative regulation of stress fiber assembly
GO:0032455: nerve growth factor processing
GO:0000981: sequence-specific DNA binding RNA polymerase II transcription factor activity
GO:0003149: membranous septum morphogenesis
GO:0001553: luteinization
GO:0051041: positive regulation of calcium-independent cell-cell adhesion
GO:0070493: thrombin receptor signaling pathway
GO:0050667: homocysteine metabolic process
GO:0010744: positive regulation of macrophage derived foam cell differentiation
GO:0042339: keratan sulfate metabolic process
GO:0032902: nerve growth factor production
GO:0004601: peroxidase activity
GO:0000722: telomere maintenance via recombination
GO:0050613: delta14-sterol reductase activity
GO:0000038: very long-chain fatty acid metabolic process
GO:0006534: cysteine metabolic process
GO:0050880: regulation of blood vessel size
GO:0022028: tangential migration from the subventricular zone to the olfactory bulb
GO:0052547: regulation of peptidase activity
GO:0045899: positive regulation of RNA polymerase II transcriptional preinitiation complex assembly
GO:0045806: negative regulation of endocytosis
GO:0071222: cellular response to lipopolysaccharide
GO:0016601: Rac protein signal transduction
GO:0003986: acetyl-CoA hydrolase activity
GO:0032436: positive regulation of proteasomal ubiquitin-dependent protein catabolic process
GO:0048406: nerve growth factor binding
GO:0031982: vesicle
GO:0030277: maintenance of gastrointestinal epithelium
GO:0005099: Ras GTPase activator activity
GO:0048185: activin binding
GO:0005976: polysaccharide metabolic process
GO:0001875: lipopolysaccharide receptor activity
GO:0060907: positive regulation of macrophage cytokine production
GO:0016585: chromatin remodeling complex
GO:0021847: ventricular zone neuroblast division
GO:0009605: response to external stimulus
GO:0048244: phytanoyl-CoA dioxygenase activity
GO:0001518: voltage-gated sodium channel complex
GO:0032507: maintenance of protein location in cell
GO:0080146: L-cysteine desulfhydrase activity
GO:0021696: cerebellar cortex morphogenesis
GO:0055010: ventricular cardiac muscle tissue morphogenesis
GO:0001741: XY body
GO:0031749: D2 dopamine receptor binding
GO:0048853: forebrain morphogenesis
GO:0060203: clathrin sculpted glutamate transport vesicle membrane
GO:0051925: regulation of calcium ion transport via voltage-gated calcium channel activity
GO:0060059: embryonic retina morphogenesis in camera-type eye
GO:0033688: regulation of osteoblast proliferation
GO:0001662: behavioral fear response
GO:0060836: lymphatic endothelial cell differentiation
GO:0032589: neuron projection membrane
GO:0034374: low-density lipoprotein particle remodeling
GO:0046101: hypoxanthine biosynthetic process
GO:0003326: pancreatic A cell fate commitment
GO:0000188: inactivation of MAPK activity
GO:0071504: cellular response to heparin
GO:0034587: piRNA metabolic process
GO:0061364: apoptosis involved in luteolysis
GO:0004602: glutathione peroxidase activity
GO:0002636: positive regulation of germinal center formation
GO:0017059: serine C-palmitoyltransferase complex
GO:0045335: phagocytic vesicle
GO:0043395: heparan sulfate proteoglycan binding
GO:0043621: protein self-association
GO:0048554: positive regulation of metalloenzyme activity
GO:0034185: apolipoprotein binding

GO:0034046: poly(G) RNA binding
GO:0051963: regulation of synaptogenesis
GO:0021520: spinal cord motor neuron cell fate specification
GO:0043522: leucine zipper domain binding
GO:0015485: cholesterol binding
GO:0007423: sensory organ development
GO:0050910: detection of mechanical stimulus involved in sensory perception of sound
GO:0003350: pulmonary myocardium development
GO:0005513: detection of calcium ion
GO:0016236: macroautophagy
GO:0006637: acyl-CoA metabolic process
GO:0034109: homotypic cell-cell adhesion
GO:0045750: positive regulation of S phase of mitotic cell cycle
GO:0016772: transferase activity, transferring phosphorus-containing groups
GO:0071672: negative regulation of smooth muscle cell chemotaxis
GO:0030857: negative regulation of epithelial cell differentiation
GO:0030240: skeletal muscle thin filament assembly
GO:0004321: fatty-acyl-CoA synthase activity
GO:0033631: cell-cell adhesion mediated by integrin
GO:0051973: positive regulation of telomerase activity
GO:0019369: arachidonic acid metabolic process
GO:0042392: sphingosine-1-phosphate phosphatase activity
GO:0006848: pyruvate transport
GO:0048013: ephrin receptor signaling pathway
GO:0043083: synaptic cleft
GO:0001964: startle response
GO:0010035: response to inorganic substance
GO:0031513: nonmotile primary cilium
GO:0048565: digestive tract development
GO:0046777: protein autophosphorylation
GO:0043616: keratinocyte proliferation
GO:0035265: organ growth
GO:0055074: calcium ion homeostasis
GO:0060595: fibroblast growth factor receptor signaling pathway involved in mammary gland specification
GO:0043257: laminin-8 complex
GO:0071229: cellular response to acid
GO:0030285: integral to synaptic vesicle membrane
GO:0032488: Cdc42 protein signal transduction
GO:0006471: protein ADP-ribosylation
GO:0005006: epidermal growth factor receptor activity
GO:0030323: respiratory tube development
GO:0005548: phospholipid transporter activity
GO:0060667: branch elongation involved in salivary gland morphogenesis
GO:0008273: calcium, potassium:sodium antiporter activity
GO:0048791: calcium ion-dependent exocytosis of neurotransmitter
GO:0004441: inositol-1,4-bisphosphate 1-phosphatase activity
GO:0061001: regulation of dendritic spine morphogenesis
GO:0019217: regulation of fatty acid metabolic process
GO:0030675: Rac GTPase activator activity
GO:0003847: 1-alkyl-2-acetylglycerophosphocholine esterase activity
GO:0048268: clathrin coat assembly
GO:0010942: positive regulation of cell death
GO:0060414: aorta smooth muscle tissue morphogenesis
GO:0035604: fibroblast growth factor receptor signaling pathway involved in positive regulation of cell proliferation in bone marrow
GO:0045987: positive regulation of smooth muscle contraction
GO:0004522: pancreatic ribonuclease activity
GO:0008046: axon guidance receptor activity
GO:0004716: receptor signaling protein tyrosine kinase activity
GO:0034436: glycoprotein transport
GO:0002042: cell migration involved in sprouting angiogenesis
GO:0051414: response to cortisol stimulus
GO:0045569: TRAIL binding
GO:0042577: lipid phosphatase activity
GO:0007519: skeletal muscle tissue development
GO:0002903: negative regulation of B cell apoptosis
GO:0043088: regulation of Cdc42 GTPase activity
GO:0033484: nitric oxide homeostasis
GO:0021860: pyramidal neuron development
GO:0021549: cerebellum development
GO:0044330: canonical Wnt receptor signaling pathway involved in positive regulation of wound healing
GO:0030510: regulation of BMP signaling pathway
GO:0031116: positive regulation of microtubule polymerization
GO:0060349: bone morphogenesis
GO:0048593: camera-type eye morphogenesis
GO:0070301: cellular response to hydrogen peroxide
GO:0045749: negative regulation of S phase of mitotic cell cycle
GO:0090072: positive regulation of sodium ion transport via voltage-gated sodium channel activity
GO:0003215: cardiac right ventricle morphogenesis
GO:0015307: drug:hydrogen antiporter activity
GO:0021631: optic nerve morphogenesis
GO:0030849: autosome
GO:0035255: ionotropic glutamate receptor binding
GO:0009072: aromatic amino acid family metabolic process
GO:0050966: detection of mechanical stimulus involved in sensory perception of pain
GO:0051297: centrosome organization
GO:0051984: positive regulation of chromosome segregation
GO:0016167: glial cell line-derived neurotrophic factor receptor activity
GO:0010827: regulation of glucose transport
GO:0030828: positive regulation of cGMP biosynthetic process
GO:0060442: branching involved in prostate gland morphogenesis
GO:0030666: endocytic vesicle membrane
GO:0030148: sphingolipid biosynthetic process
GO:0032024: positive regulation of insulin secretion
GO:0001960: negative regulation of cytokine-mediated signaling pathway
GO:0043121: neurotrophin binding
GO:0010593: negative regulation of lamellipodium assembly
GO:0048730: epidermis morphogenesis
GO:0060992: response to fungicide
GO:0032956: regulation of actin cytoskeleton organization
GO:0015227: acyl carnitine transporter activity
GO:0071230: cellular response to amino acid stimulus
GO:0060201: clathrin sculpted acetylcholine transport vesicle membrane
GO:0045576: mast cell activation
GO:0021575: hindbrain morphogenesis
GO:0005681: spliceosomal complex
GO:0032201: telomere maintenance via semi-conservative replication
GO:0010468: regulation of gene expression
GO:0008542: visual learning
GO:0005369: taurine:sodium symporter activity
GO:0005815: microtubule organizing center
GO:0001975: response to amphetamine
GO:0016290: palmitoyl-CoA hydrolase activity
GO:0071445: cellular response to protein stimulus
GO:0016444: somatic cell DNA recombination
GO:0005477: pyruvate secondary active transmembrane transporter activity
GO:0050062: long-chain-fatty-acyl-CoA reductase activity
GO:0008188: neuropeptide receptor activity
GO:0030889: negative regulation of B cell proliferation
GO:0007589: body fluid secretion
GO:0042627: chylomicron
GO:0043006: activation of phospholipase A2 activity by calcium-mediated signaling
GO:0048489: synaptic vesicle transport
GO:0034380: high-density lipoprotein particle assembly
GO:0051412: response to corticosterone stimulus
GO:0042584: chromaffin granule membrane
GO:0032367: intracellular cholesterol transport
GO:2000096: positive regulation of Wnt receptor signaling pathway, planar cell polarity pathway
GO:0021836: chemorepulsion involved in postnatal olfactory bulb interneuron migration
GO:0048169: regulation of long-term neuronal synaptic plasticity
GO:0004713: protein tyrosine kinase activity

GO:0070367: negative regulation of hepatocyte differentiation
GO:0071363: cellular response to growth factor stimulus
GO:0000979: RNA polymerase II core promoter sequence-specific DNA binding
GO:0043433: negative regulation of transcription factor activity
GO:0007346: regulation of mitotic cell cycle
GO:0017134: fibroblast growth factor binding
GO:0017156: calcium ion-dependent exocytosis
GO:0050690: regulation of defense response to virus by virus
GO:0004972: N-methyl-D-aspartate selective glutamate receptor activity
GO:0050929: induction of negative chemotaxis
GO:0016155: formyltetrahydrofolate dehydrogenase activity
GO:0017129: triglyceride binding
GO:0045861: negative regulation of proteolysis
GO:0044328: canonical Wnt receptor signaling pathway involved in positive regulation of endothelial cell migration
GO:0014063: negative regulation of serotonin secretion
GO:0050919: negative chemotaxis
GO:0021972: corticospinal neuron axon guidance through spinal cord
GO:0004502: kynurenine 3-monooxygenase activity
GO:0030345: structural constituent of tooth enamel
GO:0033993: response to lipid
GO:0045666: positive regulation of neuron differentiation
GO:0018272: protein-pyridoxal-5-phosphate linkage via peptidyl-N6-pyridoxal phosphate-L-lysine
GO:0015630: microtubule cytoskeleton
GO:0046061: dATP catabolic process
GO:0031146: SCF-dependent proteasomal ubiquitin-dependent protein catabolic process
GO:0071359: cellular response to dsRNA
GO:0002158: osteoclast proliferation
GO:0007189: activation of adenylate cyclase activity by G-protein signaling pathway
GO:0048266: behavioral response to pain
GO:0033700: phospholipid efflux
GO:0019005: SCF ubiquitin ligase complex
GO:0021891: olfactory bulb interneuron development
GO:0008344: adult locomotory behavior
GO:0010872: regulation of cholesterol esterification
GO:0051480: cytosolic calcium ion homeostasis
GO:0009720: detection of hormone stimulus
GO:0040023: establishment of nucleus localization
GO:0061003: positive regulation of dendritic spine morphogenesis
GO:0046464: acylglycerol catabolic process
GO:0005547: phosphatidylinositol-3,4,5-trisphosphate binding
GO:0043186: P granule
GO:0034041: sterol-transporting ATPase activity
GO:0045499: chemorepellent activity
GO:0060056: mammary gland involution
GO:0060915: mesenchymal cell differentiation involved in lung development
GO:0005250: A-type (transient outward) potassium channel activity
GO:0051668: localization within membrane
GO:0048488: synaptic vesicle endocytosis
GO:0042417: dopamine metabolic process
GO:0060029: convergent extension involved in organogenesis
GO:0042089: cytokine biosynthetic process
GO:0016174: NAD(P)H oxidase activity
GO:0060311: negative regulation of elastin catabolic process
GO:0010332: response to gamma radiation
GO:0042058: regulation of epidermal growth factor receptor signaling pathway
GO:0042445: hormone metabolic process
GO:0030235: nitric-oxide synthase regulator activity
GO:0035555: initiation of Roundabout signal transduction
GO:0019344: cysteine biosynthetic process
GO:0090007: regulation of mitotic anaphase
GO:0021545: cranial nerve development
GO:0000096: sulfur amino acid metabolic process
GO:0004121: cystathionine beta-lyase activity
GO:0051150: regulation of smooth muscle cell differentiation
GO:0010875: positive regulation of cholesterol efflux
GO:0060045: positive regulation of cardiac muscle cell proliferation
GO:0030595: leukocyte chemotaxis
GO:0016331: morphogenesis of embryonic epithelium
GO:0042059: negative regulation of epidermal growth factor receptor signaling pathway
GO:0030512: negative regulation of transforming growth factor beta receptor signaling pathway
GO:0000730: DNA recombinase assembly
GO:0015355: secondary active monocarboxylate transmembrane transporter activity
GO:0043274: phospholipase binding
GO:0071805: potassium ion transmembrane transport
GO:0017146: N-methyl-D-aspartate selective glutamate receptor complex
GO:0051017: actin filament bundle assembly
GO:0032410: negative regulation of transporter activity
GO:0015068: glycine amidinotransferase activity
GO:0071300: cellular response to retinoic acid
GO:0005248: voltage-gated sodium channel activity
GO:0008074: guanylate cyclase complex, soluble
GO:0050928: negative regulation of positive chemotaxis
GO:0015226: carnitine transporter activity
GO:0007140: male meiosis
GO:0014047: glutamate secretion
GO:0006975: DNA damage induced protein phosphorylation
GO:0043408: regulation of MAPKKK cascade
GO:0070976: TIR domain binding
GO:0046952: ketone body catabolic process
GO:0043292: contractile fiber
GO:0032091: negative regulation of protein binding
GO:0045920: negative regulation of exocytosis
GO:0017127: cholesterol transporter activity
GO:0008815: citrate (pro-3S)-lyase activity
GO:0010952: positive regulation of peptidase activity
GO:0007157: heterophilic cell-cell adhesion
GO:0051605: protein maturation by peptide bond cleavage
GO:0004677: DNA-dependent protein kinase activity
GO:0042063: gliogenesis
GO:0010044: response to aluminum ion
GO:0071777: positive regulation of cell cycle cytokinesis
GO:0014051: gamma-aminobutyric acid secretion
GO:0006968: cellular defense response
GO:0035108: limb morphogenesis
GO:0050544: arachidonic acid binding
GO:0043113: receptor clustering
GO:0030888: regulation of B cell proliferation
GO:0090260: negative regulation of retinal ganglion cell axon guidance
GO:0004016: adenylate cyclase activity
GO:0047372: acylglycerol lipase activity
GO:0031659: positive regulation of cyclin-dependent protein kinase activity involved in G1/S
GO:0090288: negative regulation of cellular response to growth factor stimulus
GO:0032497: detection of lipopolysaccharide
GO:0030644: cellular chloride ion homeostasis
GO:0050693: LBD domain binding
GO:0031532: actin cytoskeleton reorganization
GO:0032808: lacrimal gland development
GO:0043981: histone H4-K5 acetylation
GO:0033278: cell proliferation in midbrain
GO:0051058: negative regulation of small GTPase mediated signal transduction
GO:0017158: regulation of calcium ion-dependent exocytosis
GO:0007431: salivary gland development
GO:0048608: reproductive structure development
GO:0046619: optic placode formation involved in camera-type eye formation
GO:0010212: response to ionizing radiation
GO:0051585: negative regulation of dopamine uptake
GO:0008131: primary amine oxidase activity
GO:0019363: pyridine nucleotide biosynthetic process
GO:0007276: gamete generation
GO:0051597: response to methylmercury

GO:0005958: DNA-dependent protein kinase-DNA ligase 4 complex
GO:0003329: pancreatic PP cell fate commitment
GO:0010886: positive regulation of cholesterol storage
GO:0060039: pericardium development
GO:0055100: adiponectin binding
GO:0016600: flotillin complex
GO:0006907: pinocytosis
GO:0071547: piP-body
GO:0004710: MAP/ERK kinase kinase activity
GO:0003956: NAD(P)+-protein-arginine ADP-ribosyltransferase activity
GO:0042524: negative regulation of tyrosine phosphorylation of Stat5 protein
GO:0033327: Leydig cell differentiation
GO:0021754: facial nucleus development
GO:0015057: thrombin receptor activity
GO:0005121: Toll binding
GO:0006465: signal peptide processing
GO:0009651: response to salt stress
GO:0047961: glycine N-acyltransferase activity
GO:0015491: cation:cation antiporter activity
GO:0060449: bud elongation involved in lung branching
GO:0051622: negative regulation of norepinephrine uptake
GO:0008406: gonad development
GO:0060612: adipose tissue development
GO:0001517: N-acetylglucosamine 6-O-sulfotransferase activity
GO:0001561: fatty acid alpha-oxidation
GO:2000016: negative regulation of determination of dorsal identity
GO:0006904: vesicle docking involved in exocytosis
GO:0030122: AP-2 adaptor complex
GO:0016742: hydroxymethyl-, formyl- and related transferase activity
GO:0050902: leukocyte adhesive activation
GO:0031047: gene silencing by RNA
GO:0006261: DNA-dependent DNA replication
GO:0071456: cellular response to hypoxia
GO:0050692: DBD domain binding
GO:0070876: SOSS complex
GO:0006584: catecholamine metabolic process
GO:0035249: synaptic transmission, glutamatergic
GO:0001669: acrosomal vesicle
GO:0021679: cerebellar molecular layer development
GO:0032569: gene-specific transcription from RNA polymerase II promoter
GO:0045773: positive regulation of axon extension
GO:0010269: response to selenium ion
GO:0010721: negative regulation of cell development
GO:0006725: cellular aromatic compound metabolic process
GO:0030273: melanin-concentrating hormone receptor activity
GO:0070328: triglyceride homeostasis
GO:0007387: anterior compartment pattern formation
GO:0032314: regulation of Rac GTPase activity
GO:0030349: syntaxin-13 binding
GO:0010551: regulation of gene-specific transcription from RNA polymerase II promoter
GO:0060205: cytoplasmic membrane-bounded vesicle lumen
GO:0010887: negative regulation of cholesterol storage
GO:0030117: membrane coat
GO:0050671: positive regulation of lymphocyte proliferation
GO:0048541: Peyer's patch development
GO:0051612: negative regulation of serotonin uptake
GO:0043069: negative regulation of programmed cell death
GO:0002027: regulation of heart rate
GO:0030146: diuresis
GO:0050927: positive regulation of positive chemotaxis
GO:0034103: regulation of tissue remodeling
GO:0050775: positive regulation of dendrite morphogenesis
GO:0033563: dorsal/ventral axon guidance
GO:0010890: positive regulation of sequestering of triglyceride
GO:0080008: CUL4 RING ubiquitin ligase complex
GO:0007499: ectoderm and mesoderm interaction
GO:0060313: negative regulation of blood vessel remodeling
GO:0016830: carbon-carbon lyase activity
GO:0042986: positive regulation of amyloid precursor protein biosynthetic process
GO:0045019: negative regulation of nitric oxide biosynthetic process
GO:0043548: phosphatidylinositol 3-kinase binding
GO:0021750: vestibular nucleus development
GO:0007616: long-term memory
GO:0060849: regulation of transcription involved in lymphatic endothelial cell fate commitment
GO:0005607: laminin-2 complex
GO:0043206: fibril organization
GO:0048846: axon extension involved in axon guidance
GO:0015697: quaternary ammonium group transport
GO:0018146: keratan sulfate biosynthetic process
GO:0030193: regulation of blood coagulation
GO:0051964: negative regulation of synaptogenesis
GO:0006983: ER overload response
GO:0006790: sulfur compound metabolic process
GO:0006283: transcription-coupled nucleotide-excision repair
GO:0060615: mammary gland bud formation
GO:0003322: pancreatic A cell development
GO:0061037: negative regulation of cartilage development
GO:0032747: positive regulation of interleukin-23 production
GO:0009346: citrate lyase complex
GO:0015347: sodium-independent organic anion transmembrane transporter activity
GO:0015375: glycine:sodium symporter activity
GO:0034437: glycoprotein transporter activity
GO:0045080: positive regulation of chemokine biosynthetic process
GO:0071902: positive regulation of protein serine/threonine kinase activity
GO:0031694: alpha-2A adrenergic receptor binding
GO:0016486: peptide hormone processing
GO:0042327: positive regulation of phosphorylation
GO:0042997: negative regulation of Golgi to plasma membrane protein transport
GO:0043983: histone H4-K12 acetylation
GO:0035603: fibroblast growth factor receptor signaling pathway involved in hemopoiesis
GO:0033138: positive regulation of peptidyl-serine phosphorylation
GO:0008610: lipid biosynthetic process
GO:0008266: poly(U) RNA binding
GO:0005123: death receptor binding
GO:0033344: cholesterol efflux
GO:0045717: negative regulation of fatty acid biosynthetic process
GO:0070652: HAUS complex
GO:0001843: neural tube closure
GO:0035607: fibroblast growth factor receptor signaling pathway involved in orbitofrontal cortex development
GO:0070100: negative regulation of chemokine-mediated signaling pathway
GO:0006844: acyl carnitine transport
GO:0070858: negative regulation of bile acid biosynthetic process
GO:0043114: regulation of vascular permeability
GO:0048495: Roundabout binding
GO:0043982: histone H4-K8 acetylation
GO:0030215: semaphorin receptor binding
GO:0007128: meiotic prophase I
GO:0005030: neurotrophin receptor activity
GO:0006271: DNA strand elongation involved in DNA replication
GO:0008306: associative learning
GO:0010350: cellular response to magnesium starvation
GO:0030288: outer membrane-bounded periplasmic space
GO:0045600: positive regulation of fat cell differentiation
GO:0090136: epithelial cell-cell adhesion
GO:0007256: activation of JNKK activity
GO:0021590: cerebellum maturation
GO:0090083: regulation of inclusion body assembly
GO:0005326: neurotransmitter transporter activity
GO:0001101: response to acid
GO:0032914: positive regulation of transforming growth factor-beta1 production

GO:0004758: serine C-palmitoyltransferase activity
GO:0001950: plasma membrane enriched fraction
GO:0000790: nuclear chromatin
GO:0008184: glycogen phosphorylase activity
GO:0071425: hemopoietic stem cell proliferation
GO:0046618: drug export
GO:0030032: lamellipodium assembly
GO:0048066: developmental pigmentation
GO:0042640: anagen
GO:0044329: canonical Wnt receptor signaling pathway involved in positive regulation of cell-cell adhesion
GO:0003148: outflow tract septum morphogenesis
GO:0032261: purine nucleotide salvage
GO:0014823: response to activity
GO:0002238: response to molecule of fungal origin
GO:0007158: neuron cell-cell adhesion
GO:0010040: response to iron(II) ion
GO:0060407: negative regulation of penile erection
GO:0010642: negative regulation of platelet-derived growth factor receptor signaling pathway
GO:0051902: negative regulation of mitochondrial depolarization
GO:0044424: intracellular part
GO:0031177: phosphopantetheine binding
GO:0042582: azurophil granule
GO:0016291: acyl-CoA thioesterase activity
GO:0004708: MAP kinase kinase activity
GO:0006601: creatine biosynthetic process
GO:0060670: branching involved in embryonic placenta morphogenesis
GO:0007159: leukocyte cell-cell adhesion
GO:0010711: negative regulation of collagen catabolic process
GO:0003151: outflow tract morphogenesis
GO:0042312: regulation of vasodilation
GO:0045197: establishment or maintenance of epithelial cell apical/basal polarity
GO:0000060: protein import into nucleus, translocation
GO:0003851: 2-hydroxyacylsphingosine 1-beta-galactosyltransferase activity
GO:0070435: Shc-EGFR complex
GO:0031576: G2/M transition checkpoint
GO:0001836: release of cytochrome c from mitochondria
GO:0030977: taurine binding
GO:0071813: lipoprotein particle binding
GO:0031394: positive regulation of prostaglandin biosynthetic process
GO:0043405: regulation of MAP kinase activity
GO:0030119: AP-type membrane coat adaptor complex
GO:0043208: glycosphingolipid binding
GO:0001736: establishment of planar polarity
GO:0071407: cellular response to organic cyclic compound
GO:0030916: otic vesicle formation
GO:0008294: calcium- and calmodulin-responsive adenylate cyclase activity
GO:0061202: clathrin sculpted gamma-aminobutyric acid transport vesicle membrane
GO:0034765: regulation of ion transmembrane transport
GO:0070372: regulation of ERK1 and ERK2 cascade
GO:0032792: negative regulation of CREB transcription factor activity
GO:0001967: suckling behavior
GO:0008513: secondary active organic cation transmembrane transporter activity
GO:0032926: negative regulation of activin receptor signaling pathway
GO:0060529: squamous basal epithelial stem cell differentiation involved in prostate gland acinus development
GO:0032353: negative regulation of hormone biosynthetic process
GO:0007199: G-protein signaling, coupled to cGMP nucleotide second messenger
GO:0002906: negative regulation of mature B cell apoptosis
GO:0048015: phosphatidylinositol-mediated signaling
GO:0009314: response to radiation
GO:0060429: epithelium development
GO:0042747: circadian sleep/wake cycle, REM sleep
GO:0050702: interleukin-1 beta secretion
GO:0015949: nucleobase, nucleoside and nucleotide interconversion
GO:0071679: commissural neuron axon guidance
GO:0055009: atrial cardiac muscle tissue morphogenesis
GO:0055096: low-density lipoprotein particle mediated signaling
GO:0002689: negative regulation of leukocyte chemotaxis
GO:0010703: negative regulation of histolysis
GO:0006944: cellular membrane fusion
GO:0043620: regulation of transcription in response to stress
GO:0071676: negative regulation of mononuclear cell migration
GO:0046882: negative regulation of follicle-stimulating hormone secretion
GO:0016576: histone dephosphorylation
GO:0005152: interleukin-1 receptor antagonist activity
GO:0008260: 3-oxoacid CoA-transferase activity
GO:0048762: mesenchymal cell differentiation
GO:0048841: regulation of axon extension involved in axon guidance
GO:0010517: regulation of phospholipase activity
GO:0055106: ubiquitin-protein ligase regulator activity
GO:0009967: positive regulation of signal transduction
GO:0042094: interleukin-2 biosynthetic process
GO:0005251: delayed rectifier potassium channel activity
GO:0070830: tight junction assembly
GO:0005109: frizzled binding
GO:0070495: negative regulation of thrombin receptor signaling pathway
GO:0032740: positive regulation of interleukin-17 production
GO:0005522: profilin binding
GO:0045964: positive regulation of dopamine metabolic process
GO:0042805: actinin binding
GO:0042987: amyloid precursor protein catabolic process
GO:0050883: musculoskeletal movement, spinal reflex action
GO:0004334: fumarylacetoacetase activity
GO:0005007: fibroblast growth factor receptor activity
GO:0007243: intracellular protein kinase cascade
GO:0030282: bone mineralization
GO:0050432: catecholamine secretion
GO:0035067: negative regulation of histone acetylation
GO:0071287: cellular response to manganese ion
GO:0005657: replication fork
GO:0001554: luteolysis
GO:0030118: clathrin coat
GO:0031045: dense core granule
GO:0035413: positive regulation of catenin import into nucleus
GO:0006157: deoxyadenosine catabolic process
GO:0071396: cellular response to lipid
GO:0032584: growth cone membrane
GO:0050850: positive regulation of calcium-mediated signaling
GO:0010460: positive regulation of heart rate
GO:0043199: sulfate binding
GO:0010453: regulation of cell fate commitment
GO:0031267: small GTPase binding
GO:0007388: posterior compartment specification
GO:0006303: double-strand break repair via nonhomologous end joining
GO:0022612: gland morphogenesis
GO:0051798: positive regulation of hair follicle development
GO:0043537: negative regulation of blood vessel endothelial cell migration
GO:0001829: trophectodermal cell differentiation
GO:0032277: negative regulation of gonadotropin secretion
GO:0015651: quaternary ammonium group transmembrane transporter activity
GO:0005068: transmembrane receptor protein tyrosine kinase adaptor activity
GO:0015012: heparan sulfate proteoglycan biosynthetic process
GO:0051443: positive regulation of ubiquitin-protein ligase activity
GO:0042272: nuclear RNA export factor complex
GO:0048286: lung alveolus development
GO:0010633: negative regulation of epithelial cell migration
GO:0060601: lateral sprouting from an epithelium
GO:0002244: hemopoietic progenitor cell differentiation
GO:0060155: platelet dense granule organization
GO:0006171: cAMP biosynthetic process
GO:0015802: basic amino acid transport

GO:0019907: cyclin-dependent protein kinase activating kinase holoenzyme complex
GO:0048041: focal adhesion assembly
GO:0002088: lens development in camera-type eye
GO:0005662: DNA replication factor A complex
GO:0001780: neutrophil homeostasis
GO:0008045: motor axon guidance
GO:0050862: positive regulation of T cell receptor signaling pathway

Supplementary Table S3. Pathway analysis by Oncobox.

Pathway	.10_7xIR..1	.10_7xIR..2	.10_7xIR..3	1051_.7xIR	1051_.7xIR	1051_.7xIR	1063_7xIR.	1063_7xIR.	1063_7xIR.	1083_7xIR.	1083_7xIR.	1083_7xIR.	.1043_IR_1	.1043_IR_2	.1043_IR_3	.1095_IR_1	.1095_IR_2	.1095_IR_3	p_val	p_fdr
biocarta_role_of_mitochondria_in_apoptotic_sigr	-0,14958	0,108168	-0,22839	1,066769	0,815433	0,939422	-0,62127	-0,44727	-0,70964	-0,77647	0,443428	-1,0938	1,156836	0,660353	0,72009	-0,53909	-0,11533	-0,30738	2,08E-05	0,023558
NCI_Endogenous_TLR_signaling_Pathway_(cytoki	0,086121	-0,00234	0,018757	-1,03223	-0,96908	-1,15655	-1,6764	-1,48645	-1,32862	0,624465	1,010173	0,813516	-0,40502	-1,22289	-0,58846	0,169557	-0,33177	-0,0985	7,59E-05	0,023558
NCI_p75_NTR_mediated_signaling_Pathway_(neu	-0,27754	-0,20711	-0,20661	0,250039	0,012991	0,86211	-1,46744	-1,47759	-1,31897	-1,08826	-0,86972	-1,63209	0,533027	0,115468	0,420156	-0,81953	-1,07129	-0,81789	8,32E-05	0,023558
NCI_PAR1_mediated_thrombin_signaling_events_	0,019679	-0,04977	-0,08242	0,611616	0,307613	0,202424	-0,26641	-0,17778	-0,45536	-0,33794	-0,22197	-0,57981	0,165406	0,28673	0,214814	-0,62542	-0,34248	-0,28117	6,91E-05	0,023558
NCI_PAR1_mediated_thrombin_signaling_events_	0,019679	-0,04977	-0,08242	0,611616	0,307613	0,202424	-0,26641	-0,17778	-0,45536	-0,33794	-0,22197	-0,57981	0,165406	0,28673	0,214814	-0,62542	-0,34248	-0,28117	6,91E-05	0,023558
NCI_PAR4_mediated_thrombin_signaling_events_	0,019679	-0,04977	-0,08242	0,611616	0,307613	0,202424	-0,26641	-0,17778	-0,45536	-0,33794	-0,22197	-0,57981	0,165406	0,28673	0,214814	-0,62542	-0,34248	-0,28117	6,91E-05	0,023558
NCI_PAR4_mediated_thrombin_signaling_events_	0,019679	-0,04977	-0,08242	0,611616	0,307613	0,202424	-0,26641	-0,17778	-0,45536	-0,33794	-0,22197	-0,57981	0,165406	0,28673	0,214814	-0,62542	-0,34248	-0,28117	6,91E-05	0,023558
NCI_Validated_targets_of_C_MYC_transcriptional	-0,10294	-0,10014	-0,19995	0,133095	0,522621	0,511793	-0,68228	-0,69148	-0,95971	-0,24867	-0,08747	0,032876	0,335442	0,251899	0,490359	-0,86282	-0,39003	-0,25377	7,59E-05	0,023558
NCI_Validated_targets_of_C_MYC_transcriptional	-0,09933	-0,17385	-0,01442	0,352699	1,168318	1,186986	0,190536	0,432512	-0,08448	-0,46136	-0,64404	-1,21231	0,633951	0,55121	0,433488	-1,17807	-0,42777	-0,48906	6,18E-05	0,023558
NCI_Validated_targets_of_C_MYC_transcriptional	-0,0671	-0,07652	-0,05544	0,490381	1,157492	1,287886	-0,32805	-0,08286	-0,57644	-0,86208	-0,56088	-1,48117	0,421759	0,629012	0,530508	-1,87506	-0,67736	-0,99081	7,95E-05	0,023558
reactome_Glucuronidation_Main_Pathway	0,025845	0,460018	0,517806	-2,89741	-3,77714	-3,90748	2,564328	2,89228	3,803521	-0,48118	1,50309	2,955202	-0,85483	-2,34056	-3,63005	4,376273	5,064022	1,795472	6,00E-05	0,023558
KEGG_Drug_metabolism_cytochrome_P450_Mair	-0,13804	0,990738	0,662211	-3,11624	-3,45942	-5,35979	2,963668	2,628548	4,71111	1,749441	1,011775	4,060237	-1,19282	-3,06558	-4,31368	7,955511	6,624248	2,135931	0,000101	0,026335
KEGG_Ascorbate_and_aldarate_metabolism_Mai	-0,13352	0,330468	0,478069	-2,8865	-3,36696	-4,13266	2,755773	2,889777	4,080533	-0,05015	1,02921	2,376087	-0,91114	-2,88301	-3,94868	5,370974	5,964281	1,837368	0,000123	0,027302
reactome_CDO_in_myogenesis_Main_Pathway	-0,15813	-0,51278	-0,55066	2,206044	2,002585	1,134712	-1,14989	-1,05512	-1,7197	-1,37334	0,827326	-1,30725	0,714101	0,747423	1,266129	-0,44645	-1,06352	-0,88965	0,000117	0,027302
KEGG_Drug_metabolism_other_enzymes_Main_F	0,120452	0,52913	0,582473	-3,24965	-3,01768	-5,73682	1,848922	1,98581	3,624067	0,37784	1,507251	3,642203	-0,6024	-3,38571	-3,78879	5,163789	5,110386	0,050567	0,000159	0,033069
biocarta_lck_and_fyn_tyrosine_kinases_in_initiati	-0,13071	0,253825	0,005441	-0,29007	-0,3834	0,068962	0,091746	-0,05249	0,685173	0,962416	0,885057	0,857877	-0,37971	-0,80498	-0,4006	0,902364	0,811811	1,30082	0,000204	0,035303
KEGG_Pentose_and_glucuronate_interconversior	0,079856	0,381208	0,398672	-4,22015	-3,9356	-6,15747	3,025307	3,180203	4,339421	-0,59183	1,22191	3,601359	-1,22485	-2,78095	-4,10165	2,135802	3,110858	-1,08992	0,000192	0,035303
reactome_SMAD2_SMAD3_SMAD4_heterotrimer	0,007194	-0,19804	-0,20729	2,29621	1,396153	1,390724	0,917773	1,006106	0,29095	-2,4387	-1,01322	-0,53424	0,540811	0,599254	0,561005	-2,7353	-2,05831	-1,19866	0,000204	0,035303
D-imyoi-inositol_3456-tetrakisphosphate_biosynt	-0,08352	-0,12952	-0,10782	0,101265	0,466747	0,107686	-0,13454	-0,14139	-0,19234	-0,2983	-0,49885	-0,56025	0,067663	0,167081	-0,02826	-0,52579	-0,45567	-0,33528	0,00027	0,042085
NCI_Endogenous_TLR_signaling_Main_Pathway	-0,2083	-0,03639	-0,22704	-0,49885	-0,94217	-1,34238	-3,6623	-3,40708	-3,125	2,978254	1,761968	1,199439	-1,11914	-1,88658	-0,6393	0,993109	1,135343	1,010564	0,00027	0,042085
NCI_Alpha6_beta4_integrin_ligand_interactions_I	-0,15731	-0,1499	0,014382	-0,66008	-0,42603	-0,28668	-2,41016	-2,54263	-2,39151	1,262325	0,677398	0,772455	-0,47859	-0,40212	-0,45949	0,72908	0,651952	0,440399	0,000315	0,043668
NCI_LKB1_signaling_events_Pathway_(anoikis)	0,005985	-0,08613	-0,09867	0,595473	0,473353	0,097747	0,116104	0,081546	0,073613	-0,35051	0,051694	0,270603	0,435181	0,581987	0,577376	-0,41423	-0,79423	-0,72186	0,000322	0,043668
nicotine_degradation_IV	-0,18208	0,226515	0,075623	-0,64075	-0,74805	-0,06078	1,129096	0,610115	1,250764	1,465354	0,304847	0,622721	-0,56572	-1,35889	-0,87041	1,698355	1,00742	0,306358	0,00035	0,043668
reactome_Constitutive_Signaling_by_NOTCH1_t_	0,076846	0,029433	0,011175	0,529984	0,132957	0,246417	-0,87899	-0,79353	-1,04301	-0,20202	-0,00532	0,095628	0,49823	0,5019	0,632352	-0,68201	-0,45136	-0,20687	0,000337	0,043668
KEGG_Steroid_hormone_biosynthesis_Main_Path	-0,02577	0,197042	0,378945	-3,15392	-4,14888	-6,04125	3,168002	2,092591	5,085098	3,921736	0,568529	4,433452	-0,24727	-2,67164	-3,19856	8,062275	8,211785	2,583512	0,000368	0,044069
NCI_Regulation_of_retinoblastoma_protein_Path	-0,10954	-0,14345	-0,13561	0,043855	0,580864	-0,00318	0,644247	0,750113	0,556679	-0,83082	-0,67201	-1,00326	0,231162	0,164172	0,182089	-0,25511	-0,66023	-0,35737	0,000391	0,045106
KEGG_Notch_signaling_Main_Pathway	-0,20097	0,095694	0,005506	1,078425	1,762794	1,556184	0,34296	0,796908	0,276213	-0,64188	-1,61999	-1,48763	1,394343	1,212587	1,50736	0,609676	0,207714	1,037677	0,000418	0,046547
reactome_p75NTR_negatively_regulates_cell_cyc	-0,04891	-0,11963	-0,01519	-0,04347	0,470543	0,278272	-0,05932	-0,04684	-0,16922	-0,22044	-0,09581	-0,54784	0,659449	0,303915	0,593984	-1,08909	-0,9484	-0,78554	0,000484	0,05203
reactome_Amine_ligand_binding_receptors_Mair	-0,17773	0,123431	0,07145	-0,43871	-2,2026	-0,82668	0,537068	0,404957	0,676779	2,546742	1,754658	0,954531	-0,7921	-0,30954	-0,41963	2,077395	1,913025	1,346004	0,00051	0,052997
reactome_Eicosanoids_Main_Pathway	0,365536	0,515161	0,745429	-0,65605	-0,50885	-0,88069	-1,15745	-1,359	-0,03347	2,420064	1,826808	1,728255	0,43609	0,052085	0,643681	2,65259	2,043616	1,664627	0,000579	0,058169
biocarta_mets_affect_on_macrophage_differenti	0,036197	-0,01315	-0,13217	1,037661	0,803225	0,847009	0,268184	0,292248	-0,44865	-1,84305	-1,11256	-1,46049	0,173162	0,008597	0,364986	-2,11522	-0,98502	-0,44673	0,000633	0,058487
KEGG_Olfactory_transduction_Main_Pathway	-11,3492	6,284344	1,503138	-30,7025	-29,1757	-30,8379	58,87917	50,87613	79,40178	73,48898	26,15869	36,61434	-11,6201	-19,6467	-20,1876	92,49403	70,00201	54,87789	0,000695	0,058487
NCI_PAR4_mediated_thrombin_signaling_events_	-0,21913	-0,4352	-0,37696	0,599243	0,582132	-0,01463	-2,12487	-2,07685	-2,14323	-0,74618	0,00954	-0,97181	-0,0592	0,392387	0,703571	-1,21648	-0,52496	-0,00314	0,000694	0,058487
reactome_Olfactory_Signaling_Pathway_Main_Pa	-10,9742	5,953928	1,559817	-30,0493	-29,3336	-31,5494	56,35148	49,2017	76,91683	70,61092	27,39566	32,78875	-11,2339	-18,0166	-19,4064	88,65726	67,43098	55,12345	0,000631	0,058487
reactome_Synthesis_of_Leukotrienes_LT_and_Eo	0,328151	0,322715	1,044423	-0,80719	-1,31864	-0,6823	-0,83106	-1,15237	-0,01706	1,312519	0,696789	1,220214	0,25169	-0,26002	0,241687	1,925048	2,355245	1,025418	0,000675	0,058487
WNT_Pathway_Cytoskeletal_Rearrangement	0,028669	-0,0038	0,103743	0,583932	0,316612	0,397951	-0,37588	-0,23914	-0,43943	0,10985	-0,09449	-0,24	0,379035	0,256914	0,349731	-0,5388	-0,90552	-0,51975	0,000656	0,058487
Akt_Signaling_Pathway_Translation	0,139073	-0,03523	-0,0191	0,583866	0,370625	0,170789	-0,09719	0,05474	-0,5335	-0,21271	-0,40911	0,039746	0,301562	0,56512	0,574113	-1,24218	-0,72418	-0,51335	0,000718	0,058681
NCI_Regulation_of_retinoblastoma_protein_Path	-0,21649	-0,36956	-0,21664	-0,22505	1,023669	-0,20141	1,64917	1,726205	1,569944	-2,45084	-1,66407	-1,95802	0,599669	0,317547	0,55966	-0,87251	-1,40661	-0,92173	0,000735	0,058681
reactome_PERK_regulates_gene_expression_Mai	0,050748	0,005902	-0,02154	0,512705	0,852566	0,436077	-0,17849	-0,1351	-0,38047	-0,40515	-0,17814	-0,41943	0,188279	0,182338	0,116177	-1,11182	-0,93274	-0,55004	0,000804	0,062645
JNK_Pathway_Gene_Expression_Apoptosis_Inflan	-0,00433	-0,38992	-0,64879	2,290071	2,633478	0,724178	-1,05136	-0,83246	-1,34451	-4,78878	0,806943	-1,75191	1,764831	1,689732	3,218764	-1,21348	-2,93488	1,162334	0,000908	0,062851
KEGG_Metabolism_of_xenobiotics_by_cytochrom	-0,79504	0,756295	0,161347	-6,91693	-5,58795	-7,26504	2,730761	1,541534	5,95265	2,724735	1,525547	5,963418	-0,14277	-1,2099	-4,05333	8,515152	7,44621	1,686386	0,000862	0,062851
NCI_BCR_signaling_Pathway_(ubiquitin_depende	0,2597	-0,11881	-0,11159	-0,22924	0,551391	0,518126	-0,82616	-0,62191	-1,28819	-0,93155	-0,92756	-0,9328	0,933244	0,146505	0,427983	-1,68723	-1,52785	-1,56651	0,000894	0,062851
NCI_S1P1_Pathway_(receptor_internalization)	-0,02394	-0,22016	-0,07383	1,135508	0,7722	0,689745	-0,18266	-0,03355	-0,12493	-0,01756	-0,69974	-1,46546	0,021828	0,334005	0,296165	-1,39366	-0,5581	-0,34986	0,000828	0,062851
reactome_NRAGE_signals_death_through_JNK_M	-0,04743	-0,76402	-0,36868	0,330815	1,813705	1,080477	-2,88199	-2,92077	-3,76756	-6,16635	-1,50951	-1,72992	1,03784	2,095558	1,516638	-3,54119	-1,59117	-0,86652	0,000906	0,062851
spermine_biosynthesis	0,04948	-0,02335	-0,04708	0,114467	-0,00984	0,22599	-0,27034	-0,25137	-0,411	-0,46226	-0,12777	-0,51907	0,295483	0,156952	0,217575	-0,67296	-0,19036	-0,21378	0,000957	0,064808
1D-imyoi-inositol_hexakisphosphate_biosynthesis	-0,05598	-0,14298	-0,10449	0,106725	0,610874	0,159464	-0,35097	-0,3615	-0,49166	0,120707	-0,6478	-0,40829	0,223166	0,23516	0,000255	-0,73117	-0,68576	-0,38208	0,001053	0,067945
biocarta_granzyme_a_mediated_apoptosis_Main	-0,04572	0,153472	0,305432	0,807314	0,872632	0,307348	0,18121	0,247863	0,554305	-1,38217	-0,55537	-0,56473	0,465972	0,438177	0,517028	-1,15294	-0,13754	0,046203	0,001178	0,067945
KEGG_Neurotrophin_signaling_Main_Pathway	-0,03477	-1,68897	-1,32481	1,102899	2,25409	2,313286	-9,6683	-8,50106	-11,6057	-4,6522	-2,8921	-0,25957	1,955569	0,915712	3,304433	-10,7347	-7,31779	-4,24741	0,001133	0,067945
NCI_Regulation_of_retinoblastoma_protein_Mair	-0,09914	-0,43692	-0,49653	-0,22322	0,96223	0,316304	2,117785	2,463083	1,206526	-2,89433	-1,68661	-3,98987	0,942549	1,012485	1,148319	-5,82288	-3,80331	-2,62108	0,001057	0,067945
NCI_Regulation_of_retinoblastoma_protein_Path	-0,12005	-0,26703	-0,17133	-0,07151	0,695636	0,712599	1,533365	1,638233	1,149673	-1,45539	-0,55618	-1,98669	0,094811	0,348145	0,486296	-2,66477	-1,51943	-0,74442	0,001166	0,067945
reactome_Constitutive_Signaling_by_NOTCH1_HI	0,298436	-0,25875	-0,3051	0,126271	0,858802	0,431607	-1,52422	-1,26603	-2,41204	-4,15988	-1,31995	-3,35808	2,209234	1,378743	2,297405	-6,8966	-4,02761	-2,87834	0,001164	0,067945
reactome_Constitutive_Signaling_by_NOTCH1_PE	0,298436	-0,25875	-0,3051	0,126271	0,858802	0,431607	-1,52422	-1,26603	-2,41204	-4,15988	-1,31995	-3,35808	2,209234	1,378743	2,297405	-6,8966	-4,02761	-2,87834	0,001164	0,067945
reactome_Purine_salvage_Main_Pathway	0,201878	-0,16699	-0,14844	0,619927	0,684092	0,825656	0,517687	0,852412	0,136251	-1,46183	-0,6593	-1,414	0,413967	0,695533	0,7728	-3,37788	-2,41143	-2,1471	0,001167	0,067945
NCI_Regulation_of_cytoplasmic_and_nuclear_SM	-0,08855	-0,21052	-0,13954	0,779189	0,162475	0,416762	-0,13051	0,077696	-0,31168	-1,26013	0,224929	-0,31913	0,698156	0,912181	0,77938	-2,27264	-1,81738	-0,65942	0,001308	0,068906
NCI_Regulation_of_retinoblastoma_protein_Path	-0,2356	-0,2792	-0,24783	-0,19397	0,953893	0,502318	1,623572	1,81446	1,272483	-1,46621	-1,08214	-2,90334	0,255125	0,416766	0,529936	-1,66293	-0,979	-0,31409	0,001223	0,068906
reactome_Anchoring_fibril_formation_Main_Path	-0,12521	0,056589	-0,00343	-0,16972	-0,03197	-0,23793	-0,34059	-0,63405	0,049337	0,223533	0,408195	0,6631	-0,46774	-0,29023	-0,59576	1,32651	0,715208	0,891051	0,001245	0,068906
reactome_Constitutive_PI3K_AKT_Signaling_in_C	0,157193	-0,39356	-0,14151	0,160252	1,616971	0,437152	-0,737	-0,44209	-1,57623	-1,08606	0,581818	-1,58985	1,079349	0,886195	0,842736	-2,93566	-2,39996	-1,56985	0,001327	0,068906
reactome_NICD_traffics_to_nucleus_Main_Pathw	-0,0618	-0,14062	-0,15022	0,118538	0,34086	-0,23949	0,22223	0,296818	0,048155	-1,88116	-0,9171	-0,72768	0,582068	0,032983	0,534229	-1,88633	-1,63538	-1,08067	0,001301	0,068906
reactome_Notch_HLH_transcription_Main_Pathw	-0,0618	-0,14062	-0,15022	0,118538	0,34086	-0,23949	0,22223	0,296818	0,048155	-1,88116	-0,9171	-0,72768	0,582068	0,032983	0,534229	-1,88633	-1,63538	-1,08067	0,001301	0,068906
fatty_acid_alpha-oxidation_III	-0,06648	-0,0885	0,008094	0,220667	0,050827	-0,10399	-0,30593	-0,29298	-0,40153	-0,48725	-0,48879	-0,18898	0,2093	0,066637	0,21226	-0,67509	-0,4079	-0,12668	0,001377	0,069186
nicotine_degradation_III	-0,06118	-0,02273	0,017201	-0,63329	-0,26033	-0,10979	0,429827	0,156758	0,503847	0,338642	0,365656	0,761372	-0,15839	-0,77159	-0,42063	0,944909	1,236017	-0,01459	0,001359	0,069186
reactome_Regulation_of_Commissural_axon_pat	-0,02606	-0,01774	0,010192	0,093097	-0,23232	-0,1739	-0,64778	-0,53657	-0,6346	0,474807	0,927902	0,585954	-0,58379	-0,09774	-0,39027	0,229682	0,527525	0,456529	0,001499	0,074116
Notch_Signaling_Pathway	0,251148	0,09231	0,058026	0,576489	1,954441	0,70865	0,831535	1,291969	0,691204	0,023926	0,139553	0,114096	1,21983	1,101441	1,255149	-2,45699	-2,00916	-2,11191	0,001555	0,075686
KEGG_Retinol_metabolism_Main_Pathway	-0,85789	0,78026	0,881948	-7,03031	-5,83958	-8,57198	3,463409	2,658617	6,850481	5,470389	3,484999	7,576409	1,555333	-0,79091	-2,38318	12,55381	11,32114	5,00261	0,001677	0,08037
biocarta_akt_signaling_Main_Pathway	-0,05334	-0,08606	-0,29587	0,309295	0,351091	0,449635	1,279718	1,228955	1,14464	-0,84878	0,21499	0,616217	0,77711	0,796107	1,064824	-0,7426	-0,25754	0,339898	0,002602	0,081203
biocarta_hypoxia_and_p53_in_the_cardiovascula	0,17616	-0,29347	-0,16128	0,995311	1,833722	1,379115	-0,14587	0,090261	-0,59405	-0,2871	-0,31218	-0,7454	0,323202	0,149647	0,159592	-3,32215	-2,07477	-1,80674	0,002699	0,081203
biocarta_il_4_signaling_Pathway_(cell_proliferatio	0,034625	0,402553	0,247747	-0,63952	0,08901	0,27129	0,255358	0,254547	0,803402	1,261456	0,70245	1,314242	0,220025	-0,28737	-0,16291	0,783041	1,223914	0,459649	0,002358	0,081203
biocarta_mechanisms_of_transcriptional_repress	-0,03054	-0,12557	-0,17822	-0,19169	0,440566	0,059669	1,178278	1,27771	0,911492	-2,3437	-1,073	-1,42199	0,276079	0,205857	0,116233	-2,71955	-1,85796	-1,96015	0,00237	0,081203
biocarta_mechanisms_of_transcriptional_repress	-0,03054	-0,12557	-0,17822	-0,19169	0,440566	0,059669	1,178278	1,27771	0,911492	-2,3437	-1,073	-1,42199	0,276079	0,205857	0,116233	-2,71955	-1,85796	-1,96015	0,00237	0,081203
biocarta_opposing_roles_of_aif_in_apoptosis_and	0,027813	0,017365	0,019108	0,246402	0,259977	0,248602	0,655175	0,681736	0,581776	-0,03294	-0,16302	-0,36222	0,210812	0,002706	0,166177	-0,81443	-0,6909	-0,41914	0,002472	0,081203
biocarta_opposing_roles_of_aif_in_apoptosis_and	0,027813	0,017365	0,019108	0,246402	0,259977	0,248602	0,655175	0,681736	0,581776	-0,03294	-0,16302	-0,36222	0,210812	0,002706	0,166177	-0,81443	-0,6909	-0,41914	0,002472	0,081203
biocarta_opposing_roles_of_aif_in_apoptosis_and	0,027813	0,017365	0,019108	0,246402	0,259977	0,248602	0,655175	0,681736	0,581776	-0,03294	-0,16302	-0,36222	0,210812	0,002706	0,166177	-0,81443	-0,6909	-0,41914	0,002472	0,081203
biocarta_oxidative_stress_induced_gene_express	-0,15522	-0,23077	-0,17394	-0,11341	0,363098	0,127923	-0,46478	-0,38875	-0,64716	-1,14625	-0,9596	-0,11931	0,155118	0,155991	-0,00482	-0,87244	-0,14973	-0,37875	0,00273	0,081203
biocarta_role_of_erk5_in_neuronal_survival_Path	-0,01449	-0,08556	-0,13897	0,048024	0,94702	0,672373	-1,0873	-1,10501	-1,53173	-1,30853	0,168758	-0,40224	0,38502	0,297202	0,6071	-2,12578	-0,86895	-0,57903	0,002175	0,081203
biocarta_sumoylation_by_ranbp2_regulates_tran	0,035442	-0,28842	-0,13322	0,677021	1,041997	0,570794	-0,06135	-0,01817	-0,62608	-1,22272	-1,39426	-1,54973	-0,07555	0,447467	0,473143	-4,20285	-2,87355	-1,9393	0,001978	0,081203
IL-10_Pathway_Stability_Determination	-0,08594	-0,16094	-0,12462	-0,4609	-1,08781	-0,68747	-0,81754	-0,72131	-0,65877	0,488173	0,161437	0,453507	-0,43928	-0,66856	-0,4821	-0,51929	-0,5369	-0,16268	0,002717	0,081203
KEGG_Asthma_Main_Pathway	-0,92536	0,984838	0,750134	-1,18581	1,304749	-0,46151	0,345053	-0,32538	3,118863	3,544877	2,944382	3,417157	-1,93547	-1,0916	-2,48962	7,38825	7,646953	6,278714	0,002015	0,081203
KEGG_Chemical_carcinogenesis_Main_Pathway	-0,48611	0,482025	0,299496	-6,00179	-5,6727	-6,50256	2,258246	1,697655	4,608719	-0,48462	-0,4126	2,434626	0,352365	-1,16738	-4,35911	7,398341	7,438082	2,374476	0,002529	0,081203
KEGG_Glycosphingolipid_biosynthesis_ganglio_se	-0,05158	-0,05223	-0,14588	0,215666	-0,00895	0,587639	-2,72306	-2,56964	-2,90637	-0,55121	-0,17887	0,123933	0,366303	0,419149	1,00047	-1,58553	-1,44862	-0,65406	0,002659	0,081203
melatonin_degradation_I	-0,33032	-0,08964	-0,05181	-1,08487	0,091712	-0,75844	0,254946	0,003498	0,430858	0,472514	0,076249	1,13783	-0,72573	-0,34017	-0,28765	1,456982	1,215032	0,606843	0,002737	0,081203
methylglyoxal_degradation_I	0,006641	-0,17513	-0,0566	-0,1148	0,110834	-0,02071	0,213296	0,266281	0,16545	-0,37605	-0,10676	-0,28853	0,058353	0,262846	0,192927	-0,6134	-0,2716	-0,39179	0,001865	0,081203
NCI_Coregulation_of_Androgen_receptor_activity	-0,0603	-0,09422	-0,04527	0,11928	-0,21246	0,482637	-0,45888	-0,54222	-0,61212	-1,11735	-0,94561	-0,42526	0,149743	0,239523	0,375721	-1,78485	-1,57482	-1,10153	0,00193	0,081203
NCI_Endogenous_TLR_signaling_Pathway_(regula	0,114439	0,007704	-0,06281	-0,99746	-0,79636	-0,76134	-1,23833	-1,10726	-1,00608	0,260702	0,679227	0,309124	-0,22037	-0,87356	-0,02113	2,251522	1,854248	2,245762	0,001917	0,081203
NCI_Endogenous_TLR_signaling_Pathway_(regula	0,114439	0,007704	-0,06281	-0,99746	-0,79636	-0,76134	-1,23833	-1,10726	-1,00608	0,260702	0,679227	0,309124	-0,22037	-0,87356	-0,02113	2,251522	1,854248	2,245762	0,001917	0,081203
NCI_Ephrin_B_reverse_signaling_Main_Pathway	-0,11314	-0,04325	-0,21819	0,15293	1,494103	0,370099	-1,58519	-1,55369	-1,71124	-1,42465	-0,90702	-0,81251	0,444975	0,571204	0,834972	-0,0421	-0,19591	0,22203	0,001994	0,081203
NCI_IL5_mediated_signaling_events_Pathway_(re	-0,14444	-0,32328	-0,04696	-0,33492	0,008625	-0,40112	0,364576	0,425323	0,454352	0,870177	0,295824	0,133588	-0,69628	-0,671	-0,43069	0,248487	0,666583	0,243117	0,001872	0,081203
NCI_Neurotrophic_factor_mediated_Trk_recepto	-0,11487	-0,32096	-0,40676	-0,36682	-0,23133	0,022017	-4,09989	-3,99654	-4,51468	-1,22128	-0,61758	-1,62644	0,351863	0,507784	0,022883	-1,42663	-0,7119	-1,79564	0,001797	0,081203
NCI_Nongenotropic_Androgen_signaling_Pathwa	0,029913	-0,07291	-0,14468	0,426688	-0,00662	0,243142	-0,14317	-0,05238	-0,35013	-1,14497	-0,85622	-1,11174	0,257842	0,238061	0,407285	-2,36945	-2,53801	-1,5065	0,002633	0,081203
NCI_PAR1_mediated_thrombin_signaling_events_	-0,03368	-0,17067	-0,12251	0,290093	-0,07593	0,055936	-0,64874	-0,46627	-1,07919	-1,94782	-0,52942	-1,53765	0,527148	0,602407	0,769191	-3,04092	-1,4976	-0,76503	0,002609	0,081203
NCI_Signaling_events_mediated_by_TCPTP_Main	0,127507	-0,12052	-0,08541	0,846363	-0,48473	0,026656	-1,84365	-1,64226	-2,44069	-3,3171	-1,66148	-1,32179	0,115219	1,598488	0,614203	-3,52936	-3,53357	-2,15069	0,002712	0,081203
NCI_Signaling_events_regulated_by_Ret_tyrosine	-0,05739	0,153727	0,101735	-0,45755	-0,217	-0,00901	-1,6678	-1,88141	-1,46243	0,680061	-0,28615	0,869617	-0,11541	-0,33628	-0,37381	0,959401	0,434355	0,51162	0,002128	0,081203
NCI_Signaling_events_regulated_by_Ret_tyrosine	-0,05739	0,153727	0,101735	-0,45755	-0,217	-0,00901	-1,6678	-1,88141	-1,46243	0,680061	-0,28615	0,869617	-0,11541	-0,33628	-0,37381	0,959401	0,434355	0,51162	0,002128	0,081203
NCI_Sumoylation_by_RanBP2_regulates_transcrip	0,049241	-0,32325	-0,18699	0,97898	1,149275	0,721621	-0,69292	-0,50709	-1,06695	-1,55076	-1,7143	-1,60274	-0,08816	0,666769	0,642648	-5,00405	-3,17105	-2,00091	0,001749	0,081203
NGF_Pathway_Actin_Polymerization_Neurite_Ou	0,042626	-0,14272	-0,23564	0,323126	0,486401	0,243973	-0,01568	0,03754	-0,21091	-0,43728	-0,88855	-0,26278	-0,08017	0,289952	0,008113	-1,26316	-0,934	-0,04972	0,002251	0,081203
Notch_Signaling_Pathway_Gene_Expression_Chro	0,137521	0,108204	0,071085	0,566124	1,531634	0,724284	1,214535	1,564542	1,322961	0,169469	0,072171	0,094197	0,697746	0,355062	0,60213	-1,00937	-1,75603	-1,35828	0,002711	0,081203
Notch_Signaling_Pathway_Transcription_of_Targ	0,137521	0,108204	0,071085	0,566124	1,531634	0,724284	1,214535	1,564542	1,322961	0,169469	0,072171	0,094197	0,697746	0,355062	0,60213	-1,00937	-1,75603	-1,35828	0,002711	0,081203
reactome_Activation_of_NOXA_and_translocatio	-0,02983	-0,11984	-0,10647	0,317031	0,324093	-0,15977	0,742677	0,735594	0,66342	-0,12817	0,01802	-0,3941	0,332458	0,066539	0,293166	-0,24113	-0,45857	-0,49227	0,002404	0,081203
reactome_Activation_of_PUMA_and_translocatio	-0,02983	-0,11984	-0,10647	0,317031	0,324093	-0,15977	0,742677	0,735594	0,66342	-0,12817	0,01802	-0,3941	0,332458	0,066539	0,293166	-0,24113	-0,45857	-0,49227	0,002404	0,081203
reactome_CaMK_IV_mediated_phosphorylation_	0,034595	-0,06736	-0,04585	0,226603	0,111048	0,269397	0,057308	0,051778	-0,08965	-1,11886	-0,45917	-0,4845	0,026748	-0,08834	-0,00401	-0,86819	-0,64394	-0,5757	0,002399	0,081203
reactome_G0_and_Early_G1_Main_Pathway	0,192187	-0,15086	-0,161	1,18653	1,433092	0,06572	2,838384	3,141435	2,116802	-1,70443	-0,77511	-2,02123	0,127465	0,415291	0,485978	-4,25404	-3,45457	-2,58284	0,002237	0,081203
reactome_Metabolism_of_Angiotensinogen_to_A	-0,50788	-0,00558	-0,24366	-1,52273	-1,47465	-0,06621	0,228588	-0,05919	1,25462	2,226922	1,260365	1,881896	-0,19178	-0,40264	-0,0223	2,881621	2,515343	2,231536	0,002228	0,081203
reactome_Multifunctional_anion_exchangers_Ma	-0,01461	-0,18217	-0,07586	-1,51667	-0,41282	-1,43484	-0,76649	-0,42688	-0,68511	3,252192	0,621878	0,780823	-0,7738	-0,52552	-0,8069	0,624024	1,03096	0,155606	0,001767	0,081203
reactome_PLC_beta_mediated_events_Main_Pat	0,282639	-0,14341	-0,13262	0,984622	1,842214	0,459844	0,007973	0,136243	-0,15701	-0,05965	0,637425	-0,31582	0,796171	0,706077	0,884993	-0,52617	-0,67273	0,35075	0,001801	0,081203
Regulation_of_Cytoskeleton_Remodeling_by_Act	0,040356	0,15441	-0,17124	0,408972	0,728413	0,693667	-1,30805	-0,68508	-2,19859	-2,97383	-1,26011	-2,76706	0,27257	0,417598	0,486318	-3,39871	-2,20052	-0,989	0,002221	0,081203
NCI_Class_I_PI3K_signaling_events_mediated_b	0,124782	0,074642	0,041454	0,776814	1,106506	0,220771	-1,38919	-1,20283	-1,88045	-0,69173	-0,43629	-0,13272	0,119288	0,337412	0,303082	-1,76055	-0,78325	-0,47648	0,002829	0,081308
NCI_p75_NTR_mediated_signaling_Pathway_(cell	0,034375	-0,10632	-0,01919	1,511664	0,771288	0,712369	-1,14214	-1,0956	-1,34372	-0,50793	0,452991	-0,55369	0,298692	0,157085	0,259083	-1,68948	-1,11554	-1,02811	0,002897	0,081308
reactome_Interleukin_2_signaling_Main_Pathway	-0,21031	0,22216	0,160367	-1,23468	0,09779	-1,42831	0,222791	-0,37618	0,460624	1,822389	0,752917	1,282398	-0,18683	-0,69845	-0,27337	0,871486	0,295853	0,396085	0,002841	0,081308
reactome_NGF_processing_Main_Pathway	-0,16621	-0,23912	-0,01481	-0,5779	-0,3693	-0,41932	-0,68978	-0,86825	-0,63888	1,045006	0,225021	0,850902	-0,17669	-0,04446	-0,00725	0,938266	0,71957	0,702815	0,002795	0,081308
reactome_Plasmalogen_biosynthesis_Main_Path	0,164855	-0,04833	-0,01841	0,499465	0,403955	0,516314	-1,23467	-1,05925	-1,51991	-0,66487	-0,20651	-0,835	0,028041	0,215781	0,10157	-1,82289	-1,20858	-0,84132	0,002872	0,081308
reactome_SOS_mediated_signalling_Main_Pathw	-0,06919	-0,16539	-0,15149	0,228839	0,110631	-0,21716	-0,53992	-0,57281	-0,77118	-0,47192	-0,19554	-1,23045	-0,07708	0,28139	0,140775	-1,17578	-0,90892	-1,00482	0,002851	0,081308
NCI_Plasma_membrane_estrogen_receptor_sign	0,03981	-0,23077	-0,24939	0,409578	1,407064	0,888186	-0,40935	-0,45065	-0,56691	-1,12626	-0,33571	-1,09283	-0,10393	0,076133	-0,08964	-2,59999	-2,34693	-1,37253	0,002951	0,081346
NCI_Plasma_membrane_estrogen_receptor_sign	0,03981	-0,23077	-0,24939	0,409578	1,407064	0,888186	-0,40935	-0,45065	-0,56691	-1,12626	-0,33571	-1,09283	-0,10393	0,076133	-0,08964	-2,59999	-2,34693	-1,37253	0,002951	0,081346
NCI_S1P1_Main_Pathway	-0,02005	-0,23685	-0,1077	0,906732	0,655889	0,419407	-0,43731	-0,24164	-0,36069	0,238526	-0,35961	-1,64299	-0,08613	0,216708	0,327804	-1,93713	-1,37266	-0,8148	0,003078	0,084115
reactome_Linoleic_acid_LA_metabolism_Main_Pa	0,021355	-0,04786	-0,04559	-0,65337	-0,36228	-0,61139	0,252845	0,228589	0,000738	1,597947	0,960794	0,835714	-0,59897	-0,53073	-0,52997	-0,56841	0,147337	0,344944	0,003127	0,084714
reactome_Transport_of_organic_anions_Main_Pa	-0,11214	0,486867	0,434901	0,171633	-0,56148	-1,83683	-0,52541	-0,49783	0,065711	1,740421	0,341735	-0,28032	-0,61879	-0,57438	-1,64367	1,787401	1,802567	0,108966	0,003165	0,084979
reactome_Role_of_second_messengers_in_netrir	-0,10521	-0,23628	-0,16588	0,021297	-0,46634	-0,01575	-1,44021	-1,31418	-1,20117	-1,6255	-1,72624	-2,26509	0,253259	-0,11507	-0,01865	-2,27169	-1,76511	-2,48605	0,003256	0,086683
NCI_p38_MAPK_signaling_Main_Pathway	0,114179	-0,07954	-0,07828	1,084542	0,935193	0,296963	-0,6394	-0,26754	-1,26681	-1,66386	-0,51446	-1,19279	-0,05149	0,007869	-0,28334	-2,01846	-1,97821	-1,34132	0,0033	0,087119
NCI_Netrin_mediated_signaling_events_Pathway	-0,30656	-0,41224	-0,27616	-0,75256	0,079465	-0,30833	-2,051	-1,98966	-2,09781	-1,54093	-0,79714	-0,97457	0,360789	0,151922	0,091557	-1,5204	-1,13836	-1,13353	0,003358	0,087895
NCI_LKB1_signaling_events_Pathway_(Pathway_r	0,04113	-0,11998	-0,09654	0,636191	0,476933	-0,03471	-0,43211	-0,31267	-0,53211	-3,05836	-0,79641	-1,20277	0,289718	0,482856	0,155393	-2,46602	-2,52965	-0,96668	0,003488	0,090319
NCI_PAR1_mediated_thrombin_signaling_events_	-0,28754	-0,76407	-0,55811	0,531613	0,985669	0,085147	-3,87892	-3,52971	-4,02982	-2,39648	0,025107	-1,65994	0,279277	0,45231	1,483285	-2,14571	-0,48487	0,823022	0,003619	0,090319
reactome_ABC_family_proteins_mediated_transp	-0,1613	0,341882	0,294281	-1,27249	-0,11147	-0,73757	0,714656	0,566819	1,194844	2,342973	1,293563	1,091951	0,442688	0,192458	-0,29343	3,290766	2,034527	2,54984	0,003616	0,090319
reactome_Kinesins_Main_Pathway	0,185416	-0,24208	-0,29476	1,160384	0,596642	-0,11045	0,39491	0,74351	-0,46499	-2,33285	-4,45338	-4,01363	-0,46757	-0,01901	0,203391	-4,70447	-3,16549	-1,8175	0,003624	0,090319
reactome_LRR_FLII_interacting_protein_1_LRRFII	0,048853	-0,05635	-0,0684	-0,08546	0,239798	0,185501	-0,8095	-0,77691	-0,96842	-0,94568	-0,11804	-0,58652	0,208702	0,232896	0,358744	-0,28392	-0,20882	-0,05254	0,00355	0,090319
Tight_Junctions	-0,45676	-0,05959	-0,22023	-0,54037	-2,44549	-2,34767	-3,83851	-4,43529	-2,84513	0,788183	-0,06026	-0,51839	-0,47189	-0,92495	-0,96088	1,735229	1,400901	1,480309	0,003533	0,090319
IL-2_Pathway_IL2_Gene_Expression_via_POLR1E_	-1,08591	-1,09458	-1,28446	-1,26131	0,088638	-1,46782	-4,62712	-3,89585	-5,75359	-1,49094	-2,22933	-3,16097	0,937787	0,373534	1,148767	-6,20952	-6,56523	-4,87056	0,003829	0,091047
reactome_Constitutive_Signaling_by_NOTCH1_HI	0,208887	0,089149	0,038767	0,296627	0,686674	0,434293	-0,11942	-0,06169	-0,15097	-1,5664	0,518935	-0,71481	1,005318	0,680146	0,813169	-2,58177	-1,85462	-1,1592	0,003749	0,091047
reactome_Formation_of_Senescence_Associated	0,25044	-0,19168	-0,17694	0,305297	0,23937	0,153014	-1,39011	-1,12626	-1,53082	-1,64258	-0,56005	-0,01179	0,559631	-0,14721	0,659699	-1,48435	-1,01963	-0,63108	0,003785	0,091047
reactome_Miscellaneous_substrates_Main_Pathw	0,017428	0,353627	0,556065	-0,08406	-0,07702	-0,56761	-0,67705	-0,87782	0,385096	1,198278	0,016849	0,865471	0,357676	-0,13954	0,206959	1,492806	1,682114	1,050923	0,003813	0,091047
reactome_Thyroxine_biosynthesis_Main_Pathway	-0,02683	0,144091	-0,02859	-1,03104	0,117469	-0,06248	-0,08533	-0,15443	0,573787	0,520208	0,395925	0,333536	-0,60201	-0,38138	-0,13007	1,252365	1,807767	1,242716	0,00374	0,091047

reactome_VEGF_ligand_receptor_interactions_M	-0,13926	-0,00881	-0,0362	0,066174	0,375263	-0,01318	-0,26704	-0,46124	-0,19967	0,012583	-1,24582	-0,6436	0,209331	0,365398	0,229644	-0,22071	-0,36741	-0,39529	0,003703	0,091047
NCI_LPA_receptor_mediated_events_Pathway_(s	-0,10951	0,079899	-0,00981	-0,03315	-0,04512	-0,67212	-0,18503	-0,28834	-0,08706	0,838569	0,540999	0,245306	-0,34488	-0,8891	-0,67678	-0,28011	0,378436	0,519508	0,00393	0,092747
NCI_GMCSF_mediated_signaling_events_Main_Pa	-0,08814	-0,10661	-0,01249	-0,41792	-0,19793	-0,16701	0,228259	0,22667	0,243142	0,62259	-0,08379	0,373506	-0,09974	-0,37169	-0,18296	0,081252	0,044137	0,048959	0,004143	0,092975
NCI_IL2_signaling_events_mediated_by_STAT5_N	-0,08814	-0,10661	-0,01249	-0,41792	-0,19793	-0,16701	0,228259	0,22667	0,243142	0,62259	-0,08379	0,373506	-0,09974	-0,37169	-0,18296	0,081252	0,044137	0,048959	0,004143	0,092975
NCI_IL2_signaling_events_mediated_by_STAT5_P	-0,08814	-0,10661	-0,01249	-0,41792	-0,19793	-0,16701	0,228259	0,22667	0,243142	0,62259	-0,08379	0,373506	-0,09974	-0,37169	-0,18296	0,081252	0,044137	0,048959	0,004143	0,092975
NCI_IL2_signaling_events_mediated_by_STAT5_P	-0,08814	-0,10661	-0,01249	-0,41792	-0,19793	-0,16701	0,228259	0,22667	0,243142	0,62259	-0,08379	0,373506	-0,09974	-0,37169	-0,18296	0,081252	0,044137	0,048959	0,004143	0,092975
reactome_FMO_oxidizes_nucleophiles_Main_Pat	-0,04646	0,16072	0,175666	-0,55705	-1,07223	-0,33686	0,070366	0,022251	0,125835	1,009552	0,329642	-0,82811	-0,34366	-0,60742	-0,42534	0,14832	0,066744	0,040707	0,004026	0,092975
reactome_Transport_of_Mature_mRNA_Derived_	0,490446	-0,00903	0,080245	2,179989	2,397256	1,487998	2,119921	3,004093	0,385801	-3,34421	-3,3053	-3,29252	0,575909	-0,03505	0,38018	-10,4717	-7,14652	-5,73848	0,004179	0,092975
reactome_Transport_of_Ribonucleoproteins_into	0,324778	-0,15474	0,058049	2,355741	2,174812	1,482774	1,60423	2,29254	0,104237	-4,28068	-2,35064	-2,12757	0,053592	0,124026	0,310148	-9,36847	-6,75906	-5,15377	0,004157	0,092975
sphingosine_and_sphingosine-1-phosphate_meta	0,051428	-0,03852	0,34413	-0,77395	-1,4062	-0,9134	-0,4752	-0,58782	-0,60528	1,771181	0,439893	0,336867	-0,22632	0,109721	-0,12432	0,605361	0,226244	1,102068	0,004036	0,092975
reactome_DSCAM_interactions_Main_Pathway	0,144341	-0,34217	-0,18591	0,300706	0,167991	0,043606	-0,63227	-0,50028	-0,8642	-1,32348	-0,87269	-2,2196	0,402938	0,166501	0,25003	-0,60345	-0,52016	-0,37295	0,004256	0,094029
biocarta_role_of_mef2d_in_t_cell_apoptosis_Mai	0,516815	0,157868	-0,0491	0,155656	0,407384	0,336715	0,414888	1,113465	-0,50472	-1,33395	-1,47793	-0,67512	0,059042	0,707609	0,336744	-2,39536	-1,82522	-1,95926	0,004426	0,094596
NCI_Glucocorticoid_receptor_regulatory_networl	-0,03704	-0,31682	-0,24697	0,598805	0,89566	0,908903	-0,29445	-0,382	-0,47378	-0,28131	-0,19225	0,013492	-0,3082	0,444586	0,353552	-2,52176	-1,74886	-1,31697	0,004507	0,094596
NCI_Glucocorticoid_receptor_regulatory_networl	-0,03704	-0,31682	-0,24697	0,598805	0,89566	0,908903	-0,29445	-0,382	-0,47378	-0,28131	-0,19225	0,013492	-0,3082	0,444586	0,353552	-2,52176	-1,74886	-1,31697	0,004507	0,094596
NCI_Glucocorticoid_receptor_regulatory_networl	-0,03704	-0,31682	-0,24697	0,598805	0,89566	0,908903	-0,29445	-0,382	-0,47378	-0,28131	-0,19225	0,013492	-0,3082	0,444586	0,353552	-2,52176	-1,74886	-1,31697	0,004507	0,094596
NCI_HIF_1_alpha_transcription_factor_network_I	-0,10807	-0,175	-0,25129	-0,03782	0,023566	0,3025	-0,63148	-0,5289	-0,9656	-0,35288	-0,47869	-1,05672	0,194947	0,482171	0,288967	-2,61395	-1,44488	-1,16972	0,004449	0,094596
reactome_NEP_NS2_Interacts_with_the_Cellular_	0,357428	-0,11582	0,094112	2,034069	2,035807	1,38902	1,609345	2,371502	0,164926	-4,33712	-2,83032	-2,73396	-0,10621	0,02481	0,217382	-9,53054	-6,75931	-5,08554	0,004378	0,094596
reactome_Regulation_of_Glucokinase_by_Glucok	0,201387	-0,2758	-0,01022	1,65132	1,8253	1,235129	1,688319	2,105725	0,417305	-3,59928	-2,28377	-2,09025	-0,21869	-0,3149	0,243426	-8,12196	-5,63879	-4,47365	0,004528	0,094596
reactome_Scavenging_of_heme_from_plasma_M	-0,23702	0,564066	-0,08002	-0,54336	-0,10406	0,185482	0,091028	-0,47946	1,041319	2,036783	1,976368	2,067233	0,423617	0,246351	-0,07253	2,618769	1,596414	2,695202	0,004359	0,094596
spermidine_biosynthesis	0,128669	-0,04937	-0,05696	0,388103	-0,05463	0,315111	-0,15191	-0,33632	-0,33774	-0,37013	-0,12475	-0,06803	0,434467	0,135008	0,133983	-0,59469	-0,09674	-0,01395	0,004555	0,094596
reactome_Transcriptional_regulation_of_white_a	0,057783	-0,35339	-0,24365	-1,10426	0,252386	-0,16563	-1,03228	0,525359	-2,86052	-2,15163	-4,26476	-3,73671	1,420176	0,837294	2,39213	-9,1637	-6,33213	-5,27591	0,004588	0,094637
biocarta_mapkinase_signaling_Main_Pathway	0,15214	-0,49961	-0,50227	-0,49057	-0,34076	-0,24517	-2,83486	-2,0932	-5,04139	-5,45509	-1,05352	-1,69712	1,922634	1,390108	2,343973	-5,15072	-2,94417	-2,00084	0,004736	0,095054
KEGG_Base_excision_repair_Main_Pathway	0,329281	0,068826	0,02713	-0,24994	1,127527	1,523839	3,276761	3,205608	2,094358	-4,47274	-0,71899	-2,054	0,371882	0,690497	0,833234	-5,75229	-4,66845	-2,94386	0,004878	0,095054
NCI_Class_I_PI3K_signaling_events_mediated_by_	0,086403	-0,04604	-0,09364	0,281468	1,218478	0,217666	-1,40161	-1,29057	-1,97267	-1,05161	-0,05747	0,463119	0,552525	0,712717	0,602111	-2,39242	-1,18843	-0,64496	0,004829	0,095054
NCI_PAR1_mediated_thrombin_signaling_events_	-0,09951	-0,26593	-0,20777	-0,09937	-0,01415	-0,18369	-0,84644	-0,64785	-1,20292	-1,90953	-0,48334	-1,35542	0,310586	0,547092	0,519359	-3,0816	-1,55866	-0,81713	0,004824	0,095054
NCI_PLK1_signaling_events_Pathway_(spindle_sta	0,18571	-0,02813	0,124284	0,139469	-0,02635	0,007185	1,669825	1,835299	1,093846	-1,27052	-1,48674	-1,6017	-0,00744	0,330194	0,145408	-2,01232	-1,67395	-1,14926	0,004806	0,095054
reactome_Adherens_junctions_interactions_Mair	-0,5524	-0,09794	-0,00472	0,137396	0,169693	-1,3007	-1,88874	-2,09796	-1,46515	1,906433	0,866096	2,503772	-1,22192	-0,4142	-0,24151	2,048107	2,178479	0,874538	0,004871	0,095054
reactome_Attachment_of_GPI_anchor_to_uPAR_	-0,07794	-0,10751	-0,19547	-0,0721	0,971666	0,024763	0,072112	0,202164	-0,17536	-1,99824	0,055405	-0,81987	0,048293	0,3402	0,370832	-1,63216	-0,60837	-0,97512	0,004711	0,095054
reactome_Nuclear_Pore_Complex_NPC_Disassem	0,38447	-0,11808	0,021443	2,194075	2,402817	1,82337	0,701465	1,567839	-0,83611	-2,89059	-2,89662	-2,81126	-0,4103	-0,2046	0,179398	-9,30201	-7,05057	-5,44757	0,00478	0,095054
reactome_Recycling_of_bile_acids_and_salts_Ma	-0,39844	0,51143	0,361644	-1,2493	-0,51499	-0,80277	0,84153	0,69967	1,581011	2,737803	-0,76917	-0,39082	-0,4035	-0,85866	-1,38967	2,070843	2,843971	0,976419	0,004882	0,095054
biocarta_toll_like_receptor_Pathway_(Pathway_p	0,178805	0,021698	-0,03059	0,543159	0,034792	0,068476	-0,26047	0,040941	-0,49276	0,026016	-0,16382	-0,83672	0,270438	0,192011	0,215946	-0,46243	-0,47325	-0,22804	0,005008	0,0951
JAK-STAT_Pathway_Gene_Expression_via_MYC	0,03087	-0,12358	-0,04009	0,223182	0,903241	0,610157	0,51851	0,630217	0,387103	-0,42112	0,030341	-0,45533	-0,07591	0,11302	0,261507	-1,21189	-0,55419	-0,28237	0,005029	0,0951
KEGG_Hematopoietic_cell_lineage_Main_Pathwa	-1,1086	0,040489	0,602951	-0,42635	-2,03481	0,414509	-4,83681	-7,21508	0,563654	10,38103	1,611251	4,646752	-1,23867	-1,99177	-3,45328	11,46969	5,610588	4,720999	0,004943	0,0951
reactome_Lysosphingolipid_and_LPA_receptors_I	-0,34443	-0,31834	-0,09553	-0,2951	-0,3529	-0,17476	-0,03783	-0,22168	0,799437	0,310898	0,54334	1,250453	-0,16893	-1,29833	-0,47817	1,306917	1,954756	1,091384	0,004995	0,0951
reactome_NOSTRIN_mediated_eNOS_trafficking_	-0,18101	-0,19608	-0,1271	-0,7889	-0,38788	-0,27306	-1,3252	-1,35346	-1,41331	-0,18015	0,032938	0,078715	-0,38028	-0,32964	-0,68008	0,167487	-0,49596	0,112019	0,005037	0,0951
NCI_S1P3_Pathway_(Sinus_Bradycardia)	0,030714	-0,2095	-0,15502	0,607412	0,19122	0,678142	-0,0742	-0,09584	-0,14028	-0,07037	0,056787	-0,34286	0,213752	0,003448	0,342211	-1,56683	-1,7576	-1,10071	0,005115	0,095985
biocarta_deregulation_of_cdk5_in_alzheimers_di	-0,07859	-0,06435	-0,00595	0,868257	0,262168	0,847853	0,112227	0,244696	0,138115	-0,20997	-0,03135	-0,51982	0,189812	0,239974	0,282114	-0,27365	0,226516	-0,07718	0,005296	0,095987
biocarta_deregulation_of_cdk5_in_alzheimers_di	-0,07859	-0,06435	-0,00595	0,868257	0,262168	0,847853	0,112227	0,244696	0,138115	-0,20997	-0,03135	-0,51982	0,189812	0,239974	0,282114	-0,27365	0,226516	-0,07718	0,005296	0,095987
folate_polyglutamylation	0,073292	0,01128	-0,05645	-0,0763	0,446297	-0,28675	-0,04876	0,002001	-0,08448	-0,74946	-0,26734	-0,67669	0,227193	0,134056	0,391404	-0,82501	-0,60342	-0,6797	0,005192	0,095987
NCI_PLK1_signaling_events_Pathway_(cytokinesis	0,130756	0,007083	0,062405	0,223424	-0,25314	0,068334	1,230545	1,483512	0,781478	-1,02899	-1,09172	-1,46318	0,026512	0,325603	0,21573	-2,06105	-1,83567	-1,11828	0,005348	0,095987
reactome_Erythrocytes_take_up_carbon_dioxide	-0,0874	0,208032	0,120002	-0,96361	-0,25502	-0,52759	0,284059	-0,24415	0,860653	0,597152	1,221352	1,930668	0,204114	0,250087	0,408401	2,106673	1,592503	2,253565	0,005362	0,095987
reactome_Erythrocytes_take_up_oxygen_and_re	-0,0874	0,208032	0,120002	-0,96361	-0,25502	-0,52759	0,284059	-0,24415	0,860653	0,597152	1,221352	1,930668	0,204114	0,250087	0,408401	2,106673	1,592503	2,253565	0,005362	0,095987
reactome_G2_Phase_Main_Pathway	-0,08932	-0,26048	-0,10224	-0,16628	0,159573	-0,703	0,390348	0,552102	0,401655	-1,85906	-1,72072	-1,77526	0,243851	0,265056	0,021071	-1,02565	-1,13714	-0,82597	0,005332	0,095987
TGF-Beta_Pathway	-3,21271	-2,15567	-0,08388	-10,1033	-5,07797	-7,18603	-7,28975	-10,0646	-3,33012	7,993852	1,42469	2,679687	0,218988	-1,95174	-1,28255	7,376712	7,706526	8,720771	0,005208	0,095987
NCI_amb2_Integrin_signaling_Pathway_(positive_	-0,00687	-0,23085	-0,1408	0,593109	-0,14595	0,023577	-1,02663	-1,12979	-0,65482	-1,58094	-0,22141	-0,48169	0,501445	0,23052	0,54208	-0,16653	-0,54748	-0,09254	0,005418	0,0961
reactome_Transport_of_the_SLBP_independent_	0,430141	-0,04215	0,041511	2,043889	2,03221	1,163618	1,868945	2,658302	0,32675	-3,42126	-2,73476	-2,88551	0,357971	-0,38361	0,101035	-9,63286	-6,58762	-5,22115	0,00543	0,0961
ATM_Pathway_Repair_and_Recombination	0,042693	-0,00306	-0,04541	0,017551	0,507643	0,492437	0,229529	0,355623	-0,06456	-0,08199	-0,54418	-0,46827	-0,03087	0,177098	0,1065	-0,60731	-0,16759	-0,10793	0,005614	0,0972
KEGG_Taurine_and_hypotaurine_metabolism_Ma	0,144846	0,119804	0,093169	-1,16202	-0,60012	-0,50087	-0,80241	-0,84586	-0,24654	0,550045	1,142322	0,048227	-0,2305	-0,03084	0,301438	1,310169	0,577037	0,861565	0,005571	0,0972
NCI_IL2_mediated_signaling_events_Pathway_(T_	-0,13716	0,408967	0,314045	-1,36494	0,073322	-1,40191	-0,31468	-0,76729	-0,12825	0,702533	0,398184	0,248033	-0,09202	-0,39198	-0,20332	1,384106	0,894274	0,989351	0,005582	0,0972
reactome_NOTCH2_intracellular_domain_regulat	0,078362	-0,08362	-0,05056	-0,15978	0,221072	-0,20914	-0,48565	-0,37465	-0,63742	-1,11971	-0,9357	-0,90779	0,427041	-0,09054	0,182531	-2,22974	-1,33507	-1,38157	0,005617	0,0972
biocarta_polyadenylation_of_mrna_Main_Pathwa	0,230794	0,058398	0,012254	0,600653	0,621984	0,446425	0,4685	0,642127	-0,03087	-0,67451	-0,49559	-0,13562	0,021085	0,428214	0,371337	-2,30722	-1,77636	-0,84072	0,005861	0,09738
GPCR_Pathway_Gene_Expression_via_JUN_NFKB	0,021399	-0,87946	-0,23106	-0,91108	2,262803	0,120907	-6,29871	-4,46163	-7,81377	-3,59442	-0,53321	-1,34012	1,090731	1,043209	2,793744	-6,46412	-6,39922	-1,35781	0,005789	0,09738
KEGG_Endocytosis_Main_Pathway	-0,03054	-0,33468	-0,47162	0,076349	-0,82955	-0,18696	-1,7828	-1,46874	-2,23091	-1,29742	-1,62199	-1,35772	0,861697	0,923972	0,803004	-5,01682	-3,95663	-3,38812	0,005895	0,09738
KEGG_Fat_digestion_and_absorption_Main_Path	-0,06141	0,166883	-0,03364	-0,5471	-0,60565	-0,00666	0,128447	-0,15713	0,188104	0,336931	0,241847	0,624643	0,160361	-0,36849	-0,09056	0,713779	0,55108	0,190244	0,005908	0,09738
NCI_Validated_nuclear_estrogen_receptor_alpha_	-0,3862	-0,2944	-0,41817	0,213621	0,072796	0,080886	1,092785	1,067373	0,57523	-2,48534	-0,42221	-1,77213	0,91801	0,347862	1,162283	-6,46279	-4,66157	-3,97173	0,005854	0,09738
reactome_Amino_acid_and_oligopeptide_SLC_tra	-0,12255	0,33196	-0,00216	-0,66875	-0,39602	-0,98403	0,321828	0,04225	0,182234	0,686967	0,606658	-0,12707	-0,06541	-0,22539	0,100331	1,922927	1,825114	2,26736	0,005779	0,09738
reactome_Regulation_of_KIT_signaling_Main_Pat	-0,36288	-0,30148	-0,19516	0,475922	0,270247	-0,01955	-1,62963	-1,89753	-1,97324	-2,54334	-0,25431	-0,30071	-0,17731	-0,0246	0,295392	-1,30201	-1,86081	-1,07237	0,005862	0,09738
reactome_Synthesis_of_IPs_in_the_nucleus_Mair	0,005333	-0,1967	-0,11284	0,016281	0,37743	-0,17443	-0,82058	-0,73619	-1,20989	0,035132	-1,05847	-0,78776	0,100164	0,099447	0,047782	-1,10018	-1,49514	-1,18466	0,005724	0,09738
reactome_Transport_of_inorganic_cations_anion	-0,12255	0,33196	-0,00216	-0,66875	-0,39602	-0,98403	0,321828	0,04225	0,182234	0,686967	0,606658	-0,12707	-0,06541	-0,22539	0,100331	1,922927	1,825114	2,26736	0,005779	0,09738
NCI_Coregulation_of_Androgen_receptor_activity	-0,12645	-0,11874	-0,09545	-0,06794	-0,50934	0,567272	-0,36308	-0,54381	-0,67174	-1,63956	-0,50803	-0,69676	0,190798	0,412224	0,476644	-2,61629	-2,5853	-1,6137	0,005997	0,097807
reactome_G_alpha_12_13_signalling_events_Mai	-0,09958	-0,9166	-0,71203	1,989534	2,694947	0,63687	-4,06944	-4,44415	-5,0175	-7,46635	-0,0014	-0,60206	0,054082	1,26693	0,343622	-7,84511	-4,99978	-2,14301	0,005973	0,097807
KEGG_Pathogenic_Escherichia_coli_infection_Ma	-0,28428	-0,6319	-0,29528	0,692331	0,28552	-0,04111	-1,92879	-2,30242	-1,91968	-2,67296	-2,38937	-1,09437	-0,4785	-0,81742	-0,39245	-4,70468	-3,52368	-3,19108	0,006083	0,098694
reactome_Fatty_acids_Main_Pathway	0,310177	0,583648	0,722699	1,480539	0,304362	0,628488	-1,29072	-1,38687	0,506399	2,505423	2,047135	2,33728	-0,58579	0,04558	-0,60101	3,683586	2,38941	1,672429	0,006169	0,09886
reactome_Nuclear_import_of_Rev_protein_Main	0,39482	-0,12323	0,076102	1,951038	1,714121	1,614219	2,067484	2,781065	0,539372	-3,95498	-2,45203	-1,80008	0,093772	0,117398	0,61558	-10,159	-6,93391	-5,49036	0,00615	0,09886
reactome_PCP_CE_Main_Pathway	-0,69508	-0,51323	-0,22858	0,453358	0,896581	-0,23808	1,139601	1,211839	1,711997	-4,28327	-0,85907	-1,97332	0,683539	0,787818	0,061674	-0,7957	-0,34562	-0,56969	0,006189	0,09886
NCI_Neurotrophic_factor_mediated_Trk_recepto	0,138472	-0,70691	-0,69386	-0,80868	-0,89551	0,067767	-7,23781	-7,07829	-8,57017	-4,46474	-2,71159	-3,35134	0,085157	0,257959	0,581344	-7,89142	-4,6749	-4,27351	0,006393	0,09916
NCI_Validated_nuclear_estrogen_receptor_beta_	-0,05888	-0,30978	-0,15332	0,012134	0,452261	0,08665	-0,90513	-1,08815	-1,035	-0,86855	-0,00272	-0,81625	0,780145	0,472803	0,634472	-3,59636	-2,40658	-2,53355	0,006354	0,09916
reactome_Rev_mediated_nuclear_export_of_HIV	0,427435	-0,05745	0,08486	2,147413	1,665066	1,617491	1,618489	2,461306	0,180258	-4,16698	-2,79315	-2,13829	-0,12674	0,031199	0,254493	-10,2969	-6,81652	-5,16619	0,006358	0,09916
reactome_Termination_of_O_glycan_biosynthesis	-0,48375	0,51592	0,324225	-0,29686	-0,35648	0,295538	0,950961	-0,34392	1,686616	3,340558	1,008034	0,381338	-0,79144	-0,52343	-0,96667	4,534635	4,079936	3,95286	0,006313	0,09916
reactome_Transport_of_the_SLBP_Dependant_M	0,434969	-0,066	0,030105	2,014849	2,02032	1,19371	2,005039	2,770742	0,445045	-3,31598	-2,70699	-2,87576	0,374299	-0,38489	0,031008	-10,1664	-7,1018	-5,61692	0,006398	0,09916
reactome_Vpr_mediated_nuclear_import_of_PIC	0,192383	-0,31407	-0,03434	1,805007	2,040093	0,969867	1,384036	2,158988	-0,06077	-3,64747	-2,17155	-2,59151	-0,12679	-0,16625	0,101942	-9,72757	-7,39906	-5,63708	0,006277	0,09916
reactome_Histidine_catabolism_Main_Pathway	-0,01125	-0,10131	0,073656	-0,56762	-0,08685	0,071222	-0,13989	-0,32595	0,137836	1,162992	-0,00175	0,485964	0,01502	-0,26375	-0,20953	1,275376	0,882077	0,66423	0,006439	0,099293
reactome_TGF_beta_receptor_signaling_activate	-0,11114	-0,1674	-0,28416	0,220282	-0,10927	0,051424	-0,80663	-0,83289	-1,17437	-1,28336	-0,2668	-0,35424	0,041621	0,1802	-0,19824	-1,60979	-1,35282	-0,63631	0,006517	0,100004
Glucocorticoid_Receptor_Signaling_Pathway	2,150534	-0,44882	-0,59244	4,716058	3,742243	4,224541	5,33951	8,497734	0,343709	-10,8979	-1,59856	-2,40433	0,525157	1,58176	3,637146	-22,0783	-18,0892	-14,2953	0,006925	0,102768
IL-2_Pathway_Apoptosis_Inhibition	-0,35465	-0,30087	-0,2512	0,281281	0,592189	0,295588	-0,15124	0,355057	-0,31867	0,100931	-0,90509	0,80358	0,294412	0,496006	0,822055	-2,11164	-1,4353	-0,55813	0,006899	0,102768
KEGG_Cell_cycle_Main_Pathway	-0,79408	-0,32781	-0,49789	-1,49098	-0,56583	-1,71469	-9,15923	-9,54893	-7,3149	6,720622	5,865034	7,768711	0,5678	-0,30811	0,742164	8,808532	6,060961	3,263116	0,006806	0,102768
KEGG_Glycosphingolipid_biosynthesis_lacto_and_	0,282899	-0,5024	-0,42233	2,757659	1,803323	2,484517	-1,85844	-1,87535	-2,09321	0,356948	-0,3069	-0,57764	1,1176	0,371676	0,198557	-1,07608	-1,19057	0,216445	0,006892	0,102768
NCI_Endogenous_TLR_signaling_Pathway_(respira	-0,18041	-0,16465	-0,15475	-0,46039	-0,56785	-0,28175	-1,20636	-1,27114	-1,1619	1,932419	0,859575	1,235605	-0,95463	-0,8396	-0,46183	-0,11579	0,282499	-0,37639	0,006863	0,102768
reactome_Activation_of_DNA_fragmentation_fac	0,173223	-0,24193	-0,11838	0,514235	0,876514	0,78974	0,228174	0,438624	-0,00169	-0,93862	0,182976	0,585453	0,480648	0,009787	0,486525	-2,08535	-0,9403	-0,73263	0,00699	0,102768
reactome_Pre_NOTCH_Processing_in_the_Endop	-0,05471	-0,07489	-0,00345	0,538556	0,112636	0,106883	0,431556	0,496095	0,158526	-0,01789	-0,20725	-0,00367	0,320305	0,132914	0,331698	-1,04813	-1,29307	-0,29844	0,006994	0,102768
reactome_Synthesis_secretion_and_inactivation_	0,056529	0,259257	-0,02399	-0,1981	0,517954	1,000629	-1,48117	-1,63007	-1,80393	-0,19705	-0,11847	0,583634	0,933056	1,243278	0,870821	-1,38559	-1,13791	-0,12543	0,006891	0,102768
reactome_Transport_of_Mature_mRNA_derived_	0,910998	-0,00369	0,192462	2,823239	2,038272	1,351689	2,938813	4,102156	1,028218	-5,38329	-4,57505	-4,54985	0,825803	0,026246	0,475149	-15,3554	-12,2176	-9,62745	0,006958	0,102768
Ephrin-B_Pathway	-0,98651	-1,39566	-1,2429	2,092422	0,611486	0,376646	-6,33758	-6,70345	-6,60382	-6,94776	-1,37912	-0,33522	0,279957	-0,15775	1,079217	-11,7192	-9,0455	-5,07937	0,007113	0,104017
NCI_Signaling_events_mediated_by_TCPTP_Path	0,188932	0,005837	0,024253	0,148964	-0,49847	0,091544	-0,58929	-0,34637	-1,11796	-2,07039	-0,61134	-0,81282	0,413645	0,798779	0,432589	-2,27193	-2,26286	-1,20478	0,007166	0,104311
biocarta_ceramide_signaling_Pathway_(Pathway_	0,215957	-0,18053	0,074708	-0,38236	-0,53307	-1,17182	-1,77974	-1,64585	-1,72666	0,451418	-0,23338	0,069964	-0,57604	-0,12529	-0,57392	-0,02264	0,028459	-0,287	0,007315	0,104749
KEGG_Alzheimer_s_disease_Main_Pathway	-0,09347	-0,1221	-0,14687	0,123658	-0,23686	0,417974	-0,11891	0,108317	-0,11352	-0,38176	-0,06125	-0,25974	0,404032	0,39939	0,516198	-0,27586	-0,10309	-0,38741	0,007319	0,104749
NCI_EPHA_forward_signaling_Main_Pathway	-0,37589	-0,13629	-0,29753	-1,072	-1,47225	-1,5741	-1,94089	-2,45112	-1,88565	0,468236	0,891024	0,612408	-0,71724	-0,08635	0,349539	0,785941	1,649305	1,350117	0,007331	0,104749
reactome_Displacement_of_DNA_glycosylase_by_	-0,10542	0,013628	-0,02175	0,692047	0,466498	0,42791	0,107971	-0,06117	0,204648	-0,27123	-0,17799	-0,15767	0,194245	0,03609	-0,12225	-1,68885	-1,05787	-0,51469	0,007323	0,104749
biocarta_integrin_signaling_Main_Pathway	-0,20831	-0,34396	-0,50645	0,851252	1,065516	-0,38582	-2,61911	-2,20781	-2,85453	-0,66528	1,614696	-0,51399	1,820473	0,790194	1,529893	-1,20406	-0,99226	-0,72217	0,007626	0,106524
Cell_Migration_Adhesion_and_Angiogenesis_driv	-0,00279	-0,07892	-0,02533	0,231901	0,576243	0,203638	-0,19466	-0,15771	-0,53815	-0,23874	0,011361	-0,37596	-0,00792	0,011748	0,011437	-0,70723	-0,39612	-0,1837	0,007542	0,106524
reactome_cell_division_Main_Pathway	-0,01974	-0,1067	-0,06669	0,152148	-0,24601	-0,08624	0,619456	0,6866	0,353108	-0,34529	-0,99596	-0,68835	0,00722	0,04854	0,120613	-1,51791	-1,36319	-0,92848	0,007604	0,106524
reactome_Platelet_Adhesion_to_exposed_collage	-0,10331	0,069862	0,02442	-0,55317	-0,51504	-0,2814	-0,33839	-0,57428	0,347722	-0,0338	0,232227	-0,40714	-0,11976	-0,35363	-0,08515	0,782774	0,06669	0,486878	0,007622	0,106524
reactome_U12_Dependent_Splicing_Main_Pathw	0,983231	0,16757	0,086789	2,332564	1,815558	2,679301	1,79898	2,870693	-0,04288	-6,38505	-3,70927	-3,4053	0,192147	-0,34868	0,53838	-14,2772	-11,457	-9,70218	0,007561	0,106524
biocarta_mechanism_of_protein_import_into_the	0,060584	-0,11737	-0,04717	0,045731	-0,31066	0,237994	0,199595	0,332202	-0,02914	-1,66119	-0,86031	-0,12486	0,523995	0,632204	0,809256	-3,2433	-1,6587	-1,06581	0,008079	0,107555
dolichol_and_dolichyl_phosphate_biosynthesis	-0,1198	-0,14954	-0,12643	-0,26674	0,177569	-0,00913	0,049719	0,089632	-0,06786	-0,20904	-0,62243	-0,33731	-0,10966	0,011947	0,246543	-0,26555	-0,43503	-0,49919	0,008149	0,107555
KEGG_Caffeine_metabolism_Main_Pathway	-0,05251	0,088169	0,197325	-0,3654	0,207714	0,072272	0,244441	-0,08891	0,177248	1,709793	0,440294	0,757601	-0,51003	-0,59056	-0,39074	0,655457	0,262271	-0,01539	0,007821	0,107555
mTOR_Pathway_Metabolism_Stress_Response_a	-0,01572	-0,15891	-0,06657	-0,04972	0,757553	0,274366	-0,34762	-0,36161	-0,46524	-0,59738	-0,24016	-0,70845	0,419578	0,301531	0,487081	0,004049	0,152381	0,409781	0,008014	0,107555
NCI_Arf6_downstream_Pathway_(cortical_actin_	0,079701	-0,00012	-0,03363	0,167213	-0,26248	0,090284	-0,78249	-0,78034	-0,96781	-0,31003	-0,38932	-0,40414	0,201666	0,093962	-0,00617	-0,50031	-0,41752	-0,42261	0,008143	0,107555
NCI_Arf6_downstream_Pathway_(lamellipodium_	0,079701	-0,00012	-0,03363	0,167213	-0,26248	0,090284	-0,78249	-0,78034	-0,96781	-0,31003	-0,38932	-0,40414	0,201666	0,093962	-0,00617	-0,50031	-0,41752	-0,42261	0,008143	0,107555
NCI_Signaling_events_mediated_by_HDAC_Class_	0,006264	-0,19807	-0,16777	1,320413	0,251344	0,319348	-0,71588	-0,56077	-1,29865	-0,1346	-2,24727	-0,73915	-0,13873	0,380897	0,542106	-4,42859	-3,48121	-1,91	0,007825	0,107555
reactome_Activation_of_BIM_and_translocation_	0,069963	-0,04349	-0,01536	-0,02168	0,093568	0,377232	0,456269	0,622211	0,221012	-0,43107	-0,01488	-0,24664	0,048459	0,052857	0,149007	-0,95554	-0,80201	-0,45887	0,007915	0,107555
reactome_E2F_mediated_regulation_of_DNA_rep	-0,03833	-0,0926	-0,07684	-0,01587	0,332568	-0,26394	0,66201	0,657314	0,622613	-0,43705	-0,16272	-0,46538	0,122782	-0,04107	0,093038	-0,42057	-0,59128	-0,48932	0,007888	0,107555
reactome_G1_S_Specific_Transcription_Main_Pat	-0,03833	-0,0926	-0,07684	-0,01587	0,332568	-0,26394	0,66201	0,657314	0,622613	-0,43705	-0,16272	-0,46538	0,122782	-0,04107	0,093038	-0,42057	-0,59128	-0,48932	0,007888	0,107555
STAT3_Pathway_G1_to_S_Cell_Cycle_Progression	-0,05936	-0,09802	-0,11764	0,43123	0,058728	-0,13633	-0,14747	-0,16128	-0,25146	0,366063	-0,44856	-0,36128	0,190855	0,178606	0,248486	-1,02623	-0,76472	-0,7802	0,008055	0,107555
threonine_degradation	0,082251	0,015268	0,038428	0,158859	0,529653	-0,27552	-0,21295	-0,11682	-0,42967	-0,18365	-0,19825	-1,52893	0,132197	0,543905	0,740366	-2,13629	-1,96195	-1,30543	0,007955	0,107555
TNF_Signaling_Pathway_Gene_Expression_and_C	-0,02321	-0,17612	-0,25574	-0,4306	-0,9983	-0,31896	-1,89369	-1,18476	-2,81495	-0,88753	-2,39404	-2,62822	0,797798	0,605341	0,975541	-3,54164	-2,96388	-1,65761	0,008055	0,107555
2-deoxy-alpha-D-ribose_1-phosphate_degradatio	-0,0311	-0,06056	-0,09551	-0,08033	-0,24202	0,180834	-0,17648	-0,3371	-0,03572	-1,20272	-0,3177	-0,66501	0,121718	0,173741	0,119255	-0,30002	-0,54522	-0,32115	0,009369	0,107614
adenine_and_adenosine_salvage_II	0,064849	0,024979	0,025587	0,216034	0,323673	0,17127	0,081691	0,134164	-0,03481	-0,12249	0,162168	0,030705	0,046615	0,101259	0,167342	-0,56831	-0,37403	-0,37708	0,008437	0,107614
biocarta_alk_in_cardiac_myocytes_Main_Pathwa	-0,34088	-0,25234	-0,11889	-0,31794	-0,13526	-0,35553	-0,4537	-0,23444	-0,79698	-1,3197	-0,872	-1,00235	2,206649	2,149313	2,078248	-2,71724	-2,21416	-1,55033	0,009487	0,107614
biocarta_cycling_of_ran_in_nucleocytoplasmic_tr	0,053941	-0,09505	-0,02954	0,248452	0,111926	0,418324	-0,24175	-0,06471	-0,36995	-0,34322	-0,68529	0,133277	0,250178	0,414416	0,443616	-2,27807	-0,95218	-0,61685	0,009426	0,107614
biocarta_hop_pathway_in_cardiac_development_	-0,14471	0,005499	-0,03926	-0,30809	-0,04292	-0,25137	-0,25415	-0,46415	-0,10287	0,44302	0,178429	0,433734	0,261102	-0,20162	-0,10713	0,426672	0,732436	0,783277	0,009636	0,107614
biocarta_hop_pathway_in_cardiac_development_	-0,14471	0,005499	-0,03926	-0,30809	-0,04292	-0,25137	-0,25415	-0,46415	-0,10287	0,44302	0,178429	0,433734	0,261102	-0,20162	-0,10713	0,426672	0,732436	0,783277	0,009636	0,107614
biocarta_hop_pathway_in_cardiac_development_	-0,14471	0,005499	-0,03926	-0,30809	-0,04292	-0,25137	-0,25415	-0,46415	-0,10287	0,44302	0,178429	0,433734	0,261102	-0,20162	-0,10713	0,426672	0,732436	0,783277	0,009636	0,107614
biocarta_il_4_signaling_Pathway_(G1_phase_of_r	0,019905	0,365587	0,18809	-0,74824	-0,15038	0,071738	-0,36836	-0,43367	0,167212	1,436913	1,047042	1,361308	0,302457	-0,38915	-0,16587	-0,05293	0,802718	0,097457	0,008272	0,107614
biocarta_mets_affect_on_macrophage_differenti	0,039715	0,007236	-0,02413	0,567701	0,574135	0,547768	-0,01391	0,011957	-0,13006	-0,03784	-0,18325	-0,19427	-0,01285	0,092211	0,002962	-0,49526	-0,44518	-0,21756	0,009387	0,107614
diphthamide_biosynthesis	0,04742	-0,04263	-0,02206	0,2936	0,243687	0,226057	0,236526	0,389275	-0,02033	-0,18837	-0,259	-0,04869	0,025722	0,090951	0,107471	-1,24848	-1,07906	-1,04528	0,009635	0,107614
dTMP_ide_novoi_biosynthesis_mitochondrial	0,026449	0,011495	0,031039	0,414073	0,312421	0,291067	0,528803	0,52303	0,475544	-0,19657	0,115847	-0,13391	0,160868	0,093338	0,084135	0,018489	0,090659	0,150225	0,009671	0,107614
eicosapentaenoate_biosynthesis	0,264528	0,25125	0,127366	-1,44841	0,311702	-1,43409	0,93264	0,768783	0,697554	1,117983	0,549573	1,050009	-0,24644	-0,41531	-0,54982	-0,10262	0,113253	0,746601	0,009723	0,107614
Erythropoietin_Pathway_Anti-Apoptosis	0,085006	0,025381	0,097538	0,480436	0,734788	0,03216	-1,11466	-1,32419	-1,08615	-2,15604	-0,08068	-0,64451	0,869866	0,067406	0,085099	-1,98035	-0,76332	-0,45871	0,008342	0,107614
fatty_acid_activation	-0,00672	0,045611	0,14759	-0,37984	0,104341	-0,24463	0,395564	0,297717	0,242083	0,963321	0,279055	0,485837	0,205577	0,014187	-0,07623	0,197754	0,782985	0,549518	0,008266	0,107614
Glucocorticoid_Receptor_Signaling_Pathway_Hist	-0,03329	-0,05165	-0,04158	-0,02958	0,12792	0,055209	-0,53125	-0,53998	-0,69812	-0,41257	-0,28084	-0,30571	-0,1032	-0,08578	-0,05851	-0,84194	-0,51605	-0,38184	0,009278	0,107614
ILK_Signaling_Pathway_Tumor_Angiogenesis	-0,10001	-0,0489	-0,06487	-0,10652	0,002766	0,00606	-0,26387	-0,34718	-0,31065	0,22607	-0,6362	-0,39105	0,230999	0,236495	0,287247	-0,21155	-0,48585	-0,69061	0,008592	0,107614
KEGG_ABC_transporters_Main_Pathway	-0,28561	0,084039	0,402088	-1,49852	0,079059	-0,62856	-0,30256	-0,40577	0,207189	3,223924	0,034486	1,637661	0,23392	0,222038	-0,72017	5,153429	3,512079	3,603788	0,008671	0,107614
KEGG_Fatty_acid_elongation_Main_Pathway	0,046608	-0,05252	-0,03377	-0,05323	0,256211	0,07967	0,01182	0,105241	-0,11905	-0,0237	-0,34292	-0,01992	0,189657	-0,00503	0,188833	-0,79285	-0,70333	-0,58204	0,008893	0,107614
KEGG_Phenylalanine_metabolism_Main_Pathway	-0,28562	0,171187	0,073344	-0,47486	-0,90177	-0,72992	-1,73122	-2,27944	-0,79816	-0,26452	-0,39231	1,021461	-0,25079	-0,52393	-0,56353	2,706749	1,950341	1,716522	0,008516	0,107614
KEGG_Purine_metabolism_Main_Pathway	0,576859	-0,02903	-0,36178	0,84339	-1,09429	-1,00386	0,870264	2,387264	-2,93179	-7,78508	-5,263	-7,02926	2,67185	2,253051	3,493696	-20,2418	-19,2561	-17,0399	0,008328	0,107614
NCI_Aurora_B_signaling_Main_Pathway	0,469618	-0,12565	0,052225	0,805406	0,835509	1,341205	3,632505	3,847007	2,717743	-4,97778	-3,46247	-3,23141	-0,39648	-0,83039	-0,86532	-8,01654	-6,67294	-4,36848	0,008403	0,107614
NCI_Hedgehog_signaling_events_mediated_by_G	-0,01794	-0,00982	-0,19392	0,3899	0,216927	-0,05568	0,702291	0,503727	0,399384	1,117376	0,44677	1,440743	-0,44617	-0,14951	-0,44311	1,273495	1,050172	1,093303	0,009638	0,107614
NCI_N_cadherin_signaling_events_Pathway_(adh	0,02114	-0,03067	-0,05961	0,584502	0,263842	0,153007	-0,0722	-0,01126	-0,20475	-0,04019	0,009251	-0,32936	0,016113	0,038722	0,017654	-0,47614	-0,26664	-0,40478	0,009863	0,107614
NCI_PLK1_signaling_events_Main_Pathway	0,612867	-0,06188	0,115313	1,093162	0,872179	-0,51027	5,3663	6,444287	3,504946	-5,81034	-5,87354	-6,53976	-0,98099	-0,20396	-0,46363	-11,7629	-7,32312	-5,42567	0,009648	0,107614

NCI_PLK1_signaling_events_Pathway_(Golgi_orga	0,20705	-0,06283	-0,02175	0,110435	0,042336	0,054141	2,395815	2,803206	1,791598	-1,93685	-1,83782	-1,95751	-0,38883	-0,06834	-0,35207	-2,55203	-1,92892	-1,28903	0,009515	0,107614
NCI_PLK1_signaling_events_Pathway_(metaphase	0,148834	0,009196	0,080593	0,080929	-0,27025	-0,04242	1,607203	1,789812	1,152761	-0,91059	-1,12437	-1,19387	-0,0525	0,202405	0,068462	-1,4916	-1,33167	-0,83677	0,009024	0,107614
NCI_PLK1_signaling_events_Pathway_(positive_re	0,169419	-0,0052	0,055559	0,174246	-0,1496	0,01191	1,538634	1,732055	1,021354	-0,88479	-1,08547	-1,16558	-0,05709	0,282031	0,097199	-2,10708	-1,77687	-1,08765	0,008239	0,107614
NCI_PLK1_signaling_events_Pathway_(regulation_	0,20705	-0,06283	-0,02175	0,110435	0,042336	0,054141	2,395815	2,803206	1,791598	-1,93685	-1,83782	-1,95751	-0,38883	-0,06834	-0,35207	-2,55203	-1,92892	-1,28903	0,009515	0,107614
NCI_PLK1_signaling_events_Pathway_(spindle_as	0,231336	-0,07599	0,025851	0,035488	-0,06937	-0,08198	2,635639	3,015971	2,048619	-1,56488	-1,95168	-2,09911	-0,0682	-0,06356	-0,21093	-3,05872	-2,22882	-1,67854	0,009855	0,107614
NCI_PLK1_signaling_events_Pathway_(spindle_elo	0,20705	-0,06283	-0,02175	0,110435	0,042336	0,054141	2,395815	2,803206	1,791598	-1,93685	-1,83782	-1,95751	-0,38883	-0,06834	-0,35207	-2,55203	-1,92892	-1,28903	0,009515	0,107614
NCI_Sphingosine_1_phosphate_S1P_Main_Pathw	-0,06937	-0,62781	-0,20192	1,027599	0,763186	1,081729	-0,56276	-0,55649	-0,29497	-1,94897	-0,01945	-1,31559	0,028218	-0,39625	0,03048	-1,01313	-0,18802	-0,2301	0,009384	0,107614
NCI_Stabilization_and_expansion_of_the_E_cadh	0,02114	-0,03067	-0,05961	0,584502	0,263842	0,153007	-0,0722	-0,01126	-0,20475	-0,04019	0,009251	-0,32936	0,016113	0,038722	0,017654	-0,47614	-0,26664	-0,40478	0,009863	0,107614
NCI_Syndecan_3_mediated_signaling_events_Pat	0,01726	0,008185	0,018636	0,127704	0,408336	0,156281	0,657481	0,590855	0,282111	-0,91074	0,046623	-1,07649	0,223369	0,345206	0,247956	-1,16135	0,133873	-0,36695	0,008451	0,107614
Notch_Signaling_Pathway_gamma_Secretase	0,113627	-0,01589	-0,01306	0,010365	0,422806	-0,01563	-0,383	-0,27257	-0,63176	-0,14554	0,067382	0,019898	0,522084	0,746379	0,653019	-1,44762	-0,25313	-0,75363	0,009311	0,107614
pyrimidine_deoxyribonucleosides_salvage	-0,00646	0,010596	-0,03377	0,839857	0,64964	0,478011	0,588685	0,761877	0,671216	-0,67474	-0,40214	-0,80546	0,181638	0,019237	0,10402	0,135463	0,059425	0,123293	0,009625	0,107614
Ras_Pathway_Gene_Expression_Cell_Proliferation	0,97981	-0,62099	-0,09616	1,409499	1,029019	-0,21627	-2,62945	-1,12238	-4,76859	-6,0666	-1,7562	-2,04649	1,31399	1,141443	1,841284	-12,9758	-8,90082	-7,70351	0,008941	0,107614
reactome_Association_of_TriC_CCT_with_target_	0,298082	-0,14092	0,136766	0,746444	0,82395	0,103498	-1,29051	-0,45686	-2,04758	-3,35733	-0,99605	-2,58509	0,066312	0,690031	0,619963	-8,04069	-4,466	-3,37325	0,009889	0,107614
reactome_Cyclin_A_Cdk2_associated_events_at_	0,385059	-0,13649	-0,17855	0,948825	1,059985	-0,46095	0,133516	0,56139	-0,42781	-1,93029	-1,63013	-0,91098	0,16067	0,295799	-0,23029	-5,23107	-3,53348	-3,24894	0,009731	0,107614
reactome_E2F_enabled_inhibition_of_pre_replica	0,588814	0,096064	-0,0455	1,08032	0,769731	0,732311	0,717409	1,2469	-0,09938	-2,02936	-0,83714	-2,2853	-0,20359	0,153097	-0,3979	-4,22534	-2,8646	-1,31774	0,009915	0,107614
reactome_Elongation_arrest_and_recovery_Main	0,354236	-0,2417	-0,33973	0,950656	0,111812	0,242792	0,186612	0,728776	-0,73234	-3,30689	-1,80261	-1,21383	-0,28614	0,036548	0,417075	-6,59669	-5,27658	-3,74742	0,009824	0,107614
reactome_HIV_elongation_arrest_and_recovery_	0,354236	-0,2417	-0,33973	0,950656	0,111812	0,242792	0,186612	0,728776	-0,73234	-3,30689	-1,80261	-1,21383	-0,28614	0,036548	0,417075	-6,59669	-5,27658	-3,74742	0,009824	0,107614
reactome_Organic_anion_transporters_Main_Pat	-0,20688	-0,07153	0,0502	-0,19987	-0,07122	-0,14325	0,110554	-0,17492	0,285365	0,672234	0,454356	0,688079	-0,22996	0,160019	-0,14198	1,570713	0,792711	0,705015	0,009002	0,107614
reactome_Pausing_and_recovery_of_HIV_elonga	0,354236	-0,2417	-0,33973	0,950656	0,111812	0,242792	0,186612	0,728776	-0,73234	-3,30689	-1,80261	-1,21383	-0,28614	0,036548	0,417075	-6,59669	-5,27658	-3,74742	0,009824	0,107614
reactome_Polo_like_kinase_mediated_events_M	-0,07718	-0,12352	-0,24174	0,636404	0,499747	0,202597	1,606907	2,07066	1,10368	-2,12873	-2,62738	-1,25893	-0,79778	-0,61949	-0,21916	-4,24885	-2,15101	-2,06965	0,009688	0,107614
reactome_Processing_of_Intronless_Pre_mRNAs_	0,455305	0,055234	0,000565	1,081567	0,630544	1,180544	0,239085	0,55669	-0,47774	-1,43867	-0,83246	-0,95543	-0,00806	-0,02174	0,379132	-4,61881	-4,04142	-2,35483	0,009126	0,107614
reactome_Removal_of_the_Flap_Intermediate_fr	0,252134	0,027847	0,065368	0,160373	0,759691	0,776361	2,567337	2,93805	2,559623	-1,0036	-0,26157	-0,5505	-0,01694	-0,00185	-0,17055	-1,6015	-1,71598	-1,10767	0,009542	0,107614
reactome_Role_of_LAT2_NTAL_LAB_on_calcium_	0,082338	0,022627	-0,14277	0,100814	-0,07539	0,048299	-0,86048	-0,93137	-1,29048	-1,2888	-1,00497	0,373415	0,601808	0,533311	0,549939	-2,67041	-1,9289	-1,56232	0,009264	0,107614
reactome_Signaling_by_NOTCH3_Main_Pathway	0,104381	0,02715	-0,04965	-0,01209	0,948188	0,449182	-0,61409	-0,52581	-0,72351	-0,01951	0,848377	0,22146	0,992779	1,108233	0,91636	-1,14852	-0,04093	-0,26971	0,008644	0,107614
reactome_snRNP_Assembly_Main_Pathway	1,299668	0,427383	0,28088	4,691047	3,605535	4,436679	1,990829	3,08403	-0,92955	-5,39383	-2,23523	-3,08316	-0,19405	-0,18704	0,105547	-16,2036	-13,1087	-10,3402	0,008878	0,107614
Role_of_Small_GTPases_in_G1-S_Transition	-0,01977	-0,87932	-0,71823	-0,99769	1,125139	-0,12509	-2,30968	-1,73035	-3,72615	-3,30734	-0,6757	0,04088	1,023002	0,423479	0,824114	-6,03348	-4,05651	-2,77559	0,009631	0,107614
STAT3_Pathway_Growth_Arrest_and_Differentiat	-0,70468	-0,01375	0,133965	-0,84527	-0,81959	-4,42017	-8,75159	-9,36588	-4,28118	11,36867	3,590713	2,130397	1,907408	-0,27581	-0,06328	10,69921	10,31023	7,824909	0,009773	0,107614
KEGG_Pyrimidine_metabolism_Main_Pathway	0,8552	0,040625	-0,59705	0,871252	1,717468	-1,66616	2,768879	4,411151	-0,44566	-7,43609	-7,55159	-6,9394	0,466564	0,857057	1,423587	-21,6484	-17,3121	-15,5255	0,009989	0,107666
KEGG_Ribosome_biogenesis_in_eukaryotes_Main	1,142677	-0,65822	-0,5205	3,725104	5,090237	2,476367	0,121148	1,408367	-3,95499	-7,22199	0,956531	-3,49152	1,121071	1,758065	2,079124	-22,0408	-17,2179	-14,9143	0,009973	0,107666
Regulation_of_Cytoskeleton_Remodeling_by_Pro	-0,45335	-0,63488	-0,67338	2,131701	2,770415	0,606161	-3,50863	-3,44865	-4,55988	-5,55393	-0,1836	0,582979	-0,46821	0,565246	0,998597	-5,98196	-3,42716	-0,10198	0,010052	0,10797
NCI_Signaling_events_mediated_by_TCPTP_Path	-0,08576	-0,22674	-0,18261	0,033039	-0,61785	-0,54937	-1,10374	-1,02796	-1,4094	-1,86906	-0,95788	-0,41944	0,710567	1,065072	0,764426	-2,33507	-2,40935	-1,01216	0,010152	0,108296
WNT_Pathway_Cell_Survival	0,00021	0,184683	0,074178	-0,34771	0,772603	-0,07713	-1,27716	-1,11763	-2,14083	-1,61328	-0,88688	-0,54558	1,628937	1,369534	1,227456	-1,39627	-0,66253	0,171966	0,010148	0,108296
biocarta_inhibition_of_matrix_metalloproteinase	-0,14348	-0,03027	-0,04858	0,391546	0,27518	-0,21467	-0,76156	-0,65772	-0,78191	-1,19042	0,40045	-0,56691	0,221512	0,394302	0,262645	-0,51045	-0,48026	-0,15794	0,010983	0,109677
biocarta_inhibition_of_matrix_metalloproteinase	-0,14348	-0,03027	-0,04858	0,391546	0,27518	-0,21467	-0,76156	-0,65772	-0,78191	-1,19042	0,40045	-0,56691	0,221512	0,394302	0,262645	-0,51045	-0,48026	-0,15794	0,010983	0,109677
biocarta_lissencephaly_gene_lis1_in_neuronal_m	0,029623	-0,05983	0,032277	0,329907	-0,28717	0,230896	-1,05757	-1,06388	-1,40948	0,096054	-0,01877	-0,14376	0,392067	0,707657	0,76089	-1,19432	-1,00015	-0,64682	0,010418	0,109677
biocarta_regulation_of_bad_phosphorylation_Ma	0,012339	-0,25715	-0,39183	0,384881	1,860818	1,244647	-2,91473	-2,88537	-3,22508	-0,38431	0,162164	-0,46508	-0,16118	-0,06278	0,070893	-2,95172	-3,02162	-2,62423	0,010824	0,109677
biocarta_role_of_ran_in_mitotic_spindle_regulati	0,14069	-0,00345	-0,05944	0,077515	-0,82657	0,599431	1,053529	1,269476	0,710973	-2,94443	-1,55177	-1,66484	0,032718	0,28952	0,305249	-3,62174	-1,81101	-1,04378	0,010874	0,109677
biocarta_the_co_stimulatory_signal_during_t_cel	-0,33977	0,206284	0,025137	-0,61496	-0,74388	0,751749	0,500081	0,219926	1,104716	3,073517	-0,34585	0,940686	-0,92874	-0,61013	-0,72065	1,627099	1,744688	1,021913	0,010509	0,109677
biocarta_the_co_stimulatory_signal_during_t_cel	-0,33977	0,206284	0,025137	-0,61496	-0,74388	0,751749	0,500081	0,219926	1,104716	3,073517	-0,34585	0,940686	-0,92874	-0,61013	-0,72065	1,627099	1,744688	1,021913	0,010509	0,109677
fatty_acid_biosynthesis_initiation	0,059921	-0,03397	-0,03296	-0,22938	0,098544	0,323505	0,131321	0,158175	-0,01587	-0,84042	0,017084	-0,75702	0,011973	0,005288	0,152585	-0,71354	-0,97739	-0,54901	0,010409	0,109677
NCI_IL27_mediated_signaling_events_Pathway_(c	0,012984	-0,12163	-0,04514	-0,53432	-0,21711	-0,54601	-0,42459	-0,42305	-0,57563	1,435751	0,731876	0,727491	-0,51456	0,102594	0,060895	-0,29149	0,4264	0,549103	0,011056	0,109677
NCI_IL27_mediated_signaling_events_Pathway_(I	0,012984	-0,12163	-0,04514	-0,53432	-0,21711	-0,54601	-0,42459	-0,42305	-0,57563	1,435751	0,731876	0,727491	-0,51456	0,102594	0,060895	-0,29149	0,4264	0,549103	0,011056	0,109677
NCI_IL27_mediated_signaling_events_Pathway_(n	0,012984	-0,12163	-0,04514	-0,53432	-0,21711	-0,54601	-0,42459	-0,42305	-0,57563	1,435751	0,731876	0,727491	-0,51456	0,102594	0,060895	-0,29149	0,4264	0,549103	0,011056	0,109677
NCI_IL27_mediated_signaling_events_Pathway_(p	0,012984	-0,12163	-0,04514	-0,53432	-0,21711	-0,54601	-0,42459	-0,42305	-0,57563	1,435751	0,731876	0,727491	-0,51456	0,102594	0,060895	-0,29149	0,4264	0,549103	0,011056	0,109677
NCI_IL27_mediated_signaling_events_Pathway_(T	0,012984	-0,12163	-0,04514	-0,53432	-0,21711	-0,54601	-0,42459	-0,42305	-0,57563	1,435751	0,731876	0,727491	-0,51456	0,102594	0,060895	-0,29149	0,4264	0,549103	0,011056	0,109677
NCI_IL27_mediated_signaling_events_Pathway_(T	0,012984	-0,12163	-0,04514	-0,53432	-0,21711	-0,54601	-0,42459	-0,42305	-0,57563	1,435751	0,731876	0,727491	-0,51456	0,102594	0,060895	-0,29149	0,4264	0,549103	0,011056	0,109677
NCI_IL27_mediated_signaling_events_Pathway_(T	0,012984	-0,12163	-0,04514	-0,53432	-0,21711	-0,54601	-0,42459	-0,42305	-0,57563	1,435751	0,731876	0,727491	-0,51456	0,102594	0,060895	-0,29149	0,4264	0,549103	0,011056	0,109677
NCI_PAR1_mediated_thrombin_signaling_events_	0,013998	-0,01982	0,026777	0,284475	-0,48738	-0,06593	-0,69176	-0,60866	-0,87292	-1,48458	-0,79121	-1,14275	0,240137	-0,08923	0,19259	-1,62583	-0,96521	-0,54857	0,010564	0,109677
reactome_Cleavage_of_Growing_Transcript_in_th	0,96653	0,120858	0,177964	1,763779	0,487941	1,301026	1,741059	2,440904	0,679018	-3,18475	-2,48798	-2,75338	0,540758	0,445867	0,884248	-10,706	-9,69414	-6,97032	0,01099	0,109677
reactome_mRNA_3_end_processing_Main_Pathw	0,96653	0,120858	0,177964	1,763779	0,487941	1,301026	1,741059	2,440904	0,679018	-3,18475	-2,48798	-2,75338	0,540758	0,445867	0,884248	-10,706	-9,69414	-6,97032	0,01099	0,109677
reactome_mTOR_signalling_Main_Pathway	0,072772	0,016939	0,033611	0,212948	0,024749	0,062927	0,218504	0,276469	-0,01202	-0,4659	-0,11731	-0,02314	0,067464	0,104235	0,209511	-0,56784	-0,51659	-0,01638	0,011011	0,109677
reactome_Pausing_and_recovery_of_Tat_mediat	0,318486	-0,23163	-0,34865	1,076508	0,173073	0,248354	0,317492	0,824637	-0,51423	-3,16166	-1,39413	-0,91835	-0,3656	-0,00828	0,383445	-6,18596	-5,06896	-3,60966	0,011003	0,109677
reactome_Tat_mediated_HIV_elongation_arrest_	0,318486	-0,23163	-0,34865	1,076508	0,173073	0,248354	0,317492	0,824637	-0,51423	-3,16166	-1,39413	-0,91835	-0,3656	-0,00828	0,383445	-6,18596	-5,06896	-3,60966	0,011003	0,109677
thyroid_hormone_metabolism_II_via_conjugatio	-0,10337	-0,00574	0,175506	-0,41935	-0,48307	-0,8607	0,430055	0,508188	0,71766	0,693882	0,952986	1,026469	-0,30815	-0,65116	-0,60806	-0,63792	-0,25481	-0,58666	0,010728	0,109677
NCI_PLK1_signaling_events_Pathway_(proteasom	0,106362	-0,08013	0,027002	-0,0043	-0,02841	-0,49906	1,888433	2,093129	1,424662	-1,30779	-1,32285	-1,56552	-0,19784	0,083288	-0,09691	-1,47411	-1,64645	-1,0627	0,011127	0,110036
reactome_Phosphate_bond_hydrolysis_by_NUDT	0,010831	-0,06827	-0,08702	0,158133	0,163638	0,232912	0,316189	0,258614	0,081951	-0,02323	-0,63383	-0,06005	-0,02775	0,080666	0,275952	-0,33312	-1,3949	-0,83179	0,011311	0,111496
iSi-methyl-5-thio-alpha-D-ribose_1-phosphate_de	0,021853	-0,0795	-0,11616	0,258714	-0,21706	0,078563	-0,57326	-0,28453	-0,75824	0,200794	-0,44702	-1,13594	0,308909	0,429514	0,255147	-2,22994	-1,61664	-1,54254	0,011489	0,112192
NCI_Ceramide_signaling_Pathway_(cell_survival)	0,018835	-0,32357	-0,20426	-1,01183	-0,8707	-1,30863	-1,08305	-1,19457	-0,98632	-0,68368	-0,34939	1,533667	-0,76533	-0,03461	-0,7032	0,127198	0,107376	-0,08544	0,01142	0,112192
reactome_pre_mRNA_splicing_Main_Pathway	2,689298	0,201724	0,233858	6,173484	2,703141	4,757503	5,040588	7,525929	0,903061	-11,4627	-9,95756	-8,0222	0,119875	0,74649	1,949224	-39,2152	-31,1352	-24,3315	0,011481	0,112192
biocarta_e2f1_destruction_Main_Pathway	-0,02287	-0,32877	-0,15042	0,452673	1,253782	-0,59713	1,244329	1,382883	1,106027	-0,43571	-0,04478	-0,47236	0,18606	0,26733	0,267281	-1,75436	-1,79108	-1,37398	0,011542	0,112352
biocarta_il22_soluble_receptor_signaling_Main_F	-0,08593	-0,14527	-0,13175	-0,31873	-0,28002	-0,38867	0,344827	0,39445	0,669215	1,533512	-0,23757	0,709611	-0,22429	-0,02974	0,004031	1,18726	1,206245	1,234506	0,011655	0,113104
gamma-linolenate_biosynthesis	0,150892	0,177895	0,184078	-0,88714	0,664263	-0,78566	0,861134	0,659702	0,871541	1,220584	0,372406	0,603893	0,221597	-0,2719	-0,54597	1,013804	0,899214	1,278504	0,011712	0,113297
biocarta_double_stranded_rna_induced_gene_ex	0,25199	0,083792	0,06813	0,498532	-0,03428	0,402966	0,015559	0,179828	-0,26697	-0,31623	-0,7743	-1,22221	-0,12771	-0,19648	0,19987	-1,68914	-1,49154	-1,12533	0,01197	0,1141
NCI_EPHA_forward_signaling_Pathway_(proteaso	-0,26724	-0,00081	-0,1887	-1,87637	-1,84881	-2,15969	-1,59598	-2,02968	-1,53877	0,387703	0,211094	1,014253	-0,60258	0,015189	0,125641	0,650649	1,510705	1,03835	0,011978	0,1141
NCI_Role_of_Calcineurin_dependent_NFAT_signa	-0,01904	-0,1004	-0,09619	0,489733	0,425322	0,183344	-0,25786	-0,2804	-0,27884	-0,28126	-0,02328	-0,28285	0,259287	-0,00456	0,189639	0,204191	0,274615	0,031566	0,011883	0,1141
reactome_FCERI_mediated_NF_kB_activation_Ma	-0,01651	-0,21915	-0,28878	-1,08854	0,046401	0,02321	-1,5336	-1,41212	-2,39409	-1,26567	-1,3697	-1,19313	1,590445	0,666304	0,811189	-1,81121	-2,02466	-1,22389	0,011948	0,1141
reactome_Synthesis_of_Lipoxins_LX__Main_Path	-0,0172	-0,14024	0,173012	-0,35865	-0,68401	-0,26988	-0,31788	-0,58482	-0,36806	1,239048	0,294167	0,031591	0,079153	-0,46114	0,258683	1,376353	1,279742	0,209247	0,011952	0,1141
arsenate_detoxification_I_glutaredoxin	0,03297	0,127774	0,094789	-0,7837	-0,48533	-0,57821	-0,4216	-0,5058	-0,47314	-0,05613	0,219963	0,330908	0,135296	0,069931	-0,03497	0,804633	0,542887	0,609805	0,012306	0,114266
biocarta_p38_mapk_signaling_Main_Pathway	0,304819	-0,09214	-0,39002	-1,27198	-1,0478	-0,0812	-0,73836	-0,23155	-1,55379	-3,17998	-2,16207	-1,94927	1,121305	0,906797	1,215449	-4,40566	-3,11187	-1,9668	0,012077	0,114266
biocarta_rna_polymerase_iii_transcription_Main_	-0,04059	-0,14082	-0,24028	0,177654	0,437381	0,111928	-0,37931	-0,18096	-0,72352	-0,48158	-0,15332	-0,02623	-0,05196	-0,00434	0,056996	-1,85489	-1,34991	-1,35872	0,012542	0,114266
biocarta_rna_polymerase_iii_transcription_Pathw	-0,04059	-0,14082	-0,24028	0,177654	0,437381	0,111928	-0,37931	-0,18096	-0,72352	-0,48158	-0,15332	-0,02623	-0,05196	-0,00434	0,056996	-1,85489	-1,34991	-1,35872	0,012542	0,114266
EGF_Pathway_Cytoskeleton_Regulation	-0,21933	-0,12294	-0,15489	-2,10501	-1,78869	-1,78868	-2,66933	-2,54439	-3,01748	1,123654	0,621522	0,713624	-0,90297	-0,17898	-0,352	-0,39757	-0,97284	-0,12547	0,012471	0,114266
estradiol_biosynthesis_II	0,053058	0,039003	0,030302	0,080161	-0,14865	-0,27392	-0,4631	-0,41926	-0,32887	0,046695	0,107742	-0,0185	-0,04984	-0,19306	-0,2717	0,568624	0,015917	0,027734	0,012403	0,114266
iNi-acetylglucosamine_degradation_II	0,092674	0,071035	-0,04387	-0,27441	0,434474	0,072248	-0,77095	-0,66777	-0,94086	-0,81751	-0,2002	0,030646	0,09528	0,407607	0,403787	-1,43039	-1,44912	-1,24525	0,012766	0,114266
KEGG_RNA_polymerase_Main_Pathway	0,214763	-0,31617	-0,61983	0,84403	-0,58934	-1,05716	-1,03187	-0,18305	-2,73477	-3,54	-2,24187	-2,59718	0,634251	0,745866	0,772018	-10,2518	-8,57408	-7,72549	0,012358	0,114266
KEGG_Tyrosine_metabolism_Main_Pathway	-0,46213	0,127193	-0,04398	-1,81367	-2,26904	-2,5309	-3,12095	-3,23524	-1,59786	0,600302	-0,89304	-0,07024	-0,20415	0,014233	-0,40412	2,440457	2,197889	1,688943	0,012122	0,114266
NCI_ATM_Main_Pathway	0,271255	-0,21758	-0,35429	1,837255	2,716958	1,526586	1,963332	2,893046	0,358062	-0,8647	-0,52467	-0,94419	-0,61748	-0,13542	0,260216	-5,80759	-2,29112	-1,93995	0,012665	0,114266
NCI_Notch_signaling_Pathway_(skeletal_muscle_	-0,04792	-0,02998	-0,06871	0,762792	0,464434	0,375632	-1,44651	-1,3387	-1,42595	-0,10957	0,739428	0,663203	0,407714	0,408198	0,588074	-0,98651	-0,6457	-0,38031	0,012306	0,114266
NCI_Plasma_membrane_estrogen_receptor_signa	0,018699	-0,65933	-0,6053	-0,87844	1,761117	0,082511	-2,91535	-2,78581	-3,24203	-1,31096	-0,06533	-1,4736	0,16778	0,099325	0,447263	-4,92054	-4,73152	-2,80559	0,012571	0,114266
NCI_S1P1_Pathway_(negative_regulation_of_cAN	0,056278	-0,18235	-0,14105	0,012742	0,258123	0,401619	0,007031	0,148499	-0,12313	0,018232	0,043211	-0,57159	0,156628	0,08021	0,150532	-1,63459	-1,58129	-1,10062	0,012724	0,114266
NCI_S1P1_Pathway_(negative_regulation_of_T_c	0,056278	-0,18235	-0,14105	0,012742	0,258123	0,401619	0,007031	0,148499	-0,12313	0,018232	0,043211	-0,57159	0,156628	0,08021	0,150532	-1,63459	-1,58129	-1,10062	0,012724	0,114266
NCI_S1P4_Pathway_(cell_migration)	0,164037	-0,14599	-0,09593	0,350823	0,035822	0,63532	-0,06627	0,045929	-0,14172	-0,39702	-0,03033	-0,14843	0,035322	0,196085	0,243419	-1,51811	-1,2102	-1,29448	0,012561	0,114266
NCI_S1P5_Pathway_(negative_regulation_of_cAN	0,085543	-0,09985	-0,08732	0,513253	0,103333	0,324822	-0,17717	-0,16436	-0,20324	-0,08491	0,025292	-0,31658	0,155447	-0,01922	0,182859	-1,31775	-1,64281	-0,97332	0,012211	0,114266
reactome_Activation_of_the_AP_1_family_of_tra	0,076066	-0,26951	-0,30447	-0,16786	-0,1443	-0,14831	-0,40858	-0,1069	-1,03865	-0,5828	0,022505	-0,2473	0,559492	0,423831	0,608146	-2,19886	-1,38749	-1,46248	0,012455	0,114266
reactome_Axonal_growth_stimulation_Main_Pat	-0,13512	-0,08371	-0,02761	0,079397	0,385519	0,434832	-0,54884	-0,56749	-0,38057	0,03631	-0,20427	0,083489	0,265143	-0,16558	0,169445	-0,34855	-0,33969	-0,20913	0,012158	0,114266
reactome_CDC6_association_with_the_ORC_origi	0,543984	0,122047	0,047024	1,273672	1,17308	1,229503	0,881521	1,285208	0,170492	-1,12033	0,54452	-1,31621	-0,01229	0,31493	-0,10159	-3,15198	-2,19888	-0,77893	0,012731	0,114266
reactome_CREB_phosphorylation_through_the_a	0,012276	-0,09249	-0,29315	0,321925	0,079661	0,102794	-1,31853	-1,21133	-2,08014	-1,41766	0,710704	-0,88221	0,075117	0,15002	0,565955	-2,25898	-1,81736	-1,30538	0,012736	0,114266
STAT3_Pathway	-4,04027	0,168779	0,75789	-7,00637	-8,18024	-9,26423	-11,5091	-15,3638	-2,27209	16,51624	0,406325	3,826103	-0,04721	-1,83427	-1,63959	27,54291	26,94116	20,66256	0,012081	0,114266
NCI_Notch_mediated_HES_HEY_network_Main_F	-0,51877	-0,4646	-0,4039	-0,59646	-1,16861	-0,78083	-0,39041	-0,25349	-0,83379	-1,77284	-1,92154	-0,43201	1,401331	1,405035	1,395009	-4,21179	-3,85557	-2,62817	0,012813	0,114365
KEGG_RNA_transport_Main_Pathway	1,190681	-0,08217	0,156604	1,941598	3,196034	3,261963	2,3095	3,560917	-0,03003	-3,35138	-1,98127	-4,46883	0,305946	1,001261	1,214131	-18,7482	-16,1767	-12,9007	0,01286	0,114454
reactome_Na_Cl_dependent_neurotransmitter_t	0,07544	0,214946	0,254104	-0,54499	-0,31574	-0,53601	1,299012	0,708566	2,83842	1,808886	1,949578	-0,12164	0,091375	-0,23002	-0,646	6,304031	4,829373	4,257204	0,012944	0,114871
NCI_Reelin_signaling_Pathway_(long_term_mem	-0,02941	0,184469	0,172218	-0,083	0,065209	-0,13576	-2,29717	-2,00532	-2,04824	0,767938	0,321136	-0,24557	-0,15123	-0,35331	0,069701	1,011989	1,341652	1,462039	0,013192	0,115463
NCI_Signaling_events_mediated_by_TCPTP_Path	0,168421	-0,03829	-0,06598	0,149203	-0,56537	-0,22384	-0,42806	-0,22881	-1,0422	-1,4541	-0,3447	-0,62955	0,57399	0,904091	0,572452	-2,98344	-2,62769	-1,6668	0,013196	0,115463
NCI_Signaling_events_mediated_by_TCPTP_Path	0,168421	-0,03829	-0,06598	0,149203	-0,56537	-0,22384	-0,42806	-0,22881	-1,0422	-1,4541	-0,3447	-0,62955	0,57399	0,904091	0,572452	-2,98344	-2,62769	-1,6668	0,013196	0,115463
reactome_G2_M_DNA_replication_checkpoint_M	0,110998	-0,01205	-0,00318	0,153163	0,446032	0,276832	0,954492	1,260915	0,730853	-1,77781	-1,65869	-1,01968	-0,3431	-0,347	-0,40764	-2,30414	-1,38475	-0,92403	0,013185	0,115463
reactome_Ketone_body_catabolism_Main_Pathw	0,060947	-0,04906	-0,02747	-0,0249	-0,03475	0,15925	-0,94808	-0,85676	-1,14898	-0,63282	-0,26092	0,163909	0,192192	0,063343	0,299356	-1,00815	-1,06126	-0,70467	0,013182	0,115463
NCI_BCR_signaling_Pathway_(actin_cytoskeleton	-0,08774	0,075424	0,000289	-0,16944	-0,0506	-0,62003	-0,11203	-0,43757	-0,03725	0,18277	0,333196	-0,31166	-0,23593	-0,23013	-0,28866	0,458903	-0,27081	0,259289	0,013648	0,119087
biocarta_hypoxia_inducible_factor_in_the_cardiv	0,076446	-0,16487	-0,23945	-0,73946	0,190629	-0,01439	-0,58851	-0,36722	-1,08538	-0,69682	-0,16118	-1,25976	0,445561	0,462411	0,795387	-3,335	-2,0475	-1,85938	0,01384	0,119089
biocarta_transcription_regulation_by_methyltran	-0,17719	-0,33809	-0,45349	-0,41003	0,721337	1,155902	-2,20193	-2,00209	-2,54864	-1,59726	-0,48455	-0,09046	-0,41191	-0,26884	0,255085	-3,64616	-2,40398	-1,86648	0,013691	0,119089
NCI_LPA_receptor_mediated_events_Pathway_(p	-0,09681	0,076972	0,05044	0,411605	-0,19241	-0,16537	-0,00439	-0,11992	0,190223	0,647008	0,254961	0,807002	-0,25289	-0,74385	-0,58703	0,182268	0,590334	0,53708	0,013749	0,119089
NCI_Nongenotropic_Androgen_signaling_Pathwa	-0,11853	-0,28393	-0,30148	0,845114	0,553252	-0,48262	-0,56676	-0,26544	-0,9006	-1,70311	0,029664	-0,23889	0,17552	-0,11555	0,453569	-3,42829	-3,40903	-1,86794	0,013834	0,119089
reactome_TRAF3_dependent_IRF_activation_Mai	0,104879	-0,17621	-0,19675	0,284564	0,58122	0,466646	-0,95908	-0,90563	-1,27346	0,094915	-0,45613	-1,17401	0,319771	0,177169	0,616236	-0,11977	0,069858	0,691712	0,01382	0,119089
NCI_IL4_mediated_signaling_events_Main_Pathw	-0,14598	0,147358	0,153959	-0,94885	-0,18696	-0,21113	-1,29845	-1,69587	-0,5316	1,413992	0,947168	0,575129	0,660695	-0,17751	0,265962	0,906818	1,715939	1,357538	0,01389	0,119192
NCI_HIF_2_alpha_transcription_factor_network_I	-0,13883	-0,17148	-0,27203	-0,5324	-0,01383	-0,33721	-0,52657	-0,24888	-0,94256	-0,02141	-1,55269	-1,32411	0,634001	0,181242	0,684343	-3,63352	-2,3548	-1,46867	0,014011	0,119898
2-oxoisovalerate_decarboxylation_to_isobutanoy	0,093087	0,08663	0,063511	0,279854	0,612467	0,227401	0,073078	0,220325	-0,1158	0,057202	0,076366	-0,88319	0,019061	0,261326	0,510587	-2,09538	-1,97779	-1,48618	0,014191	0,120446
reactome_EPHB_mediated_forward_signaling_M	0,000152	-0,65727	-0,57051	1,201558	0,609408	0,153235	-4,66784	-4,13763	-5,61859	-2,87414	-0,0359	-0,37319	-0,13988	-0,00979	0,285977	-4,94152	-3,01827	-0,71073	0,014173	0,120446
triacylglycerol_degradation	-0,33701	0,109903	0,056809	-0,17755	0,073878	0,303905	-1,7399	-1,92904	-1,51175	1,797158	1,130445	0,184225	-0,30613	-0,3866	0,111942	1,001184	1,193059	1,074761	0,014183	0,120446
NCI_ATM_Pathway_(G1_S_transition_checkpoint	0,116595	-0,28727	-0,32818	0,591518	1,771533	0,903289	1,616284	2,126956	0,373425	-0,59434	-0,68683	0,001398	-0,00031	-0,18562	-0,00875	-4,41678	-2,03681	-1,7103	0,014308	0,121113
ATM_Pathway_Cell_Survival	0,36893	-0,21109	-0,13326	1,139603	1,374421	0,820782	-0,54988	-0,02897	-0,96039	-0,23215	0,564696	-0,11175	0,187594	-0,22653	0,293839	-2,29726	-1,53947	-1,31769	0,014379	0,121386
biocarta_fas_signaling_pathway_cd95__Main_Pat	0,182262	0,135046	0,06881	-0,20834	0,293191	0,120221	0,563288	0,986593	0,326382	-0,66924	-1,04979	-1,10602	0,160885	0,248639	0,145809	-3,16026	-1,87726	-1,20755	0,016712	0,121401
biocarta_granzyme_a_mediated_apoptosis_Pathv	0,030353	0,164482	0,157429	0,235492	0,391795	-0,20609	0,224241	0,33765	0,244536	-0,7671	-0,35411	-0,34474	0,24536	0,220347	0,358042	-2,07551	-0,96067	-0,55578	0,0153	0,121401
biocarta_granzyme_a_mediated_apoptosis_Pathv	0,030353	0,164482	0,157429	0,235492	0,391795	-0,20609	0,224241	0,33765	0,244536	-0,7671	-0,35411	-0,34474	0,24536	0,220347	0,358042	-2,07551	-0,96067	-0,55578	0,0153	0,121401
biocarta_granzyme_a_mediated_apoptosis_Pathv	0,030353	0,164482	0,157429	0,235492	0,391795	-0,20609	0,224241	0,33765	0,244536	-0,7671	-0,35411	-0,34474	0,24536	0,220347	0,358042	-2,07551	-0,96067	-0,55578	0,0153	0,121401
biocarta_il_2_receptor_beta_chain_in_t_cell_acti	0,00449	-0,17422	-0,22729	0,700079	0,277798	0,05357	-0,41962	-0,29106	-0,62495	-0,21312	0,367789	-0,01446	-0,07812	0,096463	0,279234	-1,29747	-0,55573	-0,80479	0,017028	0,121401
biocarta_il_4_signaling_Pathway_(cell_survival)	0,056367	0,401077	0,262725	-0,70848	0,461131	0,325869	0,160483	0,141409	0,664166	1,390788	0,700279	1,146671	0,225186	-0,28922	-0,16519	0,492574	1,127164	0,231784	0,01685	0,121401
biocarta_regulation_of_transcriptional_activity_b	0,024025	-0,14557	-0,10572	0,574266	0,516253	0,244586	-0,34677	-0,01031	-0,3803	0,326339	-0,89068	-0,08688	0,110998	0,042134	-0,00921	-2,08522	-1,58684	-0,73009	0,015914	0,121401
biocarta_regulation_of_transcriptional_activity_b	0,024025	-0,14557	-0,10572	0,574266	0,516253	0,244586	-0,34677	-0,01031	-0,3803	0,326339	-0,89068	-0,08688	0,110998	0,042134	-0,00921	-2,08522	-1,58684	-0,73009	0,015914	0,121401
biocarta_sonic_hedgehog_receptor_ptc1_regulat	0,32248	-0,07674	-0,19408	0,147736	1,413506	0,103482	0,035594	0,578245	-0,2324	-2,16995	-0,86247	-1,3544	-0,15547	-0,20813	-0,61806	-2,75409	-1,3183	-1,79127	0,014752	0,121401
biocarta_sonic_hedgehog_receptor_ptc1_regulat	0,32248	-0,07674	-0,19408	0,147736	1,413506	0,103482	0,035594	0,578245	-0,2324	-2,16995	-0,86247	-1,3544	-0,15547	-0,20813	-0,61806	-2,75409	-1,3183	-1,79127	0,014752	0,121401
biocarta_stathmin_and_breast_cancer_resistance	0,04465	-0,26926	-0,30609	1,439739	1,322422	1,721727	-1,63589	-1,57014	-1,45002	-2,48985	-2,28748	-2,30729	-1,21177	-0,87837	-0,98445	-4,09664	-2,90488	-2,26297	0,01523	0,121401
biocarta_the_prc2_complex_sets_long_term_gen	0,07647	-0,05265	-0,14418	-0,01436	0,045574	0,00694	1,720996	1,891785	1,380216	-1,07434	-0,61285	-1,01292	-0,05968	0,02286	-0,05034	-3,18392	-2,05048	-2,01224	0,016488	0,121401
biocarta_the_prc2_complex_sets_long_term_gen	0,07647	-0,05265	-0,14418	-0,01436	0,045574	0,00694	1,720996	1,891785	1,380216	-1,07434	-0,61285	-1,01292	-0,05968	0,02286	-0,05034	-3,18392	-2,05048	-2,01224	0,016488	0,121401
biocarta_the_prc2_complex_sets_long_term_gen	0,07647	-0,05265	-0,14418	-0,01436	0,045574	0,00694	1,720996	1,891785	1,380216	-1,07434	-0,61285	-1,01292	-0,05968	0,02286	-0,05034	-3,18392	-2,05048	-2,01224	0,016488	0,121401
biocarta_transcription_factor_creb_and_its_extra	0,116096	-0,28883	-0,51872	-0,34265	1,104059	1,109945	-1,28424	-1,03466	-2,03007	-0,83117	0,406772	-1,54777	-0,48885	0,174944	0,306414	-4,02002	-2,51692	-2,02445	0,015063	0,121401
Chemotaxis_Driven_by_IL-8_and_LTB4	-0,35679	-0,54969	-0,5619	2,435585	1,887218	1,455789	-2,79742	-3,26132	-3,26073	-4,07725	1,51579	-0,06629	-0,58716	-0,36064	0,212138	-7,69321	-6,29212	-4,6599	0,016061	0,121401
Erythropoietin_Pathway_BCLXL_Gene_Expression	0,047814	0,007449	0,030407	-0,90963	-0,21188	-0,28622	0,316043	0,564588	0,337355	1,594228	-0,38444	0,007948	-0,4356	-0,83895	-0,646	-0,39606	0,241948	-0,29036	0,016232	0,121401
Extracellular_Matrix_Remodeling_during_Adhesic	-0,23498	-0,22797	0,118668	-3,90117	-2,76851	-2,40434	-2,06889	-2,62162	-1,24536	-0,11944	-0,18684	-0,68729	-1,13976	0,057298	-1,4865	-0,01584	0,324632	1,11704	0,016705	0,121401
Glucocorticoid_Receptor_Signaling_Pathway_Infla	0,641238	-0,24386	-0,00237	0,269268	2,490608	0,03928	4,36103	4,875862	2,523026	-5,35484	-2,62874	-1,76831	-1,77466	0,788021	0,557925	-8,598	-6,11913	-2,60331	0,015252	0,121401
KEGG_Ether_lipid_metabolism_Main_Pathway	-0,13129	-0,37662	-0,08841	-0,52898	-2,01832	-1,35685	-3,9821	-4,54237	-2,86727	0,445246	-2,10688	-0,07934	-1,52821	-0,31564	-1,6702	2,054134	3,910805	2,69236	0,015101	0,121401
KEGG_Fatty_acid_biosynthesis_Main_Pathway	0,053612	0,004849	-0,01299	-0,17981	0,032633	0,310232	0,061087	0,134319	-0,11814	-0,90253	0,083081	-0,45191	-0,05205	0,079919	0,124907	-0,9515	-1,13341	-0,84089	0,016631	0,121401

KEGG_Homologous_recombination_Main_Pathwa	0,442907	0,008429	0,183745	1,454926	0,459744	1,531704	3,388365	4,324341	2,616967	-2,4398	-1,41216	0,163712	-0,57137	0,100744	-0,14265	-3,76407	-2,59311	-1,41424	0,017085	0,121401
KEGG_Wnt_signaling_Main_Pathway	-2,07056	-1,37017	-1,33818	0,799085	-1,61721	0,13486	-3,29855	-3,08859	-2,23714	-0,62409	-0,38229	0,065152	0,163628	1,396775	0,657082	-3,89875	-1,97437	-0,72453	0,015643	0,121401
NCI_Aurora_B_signaling_Pathway_(cytokinesis)	0,117952	-0,11485	-0,1764	-0,34265	0,004726	0,194318	2,051833	2,111241	1,631586	-2,42859	-2,23496	-2,46444	-0,4271	-0,64235	-1,0139	-2,91095	-2,24254	-1,90028	0,016744	0,121401
NCI_CD40_CD40L_signaling_Pathway_(B_cell_pro	-0,0273	0,050749	-0,00609	0,160625	0,076526	0,094419	-0,01663	0,059532	-0,10464	-0,25754	0,145888	-0,46914	-0,0424	0,12099	-0,08117	-0,59353	-0,39306	-0,43857	0,01652	0,121401
NCI_CD40_CD40L_signaling_Pathway_(regulation_	-0,0273	0,050749	-0,00609	0,160625	0,076526	0,094419	-0,01663	0,059532	-0,10464	-0,25754	0,145888	-0,46914	-0,0424	0,12099	-0,08117	-0,59353	-0,39306	-0,43857	0,01652	0,121401
NCI_EPHA_forward_signaling_Pathway_(actin_fila	-0,06268	0,150857	0,081693	-1,34405	-2,19501	-2,16625	-0,60026	-0,86923	-0,63794	0,812899	0,510617	0,288612	-0,1493	-0,0394	0,202333	1,11541	1,246241	1,461152	0,014802	0,121401
NCI_EPHA_forward_signaling_Pathway_(axon_gu	-0,24359	0,034564	-0,11344	-0,9419	-2,25849	-1,80955	-1,34795	-1,74288	-1,43431	1,580802	0,941789	0,369375	-0,30641	0,112994	0,410175	0,717691	1,668831	1,301321	0,017031	0,121401
NCI_EPHA_forward_signaling_Pathway_(cell_cell_	-0,3092	0,051778	-0,05062	-1,80664	-2,4757	-2,49003	-1,40835	-1,8619	-1,21133	1,819594	1,373338	0,5423	-0,08639	0,19057	0,406348	1,29155	1,696986	1,459648	0,016098	0,121401
NCI_EPHA_forward_signaling_Pathway_(regulatic	-0,3092	0,051778	-0,05062	-1,80664	-2,4757	-2,49003	-1,40835	-1,8619	-1,21133	1,819594	1,373338	0,5423	-0,08639	0,19057	0,406348	1,29155	1,696986	1,459648	0,016098	0,121401
NCI_Fc_epsilon_receptor_I_signaling_in_mast_ce	-0,05319	-0,04096	0,015514	0,034138	-0,03124	0,358573	0,443835	0,378273	0,552773	0,71933	0,720765	0,640354	-0,18439	-0,1659	-0,14654	0,666722	0,179132	0,476403	0,015736	0,121401
NCI_FOXM1_transcription_factor_network_Main_	0,17255	-0,0695	-0,05537	1,305093	0,978344	0,475277	-0,10312	0,255613	-0,89095	-0,70036	-0,62924	-0,51122	-0,64262	0,020664	0,027807	-3,68816	-1,99436	-1,76813	0,016965	0,121401
NCI_Netrin_mediated_signaling_events_Pathway_	-0,04158	-0,15526	-0,16728	-0,74755	-0,54261	-0,73948	-0,65229	-0,57929	-0,76169	-1,86579	-1,02106	-1,3259	0,491334	0,471628	0,502788	-2,09883	-2,16994	-2,0877	0,014937	0,121401
NCI_Nongenotropic_Androgen_signaling_Main_P	-0,09083	-0,425	-0,51706	0,162517	0,250742	-0,6466	-0,21342	0,275868	-0,67046	-2,27034	-0,68604	-0,50691	-0,00874	-0,02666	0,367207	-4,91629	-4,51758	-2,594	0,017085	0,121401
NCI_p75_NTR_mediated_signaling_Main_Pathwa	0,43018	-0,62477	-0,30417	0,367333	-0,42383	0,140619	-5,5705	-4,65012	-6,95221	-0,92126	-0,95794	-3,53119	1,675191	0,089031	1,55538	-10,1134	-6,83356	-7,50434	0,015355	0,121401
NCI_Plasma_membrane_estrogen_receptor_signa	-0,16548	-0,25704	-0,1287	-0,1777	0,113134	-0,34798	-0,88374	-0,93548	-0,77211	-1,21372	-0,47469	-0,70262	0,195343	-0,28335	-0,08574	-0,5219	-0,47767	-0,43296	0,016215	0,121401
NCI_PLK1_signaling_events_Pathway_(microtubu	0,161306	0,033745	0,139577	-0,04638	-0,29858	-0,17215	1,676283	1,876753	1,202763	-1,00452	-1,12156	-1,27907	-0,05716	0,165178	0,090105	-2,09312	-1,76931	-1,3598	0,014944	0,121401
NCI_PLK1_signaling_events_Pathway_(regulation_	0,263421	0,06869	0,111927	-0,12595	-0,2759	-0,1226	2,561128	2,90196	1,829673	-2,83535	-3,4413	-2,51798	-0,36085	-0,01699	-0,33492	-3,48496	-2,3078	-1,75149	0,015709	0,121401
NCI_S1P2_Main_Pathway	-0,06888	-0,49703	-0,66254	0,859571	0,368017	0,124729	-1,03093	-0,85165	-1,05628	-1,65189	-0,21919	-0,12278	-0,37476	-0,68097	-0,02992	-3,34235	-3,16853	-3,371	0,01707	0,121401
NCI_Signaling_events_mediated_by_HDAC_Class_	-0,29215	-0,33348	-0,51294	-0,43727	0,458697	-0,73778	-1,38154	-1,38218	-2,27503	-3,6241	-2,72645	-0,32283	0,287796	0,515964	1,167533	-10,0685	-6,79095	-4,95759	0,016333	0,121401
NCI_Signaling_events_mediated_by_TCPTP_Pathw	0,097094	-0,07661	-0,10024	-0,02711	-0,46756	-0,42183	-0,56514	-0,37763	-1,0321	-1,55336	-0,29203	-0,40638	0,645772	0,889371	0,58808	-2,60428	-2,18628	-1,45896	0,015348	0,121401
NCI_Stabilization_and_expansion_of_the_E_cadh	-0,35087	-0,00233	-0,21109	1,692367	0,644221	0,401937	-1,42945	-1,89734	-1,871	-1,66529	0,185322	-0,866	-0,59793	0,438725	0,319318	-6,08897	-3,14503	-3,11458	0,015192	0,121401
NCI_TGF_beta_receptor_signaling_Pathway_(JNK	-0,04862	-0,17	-0,09149	-0,23545	-0,2086	-0,37147	-0,54307	-0,3082	-0,80546	-1,04743	-0,74148	-0,9279	-0,00021	-0,01727	-0,07387	-1	-0,69685	-0,35993	0,016826	0,121401
RANK_Signaling_in_Osteoclasts_Pathway	-0,34335	-1,13573	-1,0863	-0,52416	-1,74902	-1,44484	-3,7064	-2,38592	-4,90821	-2,02218	-3,59498	-0,82002	1,277514	1,025715	2,35441	-6,68458	-4,74264	-2,37331	0,016253	0,121401
reactome_Abortive_elongation_of_HIV_1_transcr	0,346505	-0,22322	-0,33395	0,664592	0,238312	0,14325	-0,07862	0,376862	-0,74287	-2,62806	-2,07708	-0,6182	-0,57071	-0,42494	-0,08289	-5,62791	-4,91325	-3,68587	0,016858	0,121401
reactome_Adrenaline_signalling_through_Alpha_	-0,14743	-0,01118	-0,04701	-0,27787	0,0216	-0,27157	0,087734	-0,09898	0,190002	-0,0332	0,212051	-0,04438	-0,00447	-0,17265	-0,15211	0,183164	0,265508	0,185358	0,014478	0,121401
reactome_Butyrate_Response_Factor_1_BRF1_de	0,13936	-0,28344	-0,15482	0,940122	0,098269	0,080138	-0,89214	-0,60064	-1,52012	-2,33668	-0,53134	-1,33187	0,120012	-0,03373	0,293389	-6,86453	-5,08753	-5,44067	0,016743	0,121401
reactome_Cyclin_D_associated_events_in_G1_Ma	0,473142	-0,29193	-0,34845	0,265915	1,062126	0,057272	1,239148	1,958107	-0,53241	-4,69092	-1,20014	-0,69713	0,770829	1,145843	1,214921	-11,9867	-9,50274	-6,97298	0,015972	0,121401
reactome_Cytosolic_iron_sulfur_cluster_assembly	0,143021	-0,14751	0,046249	0,184258	0,49112	-0,22052	0,190829	0,252053	0,137608	-0,87243	-2,32849	-0,10509	0,410748	0,457477	0,55012	-0,06397	-1,17657	-0,33395	0,014483	0,121401
reactome_Downregulation_of_SMAD2_3_SMAD4	0,074847	-0,17354	-0,30963	0,220709	0,665774	0,195569	1,767042	2,301775	0,959428	-1,13441	0,39938	-0,14117	1,144138	1,169426	1,061398	-6,1068	-3,57807	-2,56894	0,014929	0,121401
reactome_Formation_of_HIV_elongation_comple	1,035391	-0,21334	-0,43185	1,489198	1,295366	0,774078	-1,1444	0,050929	-2,69844	-3,51706	-1,85223	-0,50123	0,013735	0,215671	0,343316	-10,1203	-8,76036	-6,39107	0,016402	0,121401
reactome_Formation_of_RNA_Pol_II_elongation_	1,035391	-0,21334	-0,43185	1,489198	1,295366	0,774078	-1,1444	0,050929	-2,69844	-3,51706	-1,85223	-0,50123	0,013735	0,215671	0,343316	-10,1203	-8,76036	-6,39107	0,016402	0,121401
reactome_G_alpha_i_signalling_events_Main_Pat	-4,97783	0,707155	1,07292	4,094439	4,775009	1,196824	-0,30873	-6,6373	7,300131	16,29039	11,01653	9,376979	-2,45002	-3,33274	-3,74779	27,88026	25,97031	19,35888	0,016957	0,121401
reactome_Inactivation_of_Cdc42_and_Rac_Main_	-0,04124	-0,23678	-0,18087	-1,27024	-1,48183	-1,28597	-2,03071	-1,73787	-2,26881	-0,49425	-0,10239	-0,24754	-0,94189	-0,10175	-0,06966	0,239156	0,237055	1,045051	0,01633	0,121401
reactome_ISG15_antiviral_mechanism_Main_Pat	1,017519	-0,10491	0,026762	1,054416	1,698727	-0,20542	1,010563	2,325059	-1,7767	-9,40455	-1,95048	-4,4096	0,53664	0,267296	0,764088	-19,7119	-13,9897	-11,2623	0,016802	0,121401
reactome_KSRP_destabilizes_mRNA_Main_Pathw	0,36932	-0,16738	-0,19065	1,142247	0,726522	-0,13986	0,127574	0,386538	-0,72069	-2,64411	0,005646	-0,98204	0,203689	-0,00355	0,603097	-6,44676	-4,91273	-5,17243	0,0165	0,121401
reactome_Mismatch_repair_MMR_directed_by_I	0,39916	0,187464	0,13378	0,86844	0,692426	0,695997	3,126284	3,521515	2,835525	-1,68812	-0,38463	-0,22643	-0,05032	0,060821	0,090884	-3,4759	-3,34611	-2,60348	0,015689	0,121401
reactome_Mitotic_Prometaphase_Main_Pathway	1,941304	0,025181	-0,06529	2,433072	2,844146	1,373782	10,90001	12,95751	6,3193	-12,9489	-5,0737	-8,88246	-2,53464	-1,31069	-1,20012	-25,4162	-17,2564	-13,0802	0,016607	0,121401
reactome_NOTCH1_Intracellular_Domain_Regula	0,297726	-0,28027	-0,33182	-0,31599	-0,61364	-0,58629	-0,78358	-0,47783	-1,82325	-2,23063	-1,53339	-2,62977	0,617964	0,126023	1,231171	-7,93398	-6,29619	-4,05915	0,017109	0,121401
reactome_Phosphorylation_of_CD3_and_TCR_zet	-0,77074	0,594807	0,054483	-0,58402	0,439271	1,253908	-0,02387	-0,61367	1,451013	1,401797	1,638954	2,328867	-0,38797	-0,47629	-0,47734	4,657776	3,663431	4,274996	0,014917	0,121401
reactome_Post_chaperonin_tubulin_folding_Mai	-0,0142	-0,35234	-0,17893	0,023141	0,180988	-0,57647	-1,53124	-1,54488	-1,60267	-2,16954	-1,92773	-0,86165	0,002136	-0,46489	0,070624	-4,98494	-4,16112	-3,321	0,015337	0,121401
reactome_Regulation_of_IFNG_signaling_Main_P	0,218508	-0,14567	-0,08885	-0,11511	1,056118	0,322298	-0,04933	-0,2301	-0,26498	-1,4001	-0,34899	-0,17312	-0,30851	0,590321	0,552349	-3,12334	-2,21894	-0,4467	0,016451	0,121401
reactome_Response_to_elevated_platelet_cytosc	-0,05704	-0,14828	0,076285	-0,41728	-0,45086	-0,88266	-0,09467	0,007198	0,069594	0,533945	-0,11509	-0,50209	-0,2843	-0,24978	0,152349	1,060825	1,053431	1,257453	0,016016	0,121401
reactome_RNA_Polymerase_II_Transcription_Elor	0,919513	-0,18974	-0,40438	1,268501	1,119261	0,455763	-0,9725	0,139264	-2,41162	-3,47003	-1,57558	-0,46558	0,040395	0,434839	0,507143	-9,62039	-8,09828	-5,9059	0,016667	0,121401
reactome_Signaling_by_NOTCH4_Main_Pathway	0,138662	0,164276	-0,00722	-0,36347	0,979943	0,353164	-1,01528	-0,92931	-1,03092	-0,64503	0,535642	-0,26292	0,854863	1,088783	0,852495	-1,39262	-0,0353	-0,40571	0,014803	0,121401
reactome_Synthesis_of_bile_acids_and_bile_salts	-0,20933	0,065224	0,191486	-1,28538	-0,57654	-2,0381	-1,8044	-2,60163	-1,708	0,384166	0,866001	0,917714	0,527109	-0,04816	0,371553	2,88012	2,008111	1,911106	0,016677	0,121401
reactome_Synthesis_of_very_long_chain_fatty_ac	0,090511	0,209213	-0,09445	-1,72574	-2,4038	-3,06289	-0,16658	-0,38273	-0,8594	3,568077	1,298617	0,702393	-0,53481	-1,04095	-0,38428	-2,26062	-0,17676	-0,06897	0,014897	0,121401
reactome_Transcriptional_activation_of_mitocho	0,159311	-0,26338	-0,28156	-0,27145	-1,81727	0,431917	-0,26859	0,423926	-1,1828	-2,46692	-1,47592	-1,42704	0,630465	0,401413	1,434485	-5,45242	-4,14354	-2,93532	0,015418	0,121401
VEGFR2_Signaling	-0,07169	-1,56163	-1,31622	-1,27731	1,698623	-0,09225	-5,94796	-3,88533	-7,50674	0,268541	-1,00789	-1,77086	1,954548	0,660876	2,523452	-9,89952	-5,45813	-1,97966	0,015454	0,121401
WNT_Pathway_Gene_Expression_via_CREB3	0,124734	-0,72412	-0,80569	2,687825	3,068213	1,19024	-1,82612	-1,66156	-2,62591	-3,89227	1,48375	-1,66587	0,046137	-0,6835	0,506734	-3,07686	-2,89763	0,200229	0,015359	0,121401
NCI_Neurotrophic_factor_mediated_Trk_recepto	0,031254	-0,36038	-0,3041	-0,91271	-0,6548	0,006267	-2,7911	-2,72606	-3,13894	-1,73399	-1,41063	-2,53644	0,23235	0,5148	0,156269	-1,34235	-0,74296	-1,20839	0,017357	0,122583
reactome_Integrin_alphaIIb_beta3_signaling_Mai	-0,39157	-0,30627	-0,2722	-1,2863	-1,19393	-1,53669	-0,80192	-1,12318	-0,67562	-0,55614	0,380029	-1,64463	-0,44693	-0,40558	-1,19622	0,717781	-0,09767	0,473529	0,017383	0,122583
reactome_Telomere_C_strand_Lagging_Strand_S	0,183191	0,055898	0,077381	-0,00424	0,357256	0,296916	0,958425	1,009804	0,822916	-1,37911	-0,59904	-0,54878	0,062879	-0,10343	-0,24042	-0,61137	-0,91559	-0,53135	0,017394	0,122583
reactome_RNA_Polymerase_I_Promoter_Escape_	0,729896	-0,06964	-0,28343	2,014962	1,454178	0,154341	-2,67581	-1,69168	-4,28109	-2,31338	-1,05702	-0,27368	0,7291	0,760573	0,992343	-10,7191	-9,00844	-7,94312	0,017439	0,122623
KEGG_Maturity_onset_diabetes_of_the_young_N	-0,03911	-0,04215	-0,01135	0,012494	-0,0155	0,027492	-0,01539	0,004513	0,101975	0,45421	0,199354	0,393614	-0,0352	-0,09594	-0,02283	0,303772	0,308425	0,016521	0,017629	0,1234
reactome_Regulation_of_gene_expression_in_lat	-0,03911	-0,04215	-0,01135	0,012494	-0,0155	0,027492	-0,01539	0,004513	0,101975	0,45421	0,199354	0,393614	-0,0352	-0,09594	-0,02283	0,303772	0,308425	0,016521	0,017629	0,1234
reactome_RNA_Polymerase_III_Chain_Elongation	0,100924	-0,16139	-0,43705	0,265774	-0,04805	-0,68381	-0,32709	0,237735	-1,32184	-1,80997	-0,92747	-1,65896	0,32005	0,501189	0,415488	-6,87859	-5,7123	-4,74845	0,017694	0,123577
JAK-STAT_Pathway_NmI_SOCS_BCL-XL_p21_Myc_	-3,64479	-1,18921	-0,06367	-11,2212	-5,28921	-7,43069	-10,3057	-14,5068	-2,22722	14,28711	-0,24953	0,452967	0,13768	-1,60027	-2,37499	38,04896	33,38059	29,00369	0,017754	0,123675
KEGG_Calcium_signaling_Main_Pathway	-0,25136	-0,30924	-0,04037	-5,80803	-3,41562	-7,0499	-3,50503	-4,48241	-1,73457	5,076169	1,204394	1,514491	1,734953	0,867218	1,624449	9,498583	9,700175	11,10353	0,017787	0,123675
NCI_Endogenous_TLR_signaling_Pathway_(regula	0,027273	-0,11235	-0,14572	-1,00148	-1,14381	-0,79292	-2,58013	-2,30877	-2,6328	1,451665	1,73881	1,254953	-0,58746	-1,29171	-0,69342	-0,73118	-1,14121	-0,95528	0,018043	0,123799
reactome_Formation_of_HIV_1_elongation_comp	0,99964	-0,20327	-0,44077	1,61505	1,356627	0,779641	-1,01352	0,146789	-2,48033	-3,37183	-1,44374	-0,20575	-0,06572	0,170846	0,309686	-9,70956	-8,55275	-6,25331	0,018083	0,123799
reactome_Neurofascin_interactions_Main_Pathw	-0,05856	-0,01988	-0,00739	-0,68757	-0,81177	-0,88977	-1,00174	-0,92949	-1,1834	-0,83669	0,261434	-0,70488	-0,55081	-0,41349	-0,34847	1,116454	1,527169	2,084449	0,017889	0,123799
reactome_RNA_Polymerase_I_Transcription_Initi	0,901802	-0,15863	-0,6072	1,430508	0,954917	-0,72742	-1,21702	-0,14801	-3,60932	-5,49026	-2,15772	-1,28711	0,636249	1,144676	1,009503	-15,2239	-12,7371	-10,9692	0,018013	0,123799
reactome_Serotonin_receptors_Main_Pathway	-0,48796	-0,24375	0,072384	0,567668	-0,26972	-0,29213	0,226452	-0,10915	1,574717	1,459351	0,109243	1,540087	-0,21456	-0,59909	-0,45976	3,005984	1,33761	2,049011	0,018077	0,123799
reactome_TCF_dependent_signaling_in_response	-0,20695	-0,38113	-0,24735	1,484855	0,134158	1,252665	1,608447	1,561798	1,491253	-1,12846	-0,03396	-0,51405	0,093366	0,330401	0,177566	-0,23762	0,234786	0,03222	0,01805	0,123799
reactome_Tristetraprolin_TTP_destabilizes_mRN/	0,095162	-0,32003	-0,15061	1,370799	-0,37949	0,298347	-1,01842	-0,56799	-1,53775	-2,2768	-0,56633	-1,257	0,114895	-0,15264	0,435334	-7,37963	-5,76218	-5,54085	0,017913	0,123799
biocarta_chaperones_modulate_interferon_signa	0,005813	-0,14227	-0,07978	0,262683	-0,27957	0,50588	0,257612	0,338365	-0,01379	-0,02205	-0,14424	-0,41902	0,063631	-0,01903	0,522011	-1,95305	-1,34913	-0,80178	0,018309	0,124185
biocarta_segmentation_clock_Main_Pathway	0,12007	0,141405	0,011149	0,112151	0,348285	0,218059	-1,16248	-1,25063	-1,45618	-0,16286	0,005401	-0,7839	0,337186	0,402307	0,308578	0,098515	-0,36318	0,414819	0,018559	0,124185
inositol_pyrophosphates_biosynthesis	-0,09919	-0,19058	-0,11203	-0,31371	0,559142	-0,23568	-1,23715	-1,23376	-1,67969	0,30228	-1,34617	-0,69893	0,016121	0,26911	0,155551	-0,64163	-1,54618	-0,96558	0,018519	0,124185
KEGG_Intestinal_immune_network_for_IgA_prod	0,020806	-0,31488	-0,10701	-0,25742	-0,61578	-0,49949	-0,35131	-0,09839	0,137972	0,481284	0,661863	0,727033	0,10007	0,072827	-0,14016	0,410507	0,30437	-0,03034	0,018334	0,124185
NCI_Signaling_events_mediated_by_HDAC_Class_	-0,10449	0,011017	-0,20345	-0,29277	-0,33848	0,051504	1,246107	1,340328	0,907942	-2,45032	0,064897	-0,96432	0,251344	0,230127	0,426368	-4,02067	-2,95432	-2,78091	0,018605	0,124185
reactome_Adenylate_cyclase_inhibitory_Main_Pa	-0,2409	-0,18123	-0,17182	0,081315	0,247536	-0,19592	-1,29086	-1,04908	-1,26931	-1,6441	-1,83012	-0,90207	0,20107	0,550265	0,495159	0,233761	-0,29067	0,21398	0,018657	0,124185
reactome_Adrenoceptors_Main_Pathway	-0,24234	-0,08725	0,075076	-0,44159	-0,76006	-1,57025	-0,1867	-0,67988	0,609304	-0,0442	0,372977	-0,67886	0,146438	-0,42489	-0,60028	0,845314	1,006094	0,574861	0,018689	0,124185
reactome_Cyclin_E_associated_events_during_G1	0,408333	-0,04579	-0,12447	2,092774	2,357238	0,387764	1,245076	1,804892	0,692302	-0,7269	-0,13722	-0,04953	-0,02221	0,195985	-0,13066	-5,41658	-4,14331	-3,45619	0,018238	0,124185
reactome_Resolution_of_AP_sites_via_the_multi	0,155885	0,020055	0,081832	0,030637	0,457223	0,293967	0,644299	0,674808	0,524244	-1,41037	-0,50505	-0,01467	0,131351	-0,15857	-0,12887	-0,81526	-1,0046	-0,80735	0,018371	0,124185
reactome_RNA_Polymerase_III_Transcription_Init	0,003099	-0,33246	-0,6585	0,121739	-0,23333	-0,92683	-0,87508	-0,10756	-2,20408	-2,76685	-1,39514	-1,80526	0,238606	0,493681	0,425165	-8,96908	-7,29375	-6,21088	0,018502	0,124185
reactome_SMAC_binds_to_IAPs_Main_Pathway	0,085296	-0,02662	0,08827	0,573871	-0,11996	0,527501	-0,14519	0,005664	-0,36153	0,042436	-1,03687	-0,92388	-0,04685	-0,30736	-0,03009	-1,35874	-0,94739	-1,02892	0,018698	0,124185
reactome_SMAC_mediated_dissociation_of_IAP_	0,085296	-0,02662	0,08827	0,573871	-0,11996	0,527501	-0,14519	0,005664	-0,36153	0,042436	-1,03687	-0,92388	-0,04685	-0,30736	-0,03009	-1,35874	-0,94739	-1,02892	0,018698	0,124185
reactome_Sodium_coupled_sulphate_di_and_tri_	-0,04396	0,133116	-0,09493	-0,3176	-0,10848	-0,12033	0,270493	0,093008	0,62608	0,263766	-0,64383	0,444956	-0,33567	-0,51241	-0,13017	0,90953	1,358014	0,93097	0,018263	0,124185
reactome_Tat_mediated_elongation_of_the_HIV_	0,883762	-0,17967	-0,4133	1,394353	1,180522	0,461326	-0,84162	0,235124	-2,1935	-3,3248	-1,16709	-0,1701	-0,03906	0,390015	0,473513	-9,20966	-7,89066	-5,76813	0,01849	0,124185
reactome_Receptor_ligand_binding_initiates_the	0,159889	0,106463	0,018304	-0,26747	0,459941	-0,01443	0,084157	0,217832	-0,02634	-1,03323	0,264178	-0,28918	0,641412	0,570782	0,701331	-2,94876	-2,19786	-1,44799	0,018746	0,12424
terminal_iOi-glycans_residues_modification	-0,06831	-0,37803	-0,24476	0,837818	0,046751	0,304978	-0,53366	-0,50838	-1,04605	0,213717	0,24048	-0,75991	0,030577	0,102845	0,509156	-1,53439	-3,25324	-1,58504	0,018845	0,124633
AHR_Pathway	0,340658	-0,17492	-0,18134	0,226865	-0,00791	0,674021	-1,51308	-0,66914	-2,50438	-0,7879	-1,64749	-2,40693	0,399491	0,532964	0,275576	-8,81885	-6,59528	-5,92124	0,019132	0,125203
NGF_Pathway_Gene_Expression_via_MYC_ELK1_	-0,31645	-0,31336	-0,2853	0,048285	0,383694	0,093586	-1,89744	-1,92494	-2,1539	-1,18484	0,698303	1,177471	1,074954	0,661165	1,494889	-0,91213	-1,47897	-0,59099	0,019078	0,125203
reactome_Phosphorylation_of_Emi1_Main_Pathw	0,00156	-0,1335	-0,22277	-0,12054	0,385986	-0,65838	1,483992	1,657474	1,227508	-2,12615	-1,90711	-1,33504	-0,3512	-0,16597	-0,24782	-1,4574	-1,04879	-0,71212	0,019132	0,125203
UDP-iNi-acetyl-D-galactosamine_biosynthesis_II	0,420362	0,16517	0,044679	-0,62669	0,321142	0,467908	-0,61383	-0,50209	-1,03145	-0,68216	-1,08787	-0,42926	0,063433	0,469422	0,436379	-2,4794	-2,30567	-2,13307	0,019049	0,125203
WNT_Pathway_PKC_Pathway	-0,0715	-0,68083	-0,6889	0,835521	1,612946	-0,81904	-1,45815	-1,24332	-1,84879	-3,65185	0,344936	-3,35641	0,185916	-0,60041	0,52923	-3,31024	-3,14948	0,42694	0,019027	0,125203
biocarta_erk_and_pi_3_kinase_are_necessary_fo	0,076806	-0,04805	0,01452	0,138793	-0,3228	-0,20067	-0,51421	-0,47569	-0,58539	0,033207	0,438291	0,006092	-0,0471	-0,04772	-0,05052	0,142537	0,292938	0,387334	0,019237	0,125472
biocarta_inhibition_of_cellular_proliferation_by_g	-0,10972	-0,55575	-0,50544	0,013394	0,809546	0,584441	-0,23075	0,356459	-1,02366	-1,19725	-0,00739	-0,22037	-0,32385	-0,39416	0,021908	-3,37837	-1,70028	-1,12131	0,019328	0,125472
KEGG_Linoleic_acid_metabolism_Main_Pathway	-0,23241	0,030069	0,053331	-0,06688	-0,85182	0,08997	-0,58205	-1,27342	1,208631	0,862923	-0,46907	1,611341	-0,55747	0,200915	-0,46592	2,380261	2,925587	1,101927	0,019375	0,125472
KEGG_Ubiquitin_mediated_proteolysis_Main_Pat	1,89502	-0,74284	-0,33997	-0,14488	-0,41721	-2,14	-6,63788	-3,85335	-12,4629	-12,005	-5,96732	-8,86282	1,643584	0,999982	3,377816	-35,3642	-28,0423	-20,7942	0,019367	0,125472
oleate_biosynthesis	-0,04997	0,028347	-0,26692	-0,84452	-1,52365	-0,70533	-0,24692	-0,31285	-0,31056	1,382159	0,878667	0,921596	-0,1354	0,124794	-0,25455	-0,06425	-0,10499	0,003519	0,019271	0,125472
KEGG_Transcriptional_misregulation_in_cancer_N	-2,84263	-3,40064	-2,58437	-2,71501	0,031683	-1,41752	-7,09552	-8,62883	-5,6943	-1,05081	-7,22424	-1,84391	5,24428	2,783218	5,431209	-3,23416	-3,82948	-1,81791	0,019448	0,125687
NCI_Regulation_of_Telomerase_Main_Pathway	0,478543	0,099118	-0,12432	0,453137	1,251862	-1,674	0,895174	1,284738	-0,51609	-3,73982	-2,38184	-2,58331	-0,45556	0,023457	0,291515	-8,78117	-7,16066	-4,90478	0,019534	0,125978
biocarta_p53_signaling_Main_Pathway	-0,0082	-0,06838	-0,1661	1,162734	1,012153	0,48484	0,84677	0,957216	0,526726	0,192278	-0,08108	-0,21602	-0,15147	0,017269	0,052433	-3,13279	-2,74881	-1,78906	0,019859	0,125987
JAK-STAT_Pathway	-3,66508	-1,68571	0,034896	-12,8196	-3,99803	-9,78222	-9,56731	-13,5937	-2,38259	14,83479	1,198106	6,567884	3,01512	1,536979	3,078319	31,71731	29,50802	25,40857	0,019846	0,125987
KEGG_Amoebiasis_Main_Pathway	0,155128	-0,07008	0,058335	-1,36113	-0,6619	-1,76093	-1,28204	-1,40208	-1,11083	1,967005	-0,34624	2,01389	-0,40448	-0,86499	-0,41936	-0,127	-1,08561	-0,61855	0,019688	0,125987
KEGG_Spliceosome_Main_Pathway	3,03994	-0,02067	0,23948	6,041015	5,065914	7,385674	3,473506	6,46542	-2,11876	-7,33769	-3,87037	-5,13431	0,866227	1,006844	2,160462	-45,2818	-35,2756	-27,9174	0,019823	0,125987
mRNA_capping	0,131554	-0,1671	-0,04945	0,366489	0,454632	0,069916	-0,19563	0,025686	-0,49279	0,01384	0,198565	0,410241	0,290041	0,287484	0,214176	-1,13153	-0,7972	-0,89633	0,019599	0,125987
NCI_PLK1_signaling_events_Pathway_(regulation_	0,159103	-0,00512	0,077764	-0,05925	-0,34794	-0,24421	1,533908	1,750683	1,069174	-0,74422	-0,95541	-1,1612	-0,06828	0,170563	0,073732	-1,7745	-1,50886	-1,00426	0,019813	0,125987
NGF_Pathway	-0,95298	-1,37622	-1,42625	-1,30403	1,102722	-0,33401	-3,98349	-3,34711	-6,12485	-1,83698	-0,27653	2,215989	2,358723	2,307488	3,158898	-11,8599	-8,92634	-5,46946	0,01979	0,125987
reactome_RNA_Polymerase_III_Transcription_Init	-0,02478	-0,61068	-0,76873	0,967698	0,507005	-0,02968	-1,44077	-0,71807	-2,75373	-2,51812	-0,57508	-0,87506	-0,0912	0,704844	0,198352	-10,116	-8,01073	-7,20975	0,019676	0,125987
reactome_negative_regulation_of_TCF_depender	-0,21216	-0,05912	0,076626	-1,24484	-0,35652	-1,25311	-0,69503	-0,66248	-0,09536	0,248196	0,169861	0,726119	0,276571	-0,3294	0,00311	0,973233	1,026864	0,450279	0,019906	0,126028
MAPK_Family_Pathway_Gene_Expression_via_AT	0,159025	-0,0729	-0,0998	-0,36968	-1,37187	0,474389	-0,19138	0,505371	-0,93526	-2,40217	-1,11936	-2,65816	1,000597	1,220052	1,801199	-1,85245	-2,25537	-0,39114	0,020144	0,127277
NCI_VEGFR1_specific_signals_Main_Pathway	-0,09745	-0,10801	-0,24405	-0,54316	-0,09079	-0,43721	-1,95838	-2,0369	-1,92846	-0,36245	-1,88983	-0,78173	0,286632	0,251358	0,09546	-1,43965	-1,20947	-0,63471	0,020293	0,12796
reactome_mRNA_Capping_Main_Pathway	0,938406	-0,18279	-0,33228	1,145719	1,403787	0,835911	-1,38387	-0,16101	-2,70908	-3,08599	-1,4499	0,218193	0,074398	0,25999	0,044898	-9,10709	-7,63821	-6,65188	0,020377	0,12823
iNi-acetylglucosamine_degradation_I	0,102638	0,07319	-0,03448	-0,2804	0,182695	0,129882	-0,68223	-0,56263	-0,81137	-0,71621	-0,21557	-0,04143	0,047681	0,186642	0,254898	-1,30791	-1,33536	-1,10375	0,020502	0,128257
NCI_Ephrin_B_reverse_signaling_Pathway_(cell_a	-0,21652	-0,13549	-0,3424	0,181566	1,460574	0,182273	-1,99624	-2,12738	-1,89052	-0,4672	-0,57491	-0,20411	0,217844	0,232949	0,55189	0,24054	-0,13479	0,030599	0,020546	0,128257
reactome_DCC_mediated_attractive_signaling_M	0,127283	-0,087	-0,30604	-0,51662	-0,07523	-0,57378	-0,89686	-0,76555	-1,31603	-2,86978	-0,67595	-1,30423	-0,2038	0,21222	0,154075	-4,2498	-3,81249	-3,22012	0,020545	0,128257
reactome_Regulation_of_APC_C_activators_betw	0,548462	-0,23282	-0,17057	-0,25641	-0,53646	-2,17795	1,286461	1,929201	-0,0948	-6,01514	-3,22914	-3,31562	-0,51352	0,140618	0,033147	-8,69097	-7,38564	-6,06311	0,020521	0,128257
AHR_Pathway_Cath_D_expression_via_SP1	0,285594	-0,01715	-0,04468	0,519027	0,478125	0,759256	-0,4468	0,070554	-0,92385	0,627761	-0,83066	-2,08184	0,305996	0,505453	-0,20324	-4,97176	-4,01487	-3,28879	0,020777	0,1294
NCI_ALK1_signaling_events_Pathway_(sprouting_	-0,09051	-0,05248	-0,1072	-0,56855	-0,31717	-0,35544	-0,56115	-0,63915	-0,72593	-0,18753	0,163941	0,378822	-0,1438	-0,11579	-0,10711	-0,03146	-0,2635	0,201674	0,020812	0,1294
reactome_RNA_Pol_II_CTD_phosphorylation_and	0,822528	-0,15919	-0,30482	0,925022	1,227682	0,517596	-1,21196	-0,07268	-2,42225	-3,03897	-1,17325	0,25384	0,101057	0,479158	0,208726	-8,60719	-6,97612	-6,1667	0,021022	0,130188
reactome_RNA_Polymerase_I_Transcription_Terr	0,886905	-0,03678	-0,25902	1,657747	1,210953	-0,07016	-2,55557	-1,58304	-4,16316	-1,9828	-1,20262	-0,09199	0,789876	0,930648	1,145232	-10,7468	-8,94959	-8,04747	0,020997	0,130188
reactome_Prostanoid_ligand_receptors_Main_Pa	-0,32147	0,139281	0,157778	-0,4703	-0,68967	0,045238	0,420672	-0,28216	0,980132	0,675544	-0,11956	0,18979	-0,03703	0,251689	-0,28445	1,75824	1,935408	1,341323	0,021096	0,130386
reactome_Resolution_of_Sister_Chromatid_Cohe	2,268337	0,081302	-0,02911	2,961228	2,852948	1,472445	12,54665	14,81127	7,234366	-13,5968	-4,41494	-8,90608	-2,94635	-1,44235	-1,52711	-28,1139	-19,0465	-13,7716	0,021205	0,130797
NCI_E2F_transcription_factor_network_Main_Pat	-0,01658	-0,09946	-0,01659	-0,10144	0,308897	-0,11627	1,546662	1,655179	1,097928	-0,02288	-0,40978	-0,65285	-0,19373	0,05135	0,203413	-1,70489	-1,28782	-1,2229	0,021502	0,131586
reactome_IRF3_mediated_activation_of_type_1_	0,326153	0,129205	0,085585	0,11521	0,785506	0,377395	-0,22853	-0,1949	-0,12713	-0,91929	-0,28822	-0,36686	0,265513	-0,13167	-0,09752	-0,84862	-0,60737	-0,28849	0,021454	0,131586
reactome_Mismatch_repair_MMR_directed_by_I	0,388755	0,128502	0,050762	0,682935	0,606402	0,654603	2,629236	3,080112	2,299079	-1,71504	-0,07912	0,044877	0,025644	0,194066	0,222855	-3,9023	-3,77056	-2,97416	0,021497	0,131586
reactome_Processing_of_Capped_Intron_Contain	0,835436	0,095051	-0,10666	1,589859	-0,50615	-0,09602	1,924019	2,851914	0,672774	-4,85313	-3,95618	-2,2118	-0,65465	-0,31464	0,033072	-12,3345	-9,5313	-7,38011	0,021376	0,131586
Akt_Signaling_Pathway_Aggregation_and_Neuroc	-0,10412	-0,07057	0,364371	-0,93897	0,72359	-1,21965	1,371475	1,146622	1,682396	0,619414	1,586718	-0,11157	0,258716	0,13637	-0,268	2,218375	2,0966	2,499837	0,021981	0,131608
ILK_Signaling_Pathway_Cytoskeletal_Adhesion_Co	-1,90771	-0,88878	-0,12501	-9,52615	-6,44291	-6,61156	-13,2829	-14,7223	-8,89591	3,316874	0,32788	2,91601	2,380058	0,823557	0,50692	13,31399	11,02923	9,193569	0,022076	0,131608
Mismatch_Repair_in_Eukaryotes_Pathway	0,56758	0,282244	0,239765	0,376026	0,912926	0,619673	3,909499	4,693715	3,20307	-2,81205	-0,86793	-0,30023	0,024339	0,443751	0,679864	-7,14641	-5,73188	-4,49087	0,021658	0,131608
NCI_Alpha_synuclein_signaling_Pathway_(Pathwa	0,078576	-0,02379	0,133652	-0,04908	0,14256	-0,37846	-1,1238	-1,13804	-1,11578	-0,27368	0,124571	0,480456	-0,15913	-0,10075	-0,36845	1,157755	0,418094	0,25001	0,022042	0,131608
NCI_Neurotrophic_factor_mediated_Trk_recepto	-0,03984	-0,22601	-0,09168	-0,11718	0,493139	0,900564	-1,35393	-1,62365	-1,73195	-0,15857	-0,05528	-0,23491	-0,01588	0,108207	0,216531	-1,01721	-0,18703	-0,16602	0,021782	0,131608
NCI_p73_transcription_factor_network_Main_Pat	-0,05123	-0,17746	-0,33416	-0,8786	0,186454	0,835142	-2,33529	-1,93301	-3,0821	-0,67935	-1,8134	-0,36521	-0,06319	-0,02865	-0,18971	-2,40667	-1,44324	-1,289	0,021754	0,131608
NCI_Signaling_events_mediated_by_VEGFR1_and	-0,00555	-0,14882	-0,11884	-0,05412	-0,05645	-0,17066	-0,32662	-0,2654	-0,21929	0,707224	0,515895	0,469864	0,071048	-0,08576	0,048575	0,391474	0,293723	0,132962	0,021724	0,131608
NCI_TRAIL_signaling_Main_Pathway	-0,09284	0,13284	-0,26161	0,083511	0,417997	1,267875	-0,97507	-0,9306	-1,19208	0,105826	0,850484	-0,23529	0,212277	0,323235	0,748496	-1,63816	-0,78823	-0,23885	0,022097	0,131608
reactome_A_third_proteolytic_cleavage_releases	0,028293	-0,0374	-0,03421	0,142835	0,389363	-0,22563	0,011015	0,100034	-0,19133	-0,61543	-0,11806	0,39493	0,453609	0,670977	0,583055	-1,40924	-0,06871	-0,20949	0,022096	0,131608
reactome_Formation_of_the_active_cofactor_UD	0,088099	-0,02585	-0,04643	-0,00837	0,168526	0,087769	-0,01951	0,059824	-0,27414	-0,83287	-0,382	-0,32793	-0,1138	0,021935	0,066557	-2,31011	-1,84723	-1,67672	0,021686	0,131608
reactome_Metabolism_of_polyamines_Main_Pat	0,107193	0,070891	0,068133	-0,03977	-0,23496	-0,0562	-0,1059	-0,11057	-0,25929	-0,91651	-0,27884	-0,04895	0,441509	0,274809	0,335963	-1,63259	-0,94488	-0,92345	0,021915	0,131608

reactome_Regulation_of_lipid_metabolism_by_P	-0,16506	-0,15187	-0,0921	0,533987	-1,23788	0,736603	0,08336	0,369175	-0,30515	-1,20895	-0,64412	-0,49308	0,833877	0,384787	1,198888	-1,57363	-0,96446	-1,13699	0,021883	0,131608
tyrosine_degradation	-0,04566	0,185541	-0,02733	-1,16833	-1,24515	-1,69993	0,368233	0,500349	0,616041	0,765216	-0,13623	0,368956	-0,37752	-0,05012	-0,44701	-0,29312	-0,45818	-0,34589	0,0218	0,131608
Ubiquitin-Proteasome_Dependent_Proteolysis_Pa	2,265665	0,481452	0,395151	0,083961	-2,62641	-2,87011	-2,7792	-0,70629	-8,44691	-16,0022	-3,58968	-6,90231	2,768747	4,478075	5,207812	-36,0998	-25,1598	-20,9838	0,022006	0,131608
biocarta_cell_cycle_g1_s_check_point_Main_Path	0,005043	-0,3757	-0,2156	1,713129	1,543145	-0,02576	1,391776	1,364196	0,995779	0,05176	-0,12773	-0,44198	-0,00317	-0,11518	-0,07233	-4,52914	-4,73154	-3,65485	0,022166	0,131771
reactome_Insulin_like_Growth_Factor_2_mRNA_	0,010841	-0,05139	-0,00079	-0,16643	0,338271	-0,09942	-1,35551	-1,39904	-1,42044	-0,23856	-0,25095	-0,18258	0,212066	-0,01683	0,118256	-1,19994	-1,40431	-1,28162	0,022384	0,132671
reactome_Synthesis_of_bile_acids_and_bile_salts	-0,10327	0,119523	0,055198	0,568108	-0,13272	-0,5945	-0,51092	-0,84912	-0,57653	1,232732	1,041507	0,026574	-0,34857	-0,56041	-0,31009	1,359054	-0,10457	0,476116	0,022403	0,132671
reactome_ABCA_transporters_in_lipid_homeosta	-0,43719	-0,376	-0,29136	-0,2383	0,039721	-0,58538	-0,97393	-1,12607	-0,68143	0,782865	0,11627	0,629858	-0,07469	-0,35337	-0,34691	1,76779	1,407339	0,941528	0,022468	0,132805
reactome_Sphingolipid_de_novo_biosynthesis_M	-0,004	-0,37431	-0,21986	-0,16335	1,21325	-0,1614	0,270239	0,53381	-0,21001	-0,59186	-0,22858	-0,97281	0,007784	-0,19046	0,479412	-4,02896	-3,7171	-1,4463	0,022656	0,133664
reactome_Cyclin_A_B1_associated_events_during	0,50241	-0,16819	-0,20853	0,586297	0,163002	-0,66179	0,894425	2,032129	0,213285	-3,10846	-2,92571	-2,01916	-0,40735	-0,39879	-0,72829	-7,52848	-4,55645	-3,58601	0,022773	0,134099
ATM_Pathway_Synaptic_Vesicle_Transport	0,026562	-0,07793	-0,09985	0,350871	0,689618	0,340865	-0,09767	0,083245	-0,24561	0,044175	0,24382	-0,02629	0,027761	-0,02649	0,026471	-0,74732	-0,60386	-0,30994	0,023014	0,135005
reactome_Sulfide_oxidation_to_sulfate_Main_Pa	0,016488	-0,03033	0,045345	-0,5561	0,003396	-0,13768	-0,00577	0,103815	-0,05918	-0,38092	0,207401	0,730311	-0,37318	-0,38865	-0,13638	0,160686	0,062454	-0,14311	0,023	0,135005
reactome_Repair_synthesis_for_gap_filling_by_D	0,335733	0,050385	0,144253	0,02798	0,803414	1,134415	3,267166	3,762054	2,878389	-0,86917	-1,31453	0,33836	-0,2063	0,102545	0,391793	-4,12863	-3,98581	-2,9432	0,023129	0,135175
reactome_Repair_synthesis_of_patch_27_30_bas	0,335733	0,050385	0,144253	0,02798	0,803414	1,134415	3,267166	3,762054	2,878389	-0,86917	-1,31453	0,33836	-0,2063	0,102545	0,391793	-4,12863	-3,98581	-2,9432	0,023129	0,135175
KEGG_Protein_export_Main_Pathway	0,577056	0,049329	0,056287	1,240589	1,21476	1,211989	-0,7042	0,149888	-1,71442	-4,31406	-0,14893	-0,23581	0,055081	-0,13955	0,080496	-9,16656	-7,63826	-5,9476	0,023211	0,135396
reactome_Nephrin_interactions_Main_Pathway	-0,06285	0,013462	-0,13063	-0,00043	0,939897	0,527388	-1,95829	-1,69083	-2,49627	-0,43262	0,444974	0,273282	0,726829	0,412266	0,823578	-4,56672	-2,59556	-2,321	0,023263	0,135446
reactome_Cytosolic_sensors_of_pathogen_associ	0,25056	-0,07424	-0,36423	0,379562	0,107701	-0,81781	-0,41033	0,246497	-1,58658	-2,1945	-1,15661	-1,82715	0,06849	0,319438	0,145288	-8,00073	-6,31245	-5,22548	0,023348	0,135454
reactome_Polymerase_switching_Main_Pathway	0,54432	0,28412	0,285057	0,338348	1,019631	0,753729	3,065279	3,457548	2,237272	-2,25166	-1,98889	-0,64763	-0,40312	-0,44874	-0,19697	-5,36289	-4,39017	-3,24061	0,023473	0,135454
reactome_Polymerase_switching_on_the_C_strar	0,54432	0,28412	0,285057	0,338348	1,019631	0,753729	3,065279	3,457548	2,237272	-2,25166	-1,98889	-0,64763	-0,40312	-0,44874	-0,19697	-5,36289	-4,39017	-3,24061	0,023473	0,135454
reactome_Regulation_of_IGF_Activity_by_IGFBP_	-0,89304	-0,29224	-0,24452	-1,31486	-1,66011	-3,2906	-3,58693	-4,44306	-2,43291	0,12204	1,183643	-0,05718	-0,33186	-0,1491	0,265949	2,597629	2,2525	1,778911	0,023364	0,135454
Role_of_NTN1_in_Axon_Pathfinding	-0,24027	-0,68617	-0,56826	1,140294	0,094366	1,265431	-2,58272	-2,10142	-3,79281	-3,95137	-0,1657	-2,69132	-1,59386	-1,12036	-0,04319	-10,5013	-7,65037	-5,6172	0,023482	0,135454
reactome_NrCAM_interactions_Main_Pathway	0,076145	-0,07491	0,045723	0,032941	-0,02198	0,557284	-1,93237	-1,80202	-2,20296	0,494089	-0,18196	-0,36256	0,557019	0,524892	0,680478	-0,95426	0,043262	0,183739	0,02357	0,135714
biocarta_il_3_signaling_Main_Pathway	-0,28974	-0,5581	-0,33365	-0,48467	-0,51805	-0,01573	-0,26197	-0,39296	-0,30986	0,405449	0,051831	-0,16632	-0,22834	-0,93585	-0,6459	0,397481	0,526316	-0,04541	0,023661	0,135833
HIF1Alpha_Pathway_HIF1a_Degradation	-0,0331	-0,01004	0,016009	-0,1486	-0,01893	-0,08579	-0,16465	-0,1423	-0,12675	0,06304	-0,03256	0,208771	-0,03163	0,082028	-0,03189	0,458169	0,500673	0,3287	0,023678	0,135833
AHR_Pathway_CYP1A1_CYP1B1_CYP1A2_AHRR_g	-0,01277	-0,09123	-0,05909	0,056734	-0,16746	0,185202	-0,4263	-0,24477	-0,61337	-0,58049	-0,38962	-0,03605	-0,07847	-0,05399	0,163709	-1,82368	-1,20763	-1,40118	0,023832	0,136157
reactome_Phosphorylation_of_proteins_involved	0,095575	-0,05911	-0,01619	0,697653	1,126119	-0,07353	1,543418	1,698998	1,328322	-0,00627	0,384428	-0,30273	-0,06373	0,215121	0,208266	-1,89414	-1,83056	-1,3331	0,023829	0,136157
reactome_Phosphorylation_of_proteins_involved	0,077337	-0,17133	-0,0693	-0,00551	0,217193	-0,2462	0,285182	0,399018	0,193918	-1,37812	-0,80404	-1,31282	-0,04006	-0,08543	-0,31085	-1,19576	-0,64323	-0,12984	0,023866	0,136157
NCI_Ras_signaling_in_the_CD4_TCR_Main_Pathw	-0,24981	-0,38397	-0,32721	-0,6399	-1,04347	-1,02596	-1,0641	-1,07355	-1,40884	-0,20617	0,553843	-0,53306	-0,5598	-0,26485	-0,12948	-0,40809	0,531429	0,572762	0,024057	0,136747
reactome_G_alpha_s_signalling_events_Main_Pa	-2,83127	0,150676	1,296291	-1,77622	-3,05292	-3,3694	3,097697	0,159773	7,366904	5,989516	0,733135	0,647799	0,781574	0,059736	-1,50247	19,83066	15,50107	13,44149	0,024056	0,136747
biocarta_chromatin_remodeling_by_hswi_snf_atp	-0,14125	-0,22867	-0,24305	0,078858	-0,28762	-0,20686	-0,09254	-0,00787	-0,32259	-0,91575	0,395286	0,470452	0,571595	0,666336	0,703112	-1,79838	-1,54113	-1,31132	0,025061	0,136958
biocarta_chromatin_remodeling_by_hswi_snf_atp	-0,14125	-0,22867	-0,24305	0,078858	-0,28762	-0,20686	-0,09254	-0,00787	-0,32259	-0,91575	0,395286	0,470452	0,571595	0,666336	0,703112	-1,79838	-1,54113	-1,31132	0,025061	0,136958
biocarta_il_2_signaling_Main_Pathway	-0,26006	0,106849	0,080827	-1,52365	0,023384	-1,29903	0,455024	-0,09368	0,711343	1,665108	0,597644	1,225451	-0,07433	-0,66713	-0,18013	-0,0544	-0,28176	-0,30224	0,024595	0,136958
dermatan_sulfate_biosynthesis_late_stages	-0,04573	0,127329	0,179364	-1,23344	-0,54501	-0,69011	-0,17699	-0,27584	-0,34534	0,259406	0,195971	-0,00816	-0,03402	-0,41687	0,022885	0,170733	0,251816	-0,12541	0,024409	0,136958
KEGG_Cytokine_cytokine_receptor_interaction_M	-0,97105	-0,05052	0,634897	-2,26301	0,679431	0,742606	2,301035	0,529991	3,23869	1,573333	1,397836	0,807492	-1,3155	0,011567	-0,43711	7,726306	6,533761	5,434467	0,024914	0,136958
KEGG_DNA_replication_Main_Pathway	0,98391	0,443379	0,44699	1,501175	1,435946	2,624909	7,90948	8,901416	6,567018	-3,63917	-2,2335	-2,28415	-1,43602	-0,88891	-0,46136	-11,0453	-8,87599	-7,18274	0,024637	0,136958
KEGG_Mismatch_repair_Main_Pathway	0,719218	0,271291	0,300779	1,369408	1,169121	1,449274	4,069369	4,937445	3,344305	-2,94021	-0,08999	-0,09888	-0,2008	0,173646	0,39544	-7,86204	-7,03365	-5,39299	0,025025	0,136958
NCI_FAS_CD95_signaling_Pathway_(cell_cycle)	0,021324	-0,04005	-0,00874	-0,06308	-0,06404	0,326995	-0,6304	-0,4668	-0,75581	-0,62216	-0,42353	-0,07418	-0,00611	-0,11292	-0,00123	-1,58568	-1,26046	-0,8305	0,024852	0,136958
NCI_S1P3_Main_Pathway	-0,06187	-0,56675	-0,38706	0,481761	0,578621	0,01147	-1,96996	-1,84246	-2,12988	0,248021	0,142246	-1,06271	-0,31865	-0,38811	0,626396	-3,64914	-2,79783	-2,75128	0,025059	0,136958
RANK_Signaling_in_Osteoclasts_Pathway_Resorp	-0,57783	-0,38861	-0,56969	0,384456	-1,49336	0,729516	-0,59032	0,073858	-0,96512	0,289856	-1,50528	0,288686	0,309442	0,619669	1,344071	-3,68857	-3,07096	-2,06739	0,024498	0,136958
reactome_Amyloids_Main_Pathway	-0,76783	-0,15127	-0,32488	-0,89615	-3,92561	-2,10077	3,60198	3,591853	4,05009	6,773837	2,702846	0,073576	-1,64661	-2,6111	-1,39444	-1,91125	-0,62786	-1,35381	0,024439	0,136958
reactome_Antigen_processing_Ubiquitination_Pr	3,05063	0,19069	-0,14405	-0,31826	1,332306	-2,63061	-8,71241	-6,17764	-17,4578	-16,0281	-6,67524	-9,80314	3,481237	2,913925	5,646549	-57,2924	-45,9569	-36,6646	0,024456	0,136958
reactome_CHL1_interactions_Main_Pathway	-0,08188	0,064393	0,034781	-0,40681	-0,29039	-0,06853	-0,61964	-0,57499	-0,56658	1,790322	2,261346	2,171273	0,158634	0,172074	0,120672	0,169492	0,679721	0,488815	0,024797	0,136958
reactome_Formation_of_the_Early_Elongation_C	0,911781	-0,17126	-0,3986	0,982438	1,245762	0,356222	-1,23772	-0,21265	-2,42214	-2,7912	-1,85005	0,130046	-0,24417	-0,02664	0,007175	-8,65162	-7,73494	-5,84435	0,024781	0,136958
reactome_Formation_of_the_HIV_1_Early_Elonga	0,911781	-0,17126	-0,3986	0,982438	1,245762	0,356222	-1,23772	-0,21265	-2,42214	-2,7912	-1,85005	0,130046	-0,24417	-0,02664	0,007175	-8,65162	-7,73494	-5,84435	0,024781	0,136958
reactome_Netrin_mediated_repulsion_signals_M	-0,27194	-0,32582	-0,19942	-0,70645	-0,25756	-0,09841	-2,24664	-2,32409	-2,10004	-1,24311	-0,47807	-0,99249	0,244479	0,152646	0,161772	-0,30893	-0,61097	-0,77233	0,024318	0,136958
reactome_temp_Immunoregulatory_interactions_	-3,21354	1,409367	1,363104	0,162511	3,892534	-0,55643	-0,05335	-4,92221	9,3031	10,1125	0,740128	6,876301	-1,34651	-2,7085	-1,84629	25,83129	17,80823	20,89311	0,024946	0,136958
reactome_Trafficking_of_GluR2_containing_AMP	0,059086	-0,0938	-0,10929	-0,47424	-0,10473	0,326785	-0,43112	-0,66034	-0,50253	0,952162	0,542887	0,173574	-0,16797	-0,06273	0,14231	0,482074	0,716551	0,63467	0,02459	0,136958
reactome_Vasopressin_regulates_renal_water_ho	-0,52715	-0,39556	-0,53622	1,633168	1,951159	-0,45862	-1,31598	-1,39187	-0,83848	-1,62976	0,718494	1,845855	0,401092	1,398684	1,667305	-1,83086	-1,83271	0,869321	0,024618	0,136958
reactome_VEGFA_VEGFR2_Pathway_Main_Pathv	-0,15191	-0,98163	-1,3022	-0,69388	-0,165	-1,68301	-4,15539	-3,31781	-5,272	-0,87567	-1,6676	-1,15929	1,151971	0,461106	1,159662	-9,55306	-7,28616	-5,21957	0,024985	0,136958
Slit-Robo_Pathway	-0,87972	-1,17126	-1,12141	0,641391	-2,50943	-1,23534	-5,19846	-5,20548	-5,49867	-7,93486	-2,89858	-1,68793	-0,60601	-1,52606	-0,61674	-10,6157	-7,21439	-5,61786	0,024633	0,136958
superpathway_of_melatonin_degradation	-0,28365	-0,19193	0,019725	-1,39286	0,018703	-0,85895	0,032455	-0,26375	0,17956	-0,16881	-0,63445	0,75615	-0,67774	-0,23128	-0,00129	1,655241	1,419084	0,759084	0,024751	0,136958
BRCA1_Pathway_Mismatch_Repair	0,405282	0,042211	-0,0512	1,666261	1,180182	0,152898	1,521654	2,149143	0,62523	-1,00837	-0,36847	-1,20794	-0,48211	-0,27741	-0,41412	-4,51818	-3,0142	-2,9588	0,02568	0,140092
methylthiopropionate_biosynthesis	0,025579	-0,05885	-0,12156	0,042941	-0,3984	-0,09995	-0,00036	0,043338	-0,13088	-0,13478	-0,24598	-0,17474	0,280596	0,321454	0,20777	-0,85458	-0,85107	-0,8892	0,025744	0,140198
NAD_phosphorylation_and_dephosphorylation	0,024525	0,011893	0,043458	-0,11271	0,090737	0,044919	0,213049	0,21039	0,094082	-0,51565	-0,55955	-0,47893	0,073463	0,111189	-0,05317	-0,362	-0,0426	-0,05961	0,025929	0,140956
biocarta_sprouty_regulation_of_tyrosine_kinase_	0,233167	0,207008	0,061219	0,175103	0,389988	0,511501	0,394483	0,340811	0,497701	1,114037	0,589268	0,122673	-0,06608	-0,05272	-0,3638	1,277051	1,159793	0,853307	0,026308	0,141535
BRCA1_Pathway	1,139772	-0,59178	-0,45494	2,506419	4,649848	1,275064	4,657666	6,463862	1,052229	-3,96589	0,556965	-2,17466	-1,04962	-0,08262	-0,41402	-17,4229	-12,366	-9,51938	0,026178	0,141535
ketolysis	0,118241	-0,11234	-0,08023	-0,10021	-0,38421	-0,12561	-0,7532	-0,53211	-1,01568	-1,02398	-0,21929	0,123734	0,306293	0,320077	0,490596	-1,4953	-1,23935	-1,07639	0,026185	0,141535
NCI_Nongenotropic_Androgen_signaling_Pathwa	-0,06005	-0,27993	-0,23695	0,934839	0,942695	-0,12351	-0,34503	-0,09647	-0,62596	-0,8916	0,685589	0,286319	0,122391	-0,08651	0,32369	-2,62929	-2,65312	-1,33572	0,026232	0,141535
reactome_Condensation_of_Prometaphase_Chro	0,166004	-0,01217	0,002321	0,393048	0,028846	0,095645	1,078766	1,293998	0,823542	-1,79571	-0,97084	-1,109	-0,49341	-0,3398	-0,40032	-2,55993	-2,05716	-0,91993	0,026276	0,141535
reactome_Regulated_proteolysis_of_p75NTR_Ma	0,221863	0,02652	-0,03203	0,051786	0,746862	-0,31746	-1,47562	-1,33053	-1,94993	-0,32164	0,148268	0,891708	1,05502	0,897442	1,109902	-1,83313	-0,84184	-1,23699	0,026253	0,141535
AHR_Pathway_PS2_Gene_expression_via_ESR1	0,270276	-0,00292	-0,05862	0,48024	0,266565	0,710747	-0,46859	0,009616	-0,99911	0,519611	-0,93092	-1,95031	0,142267	0,422466	-0,34752	-5,30961	-4,26948	-3,56191	0,026756	0,142509
glutamate_removal_from_folates	0,0484	0,023106	0,026637	-0,00265	0,048686	0,057682	0,608341	0,657028	0,456748	-0,21058	-0,13639	-0,17224	-0,00626	0,05556	-0,02918	-0,62181	-0,32079	-0,16592	0,026763	0,142509
NCI_Neurotrophic_factor_mediated_Trk_recepto	0,013046	-0,22403	-0,08138	0,464387	-0,18627	0,897696	-2,2962	-2,21043	-2,5479	-0,57984	-0,89301	-2,17442	-0,18257	-0,01892	-0,28137	-0,89667	-0,2512	-0,46815	0,026717	0,142509
reactome_HSF1_dependent_transactivation_Main	-0,0716	-0,34072	-0,26301	0,278126	-0,03994	0,690889	-0,63437	-0,40913	-1,1979	0,1898	0,600171	-0,80445	0,302541	0,215604	0,124346	-1,75155	-1,61871	-0,14349	0,026595	0,142509
reactome_mRNA_decay_by_3_to_5_exoribonucle	0,228878	0,056872	-0,06959	0,76884	-0,13911	-0,05344	0,153889	0,266469	-0,39445	-1,57204	-0,51041	-0,78713	-0,03767	-0,28589	0,081799	-4,32629	-3,59304	-3,6501	0,026678	0,142509
reactome_RNA_Polymerase_III_Transcription_Init	0,080742	-0,3165	-0,63471	-0,07453	-0,40496	-1,0584	-0,69766	0,131723	-2,08102	-3,06614	-1,14772	-1,53238	0,14982	0,437965	0,372622	-9,58131	-7,97823	-6,59424	0,026541	0,142509
NCI_PAR1_mediated_thrombin_signaling_events_	-0,11229	-0,38798	-0,40883	-0,09781	0,049704	0,086902	-3,10029	-2,86304	-3,16627	-0,54052	0,84957	-0,56691	0,051119	0,269307	0,908348	-2,29071	-1,3773	-0,60808	0,02682	0,142567
biocarta_tumor_suppressor_arf_inhibits_ribosom	-0,10127	-0,07435	-0,11541	0,12267	-0,35636	-0,11713	-0,09045	-0,10658	0,11695	-0,02471	0,229258	0,21649	-0,04612	-0,27064	-0,31545	0,263348	0,151773	0,12113	0,02689	0,142651
glutamate_biosynthesisdegradation	-0,0512	-0,03093	-0,1623	-0,522	-0,51196	-0,36363	-0,0639	-0,00437	-0,1548	-0,66459	-0,36867	-0,39357	-0,45522	-0,49036	-0,34479	0,756623	0,766509	1,073307	0,026979	0,142651
NCI_IL6_mediated_signaling_events_Pathway_(M	-0,00194	-0,12365	-0,1902	-0,29948	-0,37168	0,135323	-0,32206	-0,18388	-0,55004	-0,60315	-0,50224	0,110271	0,345695	0,460929	0,57262	-2,32881	-1,11777	-0,70414	0,027022	0,142651
reactome_Conversion_from_APC_C_Cdc20_to_AI	0,472535	-0,07023	-0,11325	-0,5154	-0,93144	-1,61848	0,427599	0,82897	-0,7435	-4,74316	-2,25978	-1,83804	-0,19998	0,279726	0,478121	-6,04516	-5,56614	-4,83777	0,027065	0,142651
reactome_Organic_anion_transport_Main_Pathw	0,070209	0,14573	0,200775	0,009552	-0,22105	0,114285	0,24445	-0,20771	0,712834	0,377317	0,516485	-0,68966	-0,33413	-0,14241	-0,20429	1,266629	1,04901	1,536639	0,027032	0,142651
reactome_GABA_A_rho_receptor_activation_Mai	-0,06352	0,035494	0,188184	-0,35759	0,245868	-0,52148	-0,16245	-0,39794	0,009873	0,249455	0,670615	0,674182	0,145706	-0,55689	-0,53485	0,556859	-0,17009	-0,17217	0,027117	0,142686
NCI_PDGFR_alpha_signaling_Pathway_(JAK_STAT	-0,06704	-0,09163	-0,08551	-1,71911	-1,76066	-1,49902	-2,48836	-2,40687	-2,6806	0,32782	-0,52239	0,016205	-0,10605	0,110941	-0,28764	0,6107	0,596535	1,074036	0,027224	0,143007
2-amino-3-carboxymuconate_semialdehyde_degr	-0,07489	-0,06153	0,101233	-0,35969	-0,32794	-0,19969	0,153615	0,195887	0,252429	0,215016	-0,28588	-0,3208	-0,09774	-0,30328	-0,2744	-0,2714	0,357929	-0,02935	0,028266	0,14327
biocarta_b_cell_survival_Pathway_(apoptosis)	0,039807	-0,0154	-0,02531	0,177283	0,087987	-0,14153	-0,0868	0,014861	-0,18008	-0,88178	-0,52399	-0,42945	0,092424	0,004511	-0,11081	-0,27072	-0,32531	0,009207	0,029036	0,14327
biocarta_basic_mechanisms_of_sumoylation_Mai	0,244379	0,073048	0,029855	0,36518	0,215192	0,236514	0,272436	0,349359	0,197161	-0,3298	-0,20093	-0,02221	0,011434	0,298684	0,35339	-2,51569	-1,80121	-0,9899	0,028156	0,14327
biocarta_basic_mechanisms_of_sumoylation_Path	0,244379	0,073048	0,029855	0,36518	0,215192	0,236514	0,272436	0,349359	0,197161	-0,3298	-0,20093	-0,02221	0,011434	0,298684	0,35339	-2,51569	-1,80121	-0,9899	0,028156	0,14327
biocarta_cyclins_and_cell_cycle_regulation_Main_	0,257961	-0,41038	-0,25301	2,042107	2,133069	-0,52969	1,347163	1,568048	0,81953	-1,30601	-0,60733	-0,40298	-0,01291	-0,28793	-0,82478	-6,72113	-6,14357	-4,85379	0,027844	0,14327
biocarta_role_of_U_00DF_arrestins_in_the_activa	-0,65996	-0,15053	-0,03905	-1,60576	-0,77356	-1,29232	-2,48003	-3,18821	-1,56499	3,508812	2,659115	0,482218	0,260871	0,869641	0,861028	3,089347	1,399891	2,55727	0,027522	0,14327
biocarta_sumoylation_as_a_mechanism_to_modu	0,025563	-0,17203	-0,0681	0,102427	1,074983	0,740039	0,309326	0,347008	0,027797	0,420056	0,02526	-0,96125	-0,33702	-0,0408	0,224628	-2,51724	-2,31171	-1,053	0,028289	0,14327
glycerol_degradation	0,135511	0,067297	-0,03382	-0,23074	-0,16974	0,358368	-0,10631	0,267695	-0,481	-0,49832	-0,37007	-1,86397	-0,30713	-0,13573	0,115123	-1,89236	-1,76005	-1,51087	0,028633	0,14327
KEGG_mRNA_surveillance_Main_Pathway	0,523871	-0,20485	-0,1767	-0,59422	-2,32309	-0,09809	0,411011	1,069116	-0,708	-2,76016	-2,76371	-1,21931	1,000678	0,339752	1,209037	-7,17149	-5,9744	-4,5996	0,027715	0,14327
NCI_Caspase_Cascade_in_Apoptosis_Pathway_(ad	-0,11552	-0,04459	-0,09489	-0,50655	-0,12595	-0,20926	-0,31723	-0,34771	-0,3767	0,654492	0,332409	0,287082	0,036594	0,016623	0,021447	0,094467	0,084091	0,364251	0,027856	0,14327
NCI_Cellular_roles_of_Anthrax_toxin_Pathway_(a	-0,1196	-0,06519	-0,06949	-0,31523	-0,18437	-0,25111	-0,08558	0,057503	-0,09893	-0,01605	-0,3721	-0,46444	0,360602	0,379895	0,314009	-0,98869	-0,80672	-0,74101	0,029024	0,14327
NCI_Cellular_roles_of_Anthrax_toxin_Pathway_(i	-0,1196	-0,06519	-0,06949	-0,31523	-0,18437	-0,25111	-0,08558	0,057503	-0,09893	-0,01605	-0,3721	-0,46444	0,360602	0,379895	0,314009	-0,98869	-0,80672	-0,74101	0,029024	0,14327
NCI_Cellular_roles_of_Anthrax_toxin_Pathway_(r	-0,1196	-0,06519	-0,06949	-0,31523	-0,18437	-0,25111	-0,08558	0,057503	-0,09893	-0,01605	-0,3721	-0,46444	0,360602	0,379895	0,314009	-0,98869	-0,80672	-0,74101	0,029024	0,14327
NCI_Cellular_roles_of_Anthrax_toxin_Pathway_(r	-0,1196	-0,06519	-0,06949	-0,31523	-0,18437	-0,25111	-0,08558	0,057503	-0,09893	-0,01605	-0,3721	-0,46444	0,360602	0,379895	0,314009	-0,98869	-0,80672	-0,74101	0,029024	0,14327
NCI_Cellular_roles_of_Anthrax_toxin_Pathway_(r	-0,1196	-0,06519	-0,06949	-0,31523	-0,18437	-0,25111	-0,08558	0,057503	-0,09893	-0,01605	-0,3721	-0,46444	0,360602	0,379895	0,314009	-0,98869	-0,80672	-0,74101	0,029024	0,14327
NCI_Cellular_roles_of_Anthrax_toxin_Pathway_(r	-0,1196	-0,06519	-0,06949	-0,31523	-0,18437	-0,25111	-0,08558	0,057503	-0,09893	-0,01605	-0,3721	-0,46444	0,360602	0,379895	0,314009	-0,98869	-0,80672	-0,74101	0,029024	0,14327
NCI_Cellular_roles_of_Anthrax_toxin_Pathway_(p	-0,1196	-0,06519	-0,06949	-0,31523	-0,18437	-0,25111	-0,08558	0,057503	-0,09893	-0,01605	-0,3721	-0,46444	0,360602	0,379895	0,314009	-0,98869	-0,80672	-0,74101	0,029024	0,14327
NCI_Cellular_roles_of_Anthrax_toxin_Pathway_(r	-0,1196	-0,06519	-0,06949	-0,31523	-0,18437	-0,25111	-0,08558	0,057503	-0,09893	-0,01605	-0,3721	-0,46444	0,360602	0,379895	0,314009	-0,98869	-0,80672	-0,74101	0,029024	0,14327
NCI_Insulin_mediated_glucose_transport_Main_F	0,124015	-0,10802	-0,10596	0,177438	-0,30969	-1,4071	-1,1328	-0,93829	-1,30704	-2,73386	-1,03127	-1,39864	0,770529	0,191614	0,984398	-3,04513	-2,12146	-0,71872	0,02757	0,14327
NCI_LKB1_signaling_events_Pathway_(chromatin	-0,04765	-0,05986	0,006185	-0,00072	0,161505	-0,03539	0,134204	0,155843	0,218066	0,021389	0,285733	0,362212	-0,24637	-0,09684	-0,08097	0,296296	0,497663	0,11184	0,028315	0,14327
NCI_RAC1_signaling_Pathway_(lamellipodium_as	0,037407	-0,19156	-0,11999	-0,07238	-0,87367	-0,93385	-2,19362	-2,00138	-2,79736	-2,698	-0,62284	-1,12771	0,406603	0,485699	0,931657	-5,47203	-3,57059	-2,08457	0,028455	0,14327
NCI_RhoA_signaling_Main_Pathway	0,213321	-0,05922	-0,00764	1,56901	-0,01924	-1,16906	0,895317	1,180578	-0,03525	-1,34202	0,094758	-1,03218	1,145056	1,239872	1,34488	-2,54885	-0,71956	-0,6145	0,028912	0,14327
NCI_Validated_targets_of_C_MYC_transcriptional	-0,00686	-0,11946	-0,14669	-0,32822	0,426733	0,141731	-0,53626	-0,54057	-0,73269	0,103509	-0,37715	0,234819	0,135729	0,115101	0,424389	-1,47063	-1,08936	-0,7415	0,029062	0,14327
reactome_Activation_of_BAD_and_translocation_	0,049885	-0,09861	-0,10525	0,967602	1,641534	1,197518	-0,19293	-0,03194	-0,63104	-0,80933	0,120923	-0,60011	-0,11387	0,091615	-0,1813	-1,19306	-0,62487	0,078604	0,027822	0,14327
reactome_Activation_of_NIMA_Kinases_NEK9_NE	0,079492	0,008214	-0,07296	0,190916	0,355568	0,375726	-0,52325	-0,3331	-0,75419	0,366242	-0,97457	-0,70974	-0,37244	-0,16846	0,090096	-1,0623	-1,28724	-0,83582	0,028553	0,14327
reactome_AKT_phosphorylates_targets_in_the_c	0,153988	-0,20469	-0,12245	-0,28702	1,097188	0,000918	-0,58879	-0,46341	-1,15887	-0,11462	0,822854	-1,36371	0,353038	0,400952	0,068552	-2,12236	-2,26498	-1,75541	0,028732	0,14327
reactome_Apoptotic_cleavage_of_cellular_protei	0,184085	-0,09451	-0,02733	0,094038	0,177889	-0,31809	-1,3769	-1,23499	-1,99416	0,190847	-1,05004	-0,89581	0,050328	0,330994	0,185026	-2,16954	-1,8804	-0,59777	0,027947	0,14327
reactome_Chk1_Chk2_Cds1_mediated_inactivatic	0,184059	0,130016	0,063241	0,409357	0,827132	0,239207	1,197604	1,535808	0,855901	-1,23852	-1,39029	-1,25462	-0,53779	-0,45111	-0,39675	-2,30782	-1,12818	-0,97912	0,027893	0,14327
reactome_Digestion_of_dietary_lipid_Main_Pathv	0,158324	-0,03896	0,078335	-0,58113	0,351648	0,117435	0,250687	-0,12613	0,373772	0,746227	0,170518	0,32097	0,064021	-0,12449	-0,1173	0,400385	0,837007	0,63592	0,028757	0,14327
reactome_ER_Quality_Control_Compartment_ER	0,166594	0,086705	-0,0212	0,255387	0,153523	0,41101	-0,79935	-0,4994	-1,16928	-1,08961	0,26607	-0,32501	0,018814	0,184554	0,030135	-2,89772	-2,21564	-2,33897	0,02884	0,14327
reactome_G_alpha_q_signalling_events_Main_Pa	-3,33257	0,567427	0,602428	-1,89565	-1,83486	-3,241	-4,24879	-8,02586	1,916029	5,026533	1,185185	2,786882	0,378391	-1,01108	-0,93179	21,12901	20,71068	22,20085	0,027439	0,14327
reactome_Ligand_gated_ion_channel_transport_	-0,4927	-0,05794	0,31538	-0,78694	0,69113	-1,85242	0,431733	-0,04393	1,281184	1,848338	0,790759	0,626875	0,225822	-0,7544	-0,38449	1,304356	0,788096	0,6307	0,028805	0,14327
reactome_Loss_of_Nlp_from_mitotic_centrosome	1,456798	-0,08668	-0,22122	3,078252	2,067417	1,798826	0,982407	2,408618	-2,07197	-5,16235	-0,87826	-4,73259	-1,56501	-0,5671	-0,5179	-20,8349	-14,6702	-11,3576	0,027472	0,14327
reactome_Loss_of_proteins_required_for_interpl	1,456798	-0,08668	-0,22122	3,078252	2,067417	1,798826	0,982407	2,408618	-2,07197	-5,16235	-0,87826	-4,73259	-1,56501	-0,5671	-0,5179	-20,8349	-14,6702	-11,3576	0,027472	0,14327
reactome_Processive_synthesis_on_the_C_stranc	0,196332	0,065454	0,077095	0,128898	0,270626	0,270227	1,114897	1,165364	0,980358	-1,53159	-0,43398	-0,06882	0,050786	-0,13843	-0,13572	-0,70336	-0,91971	-0,72209	0,028526	0,14327
reactome_Role_of_DCC_in_regulating_apoptosis_	-0,03449	-0,21467	-0,07693	-1,1345	-0,29254	-0,25536	-1,10121	-1,00886	-1,1363	-1,59433	-0,99509	-1,70557	0,414931	0,420986	0,228904	-1,01319	-1,54566	-1,09793	0,029068	0,14327
reactome_SUMO_is_transferred_from_E1_to_E2	0,244379	0,073048	0,029855	0,36518	0,215192	0,236514	0,272436	0,349359	0,197161	-0,3298	-0,20093	-0,02221	0,011434	0,298684	0,35339	-2,51569	-1,80121	-0,9899	0,028156	0,14327
reactome_Viral_Messenger_RNA_Synthesis_Mair	0,180059	-0,09488	-0,2229	0,559188	-0,5347	-0,18603	0,075849	0,462369	-0,42784	-2,35339	-1,40208	-0,19902	-0,29647	-0,16884	-0,015	-4,85121	-3,61279	-3,40843	0,028015	0,14327
superpathway_of_tryptophan_utilization	-0,11602	-0,07818	0,357655	-4,40403	-0,65661	-1,62553	-2,36671	-3,4333	-1,63475	-0,31944	-1,4852	-0,55165	-0,11148	-0,47908	-1,11707	4,37298	4,598592	2,332599	0,028208	0,14327
biocarta_il_4_signaling_Main_Pathway	0,026096	0,362911	0,201697	-0,84377	0,247865	0,082096	-0,5856	-0,70963	0,016363	1,566868	1,218817	1,180922	0,464668	-0,43939	-0,10801	-0,11734	0,90437	0,064154	0,029138	0,14339
reactome_Signaling_by_FGFR_mutants_Main_Pa	-0,66196	-0,41028	-0,48258	-0,90714	0,294968	-0,11651	-0,63445	-0,97756	-0,1952	-0,46856	-1,29719	-1,13923	-0,72702	-0,66365	-0,36148	-2,5313	-1,69445	-1,75291	0,02922	0,143565
reactome_Type_I_hemidesmosome_assembly_M	-0,06451	0,02324	0,120335	-0,21729	-0,25711	-0,22205	-1,48643	-1,71806	-1,18071	0,031087	-0,13653	-0,60432	-0,21158	-0,08593	-0,55675	0,405551	0,229433	0,226336	0,029333	0,143892
NCI_N_cadherin_signaling_events_Pathway_(axo	0,078162	-0,01511	-0,0553	-0,05315	-0,12242	0,005388	-0,67113	-0,56808	-0,8418	-0,07367	0,637678	-0,47584	0,659628	0,693014	0,882421	-1,23747	-0,97274	-0,6804	0,029481	0,14432
reactome_ChREBP_activates_metabolic_gene_ex	-0,15793	-0,09209	-0,12999	0,407896	0,171981	0,238291	-0,09226	-0,07608	0,053419	0,45096	0,210942	0,243579	0,268288	0,132877	0,164813	-0,01461	-0,20608	-0,18652	0,029513	0,14432
biocarta_thrombin_signaling_and_protease_activ	0,150201	-0,22292	-0,21111	-0,31172	-0,21673	-0,92806	-0,83799	-0,5091	-1,6239	-1,97522	-0,42847	-1,75136	-0,02556	0,085717	0,556101	-5,32979	-4,69905	-3,0553	0,029807	0,145529
NCI_Hypoxic_and_oxygen_homeostasis_regulatio	0,302387	0,089961	0,112613	-0,52529	0,091624	-0,66349	-0,23287	-0,10822	-1,19697	0,018402	-1,82119	-1,41014	0,48172	0,444849	0,87137	-4,37248	-3,44264	-2,53249	0,030057	0,146063
reactome_APC_C_Cdc20_mediated_degradation_	0,630902	0,04917	0,102427	-0,71652	-1,21768	-1,85444	0,612119	1,095204	-0,89169	-5,65303	-3,63028	-3,21482	-0,60482	-0,07152	0,066557	-8,21219	-7,12497	-6,30307	0,030034	0,146063
reactome_Inactivation_of_APC_C_via_direct_inhi	0,630902	0,04917	0,102427	-0,71652	-1,21768	-1,85444	0,612119	1,095204	-0,89169	-5,65303	-3,63028	-3,21482	-0,60482	-0,07152	0,066557	-8,21219	-7,12497	-6,30307	0,030034	0,146063
PTEN_Pathway	0,714029	1,473616	1,878689	-2,17144	-3,63047	-1,80686	10,29532	11,73754	11,94796	-3,9408	-3,04957	-2,43131	-2,82086	-3,64467	-4,38272	18,11257	18,684	11,71539	0,030343	0,147224
reactome_PPARA_activates_gene_expression_Ma	-0,04307	-0,2941	-0,25811	-0,51372	-0,78616	0,013763	0,438126	0,841945	-0,1843	-0,9451	-0,33033	-0,59525	0,321984	0,238246	1,389847	-3,85269	-2,6267	-1,75844	0,03039	0,147225
KEGG_Neuroactive_ligand_receptor_interaction_	-6,24498	0,414091	2,557524	-7,15271	-4,52634	-10,2622	-7,42151	-15,9298	6,78334	18,10035	5,39476	4,103077	5,82734	1,33884	3,823034	53,68357	48,36965	41,59832	0,030517	0,147612
reactome_ECM_proteoglycans_Main_Pathway	-0,25833	-0,3482	-0,44218	-0,8401	-2,7133	-2,49224	0,842753	0,480873	1,129121	0,499511	-0,54947	-0,36075	-0,14553	-0,97401	0,381308	7,567575	7,37607	6,86525	0,030605	0,147807
Estrogen_Pathway_Gene_Expression_via_FOS_JU	0,755384	-1,24322	-0,92662	0,688338	1,492209	1,076688	-6,16244	-3,66471	-9,49141	-3,34457	-2,35826	-1,3631	0,150136	-0,15482	2,349156	-25,2655	-21,3704	-14,7265	0,030697	0,148021
CD16_Pathway_in_Natural_Killer_cells	-0,02732	-0,49003	-0,45148	0,217909	-1,67541	-0,06771	-0,54216	-0,33815	-0,98502	-5,7879	-2,22193	-0,98319	-0,42887	-0,59495	-0,44905	-5,11282	-3,1544	-3,074	0,030885	0,148324
Lipoxins_Influence_on_Cell_Growth_and_Prolifer	-0,28926	-0,37767	-0,41617	-0,87823	0,229991	-1,30971	-3,70161	-3,23065	-3,47191	-1,83638	-2,03113	-0,53352	-0,33658	0,718143	1,096202	-3,48148	-1,70717	-1,48638	0,030885	0,148324
reactome_Assembly_of_the_ORC_complex_at_th	0,484064	0,102181	0,040545	0,658521	0,507353	0,571978	-0,3108	0,026224	-0,86303	-1,09815	0,397227	-1,3217	-0,19338	0,11307	-0,26316	-2,36925	-1,91386	-0,74077	0,030903	0,148324
biocarta_actions_of_nitric_oxide_in_the_heart_M	-0,05723	-0,32053	0,045663	-0,07183	0,992738	0,622832	-2,3495	-2,58611	-2,86258	1,305117	-1,15627	-0,29459	0,266509	0,395918	0,502441	-4,34676	-2,84872	-1,82474	0,031008	0,148601

KEGG_Pentose_phosphate_Main_Pathway	0,2059	-0,15925	0,408038	0,787867	0,34818	-0,60863	0,768393	1,228185	0,615971	-0,58976	-0,83943	-0,69044	0,293785	-0,38509	0,451726	-2,93287	-4,06046	-3,24109	0,031161	0,149103
Tumour_Infiltration_Pathway	-3,79738	2,452422	2,115536	-6,60479	1,514998	0,766324	-0,64304	-5,60188	9,501274	3,429684	-1,79225	7,059493	-1,44705	-3,67495	-1,70009	34,82407	29,93556	24,4753	0,031293	0,149507
biocarta_hiv_1_nef_negative_effector_of_fas_an	0,081623	-0,21731	-0,00774	0,003474	-0,28774	-1,20843	-2,01483	-2,05136	-1,95333	0,861117	0,098518	1,58186	-0,38555	0,009615	-0,53966	-0,25126	0,320007	-0,1513	0,031356	0,149575
FGF2_Signaling_in_Epithelial-mesenchymal_trans	0,217593	-0,11205	-0,05117	0,677371	0,486593	0,471851	0,214679	0,325007	-0,47137	-0,68738	0,090694	-0,40001	-0,31094	-0,2258	-0,19788	-2,07897	-1,64106	-0,99887	0,031457	0,149828
reactome_Recruitment_of_mitotic_centrosome_	1,732389	0,019912	-0,22395	3,176665	2,587565	2,730806	1,287658	2,8299	-2,22498	-3,81652	-0,00874	-3,09625	-1,62102	0,036037	0,340501	-21,4395	-16,5214	-11,7114	0,031691	0,150716
reactome_mRNA_decay_by_5_to_3_exoribonucle	0,551119	-0,20312	-0,15184	-0,04056	-0,17697	0,172651	-0,27499	0,052703	-1,03528	-1,44495	-0,23336	-0,37791	0,568919	0,282403	0,351381	-5,11298	-3,54704	-2,67045	0,031776	0,150889
KEGG_Protein_processing_in_endoplasmic_reticu	0,23788	0,237638	-0,16362	-0,66442	-0,41016	-1,10766	-3,1168	-2,73674	-3,748	-1,17869	-1,10119	-1,11775	1,44763	0,639638	0,964859	-5,20142	-4,78579	-3,3339	0,031855	0,151032
NCI_ATM_Pathway_(G2_M_transition_checkpoin	0,169068	-0,01198	-0,08087	1,087749	0,921587	1,011863	0,077773	0,209494	-0,40475	-1,02747	0,193981	-0,26062	-0,27251	-0,24343	-0,31469	-2,15389	-0,9253	-1,15096	0,031987	0,151174
NCI_Ceramide_signaling_Pathway_(ganglioside_b	0,008277	-0,32669	-0,18001	-1,25041	-0,96132	-1,4578	-0,9	-1,11099	-0,86722	-1,01592	-0,6145	1,516627	-0,77644	0,003369	-0,52975	0,082567	-0,03066	-0,08212	0,03203	0,151174
reactome_RNA_Polymerase_III_Transcription_Te	-0,03629	-0,43712	-0,7598	0,092219	-0,22887	-0,86337	0,4993	1,003931	-0,69747	-1,60721	-0,47941	-1,33665	0,128659	0,420716	0,324481	-8,9	-6,95548	-5,81431	0,031988	0,151174
Akt_Signaling_Pathway	-10,838	-2,55463	-0,28974	-21,5847	-12,7753	-22,7742	-37,6166	-45,5935	-19,2073	26,0592	-4,2611	-2,04095	5,721482	1,655796	3,320058	61,60327	55,92625	49,39033	0,032144	0,151479
mTOR_Pathway_mRNA_Biogenesis	0,005129	-0,06534	-0,03449	0,372101	0,1095	-0,31557	-0,05677	0,071966	-0,22541	-0,24472	0,193855	-0,07511	0,108368	0,119084	0,336157	-0,76841	-0,46729	-0,61486	0,032253	0,151724
NCI_Ephrin_A_reverse_signaling_Pathway_(MAPI	-0,09634	0,006563	-0,00205	-0,81255	-1,31278	-1,1534	-0,81315	-0,92684	-0,89254	0,948436	0,38704	1,021483	0,144478	0,39288	0,36205	0,493279	1,356226	0,800778	0,032342	0,151724
NCI_Ephrin_A_reverse_signaling_Pathway_(neur	-0,09634	0,006563	-0,00205	-0,81255	-1,31278	-1,1534	-0,81315	-0,92684	-0,89254	0,948436	0,38704	1,021483	0,144478	0,39288	0,36205	0,493279	1,356226	0,800778	0,032342	0,151724
AHR_Pathway_C-myc_expression_via_RELA	0,234816	-0,05011	-0,11469	0,311353	-0,0069	0,412592	-0,51006	-0,00427	-1,02	0,462475	-1,08819	-1,56449	0,17378	0,346193	-0,27174	-5,28205	-4,36774	-3,64509	0,032581	0,152389
reactome_FRS2_mediated_cascade_Main_Pathw	-0,30077	-0,31103	-0,19874	-0,77556	0,338664	-0,05631	-0,62815	-0,8189	-0,17207	-0,98851	-0,56743	-0,57204	-0,33079	0,062503	-0,15434	-1,26808	-0,74918	-0,71298	0,032563	0,152389
KEGG_Chemokine_signaling_Main_Pathway	-2,10481	0,089225	0,422491	-0,66339	4,638327	-0,16305	-2,58355	-5,53129	-1,00363	6,158995	4,290694	2,338311	-2,90519	-3,13718	0,005687	6,667982	8,317221	6,20243	0,032679	0,152397
NCI_Insulin_Pathway_Main_Pathway	0,110814	-0,60097	-0,7634	-0,91206	0,483151	-0,59553	-4,71318	-4,13203	-6,18655	0,82088	-0,92072	-0,51858	1,18825	1,278471	1,154462	-6,61213	-4,0563	-2,85294	0,032681	0,152397
reactome_Amino_acid_transport_across_the_pla	-0,32127	0,178947	0,266661	-3,94783	-3,98412	-3,02546	-4,95722	-5,50325	-4,18166	0,536414	-0,93564	-0,07617	-0,21189	0,186543	-0,40145	1,715382	0,849689	1,567207	0,032836	0,152703
reactome_Platelet_Aggregation_Plug_Formation_	-0,20271	-0,1064	0,014098	-0,12174	-0,5657	0,083607	0,231209	-0,06694	0,804991	0,779307	0,471186	-0,04667	0,140327	-0,06661	0,146921	1,038211	0,841249	1,009957	0,032845	0,152703
reactome_Removal_of_the_Flap_Intermediate_N	0,442418	0,222251	0,203436	0,499163	1,013	1,030742	3,843263	4,336324	3,410474	-1,27188	-0,86884	-0,99777	-0,52188	-0,4551	-0,58391	-3,46883	-2,8651	-1,96316	0,032925	0,152849
Glucocorticoid_Receptor_Signaling_Pathway_Cell	0,193797	-0,51228	-0,32239	0,234275	-0,07014	-0,58887	-0,00558	0,330099	-0,71241	-2,68009	-0,24204	0,715611	0,080489	0,94163	0,672083	-5,51131	-3,97244	-2,32211	0,033274	0,15424
KEGG_Renin_angiotensin_system_Main_Pathway	-0,06423	0,101888	-0,019	-1,71526	-1,60318	-0,47392	0,27578	-0,04659	0,898556	4,056633	1,298282	1,292501	-0,01794	0,12821	0,645815	1,32319	0,42364	-0,22306	0,033476	0,154943
androgen_biosynthesis	-0,29956	0,006427	0,110968	0,315176	-0,34317	-0,32932	-0,09375	-0,19585	0,682245	1,403678	0,683938	-0,03608	-0,16006	-0,33601	0,496011	1,560599	1,525494	0,633415	0,034112	0,156246
biocarta_double_stranded_rna_induced_gene_ex	0,125285	0,188274	0,080188	0,066974	0,243892	0,124489	0,369648	0,362823	0,244549	-0,5919	-0,39349	-0,24674	-0,19753	0,026481	0,073125	-2,01364	-1,52803	-1,16749	0,034387	0,156246
GSK3_Signaling_Pathway_Protein_Synthesis	0,151765	0,30577	0,048102	-1,08986	0,006083	-0,688	-0,41622	-0,73037	-0,2861	1,574306	-0,43631	3,211932	-0,5459	0,126434	-0,11395	0,040712	-0,0713	0,872253	0,034307	0,156246
KEGG_Phagosome_Main_Pathway	-0,0843	-0,55879	-0,07368	0,410036	-0,24958	-0,52773	-2,78941	-3,17064	-2,81869	-2,3308	-1,6616	-0,10237	-0,3881	-0,74716	-0,25457	-4,89496	-3,2743	-2,93273	0,034544	0,156246
NCI_ErbB2_ErbB3_signaling_events_Pathway_(ce	-0,01469	0,007509	0,02971	-0,34508	-0,46923	-0,57387	0,287945	0,362184	0,275444	0,530251	0,230494	0,67319	-0,06869	0,007888	0,096837	-0,15064	-0,06263	-0,06694	0,034243	0,156246
NCI_p75_NTR_mediated_signaling_Pathway_(neu	0,082638	-0,03368	-0,1897	-0,56993	0,321952	0,215589	-2,21502	-1,57922	-2,81529	-0,29266	1,051187	-0,3295	1,345879	0,876188	1,37041	-5,38019	-3,13679	-4,37044	0,034506	0,156246
NCI_Syndecan_3_mediated_signaling_events_Ma	0,016528	-0,05529	-0,05834	0,028779	0,522692	-0,11752	1,112395	1,094584	0,826261	-0,90003	0,285229	-0,70152	0,047658	0,119409	0,06789	-1,48311	-0,11869	-0,43625	0,03427	0,156246
NCI_Trk_receptor_signaling_mediated_by_the_M	0,119832	-0,12495	-0,16996	-0,45797	-0,39609	0,076519	-0,51826	-0,16077	-0,92962	-1,34692	0,3968	-1,30712	0,136983	0,468747	0,407107	-1,53838	-0,99688	-0,7852	0,034072	0,156246
reactome_Abacavir_metabolism_Main_Pathway	-0,0756	-0,10785	-0,0978	0,01448	0,299473	0,474182	0,404286	0,46648	0,272782	0,542412	-0,77165	0,657443	0,154528	0,479248	0,123712	-1,01287	-1,13418	-0,80936	0,034216	0,156246
reactome_Insulin_processing_Main_Pathway	-0,05391	-0,16855	-0,00134	0,143417	0,066716	-0,15453	-1,22341	-0,76619	-1,7625	-1,54946	-1,87662	-0,69301	0,377202	0,240869	0,634408	-0,18876	0,01403	0,384085	0,03411	0,156246
reactome_Intrinsic_Pathway_Main_Pathway	-0,22644	0,082598	0,05936	-0,35892	-0,67136	1,818899	-1,11041	-1,7262	-0,20325	1,910415	0,899014	0,86796	-0,21466	-0,55764	-0,79812	2,452979	2,132878	2,020024	0,034378	0,156246
reactome_Phosphorylation_of_the_APC_C_Main_	0,588782	0,069349	0,017539	-0,24973	-1,24082	-1,42592	0,963309	1,454352	-0,41204	-4,81208	-2,63924	-2,04113	-0,41734	-0,00284	0,196656	-7,60299	-6,61486	-5,53958	0,034044	0,156246
reactome_PI3K_Cascade_Main_Pathway	-0,07094	-0,10562	-0,16734	0,074232	0,053426	-0,23713	-0,73485	-0,73898	-1,01241	0,484091	-0,60123	-0,48491	0,012822	0,23639	0,215653	-1,34025	-1,03839	-0,40601	0,03456	0,156246
reactome_Signaling_by_Activin_Main_Pathway	0,217951	0,300938	0,214941	0,610134	-0,06296	0,085827	-0,15966	-0,0534	-0,35575	-1,70893	-0,49261	-1,46435	0,309293	0,058968	0,091753	-0,40292	-1,05518	0,034987	0,034327	0,156246
reactome_Stabilization_of_p53_Main_Pathway	0,063055	-0,06248	-0,074	0,557319	0,383203	0,470496	-0,07637	0,014751	-0,28519	0,271311	0,301441	0,100841	0,043515	-0,00701	0,042052	-0,88659	-0,61221	-0,58279	0,034421	0,156246
reactome_Zinc_efflux_and_compartmentalizatior	0,048047	0,026895	-0,1232	0,593366	0,383407	0,697257	-0,03146	0,074403	-0,01586	-0,06064	0,131375	0,122874	-0,22231	0,109654	0,138612	-1,14383	-0,64969	-0,07302	0,034129	0,156246
NCI_S1P5_Pathway_(telencephalon_oligodendroc	0,068926	-0,08287	-0,0827	0,628633	0,076672	0,386159	-0,35961	-0,30414	-0,32392	0,094444	0,289167	-0,33544	0,085162	-0,14404	0,132966	-1,73791	-2,07097	-1,37846	0,034613	0,15626
reactome_RNA_Polymerase_III_Abortive_And_Re	-0,17618	-0,95556	-1,24672	0,340681	-0,04373	-0,5192	-0,55351	0,289962	-2,34968	-3,12201	-0,44144	-0,75434	-0,42934	0,493633	0,022037	-13,9459	-10,8159	-9,48349	0,034694	0,156399
KEGG_Cyanoamino_acid_metabolism_Main_Path	0,09777	0,079662	0,084878	-0,84545	-0,40031	-0,78035	0,588138	0,347157	0,837864	-0,33365	0,346171	0,501343	0,310537	-0,07834	0,152835	1,068605	0,533221	0,41144	0,034923	0,15675
NCI_IL23_mediated_signaling_events_Pathway_(	0,081455	0,061066	-0,05082	-0,74599	-0,24183	-0,46566	0,13788	-0,05957	0,133325	-0,30456	-0,09693	0,408792	0,031323	0,017093	0,070131	0,716305	0,487772	0,484177	0,034913	0,15675
thiamin_salvage_III	0,001395	0,007149	-0,00941	-0,00894	-0,0582	-0,1401	0,181089	0,2689	0,103011	0,842285	0,18379	-0,25633	-0,15574	-0,12021	-0,28211	0,192733	0,386722	-0,02831	0,034834	0,15675
KEGG_Vascular_smooth_muscle_contraction_Ma	-0,21093	-0,01294	-0,18112	0,411231	-0,40401	0,262048	-1,30665	-1,38203	-1,18864	-0,43412	-0,41086	-0,23673	0,312475	0,202752	0,359269	-0,05509	0,1845	-0,10231	0,035143	0,157285
reactome_Methionine_salvage_Main_Pathway	0,069014	-0,10697	-0,07538	0,203603	-0,75709	-0,10158	-0,62791	-0,32312	-0,70183	0,444372	-0,45599	-0,9947	0,553483	0,545379	0,419642	-2,55654	-1,7459	-1,82192	0,035138	0,157285
reactome_Mitochondrial_tRNA_aminoacylation_I	0,384795	-0,15765	-0,04997	-0,14335	1,327852	-0,5869	-0,09537	0,275323	-1,19487	0,304909	-0,80868	1,105936	0,959524	1,68319	1,165137	-5,66352	-5,60737	-4,31756	0,035225	0,157427
mTOR_Pathway_Autophagy	-0,00697	-0,06324	-0,00226	0,188586	0,456824	0,226857	0,881151	0,923822	0,957984	-0,45622	0,012898	0,14743	-0,18093	0,061448	0,049636	-0,51247	-0,05909	-0,37076	0,03536	0,157577
reactome_ARMS_mediated_activation_Main_Pat	0,014243	-0,03726	0,009082	-0,19567	-0,03111	0,058782	-0,15292	-0,35786	-0,18442	-0,87926	-0,71091	0,355121	0,28819	0,331742	0,237295	-0,88344	-0,3018	-0,14854	0,03534	0,157577
reactome_Ubiquinol_biosynthesis_Main_Pathway	0,297122	0,22274	0,160584	0,423981	-0,14972	0,246092	0,954485	0,974807	0,382411	-1,22535	0,272499	-0,7427	0,089014	0,212991	0,075268	-2,70473	-2,21863	-2,97818	0,035497	0,157737
ubiquinol-10_biosynthesis	0,297122	0,22274	0,160584	0,423981	-0,14972	0,246092	0,954485	0,974807	0,382411	-1,22535	0,272499	-0,7427	0,089014	0,212991	0,075268	-2,70473	-2,21863	-2,97818	0,035497	0,157737
KEGG_T_cell_receptor_signaling_Main_Pathway	0,069855	-0,70354	-1,2041	-0,2165	3,33855	-0,29661	-4,18416	-3,08506	-4,93618	0,94912	-0,92883	0,47164	0,604378	0,722702	2,776999	-3,97887	-2,65387	0,565242	0,035648	0,158182
biocarta_repression_of_pain_sensation_by_the_t	-0,17531	-0,42296	-0,44766	-1,07327	-0,28161	-0,13752	-0,55976	-0,41612	-0,96788	-2,16038	-0,94636	0,119662	-0,41023	-0,00449	0,373399	-3,08573	-2,18397	-1,96711	0,036626	0,162058
biocarta_signal_transduction_through_il1r_Pathw	0,042747	-0,07131	-0,05622	0,341188	-0,20274	0,161854	-0,79632	-0,3869	-0,95779	-0,39938	-0,90867	-1,35083	-0,17972	-0,10658	-0,00351	-0,50645	-0,39371	-0,07771	0,036602	0,162058
reactome_Regulation_of_PLK1_Activity_at_G2_M	1,673678	-0,16682	-0,19215	2,004414	1,974896	1,673033	1,939556	3,342901	-1,49029	-5,97784	-0,6628	-5,26508	-2,05259	-0,71001	-0,64694	-25,0459	-17,9878	-13,8658	0,036957	0,16306
reactome_RNA_Polymerase_II_Pre_transcription_	1,044398	-0,42377	-0,31473	2,374259	1,420562	-0,15	-0,63501	0,768363	-3,16167	-3,44332	-0,80161	0,180123	-0,17884	0,026687	1,14908	-17,2495	-13,9887	-10,414	0,036908	0,16306
biocarta_nfkb_activation_by_nontypeable_hemo	0,13443	-0,08326	0,045438	0,068328	-0,24721	0,128992	-0,51377	-0,2855	-0,68141	-0,4942	-0,84493	-1,31694	-0,01846	-0,11797	0,026233	-0,398	-0,48943	-0,28067	0,037234	0,16317
biocarta_tpo_signaling_Main_Pathway	-0,32574	-0,61083	-0,55532	-0,95517	-1,72112	-2,23968	-0,78208	-0,56608	-1,06672	0,465557	-0,31121	0,461345	-0,7559	-1,26126	-0,64779	-1,97044	-0,77365	-0,41565	0,037045	0,16317
KEGG_ECM_receptor_interaction_Main_Pathway	-1,31184	-1,02691	0,019173	-3,22204	-3,65915	-4,50224	-6,37893	-7,63378	-4,84704	2,272668	-1,00716	2,093297	0,70483	0,83334	1,152009	8,242008	6,014598	4,329312	0,03716	0,16317
PAK_Pathway_Transciptional_Activation_and_Filc	0,158871	0,236099	0,091705	-0,19069	-0,08607	0,264497	0,19861	0,414019	-0,13792	-0,94387	0,28736	-0,08812	0,498573	0,373841	0,550295	-1,23557	-1,09344	-0,30304	0,037244	0,16317
reactome_Synthesis_secretion_and_inactivation_	-0,17572	0,092312	-0,03948	-0,25473	0,810129	0,902147	-1,68064	-1,83027	-1,67925	-0,2531	0,176417	0,569136	1,299973	1,251147	1,439537	0,027021	0,379234	0,960589	0,03713	0,16317
reactome_Rhesus_glycoproteins_mediate_ammo	0,029834	-0,11795	0,057254	-0,73057	-0,81876	0,115764	-0,00844	-0,29473	0,419713	-0,08101	-0,61383	-0,25183	-0,33184	-0,30139	-0,15057	1,099013	0,582088	0,966129	0,037397	0,163612
Estrogen_Pathway_Anti-Apoptosis	-0,3652	-0,86308	-0,84971	0,316376	1,629334	1,003752	-1,65662	-0,98508	-2,37028	-0,87502	-0,10779	0,435565	-0,83712	-0,88154	0,179422	-7,80894	-6,83338	-4,82366	0,037455	0,163635
reactome_Beta_oxidation_of_very_long_chain_fa	0,140927	-0,011	0,181741	-0,83852	-0,40429	-0,26372	-0,16764	-0,16532	-0,49814	0,364611	0,232987	0,684689	-0,32163	-0,18174	-0,23959	-0,79816	-0,33074	-0,20717	0,037624	0,164143
KEGG_Regulation_of_lipolysis_in_adipocytes_Ma	-0,63256	0,203847	-0,19971	-1,73595	-0,40947	-0,41538	-1,34104	-1,00495	-0,37624	0,853314	1,534626	1,073028	0,362568	0,93403	0,576456	6,112109	5,848615	5,337614	0,037769	0,164546
oxidized_GTP_and_dGTP_detoxification	0,019003	0,033197	0,037801	-0,0539	0,323973	0,096418	0,159915	0,22049	0,248067	-0,26797	-0,15471	-0,12641	0,050769	0,037843	-0,0084	-0,21235	-0,02878	-0,05587	0,037828	0,164573
NCI_HIF_2_alpha_transcription_factor_network_I	-0,06848	-0,05089	-0,04585	-0,36201	0,006179	-0,32044	-0,08276	-0,06576	-0,18452	-0,03621	-0,35903	-0,42224	0,325295	0,086039	0,24291	-1,65751	-1,53794	-1,22687	0,037883	0,164582
NCI_IL2_mediated_signaling_events_Pathway_(p	-0,11628	0,428753	0,242803	-0,98177	0,181595	-1,18196	-0,22031	-0,66567	-0,10403	0,692238	0,530854	0,740076	-0,09888	-0,33004	-0,13802	0,017794	-0,41743	-0,24564	0,038128	0,165414
KEGG_Biotin_metabolism_Main_Pathway	0,045474	-0,03441	-0,04582	0,165189	0,028338	0,36923	-0,07218	-0,0544	-0,14034	-0,06583	0,323979	-0,17695	0,065486	0,021165	0,036288	-0,55014	-0,24369	-0,73616	0,038224	0,165432
KEGG_Jak_STAT_signaling_Main_Pathway	0,037211	1,040519	1,298361	-4,63038	-1,14808	-6,74112	-0,38829	-2,03882	4,179895	8,0629	-0,51531	4,739345	2,336417	1,647459	3,847309	9,455789	9,967049	5,42581	0,038238	0,165432
IGF1R_Signaling_Pathway_Gene_Expression_Prol	0,273957	-0,01824	-0,19967	1,725737	0,889933	1,440444	-0,65898	-0,39192	-0,95551	-1,91725	0,272694	-1,75395	-0,12905	-0,4176	0,694483	-1,66922	-0,10289	1,091409	0,038411	0,16595
KEGG_Glycosylphosphatidylinositol_GPI_anchor_	0,959224	0,197315	0,31175	0,662104	-0,0063	-0,34708	2,121618	3,167703	0,437373	-3,49705	-1,71388	-1,35923	-0,75026	-0,19421	-0,16644	-6,45945	-4,59554	-4,12814	0,038649	0,166056
KEGG_Long_term_depression_Main_Pathway	0,181997	0,116063	0,183125	-0,36467	-1,08643	-0,68931	-2,55583	-2,58948	-3,01727	-1,50623	-0,75696	0,138484	-1,03366	-0,66761	0,053807	1,589491	0,702056	1,833594	0,038516	0,166056
reactome_Apoptotic_cleavage_of_cell_adhesion_	-0,14093	-0,0883	0,177182	-0,72738	-1,06287	-0,72999	-0,30957	-0,48176	-0,4379	0,504455	-1,60979	1,516274	-0,32593	-0,09756	0,122625	1,209982	0,807394	0,907192	0,038594	0,166056
reactome_Removal_of_DNA_patch_containing_al	0,231156	0,103581	0,12377	-0,24202	0,498061	0,034678	1,523278	1,611988	1,249594	-2,54406	-0,39253	-0,15405	0,058305	0,007593	-0,0502	-1,61138	-1,51158	-1,14165	0,038635	0,166056
biocarta_t_cell_receptor_signaling_Main_Pathwa	-0,40891	-0,30704	-0,41587	-0,55775	-1,70784	-0,73662	0,024868	0,036344	0,308167	-0,62238	0,260659	-0,60846	-0,64683	-1,49462	0,141684	0,26322	0,441571	1,992518	0,038865	0,166755
reactome_Separation_of_Sister_Chromatids_Mai	4,690544	0,961161	0,76881	2,405797	1,212008	-1,91439	13,66915	16,69913	4,526756	-20,7888	-5,09994	-9,62556	-2,31607	-0,06554	0,41	-56,7406	-43,0549	-34,0849	0,039046	0,167302
superpathway_of_pyrimidine_deoxyribonucleosic	0,011186	0,100816	-0,06335	0,23497	0,599592	0,430193	0,852064	1,047819	0,371183	0,458663	1,496708	-0,18107	0,925616	0,821073	0,90486	-3,6315	-3,44953	-2,76907	0,039247	0,167931
glutathione_redox_reactions_I	-0,07418	-0,15301	-0,17505	0,767955	0,024241	0,576814	-0,07809	-0,0801	0,025152	-0,83145	-0,28008	1,043636	-0,02228	-0,04362	0,096241	-1,3327	-0,73214	-0,97463	0,039345	0,168121
chondroitin_sulfate_degradation_metazoa	0,07344	-0,00892	-0,00538	-1,30446	-0,37277	-1,03075	-0,53588	-0,52415	-0,29043	2,067621	0,585094	-0,28874	-0,5502	-0,67933	-0,38837	-0,61101	-0,3582	-1,02203	0,039542	0,168565
NCI_Arf6_trafficking_events_Pathway_(substrate_	0,181357	0,015945	-0,11081	-0,56764	-1,27116	0,040315	-0,22974	-0,04175	-0,62392	-0,99993	-1,56568	-1,39972	0,570168	0,235897	0,00256	-3,09286	-1,76143	-1,98207	0,039619	0,168565
NCI_RhoA_signaling_Pathway_(actin_cytoskeleto	0,014646	-0,01094	0,009873	0,207332	0,059482	0,141559	-0,07544	0,01417	-0,16813	-0,07807	0,141003	-0,16861	0,008692	-0,05859	0,036743	-0,90268	-0,95085	-0,58853	0,039678	0,168565
NCI_Visual_signal_transduction_Cones_Main_Pat	-0,10122	0,045441	0,125061	0,500844	-0,20634	-0,21492	-0,36756	-0,16037	0,172236	1,281935	0,70068	1,615512	0,099763	-0,21117	0,046267	0,274931	0,920302	0,040651	0,03972	0,168565
S-adenosyl-L-methionine_biosynthesis	0,015097	0,025992	-0,0173	-0,06791	-0,02282	-0,0116	-0,24329	-0,04168	-0,19635	0,015984	-0,353	-0,43215	0,028223	-0,10484	-0,03342	-0,93892	-0,9468	-0,82309	0,039587	0,168565
retinoate_biosynthesis_II	-0,08219	-0,07942	-0,08696	0,02645	-0,11688	0,352913	-0,19383	-0,27253	-0,27552	-0,48854	-0,17544	0,068973	0,130657	0,138294	0,212023	0,150264	0,157506	-0,37495	0,039862	0,168938
biocarta_b_cell_survival_Pathway_(cell_prolifera	0,008567	-0,03961	-0,01157	-0,03572	0,729537	0,124355	-0,10878	-0,12358	-0,31348	0,109153	0,289917	-0,02128	0,142838	0,135705	0,193266	-0,94325	-0,42698	-0,38877	0,040375	0,169483
ILK_Signaling_Pathway_Epithelial_Mesenchymal_	0,783435	-0,01616	-0,05872	-0,75721	0,307349	0,722769	0,093719	0,363139	-0,61454	-1,36586	0,524862	-0,80658	1,656924	0,487821	0,033445	-5,85028	-5,48318	-3,16039	0,040272	0,169483
NCI_N_cadherin_signaling_events_Pathway_(regu	-0,0122	-0,0583	-0,01166	0,522551	0,970841	0,241629	-1,01704	-1,03636	-1,30056	-0,14557	0,23225	-0,21508	-0,17539	-0,14768	-0,11297	-1,99375	-1,39851	-0,92328	0,040234	0,169483
reactome_Frs2_mediated_activation_Main_Pathv	-0,06401	-0,13936	-0,13664	0,128566	0,27257	0,135125	-0,57315	-0,65524	-0,71077	-0,57974	0,176785	0,662944	0,220256	0,16257	0,250644	-1,95991	-1,38541	-0,77189	0,040137	0,169483
reactome_Leading_Strand_Synthesis_Main_Pathv	0,373475	0,250302	0,215449	0,334554	0,610565	0,551297	2,23435	2,408078	1,673767	-1,64739	-1,20631	-0,99605	-0,44206	-0,55668	-0,65378	-2,4787	-2,0647	-1,38684	0,040589	0,169483
reactome_NCAM_signaling_for_neurite_out_grov	0,130138	-0,10893	-0,20451	-1,064	0,105175	-0,19196	0,533264	0,832045	0,409072	-2,89636	-1,05592	-2,01481	0,113525	0,333045	0,040581	-1,45112	-0,99619	-0,39534	0,040509	0,169483
reactome_NoRC_negatively_regulates_rRNA_exp	1,105271	-0,33153	-0,86324	1,208168	1,439486	-1,65161	2,926903	4,636377	0,131488	-2,35281	-1,04541	-1,25097	0,553173	-0,22674	2,240203	-19,7866	-14,9458	-12,7795	0,040066	0,169483
reactome_Processive_synthesis_on_the_lagging_	0,373475	0,250302	0,215449	0,334554	0,610565	0,551297	2,23435	2,408078	1,673767	-1,64739	-1,20631	-0,99605	-0,44206	-0,55668	-0,65378	-2,4787	-2,0647	-1,38684	0,040589	0,169483
reactome_Progressive_trimming_of_alpha_1_2_l	0,04367	-0,0098	-0,04945	0,286372	0,228541	0,076185	-0,88749	-0,85152	-0,79176	-0,09721	-0,07336	0,113191	0,083654	0,061845	-0,15121	-1,34636	-1,38624	-1,40401	0,040531	0,169483
reactome_Translocation_of_GLUT4_to_the_plasn	0,23843	-0,19097	-0,53532	-2,77056	-1,6693	-2,29283	-1,66657	-1,2538	-2,70558	-2,0026	-2,91388	-5,20179	0,634354	1,349528	1,64766	-12,7678	-10,0324	-7,68541	0,040241	0,169483
UDP-D-xylose_and_UDP-D-glucuronate_biosynthe	0,080718	-0,02705	-0,02511	-0,06104	-0,19477	-0,23387	-0,39075	-0,29388	-0,56628	-0,19385	-0,3279	-0,23017	0,090125	0,116917	0,235471	-0,81012	-0,58254	-0,48538	0,040583	0,169483
Inside-out_Signaling_through_Integrins	-0,79206	-0,97305	-1,0318	0,657862	0,999633	0,037386	-0,33265	-0,71949	-0,77573	-1,99734	3,75815	-0,06908	0,561903	0,512073	1,449165	-3,96788	-3,82362	-1,59507	0,040759	0,169513
reactome_Mitochondrial_transcription_initiation_	0,034751	-0,01584	-0,04643	0,000543	-0,09194	-0,00315	0,056627	0,201697	-0,05608	0,169127	-0,22122	0,282502	0,284716	0,254417	0,394414	-0,7337	-0,80702	-0,70144	0,040749	0,169513
reactome_Sperm_Oocyte_Membrane_Binding_N	-0,04273	0,018015	0,039376	-0,03569	-0,2837	-0,30159	0,050907	-0,11898	0,154675	1,101509	0,137467	0,189589	-0,01931	-0,05878	0,162723	0,355481	-0,03972	0,297125	0,040667	0,169513
reactome_Activation_of_the_pre_replicative_con	0,894756	0,149506	0,508271	3,489377	2,02541	2,226539	7,263875	8,27552	5,336996	-1,5385	-1,00251	-1,9631	-1,73227	-0,65977	-1,02718	-8,72213	-6,33967	-4,13683	0,040878	0,169778
5-aminoimidazole_ribonucleotide_biosynthesis	0,088159	0,002834	-0,01113	0,309832	0,516685	0,492178	0,053917	0,134193	-0,05745	0,474361	-0,22556	-0,00687	0,055635	-0,10677	0,061817	-0,48852	-0,51634	-0,38087	0,041008	0,169865
RALB_Signaling	0,201297	0,037733	-0,03918	-0,03062	0,404657	0,016135	0,236796	0,345925	0,080961	-0,89828	-0,08621	-0,55612	-0,1343	-0,11674	0,123049	-0,63896	-0,27898	-0,29673	0,040986	0,169865
KEGG_RNA_degradation_Main_Pathway	1,269317	-0,2665	-0,61389	0,170468	-0,63388	-0,50867	-0,79711	0,723225	-4,00182	-7,25909	-1,03072	-2,32312	0,06337	0,442483	1,376145	-25,9205	-19,6151	-15,6509	0,041259	0,170461
reactome_Signaling_by_Hippo_Main_Pathway	0,127086	-0,37724	-0,01235	0,640616	0,369943	0,016112	0,100097	0,252467	-0,78117	-1,67195	0,34001	1,443494	0,223918	0,599293	0,577541	-3,27032	-3,59717	-1,90656	0,041261	0,170461
phenylethylamine_degradation_I	-0,08197	0,08527	-0,01505	-0,04197	-0,0415	-0,22723	0,811745	0,616276	1,01638	-0,24385	0,817463	0,322854	-0,09304	0,072001	0,203012	0,616975	0,637772	0,54908	0,041423	0,170827
reactome_GRB2_events_in_ERBB2_signaling_Ma	0,024993	-0,07416	-0,09227	-0,36386	-0,02879	-0,13058	0,01874	0,06749	-0,16038	-0,8899	0,033104	-0,6137	-0,15686	0,063178	-0,05258	-1,29408	-0,95288	-0,99534	0,041459	0,170827
NCI_Signaling_events_mediated_by_Stem_cell_fa	-0,02199	-0,14097	-0,11789	-0,17415	-0,54789	-0,86516	-0,52945	-0,37947	-0,62346	-0,31394	0,334403	0,644247	-0,21766	-0,3171	-0,14325	-0,29449	-0,32472	0,078437	0,041686	0,171433
tryptophan_degradation_X_mammalian_via_tryp	0,080779	0,056237	-0,00498	-0,46035	-0,42864	-0,20481	-0,74109	-0,72169	-0,61394	-0,68884	0,314554	-0,26129	0,211217	-0,34859	-0,23728	1,143258	1,084555	1,331307	0,041716	0,171433
biocarta_ifn_gamma_signaling_Main_Pathway	0,259547	0,105803	0,036014	-0,10149	0,056996	0,160673	0,4394	0,498059	0,122136	-0,19118	-0,40656	-0,49109	-0,00429	0,084553	0,260976	-1,70727	-1,07015	-0,40101	0,041886	0,171905
KEGG_Amino_sugar_and_nucleotide_sugar_meta	0,95688	0,452705	0,427922	-2,12591	0,047828	1,201974	-3,37701	-2,85028	-4,04968	-2,50376	-2,25403	0,077824	0,622389	0,954232	1,453934	-9,65881	-7,95616	-6,89289	0,042049	0,172154
NCI_AlphaE_beta7_integrin_cell_surface_interact	0,053725	-0,06631	0,191564	-0,59614	-0,10489	0,001277	-0,02494	-0,11951	0,20877	0,56653	-0,38114	0,919049	-0,12097	0,259338	0,249624	0,767273	1,152262	0,619606	0,042057	0,172154
NCI_ALK2_signaling_events_Main_Pathway	-0,08614	-0,12968	-0,20178	0,20142	1,127071	-0,07079	-0,17074	-0,13689	-0,63944	-0,25163	0,19753	0,09732	0,176491	0,068362	-0,23104	-1,85267	-1,57391	-0,62676	0,042157	0,172333
reactome_Muscarinic_acetylcholine_receptors_N	-0,03497	2,23E-05	0,0343	-0,64339	0,151015	-0,74551	-0,37328	-0,42672	-0,21015	1,24116	-0,11874	0,44412	0,468437	0,276134	0,157661	1,633841	1,441981	1,405207	0,042323	0,172785
biocarta_cdc25_and_chk1_regulatory_pathway_i	0,236752	-0,04614	0,009269	0,765273	0,729797	0,459191	0,994439	1,105066	0,769667	-0,41156	-0,15298	-0,42884	-0,22448	-0,26106	-0,20589	-1,16295	-0,85885	-0,51955	0,042525	0,173244
NCI_Aurora_A_signaling_Main_Pathway	0,189387	0,139583	-0,07108	0,040057	-1,23398	0,784521	1,928949	2,364378	1,325616	-4,10631	-3,19171	-2,74939	-0,7179	-0,06119	-0,8229	-3,84103	-1,35751	-1,24862	0,042546	0,173244
NCI_PDGFR_beta_signaling_Main_Pathway	-0,60534	-0,88765	-1,10562	-0,32573	-0,34467	-1,8919	-4,05754	-3,34315	-5,9704	-2,95892	-1,23407	-2,84058	-0,74583	0,200989	0,653455	-8,64783	-4,33345	-0,61162	0,04285	0,174253
reactome_Integrin_cell_surface_interactions_Mai	-0,80803	-0,46791	-0,04339	-2,16631	-2,60227	-4,21045	-3,53468	-4,71051	-2,50341	2,641663	0,083826	-0,45535	1,176671	0,014617	1,278583	6,418107	4,883282	4,40499	0,043	0,174634
NCI_BCR_signaling_Main_Pathway	-0,01083	-0,23477	-0,42625	-0,204	0,994227	-0,57909	0,31484	0,760621	-0,22047	-1,95861	0,152945	-0,89593	0,569603	-1,16949	-0,06359	-4,47926	-3,58038	-2,2145	0,043074	0,174707
lipoate_biosynthesis_and_incorporation	0,100867	0,052454	-0,04364	-0,12794	-0,24009	-0,08676	-0,51984	-0,41333	-0,68677	-0,68164	-0,40306	-0,19874	-0,06539	0,030691	-0,02013	-0,89361	-0,80557	-0,93976	0,043176	0,174893
biocarta_proteasome_complex_Main_Pathway	0,994385	0,557487	0,490371	0,729651	0,90031	-0,0708	0,885106	1,199031	-0,13965	-3,38489	-0,04676	-1,21641	0,014057	0,306745	0,400287	-11,3428	-9,84552	-8,04896	0,043508	0,175325
biocarta_proteasome_complex_Pathway_(Pathwa	0,994385	0,557487	0,490371	0,729651	0,90031	-0,0708	0,885106	1,199031	-0,13965	-3,38489	-0,04676	-1,21641	0,014057	0,306745	0,400287	-11,3428	-9,84552	-8,04896	0,043508	0,175325
BRCA1_Pathway_Homologous_Recombination_R	0,347826	0,078454	-0,05413	0,54466	1,077584	0,868157	1,363968	2,067186	0,338854	0,047111	-0,02239	0,299568	-0,25627	0,368951	0,311085	-4,94262	-2,52858	-2,4194	0,043434	0,175325
NCI_BARD1_signaling_events_Pathway_(DNA_rep	0,53369	0,183534	0,066534	0,828452	1,240774	1,154391	1,218996	1,6209	0,126984	-0,37365	-0,03843	-1,03062	-0,31535	-0,37614	-0,19196	-2,66285	-1,61421	-0,8447	0,043354	0,175325
NCI_Plasma_membrane_estrogen_receptor_signa	-0,03282	-0,04566	-0,11736	-0,16982	0,802994	0,197193	0,146599	0,18996	0,145932	-0,23615	-0,50032	-0,46248	-0,2092	0,018341	-0,1072	-0,82418	-0,44858	-0,34175	0,043741	0,175811
reactome_Toll_Like_Receptor_9_TLR9_Cascade_I	0,012429	-0,06361	-0,02847	0,120919	0,607733	0,709708	-0,59536	-0,47053	-0,90834	0,116766	0,530514	0,472019	0,151168	0,317585	0,043617	-1,35862	-1,59865	-1,28303	0,043703	0,175811
FGFR_Signaling	0,160438	-0,82687	-0,62511	-0,1707	0,614473	0,208287	-1,58402	-0,56215	-3,20932	-2,11264	1,609285	1,429032	1,061052	0,807812	1,622426	-7,91269	-4,17163	-3,31822	0,043906	0,176247
NCI_PDGFR_beta_signaling_Pathway_(actin_cyto	-0,06814	-0,06056	-0,06342	-0,29895	0,185086	-0,07522	-0,47361	-0,43129	-0,60988	-0,16557	-0,07426	-0,02776	0,10762	0,027729	0,114772	-1,27062	-1,03201	-1,02472	0,043999	0,176394
Integrins_Signaling_in_Natural_Killer_Cells	0,190848	-0,11905	-0,2917	-1,64246	-2,78541	-2,76779	-3,68577	-3,86039	-3,26199	1,490709	1,019068	0,771136	0,332005	-0,27747	0,136713	-0,26354	-0,1742	0,801335	0,044425	0,177873
KEGG_alpha_Linolenic_acid_metabolism_Main_P	-0,25407	-0,07173	-0,05644	-0,25361	-2,4245	-0,68303	-0,04262	-0,40375	1,307573	-0,63495	-1,41656	-1,21172	-1,20122	-0,66348	-1,18513	0,709002	2,383794	0,853803	0,044546	0,178125
biocarta__U_00DF_arrestins_in_gpcr_desensitiza	-0,73301	-0,12375	0,030673	-1,47968	-0,47232	-0,40465	-2,22695	-2,98529	-1,14236	4,265089	3,311224	0,689419	0,532905	1,210223	1,168528	3,659184	2,053911	2,719595	0,044892	0,178547

D-imyoi-inositol_1456-tetrakisphosphate_biosynt	-0,08414	-0,06775	-0,07119	-0,08176	0,205318	0,04812	0,030534	0,052567	0,178893	0,322314	0,014981	-0,24513	0,093381	0,161793	0,038153	-0,66006	-0,29293	-0,20159	0,044791	0,178547
Erythropoietin_Pathway	-1,58128	-1,65805	-1,74558	0,457594	-1,4644	-2,98191	-4,95223	-4,27851	-6,07857	-4,82873	-0,60047	0,75233	1,013466	-0,94288	1,957373	-19,1281	-14,4377	-11,3237	0,044835	0,178547
NCI_IL27_mediated_signaling_events_Pathway_(p	-0,01646	-0,14583	-0,08516	-0,48479	-0,27911	-0,77453	-0,61741	-0,63076	-0,79257	1,877922	0,897588	0,948258	-0,51746	0,051631	0,067773	-0,70135	0,097439	0,126439	0,044726	0,178547
reactome_Acyl_chain_remodeling_of_DAG_and_	-0,17112	-0,22946	-0,09725	-0,77177	-0,87609	0,005956	-0,85211	-0,8527	-0,90493	0,025442	-0,07099	0,227224	-0,16862	-0,20878	-0,00662	0,567135	0,141726	0,45256	0,044938	0,178547
biocarta_akt_signaling_Pathway_(Pathway_prote	0,057495	-0,07455	-0,06914	0,403803	0,918792	0,868008	-0,06366	-0,13074	-0,30896	-0,05975	0,195992	-0,27483	0,154164	-0,00224	0,07211	-0,12211	-0,08766	0,130759	0,045231	0,178749
KEGG_D_Glutamine_and_D_glutamate_metabolis	-0,15116	0,032408	-0,0365	-0,15377	0,040707	-0,1979	-0,21937	-0,18048	-0,25018	-0,06364	-0,05766	-0,40854	0,185064	0,076761	0,118579	-1,35569	-0,59371	-0,52301	0,045296	0,178749
KEGG_Proximal_tubule_bicarbonate_reclamation	-0,15116	0,032408	-0,0365	-0,15377	0,040707	-0,1979	-0,21937	-0,18048	-0,25018	-0,06364	-0,05766	-0,40854	0,185064	0,076761	0,118579	-1,35569	-0,59371	-0,52301	0,045296	0,178749
NCI_Netrin_mediated_signaling_events_Pathway_	-0,05442	-0,14422	-0,17698	-1,09068	-0,90072	-1,01956	-1,1609	-1,11686	-1,30941	-1,76147	-0,92474	-1,24157	0,737142	0,721796	0,785262	-2,58396	-2,54146	-2,32402	0,045333	0,178749
purine_deoxyribonucleosides_degradation	-0,01168	-0,02681	-0,03171	-0,00672	-0,2568	0,182295	0,57846	0,647887	0,610253	-0,21157	-0,13967	-0,60615	0,23905	0,137233	0,034955	-0,17458	-0,22322	-0,0227	0,045088	0,178749
reactome_Orc1_removal_from_chromatin_Main_	2,718841	0,836453	0,80894	2,444908	2,348738	-1,56021	3,699343	4,654428	-0,24753	-6,36119	0,098319	-2,21964	0,81561	1,6197	1,476888	-28,5882	-22,9818	-18,1738	0,045215	0,178749
NCI_Signaling_events_mediated_by_PRL_Main_P	0,170193	-0,09802	-0,12094	1,462454	1,07699	-0,66209	0,172282	0,540965	-0,15659	0,00214	-0,49146	-0,64499	-0,28274	0,006301	-0,41822	-4,61722	-4,25675	-3,00134	0,045739	0,17899
phytol_degradation	0,069804	-0,01735	-0,02019	0,213462	0,14781	0,314892	-0,67311	-0,60893	-0,79958	-0,02197	0,491485	0,32116	0,184066	0,174166	0,260225	-0,63533	-0,65931	-0,50969	0,045506	0,17899
reactome_Beta_oxidation_of_myristoyl_CoA_to_	0,056981	0,086345	-0,02253	-0,31498	-0,09333	0,296278	0,215545	0,154902	0,012122	0,121978	-0,33346	0,01932	0,265154	0,206957	0,322971	-1,45866	-0,96394	-1,04832	0,045704	0,17899
reactome_Nicotinamide_salvaging_Main_Pathwa	0,013015	0,114222	-0,05007	-1,45626	-0,42564	-0,95335	-0,79083	-0,88795	-0,70774	0,704171	1,250545	1,007958	0,370994	-0,02205	0,469593	0,385216	0,41756	0,577093	0,045715	0,17899
reactome_Toll_Like_Receptor_TLR6_TLR2_Cascad	0,180261	0,03468	0,147643	-0,15544	-0,15166	-0,11039	-0,21121	-0,22296	-0,01788	0,408653	0,695801	0,128401	-0,04858	-0,69304	0,078743	0,25174	-0,25812	-0,24657	0,045658	0,17899
urea_cycle	-0,19723	-0,15169	-0,06524	-0,15348	-0,55109	0,11458	0,03823	-0,27841	0,095104	0,722412	-0,27809	0,075694	-0,16028	-0,04966	-0,27405	1,18853	0,262091	1,168968	0,045562	0,17899
Erythropoietin_Pathway_Cell_Survival	-0,78914	-0,90687	-1,19135	1,321163	-0,0202	-0,74441	-2,63625	-2,07346	-3,71256	-2,79682	0,265592	3,475578	0,801345	-0,15659	1,690914	-8,14774	-6,11131	-5,73543	0,045928	0,179503
biocarta_role_of_parkin_in_ubiquitin_proteasom	0,064666	-0,04578	-0,03716	-0,34065	0,192401	-0,17061	-1,83182	-1,70025	-2,10782	-0,63532	-1,10399	-0,03893	0,082057	-0,01348	-0,15585	-1,8632	-1,7161	-0,59974	0,046317	0,180799
biocarta_b_cell_survival_Main_Pathway	0,055584	-0,02718	-0,07503	-0,28372	0,962923	-0,38864	0,058525	0,220037	-0,22735	-1,32634	0,009476	-0,34669	0,228642	0,339213	0,320594	-1,28956	-0,69126	0,002704	0,046988	0,182958
fatty_acid_elongation_--_saturated	0,017158	-0,03114	-0,03534	0,23314	0,101395	0,088119	-0,33095	-0,34302	-0,49785	0,283323	0,104696	-0,66382	0,055823	-0,07209	0,074628	-0,76517	-0,51	-0,30691	0,046972	0,182958
biocarta_cdk_regulation_of_dna_replication_Mai	0,958751	0,275789	0,347547	2,78513	1,886353	0,65651	3,599251	4,04907	2,350774	-0,45898	0,958009	-1,40712	-0,48419	-0,10184	-0,20134	-5,4098	-4,50856	-3,00778	0,047078	0,183076
MAPK_Family_Pathway_Chromatin_Remodelling	-0,03853	-0,48632	-0,23989	0,391616	0,149673	-0,80053	1,557825	1,68926	0,76091	-0,15346	0,802697	-0,86426	0,530454	-0,23717	1,758413	-2,68619	-1,99124	-1,76371	0,047253	0,183076
NCI_Downstream_signaling_in_naive_CD8_T_cell:	0,079627	0,434792	0,184254	0,073953	0,049859	0,238605	0,294997	0,029208	0,858338	1,235843	0,656252	0,028832	-0,23629	-0,45093	0,02476	0,222499	-0,08176	0,137392	0,047244	0,183076
reactome_APC_Cdc20_mediated_degradation_of_	0,796039	0,013546	0,149749	-1,24056	-1,60029	-2,37711	1,273635	1,827475	-0,39657	-6,26907	-3,79459	-3,06555	-0,70111	-0,23134	-0,07106	-10,538	-8,83356	-7,56986	0,047156	0,183076
KEGG_GABAergic_synapse_Main_Pathway	0,588687	-0,14382	-0,17213	2,537146	2,496208	2,717257	2,511783	2,221424	1,297946	-1,38789	1,06422	0,391675	-0,46072	-1,3456	-0,81373	-7,50928	-7,75755	-6,32902	0,047396	0,183401
reactome_CREB_phosphorylation_through_the_a	-0,01013	-0,15841	-0,03459	0,307995	0,229639	-0,10161	-0,01414	0,009583	-0,12568	-1,03167	-0,2021	-0,96416	-0,08626	-0,3079	-0,19027	-0,3554	-0,28655	-0,30899	0,047577	0,183872
D-mannose_degradation	0,028283	0,014698	-0,02948	0,32275	0,180183	0,075112	0,193521	0,183881	0,124692	-0,27064	0,02161	-0,0821	-0,09306	0,084832	0,098172	0,157428	-0,17471	-0,07259	0,047779	0,184006
lactose_degradation_III	0,006441	0,049182	0,018555	-0,05965	0,119935	0,072437	-0,35776	-0,34657	-0,38299	-0,26873	-0,03713	0,096674	0,024255	0,038491	-0,0132	-0,40458	-0,28933	-0,20588	0,047766	0,184006
Mitosis_Initiation	0,863659	0,30024	0,09454	0,544499	0,464415	-0,87896	-0,96177	-0,1306	-2,54674	-2,51545	-0,84028	-0,85723	-0,1531	0,124966	0,328118	-9,32116	-6,91735	-6,72384	0,047788	0,184006
BRCA1_Pathway_Chromatin_Remodeling	0,015429	-0,67376	-0,50081	0,35496	0,439432	-0,29813	0,404316	1,045825	-0,8407	-1,6277	0,351118	-0,26634	-0,2747	-0,13662	-0,07542	-6,63301	-3,85623	-2,94047	0,047878	0,184122
biocarta_role_of_mef2d_in_t_cell_apoptosis_Patl	0,028251	0,09714	0,080988	-0,05505	0,209494	0,39176	0,182359	0,248713	0,217232	0,521713	-0,23894	0,700566	-0,41351	-0,15873	-0,26476	0,800802	0,470775	0,438175	0,048309	0,18516
reactome_NRIF_signals_cell_death_from_the_nu	0,379068	0,003458	0,125187	-0,03425	0,780259	0,06094	-0,31562	-0,17022	-0,71842	-0,36516	0,760041	0,489737	0,853336	0,665163	0,855376	-4,76548	-3,0147	-2,97728	0,048253	0,18516
reactome_Synthesis_of_bile_acids_and_bile_salts	-0,3965	-0,10623	0,081555	-1,78362	-0,12684	-1,49698	-2,03543	-2,66465	-1,48335	0,596581	1,074844	0,298636	0,531509	-0,02717	0,635028	2,757975	1,542605	1,490107	0,048326	0,18516
biocarta_overview_of_telomerase_rna_compone	-0,04039	-0,14025	-0,05504	-0,61082	-0,18595	-0,15837	0,369655	0,4537	0,03313	-0,78839	-0,23791	-0,55981	-0,16295	0,210026	0,15818	-2,41239	-1,64477	-1,27779	0,048463	0,185279
reactome_Reuptake_of_GABA_Main_Pathway	0,037342	-0,13282	0,034189	-0,05521	0,506949	0,54682	0,737466	0,909052	1,082002	0,160269	0,666861	0,335621	-0,61264	-0,43533	-0,47158	1,450755	0,966413	1,466082	0,048476	0,185279
reactome_PI3K_AKT_activation_Main_Pathway	-0,20503	-0,17615	-0,18233	0,018283	0,100722	0,173602	-0,6251	-0,68443	-0,65304	0,567617	-0,07678	0,343851	0,277775	0,202511	0,364016	-1,81111	-1,18139	-0,86468	0,048649	0,185487
selenocysteine_biosynthesis	0,070497	0,034534	0,04111	0,10164	0,094118	0,461365	0,357082	0,154047	0,203614	-1,01127	-0,07664	0,124525	-0,15803	-0,14622	-0,17247	-1,03579	-1,24748	-1,16608	0,048609	0,185487
NCI_IL8_and_CXCR2_mediated_signaling_events_	-0,14915	-0,19695	0,053709	-0,40115	0,014656	-1,67357	0,02537	0,120258	0,27657	1,010931	-0,41285	-0,33509	-0,27442	-0,20009	0,218828	1,355805	1,125403	1,679976	0,048736	0,185487
reactome_Cdc20_Phospho_APC_C_mediated_deg	2,508175	0,66025	0,696199	-0,95489	-0,9625	-4,41919	1,396762	2,425627	-2,77682	-10,7198	-4,0114	-4,34255	0,367136	1,235491	1,50444	-31,1687	-25,5403	-21,463	0,048769	0,185487
biocarta_skeletal_muscle_hypertrophy_is_regulat	0,376334	0,139285	0,054088	0,653232	1,039627	-0,10119	0,038977	0,2304	-0,62602	0,397161	-0,13056	0,471981	0,092049	0,333881	0,736506	-3,15803	-2,13772	-1,32719	0,048863	0,185621
ERK_Signaling_Pathway_Gene_Expression_via_CA	-0,19594	-1,14652	-1,03098	-1,88939	-0,59481	-1,40523	-0,78846	-0,14011	-2,33359	-2,0045	0,989576	-2,35287	0,651855	-0,42049	1,485778	-7,94933	-8,03741	-6,63279	0,049354	0,186254
ILK_Signaling_Pathway_Epithelial_Mesenchymal_	-2,53579	-1,07505	-0,39974	-9,11997	-5,90081	-6,67794	-13,0138	-14,1705	-8,87968	4,042289	-1,90367	2,195255	2,566612	0,97965	1,291945	10,59912	8,801721	7,10822	0,049205	0,186254
ILK_Signaling_Pathway_Migration_Vasculogenesis	-2,53579	-1,07505	-0,39974	-9,11997	-5,90081	-6,67794	-13,0138	-14,1705	-8,87968	4,042289	-1,90367	2,195255	2,566612	0,97965	1,291945	10,59912	8,801721	7,10822	0,049205	0,186254
NCI_Arf6_trafficking_events_Pathway_(endocytos	0,002915	-0,20113	-0,12241	-0,11067	-0,72712	0,19796	-0,96319	-0,95961	-0,91937	-0,61246	-1,19126	-1,15103	0,325556	0,231003	0,105138	-0,34605	-0,16698	-0,41512	0,049571	0,186254
NCI_IL23_mediated_signaling_events_Pathway_(I	0,017057	0,036371	0,004857	-1,12856	-0,31191	-0,33541	0,240195	0,130468	0,358989	-0,74573	-0,57923	0,377782	-0,37171	-0,18877	0,130471	1,033903	1,062718	0,988162	0,049633	0,186254
NCI_IL23_mediated_signaling_events_Pathway_(p	0,017057	0,036371	0,004857	-1,12856	-0,31191	-0,33541	0,240195	0,130468	0,358989	-0,74573	-0,57923	0,377782	-0,37171	-0,18877	0,130471	1,033903	1,062718	0,988162	0,049633	0,186254
NCI_IL23_mediated_signaling_events_Pathway_(p	0,017057	0,036371	0,004857	-1,12856	-0,31191	-0,33541	0,240195	0,130468	0,358989	-0,74573	-0,57923	0,377782	-0,37171	-0,18877	0,130471	1,033903	1,062718	0,988162	0,049633	0,186254
NCI_IL23_mediated_signaling_events_Pathway_(p	0,017057	0,036371	0,004857	-1,12856	-0,31191	-0,33541	0,240195	0,130468	0,358989	-0,74573	-0,57923	0,377782	-0,37171	-0,18877	0,130471	1,033903	1,062718	0,988162	0,049633	0,186254
NCI_IL23_mediated_signaling_events_Pathway_(T	0,017057	0,036371	0,004857	-1,12856	-0,31191	-0,33541	0,240195	0,130468	0,358989	-0,74573	-0,57923	0,377782	-0,37171	-0,18877	0,130471	1,033903	1,062718	0,988162	0,049633	0,186254
NCI_Validated_transcriptional_targets_of_TAp63_	0,238137	0,046146	0,286891	-0,293	-0,44335	-0,3152	-1,37493	-1,17434	-1,35431	0,796228	0,62671	-0,17378	0,132119	0,4424	0,532334	0,823752	0,866448	0,904289	0,049527	0,186254
reactome_PD_1_signaling_Main_Pathway	-0,83618	0,561686	0,027327	-0,06904	-0,16834	1,1308	-0,11314	-0,97945	1,454987	0,60883	1,693673	1,794912	0,395377	0,147228	0,292883	4,950888	4,055507	4,767136	0,049688	0,186254
NCI_PLK1_signaling_events_Pathway_(G2_M_tra	0,094348	0,032238	-0,03071	-0,36891	-0,30881	-0,33851	1,20109	1,354293	0,905614	-1,55428	-1,82717	-0,92971	-0,26706	-0,25736	-0,28192	-1,80316	-1,08811	-0,97315	0,049782	0,186382
PTEN_Pathway_Adhesion_or_Migration	1,281796	0,990489	0,982687	-1,59468	0,089054	4,404653	10,96441	11,62888	10,87152	-6,32961	1,134413	4,135421	-2,52894	-3,04639	-4,74098	13,00696	15,45994	11,0645	0,04994	0,186751
KEGG_Basal_transcription_factors_Main_Pathwa	1,279395	0,044055	0,121659	2,486901	1,511264	0,53309	-0,13435	1,338864	-2,42747	-0,97929	0,489995	0,659892	0,507057	0,332072	1,508112	-16,5107	-12,6466	-9,70489	0,050115	0,187182
ATM_Pathway_Apoptosis_and_Senescence	0,039989	-0,12746	-0,18471	0,665071	0,384844	0,240545	-0,05772	0,204134	-0,4567	0,317173	-0,02866	0,177343	0,064733	-0,14546	0,070778	-0,95668	-0,08267	-0,72991	0,050447	0,188195
serotonin_degradation	0,075217	-0,16864	0,098826	-1,73311	-0,6027	-1,30038	1,005824	1,006445	1,158058	0,56622	0,121762	1,131836	-0,22211	0,226523	-0,14031	-0,01147	0,179722	-0,5021	0,05075	0,189052
UDP-iNi-acetyl-D-galactosamine_biosynthesis_I	0,036613	0,030115	0,059557	0,121202	0,159693	0,268994	0,179422	0,096469	0,192161	-0,01048	-0,35028	-0,50202	0,077695	-0,04825	0,233805	0,029073	0,232837	-0,02991	0,050798	0,189052
Glucocorticoid_Receptor_Signaling_Pathway_Ger	0,40858	-0,44499	-0,03022	-0,40968	0,500132	-0,42788	0,128612	0,616811	-0,60741	-2,36607	-0,13147	0,861136	0,308907	0,404916	0,694109	-5,95996	-3,95413	-2,88905	0,051043	0,189738
biocarta_ras_independent_pathway_in_nk_cell_r	0,107657	-0,0639	0,08693	-0,20722	-0,43204	-0,23915	-0,59957	-0,48794	-0,75241	0,009897	0,446979	0,219004	0,222401	0,347864	0,266135	1,011777	1,407883	1,737157	0,051521	0,19015
choline_degradation	0,041723	-0,00701	0,018599	-0,32515	-0,38288	-0,32695	-0,01645	0,067534	-0,08588	0,677112	0,64487	0,37063	0,051853	0,046206	-0,08828	-0,36135	-0,27767	0,190192	0,051311	0,19015
NCI_Downstream_signaling_in_naive_CD8_T_cell:	-0,54312	-0,13526	-0,51982	0,055035	0,2512	-1,52867	-1,17766	-1,6355	-0,97875	1,450695	2,113556	1,534294	-1,17125	-1,34444	0,195122	0,128618	-0,52974	0,558185	0,051407	0,19015
NCI_Signaling_events_mediated_by_PRL_Pathwa	0,00981	0,026437	0,010799	0,812519	0,59674	0,375952	-0,77098	-0,67332	-0,98587	0,024855	0,262777	-0,19697	-0,23183	-0,19981	-0,1227	-1,56824	-1,69306	-1,08852	0,051439	0,19015
reactome_Cohesin_Loading_onto_Chromatin_Ma	0,284142	-0,0044	-0,06346	0,825838	0,510342	0,559374	1,017924	1,310548	0,285414	-0,77113	0,263749	-0,42953	-0,51758	0,039285	-0,15581	-2,88892	-1,86476	-0,6102	0,051259	0,19015
reactome_Ethanol_oxidation_Main_Pathway	0,041602	0,043101	0,033937	-0,43171	-0,24886	-0,48081	1,073155	1,146044	1,180341	0,368919	-0,3257	0,459029	0,104002	0,245872	0,236158	1,54158	0,478724	0,997339	0,051486	0,19015
biocarta_role_of_egf_receptor_transactivation_b	0,1466	-0,07249	-0,00353	-0,21854	-0,41236	-0,2485	-0,18529	-0,10159	-0,25505	0,536475	-0,08454	-0,72952	-0,05408	-0,25095	0,025891	0,726869	0,479431	0,431013	0,051907	0,190897
biocarta_trefoil_factors_initiate_mucosal_healing	0,1466	-0,07249	-0,00353	-0,21854	-0,41236	-0,2485	-0,18529	-0,10159	-0,25505	0,536475	-0,08454	-0,72952	-0,05408	-0,25095	0,025891	0,726869	0,479431	0,431013	0,051907	0,190897
superpathway_of_choline_degradation_to_L-seri	-0,02683	0,137112	-0,02873	-2,13181	-1,96075	-1,40365	0,426749	0,436428	0,455013	1,108681	1,25976	0,705047	0,45843	0,106355	0,078441	0,353531	-0,08732	0,079533	0,051894	0,190897
NCI_RXR_and_RAR_heterodimerization_with_oth	-0,52962	-0,15106	-0,42927	-1,24724	-0,3726	-0,35627	-1,24681	-1,39974	-1,01785	-0,54315	0,619742	0,423094	-0,29863	-0,19871	0,139023	0,841888	1,239813	0,583594	0,052306	0,192138
IGF1R_Signaling_Pathway	-0,12018	-1,31183	-1,37706	2,082327	2,64459	-1,02345	-13,1607	-12,2455	-15,3071	0,105618	3,31428	2,859398	1,673124	0,829689	4,4131	-13,3592	-8,52011	-4,06463	0,052419	0,192325
biocarta_sprouty_regulation_of_tyrosine_kinase_	0,118085	-0,16959	-0,24925	-0,29915	-0,35251	-0,76725	-0,3991	-0,21505	-0,45476	-0,38056	1,039954	0,795688	-0,3484	-0,77006	-0,56697	-0,90983	-0,11445	-0,43545	0,05261	0,192746
NCI_PDGF_receptor_signaling_network_Main_Pa	-0,06439	-0,13261	-0,06189	-1,28497	-1,57584	-1,38869	-1,27148	-1,07678	-1,35219	-0,59693	-1,13568	-0,28626	-0,33743	-0,29954	0,041738	0,294868	0,737635	0,861347	0,052897	0,192746
NCI_PDGF_receptor_signaling_network_Pathway_	-0,06439	-0,13261	-0,06189	-1,28497	-1,57584	-1,38869	-1,27148	-1,07678	-1,35219	-0,59693	-1,13568	-0,28626	-0,33743	-0,29954	0,041738	0,294868	0,737635	0,861347	0,052897	0,192746
p38_Signaling_Pathway_Gene_Expression_Cell_M	-0,2162	-0,48566	-0,57057	-0,98793	-0,2678	-0,07955	-2,49204	-2,18138	-2,91527	-1,25358	0,634833	-0,63174	1,083894	0,762056	1,401154	-1,12158	-1,42445	-0,75297	0,052885	0,192746
reactome_ADP_signalling_through_P2Y_purinoce	0,010601	-0,18541	-0,35131	2,681912	2,271829	1,430076	0,017036	-0,21932	-0,11865	-1,52808	1,479409	1,156664	-0,38168	-0,57824	-0,35809	-3,83726	-4,24567	-2,29261	0,052822	0,192746
reactome_Phenylalanine_and_tyrosine_catabolisi	-0,1457	0,17285	-0,05627	-1,7946	-1,79395	-2,34205	-0,75373	-0,69122	-0,61157	0,962635	-0,31279	-0,19218	-0,69579	0,07778	-0,59338	-0,67332	-0,6775	-1,06175	0,052905	0,192746
biocarta_prion_Main_Pathway	-0,1064	-0,22437	0,056222	-0,58581	-0,02534	0,535128	-3,3784	-3,45757	-3,54035	1,624862	1,287043	0,998297	-0,12264	-0,43765	-0,16741	0,393378	-0,02786	0,082914	0,053135	0,193359
NCI_C_MYC_Main_Pathway	0,225649	-0,20824	-0,25563	-0,1561	-0,08614	-0,27387	-0,1205	0,233905	-0,94794	-2,18221	0,300631	-0,07317	-0,02706	0,109343	0,001451	-5,38562	-4,85146	-4,8705	0,053401	0,1941
ATM_Pathway_Checkpoint_Activation	0,032085	-0,00474	-0,05062	0,121962	0,338306	0,092602	-0,10225	0,006427	-0,2322	0,032121	-0,00106	0,084555	0,080394	-0,01004	-0,09356	-0,6476	-0,38474	-0,18285	0,054059	0,194417
biocarta_sodd_tnfr1_signaling_Main_Pathway	0,087194	-0,1064	0,081384	-0,7196	-0,354	-1,4953	-1,16193	-1,30099	-1,05524	0,152388	0,063058	1,099683	-0,4473	0,243267	-0,32709	-0,21233	-0,01337	-0,32878	0,053608	0,194417
Caspase_Cascade_Pathway	3,280789	2,477885	2,141268	3,758587	1,912174	6,65211	15,74759	16,30248	15,77365	5,015348	6,655193	4,469758	-4,49275	-3,41959	-6,61475	8,363146	9,15555	3,472678	0,054226	0,194417
Growth_Hormone_Signaling_Pathway_Cell_Surviv	-0,32317	-0,42654	-0,34271	0,708567	0,830722	0,848353	0,546766	1,165046	-0,05108	2,113818	-0,49936	1,867472	0,573669	1,128685	1,225325	-2,53226	-1,70961	-0,27251	0,053962	0,194417
Growth_Hormone_Signaling_Pathway_Glucose_U	-0,49226	-0,47555	-0,24677	-0,02742	-0,23253	0,529099	1,320242	1,827232	0,922149	0,95749	-0,37749	1,255241	0,219135	0,920538	1,304912	-2,53094	-1,87075	-1,11196	0,054174	0,194417
KEGG_Malaria_Main_Pathway	-0,06241	-0,02218	0,092339	-0,56064	0,183114	-0,51059	-1,41526	-1,55671	-1,3611	1,162764	0,972841	0,851022	-0,29587	-0,67801	-0,52877	-0,10147	-1,03606	-0,31523	0,054237	0,194417
malate-aspartate_shuttle	-0,05733	0,023398	-0,01842	-0,06795	-0,56765	-0,31564	-0,05958	-0,01322	-0,17978	-0,71657	-0,28882	-0,1646	0,274614	0,21263	0,294448	-0,91095	-1,08258	-1,15228	0,054208	0,194417
NCI_ATM_Pathway_(apoptosis)	0,072899	0,018742	-0,02978	0,286508	0,353468	0,651282	0,147041	0,141075	-0,08983	-0,30914	0,205494	0,278005	0,026144	-0,03493	-0,12504	-0,80081	-0,37322	-0,62063	0,054176	0,194417
NCI_Canonical_NF_kappaB_Main_Pathway	0,264556	-0,5053	-0,16968	-0,65609	-0,30481	-0,80374	-2,16357	-1,54419	-2,67873	-0,27159	-1,30617	-1,1193	-0,08136	-0,13266	0,701815	-4,70446	-3,64917	-2,3489	0,054164	0,194417
reactome_Activation_of_NF_kappaB_in_B_cells_I	1,906999	0,608919	0,565419	0,144433	0,2808	-1,97286	0,343482	0,987814	-2,26486	-3,83602	-0,43694	-1,4598	1,469534	1,487903	2,05791	-21,1593	-17,1242	-14,4951	0,053816	0,194417
reactome_HuR_stabilizes_mRNA_Main_Pathway	-0,02567	-0,20941	-0,13806	-0,38652	-0,44671	-0,54264	-0,98217	-0,78421	-1,16331	-1,25291	-0,34075	-0,47193	0,219846	0,260701	0,260773	-1,93557	-1,11171	-0,50966	0,054154	0,194417
reactome_Unwinding_of_DNA_Main_Pathway	0,444186	0,280814	0,221851	1,06419	0,950988	1,03887	3,727469	4,024205	3,018052	-0,98363	-0,64777	-0,79692	-0,55774	-0,77813	-0,43478	-3,44668	-1,90862	-1,87697	0,05415	0,194417
reactome_Formation_of_tubulin_folding_interme	0,187032	-0,35117	0,064293	0,143804	-0,66459	-0,97791	-1,53765	-1,39717	-1,73892	-2,11378	-1,58119	-0,91077	-0,26644	-0,58967	-0,23704	-7,09876	-5,43006	-4,70574	0,054486	0,194785
reactome_JNK_c_Jun_kinases_phosphorylation_a	0,132735	0,187801	0,069541	-0,28688	-0,12301	0,198385	0,194167	0,362447	-0,15485	-1,00899	0,157474	-0,15117	0,552425	0,378384	0,574297	-0,73368	-0,68148	-0,04231	0,054527	0,194785
reactome_Purine_ribonucleoside_monophosphat	0,186922	0,07855	0,027472	-0,5395	0,123213	-0,12859	-0,22698	0,011915	-0,64305	-0,22091	-0,36551	-1,03132	-0,07985	0,053386	0,331776	-2,91744	-2,36867	-1,67927	0,054498	0,194785
BRCA1_Pathway_Growth_Promoting_Genes_hTe	0,060939	-0,01354	-0,06223	0,548078	0,739161	0,154625	0,247518	0,527654	-0,13229	0,500146	0,11908	0,011833	-0,05245	0,050078	0,013126	-1,4546	-0,69733	-1,06115	0,05476	0,194946
NCI_VEGF_and_VEGFR_signaling_network_Main_	-0,10015	-0,02816	-0,05211	-0,5512	0,14072	-0,05347	-2,36148	-2,55627	-2,2511	0,357872	-1,58557	-0,78666	0,555705	0,522741	0,138797	-0,50567	-0,36723	-0,56854	0,054718	0,194946
reactome_Signaling_by_ERBB4_Main_Pathway	-0,03073	-0,00881	0,463769	-1,22585	-1,26726	-2,021	-0,85345	-0,72287	-0,51367	0,741781	0,420989	-0,39239	-0,08265	0,100383	-0,07902	0,253275	-0,3153	-0,06347	0,054729	0,194946
biocarta_rb_tumor_suppressor_checkpoint_signa	0,22795	-0,06159	-0,00379	1,147786	0,575863	0,660238	1,278616	1,399848	1,032368	-0,03484	-0,03018	-0,41643	-0,10838	-0,27062	-0,05783	-0,95313	-0,6136	-0,40794	0,05517	0,196021
PAK_Pathway_Actin_Organization	-6,07835	-1,57477	-0,44427	-10,8094	-3,37466	-10,2683	-7,52142	-11,69	0,0509	7,937854	-7,92645	-2,26322	-0,49337	-0,56851	0,723669	21,66689	15,85709	14,09732	0,055251	0,196021
PAK_Pathway_Cell_Survival	-6,07835	-1,57477	-0,44427	-10,8094	-3,37466	-10,2683	-7,52142	-11,69	0,0509	7,937854	-7,92645	-2,26322	-0,49337	-0,56851	0,723669	21,66689	15,85709	14,09732	0,055251	0,196021
NCI_PDGFR_alpha_signaling_Pathway_(positive_r	-0,09039	-0,17898	-0,1199	-1,9739	-2,2246	-1,53881	-3,38559	-3,28705	-3,63102	0,208856	-0,39032	0,149531	-0,18469	-0,12781	-0,41573	-0,66379	-0,34301	0,351751	0,05549	0,1962
NCI_PDGFR_alpha_signaling_Pathway_(Ras_prote	-0,09039	-0,17898	-0,1199	-1,9739	-2,2246	-1,53881	-3,38559	-3,28705	-3,63102	0,208856	-0,39032	0,149531	-0,18469	-0,12781	-0,41573	-0,66379	-0,34301	0,351751	0,05549	0,1962
reactome_Activation_of_ATR_in_response_to_re	1,240083	0,09202	0,474231	3,366832	2,463728	1,283359	6,563608	8,117043	4,413987	-0,2199	-1,12418	-1,27128	-1,39485	-0,77546	-0,60746	-13,3404	-8,97841	-6,52365	0,055405	0,1962
adenine_and_adenosine_salvage_III	-0,08544	-0,11765	-0,02288	-0,21692	-0,2268	0,049327	0,835816	0,892037	0,823728	-0,47499	0,088985	-0,54732	0,132485	0,233508	0,093682	-0,32196	-0,28474	-0,14056	0,055582	0,1963
reactome_Fatty_Acyl_CoA_Biosynthesis_Main_Pa	0,012127	-0,04741	-0,00024	-0,1182	-0,05871	0,144447	0,23402	0,189041	0,063071	-0,20268	0,35706	-0,58111	0,148941	-0,10102	0,0866	-1,00661	-0,78105	-0,77555	0,055807	0,196874
4-hydroxy-2-nonenal_detoxification	-0,10445	0,000717	-0,02233	-1,72552	-1,09734	-1,27173	0,227374	0,2256	0,14316	0,548294	0,440475	0,489634	-0,25183	-0,13964	-0,26614	-0,24164	-0,85742	-0,97682	0,056006	0,197353
NCI_Regulation_of_RhoA_activity_Main_Pathway	-0,40959	-0,40366	-0,35474	-1,17382	-0,24619	-0,07886	-0,13748	-0,72057	-0,40816	-5,36896	-0,53125	-1,21824	1,632196	0,821411	1,158561	-0,10257	-1,09622	-0,39951	0,056218	0,197876
reactome_DAP12_interactions_Main_Pathway	-0,47491	0,597115	0,51402	0,601417	0,963828	0,745812	0,032451	-0,65681	0,948348	1,851036	1,438435	0,937915	-0,91172	-0,66588	-1,3095	1,196475	0,752268	1,511951	0,056358	0,198142
NCI_N_cadherin_signaling_events_Main_Pathwa	0,001298	-0,30148	-0,25256	0,284488	0,355419	-0,97994	-1,89514	-1,69302	-2,62854	-0,22759	2,148383	-1,0081	0,981274	0,779407	1,449908	-6,96182	-5,08728	-4,40864	0,056508	0,198448
reactome_Recruitment_of_NuMA_to_mitotic_cer	0,265726	0,106971	-0,08341	-0,2956	0,069662	0,623186	0,986636	1,308905	0,267647	-0,18269	-0,74373	0,197986	-0,13479	0,034713	0,410095	-2,97605	-2,45675	-1,33524	0,056578	0,19847
reactome_SHC_mediated_signalling_Main_Pathw	-0,09155	-0,18802	-0,20877	-0,78936	-0,49548	-0,68426	-0,99	-1,0206	-1,18436	-1,14095	-0,1511	-0,8803	0,447379	0,22681	0,384518	-2,39386	-1,75934	-1,2861	0,056728	0,19877
JNK_Pathway	-3,6082	-2,12946	-1,08726	-8,33034	-0,45001	-9,2171	-16,6522	-19,6459	-11,1399	4,046286	0,333153	2,232949	1,850391	0,79858	1,87132	13,29095	10,36902	16,68205	0,056868	0,199001
NCI_Fanconi_anemia_Main_Pathway	1,052801	-0,03427	-0,14218	3,551318	4,489723	2,983087	5,423072	7,074588	2,868044	-1,09143	1,830296	0,08753	-1,43243	-0,97732	-0,60203	-13,7361	-9,19783	-6,49434	0,057113	0,199001
NCI_Retinoic_acid_receptors_mediated_signaling	0,109247	-0,21667	-0,64458	-1,08552	-0,47715	-0,86878	-1,31907	-0,62481	-2,40604	-1,78758	-0,44003	-1,74257	-0,3963	-0,0547	0,292856	-6,82207	-3,84363	-4,27801	0,056957	0,199001
reactome_APC_C_Cdh1_mediated_degradation_c	2,405971	0,754787	0,615376	-0,445	-0,9025	-3,71332	2,459821	3,215457	-1,50293	-9,00763	-2,35936	-1,87907	0,571597	1,36037	1,961534	-29,2586	-24,6815	-20,8731	0,057103	0,199001
reactome_CDT1_association_with_the_CDC6_ORC	2,355588	0,880501	0,651836	1,453911	1,190041	-1,54211	1,795403	2,6119	-1,66888	-4,81	1,062108	-1,0179	1,119674	1,814551	1,742981	-25,2368	-20,0479	-16,1461	0,057015	0,199001
NCI_HIV_1_Nef_Negative_effector_of_Fas_and_T	0,01944	-0,17415	-0,02507	0,095651	-0,31136	-1,09041	-1,75936	-1,78273	-1,71311	0,883782	0,16785	1,57001	-0,29855	0,055206	-0,43053	-0,39195	0,31262	-0,24029	0,057804	0,199313
reactome_Asymmetric_localization_of_PCP_prote	-0,24267	-0,47784	-0,24406	-0,80987	0,075756	-1,35617	0,558722	0,634573	0,56032	-2,99283	-0,72249	-0,6274	0,28966	0,860351	0,207435	-2,25546	-1,29019	-0,88402	0,057802	0,199313
reactome_HIV_Transcription_Initiation_Main_Pat	0,87022	-0,27695	-0,19789	1,98279	0,774052	-0,57882	-0,74578	0,501956	-2,85716	-2,48982	-0,40108	1,194933	-0,18917	-0,1787	0,717003	-15,504	-12,3249	-10,075	0,057842	0,199313
reactome_RNA_Polymerase_II_HIV_Promoter_Es	0,87022	-0,27695	-0,19789	1,98279	0,774052	-0,57882	-0,74578	0,501956	-2,85716	-2,48982	-0,40108	1,194933	-0,18917	-0,1787	0,717003	-15,504	-12,3249	-10,075	0,057842	0,199313
reactome_RNA_Polymerase_II_Promoter_Escape	0,87022	-0,27695	-0,19789	1,98279	0,774052	-0,57882	-0,74578	0,501956	-2,85716	-2,48982	-0,40108	1,194933	-0,18917	-0,1787	0,717003	-15,504	-12,3249	-10,075	0,057842	0,199313
reactome_RNA_Polymerase_II_Transcription_Init	0,87022	-0,27695	-0,19789	1,98279	0,774052	-0,57882	-0,74578	0,501956	-2,85716	-2,48982	-0,40108	1,194933	-0,18917	-0,1787	0,717003	-15,504	-12,3249	-10,075	0,057842	0,199313
reactome_RNA_Polymerase_II_Transcription_Init	0,87022	-0,27695	-0,19789	1,98279	0,774052	-0,57882	-0,74578	0,501956	-2,85716	-2,48982	-0,40108	1,194933	-0,18917	-0,1787	0,717003	-15,504	-12,3249	-10,075	0,057842	0,199313
reactome_RNA_Polymerase_II_Transcription_Pre	0,87022	-0,27695	-0,19789	1,98279	0,774052	-0,57882	-0,74578	0,501956	-2,85716	-2,48982	-0,40108	1,194933	-0,18917	-0,1787	0,717003	-15,504	-12,3249	-10,075	0,057842	0,199313
reactome_SUMO_is_conjugated_to_E1_UBA2_SA	0,267992	0,13045	0,07724	0,263707	0,267056	0,10062	0,302089	0,420046	0,19468	-0,33611	-0,021	0,001122	-0,04199	0,150074	0,122334	-2,23069	-1,67595	-0,875	0,057294	0,199313
reactome_Transcription_of_the_HIV_genome_M	0,87022	-0,27695	-0,19789	1,98279	0,774052	-0,57882	-0,74578	0,501956	-2,85716	-2,48982	-0,40108	1,194933	-0,18917	-0,1787	0,717003	-15,504	-12,3249	-10,075	0,057842	0,199313
KEGG_p53_signaling_Main_Pathway	0,223202	0,052597	0,022036	1,032201	1,354099	0,962458	0,095142	0,467506	-0,5846	1,005626	0,128935	0,019311	-0,19453	-0,03504	0,126225	-2,0439	-0,88994	-1,25982	0,058078	0,199903
Base_Excision_Repair_Pathway	0,435849	0,099325	0,082888	-1,74703	-0,85077	0,451106	0,119293	0,779629	-0,45223	-2,78047	0,572306	-0,67295	0,627644	1,209835	1,417713	-4,82073	-4,21846	-3,57267	0,058338	0,199914
biocarta_transcription_regulation_by_methyltran	-0,11786	-0,16665	-0,2846	-0,42718	-0,07292	0,382027	-1,20038	-1,04961	-1,41156	-0,48609	-0,07135	-0,17388	0,237758	0,135894	0,41456	-0,84564	-0,13449	0,179773	0,058333	0,199914
biocarta_transcription_regulation_by_methyltran	-0,11786	-0,16665	-0,2846	-0,42718	-0,07292	0,382027	-1,20038	-1,04961	-1,41156	-0,48609	-0,07135	-0,17388	0,237758	0,135894	0,41456	-0,84564	-0,13449	0,179773	0,058333	0,199914
reactome_Synthesis_of_UDP_N_acetyl_glucosam	0,196013	0,030854	0,040002	0,36085	0,021387	0,377803	-0,76572	-0,59456	-1,01214	0,23709	-0,36212	0,32665	-0,02078	0,430718	-0,06821	-2,25118	-1,7301	-1,54159	0,058247	0,199914
reactome_STING_mediated_induction_of_host_ir	-0,05894	0,069173	0,037137	0,24271	0,109597	-0,25514	-0,309	-0,24327	-0,48302	0,789281	0,728051	0,46576	-0,08811	-0,22501	-0,25997	-0,10469	-0,09149	0,164865	0,058531	0,200355

NCI_Calcineurin_regulated_NFAT_dependent_tra	-0,8115	-0,73595	-0,73113	-0,46731	-0,35399	-0,67725	-0,97888	-1,42212	-0,9007	1,806267	1,541078	0,943929	-0,8113	-0,0533	0,234639	0,342579	0,812461	0,573129	0,05866	0,200576
NCI_Regulation_of_nuclear_beta_catenin_signalir	-0,29448	-0,71712	-0,67192	-0,6238	-1,66595	-2,14121	-1,06913	-0,74288	-1,61318	-0,54419	-1,81359	-1,7021	0,751669	0,424645	2,182353	-6,30536	-4,76696	-2,75497	0,058748	0,200656
biocarta_akap95_role_in_mitosis_and_chromosor	-0,10214	-0,32888	-0,29247	-1,18874	-0,06344	-0,99663	0,384279	0,431154	-0,40966	-1,7649	-1,72811	-0,90242	-0,86088	-0,40394	-0,11936	-6,17229	-4,82003	-4,74901	0,059364	0,202097
biocarta_akap95_role_in_mitosis_and_chromosor	-0,10214	-0,32888	-0,29247	-1,18874	-0,06344	-0,99663	0,384279	0,431154	-0,40966	-1,7649	-1,72811	-0,90242	-0,86088	-0,40394	-0,11936	-6,17229	-4,82003	-4,74901	0,059364	0,202097
biocarta_akap95_role_in_mitosis_and_chromosor	-0,10214	-0,32888	-0,29247	-1,18874	-0,06344	-0,99663	0,384279	0,431154	-0,40966	-1,7649	-1,72811	-0,90242	-0,86088	-0,40394	-0,11936	-6,17229	-4,82003	-4,74901	0,059364	0,202097
NCI_BARD1_signaling_events_Main_Pathway	0,683326	0,270689	0,139361	0,942241	1,396527	1,020392	1,135758	1,629662	-0,13776	-0,75818	-0,26757	-1,1988	-0,56691	-0,5579	-0,46216	-3,78499	-2,21437	-1,32173	0,059618	0,20274
p38_Signaling_Pathway	-8,42645	-2,92108	-2,295	-15,3597	-5,93265	-17,6572	-26,6932	-32,7037	-14,2863	17,40455	-7,15985	-4,42269	3,395538	1,437381	3,217676	34,42606	30,79891	30,3324	0,059818	0,203198
KEGG_Type_I_diabetes_mellitus_Main_Pathway	-0,03177	1,095744	0,346206	-1,96643	-0,36065	1,295544	-0,26392	-1,10363	1,988795	2,207332	-1,10377	2,151641	0,55828	1,19678	0,301099	3,275232	3,923309	5,144905	0,059989	0,203553
NCI_p53_Main_Pathway	0,623416	-0,15815	-0,24671	1,077851	1,427934	0,475879	-0,82952	0,026406	-2,45168	-0,77071	0,801914	-0,38108	-0,63829	-0,11934	-0,01189	-10,3232	-6,99617	-6,89349	0,060053	0,203553
KEGG_Rheumatoid_arthritis_Main_Pathway	-0,16381	-0,01543	-0,03785	-0,73525	-0,37464	0,205384	0,334104	0,311909	0,500731	1,623422	-1,03053	-0,12818	-0,52249	-0,05903	-0,34921	1,003966	1,168027	0,597435	0,060409	0,204536
reactome_Synthesis_of_IP3_and_IP4_in_the_cytc	-0,01022	-0,11309	0,097576	-0,2108	-0,9374	0,057068	-0,05546	-0,39921	-0,5155	0,016024	0,474008	0,098155	-0,15522	0,17402	0,085666	0,378899	0,569122	0,792572	0,060535	0,204739
NCI_Signaling_events_regulated_by_Ret_tyrosine	-0,01757	0,113825	0,065089	-0,41519	-0,18208	0,067482	-2,16129	-2,2742	-2,02698	0,372716	-0,35977	0,949496	-0,00295	-0,1921	-0,18773	0,104638	-0,24685	0,386181	0,060635	0,204858
reactome_Telomere_Extension_By_Telomerase_I	-0,20303	-0,31576	-0,31917	-0,20939	-0,04307	0,001343	0,148237	0,291208	-0,12019	-0,3271	0,627717	-0,3475	-0,17638	0,166824	0,026607	-1,31042	-1,15232	-0,85319	0,061116	0,206257
biocarta_pelp1_modulation_of_estrogen_receptc	-0,04853	-0,10033	-0,07917	0,134012	0,465835	0,430286	-0,47655	-0,49347	-0,52507	-0,01233	-0,38813	0,02097	0,052752	0,174501	0,093475	0,105097	-0,02454	0,542604	0,061398	0,206508
HIF1Alpha_Pathway_Gene_Expression_via_JUN_(	-0,126	-0,44885	-0,30843	0,305781	1,374061	-0,28615	-1,65692	-1,3574	-1,82597	-0,14232	0,017419	1,379694	0,676801	-0,00742	0,281634	-2,8401	-2,209	-0,69388	0,061424	0,206508
KEGG_Glycosaminoglycan_biosynthesis_heparan_	-0,72832	-0,38879	-0,91086	0,337878	-1,23843	-0,71371	-0,22329	-0,09979	-0,19246	-2,03928	-0,9212	0,670949	1,339781	-0,31167	0,395242	-1,33486	-1,77677	-1,66453	0,06144	0,206508
reactome_Transcription_coupled_NER_TC_NER_	0,045929	-0,1528	-0,19551	0,317261	-0,69533	-0,42363	-0,15698	0,227206	-0,50236	-1,60857	-1,05964	0,19338	-0,38672	-0,23051	-0,14238	-3,97554	-3,01083	-2,73586	0,061455	0,206508
biocarta_nfkb_activation_by_nontypeable_hemo|	0,182521	-0,35788	-0,18085	0,543997	0,886284	0,642198	-1,20541	-0,85465	-1,4333	1,425175	0,652558	-0,06722	0,382711	0,010931	0,673848	-0,37469	-0,76023	-0,63581	0,061897	0,207769
L-carnitine_biosynthesis	0,046739	-0,10469	-0,16363	0,13154	-0,53789	-0,16005	1,112482	1,244622	0,79516	-0,1076	0,097492	-0,32069	0,28098	0,233789	0,463482	-1,80918	-0,81962	-0,83968	0,062059	0,207968
reactome_O_glycosylation_of_TSR_domain_conti	-0,92872	-0,421	0,131881	-2,38441	-1,05186	-1,81428	-3,22503	-3,3495	-0,79466	1,160697	1,232556	-0,08674	0,801912	0,555305	1,350278	6,165091	5,254711	3,08858	0,06209	0,207968
biocarta_fc_epsilon_receptor_i_signaling_in_mas	-0,05042	-0,25519	-0,12912	-0,27351	-0,54537	-0,77939	-0,48815	-0,39573	-0,43421	-0,13234	0,254666	0,537519	0,114656	-0,14655	0,006843	-0,04937	0,135588	0,407951	0,062547	0,208601
NCI_Validated_transcriptional_targets_of_TAp63_	0,200149	0,088743	0,291474	-0,44851	-0,62642	-0,39308	-1,63415	-1,50228	-1,60489	0,892152	0,626856	-0,09247	0,18603	0,513488	0,564641	0,75353	0,911497	0,794223	0,062491	0,208601
NCI_Validated_transcriptional_targets_of_TAp63_	0,200149	0,088743	0,291474	-0,44851	-0,62642	-0,39308	-1,63415	-1,50228	-1,60489	0,892152	0,626856	-0,09247	0,18603	0,513488	0,564641	0,75353	0,911497	0,794223	0,062491	0,208601
reactome_APC_C_Cdc20_mediated_degradation_	0,681226	-0,01923	0,021608	-0,86466	-1,15237	-2,22948	1,371633	1,913446	-0,06283	-4,95189	-2,06579	-1,8958	-0,62076	-0,12759	-0,03283	-9,73117	-8,44034	-7,06358	0,062384	0,208601
reactome_Antigen_activates_B_Cell_Receptor_B(	-0,00321	-0,19294	0,07708	0,906602	1,395185	-0,08302	0,418016	-0,1866	0,658152	-0,48384	0,161884	0,688301	0,253027	-0,57765	0,07275	-2,13011	-1,33586	-1,83636	0,062722	0,208962
NCI_p75_NTR_mediated_signaling_Pathway_(acti	-0,03409	0,096822	0,065579	0,504357	0,547527	0,691753	-0,56754	-0,58134	-0,63079	-0,30777	0,633578	0,19864	0,149383	-0,071	-0,05775	-0,70268	-0,30817	-0,50637	0,062814	0,209043
biocarta_atm_signaling_Main_Pathway	-0,00254	-0,11244	-0,25558	2,078005	1,629631	1,387654	0,725681	1,383184	0,125382	-0,20613	0,725178	0,061228	-0,30092	-0,49703	-0,44546	-3,02962	-1,43019	-1,40425	0,063383	0,209594
biocarta_induction_of_apoptosis_through_dr3_ai	-0,00164	-0,13568	-0,07256	-0,87791	-1,06305	-1,83957	-1,84177	-1,67317	-1,58831	0,03159	-0,19657	1,351515	0,082067	-0,05772	-0,38587	-0,05945	-0,1484	-0,43655	0,063107	0,209594
isoleucine_degradation	0,203953	0,08497	0,131978	-0,49664	0,844384	0,524457	0,823011	1,05954	0,476942	0,160184	-0,08282	-0,2118	-0,17133	0,289337	0,237966	-4,31752	-3,30747	-2,75257	0,063348	0,209594
KEGG_Carbohydrate_digestion_and_absorption_I	-0,09827	-0,02245	0,027533	-0,71492	0,250572	-0,82404	0,131842	0,158115	0,674349	-0,49544	0,247829	0,048	-0,02709	-0,13931	0,292392	1,863585	1,751055	1,517117	0,063264	0,209594
reactome_Cyclin_B2_mediated_events_Main_Pat	0,214585	0,125996	0,1196	0,062339	-0,2643	-0,50437	0,836191	1,064885	0,609901	-0,36305	-1,79408	-1,07688	-0,05095	-0,17191	0,050906	-1,91981	-1,02142	-0,99262	0,063315	0,209594
reactome_Transport_of_nucleosides_and_free_p	-0,32029	0,06725	-0,10092	0,315387	-0,96357	-0,16291	0,409953	0,247994	0,929661	0,231607	0,558019	0,515873	0,143968	0,322507	0,182239	2,360804	1,573687	1,483408	0,063335	0,209594
KEGG_Cholinergic_synapse_Main_Pathway	-0,2968	-0,72759	-0,83281	3,093657	3,204382	1,371454	1,521131	1,590951	0,450293	-0,26907	2,085231	1,425413	-1,23331	-0,33978	0,156031	-7,5913	-5,70611	-2,45647	0,063452	0,209601
biocarta_cxcr4_signaling_Main_Pathway	-0,15601	-0,03214	-0,15908	0,6875	-0,2381	-0,30801	-0,62056	-0,41278	-0,70528	1,435044	-0,35096	-0,6764	0,095005	0,290534	0,741343	-4,23134	-4,27702	-3,05947	0,063794	0,210507
KEGG_Cysteine_and_methionine_metabolism_M	0,114011	-0,06486	-0,17815	-0,01757	-1,44688	-0,37385	0,332414	0,854007	0,112053	-0,65115	-0,42422	-0,17788	0,758535	0,54477	0,561249	-4,14974	-3,58893	-3,0104	0,063877	0,210557
Akt_Pathway_Regulation_by_GH	-0,47618	-0,90417	-0,86136	-0,21703	0,253018	-1,99535	-2,60222	-2,31877	-4,06182	-1,3412	1,007199	0,460563	0,457861	1,011197	1,635643	-7,58086	-4,35719	-3,23674	0,064095	0,210829
reactome_Negative_regulation_of_the_PI3K_AKT	0,024036	-0,03634	-0,16464	1,09177	1,434595	0,320719	-1,52513	-1,52283	-1,99845	0,298444	0,508725	-0,23331	0,342355	0,001382	0,274888	-0,08811	-0,54921	0,478443	0,06404	0,210829
NCI_LKB1_signaling_events_Main_Pathway	-0,00856	-0,29772	-0,32859	-0,0786	0,230726	-1,94629	-0,95072	-0,60286	-1,03333	-2,55298	0,702289	-0,44695	0,679951	0,823207	0,407361	-3,25696	-3,32727	-2,29658	0,064165	0,210837
NCI_IL8_and_CXCR1_mediated_signaling_events_	-0,08674	-0,19752	0,128439	-0,30894	0,053143	-1,23793	-0,2129	-0,1573	-0,03748	1,416681	-0,10592	0,353673	-0,06463	0,198227	0,442608	0,879197	0,834981	1,204852	0,064323	0,211134
biocarta_sprouty_regulation_of_tyrosine_kinase_	0,039008	0,108183	0,00149	0,207674	0,344658	0,017658	0,618155	0,697972	0,746449	-0,12855	0,073621	0,196377	-0,38796	-0,33218	-0,51098	0,779132	0,962773	0,473373	0,064648	0,211318
EGF_Pathway_EGFR_Endocytosis	-0,02604	-0,06643	-0,11898	-0,04667	0,111245	-0,37447	0,7073	0,832661	0,43897	-1,12635	-0,31777	-0,40029	-0,19259	-0,26738	-0,08914	-0,73618	-0,57206	-0,43287	0,064922	0,211318
NCI_Endogenous_TLR_signaling_Pathway_(regula	0,051097	0,10742	-0,00329	-0,01732	0,041911	-0,51113	-1,26509	-1,06988	-0,84825	0,967153	1,099421	0,135295	-0,27681	-0,79845	-0,00907	0,118526	-0,50376	-0,28491	0,064721	0,211318
NCI_Endogenous_TLR_signaling_Pathway_(regula	0,051097	0,10742	-0,00329	-0,01732	0,041911	-0,51113	-1,26509	-1,06988	-0,84825	0,967153	1,099421	0,135295	-0,27681	-0,79845	-0,00907	0,118526	-0,50376	-0,28491	0,064721	0,211318
NCI_Notch_signaling_Main_Pathway	-0,06372	0,061452	-0,2903	-1,19687	1,093209	0,733191	-0,5269	0,038211	-1,60615	-0,65785	-0,09501	-0,8655	-0,17636	0,101511	-0,03199	-6,23335	-2,87967	-2,20102	0,064533	0,211318
NCI_PDGFR_beta_signaling_Pathway_(ruffle_orgi	-0,18735	-0,32044	-0,50864	0,461905	-0,9512	-0,69934	-1,1794	-1,19625	-1,84495	-1,22604	-0,26269	-0,38454	-0,35138	0,03775	0,926499	-7,94705	-4,81014	-3,03291	0,064912	0,211318
reactome_The_NLRP3_inflammasome_Main_Patl	0,128001	0,068229	0,114187	-0,40039	0,104777	-0,99742	-0,15965	-0,28499	-0,3945	-1,08463	-0,58253	-1,57678	-0,12417	-0,37979	0,22641	-2,87184	-1,99474	-2,1476	0,064573	0,211318
VEGF_Pathway_Focal_Adhesion_Turnover	0,006604	0,000506	-0,08566	-0,9518	-0,25452	-0,43763	-0,80132	-0,79721	-0,80796	0,294548	0,456176	0,028402	0,25351	0,161917	0,099159	0,710757	0,758533	0,254914	0,064898	0,211318
adenosine_nucleotides_degradation	-0,0234	0,037719	-0,07417	0,737858	0,103224	-0,5396	1,054295	1,110858	0,926491	-0,93038	-0,50858	-0,46333	0,268831	-0,09306	0,229286	-0,4423	-0,41264	0,016561	0,065305	0,212115
NCI_Fanconi_anemia_Pathway_(Sister_Chromatic	0,682763	-0,03141	-0,11221	1,766635	2,421504	0,92055	3,382572	4,492792	1,865857	-0,00636	0,686862	-0,11306	-0,60376	-0,6093	-0,2323	-9,19215	-6,40569	-4,5781	0,065306	0,212115
reactome_Cross_presentation_of_soluble_exoger	1,6159	0,97878	0,786763	-0,45418	0,241129	-1,63379	0,411122	0,628184	-1,88759	-1,69131	-1,0124	-0,28787	1,40486	2,064265	1,93001	-17,7568	-15,1256	-12,5959	0,065371	0,212115
KEGG_Retrograde_endocannabinoid_signaling_M	0,727065	-0,00692	0,074612	2,85372	2,210547	2,051358	2,070762	2,167626	1,422245	0,380055	2,887366	-0,03883	0,278834	-0,60898	-0,05701	-5,99232	-6,64199	-4,12579	0,065864	0,21305
reactome_Calmodulin_induced_events_Main_Pat	0,000123	-0,21533	-0,00985	-0,32837	-0,45095	-0,70203	-0,38688	-0,27899	-0,29826	-0,30035	-0,23586	-0,59102	-0,37682	-0,23679	0,027291	0,269441	0,194784	0,359894	0,06573	0,21305
reactome_Creatine_metabolism_Main_Pathway	0,031768	-0,10947	-0,04933	-0,00249	0,485788	0,62259	-0,84324	-0,76972	-0,55481	1,313853	0,453456	0,452856	-1,56912	-1,07644	-0,89363	0,709071	0,578384	0,830822	0,065834	0,21305
CXC_Chemokine_Receptor_Pathway	-0,38844	-0,88405	-0,95747	1,558982	0,771686	-0,53976	-2,97371	-3,00305	-3,60361	-2,04071	0,262608	1,730995	-0,9667	-0,61979	0,244048	-6,98164	-5,99226	-3,21994	0,06609	0,213337
reactome_Synthesis_of_PIPs_at_the_ER_membra	0,05161	0,054825	-0,04948	0,131192	0,172425	0,252947	-0,7277	-0,61593	-0,94034	-0,15486	-0,2479	-0,06221	-0,25486	-0,13804	-0,17166	-1,28502	-1,06047	-0,73083	0,066022	0,213337
reactome_Synthesis_of_PIPs_at_the_early_endos	0,217438	-0,14291	-0,27543	-0,821	-1,17007	0,049118	-0,61206	-0,09274	-1,27558	-3,06329	-1,44412	-1,66661	-0,66581	-0,30528	-0,45956	-2,87514	-2,01039	-1,6268	0,066179	0,213402
NCI_IFN_gamma_Main_Pathway	0,322933	-0,18998	-0,41785	0,431562	0,250275	0,134149	-1,5953	-1,00031	-2,4664	-1,96981	0,82606	-0,62501	-0,2639	-0,17603	0,739348	-3,74179	-1,61173	-0,06575	0,066365	0,213782
NCI_S1P5_Main_Pathway	0,034551	-0,07978	-0,10855	0,70878	-0,00176	0,182091	-0,31761	-0,27942	-0,3018	-0,2068	0,562503	-0,29288	-0,04204	-0,34879	-0,00203	-2,01666	-2,32522	-1,83009	0,066678	0,214346
reactome_PI3K_events_in_ERBB2_signaling_Mair	-0,05955	-0,05612	-0,15368	-0,23868	-0,01561	0,232846	0,029581	0,063931	-0,09788	-0,14143	0,042228	0,244725	0,084129	0,108984	0,194216	-1,07709	-0,52887	-0,39653	0,066636	0,214346
citrulline-nitric_oxide_cycle	-0,16151	-0,18874	-0,0133	-0,04983	-0,04315	-0,082	0,092833	-0,19395	0,179881	0,313329	-0,02688	-0,09878	-0,16286	-0,10133	-0,22785	1,033319	0,359586	1,396155	0,066836	0,214632
KEGG_Proteasome_Main_Pathway	1,77627	0,922599	0,658617	0,284535	0,317422	-1,43064	0,532361	0,753085	-1,73926	-3,13149	-0,12226	0,021837	0,882516	1,322374	1,903584	-19,1806	-15,4618	-13,211	0,067016	0,21487
reactome_Autodegradation_of_Cdh1_by_Cdh1_A	2,31402	0,817295	0,728042	-0,14654	-1,38967	-3,18341	0,210327	1,045725	-3,30972	-8,25168	-1,18961	-0,73043	0,631856	1,208523	2,021438	-27,1482	-22,7212	-18,7846	0,067108	0,21487
reactome_Ubiquitin_Mediated_Degradation_of_I	1,887268	0,77981	0,664337	0,486015	0,460324	-1,79377	0,826119	1,38112	-1,98912	-3,63638	-0,13494	-0,37011	0,894464	1,209453	1,606055	-22,9196	-18,4026	-15,3718	0,067186	0,21487
valine_degradation	0,257045	0,052825	0,113141	0,154985	0,723101	-0,03245	1,180032	1,584944	0,561322	0,283558	0,66937	-1,12039	-0,24431	0,118741	0,427724	-4,10403	-3,58939	-2,81677	0,067135	0,21487
KEGG_Vitamin_digestion_and_absorption_Main_	-0,31587	-0,37713	-0,06932	-0,75768	1,051608	0,129824	0,215967	-0,26228	0,762976	2,080326	0,933453	0,381207	-0,39188	0,41585	0,137352	3,067632	2,752488	1,63503	0,067485	0,215304
NCI_Trk_receptor_signaling_mediated_by_the_M	0,489026	-0,18542	-0,19561	-0,62805	0,113404	0,178312	-1,34364	-0,77123	-2,76619	-2,65649	0,962902	-2,01481	-1,06758	0,416039	-0,15422	-5,84231	-3,3649	-3,46286	0,067418	0,215304
reactome_p53_Dependent_G1_DNA_Damage_Re	0,25296	0,00605	0,00666	0,815478	1,401306	-0,30459	1,261426	1,325821	0,984402	0,140679	0,92485	0,135809	0,046771	0,298656	0,251165	-2,04422	-2,06848	-1,67691	0,067529	0,215304
D-imyoi-inositol-5-phosphate_metabolism	-0,0305	-0,1876	-0,09496	0,265412	0,720205	-0,2366	-0,24531	0,093605	-0,65527	-2,44913	-0,2799	-0,69006	0,665809	0,190867	0,96753	0,241074	0,080594	0,887786	0,068256	0,215399
IL-2_Pathway_Apoptosis	-0,01779	-0,04014	0,039423	0,114022	-0,2575	-0,35388	0,241156	0,066566	0,162079	0,314201	0,544888	0,086688	0,175726	-0,05255	0,108306	0,809531	0,448	-0,1141	0,06831	0,215399
KEGG_Apoptosis_Main_Pathway	0,067904	0,120341	0,101508	-0,95432	-0,55305	-1,9931	-5,13071	-4,94047	-4,72576	6,048309	2,230934	4,803765	1,293576	0,333489	-0,10385	-0,06886	0,818921	-0,57313	0,068183	0,215399
KEGG_Gastric_acid_secretion_Main_Pathway	-0,57303	-0,08104	-0,16377	-2,85563	-2,31839	-2,64106	-0,67539	-0,41107	-0,07688	1,608992	-0,57499	-0,03789	0,317432	1,638675	0,945624	4,246517	4,270637	3,522834	0,06814	0,215399
KEGG_Nucleotide_excision_repair_Main_Pathway	1,11947	-0,24128	-0,34018	-0,4117	1,498933	1,978623	1,298116	3,202186	-0,93447	-0,80817	0,155262	1,74046	-0,43548	0,527165	0,78903	-16,9843	-15,8438	-12,5337	0,068241	0,215399
NCI_PDGFR_beta_signaling_Pathway_(focal_adhe	0,099922	0,029599	0,043592	0,037354	0,069746	-0,34046	-0,25629	-0,23107	-0,36699	-0,98141	-0,30992	0,234613	-0,0488	0,238847	-0,06316	-1,28381	-0,97247	-0,8132	0,068006	0,215399
reactome_Activation_of_G_protein_gated_Potass	-0,20437	-0,26192	-0,37996	0,507273	0,656392	0,001307	-0,049	-0,21358	-0,13445	-1,31066	1,104416	0,66541	-0,12443	-0,14546	0,260389	-2,0758	-1,9248	-0,98977	0,068059	0,215399
reactome_degradation_of_DVL_Main_Pathway	1,663868	0,769387	0,669533	-0,6052	-0,09021	-2,864	0,154989	0,612364	-2,36527	-4,57359	-0,40897	-0,64837	1,401732	1,346834	2,158084	-22,7979	-18,1775	-15,5044	0,068319	0,215399
reactome_Inhibition_of_voltage_gated_Ca2_char	-0,20437	-0,26192	-0,37996	0,507273	0,656392	0,001307	-0,049	-0,21358	-0,13445	-1,31066	1,104416	0,66541	-0,12443	-0,14546	0,260389	-2,0758	-1,9248	-0,98977	0,068059	0,215399
reactome_Pregnenolone_biosynthesis_Main_Patl	0,075191	-0,04654	0,029012	0,261382	-0,78048	0,968928	0,099478	0,542162	0,279215	0,679441	-0,28435	-0,5017	-0,08089	0,048628	0,506802	-2,17404	-2,52087	-2,70746	0,06775	0,215399
reactome_Transport_of_nucleotide_sugars_Main	0,272682	-0,10759	0,026106	-0,45172	-1,11543	-0,26949	-0,80748	-0,61014	-1,27655	-0,86596	-1,14328	-1,71502	0,136937	0,434839	-0,22087	-2,35821	-1,35444	-1,55483	0,067922	0,215399
biocarta_signaling_pathway_from_g_protein_fam	0,068988	-0,4664	-0,44633	0,910937	-0,44709	-0,51957	-1,43527	-0,99421	-1,86404	-2,12022	-0,92659	-1,83176	-1,65739	-1,501	-0,31888	-5,93006	-5,28633	-3,03378	0,068501	0,215754
KEGG_HTLV_I_infection_Main_Pathway	1,926471	0,995383	0,631473	2,143177	1,018538	3,174528	1,795536	2,909653	-1,37395	-1,33312	2,345403	-0,84102	-0,06395	-0,60851	0,556687	-8,86738	-7,06077	-6,17962	0,068906	0,216811
Chemokine_Pathway_Gene_Expression_and_Apo	0,154548	-0,16997	-0,09933	0,429731	-0,46226	-0,69384	-0,5413	-0,17396	-0,8105	-1,31457	0,524145	-0,60848	0,401281	0,167688	0,417334	-1,3786	-1,52953	-0,84289	0,069544	0,216846
glycine_betaine_degradation	-0,15376	0,130128	-0,01944	-0,91954	-1,15455	-0,46339	-0,06407	-0,18509	0,088024	0,310137	0,035832	-0,01871	0,273418	0,165943	0,266659	1,430623	0,693026	0,553398	0,069196	0,216846
IGF1R_Signaling_Pathway_Cell_Survival	-0,036	0,009311	-0,0941	0,341122	0,280609	-0,43897	-1,34251	-1,40115	-2,04691	-0,10421	0,40107	-0,246	0,226763	0,159378	0,236494	-3,01021	-1,92129	-0,97185	0,069534	0,216846
NCI_IL27_mediated_signaling_events_Main_Path	0,006425	-0,32476	-0,01426	-0,93583	-0,43131	-1,27415	-0,58911	-0,53498	-0,68775	1,840059	1,130417	0,910197	-0,51649	-0,07275	0,192863	-0,76988	0,017483	-0,77181	0,069468	0,216846
PTEN_Pathway_Angiogenesis_and_Tumorigenesis	-2,61834	-0,71965	-0,31397	-8,70053	-6,03993	-5,17202	-1,91174	-3,48844	2,693364	-0,24131	-5,79072	-3,09129	2,431619	-0,36826	-0,36009	28,91484	26,82718	23,19761	0,069256	0,216846
PTEN_Pathway_Ca2+_Signaling	-2,61834	-0,71965	-0,31397	-8,70053	-6,03993	-5,17202	-1,91174	-3,48844	2,693364	-0,24131	-5,79072	-3,09129	2,431619	-0,36826	-0,36009	28,91484	26,82718	23,19761	0,069256	0,216846
reactome_G_protein_activation_Main_Pathway	-0,10991	-0,15746	-0,41734	2,144294	2,076146	1,15748	-0,0669	-0,32314	0,220296	-1,47086	1,354031	0,652581	-0,20593	-0,3756	-0,47271	-1,94605	-3,4582	-0,41178	0,069416	0,216846
VEGF_Pathway_Actin_Reorganization	-0,32453	-0,34327	-0,42787	0,259251	-0,48357	-0,0665	-1,36692	-1,29665	-1,25035	-0,95958	-0,27562	0,627215	0,632883	0,21509	0,805135	-0,2932	-0,12567	-0,1391	0,069366	0,216846
VEGF_Pathway_Cell_Migration	-0,32453	-0,34327	-0,42787	0,259251	-0,48357	-0,0665	-1,36692	-1,29665	-1,25035	-0,95958	-0,27562	0,627215	0,632883	0,21509	0,805135	-0,2932	-0,12567	-0,1391	0,069366	0,216846
NCI_Proteoglycan_syndecan_mediated_signaling_	-0,08333	-0,20903	-0,16736	-0,18398	-0,2745	-0,25883	-0,05106	-0,04386	-0,16452	-0,50004	-0,43564	-0,2314	-0,1589	-0,03417	0,016441	-0,38708	-0,38635	-0,16107	0,069651	0,216963
histidine_degradation	-0,01954	-0,09374	-0,01267	0,017268	0,248484	0,259027	-0,02822	-0,06733	0,197402	0,428685	0,411502	0,352656	0,003761	-0,15651	-0,15612	0,694461	0,646638	0,114981	0,06988	0,217331
reactome_APC_C_Cdc20_mediated_degradation_	2,327659	0,779413	0,717772	-0,70514	-1,16208	-3,99087	1,048784	1,831857	-2,74004	-7,84716	-1,1493	-1,59376	0,607016	1,305473	1,917762	-28,5624	-24,1619	-20,5485	0,069978	0,217331
zymosterol_biosynthesis	0,188121	0,111334	-0,07583	1,154265	0,603864	1,231654	0,292412	0,4004	0,050955	0,617436	0,0492	1,302808	-0,19903	0,183267	0,204231	-2,67589	-2,43903	-1,77442	0,069957	0,217331
NCI_p53_Pathway_(proteasomal_ubiquitin_deper	0,003291	-0,10156	-0,10872	0,037352	0,43306	0,04052	-0,13416	-0,07064	-0,26402	0,320497	0,025203	-0,21924	-0,18194	-0,04508	-0,01274	-1,264	-1,00089	-0,86155	0,070084	0,217443
reactome_SCF_Skp2_mediated_degradation_of_	2,237099	0,627308	0,700361	0,525919	0,935846	-2,77285	1,872491	2,620151	-1,00901	-5,33774	0,572209	-0,3605	0,724467	1,266536	1,596859	-25,6878	-21,4334	-17,0276	0,070583	0,218773
reactome_Signalling_to_RAS_Main_Pathway	-0,13016	-0,24268	-0,20198	-0,98339	-0,42849	-0,41337	-0,88994	-1,08973	-0,92699	-1,12879	-0,29372	-0,34122	0,654101	0,291742	0,527813	-2,21125	-1,46705	-0,88965	0,070738	0,219036
biocarta_apoptotic_signaling_in_response_to_dn	0,186959	0,041244	-0,03625	0,119104	0,208901	0,533358	-0,71358	-0,45801	-0,9994	0,830232	-0,49278	-0,78176	0,108946	-0,05315	-0,35749	-2,29958	-1,66702	-1,44624	0,071182	0,219795
NCI_E_cadherin_signaling_in_the_nascent_adher	0,186697	-0,04572	0,03202	-0,03977	-0,37553	-0,27003	-0,17832	-0,02799	-0,43186	-1,07153	-0,5434	-0,06798	-0,17504	0,205886	-0,12935	-1,59522	-1,37345	-1,02219	0,071283	0,219795
NCI_Glucocorticoid_receptor_regulatory_networl	-0,04722	-0,29009	-0,25777	-0,73566	0,091537	-0,07935	-1,05927	-0,82274	-1,52461	-0,75574	-0,67716	0,492482	-0,73264	0,327491	0,674839	-5,34612	-4,52461	-2,87322	0,071093	0,219795
NCI_Role_of_Calcineurin_dependent_NFAT_signa	-0,01372	-0,47546	-0,26213	-0,75529	-1,35993	-1,48695	-2,73989	-2,59885	-3,26606	-2,68828	-0,53206	-2,55117	0,765306	0,714719	1,589645	-3,77791	-2,19542	-0,66814	0,071276	0,219795
reactome_Retinoid_metabolism_and_transport_I	-0,84474	-0,15676	0,305868	-1,15617	0,564727	-1,05455	-2,36332	-3,38995	-1,37821	0,095594	0,560498	-0,49994	0,313282	0,089359	-0,62923	5,770847	5,600671	3,009264	0,071336	0,219795
NCI_mTOR_signaling_Pathway_(Pathway_rRNA_t	0,182389	0,022472	-0,02022	0,623156	0,860208	-0,64821	-0,36285	-0,28933	-0,98636	0,969532	0,539576	-0,3958	0,367343	0,526354	0,636297	-1,94744	-1,96984	-1,13813	0,071912	0,221351
reactome_Astrocytic_Glutamate_Glutamine_Upta	-0,07055	0,179871	0,025928	-0,52941	-0,25169	-0,3897	-1,25705	-1,21569	-1,60375	0,690068	0,628882	0,276449	-0,0731	0,016823	0,326073	-0,47094	0,032639	0,465091	0,072099	0,221707
superpathway_of_inositol_phosphate_compound	-0,55422	-0,71219	-0,779	-1,22802	-1,15979	-0,2803	-0,45877	0,33656	-1,85889	0,199987	-1,47735	0,484458	-0,32452	0,667186	0,888125	-6,69355	-5,52517	-4,13745	0,072684	0,223286
reactome_GRB2_events_in_EGFR_signaling_Mair	0,061213	-0,12489	-0,09101	-0,61156	-0,27751	-0,58518	0,506253	0,645646	0,242779	-1,38911	-0,15963	-0,91255	-0,17222	-0,05413	0,079091	-1,96924	-1,45208	-1,32531	0,072764	0,223309
NCI_IL5_mediated_signaling_events_Pathway_(pi	-0,0563	-0,21668	-0,03447	0,083006	0,206554	-0,23411	0,136316	0,198653	0,21121	0,247587	0,379609	-0,23992	-0,59654	-0,29931	-0,24773	0,167235	0,622446	0,194159	0,072911	0,223542
GSK3_Signaling_Pathway_b-CTNN_Degradation	1,262924	0,66358	0,648814	1,197572	-0,04402	-0,57426	-0,43301	-0,16523	-2,1063	-3,08372	1,216508	0,354747	0,450047	0,803731	1,020258	-14,5023	-11,9736	-10,1166	0,073252	0,223926
JAK-STAT_Pathway_JAK_Degradation	-1,26292	-0,66358	-0,64881	-1,19757	0,044024	0,574259	0,433011	0,165226	2,106302	3,083723	-1,21651	-0,35475	-0,45005	-0,80373	-1,02026	14,50233	11,97356	10,1166	0,073252	0,223926
reactome_RSK_activation_Main_Pathway	0,073044	0,042511	-0,09948	0,028082	-0,10115	-0,314	-1,196	-1,19484	-1,69286	-0,95526	0,681436	0,046008	0,228314	0,08706	0,417977	-2,30727	-2,14501	-1,92858	0,073176	0,223926
NCI_ALK1_signaling_events_Pathway_(endothelia	-0,01979	0,076168	-0,00932	0,010232	-0,00208	-0,03462	-0,59454	-0,56887	-0,83324	-1,46106	-0,30422	-0,34727	0,106846	0,023065	0,197818	-0,84859	-0,24872	0,279745	0,073393	0,224136
histamine_degradation	-0,012	-0,12271	0,021678	0,346269	0,020701	0,275136	-0,17499	-0,17588	-0,18676	0,097057	-0,46224	-0,11402	-0,02644	-0,20242	0,098791	-0,49026	-0,24176	0,123664	0,073957	0,225261
reactome_Autodegradation_of_the_E3_ubiquitin	1,828489	0,721673	0,610783	0,325523	0,331794	-1,88917	0,603417	1,10196	-2,12041	-3,4094	0,677478	0,302838	1,098065	1,395586	1,80175	-22,1374	-17,9903	-15,3144	0,07405	0,225261
reactome_Interconversion_of_polyamines_Main_	-0,00101	-0,08148	0,017604	-0,85121	-0,92247	-0,69002	-0,67793	-0,6502	-0,72317	-0,06908	0,233523	0,163133	0,128223	-0,08954	0,009974	-0,23806	-0,03172	0,290623	0,073962	0,225261
reactome_Pyrimidine_biosynthesis_Main_Pathwa	0,098477	0,041969	0,056947	0,232629	0,412977	0,178681	0,411525	0,485602	0,17778	-0,42795	0,53747	-0,05304	0,008133	-0,1585	0,096919	-1,66047	-0,85826	-0,8784	0,074002	0,225261
Estrogen_Pathway_Vasodilatation	0,090281	0,007924	0,015206	-0,35439	0,05132	0,242373	0,084225	-0,01032	0,083732	-0,18588	0,317239	0,109956	-0,03822	-0,01133	-0,13723	0,521848	0,4007	0,291144	0,074503	0,225976
KEGG_Morphine_addiction_Main_Pathway	-0,6354	-0,13251	-0,12691	-2,65258	-2,52943	-2,68636	-1,68362	-1,29065	-1,0907	1,228606	-1,85492	0,191112	-0,09653	1,194654	1,439564	4,73489	5,001265	4,120235	0,074496	0,225976
Ras_Pathway	-4,11879	-4,77347	-3,10831	-4,88884	-0,33346	-7,95321	-19,3715	-19,6999	-18,3648	-0,422	-5,48546	-7,87628	6,179047	6,131652	8,176454	-10,0838	-8,51264	-0,45549	0,074365	0,225976
NCI_Regulation_of_nuclear_SMAD2_3_signaling_	-0,2474	-0,16136	-0,47743	-0,77495	-0,74324	0,095398	-2,89816	-3,12424	-3,31608	-0,08129	-0,47982	-0,24895	-0,45313	-0,20851	-0,06737	2,489354	2,171623	1,3704	0,074746	0,226493
ErbB_Family_Pathway_Translation	0,033482	-0,05188	0,004434	0,080418	0,381772	-0,29725	0,099466	0,161997	-0,06601	0,127539	0,403956	-0,02032	0,2166	0,257591	0,404308	-0,90488	-0,44578	-0,54104	0,07532	0,227915
ERK_Signaling_Pathway	-10,3004	-3,88195	-1,83813	-26,6048	-11,4392	-21,7881	-26,0252	-31,9828	-12,0155	9,17472	-6,67821	-7,91583	4,975392	3,836504	5,207329	42,6101	35,71193	36,10656	0,075362	0,227915
allopregnanolone_biosynthesis	-0,01307	-0,10817	-0,03099	0,391989	0,297074	0,057789	0,0688	-0,00849	0,244664	0,203065	-0,22122	0,039617	0,183311	0,117624	0,314906	0,346632	0,316187	0,081912	0,075539	0,228023
NCI_Retinoic_acid_receptors_mediated_signaling	0,009276	-0,09158	-0,57254	-0,78037	0,194374	-0,54798	-2,03788	-1,38281	-2,66108	-1,29912	-0,31487	-0,10965	-0,61019	0,158424	0,167052	-4,26153	-2,27071	-2,61381	0,075544	0,228023
biocarta_double_stranded_rna_induced_gene_ex	0,146847	-0,02621	0,023614	0,06996	-0,24214	0,130087	-0,32321	-0,12537	-0,49194	-0,40853	-0,78054	-1,3553	-0,13782	-0,16378	0,014355	-0,3394	-0,55829	-0,17149	0,075821	0,228433
heme_biosynthesis_from_uroporphyrinogen-III_I	0,087043	-0,06176	0,02151	-0,28649	-0,31592	-0,01466	-0,19057	-0,1632	-0,34433	-0,30799	-1,04377	-0,83099	-0,1269	-0,22065	-0,25705	-0,69029	-0,85264	-0,99271	0,076046	0,228433
KEGG_Protein_digestion_and_absorption_Main_I	-1,9537	-0,72266	1,07642	-7,69536	-2,7634	-3,88901	-3,81382	-5,6622	0,459239	1,548735	-4,20964	-1,14212	1,064342	1,449439	0,910694	16,39823	12,59445	9,701853	0,075885	0,228433
NCI_Integrin_linked_kinase_signaling_Pathway_((	0,151351	0,008911	0,060832	0,676246	1,198672	0,63884	-1,55348	-1,66352	-1,95235	-0,67003	1,328955	0,937027	-0,07929	0,470753	-0,19941	-3,29136	-2,93308	-2,13328	0,076047	0,228433
NCI_TCR_signaling_in_naive_CD4_T_cells_Pathwa	-0,1748	-0,0129	0,155793	0,464299	-0,23226	0,254445	-0,08446	-0,42525	0,277788	1,665838	0,170809	0,181639	-0,10207	-0,28845	0,380247	1,336726	1,012796	0,840503	0,075984	0,228433
NCI_Nectin_adhesion_Pathway_(positive_regulati	0,089682	-0,08289	-0,0833	-0,2969	-0,43359	-0,12548	-0,22084	-0,13221	-0,39283	-1,52774	-0,54583	0,013443	-0,13741	0,151633	-0,03562	-1,49787	-0,95935	-0,747	0,076122	0,228439
biocarta_role_of_erk5_in_neuronal_survival_Mai	-0,17996	-0,49743	-0,41554	-0,63875	-0,14506	-0,15626	-2,79169	-2,78783	-3,26349	-1,3348	0,889759	0,097683	0,532949	-0,19434	0,572437	-4,56865	-2,15832	-2,18532	0,076204	0,228464
KEGG_Influenza_A_Main_Pathway	-0,434	0,477631	0,668226	2,586087	-1,36022	1,232958	1,931428	1,055944	3,155396	2,756306	0,462822	-3,04057	-2,20386	-1,99918	-3,00026	5,558758	5,253877	3,808126	0,076476	0,228732

protein_iOi-iNi-acetyl-glucosylation	-0,04264	-0,00943	-0,01213	-0,06382	-0,10961	-0,0827	-0,21874	-0,2315	-0,1356	0,164477	0,017504	0,123304	0,115947	0,113467	0,087017	0,559657	0,45875	0,326524	0,076482	0,228732
reactome_Folding_of_actin_by_CCT_TriC_Main_F	0,247347	0,04363	0,11163	-0,03596	-0,24846	-0,59442	-0,21603	0,055391	-0,59294	-1,00635	-0,02533	-0,24978	0,055775	0,201244	0,213077	-4,28375	-3,32507	-2,98697	0,076513	0,228732
biocarta_regulation_of_eif2_Main_Pathway	0,219854	0,206324	0,092769	-0,53229	-0,04407	-0,28199	0,393359	0,364189	-0,26471	-0,22737	-1,89212	-0,20491	-0,66142	0,332077	0,281959	-3,8613	-2,76751	-2,8702	0,077292	0,230616
Lipoxins_Influence_on_Neutrophil_Chemotaxis	0,057417	-0,23627	-0,1882	-0,13169	-0,36124	-0,57616	-0,048	0,044426	-0,51029	-1,52551	-0,45015	-0,11609	-0,28904	-0,15909	-0,20779	-2,48259	-1,81266	-1,47444	0,077289	0,230616
NCI_IL6_mediated_signaling_events_Main_Pathw	0,049771	-0,31845	-0,38739	-1,32163	-0,10526	-0,22224	0,478204	0,545585	-0,06448	-0,74228	-0,75337	-0,87397	0,212887	0,669991	0,789012	-1,6116	-1,11898	-0,64292	0,077643	0,231443
reactome_Adrenaline_noradrenaline_inhibits_ins	-0,21542	-0,19717	-0,40469	2,125292	2,00023	0,883294	-0,1357	-0,43374	-0,10883	-1,68803	1,238367	0,586381	-0,62505	-0,53212	-0,59801	-2,49565	-2,77102	-1,42111	0,07796	0,232167
IL-10_Pathway_Translational_Modulation	0,238738	-0,20108	-0,13268	-0,70324	-1,28077	-1,19813	-0,41374	-0,22342	-0,30148	0,796029	0,719586	0,416691	0,153158	-0,26194	-0,08812	-0,77578	-0,47294	-0,31749	0,078072	0,232278
NCI_S1P4_Main_Pathway	0,146635	-0,20093	-0,17484	0,599229	-0,28752	-0,07449	-0,31933	-0,17354	-0,38516	-0,50373	0,890097	-0,7331	-0,04852	-0,35626	0,076691	-2,98967	-3,23849	-2,588	0,078168	0,232342
reactome_CDK_mediated_phosphorylation_and_	1,856037	0,77427	0,70655	0,16798	0,60454	-2,38825	1,667938	2,121202	-1,08097	-3,17493	0,571798	0,300571	0,957878	1,502742	1,871675	-22,8293	-18,7868	-15,8599	0,078289	0,232478
reactome_SCF_beta_TrCP_mediated_degradatior	1,875388	0,70138	0,558559	-0,00848	0,113035	-3,31287	1,035939	1,548848	-1,68792	-4,26638	0,024451	-0,43764	0,719042	1,251116	1,741846	-22,1994	-18,3607	-15,1385	0,078571	0,232872
reactome_Vif_mediated_degradation_of_APOBE(	1,906543	0,836046	0,730805	-0,12787	0,372589	-2,12004	0,410703	0,960109	-2,4099	-3,78303	0,35258	-0,11754	0,796179	1,16189	1,549178	-23,1488	-18,8502	-15,9757	0,078554	0,232872
CDP-diacylglycerol_biosynthesis	0,000849	-0,26694	-0,32255	-0,81611	-0,87683	-1,81528	0,126945	0,95662	-0,79374	-1,20678	-0,8821	-1,56593	-0,18807	0,002131	0,986515	-4,65723	-3,72895	-3,19427	0,078691	0,233006
KEGG_Fanconi_anemia_Main_Pathway	0,482206	0,497431	0,42763	-0,46556	-0,75164	0,675683	-0,97171	-1,1957	-1,35133	-1,11428	-0,91432	0,396912	0,957426	1,325442	1,351774	-2,03712	-0,38615	-1,14415	0,078866	0,233083
reactome_Translocation_of_ZAP_70_to_Immuno	-0,86986	0,445125	0,130456	-0,31768	1,089813	2,025019	0,137075	-0,58777	1,729652	0,780649	0,963205	1,967407	-0,36212	-0,36168	-0,45223	4,695311	3,46817	4,271582	0,078866	0,233083
biocarta_sonic_hedgehog_receptor_ptc1_regulat	0,049736	-0,01727	-0,1107	-0,38733	0,255899	-0,13706	0,591611	0,796348	0,410082	-1,39056	-0,49593	-1,16419	-0,39057	-0,35857	-0,48555	-1,17096	-0,74427	-0,86392	0,079135	0,233248
KEGG_Adherens_junction_Main_Pathway	-0,1498	0,433183	0,393865	0,093615	-1,18327	-1,40771	3,331061	2,804931	3,804696	0,269146	-0,52487	0,033412	-1,01061	0,483233	0,576077	1,837319	1,274135	0,499706	0,079285	0,233248
KEGG_Legionellosis_Main_Pathway	-0,03115	-0,08749	0,021318	-0,91599	-0,07428	-0,47035	-1,79821	-1,92913	-1,75313	1,541216	0,444051	1,115102	-0,19724	-0,80294	-0,12203	-0,30634	-1,1615	-0,16278	0,079447	0,233248
KEGG_Porphyrin_and_chlorophyll_metabolism_N	0,08213	0,004826	0,019818	-0,23474	0,162772	0,604072	-0,32856	-0,30977	-0,80689	0,60394	0,525145	0,790881	0,850538	0,458363	0,701581	-1,69467	-1,9471	-3,04271	0,079286	0,233248
NCI_CXCR3_mediated_signaling_events_Pathway	-0,37897	0,033928	0,245171	0,386395	0,43604	0,123947	0,919018	1,036117	1,082888	1,467191	0,983628	-0,1969	-0,68606	-0,31925	-0,05247	1,387399	1,963282	0,043103	0,079394	0,233248
NCI_CXCR3_mediated_signaling_events_Pathway	-0,37897	0,033928	0,245171	0,386395	0,43604	0,123947	0,919018	1,036117	1,082888	1,467191	0,983628	-0,1969	-0,68606	-0,31925	-0,05247	1,387399	1,963282	0,043103	0,079394	0,233248
reactome_ER_Phagosome_Main_Pathway	2,217647	1,055795	0,860299	-0,08922	-0,51849	-2,15805	0,042692	0,684948	-2,8544	-3,24437	1,118977	-0,12538	1,852107	1,791657	2,870009	-29,4899	-24,2067	-19,9622	0,0794	0,233248
biocarta_akt_signaling_Pathway_(apoptosis)	-0,03718	-0,10918	-0,15991	0,241786	1,124777	0,897134	0,051193	-0,10285	-0,02161	-0,30303	0,367827	0,14429	0,14373	0,195925	0,176731	0,077084	0,324008	0,309884	0,079714	0,233373
biocarta_akt_signaling_Pathway_(cell_survival)	-0,03718	-0,10918	-0,15991	0,241786	1,124777	0,897134	0,051193	-0,10285	-0,02161	-0,30303	0,367827	0,14429	0,14373	0,195925	0,176731	0,077084	0,324008	0,309884	0,079714	0,233373
reactome_Interferon_gamma_signaling_Main_Pa	0,311676	0,00859	-0,08153	0,235842	-0,07291	0,22869	-0,48862	-0,17727	-0,87573	-0,12674	-0,22614	-1,39832	0,144574	0,073578	0,275624	-0,63656	0,004542	0,247394	0,079652	0,233373
biocarta_keratinocyte_differentiation_Pathway_(I	0,037014	-0,269	-0,22176	-0,71568	-0,85057	-0,23957	-1,84925	-1,78613	-2,0421	-0,78881	-1,17509	-0,81527	0,37637	0,287302	0,227862	-1,29285	-0,33296	-1,35635	0,079789	0,233374
biocarta_inactivation_of_gsk3_by_akt_causes_acc	-0,03961	-0,20861	-0,07077	0,543289	1,079534	1,272875	1,414714	1,605272	1,318765	-0,021	-0,8114	-1,16719	0,008928	-0,4249	-0,28848	-0,58816	0,541897	-0,37445	0,080121	0,234126
chondroitin_and_dermatan_biosynthesis	0,110793	-0,03316	-0,03798	0,187645	-0,12038	-0,28	-0,72614	-0,60399	-0,8606	-0,25623	-0,32866	-0,13991	0,105075	0,394237	0,357112	-0,47683	-0,19026	0,069605	0,080653	0,23507
reactome_Growth_hormone_receptor_signaling_	-0,40433	-0,25274	-0,16026	-0,41224	-1,08875	-1,214	-0,64169	-1,42608	-0,50862	1,789883	-0,55324	-0,04431	0,15881	0,290842	0,463288	1,913939	1,386839	1,289343	0,080652	0,23507
reactome_PIP3_activates_AKT_signaling_Main_Pa	0,103556	-0,03466	0,020454	-0,10511	0,071212	0,055606	0,84716	0,927547	0,473446	-0,68679	0,0861	0,099053	0,164253	0,77801	0,495485	-0,95871	0,181613	0,104803	0,080747	0,23507
reactome_Ubiquitin_dependent_degradation_of_	1,781515	0,757514	0,608059	-0,12437	0,029475	-2,02671	-0,19028	0,190847	-2,83086	-3,66624	0,439012	0,327877	0,901523	1,228509	1,557562	-22,3633	-18,3723	-15,394	0,080717	0,23507
reactome_Leukotriene_receptors_Main_Pathway	0,332281	0,223199	0,293588	-0,60308	-0,34087	0,129664	-0,33223	-0,38218	0,157783	-0,21125	-0,37995	-0,28445	-0,07494	0,173882	0,239003	1,457742	1,430015	1,266089	0,080885	0,235253
NCI_Canonical_NF_kappaB_Pathway_(proteasom	0,17802	-0,12027	-0,05362	-0,80203	-0,16604	-0,44286	-1,16873	-0,80935	-1,60944	-0,60246	-1,67778	-1,24082	0,05041	-0,42408	0,312749	-1,34205	-1,65584	-0,58895	0,081027	0,235447
NCI_Notch_mediated_HES_HEY_network_Pathwa	-0,15369	-0,12937	-0,07137	-1,5345	-2,08907	-1,82234	-0,1984	-0,30936	-0,03615	-0,42274	0,120662	1,015618	0,117583	0,34249	0,10403	0,450778	0,606668	0,255209	0,081213	0,235549
reactome_Regulation_of_ornithine_decarboxylas	1,801066	0,94777	0,682007	0,455921	-0,50101	-1,78083	-1,00299	-0,59902	-3,65213	-3,75468	0,196675	0,375357	0,782135	1,058245	1,46003	-21,6373	-17,6572	-14,976	0,081203	0,235549
KEGG_Basal_cell_carcinoma_Main_Pathway	-0,03078	-0,04338	-0,06253	0,811446	0,808647	0,755938	0,399068	0,352695	0,301738	0,013171	-0,61096	-0,22383	-0,26159	0,029879	-0,00647	-0,04252	0,160815	0,092391	0,081469	0,235632
reactome_Negative_regulators_of_RIG_I_MDA5_	0,388065	-0,29433	-0,06302	-0,36236	0,571692	0,184835	-0,61982	-0,29014	-1,08615	-0,14272	-0,13937	0,429403	0,216411	-0,29908	0,112657	-4,31409	-3,89698	-2,98494	0,081336	0,235632
reactome_Prefoldin_mediated_transfer_of_subst	0,443501	-0,37277	0,082034	-0,48995	-1,08888	-1,12376	-1,94218	-1,68558	-2,56561	-2,94478	-0,83287	-0,66431	-0,03064	-0,58049	-0,19653	-10,7077	-8,32592	-7,41094	0,081413	0,235632
NCI_CXCR4_mediated_signaling_events_Pathway	-0,181	-0,19501	-0,12547	0,039408	-0,51865	-0,13364	-0,30302	-0,34059	-0,46042	-0,33555	-0,71492	0,015045	-0,28114	0,184978	0,152547	-2,86043	-1,11758	-0,83539	0,082076	0,236003
reactome_ATF6_alpha_activates_chaperone_gen	0,011894	0,010855	-0,02001	0,137164	0,010465	0,004147	-0,09005	-0,05068	-0,12432	-0,16083	0,239045	0,112823	0,061484	0,04771	0,054769	-0,81127	-0,58319	-0,43165	0,08202	0,236003
reactome_Crosslinking_of_collagen_fibrils_Main_	-0,08674	0,035372	0,051582	-0,68078	0,419309	-0,93184	-1,17332	-1,25265	-0,68032	-0,74431	0,310538	0,69523	-0,14906	-0,17104	-0,17434	1,38692	-0,20605	0,950937	0,081984	0,236003
reactome_degradation_of_AXIN_Main_Pathway	1,725628	0,694908	0,538494	-0,6189	-0,12647	-2,35917	0,436152	0,922641	-2,42384	-4,30916	0,941196	0,158384	1,04373	1,509469	1,768581	-21,8816	-17,912	-14,709	0,08201	0,236003
reactome_Formyl_peptide_receptors_bind_form	-0,22443	-0,10806	-0,04369	0,214528	-1,28622	-0,27339	0,136815	-0,1118	0,258132	0,878724	0,252334	-0,54957	-0,58901	-0,21317	-0,68069	-0,37796	0,44345	-0,07406	0,082127	0,236003
reactome_Regulation_of_activated_PAK_2p34_by	1,816635	0,787445	0,651568	-0,48883	-0,03033	-2,30411	0,350311	0,873515	-2,32997	-3,76377	0,577016	0,225945	1,017059	1,40099	1,844937	-22,8725	-19,1271	-16,1619	0,081959	0,236003
reactome_Release_of_Hh_Np_from_the_secretin	-0,35497	-0,36311	-0,24262	-0,82322	0,767721	0,092087	-0,86509	-0,98835	-0,42801	1,695445	0,144713	1,332134	0,057725	-0,37392	0,008152	1,530293	0,839389	0,985994	0,082101	0,236003
biocarta_regulation_of_bad_phosphorylation_Pat	-0,00943	-0,06995	-0,10325	0,286793	0,459008	0,565348	-0,34482	-0,27488	-0,34555	-0,26052	0,457379	-0,29287	-0,10944	0,151502	-0,01577	-0,00952	0,108607	-0,10069	0,082306	0,236297
IL-2_Pathway_Protein_Synthesis	0,003082	0,416361	0,24814	-0,74681	0,556523	-0,82003	0,716835	0,413836	0,794519	0,515583	0,462288	0,737597	0,126019	-0,1209	0,093253	0,281085	0,188726	0,089268	0,082876	0,237715
glycogenolysis	-0,07469	-0,19991	0,012716	-0,09321	0,09491	-0,67682	0,207868	0,30415	-0,03856	-1,93262	0,806659	-0,4198	0,030255	0,047933	0,010702	-1,74839	-1,8494	-1,82906	0,082961	0,237741
reactome_Oxygen_dependent_asparagine_hydro	0,064339	-0,01768	-0,15816	0,124398	-0,27619	-0,67072	-0,18567	0,006812	-0,39991	-0,02276	-0,48053	-0,48482	0,129068	-0,08972	0,309706	-2,49273	-1,41227	-1,1522	0,083203	0,238214
NCI_Regulation_of_Ras_family_activation_Main_I	-0,10785	0,007065	0,24636	-3,14589	-1,28676	-1,36411	-0,31326	-0,63249	-0,45423	-1,33815	-0,16095	-0,58586	-0,36618	0,301325	-0,53545	0,643024	0,740692	0,662023	0,083323	0,238338
Chemotaxis_Driven_by_C5a	-0,07226	-0,60085	-0,51853	3,309955	2,699905	1,876278	0,005245	0,173542	-0,54521	-1,62377	2,187381	2,618532	-1,10856	-0,87931	-0,04389	-6,48095	-5,71944	-2,99501	0,083905	0,239664
KEGG_Oxytocin_signaling_Main_Pathway	-1,0881	-0,0362	-0,04754	-1,23986	-0,90362	-3,49431	-1,45098	-1,46546	-0,19693	3,017804	-2,03688	0,523891	0,448729	1,715183	1,672436	9,826643	7,856336	9,216609	0,084325	0,239664
mTOR_Pathway_VEGF_Pathway	0,049993	-0,00773	-0,03349	-0,26111	-0,22113	-0,47397	-0,23461	-0,19395	-0,44843	-0,20321	0,115373	-0,14457	0,272959	0,445941	0,434653	-1,95761	-1,77078	-1,20037	0,084026	0,239664
NCI_FOXA2_and_FOXA3_transcription_factor_ne	0,076684	0,101519	-0,03173	-0,1818	0,359035	0,220229	-0,06762	-0,16215	0,059221	0,094071	-0,3031	-0,26652	0,081814	0,0561	0,174248	-0,09208	0,017096	-0,09168	0,084179	0,239664
NCI_p38_signaling_mediated_by_MAPKAP_kinase	-0,10502	-0,34688	-0,35107	-0,48403	0,083081	-0,64862	-1,68828	-1,52768	-1,58232	-0,40178	0,383945	-0,63512	0,840936	0,362993	0,350334	-2,26424	-0,71845	-0,41489	0,084254	0,239664
reactome_Degradation_of_the_extracellular_mat	-0,37007	-0,16358	-0,02703	-0,25791	-1,06936	-0,20407	-2,64913	-3,17425	-1,68194	4,253483	1,571536	2,670241	1,394605	0,366358	1,479362	2,233798	2,413425	1,055114	0,084302	0,239664
reactome_MicroRNA_miRNA_biogenesis_Main_P	0,195306	-0,06983	-0,05501	-1,64627	-0,87193	-1,22545	0,369225	0,600795	-0,21515	-2,45959	-1,18567	-1,71934	0,855036	0,884301	0,875926	-3,08791	-2,30354	-1,68966	0,08407	0,239664
ATM_Pathway	0,301022	-0,52983	-0,60015	3,091113	2,978566	1,981373	-0,77665	0,162865	-1,83674	1,193389	1,259922	0,969679	-0,05727	-0,30965	0,564131	-2,95349	-0,1858	-0,0898	0,085034	0,241458
FLT3_Signaling_Pathway	-0,21982	-1,29766	-0,97159	-0,10682	-0,77011	-1,49406	-3,34215	-2,13179	-4,81653	-2,05602	2,538282	2,168187	0,851641	0,915271	2,317535	-11,3918	-8,27308	-7,84866	0,085309	0,241579
reactome_Calnexin_calreticulin_cycle_Main_Path	0,066637	-0,00516	-0,02146	0,241525	0,178255	0,149482	-0,06915	-0,00981	-0,18502	-0,29739	0,082026	0,422452	-0,06915	-0,13049	-0,02626	-2,20771	-1,8711	-1,49078	0,08524	0,241579
reactome_S6K1_mediated_signalling_Main_Path\	0,072709	-0,04385	0,039261	0,317242	0,128058	-0,05767	0,254676	0,331268	0,056672	0,029256	0,590119	0,205338	0,154729	0,260169	0,440841	-0,92275	-0,63649	-0,307	0,085172	0,241579
reactome_Signaling_by_constitutively_active_EGI	0,036076	-0,21634	-0,28064	-0,50008	-0,20122	-0,59702	-0,05177	0,064832	-0,58871	-1,2603	0,481573	-0,76915	-0,14156	-0,21269	0,205138	-5,13434	-3,23461	-2,9508	0,085601	0,242186
reactome_Signaling_by_FGFR_Main_Pathway	0,037607	0,108665	0,083328	-0,17821	0,258182	0,377837	0,30311	0,227037	0,384931	0,360061	-0,37315	-0,66429	0,22994	0,27469	0,086077	-0,03622	0,09737	-0,21057	0,085754	0,2424
Chemokine_Pathway	-5,01034	-1,11108	-0,12027	-4,08488	-0,39398	-1,942	-1,98505	-4,60819	2,58754	4,632215	-0,83189	-5,42769	-2,80402	-0,63068	0,441951	17,18697	20,50731	15,97358	0,085933	0,242465
reactome_RAF_activation_Main_Pathway	-0,03099	-0,04651	-0,09579	-0,38183	0,035996	-0,23876	-0,3975	-0,31893	-0,56949	-0,62127	0,098185	-0,35294	-0,16907	-0,08587	-0,09684	-1,88824	-1,55799	-1,53709	0,085901	0,242465
KEGG_Terpenoid_backbone_biosynthesis_Main_I	0,320754	0,283112	0,216644	1,097322	0,834492	1,732833	2,146695	2,572936	1,257671	-0,561	0,64426	1,145975	-0,31989	-0,10336	-0,19142	-7,02699	-3,6189	-3,73695	0,086207	0,242499
MAPK_Signaling_Pathway_Cell_Motility_Inflamm	0,443282	-0,10128	-0,05284	0,600949	0,898674	0,277677	-0,64549	-0,20903	-1,00324	0,737102	1,307909	-0,29857	0,357601	0,170942	0,108619	-2,41367	-1,93814	-1,95119	0,086185	0,242499
NCI_Cellular_roles_of_Anthrax_toxin_Pathway_(N	-0,12779	-0,05505	0,028974	-0,08869	-0,12929	0,02598	-0,69636	-0,69892	-0,41072	1,094787	-0,58198	-0,94395	0,15044	0,222942	-0,06909	-1,74741	-1,26918	-1,88653	0,086283	0,242499
tetrahydrofolate_salvage_from_510-methenyltet	0,15759	0,089814	0,000984	0,24077	0,18036	0,102971	0,072803	0,149064	-0,12195	-0,41562	0,239364	0,155051	-0,09162	-0,09988	0,118144	-0,89956	-0,60133	-0,77518	0,086216	0,242499
TRAF_Pathway_Gene_Expression_via_FOS_JUN	0,120409	-0,22367	-0,30881	0,472878	0,645849	0,265931	0,688936	0,983186	0,317796	-0,68313	-0,24222	-0,32136	0,712787	0,361743	0,575686	0,663166	0,997523	0,889821	0,086334	0,242499
reactome_Hh_ligand_biogenesis_disease_Main_F	1,834395	0,689902	0,561344	-0,54133	0,334865	-2,88989	0,903986	1,34615	-1,84733	-3,83633	0,434038	0,329136	0,938584	1,030063	1,715529	-23,4158	-19,0404	-16,2228	0,086602	0,242812
reactome_SLBP_independent_Processing_of_Hist	0,59046	0,206315	0,00227	0,327078	0,476781	0,161544	0,10681	0,438752	-0,52717	-0,23132	-0,45934	0,276446	0,064383	-0,19899	-0,19704	-2,92346	-3,07877	-1,80913	0,086542	0,242812
KEGG_cAMP_signaling_Main_Pathway	-1,7422	0,033911	0,214333	-0,99811	0,65825	-2,70687	-0,23681	0,438152	-0,88259	3,853473	-1,64297	6,521342	2,794511	2,111208	2,322201	11,71226	11,73096	14,9019	0,087057	0,243461
MAPK_Signaling_Pathway_Gene_Expression_Apo	0,037501	-0,11013	-0,14304	-0,81094	0,291877	-0,18732	-0,45833	-0,02745	-0,70164	-0,31628	0,687621	-0,76308	0,446948	-0,04198	0,548305	-2,50907	-1,64063	-1,39759	0,08713	0,243461
reactome_Dual_incision_reaction_in_TC_NER_Ma	0,700794	-0,11602	-0,41208	-0,16393	0,108348	0,079768	-1,76792	-0,52666	-3,0997	-1,67484	-0,26318	1,70728	-0,08594	0,235162	-0,01056	-9,97227	-8,61075	-6,82121	0,087146	0,243461
reactome_Formation_of_transcription_coupled_I	0,700794	-0,11602	-0,41208	-0,16393	0,108348	0,079768	-1,76792	-0,52666	-3,0997	-1,67484	-0,26318	1,70728	-0,08594	0,235162	-0,01056	-9,97227	-8,61075	-6,82121	0,087146	0,243461
cAMP_Pathway_Myocardial_Contraction	-2,16875	-0,4852	0,156591	-2,54827	-1,38123	-2,68837	-6,36842	-8,02342	-4,04995	-1,43097	0,682142	-3,66218	-1,33661	-0,55615	1,055342	15,00211	11,15327	10,62226	0,087511	0,244264
Estrogen_Pathway	-3,27573	-3,57932	-3,07096	-6,06582	-3,08851	-7,03722	-18,2038	-18,3155	-17,8708	1,871377	-5,80869	-4,16027	4,114534	2,941739	6,042763	-15,7163	-14,5555	-6,28137	0,087627	0,244368
NCI_Beta2_integrin_cell_surface_interactions_Ma	-0,65158	-0,20633	-0,22584	0,271849	-0,52467	0,249633	-3,76464	-4,36552	-1,20114	1,166481	0,772721	0,269645	0,897762	-0,35122	0,249407	6,501183	4,414329	4,569433	0,088145	0,245592
reactome_Recycling_of_eIF2_GDP_Main_Pathwa	0,37898	0,205585	0,091712	0,025091	0,127443	0,153393	0,027216	0,286716	-0,34738	0,183041	-0,59893	-0,65827	-0,29128	-0,31059	-0,04966	-3,92683	-3,22918	-2,95902	0,08824	0,245638
biocarta_protein_kinase_a_at_the_centrosome_I	-0,11091	-0,2032	-0,30331	0,013502	-0,0645	-0,29355	-0,44006	-0,30822	-0,89559	-0,57756	0,250669	-0,66756	-0,60138	-0,48509	-0,08819	-4,22709	-3,4615	-2,7622	0,088642	0,245781
biocarta_role_of_nicotinic_acetylcholine_recepto	-0,03634	0,060886	-0,09341	-0,60532	0,107733	-0,95837	-0,5162	-0,74121	-0,3476	0,325816	1,104317	0,216109	0,120052	0,183668	0,362108	0,23236	0,476897	0,886031	0,088597	0,245781
KEGG_RIG_I_like_receptor_signaling_Main_Pathv	0,675468	-0,37286	-0,15904	-0,67946	-0,04142	0,04906	-3,18529	-2,22536	-3,6319	0,635144	-0,96715	-0,09533	0,257617	-0,14878	0,402043	-5,50593	-5,4519	-3,94025	0,088403	0,245781
NCI_ErbB1_downstream_signaling_Pathway_(ruf	-0,01104	-0,17311	-0,23905	-0,29476	-0,98205	-1,24435	-1,3205	-1,19733	-1,88511	-1,90599	-0,09233	-0,90477	-0,11792	-0,23967	0,30246	-6,92429	-4,96643	-3,57231	0,088686	0,245781
reactome_Activation_of_gene_expression_by_SR	0,548426	-0,25295	-0,1653	-0,57177	-1,44836	0,902722	2,522068	3,149408	1,107115	-1,43336	0,689211	-0,99138	-0,08277	-0,05716	0,778538	-9,64647	-6,13264	-4,55851	0,088663	0,245781
biocarta_nuclear_receptors_coordinate_the_activ	-0,03879	-0,08939	-0,16076	-0,62089	-0,8366	-0,24524	-0,63531	-0,53197	-0,86351	-0,68183	-0,25012	-0,6017	0,345529	0,227064	0,415734	-2,25895	-1,04766	-1,33499	0,089019	0,245849
KEGG_Leishmaniasis_Main_Pathway	0,552298	-0,54011	-0,08233	-0,81738	0,647342	1,18324	-3,9178	-3,64739	-3,81462	-0,02785	-1,4327	-0,07687	0,287339	-1,11652	0,91034	-1,43491	-2,42221	-1,25563	0,088929	0,245849
NCI_LKB1_signaling_events_Pathway_(ATP_biosy	0,011165	-0,06708	-0,02383	-0,10642	0,125025	-0,41503	0,063348	0,099779	-0,04527	-0,49691	0,097978	0,342203	0,10256	0,404241	0,298525	-0,99969	-0,90329	-0,63176	0,089263	0,245849
NCI_LKB1_signaling_events_Pathway_(ATP_meta	0,011165	-0,06708	-0,02383	-0,10642	0,125025	-0,41503	0,063348	0,099779	-0,04527	-0,49691	0,097978	0,342203	0,10256	0,404241	0,298525	-0,99969	-0,90329	-0,63176	0,089263	0,245849
NCI_LKB1_signaling_events_Pathway_(Wnt_recep	0,011165	-0,06708	-0,02383	-0,10642	0,125025	-0,41503	0,063348	0,099779	-0,04527	-0,49691	0,097978	0,342203	0,10256	0,404241	0,298525	-0,99969	-0,90329	-0,63176	0,089263	0,245849
pentose_phosphate_pathway_oxidative_branch	-0,0438	-0,12218	-0,06519	0,3801	-0,29094	-0,62079	-0,26242	-0,27931	-0,36224	0,04203	-0,07661	-0,33216	0,225909	0,105465	0,327619	-0,74479	-0,95114	-1,29349	0,088903	0,245849
reactome_Vpu_mediated_degradation_of_CD4_I	1,88007	0,778451	0,702484	-0,29925	-0,18231	-2,87834	0,521974	1,043055	-2,14222	-3,59379	0,540406	-0,05709	0,808216	1,242827	1,885146	-22,1265	-18,3403	-14,9813	0,089073	0,245849
NCI_Alpha_synuclein_signaling_Pathway_(Pathwa	0,039581	-0,03532	0,137248	0,235918	0,311121	-0,13081	-1,1386	-1,10291	-1,0912	-0,21792	0,346939	0,639754	-0,16275	-0,10129	-0,14078	1,145468	0,296075	0,315442	0,090001	0,245924
NCI_Alpha_synuclein_signaling_Pathway_(Pathwa	0,039581	-0,03532	0,137248	0,235918	0,311121	-0,13081	-1,1386	-1,10291	-1,0912	-0,21792	0,346939	0,639754	-0,16275	-0,10129	-0,14078	1,145468	0,296075	0,315442	0,090001	0,245924
NCI_Signaling_events_mediated_by_focal_adhesi	0,291439	-0,22254	-0,33002	-1,83854	-0,02142	-2,46871	-4,13689	-3,26101	-5,4844	-2,32907	0,129482	-0,11462	1,955057	1,704806	2,589533	-8,14625	-4,89205	-2,81727	0,089612	0,245924
NGF_Pathway_Neuronal_Survival	-0,48897	-0,34086	-0,29636	0,401667	0,891914	0,74718	0,60346	1,044899	0,291105	1,442318	0,352746	1,343152	0,066929	0,034348	0,456969	-4,15876	-2,82987	-2,30817	0,090001	0,245924
reactome_Degradation_of_beta_catenin_by_the_	2,283596	0,668413	0,644542	0,417216	-0,51486	-3,29057	-0,04593	0,754731	-3,33166	-3,28258	0,964686	-0,41912	1,061552	1,469859	1,889272	-26,0493	-21,4659	-17,3458	0,089569	0,245924
reactome_IRAK1_recruits_IKK_complex_Main_Pa	0,105255	-0,13445	-0,05676	0,108377	-0,56111	0,26156	-0,50491	-0,31212	-0,80769	-0,58223	-0,89856	-1,03202	0,466055	0,168478	0,468959	0,129825	-0,21728	0,36674	0,089911	0,245924
reactome_IRAK1_recruits_IKK_complex_upon_TL	0,105255	-0,13445	-0,05676	0,108377	-0,56111	0,26156	-0,50491	-0,31212	-0,80769	-0,58223	-0,89856	-1,03202	0,466055	0,168478	0,468959	0,129825	-0,21728	0,36674	0,089911	0,245924
reactome_Purine_catabolism_Main_Pathway	0,084165	0,32011	-0,1006	1,093149	0,388193	0,372485	-0,13784	-0,07284	-0,0402	-0,01413	-0,90128	0,336658	0,009822	-0,56854	0,044969	-0,87398	-0,99295	-0,68825	0,089931	0,245924
reactome_Vitamins_Main_Pathway	-0,081	-0,03074	-0,10228	-0,94179	0,14142	0,418997	0,356186	-0,06546	0,290586	0,891016	0,552944	0,535827	0,323645	-0,1415	-0,10286	1,169256	0,307608	0,506347	0,089628	0,245924
NCI_Signaling_events_mediated_by_VEGFR1_and	-0,10258	-0,2309	-0,15981	-0,3581	-0,45497	-1,70225	-1,47606	-1,30803	-1,59286	-0,02931	0,824702	0,351581	-0,15772	-0,56727	-0,04526	-0,62922	-0,24888	0,132396	0,090432	0,246454
PAK_Pathway_Lamelliopodia_and_Filopodia_Out	-6,36884	-1,87237	-0,91737	-10,6611	-3,69352	-10,6839	-8,77404	-12,9273	-1,18557	5,27824	-9,22688	-2,74083	0,237679	-0,10087	1,37655	20,65218	14,78112	13,48459	0,090282	0,246454
Target_drugs_pathway	-0,73225	-1,1257	-1,30393	-4,71055	-1,28546	-7,63317	-9,17628	-11,2101	-10,1876	-5,31212	-6,83095	-1,37397	1,529617	-0,07807	2,213632	-17,0231	-13,326	-9,39576	0,090354	0,246454
NCI_N_cadherin_signaling_events_Pathway_(gap	-0,04993	-0,12786	-0,01887	0,255838	0,504862	0,214975	0,477311	0,401832	0,164702	-0,15504	0,939482	0,155043	-0,11476	-0,19179	0,158569	-3,48687	-2,5645	-2,37265	0,090758	0,246909
reactome_Synthesis_secretion_and_deacylation_	0,66839	-0,03177	-0,04825	-0,63973	1,123727	0,712132	-2,85332	-2,37823	-3,27795	-0,30276	0,014982	0,789283	-0,27214	0,561846	0,02385	-4,0589	-3,66059	-2,46623	0,090707	0,246909
NCI_p53_Pathway_(apoptosis)	0,556625	-0,21107	-0,28624	1,257214	1,420154	0,389832	-0,76289	0,098695	-2,25799	-0,38301	1,69391	0,518369	-0,75375	-0,18402	0,016337	-9,35947	-6,41235	-6,1478	0,090838	0,246913
NCI_Ephrin_A_reverse_signaling_Main_Pathway	-0,07909	0,012739	-0,00722	-0,90928	-1,52127	-1,33145	-0,92673	-0,99716	-1,07472	0,543524	0,118009	0,926123	0,217022	0,437096	0,431902	0,020772	0,998924	0,531973	0,091702	0,248394
NCI_Ephrin_A_reverse_signaling_Pathway_(beta_	-0,07909	0,012739	-0,00722	-0,90928	-1,52127	-1,33145	-0,92673	-0,99716	-1,07472	0,543524	0,118009	0,926123	0,217022	0,437096	0,431902	0,020772	0,998924	0,531973	0,091702	0,248394
reactome_disassembly_of_the_destruction_comp	-0,14925	-0,71816	-0,34291	0,549795	-0,49826	-0,49355	0,107838	0,328918	0,055315	-0,35177	1,116664	0,406968	0,830237	0,335571	0,425163	-2,58846	-1,46388	-1,20626	0,091525	0,248394
reactome_SLBP_Dependent_Processing_of_Repli	0,595288	0,182462	-0,00914	0,298038	0,464892	0,191636	0,242905	0,551192	-0,40887	-0,12604	-0,43157	0,286194	0,080711	-0,20026	-0,26707	-3,45704	-3,59295	-2,20491	0,09166	0,248394
reactome_Resolution_of_AP_sites_via_the_single	-0,08326	-0,10044	-0,10044	-0,46235	-0,24783	-0,35411	0,251291	0,290618	0,088184	-1,00279	-0,21103	-0,53014	0,050922	0,188136	0,32239	-0,53221	-0,49064	-0,43274	0,091808	0,248464
cAMP_Pathway_Degradation_of_Cell_Cycle_Regu	-0,56283	-0,24097	-0,14489	0,156149	1,698602	1,18519	-0,95884	-1,1209	-0,16716	0,926057	0,679504	1,101446	-1,11002	-0,78829	-0,9247	4,086037	3,547623	3,667398	0,091958	0,248655
biocarta_role_of_brca1_brca2_and_atr_in_cance	0,362883	-0,07883	-0,10598	0,909184	1,007714	0,693886	0,636387	1,438212	-0,56965	0,815006	0,466118	-0,1413	-0,30835	-0,02331	-0,16373	-5,26807	-2,46511	-2,18934	0,092235	0,249187
NCI_C_MYB_transcription_factor_network_Main_	-0,47355	-0,57451	-0,53488	-1,35522	-0,23947	-1,16116	-2,04599	-2,01518	-2,75114	0,129541	0,601263	-0,66083	0,631782	1,109733	0,988264	-4,33811	-3,8479	-2,31329	0,092533	0,249774
biocarta_role_of_egf_receptor_transactivation_b	-0,19385	-0,43184	-0,39591	0,06088	-0,1902	-0,80792	-0,2923	-0,03063	-0,63553	-1,18687	-0,03088	0,161734	-0,02362	-0,29797	0,180769	-1,52548	-1,04526	-0,86079	0,092972	0,249876
NCI_TNF_receptor_signaling_pathway__Pathway	0,068314	-0,028	-0,15954	-0,61773	-0,08766	-1,67885	-2,13356	-1,80783	-2,27798	-1,8923	-0,62639	-1,73428	0,62802	0,443881	0,491533	-2,21853	-1,41655	-0,90111	0,092769	0,249876
reactome_Scavenging_by_Class_B_Receptors_Ma	-0,09737	-0,01622	0,144689	0,209126	0,2727	0,253292	0,22843	0,105699	0,482878	0,638405	0,510321	0,098372	-0,21768	-0,08919	-0,41891	0,680884	0,388145	0,169945	0,092873	0,249876
reactome_Signaling_by_FGFR1_fusion_mutants_I	0,08847	-0,07079	-0,17974	-0,55473	0,115909	-0,38135	-0,11253	0,30077	-0,68395	-0,68892	-0,24035	0,480716	0,185121	0,048858	0,265486	-3,35105	-2,34467	-1,79148	0,092966	0,249876
UTP_and_CTP_dephosphorylation_II	0,057171	-0,05204	0,008941	-0,40533	-0,4578	-0,40395	0,985983	1,105974	0,824656	-0,83856	-1,39256	-0,11665	0,268845	0,648157	0,550347	-0,90916	-0,36535	-0,55045	0,092824	0,249876
Cytokine_Network_Pathway	1,200634	1,110728	1,505894	-1,83924	1,167263	-3,19184	0,861777	-0,43923	2,473116	2,579422	1,670353	3,278213	1,512841	2,415727	2,415074	5,149672	4,191444	1,63317	0,093582	0,250437
NCI_CDC42_signaling_events_Main_Pathway	-0,07266	-0,2501	-0,65241	-1,32951	-1,4675	-1,76963	-2,80824	-1,92771	-4,62545	-1,78453	0,356846	0,304154	0,51966	0,991943	1,817331	-13,2255	-9,33571	-7,12086	0,09354	0,250437
NCI_Fanconi_anemia_Pathway_(G1_S_transition_	0,08103	-0,06303	-0,05072	0,319203	0,533876	0,470666	-0,1071	0,140621	-0,44573	-0,50765	0,463648	0,255109	-0,28188	-0,13012	-0,16791	-1,7341	-1,14939	-0,77292	0,093299	0,250437
reactome_Conjugation_of_salicylate_with_glycine	-0,13522	0,0725	0,067356	-0,33724	0,442314	0,53277	0,834569	0,972252	0,97696	1,473226	0,165938	0,433134	0,263256	-0,18536	-0,32265	1,874462	0,985781	0,28655	0,093441	0,250437
TNF_Signaling_Pathway	0,255449	-0,42069	-0,36541	-0,37504	-0,82885	-1,95845	-4,13428	-2,91055	-5,68567	0,551314	-2,41733	-1,45543	-0,0032	0,478311	-0,32755	-8,0118	-6,07948	-4,48719	0,093368	0,250437
reactome_Synthesis_of_PIPs_at_the_late_endoso	0,225106	-0,00364	-0,08398	-0,25085	-0,69579	-0,26927	-0,31624	0,005313	-0,62656	-3,11667	-1,58069	-0,53796	0,163384	0,720397	0,359469	-1,16926	-0,29811	-0,1207	0,093801	0,250806
KEGG_Viral_carcinogenesis_Main_Pathway	1,289553	-0,65916	-1,20437	-4,56745	-3,67582	-5,759	0,29636	2,384663	-3,29989	-2,01863	-4,08268	-6,38934	0,088298	-0,89064	3,521927	-32,9865	-25,2826	-18,9192	0,093996	0,251114
KEGG_TGF_beta_signaling_Main_Pathway	0,427831	-0,26468	-0,48648	3,30158	2,652977	1,756966	2,28521	2,690491	-0,17065	1,735556	2,343054	2,457586	0,44984	-0,05071	-0,30671	-4,78038	-5,28745	-3,57167	0,094698	0,252341
NCI_LPA_receptor_mediated_events_Pathway_(p	-0,01217	-0,08563	-0,01472	0,127948	-0,01568	-0,05222	0,09574	0,241798	0,054072	-0,21187	0,185783	-0,30484	0,170284	0,180061	0,13414	0,081077	0,014293	0,101936	0,094575	0,252341
reactome_Downregulation_of_ERBB4_signaling_I	0,205415	0,0443	0,020393	-0,33018	0,328868	-0,17471	0,208449	0,378966	-0,12941	-0,40544	0,176507	0,032367	0,10826	0,072442	0,094417	-3,80951	-2,68919	-2,11078	0,094699	0,252341
Interferon_Pathway_Gene_Expression_via_HIST1	0,514799	-0,45737	-0,1544	0,852574	1,220849	0,440724	2,392738	2,932912	1,594128	1,539519	1,207515	-0,93237	0,413551	-0,80211	1,111385	-2,29169	-1,67691	-1,5926	0,094924	0,25251

Ras_Signaling	-0,2669	-0,47794	-0,16685	-2,80481	0,118656	-2,23521	-5,89125	-6,10332	-5,96512	1,846873	-1,36562	0,500894	0,05959	-0,10609	-0,15652	0,094425	1,258683	1,60999	0,094859	0,25251
KEGG_Colorectal_cancer_Main_Pathway	-0,31045	-0,45717	-0,65919	0,05294	1,055926	0,321885	-0,69389	-0,11215	-1,71053	1,385643	1,076457	0,911366	-0,05264	0,558616	0,461711	-5,59817	-3,44722	-2,95039	0,095338	0,253178
reactome_Inhibition_of_replication_initiation_of_	0,178895	-0,00068	0,119702	0,186079	0,544293	-0,13246	2,101665	2,365864	1,527113	-0,73982	-0,29926	-0,57898	-0,61619	-0,60678	-0,44961	-4,0153	-3,10217	-2,32045	0,095332	0,253178
GSK3_Signaling_Pathway_Gene_Expression_via_(	2,068004	1,09778	0,751708	-0,80354	0,483725	-0,94606	0,970376	0,943176	-1,04791	-1,464	0,446448	0,889622	1,327826	1,771546	1,677008	-14,8561	-11,857	-9,37962	0,095599	0,253183
histamine_biosynthesis	-0,16112	-0,10034	-0,03763	0,384757	-0,00243	0,01318	0,012872	0,059099	0,053889	0,184592	-0,03268	-0,34116	0,028233	0,016241	0,044741	-0,28464	0,178521	-0,06845	0,095748	0,253183
KEGG_Cardiac_muscle_contraction_Main_Pathwa	0,170934	-0,08027	0,030474	-0,86632	-1,60547	0,112074	-0,71645	-1,13175	-0,82643	0,152625	0,186539	-0,52557	-0,26101	0,036802	-0,19698	0,304375	0,278931	0,314753	0,095828	0,253183
L-dopachrome_biosynthesis	0,02499	0,005789	0,057236	-0,21735	-0,02654	-0,05853	-0,02336	-0,02569	0,030678	0,608155	-0,11543	-0,05783	0,003667	0,046102	0,05641	0,328428	0,19633	0,051255	0,095613	0,253183
reactome_temp_Nuclear_Receptor_transcription_	-0,90683	-0,27195	-0,67324	-4,01338	-1,55961	-2,97626	-0,03597	-0,85459	0,003059	-1,84508	0,043681	-0,10244	-0,75169	-1,48493	0,226031	0,035869	0,457472	-0,90252	0,095704	0,253183
S-reticuline_biosynthesis	0,02499	0,005789	0,057236	-0,21735	-0,02654	-0,05853	-0,02336	-0,02569	0,030678	0,608155	-0,11543	-0,05783	0,003667	0,046102	0,05641	0,328428	0,19633	0,051255	0,095613	0,253183
reactome_FGFR1b_ligand_binding_and_activatio	-0,13785	-0,11372	-0,06774	-0,16891	0,523033	0,150733	-0,4234	-0,44138	-0,13375	0,111627	-1,10247	0,037021	-0,00341	-0,46393	0,041672	-0,4358	-0,67032	-0,55994	0,096063	0,25359
reactome_Oxidative_Stress_Induced_Senescence	0,151529	-0,54929	-0,72878	-3,12699	-2,01536	-2,4821	0,265811	0,988049	-1,39067	-3,84993	-0,42908	-1,47661	2,298041	1,654019	1,545809	-7,71914	-6,27807	-4,74701	0,09645	0,254395
Ras_Pathway_CDC42_Pathway	0,03253	-0,10284	-0,18186	0,452262	0,478045	0,538365	-0,13858	-0,05065	-0,41134	-0,02634	0,222275	0,486889	-0,14078	0,039149	-0,26831	-1,53588	-1,00644	-0,3312	0,09665	0,254608
reactome_cGMP_effects_Main_Pathway	-0,19966	0,421205	0,039357	-1,82126	-1,08611	-2,3672	-0,25868	-0,22897	0,078137	1,548701	-0,54791	-0,3164	0,89589	1,177527	0,998033	3,982035	2,960566	2,722992	0,096694	0,254608
KEGG_Pyruvate_metabolism_Main_Pathway	0,484742	0,338526	0,200072	-0,72512	-0,39785	-2,57341	1,665044	2,214079	1,122211	-1,83257	1,043977	-0,90729	0,875074	1,515012	1,010592	-9,19595	-9,04094	-6,87853	0,097125	0,25516
reactome_Synthesis_of_PE_Main_Pathway	-0,0589	-0,0233	-0,07976	0,011683	0,407178	-0,0585	-0,63447	-0,51747	-0,58154	-0,04089	-0,13202	-0,18909	-0,84101	-0,69229	-0,33871	0,355153	1,154775	0,785212	0,097149	0,25516
trehalose_degradation	-0,0913	-0,05113	-0,10243	-0,50902	-0,13308	-0,37684	0,570572	0,543422	0,530418	0,082651	0,079305	-0,35364	-0,05387	0,162606	0,00768	0,979694	0,607444	0,231569	0,097039	0,25516
EGF_Pathway_Rab5_Regulation_Pathway	0,021027	0,074954	0,007466	0,251296	0,266557	0,212586	0,512224	0,521773	0,40457	-0,25716	0,230509	-0,52932	-0,20315	-0,02516	-0,05562	-0,32018	-0,19611	-0,1564	0,097662	0,256075
reactome_Regulation_of_gene_expression_by_H	-0,09425	-0,12545	-0,17677	-0,48407	-0,13898	-0,5892	-0,61496	-0,74744	-0,94966	0,08547	-0,23639	-0,07843	0,434028	0,194966	0,475564	-1,89279	-1,68337	-0,98646	0,097612	0,256075
Transport_between_Cytoplasm_and_Mitochondr	-0,02382	-0,07768	-0,01629	0,033951	0,48783	-0,01468	-0,43238	-0,41848	-0,63983	-0,18765	-0,30385	0,266153	0,266218	0,095046	-0,08461	-0,1964	-0,04118	0,052876	0,097812	0,256253
reactome_Synthesis_of_Prostaglandins_PG_and_	0,015673	0,186838	0,147651	-2,34327	-1,04281	-2,24066	-0,73295	-0,90589	-0,66599	0,284722	0,547559	0,657079	0,395449	0,004941	1,109802	1,166187	1,141177	0,368417	0,098026	0,256598
biocarta_trka_receptor_signaling_Pathway_(cell_	0,036844	-0,01723	-0,07572	-0,17332	0,72482	0,320766	-0,08537	-0,07371	-0,3174	0,395064	0,425208	0,322089	0,212651	0,160115	0,201692	-1,36902	-0,70657	-0,58663	0,098579	0,256733
NCI_IFN_gamma_Pathway_(Antibacterial_Respon	0,267972	-0,00782	-0,15235	-0,12461	-0,26529	-0,01588	-0,00085	0,338265	-0,42712	-1,42863	-0,0962	-1,39375	-0,06271	-0,12058	0,322483	-2,03719	-0,75858	0,32423	0,098737	0,256733
NCI_IFN_gamma_Pathway_(antigen_processing_a	0,267972	-0,00782	-0,15235	-0,12461	-0,26529	-0,01588	-0,00085	0,338265	-0,42712	-1,42863	-0,0962	-1,39375	-0,06271	-0,12058	0,322483	-2,03719	-0,75858	0,32423	0,098737	0,256733
NCI_IFN_gamma_Pathway_(antigen_processing_a	0,267972	-0,00782	-0,15235	-0,12461	-0,26529	-0,01588	-0,00085	0,338265	-0,42712	-1,42863	-0,0962	-1,39375	-0,06271	-0,12058	0,322483	-2,03719	-0,75858	0,32423	0,098737	0,256733
NCI_IFN_gamma_Pathway_(Antiviral_Response)	0,267972	-0,00782	-0,15235	-0,12461	-0,26529	-0,01588	-0,00085	0,338265	-0,42712	-1,42863	-0,0962	-1,39375	-0,06271	-0,12058	0,322483	-2,03719	-0,75858	0,32423	0,098737	0,256733
NCI_IFN_gamma_Pathway_(apoptosis)	0,267972	-0,00782	-0,15235	-0,12461	-0,26529	-0,01588	-0,00085	0,338265	-0,42712	-1,42863	-0,0962	-1,39375	-0,06271	-0,12058	0,322483	-2,03719	-0,75858	0,32423	0,098737	0,256733
NCI_IFN_gamma_Pathway_(Immunoregulation)	0,267972	-0,00782	-0,15235	-0,12461	-0,26529	-0,01588	-0,00085	0,338265	-0,42712	-1,42863	-0,0962	-1,39375	-0,06271	-0,12058	0,322483	-2,03719	-0,75858	0,32423	0,098737	0,256733
NCI_IFN_gamma_Pathway_(negative_regulation_	0,267972	-0,00782	-0,15235	-0,12461	-0,26529	-0,01588	-0,00085	0,338265	-0,42712	-1,42863	-0,0962	-1,39375	-0,06271	-0,12058	0,322483	-2,03719	-0,75858	0,32423	0,098737	0,256733
heparan_sulfate_biosynthesis	-0,93713	-0,61057	-1,01327	1,520653	-2,47589	-0,31099	-0,41412	-0,24616	-0,52454	-1,32971	-0,67354	-0,6808	1,147929	-0,14235	1,21125	-0,937	-1,3703	-2,23179	0,098875	0,256877
reactome_CRMPs_in_Sema3A_signaling_Main_Pa	-0,09333	-0,17099	-0,22267	-0,00858	0,030106	-0,493	-3,65196	-3,5893	-4,12024	-1,72123	-1,13363	-0,65175	0,258545	0,14877	0,210456	-0,24293	-0,40625	0,727028	0,099253	0,257643
2-oxobutanoate_degradation	0,284915	0,23801	0,110633	-0,35579	-0,08359	-0,10635	0,253075	0,506532	-0,40604	-0,04341	0,521085	-0,27647	0,04386	0,242976	0,68689	-4,62279	-4,0348	-3,69364	0,099451	0,257944
NCI_ErbB1_downstream_signaling_Pathway_(lam	0,057037	0,090769	0,190948	0,346145	-1,31081	-0,50895	0,752229	0,82217	1,005877	-0,3021	-0,67326	0,428064	0,09536	-0,14522	0,068751	1,691601	0,86399	0,920743	0,099768	0,258551
Akt_Signaling_Pathway_Glycogen_Synthesis_and_	0,0047	-0,04736	0,346364	-0,65453	0,362012	-1,19283	0,216119	0,156849	0,399195	0,230058	1,234605	-0,34303	0,283455	0,267537	0,066147	0,318709	1,133311	1,062286	0,099975	0,258871
NCI_CDC42_signaling_events_Pathway_(actin_cyt	-0,26405	-0,24817	-0,37419	-0,22253	-0,5069	-0,6094	-2,38553	-2,2793	-2,42361	-1,01642	0,58639	-0,65301	-0,08395	-0,22905	0,11201	-2,91838	-2,05594	-1,46146	0,100357	0,259482
NCI_EPO_signaling_Main_Pathway	-0,37619	-0,3697	-0,49864	-0,78317	0,148655	-0,39579	-2,03808	-2,08749	-2,46629	-1,09283	0,280603	0,512473	-0,53758	-0,26449	0,115584	-5,46351	-4,07039	-3,87321	0,100377	0,259482
KEGG_Other_types_of_O_glycan_biosynthesis_M	-0,46487	-0,60544	-0,54084	0,095725	0,256369	1,510894	-0,9111	-0,52606	-1,5466	2,235183	1,805979	-0,07095	0,574762	-0,25556	1,188513	-2,39423	-5,00852	-3,20543	0,100935	0,259885
NCI_Endogenous_TLR_signaling_Pathway_(regula	-0,32409	-0,17398	-0,24713	-0,40084	0,323502	-0,24391	-2,82854	-2,67452	-2,6938	2,34904	1,368383	1,031301	-1,02903	-1,11974	-0,5697	-0,55166	-0,60145	-0,70804	0,101219	0,259885
NCI_Endogenous_TLR_signaling_Pathway_(regula	-0,32409	-0,17398	-0,24713	-0,40084	0,323502	-0,24391	-2,82854	-2,67452	-2,6938	2,34904	1,368383	1,031301	-1,02903	-1,11974	-0,5697	-0,55166	-0,60145	-0,70804	0,101219	0,259885
NCI_Endogenous_TLR_signaling_Pathway_(regula	-0,32409	-0,17398	-0,24713	-0,40084	0,323502	-0,24391	-2,82854	-2,67452	-2,6938	2,34904	1,368383	1,031301	-1,02903	-1,11974	-0,5697	-0,55166	-0,60145	-0,70804	0,101219	0,259885
NCI_IL1_mediated_signaling_events_Main_Pathw	0,295541	-0,38444	-0,21978	0,9554	1,192051	-0,35842	-1,95478	-1,48483	-2,80584	0,993191	0,975312	0,689061	0,558538	0,270795	0,151335	-2,13311	-2,6272	-1,6559	0,100975	0,259885
NCI_IL8_and_CXCR1_mediated_signaling_events_	-0,14873	-0,13398	0,118017	-0,23717	0,22579	-0,04209	-2,18139	-2,52572	-1,64571	1,110141	-0,14312	-0,72782	-0,63258	0,127942	0,375015	1,961409	2,34081	2,155764	0,101001	0,259885
PTEN_Pathway_Apoptosis	0,019592	-0,21292	-0,11634	1,084475	-0,25968	-0,06193	-0,16136	-0,47467	0,152907	0,964161	-0,25698	-0,1534	-0,43106	-0,54668	-0,60641	1,79584	1,522736	1,238216	0,101284	0,259885
reactome_Hedgehog_ligand_biogenesis_Main_Pa	1,710887	0,658509	0,424037	-1,1835	0,409808	-2,7658	1,010292	1,402462	-1,47731	-2,42314	0,679691	0,471512	0,901122	0,784127	1,747407	-22,9959	-19,0276	-15,8672	0,100789	0,259885
reactome_Transport_of_fatty_acids_Main_Pathw	-0,06188	-0,1522	-0,10951	-0,0643	-0,10232	0,318033	-0,13357	-0,18887	-0,05811	0,897616	0,345275	0,270119	-0,04464	-0,22602	-0,06155	0,560623	0,346498	-0,1079	0,100834	0,259885
reactome_TRAF6_mediated_IRF7_activation_in_T	-0,06988	-0,11339	-0,13324	0,57208	0,859666	0,144991	-1,10758	-1,04015	-1,42347	0,334861	0,910532	0,131837	0,011033	-0,11028	-0,03527	-1,63478	-1,32705	-1,03618	0,101452	0,260102
Erythropoietin_Pathway_GPI_Hidrolysis_and_Ca2	-0,17241	0,081909	0,008897	-0,34506	-0,00994	-0,3467	0,105313	0,157503	0,386277	0,360292	0,270526	-0,14547	-0,06013	-0,43801	-0,30959	-0,47047	-0,00966	-0,36277	0,101716	0,260564
tryptophan_degradation	-0,08983	0,104197	0,233325	-0,83311	-1,01542	-0,39682	-1,65616	-2,11402	-1,42552	-0,95939	-0,58097	-1,0754	0,077313	-0,79728	-0,63964	0,375181	1,159274	-0,10388	0,102129	0,261406
cAMP_Pathway_Metabolic_Energy	-0,46233	-0,45347	-0,53799	1,993461	2,234162	2,165925	1,280656	1,537395	0,869449	0,006818	1,74019	2,353821	-0,78859	-0,44974	-0,00337	-4,59529	-3,69335	-1,66734	0,102238	0,261471
reactome_NCAM1_interactions_Main_Pathway	-0,8731	-0,29786	-0,11411	-1,67445	-2,39821	-2,37199	-1,72298	-2,20667	0,126361	0,346997	-2,37956	-2,16906	0,596276	-0,70679	0,969429	7,750076	6,550261	6,379387	0,102492	0,261907
biocarta_phospholipids_as_signalling_intermedia	0,036617	-0,13556	-0,10237	0,238521	0,061386	-0,20809	0,331175	0,435178	0,031596	1,114358	0,195946	-0,85273	-0,27045	0,259449	0,185227	-3,59171	-3,49736	-2,1678	0,103033	0,26217
ILK_Signaling_Pathway_Actin_Polymerization_Cyt	-0,02251	-0,02282	-0,07009	-0,55695	-0,14949	-0,85054	-1,90696	-2,13237	-2,04164	-1,15228	0,39209	0,46333	0,976934	1,004271	0,672407	-2,10059	-1,64088	-1,55452	0,102774	0,26217
KEGG_Primary_immunodeficiency_Main_Pathwa	-0,01521	0,769918	0,419643	-1,0325	0,937128	0,471164	2,316793	1,533215	4,708772	2,96651	1,342779	-2,52461	0,558952	-0,92182	0,526108	4,70467	2,927664	2,843131	0,103185	0,26217
NCI_Ephrin_B_reverse_signaling_Pathway_(angio	-0,23105	-0,15547	-0,28193	0,375074	1,481115	0,225756	-2,1315	-2,33832	-1,91821	-0,41795	-0,66206	-0,41188	0,168142	0,15729	0,437955	1,133432	0,429428	0,409945	0,10307	0,26217
NCI_Ephrin_B_reverse_signaling_Pathway_(endo	-0,23105	-0,15547	-0,28193	0,375074	1,481115	0,225756	-2,1315	-2,33832	-1,91821	-0,41795	-0,66206	-0,41188	0,168142	0,15729	0,437955	1,133432	0,429428	0,409945	0,10307	0,26217
reactome_Activation_of_PKB_Main_Pathway	-0,0406	-0,06564	-0,11818	0,335371	0,620402	0,550841	-1,5023	-1,49121	-1,63484	0,638884	0,740665	0,189063	0,320157	-0,19154	0,129657	-0,8101	-1,17445	-0,57758	0,10307	0,26217
reactome_PKA_mediated_phosphorylation_of_C	-0,05236	-0,09582	-0,05305	-0,01753	0,072809	-0,31911	-0,12819	-0,03912	-0,16291	-0,17167	-0,40999	0,135532	-0,2541	-0,1573	0,139187	-1,34351	-1,04516	-0,76056	0,103147	0,26217
biocarta_regulators_of_bone_mineralization_Mai	-0,01781	-0,0889	0,140181	-1,77415	-0,81777	-1,73219	1,102475	0,750746	1,170914	-0,64817	-0,53759	0,522222	-0,15394	0,201554	0,233603	0,917657	0,395197	0,156222	0,103323	0,262198
reactome_Ca_activated_K_channels_Main_Pathw	-0,30134	-0,06801	-0,10288	-0,29247	0,08899	-0,45719	-0,71799	-0,78314	-0,26942	1,792295	0,163493	0,771302	0,68874	1,182697	0,630465	3,775293	2,962244	3,491239	0,103364	0,262198
mTOR_Pathway_Translation_Elongation	0,363685	0,134941	0,08697	-0,34114	0,043497	-0,64752	-0,07341	0,254242	-0,69984	-0,00973	-0,02445	-0,6677	0,129649	-0,0603	0,323443	-4,78974	-3,88294	-3,66323	0,103494	0,262315
NGF_Pathway_Neurite_Outgrowth_and_Differen	-0,5888	-0,49916	-0,32634	0,025919	0,974647	0,893486	0,046052	0,27944	-0,02388	1,559197	0,215773	2,162075	0,518729	0,276987	0,751941	-3,84273	-2,33188	-1,549	0,103758	0,262668
TGF-Beta_Pathway_Epithelial_Mesenchymal_Tra	-0,32488	-0,23736	-0,24184	0,218646	0,228394	0,560306	0,50295	1,008533	0,1469	1,008053	-0,27383	1,290146	-0,18351	0,105393	0,462103	-3,65928	-2,70472	-1,98436	0,103802	0,262668
biocarta_apoptotic_signaling_in_response_to_dn	0,233497	0,084593	0,017156	0,03447	-0,05355	0,171369	-0,44556	-0,18759	-0,71636	0,837188	-0,59751	-1,03332	0,01028	-0,05788	-0,29776	-2,30166	-1,78898	-1,69971	0,103988	0,26271
reactome_Androgen_biosynthesis_Main_Pathwa	-0,32645	0,053156	-0,15166	0,548186	-0,03212	-0,13204	0,349984	0,085612	0,891162	1,797698	1,304023	0,373147	0,225329	-0,36725	0,078248	0,682727	0,4805	0,392143	0,103911	0,26271
Chromatin_Pathway	0,000624	-0,37135	-0,16675	0,07607	0,056531	-0,25766	0,365633	0,618467	-0,34146	-1,2015	0,794829	0,404875	-0,32442	0,069712	-0,10113	-5,05685	-3,50859	-2,62967	0,104176	0,262973
gamma-glutamyl_cycle	0,003621	0,000185	-0,05143	0,136756	0,097403	0,307142	-0,43185	-0,46682	-0,59081	0,262006	0,492348	0,702706	0,367848	0,472781	0,421705	-0,64336	-0,26744	-0,15448	0,104642	0,26368
NCI_Alpha4_beta1_integrin_signaling_events_Pat	-0,09421	0,065369	-0,15754	-1,64401	-0,12757	-1,71171	0,308538	0,440974	0,205858	0,058836	0,614019	-0,09321	-0,34302	-0,29924	-0,0627	-0,80974	-0,05311	-0,12114	0,104548	0,26368
PTEN_Pathway_Neuronal_Survival	-0,24036	0,189166	0,092994	0,247449	0,643576	0,018901	-0,85442	-0,89357	-0,53833	0,815516	0,000471	-0,4652	-0,05017	-0,29339	-0,22829	2,22297	2,055068	1,967067	0,10471	0,26368
heme_degradation	0,070861	-0,06439	-0,08849	0,551273	-0,17233	-0,33124	-0,45901	-0,36876	-0,38631	-0,15076	-0,12748	-0,86005	-0,10841	0,325053	-0,09131	-0,26236	-0,66235	-0,32594	0,104834	0,263779
KEGG_Proteoglycans_in_cancer_Main_Pathway	-2,46792	-2,96356	-2,19412	-1,22548	-0,54436	-3,04829	-7,33226	-7,51648	-7,22763	-3,39775	2,796454	0,880009	0,943314	2,072909	1,642286	-9,24556	-3,6265	-1,40811	0,10531	0,264122
reactome_CREB_phosphorylation_Main_Pathway	0,086486	-0,22905	-0,17808	-0,40449	-0,88079	-0,5086	-0,8113	-0,56948	-1,17507	-1,34227	0,137474	-0,66518	-0,02819	0,258863	0,198502	-1,75617	-1,52742	-1,43227	0,105118	0,264122
reactome_Establishment_of_Sister_Chromatid_C	0,466773	0,074808	0,090125	0,816315	0,352538	0,5329	2,256283	2,512923	1,212699	-1,32438	0,623947	-0,27205	-0,54472	-0,04307	-0,28747	-3,11162	-1,90738	-0,71962	0,105233	0,264122
reactome_Sialic_acid_metabolism_Main_Pathwa	0,132412	-0,24515	-0,03888	0,455032	-1,01839	0,841511	-4,65714	-4,35067	-5,17131	0,068556	0,226298	0,3027	0,580833	0,662	0,372754	-1,94302	-1,41977	-0,23912	0,105219	0,264122
reactome_Synthesis_of_bile_acids_and_bile_salts	-0,38557	0,002195	0,127838	-2,38757	-0,47207	-1,14447	-1,35526	-1,78522	-0,64434	-0,61277	0,114211	0,753703	1,14869	0,294317	0,412097	2,888594	2,874763	1,901514	0,105565	0,264549
Akt_Signaling_Pathway_Regulation_of_Na+_Tran	0,086324	-0,06935	0,021033	0,013769	0,527934	0,360053	-0,67008	-0,67876	-0,86323	-0,22839	-0,5893	-0,96809	-0,03061	0,155528	0,142381	0,198323	0,255448	0,390781	0,105879	0,264923
Chemokine_Ligand_2_Signaling	-0,49608	-0,44319	-0,67677	0,253852	-1,83007	-0,26763	-2,46219	-2,50125	-2,8693	-1,6935	-1,85029	0,795198	-0,46272	-0,53148	-0,32421	-3,43641	-2,9814	-2,37839	0,106092	0,264923
reactome_Aflatoxin_activation_and_detoxificatio	0,128508	0,099754	-0,07126	-0,85229	-0,37103	-1,29467	0,040962	-0,35863	0,023135	0,667612	0,176738	0,953255	0,535854	0,498434	0,41428	1,019021	0,783449	0,446287	0,106036	0,264923
reactome_TRAIL_signaling_Main_Pathway	-8,86E-05	-0,01219	0,021648	-0,41145	-0,38427	-0,76664	-0,9035	-0,77402	-0,68787	-0,4431	0,207656	0,660886	0,486441	-0,07853	-0,12112	0,535459	0,502113	-0,0355	0,106139	0,264923
tRNA_charging	0,846928	-0,15459	0,084043	-1,1971	-0,88116	-1,80458	-1,79126	-1,18364	-3,97659	-0,61648	0,031555	1,520309	0,990606	1,543084	1,485221	-16,5555	-13,9672	-11,3639	0,106001	0,264923
glutamine_degradationglutamate_biosynthesis	-0,08577	0,108817	-0,007	-0,03345	0,110209	-0,03251	-0,51992	-0,46347	-0,5579	0,069017	-0,46165	-0,29718	0,165443	0,010045	-0,0097	-0,44748	0,017057	0,093368	0,106557	0,265754
Hypoxia_induced_EMT_in_cancer_and_fibrosis_3	-0,02415	-0,30301	-0,14619	-0,23066	0,281732	-0,11584	-1,95502	-1,75641	-1,6739	-0,15952	-0,06588	0,472655	0,261161	0,395719	0,130538	-0,00549	-1,18022	-0,25596	0,106915	0,26622
RhoB_Signaling	-0,08265	-0,05047	-0,10115	-0,25948	0,584665	-0,49804	-0,85426	-0,8777	-0,87337	-1,12042	0,37888	-0,23517	0,182732	0,227583	0,490482	-0,57687	-0,19417	-0,43307	0,1069	0,26622
KEGG_AMPK_signaling_Main_Pathway	0,577424	-0,0332	0,708517	-5,46747	-4,82825	-3,65937	-1,37557	-1,18633	-1,26288	-3,459	-1,1838	-1,36756	-1,03791	-1,52251	-2,33058	-0,95967	-3,82479	-3,71923	0,107043	0,266326
NCI_Aurora_A_signaling_Pathway_(proteasomal_	-0,09003	0,01447	-0,08224	-0,18347	-0,33301	0,02873	0,947441	0,909111	0,900386	-1,467	-0,89948	-0,99278	-0,16011	-0,0716	-0,01724	-0,47433	0,022778	-0,14458	0,107245	0,266614
KEGG_Pancreatic_secretion_Main_Pathway	0,066985	-0,26843	0,204408	-0,59557	0,714466	-1,5001	-0,95436	-0,95432	-0,72704	1,654173	-0,44132	0,780968	0,489108	0,792079	1,122914	3,400601	3,169251	3,788986	0,107624	0,267345
BRCA1_Pathway_E2_Responsive_Genes	0,247401	0,208864	0,260123	-0,15887	0,151453	-0,22293	0,519844	0,541202	0,408639	2,041761	1,873177	0,861841	-0,59103	-0,58651	-0,71497	-0,82759	-0,85609	-0,80214	0,108369	0,268198
glycolysis	0,139678	-0,03344	-0,09362	-0,29786	0,863588	-0,03574	-0,97083	-0,93769	-0,70136	-1,85435	0,585989	-0,23224	0,47837	-0,12827	0,245304	-0,13349	-0,84314	-0,34969	0,108578	0,268198
itrans_transi-farnesyl_diphosphate_biosynthesis	0,17613	0,059534	0,031715	0,043209	0,183318	0,686177	0,60332	0,738072	0,453787	-0,59308	0,41903	0,128205	-0,17867	-0,24414	-0,04154	-2,39943	-1,36276	-1,20471	0,108327	0,268198
KEGG_Insulin_secretion_Main_Pathway	-1,05782	-0,38393	-0,05458	-1,0266	-2,15416	-2,42246	-0,80565	-0,8416	-0,002	-0,01831	-3,30148	-0,51893	-0,59816	0,214573	1,514219	9,036762	8,516124	9,716367	0,108145	0,268198
NCI_CD40_CD40L_signaling_Pathway_(B_cell_affi	0,037412	0,041113	-0,00937	0,066307	-0,51509	-0,17001	-0,36482	-0,24772	-0,62167	-0,82871	0,638019	-0,41223	0,35798	0,366944	0,142805	-0,91258	-0,73617	-1,14419	0,108564	0,268198
NCI_CD40_CD40L_signaling_Pathway_(plasma_ce	0,037412	0,041113	-0,00937	0,066307	-0,51509	-0,17001	-0,36482	-0,24772	-0,62167	-0,82871	0,638019	-0,41223	0,35798	0,366944	0,142805	-0,91258	-0,73617	-1,14419	0,108564	0,268198
NCI_Integrin_linked_kinase_signaling_Main_Path	-0,07003	-0,28984	-0,50964	0,652101	0,514919	0,129117	-2,49892	-2,59459	-3,32422	-0,81205	2,94164	1,885252	0,304182	0,641618	0,549787	-8,68383	-7,01097	-5,0265	0,108432	0,268198
NCI_PDGFR_beta_signaling_Pathway_(chemotaxi	-0,19371	-0,1207	-0,14996	0,398212	0,813177	0,25903	0,629692	0,504336	0,643193	-0,31756	-0,14736	-0,27623	-0,14232	-0,32487	0,334938	-0,0417	0,083145	0,21689	0,108938	0,268198
NCI_Signaling_events_mediated_by_Stem_cell_fa	0,059879	0,051511	0,047507	-0,18495	-0,31454	-0,22144	-0,19593	-0,1666	-0,48692	-0,41861	-0,31999	-0,32367	-0,11103	-0,12878	-0,06406	-1,57031	-2,11087	-1,34274	0,108817	0,268198
reactome_Endosomal_Sorting_Complex_Require	0,49666	-0,26898	-0,22753	-2,14194	-1,31848	-0,65753	1,410957	1,810517	0,544366	-1,95715	-1,56198	-1,47608	-0,51765	-0,78101	-0,01111	-9,78512	-7,75133	-6,03928	0,108899	0,268198
reactome_Heme_biosynthesis_Main_Pathway	0,230845	0,0554	0,080093	-0,02948	-0,18813	0,864204	0,081905	0,126698	-0,44593	-0,09663	0,292091	0,247944	0,314522	-0,05854	0,049005	-1,08214	-1,27605	-1,67616	0,108826	0,268198
reactome_The_retinoid_cycle_in_cones_daylight_	-0,1775	-0,05642	0,169225	-1,11789	0,147302	0,346893	0,399604	0,237814	0,310773	-0,6468	0,098402	-0,57038	-0,51109	-0,67346	-0,80531	0,404219	0,348243	1,157197	0,109001	0,268198
reactome_Deadenylation_of_mRNA_Main_Pathw	0,326402	0,080922	0,223645	-0,46368	-0,52148	-1,3737	-0,1905	0,455349	-1,2777	-1,66639	1,073325	-0,97714	0,599933	0,406795	0,75403	-7,97194	-5,07729	-3,84223	0,109352	0,268848
KEGG_Huntington_s_disease_Main_Pathway	0,178658	-0,12373	-0,00134	0,292588	0,727611	0,279343	-0,57732	-0,46341	-0,85501	-1,48322	-0,19698	-0,29124	0,672603	0,45897	0,704944	0,47911	0,869688	1,089785	0,109943	0,269877
reactome_Synthesis_of_glycosylphosphatidylinosi	0,8172	0,254103	0,348757	1,185794	-0,25515	0,6431	1,432728	2,230114	0,23037	-1,79302	-0,70967	0,097118	-0,96415	-0,48447	-0,59026	-5,00593	-3,54658	-3,13073	0,109858	0,269877
NCI_ATM_Pathway_(double_strand_break_repair	0,074457	-0,27221	-0,23547	-0,13169	0,807192	0,302525	0,097217	0,437251	-0,53752	0,114403	0,246825	0,310489	-0,23929	-0,17291	0,057144	-2,17965	-1,23722	-0,86144	0,110117	0,270091
reactome_Clearance_of_Nuclear_Envelope_Mem	-0,01757	0,068574	0,060175	-0,55351	0,032572	0,510582	0,432615	0,60149	0,280914	-0,63574	0,407053	-1,16871	-0,0187	-0,42207	0,004538	-1,37051	-1,26071	-0,99508	0,110343	0,270432
NCI_CDC42_signaling_events_Pathway_(retrogra	-0,00851	-0,11084	-0,08937	0,147012	-0,32376	-0,19682	-1,1487	-1,20201	-1,42503	-0,86638	0,255521	-0,21085	-0,3074	-0,47323	0,008304	-3,96934	-2,74443	-2,06175	0,110657	0,270988
KEGG_mTOR_signaling_Main_Pathway	-0,85741	-0,05099	-0,27694	-1,63204	0,018864	0,589878	-0,23685	-0,70768	0,580809	1,884852	-2,00431	1,132028	-0,21595	0,14721	-0,32384	4,351048	3,195849	2,159035	0,111287	0,272315
reactome_Eukaryotic_Translation_Elongation_Ma	-0,09928	-0,14731	-0,21658	0,402379	-0,44485	-0,06619	0,347943	0,332224	0,359062	-0,30694	-0,06817	-0,03876	0,066982	-0,40946	-0,25091	-1,24431	-1,68998	-0,9739	0,111615	0,272905
Chemotaxis_Driven_by_CCL2	0,057255	-0,40989	-0,48368	2,582332	0,790287	0,89399	-0,75055	-0,91204	-1,85115	-3,05694	2,152016	1,98331	-1,2629	-1,38916	-0,37902	-8,23737	-7,39929	-4,96747	0,111743	0,273003
Akt_Signaling_Pathway_Cell_Cycle_Progression	-0,12111	-0,16922	0,277631	-1,44915	-0,17611	-1,24429	0,315424	0,333155	0,575767	0,34573	0,875976	-0,61658	0,57818	0,426384	0,212346	0,701023	1,468099	1,585697	0,111881	0,273127
reactome_NADE_modulates_death_signalling_M	-0,04083	-0,04292	0,032505	0,156519	0,379991	0,237268	-0,28958	-0,33196	-0,33657	0,227829	0,357462	0,429376	0,190776	-0,01651	0,147984	-0,67944	-0,51077	-0,24798	0,112065	0,27336
thio-molybdenum_cofactor_biosynthesis	-0,0477	0,06853	-0,03507	0,071495	-0,2722	-0,3159	-0,00484	0,009704	0,052787	0,000885	-0,09719	-0,04357	-0,21081	-0,33028	0,093872	-0,01879	0,313761	-0,14091	0,11243	0,274037
cAMP_Pathway_Endothelial_Cell_Regulation	0,525567	0,596894	0,515999	-1,60527	-0,78726	-1,50375	1,855336	2,398003	1,877904	2,222931	0,491943	-0,5457	-1,02468	-0,76696	-0,0879	-1,3311	-1,74714	-1,71904	0,112761	0,274393
NCI_FAS_CD95_signaling_Main_Pathway	0,146411	0,080064	-0,46822	-0,82128	1,239429	0,724277	-0,16467	0,17246	-0,82451	0,741658	1,192006	1,086217	0,738294	0,131301	0,682086	-5,53381	-4,57239	-2,51848	0,113017	0,274393
NCI_Stabilization_and_expansion_of_the_E_cadh	-0,02616	-0,03828	-0,10053	0,392579	0,190342	-0,61003	-2,19119	-2,00928	-2,81753	-0,20382	1,093901	0,685242	0,071812	0,553299	0,560491	-5,88062	-3,31376	-3,19121	0,112996	0,274393
reactome_Regulation_of_AMPK_activity_via_LKB	0,096099	-0,27734	-0,24958	-0,88075	-0,31545	-2,13665	-0,21552	0,072656	-0,85899	-2,6292	-0,66004	-0,27733	0,071798	0,324698	0,113571	-4,22008	-3,42895	-2,797	0,11281	0,274393
superpathway_of_methionine_degradation	0,393317	0,508726	0,294896	-0,63046	-0,47606	-0,38757	0,123214	0,520363	-0,70319	-0,42529	-0,03176	-0,68164	-0,13221	-0,18583	0,83748	-6,24509	-5,94945	-5,34628	0,112856	0,274393
KEGG_Glycine_serine_and_threonine_metabolisn	0,12377	0,227839	0,359259	-2,31651	-1,34794	-2,57976	1,075233	0,949843	1,28876	2,944032	1,133642	3,212064	-0,25823	0,105541	0,52722	-1,25117	-2,32206	-0,86379	0,11311	0,274406
cholesterol_biosynthesis_I	0,310437	0,187066	-0,00041	1,935505	1,364305	2,584686	1,248235	1,397201	0,837745	0,499059	1,052252	1,780455	-0,57348	-0,28607	-0,41056	-4,80282	-3,72672	-3,04301	0,1142	0,27619
cholesterol_biosynthesis_II_via_2425-dihydrolanc	0,310437	0,187066	-0,00041	1,935505	1,364305	2,584686	1,248235	1,397201	0,837745	0,499059	1,052252	1,780455	-0,57348	-0,28607	-0,41056	-4,80282	-3,72672	-3,04301	0,1142	0,27619
cholesterol_biosynthesis_III_via_desmosterol	0,310437	0,187066	-0,00041	1,935505	1,364305	2,584686	1,248235	1,397201	0,837745	0,499059	1,052252	1,780455	-0,57348	-0,28607	-0,41056	-4,80282	-3,72672	-3,04301	0,1142	0,27619
purine_ribonucleosides_degradation_to_ribose-1	-0,05101	-0,03394	0,01092	-0,0756	-0,17983	-0,00701	0,806768	0,91589	0,81312	-0,63251	0,361007	-0,58698	0,102873	0,171613	-0,05583	-0,36754	-0,33112	-0,2388	0,113961	0,27619
NCI_Arf6_downstream_Main_Pathway	0,088835	-0,04851	-0,09971	-0,05261	-0,70381	-1,20365	-1,17636	-1,04775	-1,43454	-0,04004	-1,37396	-0,78737	0,456564	0,541104	0,27578	-2,03655	-1,57452	-1,10521	0,114767	0,277347
formaldehyde_oxidation	0,069792	0,083181	0,002666	-0,12425	-0,2661	-0,19452	-0,01037	0,113063	-0,11034	-0,72453	0,13163	-0,34735	-0,16457	0,112995	-0,10948	-1,45586	-1,41269	-0,92929	0,115986	0,280047
JAK-STAT_Pathway_Akt_Pathway	-0,31477	-0,33318	-0,25585	0,333177	1,100297	0,904512	0,997875	1,548474	0,525771	1,174202	0,360131	1,239588	-0,4005	-0,05128	0,441787	-3,79412	-2,30988	-1,41309	0,116064	0,280047
biocarta_aspirin_blocks_signaling_pathway_invol	0,070837	-0,19015	-0,28395	-0,59125	-0,98899	-0,7432	0,312909	0,426265	0,103239	-1,04739	0,15366	0,4959	0,547037	0,568378	0,841151	-2,99221	-3,40919	-2,27442	0,116168	0,280079
HGF_Pathway_Anoikis	-0,50074	-0,6078	-0,53911	-0,16562	-0,15723	0,497668	-0,95177	-0,16293	-2,01881	0,972039	0,149554	1,894638	0,230764	0,350141	0,982217	-4,95187	-2,80256	-2,12934	0,116869	0,281553
reactome_Signaling_by_SCF_KIT_Main_Pathway	-0,09329	-0,23307	-0,12057	0,515215	0,341296	0,525075	-0,41108	-0,49603	-0,63451	0,695932	0,084117	1,121323	-0,56681	0,166184	0,215736	-3,27502	-3,601	-2,02331	0,116979	0,2816
KEGG_Salivary_secretion_Main_Pathway	-0,09739	0,029977	-0,0004	-1,05503	-0,3658	0,927889	0,452486	0,152479	0,641039	-0,48109	0,045563	0,430738	-0,44284	-0,33589	-0,21301	1,711539	0,814935	0,534637	0,117196	0,281903
reactome_Membrane_binding_and_targetting_o	0,143573	-0,10348	-0,1751	-0,45932	-0,727	-0,06132	0,922501	1,126729	0,727593	-0,76248	-0,39394	-0,44833	-0,24593	-0,64181	0,148325	-3,81332	-3,13121	-2,10828	0,117694	0,282883
reactome_RNA_Polymerase_I_Chain_Elongation_	0,89935	0,00951	-0,60647	0,900025	-0,50431	-2,76979	2,552614	4,025622	0,453458	-0,31191	-0,06672	-0,06599	-0,212	-0,71434	1,095894	-14,6896	-11,0269	-10,3373	0,11791	0,283183
NCI_Arf6_trafficking_events_Pathway_(membran	-0,1288	-0,29338	-0,2453	0,327625	-0,75198	-0,75569	-0,24139	-0,24549	-0,52788	0,291112	-0,51512	-0,64025	0,729085	0,559732	0,210231	-0,9881	-0,95285	-0,79434	0,118176	0,283259
reactome_COPI_Mediated_Transport_Main_Path	0,159369	0,00531	-0,10622	-0,08704	-0,06301	0,101763	-0,23763	0,060274	-0,57064	-1,45055	0,665607	0,106178	-0,3168	-0,36306	-0,10591	-3,61783	-2,94791	-2,37897	0,118214	0,283259
reactome_Detoxification_of_Reactive_Oxygen_Sp	0,421474	-0,08145	-0,10407	1,206958	0,637354	-0,38431	0,622994	1,128773	0,317812	0,260767	0,855427	-0,34921	-0,29197	-1,36468	-0,10195	-8,61709	-7,15093	-6,09326	0,118065	0,283259

reactome_Voltage_gated_Potassium_channels_M	-0,59476	-0,34069	0,433102	-1,25772	0,76492	-1,151	-1,57631	-2,16191	0,76235	0,045189	-1,18727	-1,02318	0,775702	-0,75811	1,286898	11,55982	9,425794	11,01081	0,119097	0,285156
KEGG_Viral_myocarditis_Main_Pathway	0,081168	0,052285	0,111775	0,053806	0,075667	0,635384	-0,78468	-0,53205	-0,94621	0,237476	-0,49175	-0,71523	-0,16833	-0,26739	-0,32147	-1,30273	-1,08876	-0,39913	0,119433	0,28574
NCI_Signaling_mediated_by_p38_alpha_and_p38	0,366373	-0,65662	-0,7379	-2,06866	-0,86202	-1,5082	-2,49006	-2,33617	-3,60102	-1,73779	0,294035	-1,40924	0,985234	0,174056	0,787132	-5,46466	-3,84181	-2,99829	0,119627	0,285984
ascorbate_recycling_cytosolic	0,034196	0,123611	-0,01659	-0,29795	-0,00437	0,197368	-1,3606	-1,3413	-1,60045	-0,24426	-0,0204	-0,35694	-0,15347	0,007951	0,032297	-0,37617	-0,64516	-0,52751	0,119942	0,286518
KEGG_Glutamatergic_synapse_Main_Pathway	0,23018	-0,11848	0,331255	-2,28066	-2,77278	-3,75289	-4,54743	-3,69922	-3,57719	0,570884	-2,26819	-0,89932	0,884708	1,68551	1,705576	5,019201	7,523414	5,200161	0,120059	0,286578
TCA_cycle	0,528699	0,233276	0,029171	-1,15307	-0,61	-1,26065	0,476874	0,940723	-0,38735	-2,90086	0,211998	-0,25568	-0,42475	-0,13968	-0,15707	-8,80234	-8,40734	-8,13764	0,120216	0,286733
methionine_degradation	0,055721	0,082329	0,013246	-0,1606	-0,15144	0,104954	0,128648	0,303429	0,110998	0,301727	-0,5496	-0,31041	-0,0278	-0,21579	-0,05198	-1,28909	-1,27055	-1,09904	0,120958	0,287931
NCI_Arf6_trafficking_events_Main_Pathway	-0,19624	-0,14758	-0,31381	-0,46093	-1,34797	-1,10263	-4,28049	-4,27616	-5,62059	0,952674	-0,92919	0,609236	0,867075	0,930665	0,488606	-7,57712	-5,32189	-4,92986	0,120921	0,287931
reactome_Ca2_Main_Pathway	-0,23537	-0,53892	-0,34213	2,374142	2,745094	0,630458	0,060878	-0,09222	0,410782	-4,48314	1,019559	0,485827	-0,0064	0,781871	-0,09095	0,330416	-0,06573	1,955325	0,120996	0,287931
NCI_BARD1_signaling_events_Pathway_(protein_	0,082519	0,070634	0,022526	0,532548	-0,12551	0,868903	-0,69175	-0,55947	-1,08598	-0,42093	-0,08072	-0,16543	0,002734	-0,29013	-0,15505	-1,30745	-0,45894	-0,08832	0,121457	0,288807
reactome_Hyaluronan_uptake_and_degradation_	0,07666	0,230991	0,187668	-0,10291	0,385739	-0,78033	-1,34307	-1,32469	-1,38447	0,720727	0,347316	0,640035	-0,1056	0,170375	0,1808	0,029999	0,332023	-0,01169	0,121688	0,289137
NCI_Class_I_PI3K_signaling_events_Pathway_(cel	-0,02893	-0,14706	-0,24068	-0,54064	0,152903	0,492573	-0,71098	-0,7699	-0,99696	-0,39874	0,014044	-0,34991	-0,25156	0,472742	0,206554	-1,29644	-0,36756	0,273884	0,121979	0,289607
reactome_Gastrin_CREB_signalling_pathway_via_	-0,08813	-0,15182	-0,13235	-0,15104	-0,5853	-0,64803	-0,9058	-0,84063	-1,07629	-1,07147	0,25841	-0,13948	0,258497	0,325096	0,522874	-1,27857	-1,05527	-0,87648	0,122146	0,289782
125-dihydroxyvitamin_Dsub3sub_biosynthesis	-0,09547	-0,09773	-0,10926	0,031495	0,045845	0,248999	-0,2256	-0,13243	-0,25032	0,761285	0,80995	0,719607	0,019868	-0,03713	-0,23619	-0,0934	0,111558	0,348655	0,12237	0,289873
NCI_IL5_mediated_signaling_events_Main_Pathw	-0,10117	-0,32562	-0,09827	-0,52643	-0,00807	-0,40148	0,36864	0,51206	0,349198	0,889156	0,394104	0,47082	-0,54478	-0,45593	-0,12237	-1,0496	-0,10404	-0,08851	0,122347	0,289873
biocarta_yaci_and_bcma_stimulation_of_b_cell_i	0,262432	-0,24432	0,085543	-1,22687	-1,2722	-1,25555	-0,45475	-0,15605	-0,20493	0,889436	0,354453	0,332163	0,352278	0,180433	0,129622	0,047296	-0,17389	-0,51791	0,12315	0,291415
reactome_Signal_attenuation_Main_Pathway	-0,08823	-0,23666	-0,24044	-0,14516	-0,39402	-1,21305	-1,77197	-1,97911	-2,02571	-0,2317	-0,89282	-1,07599	0,694625	0,426817	0,644912	-1,35538	-1,10729	-0,09904	0,123208	0,291415
KEGG_Nicotine_addiction_Main_Pathway	-0,81359	0,395947	0,734079	0,012691	0,376724	-0,99628	-4,56861	-4,84069	-3,01025	2,957703	1,989793	0,59619	3,270261	2,228153	2,586664	13,21335	10,79824	10,63424	0,123624	0,292117
NCI_DNA_PK_pathway_in_nonhomologous_end_	0,219849	0,099445	0,057695	0,329901	0,420543	0,107088	0,013763	0,189073	-0,27148	-0,11142	0,044698	-0,40729	-0,35895	-0,08135	-0,09005	-1,32544	-0,75896	-0,35411	0,123693	0,292117
biocarta_ahr_signal_transduction_Main_Pathway	0,013691	-0,00034	-0,06062	-0,02379	-0,22195	-0,00042	-0,65915	-0,58683	-0,75865	0,636127	-0,26659	-0,02507	0,366243	0,217258	-0,18784	-1,67397	-1,54293	-1,13164	0,124137	0,292361
biocarta_atm_signaling_Pathway_(DNA_repair)	-0,03096	-0,1079	-0,10059	1,306572	0,987244	0,675584	0,381374	0,684051	0,220399	0,127446	1,003589	0,148094	-0,08444	-0,31752	-0,35304	-1,51471	-1,18469	-0,82443	0,12424	0,292361
biocarta_fmlp_induced_chemokine_gene_expres	-0,1125	-0,37594	-0,10317	0,020708	-0,58089	-0,86621	0,332476	0,924959	0,112331	0,181951	1,098096	-2,20444	-0,96891	-0,4253	0,390481	1,547325	1,503409	2,19189	0,124184	0,292361
reactome_CREB_phosphorylation_through_the_a	-0,21474	-0,14105	-0,1094	-0,11382	0,198807	-0,53763	-0,71488	-0,54192	-0,74004	-0,27854	-0,77389	0,014024	0,007296	0,139951	0,490412	-0,48441	-0,19386	-0,04541	0,124265	0,292361
reactome_Metal_ion_SLC_transporters_Main_Pa	-0,14308	0,008554	-0,23503	-0,83892	-0,02894	-0,51922	-1,03134	-1,0191	-1,40591	0,078231	0,491573	-0,17597	0,296528	0,428762	0,017563	-3,40588	-3,42766	-3,13188	0,124165	0,292361
NCI_RhoA_signaling_Pathway_(Golgi_organizatio	0,04093	0,054508	0,030899	-0,09262	-0,22224	-0,42841	0,119263	0,157536	0,128925	-0,42329	-0,11284	-0,6472	0,039402	-0,01882	-0,0036	-0,68792	-0,63941	-0,70232	0,124384	0,292421
KEGG_Aldosterone_regulated_sodium_reabsorpt	-0,22149	-0,09499	-0,04071	-0,95811	0,092154	0,229951	-0,38133	-0,44544	-0,28106	2,06291	0,340122	0,956618	-0,5367	0,271783	0,146334	-0,0475	0,410047	0,173523	0,124853	0,293157
L-dopa_degradation	-0,00067	-0,05655	-0,00989	-0,15492	0,029713	0,101154	-0,23246	-0,23768	-0,20226	-0,09219	-0,10213	-0,06483	0,093236	-0,03767	0,057107	-0,07356	-0,06518	-0,06625	0,125187	0,293157
NCI_ATM_Pathway_(chromatin_remodeling)	-0,02024	-0,3092	-0,25652	-0,45934	0,434989	-0,04597	0,064268	0,340453	-0,48033	-0,53553	-0,05246	0,428531	-0,19512	-0,22805	-0,05696	-2,03104	-1,15251	-0,99712	0,125356	0,293157
reactome_Interleukin_receptor_SHC_signaling_M	-0,14944	0,281279	0,162677	-0,77522	-0,29791	-0,92308	-0,27775	-0,74087	-0,08151	-0,24341	0,968738	0,867093	0,343105	0,013301	0,266777	-0,16157	0,288405	-0,08752	0,124924	0,293157
reactome_RMTs_methylate_histone_arginines_M	0,486639	-0,26317	-0,32691	1,328037	0,499312	0,801738	1,424208	2,218765	0,670263	0,737911	2,09005	-0,75165	-1,39167	-0,33999	0,607336	-7,39242	-6,08142	-3,71113	0,125266	0,293157
superpathway_of_pyrimidine_deoxyribonucleotic	0,371001	0,118886	0,021631	-0,57395	0,204654	-0,63914	2,224649	2,474799	1,174022	-0,18876	2,235129	1,163305	1,001629	1,317117	1,494807	-6,96306	-5,54717	-4,54204	0,125125	0,293157
TRAF_Pathway_Cell_Survival	-0,33419	-0,23969	-0,22555	0,210482	0,223238	0,2774	0,487794	0,930759	0,355997	0,942085	0,232617	1,606212	0,240092	0,295419	0,654659	-2,75431	-1,98382	-1,66651	0,125289	0,293157
ATM_Pathway_DNA_repair	0,188782	-0,06959	-0,04805	0,276867	0,820053	0,199125	1,132032	1,420043	0,481532	-0,37412	-0,02431	-0,58174	-0,52143	-0,51544	-0,43341	-2,37902	-1,53515	-0,71637	0,126022	0,294446
NCI_ATR_signaling_Main_Pathway	0,924549	0,320463	0,24593	0,772432	0,709393	0,284222	2,423016	3,513193	1,180826	2,072261	-0,18159	0,125955	-0,48139	0,322683	0,726886	-9,25079	-6,03051	-4,56458	0,126097	0,294446
biocarta_keratinocyte_differentiation_Pathway_(	0,13471	0,363479	0,183722	-0,96381	-0,005	0,642763	0,515076	0,428344	0,515055	-1,38011	-1,42321	-2,14682	0,48682	0,597034	0,336865	-0,02163	-0,35239	0,433856	0,12792	0,2945
biocarta_phospholipids_as_signalling_intermedia	0,013982	-0,17695	-0,08817	0,317843	-0,07728	-0,20011	0,274424	0,325287	0,02117	1,273386	0,081228	-1,37456	-0,47055	0,078117	-0,01074	-3,01122	-3,24032	-1,97116	0,127884	0,2945
biocarta_phospholipids_as_signalling_intermedia	0,013982	-0,17695	-0,08817	0,317843	-0,07728	-0,20011	0,274424	0,325287	0,02117	1,273386	0,081228	-1,37456	-0,47055	0,078117	-0,01074	-3,01122	-3,24032	-1,97116	0,127884	0,2945
biocarta_tgf_beta_signaling_Main_Pathway	0,110031	-0,174	-0,24828	-0,55408	-0,61981	0,149189	0,625516	0,848746	0,101696	-0,70966	-0,25871	-0,14869	-0,10992	0,306598	0,196426	-0,94376	-0,93253	-0,72637	0,126721	0,2945
biocarta_wnt_signaling_Main_Pathway	0,048075	0,077425	-0,05754	-0,20414	0,724997	-0,29855	-0,23441	-0,37471	-0,38837	1,494659	0,74411	0,689501	0,261863	0,184456	0,68962	1,276185	0,291002	1,444198	0,127708	0,2945
KEGG_One_carbon_pool_by_folate_Main_Pathw	0,651546	0,055074	-0,06914	-1,37533	-0,19581	-1,11472	0,660471	1,310029	-0,09273	-0,84028	0,689652	-0,86574	0,389036	0,375725	0,667547	-7,07933	-5,72713	-4,42251	0,126936	0,2945
NCI_ATR_signaling_Pathway_(regulation_of_doul	0,805553	0,219997	0,176439	0,487031	0,844864	0,515997	2,143048	3,093501	0,866927	1,514054	0,574353	0,57241	-0,37285	0,281278	0,578944	-9,17206	-6,3334	-4,70003	0,128011	0,2945
NCI_Endogenous_TLR_signaling_Pathway_(cell_m	-0,00639	-0,0484	-0,01952	-0,43459	-1,00886	-0,4377	-1,33618	-1,12629	-1,67764	1,587624	1,540782	1,11784	-0,46464	-0,67679	-0,71304	-1,36568	-1,20094	-1,17589	0,127197	0,2945
NCI_Endogenous_TLR_signaling_Pathway_(regula	-0,00639	-0,0484	-0,01952	-0,43459	-1,00886	-0,4377	-1,33618	-1,12629	-1,67764	1,587624	1,540782	1,11784	-0,46464	-0,67679	-0,71304	-1,36568	-1,20094	-1,17589	0,127197	0,2945
NCI_LPA_receptor_mediated_events_Pathway_(a	0,104012	-0,0888	-0,20414	0,383017	-0,39321	-0,22407	-0,13997	-0,08944	-0,10891	-0,54121	-0,08803	-0,29164	-0,41457	-0,47099	-0,29296	-2,49344	-2,27321	-1,65216	0,127819	0,2945
NCI_LPA_receptor_mediated_events_Pathway_(N	0,104012	-0,0888	-0,20414	0,383017	-0,39321	-0,22407	-0,13997	-0,08944	-0,10891	-0,54121	-0,08803	-0,29164	-0,41457	-0,47099	-0,29296	-2,49344	-2,27321	-1,65216	0,127819	0,2945
NCI_LPA_receptor_mediated_events_Pathway_(p	0,104012	-0,0888	-0,20414	0,383017	-0,39321	-0,22407	-0,13997	-0,08944	-0,10891	-0,54121	-0,08803	-0,29164	-0,41457	-0,47099	-0,29296	-2,49344	-2,27321	-1,65216	0,127819	0,2945
NCI_Trk_receptor_signaling_mediated_by_PI3K_a	-0,02783	-0,20912	-0,30407	-0,93804	0,220414	0,377445	-0,82242	-0,8289	-1,26032	0,082844	0,782453	0,019551	0,128475	0,119389	0,191797	-4,04676	-2,34954	-2,21456	0,126625	0,2945
NCI_Trk_receptor_signaling_mediated_by_PI3K_a	-0,02783	-0,20912	-0,30407	-0,93804	0,220414	0,377445	-0,82242	-0,8289	-1,26032	0,082844	0,782453	0,019551	0,128475	0,119389	0,191797	-4,04676	-2,34954	-2,21456	0,126625	0,2945
reactome_Abacavir_transmembrane_transport_M	0,187761	0,335547	0,3157	0,340247	-0,02441	-0,53717	0,187584	-0,00557	0,393536	1,101228	1,142326	0,387475	0,995889	0,708399	0,467959	1,696571	2,020988	0,389214	0,127672	0,2945
reactome_Citric_acid_cycle_TCA_cycle__Main_Pa	0,540386	0,279722	0,104842	-0,81456	0,017545	-1,0525	0,902173	1,377356	0,085514	-2,69384	0,851345	0,242965	-0,38369	-0,22248	-0,20553	-8,99193	-8,39212	-8,1653	0,126509	0,2945
reactome_mitochondrial_fatty_acid_beta_oxidati	0,144487	0,123338	0,036592	-0,16567	-0,2668	0,097822	0,530057	0,614161	0,111303	0,212971	0,007869	0,0628	0,093719	0,148915	0,142911	-2,52258	-1,93743	-1,72851	0,127744	0,2945
reactome_Organic_cation_transport_Main_Pathv	0,256499	0,495084	0,023326	-0,42934	0,19621	-0,73601	-1,50973	-1,51576	-1,29466	0,565867	1,254893	0,021353	1,01329	0,566151	0,365685	1,836373	2,142756	0,351871	0,126377	0,2945
Regulation_of_Angiogenesis_by_Interleukin-8	-0,19761	-0,14291	-0,23228	-0,14247	0,140675	-0,07472	-1,67693	-1,68923	-2,0238	0,270078	-0,31224	-0,35087	0,051518	0,129017	-0,47967	-0,53012	-0,55169	-0,37563	0,127023	0,2945
VEGF_Pathway_Cell_Survival	-0,3799	-0,25517	-0,26843	0,236688	0,665499	0,546393	0,560416	0,880811	0,249823	0,910492	0,189409	2,081477	0,100399	0,201926	0,625886	-3,43559	-2,1767	-1,65297	0,126773	0,2945
acyl_carrier_protein_metabolism	0,017651	0,014977	0,023092	0,262878	0,208982	0,192184	0,028452	-0,00188	-0,04021	0,040389	0,08282	0,071152	-0,04634	-0,11017	-0,11053	-0,38311	-0,3563	-0,36212	0,128363	0,295093
NCI_Glypican_1_network_Pathway_(BMP_signali	-0,19738	0,144514	-0,17398	-0,46524	-0,85281	-0,99581	-2,2505	-2,2944	-1,82314	1,765331	0,472363	-0,87823	-0,13835	0,462511	0,424741	0,369111	1,066802	0,352681	0,128833	0,295767
reactome_Beta_oxidation_of_lauroyl_CoA_to_de	0,098112	0,061908	0,049729	-0,21679	0,143379	0,090403	0,632574	0,639525	0,431672	0,698591	-0,14525	0,493722	0,290803	0,178269	0,1608	-2,52733	-1,99062	-2,22837	0,128846	0,295767
biocarta_fc_epsilon_receptor_i_signaling_in_mas	0,092194	0,35993	0,206648	-0,06269	0,609627	-0,85428	0,142758	-0,06686	0,304198	0,598417	-0,35356	-0,61156	-0,56309	-1,07104	-0,34642	0,195278	0,162593	0,129899	0,12914	0,296223
reactome_Activation_of_BID_and_translocation_	-0,1391	-0,09854	-0,02422	0,123757	0,589309	0,483436	-0,46021	-0,31589	-0,41412	-0,50188	0,322726	-0,10192	0,006988	-0,04242	-0,08276	-0,11381	-0,08513	0,245451	0,129304	0,29638
KEGG_Propanoate_metabolism_Main_Pathway	0,824094	0,214157	0,2559	0,336051	0,16226	0,035872	1,915456	2,653518	0,205602	0,264582	0,757515	1,969946	0,030289	0,476387	0,464465	-10,7133	-8,62261	-8,05908	0,129545	0,296715
NCI_ErbB_receptor_signaling_network_Main_Pat	-0,04759	0,108379	0,345247	-2,40763	-2,16405	-3,73317	-1,016	-1,01573	-0,73115	1,539029	-0,00287	-0,83433	0,386749	0,600152	0,909202	0,908398	0,15881	1,352567	0,129886	0,297275
reactome_Collagen_biosynthesis_and_modifying_	-1,02837	-0,71572	0,011165	-3,25202	-2,81436	-3,836	-1,69267	-3,13683	1,082162	-2,04794	-2,56552	-1,78404	0,647223	-0,10197	0,471907	5,473129	5,351938	3,789626	0,12998	0,297275
biocarta_nf_kb_signaling_Pathway_(Pathway_prc	0,164825	-0,06238	0,197789	-0,34085	-1,37618	-2,53447	-3,70714	-3,37215	-3,70848	1,576769	0,310124	1,214454	-0,29221	-0,74241	-0,93734	-1,74495	-1,66545	-1,57414	0,130784	0,298499
FLT3_Signaling_Pathway_Translation	-0,01277	-0,08401	-0,06703	0,282189	-1,00858	-0,5026	-0,98572	-0,89594	-1,26837	-2,17814	0,520855	0,232588	-0,31652	0,261323	0,327158	-2,24819	-2,40003	-2,14247	0,130803	0,298499
KEGG_Epstein_Barr_virus_infection_Main_Pathw	-0,17291	-0,64764	-0,24728	-1,14872	-0,41141	-1,11438	-1,44171	-1,03291	-2,14353	-0,85502	-1,33248	0,380669	1,291914	0,123817	1,59036	-1,66456	-2,60488	-0,83343	0,130762	0,298499
Role_of_Rho_GTPases_in_of_Actin_Cytoskeleton_	-0,76241	-0,42703	-0,70258	-0,83986	-3,69039	-1,18367	-3,27101	-3,85898	-2,51275	-2,00075	-2,5155	-1,3891	0,479673	0,522532	0,351107	-4,44964	-4,35202	-2,19298	0,130972	0,298666
NCI_Syndecan_2_mediated_signaling_events_Ma	0,008443	-0,42454	-0,50401	-1,23963	-1,85309	-0,66031	-2,22361	-2,22427	-2,42425	2,308186	3,354398	0,782025	-1,21335	-0,82764	-0,65948	-1,40973	-2,09384	-2,01112	0,131504	0,299659
purine_deoxyribonucleosides_salvage	-0,01864	-0,12318	-0,24417	0,022078	-0,3255	0,609814	-0,4747	-0,45925	-0,92133	0,959215	1,194474	0,905501	0,330971	0,251316	0,509815	-4,56644	-4,55162	-3,94733	0,131672	0,299823
Chemotaxis_Driven_by_CCR4	-0,13494	-0,51891	-0,5147	3,549574	3,159497	0,925819	-2,6446	-3,05482	-2,31243	0,534003	2,584948	1,978326	-0,15903	0,662867	0,282815	-1,49895	-1,92296	0,287423	0,131802	0,299899
NCI_Fanconi_anemia_Pathway_(regulation_of_dc	0,888072	-0,04481	-0,07694	1,629733	2,743058	1,518419	4,565409	5,92467	2,462418	0,464079	1,999881	1,260344	-1,41652	-0,8048	-0,45326	-11,4217	-7,98599	-5,97301	0,13225	0,300701
pyrimidine_ribonucleosides_degradation	-0,34098	-0,09045	0,007149	-0,08636	-0,01625	-0,82024	-1,2955	-1,45173	-1,01084	0,093604	-0,08277	-0,14017	-0,64997	-0,37297	-0,41679	0,243812	0,082961	-0,87307	0,132583	0,301236
Caspase_Cascade_Pathway_DNA_Fragmentation	0,122253	-0,04364	-0,08255	0,114606	-0,00846	-0,1615	-0,24879	-0,16134	-0,32109	0,225628	-0,43681	0,222641	0,241028	-0,1628	-0,18748	-1,48872	-1,24466	-0,92435	0,133086	0,301947
KEGG_Natural_killer_cell_mediated_cytotoxicity_	-0,87418	-0,25622	-0,67096	1,347636	4,966592	-0,19346	-4,91876	-5,07104	-3,94502	2,898447	3,132081	3,491117	-3,32635	-1,9157	-1,16779	2,482947	4,510036	6,223623	0,133089	0,301947
NCI_ErbB2_ErbB3_signaling_events_Pathway_(ac	0,07544	0,038417	-0,07116	-0,53321	0,190118	-0,12037	0,0058	0,138413	-0,26124	-0,02203	-0,12454	0,113286	0,06143	0,088513	0,16924	-1,95488	-1,23848	-1,02079	0,133286	0,302173
reactome_Regulation_of_cholesterol_biosynthesi	0,255457	-0,08977	-0,04199	-0,84761	-0,44491	-1,0545	-0,99454	-0,58862	-1,5565	-2,28389	-0,57899	-0,20312	-0,58016	-0,26516	0,005506	-5,40045	-3,70185	-2,9454	0,133821	0,303165
KEGG_Shigellosis_Main_Pathway	-0,09585	-0,57896	-0,50914	-1,06401	-2,38889	-1,67965	-0,94865	-0,69815	-1,6827	-3,96258	0,13254	0,472274	0,393633	0,086542	0,894095	-6,34143	-5,32537	-4,3601	0,134194	0,303789
UDP-iNi-acetyl-D-glucosamine_biosynthesis_II	0,186023	0,066745	0,068919	0,160514	-0,14342	0,197511	-0,27312	-0,10685	-0,51377	0,430353	-0,21349	0,206238	-0,14	0,321979	-0,01901	-1,72441	-1,35968	-1,22288	0,135404	0,306307
NCI_Trk_receptor_signaling_mediated_by_PI3K_a	-0,00963	0,015058	-0,01383	0,17246	0,06236	-0,12042	0,131449	0,125957	0,091831	0,107201	0,068105	-0,30615	0,002512	-0,1164	-0,01154	-0,48458	-0,28632	-0,30996	0,135575	0,30647
KEGG_Alcoholism_Main_Pathway	-0,49681	-0,74164	-0,5861	2,791966	3,898143	1,564837	-1,54207	-1,45055	-1,61482	-3,58861	1,775075	-0,56609	-0,30869	-0,31845	0,571487	0,210536	1,030096	2,189659	0,136571	0,308274
NCI_Ceramide_signaling_Pathway_(negative_regu	-0,01246	-0,36165	-0,2067	-0,92304	-0,62828	-1,17237	-0,95313	-1,04037	-0,94219	-0,88672	-0,46274	1,422666	-0,78225	0,03007	-0,68375	-0,64591	-0,52759	-0,5678	0,13654	0,308274
reactome_HS_GAG_biosynthesis_Main_Pathway	-1,07165	-0,8425	-1,02479	0,218513	-2,11071	-1,68399	-0,64466	-0,84823	-0,72197	-1,47478	-1,21611	-2,07806	1,353672	-0,6483	0,324194	-0,34198	-2,08679	-2,53755	0,136798	0,308563
PRPP_biosynthesis	0,016436	-0,0859	0,043131	0,566318	0,3005	0,388946	0,449124	0,422823	0,372391	-0,71559	1,058764	0,262405	0,083888	0,091281	0,115635	-0,41943	-0,31545	-0,19753	0,137007	0,308811
reactome_Endogenous_sterols_Main_Pathway	-0,56021	-0,24566	0,083171	1,164078	-0,47896	-0,35959	-2,23419	-3,04517	-1,72329	2,222236	-0,50156	0,100103	-0,04811	-1,06793	-0,06804	4,361724	0,894239	1,145887	0,137112	0,308823
NCI_CDC42_signaling_events_Pathway_(Pathway_	-0,24914	-0,14507	-0,30362	-0,32631	-0,2785	-0,32258	-1,25878	-1,1809	-1,14323	0,502656	0,304159	-0,10694	0,20643	0,240336	0,108388	0,827223	0,751165	0,490815	0,137238	0,308885
reactome_IRF3_mediated_induction_of_type_I_II	0,289467	0,242416	0,121676	0,390546	1,322836	0,226735	-0,73147	-0,59203	-1,23572	-0,50005	-0,42593	-0,565	-0,34979	-0,60141	-0,64566	-2,11499	-1,6343	-0,82663	0,137653	0,309594
reactome_Glyoxylate_metabolism_Main_Pathwa	0,000853	0,095178	0,089442	0,062051	-0,07603	-0,69278	0,407283	0,281664	0,723026	1,337387	-0,07752	1,116994	0,095711	0,161449	0,442545	1,035834	-0,12601	0,069593	0,137943	0,310023
reactome_Alpha_defensins_Main_Pathway	-0,12252	-0,02548	0,220486	0,147132	0,459988	0,492886	-0,39472	-0,3042	0,638308	-0,10348	0,19354	-0,0796	-0,45046	-0,55995	-0,33796	1,361485	0,888275	1,234156	0,138243	0,310473
reactome_Respiratory_electron_transport_Main_	1,576726	0,271537	0,226132	-3,03335	-4,95551	-4,68981	3,027069	4,220561	-0,17375	-5,6018	0,976442	-0,96916	-0,54101	-1,71997	0,702297	-39,2505	-34,217	-30,1484	0,138548	0,310933
NCI_CXCR4_mediated_signaling_events_Pathway_	-0,04147	-0,12762	-0,22718	0,244049	-1,1247	-0,6847	0,184293	0,177979	-0,34992	-0,30369	-0,64746	-0,51158	-0,63989	0,159264	0,518823	-3,57493	-2,09127	-1,5269	0,138733	0,310976
reactome_Cleavage_of_the_damaged_purine_Ma	-0,01997	0,067805	0,004594	0,548822	0,414784	-0,03601	-0,05762	-0,09353	0,074361	-0,68569	-0,22787	-0,42432	0,170342	-0,00422	-0,01639	0,316375	0,263772	0,185424	0,138866	0,310976
reactome_Recognition_and_association_of_DNA_	-0,01997	0,067805	0,004594	0,548822	0,414784	-0,03601	-0,05762	-0,09353	0,074361	-0,68569	-0,22787	-0,42432	0,170342	-0,00422	-0,01639	0,316375	0,263772	0,185424	0,138866	0,310976
heme_biosynthesis	0,074432	-0,10851	0,006638	-0,12671	-0,52696	-0,07247	-0,52965	-0,51411	-0,6031	-0,60826	-0,94658	-0,36905	-0,08627	-0,34855	0,015589	-0,29154	-0,70106	-1,01707	0,139066	0,3112
biocarta_spliceosomal_assembly_Main_Pathway	0,315095	-0,01332	0,1753	1,188404	0,380035	0,908997	0,363977	0,602912	0,073665	0,916599	-0,40336	1,266179	-0,2589	-0,1824	-0,24347	-3,33808	-2,3253	-2,76568	0,139632	0,312243
reactome_NOTCH2_Activation_and_Transmission	0,364977	0,01187	-0,047	-1,00533	-0,07339	-0,91832	0,622693	0,750276	0,48065	-1,70604	0,671236	-0,73195	0,639869	0,407306	0,403143	-4,13154	-1,87612	-1,62196	0,140012	0,312868
Ras_Pathway_Apoptosis	-0,67631	-0,54215	-0,56375	-1,37682	0,625678	0,678644	-0,08491	0,451887	-0,67285	1,942005	0,179101	0,758421	0,111807	0,26175	0,742896	-4,70359	-3,5048	-2,40099	0,140519	0,313776
UTP_and_CTP_dephosphorylation_I	0,076718	0,030239	0,022594	-0,3796	-0,31372	-0,52832	0,478336	0,514028	0,314613	-0,44133	-0,38959	0,177447	0,166738	0,33644	0,486477	-1,3774	-1,05478	-1,06953	0,140631	0,3138
reactome_Pyrimidine_salvage_reactions_Main_P	-0,15875	-0,09783	-0,16474	-0,42994	0,430042	-0,34052	-0,76176	-0,62271	-0,52189	-0,64563	-0,86784	-0,48478	-0,09339	-0,51803	-0,01638	-0,5537	-0,30879	-0,60399	0,141177	0,314793
KEGG_Mineral_absorption_Main_Pathway	0,010189	-0,04326	0,037383	-0,12514	-0,2211	0,011829	-0,14467	-0,2724	-0,17747	-0,13733	0,11854	-0,39224	-0,10328	-0,2112	0,009012	0,05142	0,224413	0,27757	0,141741	0,315736
NCI_Signaling_events_mediated_by_VEGFR1_and	0,081689	-0,41785	-0,4092	-0,72578	0,791978	0,680141	-4,14558	-3,95249	-4,67689	1,909394	0,599519	0,494202	0,187465	0,213535	0,28501	-4,88835	-3,42506	-3,05951	0,141904	0,315736
NCI_TCR_signaling_in_naive_CD8_T_cells_Main_F	-0,39346	0,515138	0,284516	-0,65161	0,186774	1,502173	2,513411	2,627111	3,643577	0,595748	-1,36517	2,391037	-1,41145	-1,67837	-0,63296	1,19418	0,344373	0,806958	0,141864	0,315736
Akt_Signaling_Pathway_NFAT_degradation	-0,39812	-0,51711	0,020323	-0,36068	1,052592	-0,69715	-0,0718	0,120265	-0,22829	1,014173	0,24555	0,701444	0,534835	0,639236	0,615832	-3,11745	-1,528	-0,78786	0,142223	0,315925
Cell_Adhesion_Regulated_by_CDHs	-0,31447	-0,09586	-0,61415	-1,08386	-2,66316	-1,92803	-3,93179	-3,86498	-4,15917	-1,6582	0,448329	-0,79671	-0,09861	0,310026	0,703743	-8,36208	-7,53286	-7,19308	0,142293	0,315925
KEGG_Tryptophan_metabolism_Main_Pathway	-0,0935	-0,20918	0,396574	-1,49121	-1,80643	-1,72254	-1,61303	-2,43053	-0,89144	-0,98121	-2,21438	-1,52346	0,541691	-0,63861	-0,28852	1,404613	1,607704	2,588483	0,142232	0,315925
NCI_CXCR4_mediated_signaling_events_Pathway_	-0,23234	0,001709	-0,14741	-0,18053	-0,7481	-0,2024	-0,24751	-0,12226	-0,3732	-0,1474	-0,6022	-0,04529	-0,24118	0,223545	0,294822	-2,77546	-1,1753	-0,87837	0,142803	0,31638
NCI_IL8_and_CXCR2_mediated_signaling_events_	-0,25812	-0,31922	-0,10799	-0,47198	0,124186	-1,42539	-2,80319	-3,00826	-2,17517	0,503243	0,199557	-1,29036	-0,8026	-0,50642	0,3363	-0,22464	1,287698	1,592598	0,14275	0,31638
NCI_Internalization_of_ErbB1_Main_Pathway	0,246732	-0,59924	-0,72489	-1,89017	-1,16301	-2,31523	-2,66977	-1,7217	-4,16865	-4,06309	0,893972	-0,39888	-0,85773	-0,60855	-0,06502	-13,091	-9,96995	-8,02284	0,142657	0,31638
NCI_Plasma_membrane_estrogen_receptor_signa	-0,09875	-0,13949	-0,01532	0,368077	0,455984	0,295053	-0,37434	-0,48171	-0,39122	-0,04126	0,131578	0,030536	0,009602	-0,07086	-0,07855	-0,02268	0,028366	0,060989	0,143015	0,316625
KEGG_Non_alcoholic_fatty_liver_disease_NAFLD_	0,149114	-0,06115	-0,26529	-1,37156	-0,97208	0,051986	-1,74436	-1,00745	-2,45958	-0,43729	-1,45981	0,230704	0,29879	0,139439	-0,07356	-5,50046	-3,51053	-2,163	0,14327	0,316964
IP3_Pathway_Gene_Expression_via_CREB3_NFAT	0,29874	-0,20321	-0,18141	1,216094	1,286294	1,476769	-1,89134	-1,52409	-2,54241	-3,20411	0,593273	-1,97438	-0,3932	-0,68897	0,323919	-0,79797	0,451237	1,378259	0,14351	0,317143
NCI_PLK1_signaling_events_Pathway_(chromosor	0,036779	0,018355	-0,07069	-0,10383	-0,39212	-0,39398	0,604786	0,662168	0,476789	-0,72516	-0,90078	-0,40679	-0,13054	-0,00585	0,047866	-0,55743	-0,49418	-0,25832	0,143563	0,317143
reactome_Nitric_oxide_stimulates_guanylate_cyc	-0,04133	-0,04184	0,009847	-0,78071	-0,59646	0,086226	0,149614	-0,07113	-0,03034	-0,59693	0,262733	-0,03892	-0,28303	-0,39578	-0,35617	0,044214	-0,47324	-0,43273	0,143758	0,317143
reactome_Role_of_Abl_in_Robo_Slit_signaling_M	-0,03368	-0,05203	-0,06484	0,0072	-0,44926	-1,11642	-2,86749	-2,67679	-2,96313	-0,55313	-0,3693	-0,96301	-0,28862	0,232851	-0,02241	-2,08519	-1,38238	-1,28995	0,143664	0,317143
biocarta_role_of_mal_in_rho_mediated_activatio	-0,04944	-0,3435	-0,43151	0,382267	0,542188	-0,95376	-0,54125	-0,2086	-0,90076	-1,12539	0,753774	-0,49969	-0,0372	-0,06129	-0,10497	-1,80287	-1,22836	-0,20858	0,144331	0,317163
Caspase_Cascade_Pathway_Activated_Tissue_Tra	-0,89498	-0,38749	-0,47984	0,124169	-0,15541	-0,11521	-0,9698	-1,3937	0,755955	1,154434	2,032754	-3,42845	0,593322	-0,10141	-0,35597	8,664748	7,332935	6,670394	0,144122	0,317163
Cellular_Apoptosis_Pathway_DNA_Fragmentatior	-0,03682	-0,11868	-0,09972	0,006235	0,022657	-0,87219	-0,07196	-0,14632	0,020619	-0,38098	-0,54492	-0,33153	0,618094	0,114379	0,455046	-0,31206	-1,01413	-0,10322	0,143999	0,317163
Mitochondrial_Apoptosis_Pathway_Gene_Expres	0,192926	-0,06261	-0,02165	0,032822	-0,1639	0,001791	-0,0831	0,16123	-0,45618	-0,09241	0,521678	0,140762	0,133025	-0,00573	0,245153	-1,69006	-1,70072	-1,04067	0,144006	0,317163
NCI_ATF_2_transcription_factor_network_Main_	0,035502	-0,31119	-0,2888	-0,40116	-0,24367	-1,21867	-0,99632	-0,47468	-1,71677	-0,30214	0,698021	-0,95716	0,353698	0,430572	0,624387	-2,92004	-1,78336	-1,24545	0,14448	0,317163
NCI_Cellular_roles_of_Anthrax_toxin_Pathway_(r	-0,0916	-0,08439	-0,10295	-0,54144	-0,31195	-0,50374	-0,275	-0,09436	-0,31976	0,167679	-0,22369	-0,57068	0,471471	0,436378	0,357914	-1,093	-0,87445	-0,76789	0,144286	0,317163
sucrose_degradation	-0,05387	0,088762	0,121302	0,620334	0,505024	-0,5572	0,774741	0,61986	0,686734	1,846927	-0,11038	1,07263	-0,06927	0,207611	-0,21938	0,854268	0,250113	0,611208	0,144415	0,317163
reactome_Switching_of_origins_to_a_post_replic	0,296229	0,20164	0,237263	0,648276	0,011318	0,174634	1,940386	2,017272	1,580751	-0,44004	-0,40896	-0,51328	-0,35739	-0,46524	-0,29208	-1,41901	-1,0948	-1,05241	0,144811	0,317667
biocarta_il_7_signal_transduction_Pathway_(cell_	0,034459	0,121336	0,075791	0,077531	0,489551	0,226778	0,234072	0,262704	0,52531	0,226865	0,639571	0,275891	0,124998	0,210518	0,279884	1,322661	1,077123	0,70008	0,144923	0,317689
Hedgehog_Signaling_in_Mammals_no_Tubulin_P	0,355534	-0,23738	-0,34699	2,114371	1,200339	1,94575	1,430403	0,837943	1,149805	-0,63689	1,804118	1,665286	0,248958	-1,00102	-0,59041	-2,55259	-4,15685	-1,6329	0,145731	0,318562
NCI_TGF_beta_receptor_signaling_Main_Pathwa	-0,11531	-0,49219	-0,36124	0,868516	2,029188	0,934831	3,097241	2,959403	2,506279	0,532026	1,792517	1,594882	0,006399	-0,28425	0,448027	-1,26378	-1,56645	-1,62905	0,145604	0,318562
reactome_Dermatan_sulfate_biosynthesis_Main_	-0,33041	-0,15055	-0,21053	-0,7086	-0,11871	-0,06004	2,848428	2,872374	2,649186	-0,26975	-0,73089	0,371274	0,126224	0,145797	0,736618	4,199931	4,162572	3,799605	0,145706	0,318562
reactome_EGFR_downregulation_Main_Pathway	0,111444	-0,45621	-0,4634	-0,2459	-0,03675	-1,4832	0,510441	0,883936	-0,41376	-2,83073	1,013691	1,209774	-0,18284	-0,10292	0,223075	-6,60218	-4,47665	-3,68258	0,145561	0,318562
Regulation_of_Cytoskeleton_Remodeling_by_TGF	-1,06768	-1,76509	-1,58414	-4,01738	-3,88075	-5,29327	-9,30437	-9,24146	-10,0003	-3,5596	-2,31634	-0,17558	2,390424	2,460403	1,944977	-12,8843	-7,55418	-7,3992	0,146091	0,319125
NCI_Signaling_events_mediated_by_Stem_cell_fa	-0,00368	0,091422	0,069299	-0,54848	-0,46777	-0,19635	-0,48834	-0,60879	-0,5901	-0,48254	-0,47104	-0,53787	-0,11685	-0,11146	-0,10838	-1,33785	-1,79772	-1,29408	0,146348	0,319462
reactome_ATF6_alpha_activates_chaperones_Ma	0,054893	-0,017	-0,09661	0,063755	0,044316	-0,23671	-0,40605	-0,31837	-0,60914	-0,80195	0,316259	0,419482	0,076448	-0,07115	-0,064	-1,56695	-1,42015	-0,74674	0,146858	0,320352
NCI_CDC42_signaling_events_Pathway_(neuron_	-0,25169	-0,12658	-0,32012	-0,87858	-0,65155	-0,44634	-1,05166	-0,99867	-0,991	0,209473	0,132497	-0,44894	0,144253	0,197797	0,14156	0,653363	0,890963	0,492825	0,147085	0,320399
reactome_SUMO_is_proteolytically_processed_M	0,299911	0,038965	-0,02671	0,000996	-0,00312	0,154702	0,178986	0,373967	-0,19705	0,046553	0,518963	0,483542	-0,08437	0,404491	0,109955	-3,34427	-2,60949	-1,56243	0,14702	0,320399

reactome_SHC1_events_in_EGFR_signaling_Main	0,046494	-0,16185	-0,15067	-0,72028	-0,5169	-0,78473	-0,11746	-0,04257	-0,39341	-1,21365	0,18496	-0,86549	-0,08979	-0,15592	0,076124	-2,80521	-1,87327	-1,6875	0,147447	0,320963
reactome_Downregulation_of_TGF_beta_recepto	0,050498	-0,1909	-0,1804	-1,45874	-1,74796	-2,63592	-3,29273	-3,09879	-4,27554	-2,50583	-1,4937	-0,69883	0,608982	0,487663	0,798267	-6,56117	-5,07631	-2,8975	0,147596	0,321062
KEGG_Circadian_rhythm_Main_Pathway	-0,00845	-0,01418	0,076072	-0,35576	-0,03448	0,606871	0,50373	0,332852	0,33342	-0,07032	-0,12646	-0,61689	0,096055	0,644216	-0,11787	-0,18016	-0,19471	-0,23059	0,147731	0,321133
reactome_Assembly_of_the_RAD51_ssDNA_nucle	0,004455	-0,05059	-0,06407	-0,1028	0,127673	0,433114	0,933821	1,094969	0,907534	1,210292	-1,09237	0,047025	0,21979	0,425826	0,352916	-0,46913	-0,17451	-0,29257	0,148162	0,321619
reactome_The_canonical_retinoid_cycle_in_rods_	-0,11342	0,116986	0,405731	-0,2035	0,761936	-0,07481	1,080878	0,784694	2,083664	0,983246	-1,33824	0,002597	0,408803	0,164642	0,026576	4,167015	3,856185	3,147061	0,148161	0,321619
ATM_Pathway_Cell_Cycle_Checkpoint_Control	-0,00379	-0,0634	-0,09178	0,290489	0,594585	0,571485	-0,47887	-0,3661	-0,64127	0,131357	0,407165	0,276164	0,024778	-0,08526	-0,16186	-0,77457	-0,41875	-0,11444	0,148446	0,322012
Akt_Signaling_Pathway_NF-kB_pathway	0,129439	-0,20504	0,312429	-1,19451	0,156676	-1,124	0,215008	0,324593	0,277129	0,705922	1,060824	-0,89486	0,512493	0,271906	0,202842	0,419744	0,884193	1,17899	0,14865	0,322164
reactome_Cholesterol_biosynthesis_Main_Pathw	0,529328	0,345353	0,039604	1,919904	1,789472	2,776515	2,285289	2,709142	1,798853	0,5715	2,039572	1,880357	-0,57335	-0,42712	-0,66637	-6,68452	-4,40181	-3,86414	0,148723	0,322164
NCI_Aurora_A_signaling_Pathway_(regulation_of	-0,00975	-0,02606	-0,12287	-0,19045	-0,28534	0,093971	0,762133	0,811736	0,666111	-1,19918	-1,05684	-0,90756	-0,31437	-0,19292	-0,05691	-0,27914	-0,04495	-0,20125	0,148836	0,322186
heparan_sulfate_biosynthesis_late_stages	-0,70712	-0,40079	-0,73698	0,960139	-2,50701	-0,96428	-1,12315	-0,93945	-1,16447	-0,49683	-0,02097	-0,8496	0,998854	-0,03551	1,028468	-1,89178	-2,68969	-2,54161	0,14903	0,32238
Akt_Signaling_Pathway_Synaptic_Transmission	-0,55572	-0,06447	0,483447	-2,85904	-3,35683	-3,49453	-2,21984	-2,41341	-1,9409	1,943826	1,593165	-0,76401	2,904837	2,321885	2,554701	6,009597	5,767568	5,270834	0,149408	0,322402
Rac1_Signaling	0,019946	-0,2513	-0,24702	-0,19512	-1,58671	-0,72052	-0,86102	-0,78222	-1,34649	-1,98111	-0,04846	0,407003	-0,32275	-0,29078	0,389716	-5,33273	-4,06538	-3,08475	0,149514	0,322402
reactome_Hyaluronan_biosynthesis_and_export_	-0,11616	-0,10748	-0,02174	-1,35651	-1,18131	-0,86075	-0,75389	-0,71984	-0,74447	-0,83279	-0,79526	-0,54618	0,191004	0,121435	0,334264	1,616582	1,616349	1,40086	0,149557	0,322402
reactome_The_fatty_acid_cycling_model_Main_F	-0,11051	-0,01658	-0,00299	0,025619	-0,55758	-0,07717	-0,11461	-0,31492	0,227161	1,058376	0,329548	-0,01144	0,386892	-0,09767	-0,01242	0,252217	0,374685	0,019788	0,149526	0,322402
reactome_The_proton_buffering_model_Main_P	-0,11051	-0,01658	-0,00299	0,025619	-0,55758	-0,07717	-0,11461	-0,31492	0,227161	1,058376	0,329548	-0,01144	0,386892	-0,09767	-0,01242	0,252217	0,374685	0,019788	0,149526	0,322402
NCI_Alternative_NF_kappaB_Main_Pathway	-0,12288	-0,05543	0,04437	0,418586	0,155887	0,636835	-0,09228	-0,14307	-0,17068	0,222758	0,159527	-0,8147	0,355035	0,197487	0,262733	0,412496	0,347434	0,663023	0,149842	0,322569
NCI_Alternative_NF_kappaB_Pathway_(regulatior	-0,12288	-0,05543	0,04437	0,418586	0,155887	0,636835	-0,09228	-0,14307	-0,17068	0,222758	0,159527	-0,8147	0,355035	0,197487	0,262733	0,412496	0,347434	0,663023	0,149842	0,322569
NCI_Nephrin_Neph1_signaling_in_the_kidney_po	0,458411	0,49309	0,216338	-0,57603	-0,93911	-0,05892	0,810096	1,170675	-0,10041	0,038816	-0,68855	-0,40849	0,147609	0,081345	0,330648	-5,4495	-3,63946	-3,12946	0,150266	0,323035
reactome_Glutamate_Neurotransmitter_Release_	-0,14742	-0,28281	-0,17347	-1,09028	-1,4998	-2,29134	-2,88463	-2,92194	-3,0891	-2,48186	-2,44819	-2,22011	-0,20302	-0,57753	0,086098	6,107524	5,349251	5,97608	0,150259	0,323035
Akt_Signaling_Pathway_AR_mediated_apoptosis	0,056168	-0,12277	0,310806	-1,10351	0,444881	-1,42866	0,040691	0,017982	0,220743	0,771645	1,875064	0,051848	0,547804	0,476529	0,100758	-0,1277	0,805807	0,883112	0,1505	0,323092
KEGG_Arachidonic_acid_metabolism_Main_Pathv	-0,78706	0,787769	0,579076	1,094406	-2,79145	-0,6145	-0,86249	-1,54949	2,059584	0,387391	-0,26697	3,694206	-0,17253	1,860385	0,645187	3,85643	2,633291	1,224304	0,150427	0,323092
NCI_ATR_signaling_Pathway_(response_to_G2_M	0,709282	0,148968	0,120988	0,672875	0,387428	-0,06191	1,65765	2,547863	0,583786	1,780286	0,308221	0,402776	-0,29181	0,039173	0,453755	-8,60419	-6,22105	-4,53891	0,15066	0,323214
biocarta_the_igf_1_receptor_and_longevity_Path	-0,00489	-0,06334	-0,11899	-0,36451	0,455996	0,384107	-0,3084	-0,12304	-0,51028	0,078573	0,850435	0,290568	0,186148	-0,22213	-0,00506	-2,13205	-2,008	-1,71275	0,151893	0,324444
biocarta_the_igf_1_receptor_and_longevity_Path	-0,00489	-0,06334	-0,11899	-0,36451	0,455996	0,384107	-0,3084	-0,12304	-0,51028	0,078573	0,850435	0,290568	0,186148	-0,22213	-0,00506	-2,13205	-2,008	-1,71275	0,151893	0,324444
ILK_Signaling_Pathway_Opsonization	-0,06758	-0,05571	-0,09406	-0,77835	-0,91945	-0,86351	-0,65758	-0,56164	-0,72737	0,497909	0,161261	0,707711	-0,0447	0,0093	0,083864	-0,67885	-0,3606	-0,4547	0,151693	0,324444
NCI_Validated_transcriptional_targets_of_deltaNp	0,406492	-0,05135	0,195344	0,109558	0,356505	-0,01512	0,600152	0,581942	0,480907	0,567704	1,081655	-0,30519	0,345735	0,608482	0,757963	-1,10612	-0,97043	-0,21555	0,151963	0,324444
PTEN_Pathway_Synaptic_Transmission	-1,00008	-0,44823	-0,55813	-0,76406	-1,27147	-0,04255	8,015228	7,702723	8,684655	-1,50624	-3,66755	-2,4318	-0,38152	-1,17833	-1,32512	11,26212	11,54496	9,133756	0,151397	0,324444
reactome_Budding_and_maturation_of_HIV_viric	0,307447	-0,25608	-0,11067	-2,05917	-1,41132	-0,70478	0,697037	1,006702	0,025721	-0,89523	-0,98763	-1,44906	-0,36691	-0,47866	0,229508	-7,83383	-6,12093	-4,35604	0,151933	0,324444
reactome_DARPP_32_events_Main_Pathway	0,097587	-0,24492	-0,08059	-0,67544	-0,08868	-0,14756	-1,10318	-0,72674	-1,48072	-2,56891	0,495614	-0,01605	-0,91604	-0,65589	-0,21319	-4,18088	-3,9672	-2,60104	0,151649	0,324444
biocarta_chaperones_modulate_interferon_signa	0,111691	0,020317	0,027588	-0,11721	-0,23345	0,471327	-0,02131	0,069219	-0,24508	0,039803	-0,01368	-0,4686	-0,08758	-0,18814	0,262776	-1,35739	-0,7449	-0,19819	0,15224	0,324751
biocarta_rac1_cell_motility_signaling_Pathway_(p	-0,25628	-0,16256	0,059602	-0,38569	-0,54703	0,155884	-0,89107	-1,35829	-0,8137	0,74922	0,231863	1,383212	0,46888	0,52018	0,362747	2,080626	0,448235	0,620219	0,152419	0,324751
reactome_Dopamine_receptors_Main_Pathway	-0,31785	-0,04161	-0,17632	-0,17171	-0,05392	-0,40262	0,077177	-0,0223	0,458108	0,0313	0,286523	0,207942	0,352231	0,358197	0,298464	1,490106	1,884529	1,458318	0,152401	0,324751
ATM_Pathway_S-phase_arrest	0,088571	-3,95E-05	0,001444	0,351852	0,438027	0,382865	-0,14104	0,040892	-0,32427	-0,19022	0,508823	-0,02814	-0,21381	-0,2028	-0,13334	-1,04542	-0,75117	-0,4211	0,153353	0,325619
NCI_Lissencephaly_gene_LIS1_in_neuronal_migra	0,082691	-0,08027	-0,05572	0,174861	0,037938	-0,05859	-0,21214	-0,0706	-0,42869	0,414243	0,139792	-0,08431	0,021936	-0,02135	0,075063	-0,82373	-0,96843	-0,48041	0,15307	0,325619
reactome_Dual_incision_reaction_in_GG_NER_M	0,657558	-0,14289	-0,28266	-0,58444	0,64598	0,822506	-0,88061	0,15764	-1,61029	0,889797	0,637579	2,161194	0,131785	0,359498	0,209765	-6,38574	-6,35246	-4,87191	0,153294	0,325619
reactome_Fanconi_Anemia_Main_Pathway	0,665582	0,098852	0,128853	0,307532	1,287157	0,115205	2,875042	3,302105	1,899227	-1,06336	0,631197	-0,54595	-0,86484	-0,65763	-0,64496	-4,84398	-3,52667	-2,32793	0,152976	0,325619
reactome_Formation_of_incision_complex_in_GC	0,657558	-0,14289	-0,28266	-0,58444	0,64598	0,822506	-0,88061	0,15764	-1,61029	0,889797	0,637579	2,161194	0,131785	0,359498	0,209765	-6,38574	-6,35246	-4,87191	0,153294	0,325619
reactome_Pyruvate_metabolism_Main_Pathway	0,342534	0,172329	0,10158	-0,35212	-0,04147	-1,44627	0,446356	0,636348	0,100028	-1,16281	-0,88519	-0,1541	-0,11066	-0,19296	-0,20745	-4,4091	-3,66738	-2,64501	0,153454	0,325619
Interferon_Pathway	1,399882	-0,81693	0,13091	-1,161	1,857382	-3,53923	3,079937	4,130779	1,104081	2,698673	4,179266	1,822897	3,573218	3,398159	7,075194	-10,1347	-7,17284	-6,3604	0,153718	0,325958
KEGG_Measles_Main_Pathway	0,271769	-0,27137	-0,07695	-0,93582	1,917692	-0,04925	-1,94991	-1,68296	-2,56119	-0,11768	0,249523	-0,09051	0,117421	-0,28667	0,184943	-1,65688	-3,17171	-1,14501	0,153925	0,326174
reactome_alpha_linolenic_acid_ALA_metabolism_	0,188622	-0,00596	0,134163	-0,82351	-0,44339	-0,41623	0,237967	0,366331	-0,39465	1,94534	1,597774	1,635893	-1,09335	-0,9043	-0,78389	-2,63674	-1,34067	-0,91993	0,154575	0,326663
reactome_Beta_oxidation_of_decanoyl_CoA_to_(	0,131647	-0,00907	0,035917	0,099857	0,406845	-0,18356	0,803379	0,978253	0,544474	0,559042	0,117271	0,475032	-0,03358	0,00993	-0,17692	-3,15827	-2,69813	-2,55993	0,154554	0,326663
reactome_Beta_oxidation_of_octanoyl_CoA_to_h	0,131647	-0,00907	0,035917	0,099857	0,406845	-0,18356	0,803379	0,978253	0,544474	0,559042	0,117271	0,475032	-0,03358	0,00993	-0,17692	-3,15827	-2,69813	-2,55993	0,154554	0,326663
reactome_Vasopressin_like_receptors_Main_Path	-0,19746	0,113484	0,140668	0,216346	0,458653	-0,23872	-0,01354	-0,1241	0,371233	0,42667	1,013849	-0,6416	-0,05655	0,156213	0,260746	1,329329	1,15052	1,804632	0,154268	0,326663
NCI_Integrin_linked_kinase_signaling_Pathway_(r	0,037003	0,052532	0,013897	0,033479	-0,13245	-0,13521	-0,546	-0,52091	-0,69081	-0,67646	0,074867	-0,13339	-0,14323	-0,12299	-0,06843	-0,89819	-0,51774	-0,62578	0,154684	0,326672
biocarta_the_igf_1_receptor_and_longevity_Mair	0,050951	-0,20726	-0,25669	-0,22253	0,448963	0,003678	-1,49436	-1,15235	-1,95732	0,756158	0,677845	0,626855	0,348374	-0,14797	0,136905	-3,89755	-2,99698	-2,19764	0,155708	0,328612
NCI_PLK2_and_PLK4_events_Main_Pathway	0,129823	0,039757	-0,0146	-0,49326	-0,5027	-0,40061	-1,18231	-1,05615	-1,39043	-0,37297	-0,12136	-0,16771	0,443967	0,533188	0,593374	-1,28652	-1,0046	-0,79121	0,155935	0,328645
NCI_PLK2_and_PLK4_events_Pathway_(regulatior	0,129823	0,039757	-0,0146	-0,49326	-0,5027	-0,40061	-1,18231	-1,05615	-1,39043	-0,37297	-0,12136	-0,16771	0,443967	0,533188	0,593374	-1,28652	-1,0046	-0,79121	0,155935	0,328645
KEGG_Valine_leucine_and_isoleucine_biosynthes	-0,09654	-0,13291	-0,10688	-0,01825	-0,21018	-0,28229	-0,62349	-0,62748	-0,5737	0,502743	0,429437	-0,45313	-0,13378	0,143209	0,248009	0,756844	0,309411	0,588577	0,156178	0,328935
KEGG_NF_kappa_B_signaling_Main_Pathway	-0,34733	-0,57599	-0,10751	-2,25526	-1,26063	-4,04186	-3,59931	-3,64238	-2,44648	2,97213	-1,70747	3,719406	0,569841	-0,71075	-0,07378	-0,70345	-1,43484	-0,82567	0,156725	0,329337
NCI_PDGFR_beta_signaling_Pathway_(cell_migrat	-0,18008	-0,24091	-0,27585	-0,2922	0,345281	-0,04511	-0,15152	-0,11351	-0,15583	-0,27961	0,410977	-0,62642	0,07869	0,036029	0,156256	-0,25037	0,083066	0,016992	0,156687	0,329337
reactome_EGFR_interacts_with_phospholipase_C	0,026592	-0,03567	-0,01258	-0,07523	-0,18636	-0,57503	0,618961	0,704113	0,494984	-0,39201	-0,12463	-0,605	-0,01285	-0,23371	0,120132	-1,15974	-0,78552	-0,63993	0,156747	0,329337
reactome_Presynaptic_function_of_Kainate_rece	-0,11945	-0,21758	-0,24285	2,242951	3,00216	1,282011	0,469486	0,314218	0,433272	-1,53262	1,607489	1,869544	-0,36409	-0,13237	0,139989	-0,8703	-1,04387	0,380107	0,156792	0,329337
CCR3_Pathway_in_Eosinophils	0,094802	-0,28699	-0,21204	3,030786	1,518059	0,212363	-0,5881	-0,88888	-0,64991	-3,08324	2,639875	0,450515	-1,95524	-0,91839	-0,67716	-6,02133	-4,91871	-2,96664	0,157655	0,330481
reactome_Antigen_Presentation_Folding_assemb	0,655531	0,091289	0,182448	-0,84284	-0,61509	-1,16285	-2,96939	-2,58032	-3,74574	0,724438	2,591261	0,541222	0,906507	1,058735	1,38407	-12,4768	-10,0661	-8,12116	0,157482	0,330481
reactome_Prostacyclin_signalling_through_prosta	-0,07351	-0,04127	-0,19291	1,374168	1,118671	0,409589	0,038859	-0,24759	0,098077	-1,19414	0,927718	0,961665	-0,26209	-0,19195	-0,08462	-1,12857	-1,15757	0,034578	0,15764	0,330481
KEGG_Dopaminergic_synapse_Main_Pathway	-0,62425	-0,5115	-0,43269	3,442745	4,792519	-0,46889	-1,80537	-1,44609	-2,16478	-1,30862	0,88628	0,604029	1,190393	0,178493	2,375039	0,859329	1,556295	3,146294	0,158055	0,330814
NCI_Fanconi_anemia_Pathway_(DNA_repair)	0,200464	0,100584	0,099074	0,710585	0,279246	0,946859	0,412411	0,536014	0,177327	-0,61402	0,272546	-0,00608	-0,32904	0,004971	-0,09919	-0,75974	-0,3287	0,028116	0,158132	0,330814
reactome_ADP_signalling_through_P2Y_purinoce	0,082898	-0,31152	-0,44452	1,068098	0,588688	-0,0499	0,431962	0,291717	0,084488	-2,34316	1,016147	0,707619	-0,99581	-0,65396	-0,65665	-3,65414	-3,07305	-1,85009	0,157925	0,330814
NCI_TCR_signaling_in_naive_CD8_T_cells_Pathwa	0,001925	0,146448	0,000952	-0,12829	-0,60939	-0,43996	0,604206	0,676042	0,935677	-0,44863	-0,16793	-0,4572	-0,2739	-0,37592	-0,12464	0,030096	-0,54068	0,082227	0,158469	0,331073
reactome_temp_Post-translational_modification_	-1,12339	0,479286	0,871222	-1,53124	1,844127	-0,54697	-1,10084	-2,46331	0,778865	-7,13281	-0,58499	0,387017	-1,39053	-1,86095	-0,93338	9,663837	9,550499	9,122257	0,158443	0,331073
NCI_ErbB4_signaling_events_Pathway_(neural_cr	-0,11767	-0,00855	-0,02921	-1,27025	-1,03881	-1,62865	0,501106	0,470305	0,392731	0,207799	-0,19115	0,052516	0,300182	0,284276	0,221893	0,255269	0,116523	0,433981	0,159131	0,331346
NCI_Plasma_membrane_estrogen_receptor_signa	-0,01239	-0,0052	-0,04381	-0,15188	-0,3626	-0,40512	-0,31745	-0,22607	-0,34263	0,206129	-0,09136	-0,50323	0,105027	0,208221	0,182002	-1,2407	-0,98032	-0,68028	0,158931	0,331346
NCI_Plasma_membrane_estrogen_receptor_signa	-0,01239	-0,0052	-0,04381	-0,15188	-0,3626	-0,40512	-0,31745	-0,22607	-0,34263	0,206129	-0,09136	-0,50323	0,105027	0,208221	0,182002	-1,2407	-0,98032	-0,68028	0,158931	0,331346
NCI_Plasma_membrane_estrogen_receptor_signa	-0,01239	-0,0052	-0,04381	-0,15188	-0,3626	-0,40512	-0,31745	-0,22607	-0,34263	0,206129	-0,09136	-0,50323	0,105027	0,208221	0,182002	-1,2407	-0,98032	-0,68028	0,158931	0,331346
reactome_Glucose_transport_Main_Pathway	-0,27557	-0,09995	-0,08938	-1,40625	-1,21279	-2,05814	0,58719	0,239213	0,752112	-0,64414	1,028365	1,00925	0,28638	0,49072	0,60295	0,81906	0,077476	0,461975	0,159052	0,331346
NCI_Beta3_integrin_cell_surface_interactions_Ma	-0,91928	-0,61711	-0,31033	-2,61758	-3,47383	-2,50679	-7,22132	-7,34164	-6,41432	1,835289	0,669981	-0,09571	1,309698	0,779703	1,490558	2,586611	2,451861	1,293079	0,159753	0,332197
pyrimidine_deoxyribonucleotides_ide_novoi_bios	0,244392	0,051762	-0,14844	-0,24719	-0,16296	0,042507	1,309423	1,439863	0,502155	0,42274	2,43464	0,948884	0,749806	1,021776	1,168331	-5,02397	-3,85845	-3,20303	0,15965	0,332197
RANK_Signaling_in_Osteoclasts_Pathway_Inhibiti	-0,3922	-0,64317	-0,4185	-0,60447	-0,24715	-0,23546	-1,16563	-0,44343	-1,76202	0,087498	-0,21558	1,975437	0,001404	0,089384	0,591028	-4,4818	-3,64014	-2,40892	0,159986	0,332458
biocarta_how_does_salmonella_hijack_a_cell_Ma	-0,00272	-0,20053	-0,24123	-0,32439	-1,23479	-0,12434	-0,95241	-0,69195	-1,37216	-0,93423	0,249324	-0,65377	-0,57276	-0,33192	-0,16228	-4,52913	-3,78485	-2,39207	0,160555	0,332532
biocarta_how_does_salmonella_hijack_a_cell_Pat	-0,00272	-0,20053	-0,24123	-0,32439	-1,23479	-0,12434	-0,95241	-0,69195	-1,37216	-0,93423	0,249324	-0,65377	-0,57276	-0,33192	-0,16228	-4,52913	-3,78485	-2,39207	0,160555	0,332532
NCI_Plexin_D1_Signaling_Pathway_(focal_adhesic	-0,07835	-0,11635	-0,11676	0,05598	0,068789	0,014747	-0,29946	-0,21934	-0,21079	-0,40716	-0,72154	-1,20419	0,236381	0,421889	0,35116	0,5332	0,571886	0,4056	0,16053	0,332532
NCI_Plexin_D1_Signaling_Pathway_(regulation_of	-0,07835	-0,11635	-0,11676	0,05598	0,068789	0,014747	-0,29946	-0,21934	-0,21079	-0,40716	-0,72154	-1,20419	0,236381	0,421889	0,35116	0,5332	0,571886	0,4056	0,16053	0,332532
Neuronal_Development_Induced_by_CDK5	-0,44798	-0,86513	-0,51588	-2,63193	-4,74945	-1,51491	-7,08057	-6,8355	-8,48555	-4,20462	-0,62197	-0,70797	-0,88613	-1,22717	0,891193	-18,5105	-11,3957	-9,3298	0,160243	0,332532
reactome_Regulation_of_signaling_by_NODAL_M	-0,04832	-0,03304	0,090679	-0,16333	-0,2609	-0,67662	0,572781	0,517676	0,989915	-0,65675	-0,35014	-0,22454	0,224988	-0,18023	0,233722	1,640795	1,478534	1,11692	0,160814	0,332848
cAMP_Pathway_Axonal_Growth	0,125226	0,142483	0,027402	0,142634	-0,68997	-0,95788	-1,57471	-1,38836	-1,80004	-1,63505	-1,16185	-0,76974	-0,27848	-0,09775	0,259463	-2,48759	-1,24677	-0,24751	0,161343	0,33316
KEGG_MicroRNAs_in_cancer_Main_Pathway	0,869106	2,180588	3,593383	5,254203	7,797126	5,650673	2,132258	2,438616	6,608363	5,757505	2,270461	0,559288	-3,44289	-4,6388	-3,96579	22,5722	12,84488	4,134161	0,16118	0,33316
KEGG_Primary_bile_acid_biosynthesis_Main_Patl	-0,10396	0,257604	0,323687	-2,7132	-0,39344	-1,20235	-0,80056	-1,32448	-0,55323	-0,60414	0,104785	-0,07917	0,155744	-0,41792	0,361993	0,468504	0,028579	0,147236	0,161393	0,33316
NCI_Regulation_of_Androgen_receptor_activity_	-0,10583	-0,06756	-0,25927	0,33731	-0,25722	0,629787	0,319989	0,484055	-0,41888	-0,96763	-0,48781	-0,36315	0,124843	0,279773	0,445197	-0,10693	0,793612	0,833871	0,16109	0,33316
NCI_BCR_signaling_Pathway_(re_entry_into_mitc	-0,22884	0,204784	0,129953	0,978243	0,582375	-0,22589	1,51937	1,056559	2,040875	0,347741	0,501151	0,666802	-0,36242	-0,71993	-0,2409	1,756466	0,132936	0,927441	0,162042	0,334111
reactome_Class_C_3_Metabotropic_glutamate_p	0,196906	0,272883	0,403441	1,263422	3,486272	2,940104	0,209434	0,034351	-0,14906	6,136125	5,207912	3,786545	-0,54288	-0,64044	-1,98481	1,744752	2,267469	2,218189	0,162068	0,334111
biocarta_alternative_complement_Main_Pathway	0,001293	0,394007	0,460791	-0,90293	-0,26769	1,219315	0,599241	0,44364	1,097891	-0,14092	-0,33513	0,257106	-1,02717	-1,06017	-1,10576	0,088156	0,140735	-0,06074	0,162241	0,334216
Mitochondrial_Apoptosis_Pathway_Depolarizatio	0,248481	0,067913	0,028493	0,056361	-0,19567	0,451576	0,053353	0,212551	-0,28941	-0,0181	0,746231	0,139151	0,117347	0,101125	0,210267	-1,51356	-1,64635	-0,91006	0,162334	0,334216
BRCA1_Pathway_Base_Excission_Repair	-0,00676	-0,04436	-0,02535	0,666724	0,188953	-0,43947	0,598244	0,592696	0,374389	0,127465	0,297228	0,237836	0,287282	0,214277	0,32106	-0,47093	-0,41528	-0,20613	0,162454	0,334242
ErbB_Family_Pathway_Gene_Expression_via_JUN	-0,21837	-0,58539	-0,47386	0,060553	-1,17159	-1,49568	-0,43804	0,294094	-0,87009	-1,40935	0,664955	-1,58055	0,464013	0,075448	0,717018	-2,44617	-1,71033	-0,74273	0,162597	0,334317
reactome_MHC_class_II_antigen_presentation_M	0,055517	0,049077	-0,3365	-4,33778	-1,82135	-2,2995	-8,34624	-6,91684	-9,8056	-3,50153	-0,81051	-1,16199	-0,04973	0,67121	1,999869	-12,9272	-9,97769	-4,69904	0,162705	0,334319
superpathway_of_purine_nucleotide_salvage	0,01197	-0,47962	-0,43059	-0,2795	-0,64331	-0,60657	1,540433	1,862742	0,824266	0,874401	2,822004	0,671376	0,455472	0,82418	1,405892	-7,26214	-6,18511	-5,15731	0,162959	0,334618
reactome_MyD88_independent_cascade_Main_F	-0,1642	-0,11946	-0,22106	-1,14333	-0,93797	-0,9429	-1,95891	-1,77407	-2,2742	1,888752	1,72194	1,852325	-0,28499	-0,48175	-0,72147	-1,29104	-1,74826	-1,70366	0,163978	0,33649
reactome_G_beta_gamma_signalling_through_PI	-0,0822	-0,18759	-0,22926	2,131446	2,717865	1,11607	0,234008	0,04351	0,22795	-1,4846	1,517539	1,524585	-0,41043	-0,26556	-0,09805	-1,07755	-1,2438	0,134536	0,164087	0,336492
KEGG_Hepatitis_C_Main_Pathway	0,631228	-0,54209	-0,372	-1,38088	-0,31713	-1,81202	-2,53577	-1,49012	-3,65375	1,282537	0,618628	-0,14476	0,920413	0,829052	1,172791	-7,41368	-6,37822	-5,18862	0,164836	0,337806
reactome_Generation_of_second_messenger_mo	-0,95182	0,315516	0,3687	-0,25104	1,278358	2,168611	-1,4809	-2,29429	0,060998	0,323823	1,33503	1,875525	0,223645	0,488816	0,771702	5,712299	4,615831	5,790729	0,16508	0,338082
reactome_Gap_junction_degradation_Main_Path	-0,0376	0,028143	0,006976	-0,10649	-0,30131	-0,49085	-2,38412	-2,2224	-2,43421	-1,34708	0,519158	-0,44722	0,031252	0,176603	0,301914	-1,09808	-0,96897	-0,40355	0,165544	0,33881
KEGG_Glycosaminoglycan_degradation_Main_Pat	-0,04458	-0,06692	0,037903	-2,44636	-2,0551	-1,4506	-2,17473	-2,12306	-1,8588	2,183502	0,78578	1,530713	-0,4099	-0,87547	0,10243	-2,42769	-1,60666	-2,24018	0,165765	0,338817
resolvin_D_biosynthesis	0,00836	-0,0326	0,174881	-0,17837	-0,6135	-0,41461	-0,00912	-0,17897	0,077902	0,449813	0,557545	0,811472	0,337415	0,291403	0,316293	0,047224	0,457651	-0,13526	0,165754	0,338817
NCI_amb2_Integrin_signaling_Pathway_(actin_fila	-0,00762	-0,12299	-0,1065	0,9763	0,18573	0,626987	-0,25308	-0,20816	0,035624	0,356671	-0,36524	-0,35395	-0,1239	-0,03945	0,028932	0,258262	-0,28726	0,343526	0,16628	0,339425
reactome_HDMs_demethylate_histones_Main_Pa	0,171944	-0,19199	-0,3031	-1,07221	-0,97931	0,295953	1,237076	1,691738	0,400988	-0,73648	0,988626	0,484886	0,690025	0,596564	1,115106	-2,84062	-1,95892	-2,30848	0,166233	0,339425
pyruvate_decarboxylation_to_acetyl_CoA	0,196874	0,10866	0,065896	-0,53973	-0,2181	-0,67413	0,214226	0,299547	0,072067	-0,91838	-0,34904	-0,63799	-0,17401	-0,0603	-0,12713	-2,35313	-1,79586	-1,24414	0,166683	0,340024
reactome_Chylomicron_mediated_lipid_transpor	-0,45577	-0,16511	0,049356	-1,03946	-0,27194	-1,18891	-2,28537	-2,846	-1,86257	1,076718	0,65952	-0,71128	0,388992	0,691103	0,299594	0,812994	1,301932	1,171637	0,167735	0,341946
biocarta_ras_independent_pathway_in_nk_cell_r	0,023785	-0,35788	0,251588	1,159194	1,953607	1,790386	1,340309	1,26867	1,449907	-0,029	-2,04322	-0,37234	1,543847	2,680774	1,168694	3,075375	2,809803	3,249453	0,168075	0,341969
HGF_Pathway_PKC_Pathway	-0,25832	-0,24395	-0,04079	-1,02273	-1,1833	-1,52989	-1,21988	-1,08476	-0,97037	0,726371	0,571168	-0,23668	-0,12246	-0,28787	0,080397	-0,85506	-0,91265	-0,48259	0,168066	0,341969
reactome_Alpha_oxidation_of_phytanate_Main_	0,020214	-0,09344	-0,02995	0,188288	0,347288	0,35251	-0,02675	0,113416	0,006424	-0,17084	0,46434	0,056896	0,005211	-0,46586	-0,37395	-1,14674	-1,29543	-1,13627	0,167859	0,341969
NCI_SHP2_signaling_Main_Pathway	0,271182	-0,39313	-0,23353	-0,83915	-1,02487	-1,84624	-4,64468	-4,92994	-4,87463	0,644327	-1,98467	-1,37915	0,678144	0,139261	1,230662	-4,53227	-2,60769	-1,70475	0,168362	0,342328
reactome_Sperm_Motility_And_Taxes_Main_Patl	0,010695	0,223955	0,31924	0,408172	-0,01917	0,461161	-0,09612	-0,54728	0,232674	0,263482	0,102311	-0,00063	-0,34863	0,223745	-0,2077	1,307789	0,793382	0,328034	0,168542	0,342471
KEGG_Citrate_cycle_TCA_cycle__Main_Pathway	0,696985	0,290194	0,162902	-1,68683	-1,06898	-2,49595	1,374626	1,937477	0,379898	-2,50259	0,5988	-1,18707	-0,51788	-0,3468	-0,33422	-10,9005	-10,5961	-10,2568	0,16915	0,343483
biocarta_eukaryotic_protein_translation_Main_Pa	0,390631	0,365341	0,255419	-0,13188	0,190206	-0,77759	0,101276	0,354885	-0,58352	0,130416	0,144077	-0,27033	0,130771	-0,12928	0,106519	-6,02672	-4,16529	-2,68592	0,169357	0,34368
biocarta_erk_and_pi_3_kinase_are_necessary_fo	0,284574	0,217319	0,324852	0,198813	-0,1055	1,001191	2,001627	1,85539	2,002561	0,35348	-0,51359	0,191204	-1,55392	-1,0043	-1,45527	0,048281	0,19238	0,585693	0,169553	0,343853
GPCR_Pathway	-1,62009	-3,35342	-2,04174	-7,99777	-0,4226	-5,10867	-15,2071	-13,9426	-16,1499	-6,29592	-5,33025	-7,6056	1,491534	2,390933	5,302866	-9,80593	-6,06014	3,311557	0,169915	0,344364
NCI_VEGFR3_signaling_in_lymphatic_endothelium	0,111923	-0,12538	-0,09954	-0,81257	0,27116	-0,39738	-0,82847	-0,91727	-1,15508	0,222146	0,574239	-0,0288	0,43191	0,247314	0,356926	-3,11543	-1,60463	-1,84071	0,17106	0,346233
reactome_Beta_catenin_phosphorylation_cascad	0,494021	0,030388	-0,057	0,391754	-0,17754	-0,4741	-0,64632	-0,35974	-1,16387	-0,29056	0,476032	0,43151	0,299219	0,28592	0,087531	-2,49528	-2,07853	-1,24322	0,171014	0,346233
reactome_PLC_gamma1_signalling_Main_Pathwa	-0,04824	-0,0396	-0,00705	-0,02157	0,129351	0,150474	0,231508	0,056826	0,3492	0,119355	-0,07451	0,232931	0,209234	-0,05147	0,131756	-0,30197	0,005975	0,086485	0,171587	0,347073
biocarta_calcium_signaling_by_hbx_of_hepatitis_	-0,12306	-0,10054	-0,06178	0,356149	0,614943	0,554331	0,182853	0,137255	0,346122	-0,2361	0,437313	0,458475	-0,34877	-0,51809	-0,32747	1,628345	1,552248	1,137234	0,172212	0,348113
reactome_Oxygen_dependent_proline_hydroxyla	0,288294	-0,04847	0,010004	-0,83117	-0,47764	-1,36718	0,167158	0,263535	-0,50247	-0,23113	0,42011	-0,40128	-0,04395	0,110268	0,464754	-6,19401	-5,48921	-4,02316	0,17261	0,348689
dolichyl-diphosphooligosaccharide_biosynthesis	0,152394	0,064652	-0,00744	0,610214	0,119369	-0,11468	0,732322	1,030885	0,398584	0,365045	1,204101	0,363373	0,069807	-0,00644	-0,13277	-4,4987	-2,77692	-3,03153	0,17292	0,348864
Growth_Hormone_Signaling_Pathway	-0,66585	-0,90013	-0,63042	0,048548	-2,75385	-2,86226	-0,79446	0,150153	-1,54402	1,270417	0,293293	-0,85304	1,618883	0,90872	2,058453	-7,53473	-6,29017	-3,58201	0,172846	0,348864
reactome_Rho_GTPase_cycle_Main_Pathway	0,294999	-1,0803	-0,53341	-0,72504	-2,03996	-2,66451	-7,11658	-6,77495	-8,803	-3,06292	0,681753	1,661567	-0,63427	-0,47751	0,834374	-14,6451	-10,4	-7,71992	0,173185	0,349172
biocarta_tsp_1_induced_apoptosis_in_microvasc	-0,16774	-0,16173	-0,21471	-0,35306	-0,27305	-0,73205	-0,42304	-0,18302	-0,64309	-0,31704	-0,28398	-0,12656	9,81E-05	0,012209	0,192256	-1,78641	-0,55712	-1,1269	0,173869	0,349646
CMP-iNi-acetylneuraminate_biosynthesis_I_eukar	0,159743	0,025302	0,028706	-0,05048	0,230602	-0,00298	-0,23849	-0,14189	-0,50529	0,038796	0,195849	0,06999	-0,19346	0,165789	-0,389	-1,74588	-1,66858	-1,44386	0,173707	0,349646
NCI_ATR_signaling_Pathway_(Pathway_negative_	0,700304	0,160149	0,138758	0,500554	0,679516	0,097946	1,603218	2,484906	0,557866	1,637974	0,859369	0,796537	-0,35461	-0,02747	0,361586	-8,34284	-6,08662	-4,49931	0,1736	0,349646
reactome_Conjugation_of_benzoate_with_glycine	-0,10974	-0,00932	0,01505	-0,08622	0,212547	0,35317	1,005122	1,153121	1,023864	0,494837	0,081137	0,266849	0,507705	-0,02947	-0,22656	1,480098	1,387501	0,470504	0,173847	0,349646
superpathway_of_pyrimidine_ribonucleotides_ide	0,205792	0,151659	0,124606	-0,64241	0,050636	-0,58243	0,665096	0,822486	-0,03358	0,559563	1,581587	1,111234	0,851125	0,791473	1,086017	-5,41195	-4,67399	-3,93506	0,174247	0,35018
NCI_RAC1_signaling_Main_Pathway	-0,19809	-0,18971	-0,34086	-3,22929	-1,62661	-2,39775	-1,46263	-1,18732	-2,57498	-2,58078	-0,17752	-0,05065	0,760353	0,736337	1,88483	-9,12493	-6,18037	-5,04431	0,174545	0,350553
NCI_Signaling_events_mediated_by_VEGFR1_and	0,091365	-0,33665	-0,33823	-0,70571	0,856641	0,719268	-3,72407	-3,54386	-4,45952	1,819786	0,797537	0,392401	-0,02894	0,069679	-0,01097	-5,06901	-3,58078	-3,03612	0,174757	0,350647
reactome_BH3_only_proteins_associate_with_an	-0,19239	-0,20938	-0,13312	0,050082	0,698026	0,645819	-0,25065	-0,25112	-0,32067	-0,06013	0,286165	0,198733	0,02467	0,255302	-0,07941	0,012264	0,1042	0,334311	0,174817	0,350647
biocarta_regulation_of_map_kinase_pathways_th	0,292863	0,161525	0,067677	-0,31141	0,248148	-0,17272	0,615705	0,767194	0,252231	-0,418	0,11534	-1,17217	-0,06352	0,339083	0,064977	-0,81044	-0,27785	-0,27925	0,174946	0,350681
reactome_IKK_complex_recruitment_mediated_b	0,258584	-0,0159	0,096419	-0,31877	-0,52163	-0,16891	-0,17273	0,042939	-0,44632	0,354334	-0,28635	-0,13573	-0,02515	-0,19152	0,091312	-0,04892	-0,29264	-0,12806	0,175885	0,352335
NCI_FGF_signaling_Pathway_(MAPKKK_cascade)	0,050247	0,095663	0,12473	0,353596	0,179657	0,249079	0,247913	0,108535	0,374995	0,535757	0,528255	-0,24584	0,197499	0,11374	0,095601	1,201097	0,82519	0,74683	0,176601	0,353543
biocarta_ca_calmodulin_dependent_protein_kina	-0,10099	-0,27544	-0,19585	0,260907	0,407879	0,468323	-0,21511	-0,1069	-0,44503	-0,91676	-0,06894	-1,75748	0,010576	-0,10676	0,064783	0,404512	0,493784	0,599903	0,176894	0,353771
KEGG_Starch_and_sucrose_metabolism_Main_Pa	-0,26574	-0,27618	0,109463	-8,32631	-9,75999	-12,6919	3,339387	3,432275	4,162043	-4,50858	1,532553	2,60551	0,54968	-1,75368	-2,26245	-4,9864	-4,81373	-8,00085	0,176943	0,353771
NCI_Insulin_Pathway_Pathway_(negative_regulat	0,189969	-0,045	-0,04116	0,159672	0,042708	0,015406	-0,7678	-0,70733	-1,03954	0,664271	0,113385	-0,2164	-0,05077	0,00464	0,068935	-1,87126	-1,54367	-0,59245	0,177403	0,354465
Growth_Hormone_Signaling_Pathway_Gene_Exp	-0,53319	-0,85515	-0,71429	-0,71095	-3,65328	-2,87775	-0,35214	0,801564	-1,11185	-0,16203	0,884742	-1,84616	1,307628	0,749968	1,8723	-8,65827	-7,26108	-4,61277	0,177814	0,35483

KEGG_SNARE_interactions_in_vesicular_transpor	0,11048	-0,01458	0,059862	0,227329	0,316941	-0,04783	-0,38606	-0,03304	-0,85035	0,511686	0,605006	0,858128	-0,12257	0,127758	0,206428	-2,6654	-2,6384	-2,42951	0,1777	0,35483
reactome_Toxicity_of_botulinum_toxin_type_E_E	-0,14071	0,038156	0,017494	0,024632	-0,17559	-0,36155	-0,72321	-0,76195	-0,70437	-1,03875	-0,01499	-0,77566	-0,06343	0,080475	-0,19465	2,069472	2,503778	2,574462	0,178239	0,355451
NCI_ALK1_signaling_events_Main_Pathway	-0,52633	-0,11057	-0,2789	-0,73186	-0,58306	-0,86841	-2,88993	-2,90573	-3,63996	-0,71951	-0,67529	-0,09556	-0,03167	-0,09591	-0,54002	-3,45556	-2,91975	-1,14591	0,179451	0,357182
TGF-Beta_Pathway_Transciption_Arrested_Growt	0,038693	-0,06578	0,117416	0,362661	-0,88281	-1,0886	-0,42544	-0,13482	-1,1856	-0,92663	1,016053	-0,72316	0,294018	-0,08572	0,116572	-3,77711	-3,05653	-2,19625	0,179401	0,357182
TGF-Beta_Pathway_Transciption_Cell_Growth_ar	0,038693	-0,06578	0,117416	0,362661	-0,88281	-1,0886	-0,42544	-0,13482	-1,1856	-0,92663	1,016053	-0,72316	0,294018	-0,08572	0,116572	-3,77711	-3,05653	-2,19625	0,179401	0,357182
KEGG_Thyroid_hormone_synthesis_Main_Pathwa	-0,05982	-0,00876	0,056499	-0,65592	-0,02409	0,164652	0,175351	0,003738	0,450756	0,634964	0,394687	-0,01077	0,38167	0,368886	0,387416	1,144997	0,74087	0,932967	0,179604	0,357257
KEGG_Oxidative_phosphorylation_Main_Pathway	1,623809	0,315101	0,421454	-4,32872	-6,79706	-4,4817	1,229243	2,181121	-2,25703	-6,11141	2,948322	-2,15605	-2,12007	-2,25113	-0,49742	-40,3101	-35,1806	-32,259	0,179802	0,357397
reactome_FCERI_mediated_MAPK_activation_Ma	-0,00696	-0,05706	0,11957	-1,04492	-0,8198	-0,50292	-1,1476	-1,18159	-2,25361	-1,18487	0,298625	0,303384	0,476368	0,887619	0,873345	-3,08131	-2,24804	-1,26663	0,179903	0,357397
KEGG_Amphetamine_addiction_Main_Pathway	0,046845	-0,28976	-0,2281	1,62312	1,638678	2,503697	0,312303	0,454783	0,036745	-3,90763	-1,11311	-1,94463	-0,5436	-0,33354	0,090754	0,868886	1,884687	1,91725	0,180418	0,358066
NCI_HIV_1_Nef_Negative_effector_of_Fas_and_T	0,21032	0,092993	0,058915	-0,01313	0,00526	-0,09046	-1,4673	-1,05375	-1,66336	0,618922	-1,42344	-0,2461	0,005207	-0,40729	-0,61423	-2,69647	-1,96748	-1,56036	0,18047	0,358066
biocarta_signal_dependent_regulation_of_myoge	0,044532	0,043879	-0,04633	-0,06967	-0,13512	-0,29074	-1,55949	-1,61061	-1,60734	-1,26787	-0,1714	-0,30563	0,272903	0,108752	-0,17393	-0,26414	-0,72337	0,081596	0,180679	0,358167
FN1_Influence_on_Cell_Motility_via_Integrins	-0,41448	-0,72891	-0,54852	-2,49804	-2,16598	-2,07018	-4,51196	-4,07198	-5,6014	-2,10146	-1,43994	0,285869	-0,11066	-0,06852	0,327362	-8,80948	-6,30941	-4,84766	0,181243	0,358167
NCI_DNA_PK_pathway_in_nonhomologous_end_	0,176167	-0,01763	0,033455	0,614557	0,05151	-0,08061	-0,48631	-0,25981	-0,90648	-0,08888	0,6027	-0,35226	-0,56018	-0,06699	0,039327	-2,34222	-1,56683	-0,9355	0,18094	0,358167
NCI_mTOR_signaling_Main_Pathway	0,479429	-0,33544	-0,53452	-1,27633	-0,95899	-4,28191	-5,01922	-4,3398	-7,03693	-0,05235	-0,56594	-2,81188	0,724478	0,215324	1,039225	-9,79901	-7,31764	-4,38949	0,181257	0,358167
NCI_Posttranslational_regulation_of_adherens_ju	-0,36735	-0,30929	-0,25392	-1,18197	-0,7685	-1,45516	-9,72631	-9,76895	-9,89368	-1,79049	0,778159	1,938006	1,247002	0,39763	0,758203	-5,18237	-3,65627	-4,92626	0,181326	0,358167
reactome_CS_DS_degradation_Main_Pathway	-0,29719	-0,30679	-0,27223	-0,84859	-0,10313	0,107045	1,591393	1,689854	1,803249	0,924504	-0,64905	1,810431	0,79994	0,664632	1,634512	4,320026	4,142498	3,579828	0,181069	0,358167
reactome_Vitamin_C_ascorbate_metabolism_Ma	-0,09735	-0,18259	-0,17252	-0,62668	0,285198	-0,13434	-0,95923	-0,91581	-1,15526	0,278943	0,27655	0,021518	0,635902	0,346923	0,280403	-0,56476	-0,49373	-0,70783	0,181032	0,358167
biocarta_pdgf_signaling_Main_Pathway	-0,10503	-0,39286	-0,51007	-1,90237	-2,70321	-2,88509	-1,59045	-1,1148	-2,25435	-0,92008	0,33295	-0,15229	-0,20534	-0,61036	-0,06991	-2,8103	-0,469	0,376398	0,181463	0,358211
reactome_CD28_dependent_PI3K_Akt_signaling_	-0,32761	-0,26799	-0,29717	-1,36238	0,369283	0,387649	1,516755	1,689598	0,97283	0,163435	-0,82551	0,061011	0,111569	1,281665	0,885645	-2,10593	0,311936	0,031803	0,182307	0,359476
reactome_Regulation_of_innate_immune_respor	-0,27695	-0,24246	-0,25272	-1,07832	-1,39307	-1,05576	-0,11433	-0,26803	-0,2529	1,316094	0,001177	-0,61665	-0,66706	-0,38189	-0,72172	-1,454	-1,39281	-0,98838	0,182334	0,359476
reactome_Glycoprotein_hormones_Main_Pathwa	0,091968	0,417454	0,294035	0,624566	0,610457	0,444637	0,209867	0,136177	0,409396	0,441397	0,75257	0,148262	-0,14166	0,174117	-0,69576	0,589546	1,024148	0,877476	0,182517	0,359608
Interferon_Pathway_Transciption	-0,19911	-0,47688	-0,41527	-1,15067	-0,044	-1,13317	-0,16295	0,685312	-0,97382	0,087278	0,544627	1,200563	0,080543	0,497816	0,90704	-5,65986	-4,4845	-3,03989	0,183629	0,36157
biocarta_ucalpain_and_friends_in_cell_spread_M	-0,21169	-0,22101	-0,41052	-0,91102	-0,64022	-1,16053	-2,18142	-1,94772	-2,30854	-0,86016	0,877803	-1,06119	0,455897	-0,00179	0,112183	-2,45694	-2,63549	-2,29449	0,183892	0,36163
reactome_negative_regulation_of_TCF_depender	0,006472	0,0967	-0,02872	0,066693	-0,09384	0,043693	0,215442	0,183147	0,232515	0,157965	-0,02868	0,093469	0,305141	0,184845	0,322951	-0,38491	0,363502	-0,31218	0,183881	0,36163
Mitochondrial_Apoptosis_Pathway_DNA_Fragme	-0,05888	-0,07435	-0,03647	0,205864	0,109458	-0,60851	0,373231	0,307083	0,427867	0,017042	0,064088	-0,24992	0,430502	0,090177	0,33517	-0,22101	-0,57713	-0,19103	0,184459	0,361684
NCI_amb2_Integrin_signaling_Main_Pathway	-0,22797	-0,23617	0,039544	0,269461	0,238631	0,118025	-3,86649	-4,06117	-2,97256	0,551702	-1,23959	-0,94954	0,521448	0,953551	0,324228	1,061125	-0,24162	1,098632	0,184049	0,361684
NCI_DNA_PK_pathway_in_nonhomologous_end_	0,287979	0,010487	0,051431	0,417057	0,114882	0,208787	-0,5292	-0,35093	-0,99974	-0,30212	0,559634	-0,18541	-0,51826	-0,07271	-0,03919	-2,18567	-1,32745	-0,7802	0,184616	0,361684
Neuronal_Intermediate_Filaments	-0,00987	-0,13542	-0,0643	-0,13952	-1,08239	-0,42276	-0,81377	-0,9123	-0,9085	-2,10807	-0,45411	-0,35	-0,12455	-0,15314	0,133896	-1,21353	-0,97367	-0,99488	0,184564	0,361684
reactome_DNA_replication_initiation_Main_Path	0,245434	0,229906	0,191441	0,317557	0,289854	0,810993	1,926168	2,111887	1,359596	-0,17918	-0,8091	-0,39413	-0,81704	-0,49663	-0,40978	-2,08324	-1,34839	-0,96125	0,184498	0,361684
reactome_Telomere_C_strand_synthesis_initiatio	0,245434	0,229906	0,191441	0,317557	0,289854	0,810993	1,926168	2,111887	1,359596	-0,17918	-0,8091	-0,39413	-0,81704	-0,49663	-0,40978	-2,08324	-1,34839	-0,96125	0,184498	0,361684
reactome_Relaxin_receptors_Main_Pathway	0,0524	0,126263	-0,07919	-0,02689	-0,01509	-0,46523	-0,17794	-0,30868	0,003767	0,933757	-0,21064	0,416348	-0,01535	-0,00313	-0,0817	-0,34127	0,026274	0,005394	0,185247	0,362693
KEGG_Glycosaminoglycan_biosynthesis_keratan_	-0,13816	-0,10167	-0,44372	1,506109	0,365225	2,393713	-1,82113	-1,94734	-2,03409	1,337092	1,09429	-0,75181	-0,5032	-0,44356	-0,09833	-1,75224	-1,67455	-0,33755	0,185395	0,362754
reactome_Branched_chain_amino_acid_catabolis	0,488952	0,138466	0,137054	0,178246	0,065539	-0,71632	1,276418	1,885677	0,138466	0,364449	1,897588	0,056131	-0,22288	0,39659	0,4518	-6,39571	-5,48034	-4,38655	0,186003	0,363715
glutaryl-CoA_degradation	0,030669	-0,0649	0,021266	-0,21872	0,338653	0,620294	0,23935	0,185854	0,292495	-0,14593	-0,29902	0,312764	-0,15621	-0,07198	-0,14439	-0,53182	-0,45257	-0,46214	0,186853	0,364568
NCI_N_cadherin_signaling_events_Pathway_(lam	-0,0533	-0,06879	-0,09455	-0,01174	0,797557	0,039938	-0,36648	-0,38125	-0,66563	0,372111	0,964098	0,572058	-0,09131	-0,074	0,03194	-3,01565	-2,08434	-1,58466	0,186868	0,364568
PRL_Pathway	-0,95026	-1,12238	-1,32865	1,434441	1,32365	-1,7917	-4,11607	-4,51532	-5,29244	2,358692	-0,80696	4,346858	-0,94182	0,395187	0,393284	-8,30897	-5,90395	-5,35672	0,187024	0,364568
reactome_Deposition_of_new_CENPA_containing	0,754548	0,064418	-0,13469	0,366748	0,279094	-0,73045	7,509824	8,431211	5,577711	0,367841	0,764367	-0,90715	-1,49334	-1,26946	-0,13568	-12,164	-7,31686	-6,52064	0,186574	0,364568
reactome_TRAF6_mediated_induction_of_TAK1_	0,167036	-0,04342	-0,02383	-0,76867	-0,88744	-0,90272	0,223377	0,390346	-0,15073	-0,74596	0,085295	-0,36852	0,231007	0,264238	0,142693	-3,45967	-2,81412	-2,3666	0,18692	0,364568
biocarta_human_cytomegalovirus_and_map_kina	0,111309	-0,20158	-0,3353	-0,40195	0,219453	-0,47178	0,050975	0,350204	-0,65313	-0,38259	1,173664	1,251903	0,142583	0,403935	0,482727	-4,0641	-2,68035	-2,32474	0,187697	0,365013
NCI_Beta1_integrin_cell_surface_interactions_Ma	-1,41367	-0,93467	-0,77072	-5,34221	-6,96752	-5,67914	-7,97496	-8,69363	-6,95346	1,379577	0,081013	0,328234	1,377796	0,40127	0,498502	1,159539	-0,61881	-1,49009	0,187412	0,365013
purine_nucleotides_ide_novoi_biosynthesis	0,124982	-0,2984	-0,43281	-0,21865	-0,24459	-0,70856	0,483439	0,742639	-0,33514	1,803319	2,264578	0,436146	0,209448	0,159697	0,997795	-7,73456	-7,14804	-5,76368	0,187721	0,365013
UTP_and_CTP_ide_novoi_biosynthesis	0,13738	0,1276	0,041779	-0,82461	-0,45347	-0,34865	0,626804	0,726954	-0,02938	0,828686	1,261693	0,698042	0,76359	0,87089	1,030895	-4,75766	-4,13349	-3,55018	0,187597	0,365013
KEGG_Pertussis_Main_Pathway	0,258582	-0,28892	-0,49935	-0,10865	0,058856	-1,50778	-2,83514	-2,22686	-3,74787	-0,02839	0,685871	0,836835	0,404526	-0,30197	-0,23505	-5,61445	-5,38014	-4,61367	0,188062	0,365301
reactome_Recycling_pathway_of_L1_Main_Pathv	0,337143	-0,01474	-0,16485	-0,13193	-0,5401	-0,95466	-2,92335	-2,63019	-4,0319	-1,55099	0,971912	-1,7421	-0,30275	-0,59733	0,148319	-3,54775	-2,81097	-1,81038	0,188104	0,365301
HIF1Alpha_Pathway_NOS_Pathway	-0,28265	-0,38419	-0,30301	0,28471	0,859589	-0,53135	-1,2374	-1,10145	-1,20933	0,424346	-0,11615	0,939036	0,599478	-0,01083	-0,01326	-1,51815	-1,30264	0,217194	0,188391	0,365403
HIF1Alpha_Pathway_VEGF_Pathway	-0,28265	-0,38419	-0,30301	0,28471	0,859589	-0,53135	-1,2374	-1,10145	-1,20933	0,424346	-0,11615	0,939036	0,599478	-0,01083	-0,01326	-1,51815	-1,30264	0,217194	0,188391	0,365403
Akt_Signaling_Pathway_Proto-Oncogenic_and_RT	-0,02358	-0,16723	0,318774	-1,37508	0,471961	-1,12554	0,464341	0,37109	0,573745	0,699283	1,747049	0,023647	0,51417	0,381363	0,098533	-0,11582	0,423491	0,715391	0,188763	0,365897
KEGG_Adrenergic_signaling_in_cardiomyocytes_I	-1,74591	-0,91782	-0,87076	-3,38281	-1,06368	-3,33949	-1,70054	-2,98638	0,199762	-2,8017	-1,32471	-4,6971	0,487278	1,918208	1,838128	16,00031	14,50736	16,49244	0,189141	0,366402
reactome_Terminal_pathway_of_complement_M	0,050998	0,222492	0,209417	-0,53156	0,127318	0,83479	0,608717	0,623153	0,913571	-0,31638	0,280605	0,352663	-1,07045	-0,97623	-1,00854	-0,32571	0,214936	-0,25787	0,189789	0,367428
NCI_Alpha_synuclein_signaling_Main_Pathway	-0,07372	-0,25247	0,027448	0,065898	1,469789	0,412	-1,72216	-2,03116	-1,76718	-1,50818	-0,02281	0,495974	0,128915	0,629676	1,148887	1,636737	0,092625	0,840658	0,190532	0,36841
NCI_Aurora_C_signaling_Main_Pathway	0,091599	0,004394	-0,03599	0,236953	0,032259	0,120542	0,662355	0,729076	0,695623	-0,40587	-0,23056	-0,34104	-0,06435	-0,2139	-0,26217	-0,06706	-0,21661	-0,24767	0,190512	0,36841
NCI_Plasma_membrane_estrogen_receptor_signa	-0,01829	-0,01741	-0,04178	0,33192	0,375471	0,169649	0,137812	0,145784	0,173793	-0,31012	-0,16591	-0,57054	-0,31568	-0,20432	-0,17642	-0,07514	-0,11768	-0,1722	0,190743	0,368588
pyrimidine_deoxyribonucleotides_biosynthesis_fr	0,203753	0,05255	-0,11665	-0,63838	-0,26465	-0,00246	0,854057	0,966974	0,034514	1,143982	2,552056	0,949979	1,023936	1,006767	1,407881	-5,60919	-4,39699	-3,64332	0,191063	0,368977
eumelanin_biosynthesis	0,190975	0,099297	0,299426	-0,45236	0,607414	0,158667	0,000884	-0,11101	-0,12245	0,794721	-0,14498	0,418208	-0,6105	-0,56573	-0,27376	0,020656	-0,08457	-0,39162	0,191571	0,369501
reactome_Degradation_of_cysteine_and_homocy	0,10275	0,125516	0,141399	-0,42951	-0,11705	-0,64293	0,510908	0,574118	0,29074	0,715885	0,310575	0,689497	-0,33583	-0,11914	-0,00175	-0,66763	-0,7974	-0,48595	0,191505	0,369501
cAMP_Pathway	-6,93506	-3,4101	-1,693	-11,904	-6,04124	-13,6583	-25,2078	-28,676	-18,298	1,050086	-2,72317	-10,9501	3,970001	1,726879	7,07777	26,44164	21,41325	21,29186	0,191919	0,369943
NCI_Canonical_Wnt_signaling_Main_Pathway	-0,25949	-0,46728	-0,41177	-0,59772	-1,04906	-0,96028	-1,44017	-1,33142	-1,89729	1,123296	-0,59912	-1,10013	0,378402	0,859856	0,488932	-2,10561	-2,38069	-1,10822	0,192166	0,370189
Akt_Signaling_Pathway_NO_production	0,095264	-0,13421	0,313246	-1,59692	0,392815	-1,09671	0,247582	0,103858	0,359702	0,103763	1,620288	-0,17909	0,633533	0,563268	0,13746	0,299677	0,995912	1,050663	0,19304	0,371645
cysteine_biosynthesis	0,050481	0,181394	0,073909	-0,21317	-0,0454	-0,00709	0,168315	0,256311	0,20778	0,425876	-0,45735	-0,12035	0,099355	-0,33385	0,101689	-1,27941	-1,47889	-1,05316	0,194135	0,373521
KEGG_Glycerolipid_metabolism_Main_Pathway	-0,05041	0,274483	0,007327	-2,97124	-1,80394	-2,29191	1,0062	1,120842	0,413636	3,664245	0,995948	1,490645	0,38244	0,265401	1,48558	-1,90828	-0,84174	0,130037	0,194631	0,373783
reactome_Regulation_of_pyruvate_dehydrogena	0,044397	-0,01168	0,002081	-2,35992	-1,67523	-2,18373	1,011125	1,019103	0,680015	-1,4146	-1,3071	-0,65002	1,256258	0,96101	1,211866	-5,26153	-4,37021	-3,50994	0,194424	0,373783
reactome_Trafficking_of_AMPA_receptors_Main_	-0,2332	-0,38454	-0,28063	0,865018	0,247424	1,23118	-0,85057	-0,67319	-1,11038	-0,10684	0,210183	-2,08418	0,183304	0,248778	0,424137	0,294605	0,749383	1,826935	0,194614	0,373783
reactome_Interleukin_7_signaling_Main_Pathway	-0,25227	-0,08916	-0,0431	-1,97275	-0,96558	-0,90189	0,003535	0,177594	0,34941	0,94533	0,670925	1,045534	0,570422	-0,25	0,054955	-0,60998	-0,40666	-0,97025	0,194824	0,373923
KEGG_Sphingolipid_signaling_Main_Pathway	-0,7248	-0,55866	-0,45036	-0,24734	1,182214	-3,87367	-0,69209	-0,87796	-0,43715	-0,96305	-0,59443	-1,43507	-2,20731	-0,45856	-0,37527	1,221619	2,424572	3,635679	0,195646	0,37527
HGF_Pathway_Cell_Cycle_Progression	-0,18311	-0,2892	-0,36916	-0,39735	-0,9879	-0,23356	-1,19577	-0,92012	-1,69279	-0,23733	-0,16235	0,445731	0,049907	-0,13763	0,262803	-2,62651	-1,5802	-1,83434	0,196706	0,377069
reactome_Thrombin_signalling_through_proteina	-0,33198	-0,64932	-0,62849	0,706996	1,381298	0,614857	-2,12977	-2,29519	-2,08875	-0,82123	1,921297	1,465644	-0,29911	-0,05393	0,323719	-1,50909	-0,95541	-0,0135	0,197203	0,377791
NCI_Nephrin_Neph1_signaling_in_the_kidney_po	0,073412	0,035436	-0,18013	-0,70006	-0,41027	-0,21739	-0,13421	0,051539	-0,60899	0,045296	-0,21976	-0,11814	-0,11306	0,13976	0,118169	-2,59275	-1,89134	-1,3573	0,19789	0,377945
PAK_Pathway	-6,96767	-1,47325	-1,02202	-14,024	-5,85363	-11,1892	-17,8155	-22,4615	-9,7999	4,132367	-12,0989	-4,67579	2,365553	2,81153	3,562776	19,22551	12,01506	15,95865	0,197761	0,377945
PTEN_Pathway_Proliferation	-0,8579	-0,13313	-0,15541	-2,44837	-2,72834	-0,34661	7,324681	7,124187	8,402838	-0,40221	-6,41443	-4,46454	1,182425	0,343514	0,052654	16,32861	15,59735	11,73493	0,197539	0,377945
PTEN_Pathway_Protein_Synthesis	-0,01174	0,050407	0,010543	-0,14938	-0,00965	0,351828	-0,19434	-0,27513	-0,07322	0,001794	-0,40613	-0,14725	-0,21144	-0,25945	-0,4066	0,614412	0,349026	0,313177	0,19775	0,377945
reactome_Opioid_Signalling_Main_Pathway	-0,15305	0,052551	0,014673	-0,02466	0,013286	-0,07885	0,04059	-0,10691	0,29912	-0,02739	-0,17722	0,028952	0,068015	-0,00552	-0,32207	0,774621	-0,09007	0,602515	0,197792	0,377945
cAMP_Pathway_Cell_Survival	-0,15161	-0,35385	-0,13555	0,743676	0,740987	-1,5554	-2,67875	-2,63981	-2,64139	-4,52785	-1,65518	-0,51581	-1,06909	-0,71953	-0,12653	-1,94125	-1,76214	0,339634	0,198072	0,37806
NCI_Nectin_adhesion_Pathway_(positive_regulati	0,005047	-0,10937	-0,14759	-0,60353	-0,24823	-0,87317	-0,06142	0,031323	-0,15477	0,005021	-0,16318	-0,04853	-0,20392	-0,05393	-0,20759	-0,46227	-0,3579	-0,2548	0,199232	0,37984
superpathway_of_D-imyoi-inositol_145-trisphosp	0,250655	-0,2869	-0,05118	-0,14021	-0,51572	0,510358	-2,91048	-2,71219	-3,94485	0,174731	-0,29822	-0,676	-0,68382	-0,21482	-0,75429	-2,24413	-2,64835	-2,12088	0,199248	0,37984
biocarta_role_of_brca1_brca2_and_atr_in_cancer	0,242484	-0,08915	-0,07578	0,974986	0,716669	0,303764	0,296755	0,917842	-0,48118	1,088424	0,567701	-0,17857	-0,27179	-0,41921	-0,39149	-3,52964	-1,63032	-1,55024	0,200682	0,381645
KEGG_cGMP_PKG_signaling_Main_Pathway	-1,10948	-0,44637	-0,47759	1,819094	5,487164	2,49477	-3,46516	-3,31558	-3,50037	0,430415	-1,84743	-0,7228	-0,17849	0,4859	2,231215	2,975662	1,965901	4,595463	0,200459	0,381645
NCI_BCR_signaling_Pathway_(cell_survival)	-0,15349	0,073956	-0,00286	0,560982	0,215259	-0,16422	-0,06297	-0,37591	0,324963	0,38001	0,109172	0,314602	-0,27364	-0,62067	-0,32388	0,84939	-0,1002	0,121923	0,200685	0,381645
NCI_RhoA_signaling_Pathway_(actin_filament_de	0,080198	-0,01035	-0,001	0,333727	-0,0025	0,070655	0,164141	0,20106	-0,00672	-0,44277	-0,26498	-0,2828	-0,2825	-0,05767	-0,1657	-0,69242	-0,11872	0,079732	0,200509	0,381645
BRCA1_Pathway_NHEJ_DSB_Repair	0,135036	-0,03394	-0,09291	0,511902	0,754329	0,199871	0,079685	0,548235	-0,55155	0,539454	0,439468	0,507977	-0,32537	-0,13016	-0,27302	-2,56491	-1,33493	-1,46294	0,201481	0,381993
NCI_Ceramide_signaling_Pathway_(regulation_of_	-0,02648	-0,0752	-0,09834	-0,7799	0,04352	-0,42636	-1,24917	-1,13397	-1,48853	-0,46817	-1,1532	0,299415	0,242874	-0,20476	0,223231	-0,89612	-1,11955	-0,73036	0,201244	0,381993
NCI_Plexin_D1_Signaling_Pathway_(cell_adhesior	-0,06977	-0,10958	-0,26199	0,288711	-1,02986	0,059534	-3,22305	-3,0866	-3,77634	0,796159	0,840167	0,254843	0,58279	0,574877	0,217013	-1,97909	-1,88647	-2,25614	0,201373	0,381993
reactome_Biosynthesis_of_the_N_glycan_precurs	0,179804	0,034333	-0,01077	0,444082	0,420503	0,40118	0,501232	1,048987	-0,36913	-0,84076	2,66747	0,052637	-0,61174	-0,63851	-0,28155	-5,53626	-4,13328	-4,34037	0,201035	0,381993
reactome_EGFR_Transactivation_by_Gastrin_Mai	0,067097	-0,12337	-0,10032	-0,74875	-1,05157	-0,75442	-0,37773	-0,21127	-0,47585	-0,95943	0,248568	-0,83755	0,069039	0,066304	0,144534	-2,3838	-2,3196	-2,0422	0,201245	0,381993
reactome_G_beta_gamma_signalling_through_PI	-0,37457	-0,23783	-0,39484	2,383537	1,992169	1,467491	-0,1783	-0,61391	-0,24267	-0,52224	1,805564	1,492935	-0,70644	-0,41213	-0,31997	-1,99449	-1,45121	-0,20551	0,201645	0,382071
biocarta_tnfr1_signaling_Main_Pathway	0,173504	-0,05319	0,177817	-0,75222	-0,27606	-1,68097	-1,38543	-1,42148	-1,41608	-1,05624	0,337363	1,237407	-0,36673	0,074799	-0,40209	-0,80597	-0,35069	-0,63685	0,202089	0,38235
inosine-5-phosphate_biosynthesis	0,088596	-5,34E-05	-0,01488	-0,44723	-0,01598	-0,67938	-0,23747	-0,14095	-0,39169	-0,78429	-0,12449	-1,00705	-0,26185	-0,12912	-0,05033	-1,16449	-1,02083	-0,77448	0,202161	0,38235
WNT_Pathway_Cell_Fate_Proliferation_Differenti	-1,93625	-1,60671	-1,58544	0,320447	1,535653	1,93813	0,674138	0,429741	2,37638	1,581679	0,849944	1,518692	0,617611	-0,46727	-0,07107	12,55383	7,863539	9,168931	0,202146	0,38235
NGF_Pathway_Apoptosis	-0,03332	-0,1687	-0,11846	0,15896	0,337863	0,317918	-0,34962	-0,35049	-0,56382	0,758247	1,114342	0,363018	0,928319	0,481563	1,032157	0,504513	-0,18878	0,100538	0,202371	0,382515
PTEN_Pathway_Proliferation_CyclinA2_Geminin_	-0,24167	0,056037	0,07594	0,212859	-1,57776	-2,31862	-1,20972	-1,11136	-1,44191	-0,21808	-0,37892	1,430459	-0,87444	0,419628	1,033586	0,889099	1,181368	-0,18986	0,203154	0,38315
PTEN_Pathway_Senescence_Ets2_p16	-0,24167	0,056037	0,07594	0,212859	-1,57776	-2,31862	-1,20972	-1,11136	-1,44191	-0,21808	-0,37892	1,430459	-0,87444	0,419628	1,033586	0,889099	1,181368	-0,18986	0,203154	0,38315
reactome_Activated_NOTCH1_Transmits_Signal_t	0,304155	-0,04368	-0,18489	-1,8839	-0,08666	-0,72802	0,351691	0,665669	0,14837	-2,51207	0,822894	-0,14007	0,886838	0,907475	0,892594	-4,11424	-1,39204	-1,18257	0,203087	0,38315
reactome_Elevation_of_cytosolic_Ca2_levels_Mai	-0,02305	-0,20464	-0,06831	-0,30712	0,5373	-0,18433	-0,29953	-0,36531	-0,32267	1,374694	0,137952	-0,32923	0,331544	-0,13782	0,079954	1,039237	1,056172	0,4997	0,203199	0,38315
reactome_Thromboxane_signalling_through_TP_	0,010832	-0,20361	-0,29937	1,941017	2,254703	1,086915	-0,59056	-0,90205	-0,66069	-1,74732	1,090267	0,729334	-0,86628	-0,69281	-0,42551	-1,6735	-1,43348	0,251785	0,203355	0,383213
KEGG_Vasopressin_regulated_water_reabsorptio	-0,28123	-0,30568	-0,3622	-0,47418	-0,48279	-1,37662	0,073997	0,264532	0,335745	-0,78533	-0,29536	0,766059	-0,62377	-0,47831	0,610823	-0,39542	0,351245	1,116034	0,203941	0,384084
ATM_Pathway_NF-kB_Pathway	0,061021	0,010379	-0,01444	0,481122	0,103658	0,183026	0,180701	0,268175	0,111708	-0,03189	0,405407	-0,20297	-0,25473	0,03879	-0,20848	-0,77259	-0,45954	-0,40245	0,205161	0,385421
KEGG_Insulin_signaling_Main_Pathway	-0,75261	-0,63782	-1,03059	0,060914	-0,18229	-2,87613	-2,42101	-2,38307	-2,89829	0,011052	1,096915	1,515045	1,36669	0,224515	1,840209	-7,15875	-3,69895	-2,0489	0,205689	0,385421
KEGG_Pancreatic_cancer_Main_Pathway	-0,15151	-0,17211	-0,45051	0,313019	0,68445	0,988986	-2,22741	-1,89786	-3,47728	1,203282	2,130344	3,212774	0,649492	0,71523	0,770538	-5,8674	-3,0564	-2,23044	0,205056	0,385421
NCI_LPA_receptor_mediated_events_Pathway_(c	-0,07628	-0,01632	-0,10905	-0,36749	-0,48196	-0,49423	0,397337	0,381775	0,271305	-0,91257	0,29359	-0,08295	0,1838	-0,10292	0,000332	-1,86104	-1,31494	-1,10967	0,20479	0,385421
NCI_N_cadherin_signaling_events_Pathway_(axor	-0,06783	-0,08878	-0,03408	0,181768	0,818098	0,08342	-0,50174	-0,59219	-0,69332	0,421355	0,876944	0,364292	-0,14101	-0,14965	-0,08199	-2,12276	-1,52012	-1,20532	0,206506	0,385421
NCI_N_cadherin_signaling_events_Pathway_(carc	-0,06783	-0,08878	-0,03408	0,181768	0,818098	0,08342	-0,50174	-0,59219	-0,69332	0,421355	0,876944	0,364292	-0,14101	-0,14965	-0,08199	-2,12276	-1,52012	-1,20532	0,206506	0,385421
NCI_N_cadherin_signaling_events_Pathway_(cort	-0,06783	-0,08878	-0,03408	0,181768	0,818098	0,08342	-0,50174	-0,59219	-0,69332	0,421355	0,876944	0,364292	-0,14101	-0,14965	-0,08199	-2,12276	-1,52012	-1,20532	0,206506	0,385421
NCI_N_cadherin_signaling_events_Pathway_(end	-0,06783	-0,08878	-0,03408	0,181768	0,818098	0,08342	-0,50174	-0,59219	-0,69332	0,421355	0,876944	0,364292	-0,14101	-0,14965	-0,08199	-2,12276	-1,52012	-1,20532	0,206506	0,385421
NCI_N_cadherin_signaling_events_Pathway_(long	-0,06783	-0,08878	-0,03408	0,181768	0,818098	0,08342	-0,50174	-0,59219	-0,69332	0,421355	0,876944	0,364292	-0,14101	-0,14965	-0,08199	-2,12276	-1,52012	-1,20532	0,206506	0,385421
NCI_N_cadherin_signaling_events_Pathway_(prot	-0,06783	-0,08878	-0,03408	0,181768	0,818098	0,08342	-0,50174	-0,59219	-0,69332	0,421355	0,876944	0,364292	-0,14101	-0,14965	-0,08199	-2,12276	-1,52012	-1,20532	0,206506	0,385421
NCI_N_cadherin_signaling_events_Pathway_(regu	-0,06783	-0,08878	-0,03408	0,181768	0,818098	0,08342	-0,50174	-0,59219	-0,69332	0,421355	0,876944	0,364292	-0,14101	-0,14965	-0,08199	-2,12276	-1,52012	-1,20532	0,206506	0,385421
NCI_N_cadherin_signaling_events_Pathway_(regu	-0,06783	-0,08878	-0,03408	0,181768	0,818098	0,08342	-0,50174	-0,59219	-0,69332	0,421355	0,876944	0,364292	-0,14101	-0,14965	-0,08199	-2,12276	-1,52012	-1,20532	0,206506	0,385421
NCI_N_cadherin_signaling_events_Pathway_(syna	-0,06783	-0,08878	-0,03408	0,181768	0,818098	0,08342	-0,50174	-0,59219	-0,69332	0,421355	0,876944	0,364292	-0,14101	-0,14965	-0,08199	-2,12276	-1,52012	-1,20532	0,206506	0,385421
reactome_Meiotic_recombination_Main_Pathwa	0,6918	0,023792	-0,03401	2,203436	-0,19794	-0,5167	8,326969	9,341505	6,470394	2,73511	1,988063	-0,98675	-0,64395	-0,57366	0,41459	-9,68589	-5,57517	-6,26386	0,206256	0,385421
reactome_Pentose_phosphate_pathway_hexose_	0,148083	-0,12212	-0,07805	0,232029	-0,48423	-1,49431	-0,1077	-0,13732	-0,42764	-0,46287	-0,75385	-0,93807	0,121161	-0,1076	0,149948	-1,53415	-1,08036	-1,75622	0,205574	0,385421
bile_acid_biosynthesis_neutral_pathway	-0,27433	-0,17483	0,03221	-2,34321	0,355515	-0,25436	-1,35056	-1,52267	-1,04384	1,329842	1,6515	0,822128	0,588987	-0,92395	0,189983	-0,01911	-0,62217	0,011246	0,20781	0,387621
NCI_Lissencephaly_gene_LIS1_in_neuronal_migra	0,320213	-0,25604	-0,09212	-0,19354	-1,04476	-0,15625	-2,16036	-1,86417	-2,78319	1,73068	-0,55183	-0,46392	0,038443	0,5726	0,79314	-3,0914	-2,54904	-1,81634	0,207947	0,387645
NCI_Alpha4_beta1_integrin_signaling_events_Pat	-0,19773	-0,08093	-0,31581	-1,52423	-2,28949	-2,28864	-1,90588	-1,65035	-1,95418	1,993212	1,097385	0,657545	0,53916	0,437032	0,455597	-0,79712	-0,80086	-0,50218	0,208148	0,387788
IL-2_Pathway	-2,14774	-0,72564	-1,65134	-3,13161	-0,3728	-2,87538	-6,27915	-5,90939	-7,22598	-0,21477	1,910815	4,565612	1,45694	1,236852	3,260545	-12,6522	-8,93689	-5,58884	0,208368	0,387966
reactome_Cytosolic_tRNA_aminoacylation_Main_	0,779439	0,072595	0,207667	-1,70739	-1,31981	-1,26301	-2,24508	-1,83143	-3,58611	0,360573	1,11037	1,431594	0,292192	0,382041	0,378509	-14,2767	-11,1394	-9,49268	0,208626	0,388215
ILK_Signaling_Pathway_MMP2_MMP9_Gene_Exp	-0,55335	-0,56003	-0,76067	-1,74348	-0,21904	-1,72518	-3,0223	-2,69591	-3,48747	-0,33	-0,49238	0,218372	0,547391	0,240798	0,147942	-3,40067	-3,24134	-2,28747	0,208903	0,388265
ILK_Signaling_Pathway_Tissue_Morphogenesis	-0,55335	-0,56003	-0,76067	-1,74348	-0,21904	-1,72518	-3,0223	-2,69591	-3,48747	-0,33	-0,49238	0,218372	0,547391	0,240798	0,147942	-3,40067	-3,24134	-2,28747	0,208903	0,388265
cAMP_Pathway_Regulation_of_Cytoskeleton	-0,07163	0,055702	0,064426	-0,29633	0,884969	0,906324	-1,13426	-1,07402	-1,00347	-0,54189	0,735642	0,272147	-0,46245	-0,1777	-0,11681	-1,84456	-0,85698	-0,99538	0,210439	0,389726
NCI_IL4_mediated_signaling_events_Pathway_(T_	-0,01555	-0,0012	-0,00137	-0,02657	0,026123	-0,04422	-0,12236	-0,16283	-0,01161	0,000622	0,173946	-0,01282	0,15705	-0,04839	0,060152	0,226058	0,198402	0,194562	0,2102	0,389726
NCI_IL4_mediated_signaling_events_Pathway_(T_	-0,01555	-0,0012	-0,00137	-0,02657	0,026123	-0,04422	-0,12236	-0,16283	-0,01161	0,000622	0,173946	-0,01282	0,15705	-0,04839	0,060152	0,226058	0,198402	0,194562	0,2102	0,389726
NCI_IL4_mediated_signaling_events_Pathway_(T_	-0,01555	-0,0012	-0,00137	-0,02657	0,026123	-0,04422	-0,12236	-0,16283	-0,01161	0,000622	0,173946	-0,01282	0,15705	-0,04839	0,060152	0,226058	0,198402	0,194562	0,2102	0,389726
NCI_Integrin_linked_kinase_signaling_Pathway_(I	0,049729	-0,00249	0,013205	0,656602	1,006292	0,260586	-2,02776	-2,18081	-2,33223	0,285557	2,114393	1,491658	0,221814	0,550022	0,104746	-2,45817	-2,38371	-1,57312	0,209967	0,389726
reactome_Signaling_by_NODAL_Main_Pathway	-0,17631	0,288715	0,235011	0,267066	-0,20075	-0,07793	-1,77624	-1,87567	-1,66118	0,30377	-0,42789	-1,48306	0,212997	0,236333	0,371534	0,223442	-0,13157	-0,11448	0,210346	0,389726
reactome_VEGF_binds_to_VEGFR_leading_to_rec	0,046211	0,04796	0,025342	-0,2625	0,020972	0,268834	-0,51528	-0,61163	-0,41387	0,887624	0,032031	-0,31479	0,185137	0,100483	-0,01787	0,590222	0,604611	0,273655	0,210569	0,389734
reactome_HATs_acetylate_histones_Main_Pathw	0,653181	-0,36293	-0,79481	-1,72156	-1,37703	-3,8016	5,975545	7,571185	3,281165	-0,83389	2,983765	-1,09653	-0,53196	0,898418	1,598921	-14,8012	-10,8773	-8,36849	0,211008	0,390314
p38_Signaling_Pathway_Actin_Cytoskeleton_Reo	-0,04767	-0,16118	-0,19803	-0,35015	-0,50395	0,035341	-0,16122	-0,09804	-0,23169	0,114391	0,336459	0,19861	0,280161	0,142957	0,428469	-1,43723	-0,82995	-0,8415	0,211206	0,390449
Chemotaxis_of_Immune_Cells_During_Allergic_A	-0,25032	-0,491	-0,55967	3,356645	2,623927	0,176382	-2,72989	-3,07626	-2,30945	0,472498	2,503598	2,203195	0,011038	0,649144	0,356964	-1,31866	-1,04626	0,43999	0,211601	0,390947
biocarta_epo_signaling_Main_Pathway	-0,2088	-0,16422	-0,07888	-1,0856	-0,35191	-0,56015	-0,04207	0,003823	0,064148	0,812388	0,304793	-0,02498	-0,47979	-0,96964	-0,54551	-1,82951	-0,72205	-0,7751	0,211752	0,390994
L-glutamine_tRNA_biosynthesis	0,152062	0,051148	0,046071	-0,21759	-0,18323	-0,41868	-0,07167	0,082755	-0,19198	-0,65719	-0,65951	-0,0744	-0,02683	-0,04551	0,067888	-0,72098	-0,46687	-0,54323	0,212601	0,392329
biocarta_rac1_cell_motility_signaling_Main_Path	-0,282	-0,26496	-0,07314	-1,82911	-1,96837	-0,73593	-2,59364	-2,80127	-2,92011	0,065076	0,474354	1,628207	1,052281	1,416712	1,110879	2,071074	1,339319	2,404212	0,212876	0,392604
KEGG_Non_homologous_end_joining_Main_Path	0,449026	0,02836	0,211084	0,187311	0,274457	0,313797	-0,84654	-0,4847	-1,44598	-0,18594	0,489375	0,006617	-0,61776	-0,20316	0,193718	-2,44514	-1,83657	-1,03967	0,213337	0,393191

pyrimidine_deoxyribonucleotide_phosphorylation	0,017651	0,09022	-0,02959	-0,60489	-0,05005	-0,04782	0,26338	0,285942	-0,30003	1,133401	1,898846	0,624391	0,743978	0,801836	0,80084	-3,76696	-3,50896	-2,89237	0,213573	0,393191
reactome_Negative_regulation_of_FGFR_signalin	-0,50057	-0,42472	-0,17657	-1,00721	0,292069	-0,37594	-0,75479	-1,09938	0,268608	-0,53627	-1,11921	-0,01438	-0,8272	-0,43224	-0,74514	-2,47809	-1,82544	-1,53631	0,213568	0,393191
NCI_IL8_and_CXCR2_mediated_signaling_events_	-0,05152	-0,11537	-0,15965	-0,3173	0,066418	-0,19784	-2,89672	-2,9654	-2,66484	-0,34949	0,29927	-1,01939	-0,21828	-0,41317	0,045941	-2,30657	-0,52697	-0,51408	0,21384	0,393451
biocarta_erk_and_pi_3_kinase_are_necessary_fo	0,332011	0,231039	0,390831	0,567814	-0,46342	1,133982	1,652869	1,542931	1,462657	-0,35414	0,104682	0,170069	-1,63077	-1,01847	-1,46591	-0,30623	0,315038	0,743018	0,214415	0,394258
NCI_Signaling_events_mediated_by_VEGFR1_and	-0,02093	0,013017	-0,02333	0,0945	0,129041	-0,3016	0,137274	0,13941	0,102089	-0,14714	-0,35538	-0,4313	0,097816	0,009791	0,008174	0,044292	0,022822	-0,0702	0,214532	0,394258
estradiol_biosynthesis_I	0,187201	0,054171	0,126432	0,434679	0,257008	0,252426	0,058354	0,109339	-0,00122	0,168066	0,035891	0,505376	-0,12333	-0,03977	-0,32968	-0,84029	-1,29049	-1,33295	0,214943	0,394548
NCI_FGF_signaling_Pathway_(cell_migration)	0,069932	0,090218	0,090594	0,368847	-0,07678	0,438678	0,637585	0,598159	0,568474	-0,07382	-0,46638	-0,21858	0,029833	0,168065	0,142601	0,138847	0,248812	0,367131	0,214882	0,394548
reactome_Gap_junction_assembly_Main_Pathwa	-0,47311	-0,11829	-0,46552	0,552271	1,20886	-0,79855	0,416355	-0,23204	1,965146	0,433211	0,610968	0,551624	0,270545	0,332742	0,038879	3,161193	2,443781	2,552191	0,215515	0,395364
Akt_Signaling_Pathway_Blood_cell_differentiatio	-0,01381	-0,11453	0,251415	-1,17442	0,507388	-1,56755	0,164284	0,033892	0,35442	0,560983	1,620332	0,462987	0,658744	0,623633	0,277838	0,147237	0,750449	0,730538	0,215999	0,395444
biocarta_influence_of_ras_and_rho_proteins_on	0,193454	-0,27161	-0,15541	-0,45963	0,098177	-0,24926	-0,96977	-0,55205	-1,48923	0,589248	0,836903	0,105519	0,065325	-0,18332	0,300328	-0,3843	-0,05524	0,638285	0,216999	0,395444
biocarta_lissencephaly_gene_lis1_in_neuronal_m	0,004719	0,012634	0,003382	-0,22169	-1,50251	0,22208	-1,03371	-1,01128	-1,88776	0,358162	-0,00648	0,168905	0,445678	0,840014	0,921599	-1,86185	-1,53512	-1,67952	0,216796	0,395444
ILK_Signaling_Pathway_Cell_Adhesion	-0,0463	-0,04276	-0,07755	-0,36632	-0,56509	-0,48402	-0,844	-0,75718	-0,88829	0,455757	0,098799	0,775945	0,006892	0,063092	0,127964	-0,44086	-0,24238	-0,2411	0,216969	0,395444
ILK_Signaling_Pathway_Regulation_of_Junction_A	-0,0463	-0,04276	-0,07755	-0,36632	-0,56509	-0,48402	-0,844	-0,75718	-0,88829	0,455757	0,098799	0,775945	0,006892	0,063092	0,127964	-0,44086	-0,24238	-0,2411	0,216969	0,395444
ILK_Signaling_Pathway_Wound_Healing	-0,0463	-0,04276	-0,07755	-0,36632	-0,56509	-0,48402	-0,844	-0,75718	-0,88829	0,455757	0,098799	0,775945	0,006892	0,063092	0,127964	-0,44086	-0,24238	-0,2411	0,216969	0,395444
KEGG_Sphingolipid_metabolism_Main_Pathway	-0,27534	-0,53848	-0,25836	0,022748	-0,0204	-1,49078	-5,47764	-4,9355	-5,61256	-1,62288	-1,36835	-0,06877	-0,83958	-0,83893	-0,65938	-2,75388	-3,10937	-0,8708	0,215774	0,395444
Mitochondrial_Apoptosis_Pathway_Apoptosis	-0,03787	-0,03901	0,00699	-0,16931	-0,18527	-0,79039	-0,16092	-0,14945	-0,15324	0,164977	0,814943	-0,08952	0,711569	-0,26753	0,266953	-2,5912	-2,66372	-1,92911	0,216115	0,395444
NCI_Glypican_1_network_Pathway_(cell_growth)	-0,20475	0,137654	-0,1976	-0,61144	-1,16519	-1,14229	-2,32124	-2,38528	-1,98771	1,45482	0,571825	-0,71916	0,043868	0,489345	0,644621	-0,02816	0,902599	0,141232	0,217412	0,395444
NCI_Glypican_1_network_Pathway_(fibroblast_gr	-0,20475	0,137654	-0,1976	-0,61144	-1,16519	-1,14229	-2,32124	-2,38528	-1,98771	1,45482	0,571825	-0,71916	0,043868	0,489345	0,644621	-0,02816	0,902599	0,141232	0,217412	0,395444
NCI_Syndecan_4_mediated_signaling_events_Pat	-0,05723	-0,26649	-0,0583	0,640766	0,38709	0,869915	-0,05059	0,04249	-0,21673	-0,28227	-0,11835	-0,18049	-0,68325	-0,52933	-0,03571	-0,62988	0,06911	-0,00913	0,217139	0,395444
reactome_Activated_point_mutants_of_FGFR2_M	-0,54665	-0,24509	-0,14265	-0,70153	0,434572	-0,13915	0,372055	-0,03051	1,090906	0,990009	-1,35842	-0,41159	-0,92111	-0,94507	-0,77745	0,50599	0,334456	0,010869	0,217462	0,395444
reactome_Interleukin_1_signaling_Main_Pathway	0,514558	-0,22435	-0,10019	-0,68624	-1,18062	-1,81931	-2,14231	-1,84044	-3,50235	-1,0476	-2,05744	-1,74003	-0,27218	0,312706	-0,00313	-2,96446	-2,34238	-1,37125	0,216411	0,395444
reactome_Senescence_Associated_Secretory_Phe	0,439014	-0,41969	-0,80399	-1,33083	-4,05997	-4,91606	1,468819	2,190447	-0,28914	-3,67137	-1,64694	-0,68348	-0,37787	-0,08445	1,417913	-10,822	-8,95726	-7,7381	0,216341	0,395444
reactome_Synthesis_of_15_eicosatetraenoic_acic	-0,00925	0,063926	-0,08162	-0,12464	-0,18094	0,122177	-0,28136	-0,25296	-0,13788	0,170033	0,005135	0,650204	-0,12739	-0,26512	0,046944	-0,08377	-0,06339	-0,23955	0,216705	0,395444
reactome_Molecules_associated_with_elastic_fib	-1,00708	-0,34112	-0,64795	0,202478	-0,89761	-0,26463	1,042226	0,895543	1,162495	0,378027	-0,33127	-0,13743	-0,25345	-0,31236	0,066635	1,730042	1,375206	0,919977	0,217664	0,395579
beta-alanine_degradation	-0,02157	0,006869	-0,0466	-0,16784	-0,08947	0,027934	0,514861	0,597244	0,380718	0,079937	0,113741	-0,00751	-0,07709	-0,10481	-0,03904	-0,13471	-0,2308	0,102924	0,218862	0,396028
biocarta_role_of_ppar_gamma_coactivators_in_c	-0,09487	0,040076	-0,04891	-0,74475	-0,55923	-0,06483	-0,09334	-0,07535	-0,10433	-0,54032	-0,53884	-0,83756	0,242127	0,3985	0,272613	-0,89383	-0,26922	-0,19079	0,218928	0,396028
biocarta_role_of_ppar_gamma_coactivators_in_c	-0,09487	0,040076	-0,04891	-0,74475	-0,55923	-0,06483	-0,09334	-0,07535	-0,10433	-0,54032	-0,53884	-0,83756	0,242127	0,3985	0,272613	-0,89383	-0,26922	-0,19079	0,218928	0,396028
KEGG_Inositol_phosphate_metabolism_Main_Pat	-0,22796	-0,88978	-0,64133	-0,88377	-2,37327	-1,55478	-2,17386	-1,55031	-3,76939	-0,9073	-0,92041	-2,45577	-1,30816	-0,60053	0,464953	-4,8086	-3,66274	-2,45588	0,218294	0,396028
KEGG_Nitrogen_metabolism_Main_Pathway	0,05479	-0,0195	0,056921	-0,23187	-0,16622	-0,02145	0,705537	0,820604	0,679245	-0,2544	0,512791	0,015195	-0,10624	-0,04572	0,343137	-1,68033	-0,88748	-1,05143	0,218629	0,396028
KEGG_Ubiquinone_and_other_terpenoid_quinon	0,215318	0,119892	0,079585	0,118699	0,147117	-0,10342	0,517179	0,601254	0,187443	-0,11053	0,821878	-0,16963	-0,04043	0,149141	-0,19147	-1,37187	-1,16969	-1,9065	0,218079	0,396028
reactome_Retrograde_neurotrophin_signalling_M	-0,01316	-0,12631	0,033038	-0,3449	-0,01661	-0,88751	-2,13927	-2,24414	-2,32236	-0,28997	0,885842	0,724022	0,483984	-0,00719	0,758326	0,261054	0,833114	1,171153	0,21867	0,396028
superpathway_of_cholesterol_biosynthesis	0,429918	0,287924	0,004873	2,191999	1,649916	3,429732	2,679151	2,687081	2,090887	0,663985	2,625731	3,291173	-0,92502	-0,74332	-0,87474	-7,9616	-5,09862	-4,4737	0,218717	0,396028
PLAU-mediated_Signaling_Events_During_Cell_Ac	-0,38804	-0,6094	-0,73584	-1,77663	-1,38287	-0,81113	-0,21458	0,144845	-0,92136	0,527421	0,171315	1,526771	0,080383	0,316313	1,050344	-7,68056	-6,12184	-4,17308	0,219433	0,396711
biocarta_calcium_signaling_by_hbx_of_hepatitis	-0,22292	-0,49329	-0,42234	-0,18485	-0,20481	-0,1225	-0,9453	-0,82144	-1,10838	-1,24083	0,521973	0,745528	-0,42115	-0,8089	-0,55385	-0,49705	0,839933	0,319135	0,220028	0,397507
reactome_Gluconeogenesis_Main_Pathway	-0,09966	0,037884	0,025925	-1,44069	-1,2888	-2,37081	0,377005	0,485558	0,109686	-0,14741	-1,58554	-0,91837	-0,09465	-0,3431	0,500667	-5,28345	-5,59131	-4,72689	0,220128	0,397507
HGF_Pathway_Cell_Scattering	-0,41976	-0,3654	-0,33744	-0,47917	0,109033	0,114473	-0,65748	-0,19308	-1,05788	1,473515	0,655982	1,745188	0,061585	0,267255	0,42893	-4,54013	-3,84727	-2,74891	0,220389	0,397517
HGF_Pathway_Cell_Survival	-0,41976	-0,3654	-0,33744	-0,47917	0,109033	0,114473	-0,65748	-0,19308	-1,05788	1,473515	0,655982	1,745188	0,061585	0,267255	0,42893	-4,54013	-3,84727	-2,74891	0,220389	0,397517
ATM_Pathway_Apoptosis	-0,11861	-0,06702	-0,13591	0,265807	0,184032	0,316706	0,295141	0,419961	0,216014	-0,00771	0,218205	0,058075	-0,10032	-0,1323	-0,15406	-0,3044	-0,1891	-0,08815	0,221445	0,399191
NCI_DNA_PK_pathway_in_nonhomologous_end	0,431139	0,01615	0,185841	0,430946	0,045662	0,338582	-0,81156	-0,53043	-1,3001	0,156549	0,093882	-0,46566	-0,62372	-0,23092	0,195345	-1,69299	-1,27712	-0,57387	0,221905	0,399788
reactome_Ion_transport_by_P_type_ATPases_M	-0,36045	0,53048	-0,05649	1,412309	2,839254	2,273557	-0,90043	-1,09825	-0,83321	-1,19078	-1,37867	0,744467	1,811057	2,35074	1,875549	3,072945	3,998227	4,486265	0,222655	0,400907
cAMP_Pathway_Gene_Expression_via_NFKB2_CR	-0,52488	-0,47868	-0,29999	-0,37977	0,14818	-0,52839	-2,32206	-2,00244	-2,69089	-3,03919	0,447667	1,586889	-0,38317	-0,25162	0,466238	-1,77012	-2,53562	-0,7039	0,223292	0,401823
NCI_Signaling_events_mediated_by_Stem_cell_fa	0,037936	0,042226	0,073119	-0,30404	-0,4594	-0,44638	-0,16076	-0,15301	-0,44546	-0,27195	-0,36323	-0,28137	-0,11214	-0,33649	-0,00205	-1,39289	-2,27577	-1,49438	0,223546	0,40197
NCI_VEGFR3_signaling_in_lymphatic_endotheliun	-0,05306	-0,04487	0,126791	-0,08998	-0,15703	0,255268	-0,60441	-0,73196	-0,59178	0,113799	0,309204	-0,01158	0,181921	0,017689	-0,06694	-0,89618	-0,45616	-0,56953	0,223632	0,40197
biocarta_role_of_pi3k_subunit_p85_in_regulatior	0,094247	-0,13353	-0,12464	-0,10871	-1,04814	-0,17117	-0,73396	-0,59592	-1,04527	-0,77337	0,251595	-0,13703	-0,47902	-0,41087	-0,21309	-3,80432	-3,05932	-2,01422	0,223852	0,402133
biocarta_regulation_of_ck1_cdk5_by_type_1_glu	-0,03599	0,016137	-0,15888	0,55621	0,684657	-0,60616	0,66731	0,482119	0,426966	1,801505	0,904481	0,682939	1,036158	0,734885	0,855673	2,305024	1,785639	2,354067	0,224222	0,402334
KEGG_Progesterone_mediated_oocyte_maturatio	0,207289	0,176727	-0,118	-1,17104	-1,6499	-2,2239	0,704497	0,94398	-0,11749	-1,27003	-2,43983	-2,305	-0,58485	-0,42297	0,531844	-4,54688	-3,50016	-2,37236	0,224141	0,402334
biocarta_rac1_cell_motility_signaling_Pathway_(	-0,03912	-0,13185	-0,0315	-0,41469	-0,09967	-0,339	-0,3646	-0,36929	-0,49503	-0,20222	-0,04952	0,100155	0,317771	0,387515	0,224084	0,804749	1,09247	1,367207	0,224856	0,40272
NCI_ATM_Pathway_(Pathway_rRNA_transcriptior	-0,02089	-0,21978	-0,22112	-0,12921	0,534377	0,22079	0,197396	0,378463	-0,21592	-0,41746	0,126407	0,519164	-0,22517	-0,33538	-0,2442	-1,3342	-0,60964	-0,65652	0,225371	0,40272
NCI_RAC1_signaling_Pathway_(positive_regulatio	-0,02459	0,049092	-0,06208	-0,19688	-0,01506	-0,11315	-0,25465	-0,2147	-0,34987	0,062513	0,124676	0,263786	0,240871	0,068834	0,177278	-0,95052	-0,75134	-0,24639	0,224577	0,40272
NCI_Stabilization_and_expansion_of_the_E_cadh	0,067669	0,071368	0,058191	0,318874	0,123146	-0,26453	-0,8281	-0,7148	-1,15109	0,348332	0,615035	0,603843	-0,40155	-0,01641	0,039008	-3,1949	-2,58637	-2,48919	0,225471	0,40272
NCI_Thromboxane_A2_receptor_signaling_Pathw	-0,10794	0,036314	0,024808	0,004055	-0,13966	-0,40986	-0,2603	-0,40376	-0,13109	-1,09947	-0,00453	0,018561	-0,10131	-0,28095	-0,16394	-1,17795	-0,86434	-0,58013	0,22536	0,40272
NCI_Validated_transcriptional_targets_of_AP1_fa	-0,13105	-0,1126	-0,20336	0,027838	-0,1225	-0,16197	-0,31502	-0,27412	-0,27757	-0,15383	-0,07631	0,568137	-0,0923	0,099812	0,168345	-0,54053	-0,5972	-0,31	0,22478	0,40272
reactome_Acyl_chain_remodelling_of_PI_Main_F	-0,18152	0,094117	-0,04675	-0,86539	-1,38951	-1,64879	-0,34559	-0,52407	0,281835	-1,40399	-1,28101	-0,10117	-0,12777	-0,08834	-0,2062	0,225168	0,243421	0,01322	0,225285	0,40272
reactome_FGFR2c_ligand_binding_and_activatior	-0,39989	-0,19959	-0,03848	-0,64062	-0,11678	-0,63306	-0,63399	-0,99368	-0,04396	0,482779	-0,78948	-0,06711	-0,27919	-0,22615	-0,33524	-0,13997	0,223989	-0,60399	0,225453	0,40272
NCI_IGF1_Pathway_(positive_regulation_of_MAP	0,018142	-0,02623	-0,01184	0,034353	0,607254	0,475789	-0,34673	-0,50654	-0,512	0,702149	0,460685	0,090979	0,25219	0,137001	0,12761	-0,55665	-0,02535	0,107545	0,22563	0,402772
reactome_Toll_Like_Receptor_4_TLR4_Cascade_I	-0,24595	-0,39292	-0,26266	-1,3776	-1,50928	-1,58577	-3,5526	-3,08007	-3,86341	0,358202	1,223992	0,363221	-0,30559	-0,77952	-0,38596	-2,00608	-1,54986	-1,53953	0,225952	0,403116
NCI_Regulation_of_nuclear_SMAD2_3_signaling	-0,32516	-0,22917	-0,48813	-0,51459	-0,55674	0,643895	-3,11953	-3,29633	-3,51318	-0,27479	-0,89124	-0,36171	-0,26885	-0,20814	-0,1693	2,861983	2,037331	1,708256	0,226252	0,40319
p53_Signaling_Pathway	-0,11286	-0,40838	-0,40022	0,62042	0,650679	-0,31996	-2,07881	-1,31102	-3,65835	1,585933	1,83225	2,85445	1,411988	1,115952	1,484721	-3,58321	-1,78949	-0,74508	0,226234	0,40319
NCI_Signaling_events_mediated_by_Stem_cell_fa	-0,06613	-0,03236	0,001231	-0,61989	-0,35823	-0,66543	-0,64236	-0,71925	-0,80289	-0,341	-0,40677	-0,28299	-0,03449	-0,13196	0,077305	-1,19644	-2,09505	-1,34604	0,226394	0,403212
MAPK_Family_Pathway	-3,10537	-2,72269	-1,45174	-10,618	-2,55003	-9,32422	-8,27252	-10,2363	-5,9018	4,557499	-3,3391	-1,49096	2,989393	1,329194	3,512085	3,81077	6,724565	10,49358	0,226889	0,403862
ITGA4-mediated_Cell_Adhesion_and_Migration	0,196593	-0,29697	-0,36419	-0,99032	-2,95629	-1,67513	-0,06442	0,160832	-0,88209	-2,95371	-0,14849	0,112262	-0,29776	-0,34205	-0,14388	-7,66743	-5,8622	-5,56208	0,227731	0,404943
reactome_Nuclear_signaling_by_ERBB4_Main_Pa	0,055036	-0,2171	-0,12308	-0,37445	0,491195	-0,069	0,04834	0,138133	-0,26594	0,281405	0,755232	1,127765	0,272302	0,55812	0,650712	-1,76534	-0,56766	-0,89596	0,227756	0,404943
NCI_ErbB4_signaling_events_Pathway_(epithelial	-0,24426	0,003101	-0,03331	-2,04215	-1,78273	-1,23865	-0,66146	-0,84454	-0,84262	-0,24135	-0,38884	0,074774	0,017704	-0,084	0,141781	-0,81176	-0,57532	-0,27341	0,228085	0,405065
NCI_Fc_epsilon_receptor_I_signaling_in_mast_ce	0,000604	0,075929	0,379844	-0,03375	-1,66013	2,045649	-0,37795	-0,60456	-0,51091	-0,43969	-0,22499	-1,52157	-1,34597	0,291373	-0,49521	-2,76202	-2,23455	-3,16118	0,228054	0,405065
cAMP_Pathway_Lipolysis	-0,1283	-0,01737	-0,033	-0,09856	0,73763	0,322888	-1,10674	-1,0566	-1,05288	-1,24966	0,205002	0,215002	-0,72136	-0,45399	-0,1187	-1,28901	-0,987	-0,59065	0,228608	0,405531
reactome_Classical_antibody_mediated_complen	-0,15631	0,06575	-0,13906	0,314523	-0,01652	0,007671	-0,33937	-0,54451	-0,01561	0,621869	-0,48547	0,453466	0,298792	0,533357	-0,00069	1,952267	1,425888	1,415815	0,228492	0,405531
biocarta_y_branching_of_actin_filaments_Main_I	0,234567	-0,15583	-0,14157	-0,12439	-1,47659	-0,7946	-0,82651	-0,49017	-1,34941	-0,81142	0,775985	-1,01019	-0,69489	-0,55144	-0,30293	-6,33143	-5,44463	-3,37353	0,228784	0,405613
NCI_IGF1_Main_Pathway	-0,03276	-0,1066	-0,32779	-0,40715	1,119234	-0,82249	-1,86858	-1,88362	-2,09578	1,302213	1,289997	1,195959	1,517369	0,926319	1,254956	-2,58351	-1,06456	-0,94255	0,22935	0,406386
reactome_Presynaptic_phase_of_homologous_DI	-0,06926	-0,09448	-0,07915	0,228031	0,075021	0,339914	0,789191	0,931398	0,922568	1,138029	-0,59265	0,396677	0,147843	0,168532	0,152076	-0,38291	-0,31773	-0,42169	0,229672	0,406724
NCI_Integrin_linked_kinase_signaling_Pathway_(	-0,04831	-0,16829	-0,23355	-0,31673	-0,37598	-0,34075	0,038338	0,009735	-0,10658	-0,49309	0,396836	0,649996	-0,09724	0,137099	-0,08006	-2,30952	-2,23142	-1,51372	0,229847	0,406804
reactome_Hypusinylation_Main_Pathway	-0,18287	-0,06327	0,000621	0,105442	-0,13446	-0,33654	-0,26904	-0,15932	-0,23903	0,401553	0,149935	-0,43665	-0,13121	-0,11404	0,144448	-1,26659	-0,76315	-0,69698	0,230498	0,407724
leucine_degradation	0,266419	0,149993	-0,03556	-0,24698	-0,14735	-0,88635	0,096924	0,326391	-0,42694	0,737002	0,697172	-0,0248	0,203715	0,027307	0,437987	-3,66917	-3,47896	-3,10591	0,231226	0,408779
NCI_N_cadherin_signaling_events_Pathway_(acti	-0,06125	-0,08612	-0,13686	-0,15359	0,723693	0,030241	-0,60161	-0,63888	-0,91084	0,726575	0,802925	0,69885	0,080364	-0,19045	0,009802	-3,12543	-2,04662	-1,61643	0,231447	0,408938
biocarta_visceral_fat_deposits_and_the_metabol	-0,00159	0,022476	-0,06966	-0,20336	0,298872	0,180299	0,06949	-0,02574	0,093458	-0,23318	-0,44274	0,190772	-0,16632	0,04664	-0,2486	-0,28826	-0,27049	-0,3576	0,232917	0,411302
PTEN_Pathway_Migration	-0,98985	-0,04152	-0,05325	-2,32026	-2,48352	-0,22879	7,059303	6,9246	8,017753	-1,49832	-6,25354	-5,47027	1,574615	0,297328	0,312977	16,74307	16,68566	12,93001	0,23334	0,411815
KEGG_Ras_signaling_Main_Pathway	-2,59868	-3,38859	-2,4382	-4,4813	-3,38622	-5,2717	-15,446	-15,8572	-15,2827	-3,87528	-7,47805	-0,09955	2,863632	3,362836	4,740845	-7,08786	-2,69992	2,192027	0,233998	0,412742
NCI_ErbB2_ErbB3_signaling_events_Pathway_(ap	0,071526	0,07814	-0,0385	-0,49264	0,108923	-0,27727	-0,0908	-0,00514	-0,4142	0,094299	-0,0261	0,462399	0,159434	0,07002	0,220223	-2,08827	-1,26723	-1,06584	0,235273	0,414522
NCI_Nephrin_Neph1_signaling_in_the_kidney_po	0,071526	0,07814	-0,0385	-0,49264	0,108923	-0,27727	-0,0908	-0,00514	-0,4142	0,094299	-0,0261	0,462399	0,159434	0,07002	0,220223	-2,08827	-1,26723	-1,06584	0,235273	0,414522
guanosine_deoxyribonucleotides_ide_novoi_bios	0,226424	0,037244	-0,09481	-0,44676	-0,38438	0,056651	0,492484	0,672782	-0,1474	1,059459	2,037838	0,714404	0,664428	0,615965	0,976321	-4,31746	-3,38548	-2,93378	0,235937	0,415457
NCI_FAS_CD95_signaling_Pathway_(cell_migratio	-0,07203	-0,04084	-0,19272	-0,36414	0,784835	-0,07219	-0,18884	-0,19755	-0,26423	0,609194	0,022992	0,686277	0,071765	0,160851	0,184763	-1,6186	-0,9275	-0,52277	0,236284	0,415833
NCI_CXCR3_mediated_signaling_events_Main_Pa	-0,10195	-0,26233	-0,26789	-0,24089	1,030484	-0,59592	-1,40279	-0,83029	-2,28898	0,868349	2,607303	0,133772	-0,4383	0,309651	1,281197	-4,62449	-2,01003	-3,39586	0,23685	0,416594
biocarta_g_secretase_mediated_erbb4_signaling	0,038418	-0,07605	0,201459	-0,46851	-1,51984	-2,09233	-0,10896	0,025641	-0,19775	-0,17124	-0,44904	-1,47404	0,479987	0,262519	0,470137	1,539131	1,186945	1,117234	0,237174	0,416897
reactome_Na_dependent_glucose_transporters_	0,056638	0,07656	0,031088	0,560261	-0,30673	0,531	0,006717	-0,04325	0,473496	-0,64912	-0,23716	-1,04645	0,385361	0,006156	-0,05129	0,612729	0,203043	0,186666	0,23729	0,416897
NCI_N_cadherin_signaling_events_Pathway_(regu	0,064181	-0,06976	-0,04467	-0,12078	0,980845	-0,10357	-1,04164	-0,94456	-1,36121	0,841156	1,055985	0,697736	-0,06751	-0,3642	-0,30243	-4,47939	-3,3084	-2,89986	0,237517	0,41706
biocarta_map_kinase_inactivation_of_smrt_corep	0,198917	-0,08804	-0,10272	-0,91235	-1,22067	-0,58146	-0,04486	0,235682	-0,43403	-1,33632	0,326639	-1,36091	-0,25292	-0,02075	0,226791	-3,35135	-2,12477	-1,53889	0,237868	0,417383
NCI_Effects_of_Botulinum_toxin_Main_Pathway	-0,12797	-0,12963	-0,17692	-0,29429	-0,22814	-0,15444	-0,6767	-0,64557	-0,69805	-0,61705	-0,2069	-0,38219	0,540573	0,260967	0,172305	2,595787	2,181962	3,083152	0,23837	0,417383
NCI_IL12_signaling_mediated_by_STAT4_Pathwa	0,035023	-0,0613	0,073115	0,32895	0,076979	0,068208	-0,16214	-0,06527	0,047963	-0,34019	-0,1756	-0,15913	-0,07345	0,101914	0,245658	0,435168	0,418776	-0,36667	0,238349	0,417383
NCI_IL12_signaling_mediated_by_STAT4_Pathwa	0,035023	-0,0613	0,073115	0,32895	0,076979	0,068208	-0,16214	-0,06527	0,047963	-0,34019	-0,1756	-0,15913	-0,07345	0,101914	0,245658	0,435168	0,418776	-0,36667	0,238349	0,417383
NCI_IL12_signaling_mediated_by_STAT4_Pathwa	0,035023	-0,0613	0,073115	0,32895	0,076979	0,068208	-0,16214	-0,06527	0,047963	-0,34019	-0,1756	-0,15913	-0,07345	0,101914	0,245658	0,435168	0,418776	-0,36667	0,238349	0,417383
biocarta_nf_kb_signaling_Main_Pathway	0,144591	-0,12324	0,09627	-1,17139	-1,24778	-2,92005	-4,4699	-4,03442	-4,64353	1,558078	-0,52375	1,954806	-0,06875	-0,91929	-0,71258	-2,14422	-2,39981	-1,93014	0,240001	0,420003
NCI_Arf6_trafficking_events_Pathway_(calcium_i	-0,05741	-0,17209	-0,09454	0,116429	-0,41848	-0,75684	-0,81641	-0,92804	-1,31722	1,017874	0,28522	0,608165	0,348388	0,449792	0,341901	-2,92793	-2,17684	-1,99788	0,241114	0,421713
FAK_Pathway	0,407814	-0,77785	-1,03808	-1,2472	-0,28545	-1,73467	-2,61608	-1,86571	-5,19981	-0,04206	2,890047	1,281754	1,20999	1,38949	2,444251	-8,53207	-4,78384	-1,39465	0,241452	0,422066
biocarta_ctcf_first_multivalent_nuclear_factor_M	-0,32428	-0,41519	-0,30931	-0,36985	-0,40493	-0,55219	0,70816	0,548275	-0,19081	0,46326	0,551363	0,572149	0,830357	1,007309	0,883856	-1,77604	-1,29449	0,085173	0,241644	0,422166
Akt_Signaling_Pathway_Genetic_Stability	-0,05332	-0,18421	0,256345	-1,5636	-0,07065	-1,6985	-0,08558	-0,18031	0,129796	0,350055	1,383576	-0,0814	0,728481	0,719772	0,362431	0,119209	0,740366	0,780329	0,242532	0,423033
biocarta_bone_remodeling_Pathway_(osteoclast	-0,35472	-0,23944	-0,22463	0,463041	0,58668	0,411102	-0,64431	-0,93904	-0,43093	-0,20059	-0,7083	0,219846	-0,13084	-0,18641	0,155853	0,607725	0,354382	0,353673	0,242453	0,423033
KEGG_Glyoxylate_and_dicarboxylate_metabolism	0,098824	-0,03818	-0,049	-0,99272	0,07846	-1,49164	0,552219	0,276012	0,54177	1,002843	0,736453	2,835202	-0,24409	-0,4854	-0,49697	-2,02043	-0,98248	-1,20457	0,242548	0,423033
reactome_SHC1_events_in_ERBB4_signaling_Mai	-0,05668	-0,06921	0,310593	-1,45074	-1,28672	-1,89652	-1,94593	-1,92175	-1,71339	0,526553	0,991421	-0,66018	-0,14171	0,179089	-0,26624	-1,20162	-1,19016	-1,09103	0,242922	0,423448
phosphatidylethanolamine_biosynthesis_II	0,081515	-0,06615	0,039462	-0,27121	0,42278	-0,61271	-0,27117	-0,09575	-0,39894	0,154111	-0,01362	0,226426	-0,47628	-0,21123	-0,05598	-0,41738	0,225968	-0,16582	0,243194	0,423515
reactome_PECAM1_interactions_Main_Pathway	-0,1791	-0,03792	0,110568	-0,09395	-0,35823	-0,13452	-1,45086	-1,71091	-1,7203	-0,571	0,647386	-0,09014	-0,1118	-0,13181	0,521095	-1,77307	-0,71849	-0,70586	0,243232	0,423515
D-imyoi-inositol_145-trisphosphate_biosynthesis	-0,11105	-0,24022	-0,58613	-0,53916	-0,97334	-1,4861	0,812652	1,007302	0,303718	-1,44725	-0,48836	0,210005	0,495424	0,217516	0,961642	-1,76512	-1,50376	-1,07812	0,244171	0,424911
biocarta_nfat_and_hypertrophy_of_the_heart_	-0,29883	-0,54317	-0,20095	-1,92729	1,659244	1,655554	-2,19906	-2,22056	-1,9113	-1,08548	0,123406	-0,42806	0,426749	-0,24255	0,340649	-1,06301	-0,53405	0,288303	0,245005	0,426126
VEGF_Pathway_Gene_Expression_and_Cell_Prolif	0,026203	-0,06128	-0,04521	0,350471	-0,09734	-0,62455	-0,47755	-0,07042	-0,86668	-1,80311	0,744663	0,002901	0,220033	0,231977	0,560825	-0,69564	-0,56541	0,151579	0,245199	0,426224
glutathione-mediated_detoxification	-0,46737	0,268558	0,205032	-3,85718	-2,99802	-2,93861	2,002051	2,015028	1,277243	2,18718	0,886664	1,416274	-1,01938	-0,7487	0,355778	-3,37001	-3,80475	-3,19353	0,246265	0,4276
reactome_Extrinsic_Pathway_Main_Pathway	0,116999	0,025918	0,166136	-0,48215	-0,71248	0,464041	0,376316	0,26631	0,451977	-0,54505	-0,51908	0,043193	0,009352	0,20532	-0,07786	-1,31997	-0,66862	-0,9159	0,246352	0,4276
reactome_Processing_of_DNA_ends_prior_to_en	0,123725	0,028412	0,029662	0,48612	-0,02976	-0,27516	0,182775	0,303632	-0,06377	-0,23963	0,104804	-0,53393	-0,23863	-0,17339	-0,26649	-1,51345	-0,56743	-0,4885	0,246402	0,4276
geranylgeranyldiphosphate_biosynthesis	0,028741	-0,03527	-0,02281	0,01264	-0,05806	0,237277	-0,0406	-0,03143	-0,1332	0,145625	0,315315	0,234735	-0,01538	0,023156	0,09042	-0,84351	-0,42491	-0,43373	0,247486	0,429243
Regulation_of_Repulsive_Axonal_Pathfinding	-0,73567	-1,30673	-1,0788	-2,79406	-4,30608	-4,19098	-6,25984	-6,40873	-7,36602	-7,08979	-1,80476	-0,54661	-0,61031	-0,55835	0,156899	-10,324	-6,63816	-4,44478	0,247879	0,429685
cAMP_Pathway_Glycogen_Synthesis	-0,09338	-0,12964	0,043081	-0,57779	0,864747	0,706154	-0,94113	-0,80564	-0,93079	-2,10446	-0,32307	-0,08131	-1,03891	-0,72256	-0,22025	-1,34594	-1,14629	-0,66601	0,250176	0,432716
NCI_Arf6_trafficking_events_Pathway_(clathrin_c	-0,06664	-0,1236	-0,08553	0,065506	-0,46516	-0,86154	-0,87388	-0,98827	-1,44095	1,04953	0,291285	0,656274	0,401076	0,546595	0,419406	-3,14169	-2,33604	-2,00636	0,249912	0,432716
NCI_Caspase_Cascade_in_Apoptosis_Pathway_(a	-0,01808	0,038984	0,053496	-0,40625	-0,11887	0,053029	-0,56007	-0,45785	-0,62624	-0,15207	0,226533	-0,13169	0,11223	-0,00933	-0,15699	-0,92553	-0,99633	-0,77771	0,250184	0,432716
stearate_biosynthesis	0,01959	-0,04504	-0,02375	-0,94153	-0,96256	-0,67717	-0,26213	-0,37124	-0,492	1,534369	0,46409	0,775629	0,504969	0,382889	0,024985	-0,63436	-0,36444	-0,37317	0,249775	0,432716
folate_transformations	0,230143	0,095438	-0,18256	-0,71097	-0,63277	-0,824	-0,55234	-0,22976	-0,71228	0,245946	0,268597	-0,54511	0,190664	0,343752	0,362184	-3,09108	-2,44137	-1,84089	0,250335	0,432737
NCI_PLK3_signaling_events_Main_Pathway	-0,00491	0,000871	-0,11216	0,566773	0,387691	0,045867	-0,18524	0,125968	-0,31539	0,890076	-0,44834	-0,08565	-0,11428	0,0295	0,217202	-0,20651	0,272878	-0,45964	0,250593	0,432944
reactome_Attenuation_phase_Main_Pathway	-0,1609	-0,35752	-0,1688	-0,40036	-0,11309	0,439651	-0,83558	-0,59783	-1,29032	0,404758	0,705496	0,134366	0,082302	-0,31412	-0,34399	-2,34881	-2,30642	-1,22484	0,25095	0,43332
aspirin-triggered_lipoxin_biosynthesis	-0,10575	0,100289	0,117501	-0,25555	-0,78326	-0,67069	0,002974	-0,04074	-0,07354	0,483459	-0,06243	-0,15748	0,097187	0,194707	0,360054	0,174474	0,289371	-0,09319	0,251855	0,434161
aspirin_triggered_resolvin_D_biosynthesis	-0,10575	0,100289	0,117501	-0,25555	-0,78326	-0,67069	0,002974	-0,04074	-0,07354	0,483459	-0,06243	-0,15748	0,097187	0,194707	0,360054	0,174474	0,289371	-0,09319	0,251855	0,434161
aspirin_triggered_resolvin_E_biosynthesis	-0,10575	0,100289	0,117501	-0,25555	-0,78326	-0,67069	0,002974	-0,04074	-0,07354	0,483459	-0,06243	-0,15748	0,097187	0,194707	0,360054	0,174474	0,289371	-0,09319	0,251855	0,434161
mTOR_Pathway_Mitochondria_Proliferation_and	0,010669	0,002331	-0,06066	-0,86649	-0,60097	-1,09477	-0,14038	-0,02619	-0,2692	0,332188	0,231152	0,115935	0,038339	-0,0125	0,079792	-0,9322	-0,43214	-0,24617	0,252845	0,435626
KEGG_D_Arginine_and_D_ornithine_metabolism	0,00197	0,146176	0,132876	0,214507	0,109759	0,208573	0,091478	0,049983	0,149941	0,360213	-0,10989	0,218568	-0,05179	0,006726	0,159	0,775789	0,086964	0,426597	0,253159	0,435925
HIF1Alpha_Pathway_p53_Hypoxia_Pathway	-0,2742	-0,28597	-0,14821	-0,24176	0,981634	-0,64112	-0,24058	-0,22167	0,116134	0,225152	0,364121	2,487286	1,01142	0,602866	0,031876	2,190611	1,082918	1,969616	0,253354	0,436021
biocarta_activation_of_pkc_through_g_protein_c	-0,23364	-0,13286	-0,1107	-0,00188	1,417992	-0,31378	-0,88689	-0,92892	-0,69802	1,091475	0,78176	-0,49293	0,034894	-0,08033	0,464442	2,192456	1,540545	2,471669	0,253497	0,436064
reactome_p130Cas_linkage_to_MAPK_signaling	-0,31412	-0,19808	-0,12633	-1,11866	0,083699	-0,72507	-0,75858	-0,84818	-0,67759	-0,16315	0,148563	-0,9232	-0,09131	-0,3025	-0,80975	0,182295	-0,37685	-0,26739	0,253799	0,436064
reactome_Regulation_of_the_Fanconi_anemia_N	0,176996	-0,04293	0,12229	0,033734	0,2466	-0,01342	1,124906	1,280768	0,996189	-0,59675	1,018968	0,406372	-0,21059	-0,08957	0,011624	-2,54542	-1,63452	-1,10578	0,253753	0,436064
KEGG_Selenocompound_metabolism_Main_Path	0,244478	0,149659	0,164052	0,164683	0,225993	-0,21359	0,520845	0,608221	0,065139	-0,5315	0,035046	0,363087	-0,16951	-0,76562	-0,39927	-3,05544	-3,20116	-2,35298	0,254345	0,436762
FLT3_Signaling_Pathway_Transcription_via_ELK3	0,407223	-0,52753	-0,38108	-1,1782	-1,15707	-2,60709	-1,73618	-0,8483	-2,98277	-3,14103	2,556226	-0,27617	0,373827	0,014723	1,324594	-6,01895	-4,56912	-4,91294	0,254881	0,437441
glycine_biosynthesisdegradation	-0,01949	0,056156	0,035623	-0,17156	-0,00316	-0,1628	0,045956	-0,0129	0,344009	0,598285	-0,41244	0,526758	0,118505	0,257139	0,279557	0,67183	0,273647	0,253436	0,257373	0,441474
progesterone_biosynthesis	-0,195	0,012547	0,167193	-0,01482	-0,13369	-0,08175	0,010772	0,094304	0,28697	0,468305	-0,08142	-0,17765	-0,26497	0,124313	0,218224	0,600147	0,057621	0,154816	0,25772	0,441583
superoxide_radicals_degradation	-0,02721	-0,02613	-0,10374	-0,38469	-0,28937	0,019858	-0,08777	0,161615	-0,16519	-0,36573	0,512381	0,176245	0,023199	-0,30658	-0,09281	-1,65591	-1,86565	-1,50547	0,25758	0,441583
NCI_EPO_signaling_Pathway_(beta_integrin_med	-0,17413	-0,13983	-0,03474	0,063546	0,459134	0,484682	-0,41852	-0,50818	-0,45519	-0,7109	-0,09987	0,080072	-0,4765	-0,051	-0,29321	-0,58611	-0,28385	0,194988	0,258303	0,442339
PTEN_Pathway_Genomic_Stability	-0,91031	-0,3493	-0,41926	-0,0441	-0,39914	0,753124	7,139926	6,865355	7,394548	-2,12363	-4,23786	-2,98325	0,167101	-0,50532	-0,45735	12,57745	12,54918	10,99454	0,258692	0,442761

KEGG_Ribosome_Main_Pathway	4,50151	1,193301	0,633905	-9,89544	-10,2319	-13,3445	10,17568	12,28188	5,011719	-7,66409	6,610717	2,75473	-1,55269	-0,97057	1,689133	-64,7002	-54,6785	-49,4675	0,259366	0,443249
NCI_Caspase_Cascade_in_Apoptosis_Pathway_(D	0,070926	0,014995	0,065494	-0,44917	-0,10656	-0,25931	-0,39321	-0,20338	-0,42456	-0,05807	-0,18342	0,013025	0,203112	0,035525	-0,26979	-1,71821	-1,10773	-0,93217	0,259236	0,443249
NCI_IL3_mediated_signaling_events_Main_Pathw	-0,1005	-0,38032	-0,17683	-0,53428	-0,34981	-0,2471	-0,22467	-0,40349	-0,34482	0,710941	0,061041	-0,4403	-0,02435	-0,67238	-0,21866	-0,62126	-0,2455	-0,19393	0,259404	0,443249
VEGF_Pathway	-0,84614	-1,18571	-1,21659	-3,90379	-2,77191	-3,31776	-3,1438	-2,31047	-4,00163	-2,03018	0,641044	0,313444	1,802602	1,028757	2,485111	-9,45714	-5,96716	-4,36149	0,259715	0,443537
UMP_biosynthesis	0,068412	0,024059	0,082827	0,182203	0,504107	-0,23377	0,038292	0,095532	-0,0042	-0,26912	0,319894	0,413193	0,087536	-0,07942	0,055122	-0,65429	-0,5405	-0,38488	0,26004	0,44385
reactome_Free_fatty_acid_receptors_Main_Path	-0,27103	-0,06595	-0,08483	-0,08117	0,238492	0,449582	0,19225	0,029888	0,700235	0,347125	-0,02477	-0,35572	-0,02604	0,009497	0,519629	2,085937	2,000253	2,106979	0,260264	0,443989
Regulation_of_Cell_Migration_and_Proliferation_	-0,49694	-0,61821	-0,75997	2,889869	2,258742	-0,65894	-1,67797	-1,76141	-1,53406	-2,49194	1,377521	2,282648	-0,80581	-0,91605	-0,23722	-1,97361	-2,27408	-0,60698	0,260604	0,444326
KEGG_Chagas_disease_American_trypanosomiasi	0,66375	0,034648	0,030431	-0,57282	0,274616	-0,17383	-4,45579	-4,21714	-4,65089	0,418227	1,43221	0,922305	1,088624	-0,62771	1,231162	-4,0695	-3,6468	-1,98922	0,260874	0,444542
KEGG_Fructose_and_mannose_metabolism_Mair	-0,03016	0,268403	0,432509	-0,59314	-0,91421	-0,17575	-2,30332	-2,08679	-2,04326	2,880389	1,098578	2,35258	0,785321	0,157118	0,200154	-0,14792	-1,71664	-0,31776	0,2614	0,444784
reactome_Glycogen_breakdown_glycogenolysis_	0,08123	-0,17062	0,335176	-0,95654	0,223531	-0,99777	-0,9046	-0,5318	-1,17516	-0,92151	1,286082	0,471426	1,070465	0,136699	0,957477	-2,52399	-1,49582	-2,53661	0,261586	0,444784
reactome_TGFBR1_KD_Mutants_in_Cancer_Mair	-0,08192	-0,07527	-0,11554	-0,77384	-0,81586	-0,44127	-0,70143	-0,7865	-1,00396	0,111314	0,043771	0,458287	-0,13364	0,009317	-0,08925	-0,62006	-0,84211	-0,17296	0,261587	0,444784
reactome_TGFBR2_Kinase_Domain_Mutants_in_	-0,08192	-0,07527	-0,11554	-0,77384	-0,81586	-0,44127	-0,70143	-0,7865	-1,00396	0,111314	0,043771	0,458287	-0,13364	0,009317	-0,08925	-0,62006	-0,84211	-0,17296	0,261587	0,444784
lipoxin_biosynthesis	0,022321	-0,06119	0,132489	-0,04511	-0,40823	-0,24859	0,080149	-0,25473	0,150475	0,159285	0,569676	0,736099	0,359349	0,437335	0,405319	0,394652	0,718314	0,027762	0,263425	0,447664
IGF1R_Signaling_Pathway_Protein_Synthesis	0,348816	0,249353	0,104749	0,357153	-0,03946	-0,70241	-0,04725	0,283975	-0,70528	0,488565	0,334957	-0,46517	-0,64609	-0,25543	0,009387	-3,61525	-3,00613	-2,5248	0,264061	0,4485
HGF_Pathway_Regulation_of_Cytoskeleton_Cell_	0,00804	-0,33184	-0,44809	-1,2298	-0,65217	-0,66738	0,213058	0,387717	-0,32128	-1,24252	0,110048	-0,08781	-0,64761	0,068281	-0,05911	-3,75914	-2,20045	-1,87215	0,264469	0,448949
NCI_ErbB2_ErbB3_signaling_events_Pathway_(ce	0,216589	0,041812	-0,04576	-0,15111	-0,78237	-0,01669	0,422326	0,692346	-0,09048	-0,2561	0,683616	0,338499	-0,04525	0,089746	0,107949	-2,6562	-2,26667	-2,00571	0,264666	0,449039
NCI_Trk_receptor_signaling_mediated_by_the_M	-0,0242	-0,17138	-0,12835	-0,01661	-0,08668	-0,38207	-1,10611	-0,9635	-1,26057	0,325741	1,066996	0,445829	-0,27499	0,060293	-0,10319	-0,65921	-0,21139	0,021842	0,265403	0,449955
PTEN_Pathway_Cell_Cycle	-1,1748	-0,47863	-0,49303	-0,94338	-1,19215	-0,12322	7,010394	6,9548	7,59945	-1,93451	-4,57506	-5,23907	0,257127	-0,66036	-0,32482	11,82021	12,54643	11,36081	0,26564	0,449955
PTEN_Pathway_Growth	-1,1748	-0,47863	-0,49303	-0,94338	-1,19215	-0,12322	7,010394	6,9548	7,59945	-1,93451	-4,57506	-5,23907	0,257127	-0,66036	-0,32482	11,82021	12,54643	11,36081	0,26564	0,449955
reactome_Dimerization_of_procaspase_8_Main_	0,038329	-0,03007	-0,0645	-0,58207	-0,62893	-2,18319	-1,27419	-0,93699	-1,12342	0,126763	-0,72916	1,01275	-0,135	0,255632	-0,24505	-0,61013	-0,55182	-0,28484	0,266015	0,450101
reactome_Regulation_by_c_FLIP_Main_Pathway	0,038329	-0,03007	-0,0645	-0,58207	-0,62893	-2,18319	-1,27419	-0,93699	-1,12342	0,126763	-0,72916	1,01275	-0,135	0,255632	-0,24505	-0,61013	-0,55182	-0,28484	0,266015	0,450101
MAPK_Family_Pathway_Translation	-0,09729	-0,04865	-0,21495	0,107693	0,082472	-0,81166	-0,58013	-0,60637	-0,56918	-0,74258	-0,29709	-1,35487	0,666514	0,211574	0,521225	-0,03124	0,168819	0,760814	0,266193	0,450157
KEGG_Renal_cell_carcinoma_Main_Pathway	-0,29852	-0,41565	-0,4879	-1,81676	-1,26611	-0,81014	-1,87738	-1,65336	-2,1079	0,13984	-0,4194	2,462778	0,388133	0,839383	1,023469	-6,23976	-4,30825	-3,55391	0,267737	0,451589
MAPK_Family_Pathway_Cytoskeleton	0,062271	-0,2609	-0,18498	0,298147	-0,19807	0,088757	-0,41001	-0,25745	-0,28798	0,661027	0,3705	0,096684	0,506771	-0,15518	0,307546	-0,65701	-0,35363	-0,30296	0,267361	0,451589
reactome_AKT_mediated_inactivation_of_FOXO1	-0,03539	-0,117	-0,04903	0,150988	0,527747	0,313231	0,173285	0,219361	0,022247	-0,20746	0,263043	-0,04772	-0,01541	-0,14394	-0,13319	-0,15806	0,035911	0,077179	0,267764	0,451589
reactome_Signal_transduction_by_L1_Main_Path	-0,0563	-0,15635	-0,27161	-1,08569	-0,71932	-1,37397	-1,25397	-0,89375	-1,26298	-0,07799	-0,03874	-0,16856	-0,00231	-0,33872	-0,19477	-1,10779	-0,79226	-0,2084	0,267629	0,451589
the_visual_cycle_I_vertebrates	0,05006	-0,24933	0,04473	-1,11369	-0,22256	-1,03817	0,497604	0,858127	0,601628	-0,58552	-0,21365	-1,03148	0,599389	0,236248	-0,19269	1,087425	1,382245	0,992169	0,267417	0,451589
biocarta_attenuation_of_gpcr_signaling_Main_Pa	-0,03599	-0,27141	-0,20115	-0,71147	-0,22102	-0,10685	-1,93863	-1,94421	-1,8712	-0,42093	-0,14192	-0,05486	-0,81689	-0,6553	0,019339	-2,45382	-2,28925	-1,70764	0,268772	0,453043
biocarta_activation_of_pkc_through_g_protein_c	0,032264	-0,07643	-0,02636	-0,12511	0,502642	-0,56262	-0,38247	-0,33452	-0,33803	0,374931	0,104056	0,047206	0,297389	-0,17204	0,32852	0,801719	0,547837	0,679959	0,269391	0,453841
biocarta_role_of_egf_receptor_transactivation_b	-0,09285	-0,62188	-0,48383	-0,30407	-0,92542	-1,14177	-1,0171	-0,65748	-1,47866	-0,94907	0,018818	-0,31884	-0,15198	-0,79132	0,126315	-2,60792	-1,80112	-1,66047	0,2697	0,454117
biocarta_keratinocyte_differentiation_Main_Path	0,280019	-0,2877	-0,08596	-2,93196	-2,11919	-1,6492	-2,88663	-2,16609	-3,01815	-0,99587	-3,25848	-4,15562	0,609047	0,69596	0,920152	-4,08818	-2,00135	-1,43759	0,270407	0,455061
NCI_Signaling_events_mediated_by_Stem_cell_fa	0,019619	-0,45915	-0,54107	-1,60963	-0,21457	-0,78939	-4,76409	-4,54651	-5,70125	1,406415	1,636691	0,965957	-0,35331	-0,61204	0,571802	-7,57375	-7,69266	-6,63306	0,271735	0,457049
Role_of_KITLG_in_Haematopoiesis	-0,75657	-0,83978	-0,86653	-0,03852	-1,71936	-3,12732	-6,20592	-6,42349	-7,60077	2,767441	1,805639	2,837492	-0,20061	0,866881	2,310548	-11,6899	-9,71876	-7,15143	0,272048	0,457328
reactome_FCGR_activation_Main_Pathway	-0,13018	0,028722	0,07062	-0,02169	-0,23725	0,908557	0,220735	-0,17887	0,533738	0,996948	-0,15201	1,518398	0,234978	0,595671	0,277522	1,531756	0,766857	0,921867	0,272419	0,457458
tryptophan_degradation_to_2-amino-3-carboxym	-0,01494	0,165722	0,132092	-0,47342	-0,68748	-0,19713	-1,80978	-2,30991	-1,67795	-1,17441	-0,29509	-0,7546	0,175051	-0,49401	-0,36524	0,646581	0,801345	-0,07453	0,272344	0,457458
phenylalanine_degradationtyrosine_biosynthesis	0,005943	0,051541	0,037358	0,16828	0,027438	-0,12514	0,413223	0,448853	0,451467	0,153658	-0,22044	-0,17501	-0,15134	-0,16605	-0,11388	0,1427	0,192918	0,011622	0,272624	0,457556
BRCA1_Pathway_Nucleotide_Excission_Repair	0,083864	-0,07145	-0,03963	0,230483	-0,1013	0,054805	0,098676	0,31394	-0,10788	0,64846	-0,01364	0,324457	0,35329	0,281775	0,226668	-0,06684	-0,37159	-0,15806	0,272978	0,457903
adenosine_deoxyribonucleotides_ide_novoi_bios	0,079183	0,084535	-0,04546	-0,31565	-0,31698	0,099225	-0,25013	-0,21245	-0,70545	1,171059	1,781098	0,896813	0,599303	0,496131	0,75954	-3,47286	-2,98527	-2,59605	0,273661	0,458555
CMP_phosphorylation	0,070765	0,085571	-0,03586	-0,3775	-0,21078	0,139136	0,128311	0,19028	-0,42362	1,238293	2,206448	0,876395	0,654973	0,62128	0,683266	-3,69252	-3,42024	-2,88603	0,273604	0,458555
NCI_Regulation_of_RAC1_activity_Main_Pathway	0,347016	-0,00842	-0,01155	-3,06076	-1,05447	-1,73471	-2,3624	-2,27975	-2,74349	-2,52528	0,435881	-1,13556	-0,07695	1,343621	1,130044	-4,35908	-3,90266	-3,98373	0,275407	0,460985
reactome_Reduction_of_cytosolic_Ca_levels_Mai	-0,13868	-0,10845	0,029907	0,229783	1,482562	0,176	-0,93503	-1,27501	-0,87724	-2,42149	-1,1714	-1,30458	-0,19426	0,252155	0,058626	1,059191	0,894762	1,281678	0,275359	0,460985
KEGG_Steroid_biosynthesis_Main_Pathway	0,349984	0,247456	-0,04269	1,037641	1,216786	0,93408	1,514101	1,849859	1,243155	1,161631	1,63432	1,647518	-0,47292	-0,43036	-0,62306	-4,94309	-3,60303	-3,33955	0,275878	0,461526
biocarta_t_cell_receptor_signaling_Pathway_(Pat	-0,02838	-0,15546	-0,0206	-0,0274	-0,20171	-0,90681	-0,30932	-0,36239	-0,21409	-0,25121	0,010579	-0,66665	0,19047	-0,12793	0,112218	0,464492	0,707935	0,821355	0,276333	0,46179
tRNA_splicing	-0,07017	-0,03429	-0,13757	-0,45659	0,589028	0,416858	0,268846	0,322397	0,155635	0,207567	0,342901	-0,34312	-0,31763	-0,30786	-0,3113	-1,24072	-0,89193	-1,14599	0,276267	0,46179
reactome_The_activation_of_arylsulfatases_Main	-0,26017	-0,21807	0,107279	0,528092	0,056023	0,049954	-2,06819	-1,68525	-1,8177	1,694214	0,003777	0,059424	0,072332	0,133274	0,463436	-0,20457	-1,26416	-0,96568	0,276691	0,462141
biocarta_wnt_lrp6_signalling_Main_Pathway	-0,11991	-0,00469	0,083314	-0,45839	0,443913	-0,13344	-0,53742	-0,4998	-0,2087	-0,97784	-0,31225	-0,14312	0,357644	0,048551	0,018495	-0,1009	0,221874	-0,04325	0,277838	0,463093
NCI_Signaling_events_mediated_by_Hepatocyte_	-0,32649	-1,11603	-1,55551	-3,71466	-2,27596	-3,22593	-7,59551	-6,46025	-9,0205	-2,87635	1,271461	-0,66959	2,102356	1,219744	2,234649	-8,79526	-4,3388	-2,34337	0,277868	0,463093
palmitate_biosynthesis	-0,11159	-0,04991	-0,25495	-0,22043	-0,55716	0,385429	-0,97568	-1,06533	-1,33639	0,329919	0,515819	-0,40109	0,033062	0,176707	0,088005	-0,64243	-0,93986	-0,72686	0,278004	0,463093
reactome_Nonhomologous_End_joining_NHEJ__I	0,172332	0,06027	0,032887	0,288563	-0,27088	-0,14036	-0,60678	-0,43581	-0,90309	-0,46485	0,273751	0,030974	-0,44184	-0,23356	-0,24833	-2,3445	-1,45594	-1,20844	0,277644	0,463093
reactome_Norepinephrine_Neurotransmitter_Re	-0,1807	-0,53102	-0,0974	-0,61085	-0,63455	-1,39596	-2,86081	-2,86733	-2,60946	-2,48399	-2,28594	-1,89796	0,324516	-0,0347	0,411283	6,309236	5,868177	5,484202	0,277517	0,463093
KEGG_Peroxisome_Main_Pathway	0,777599	-0,46819	-0,41942	-1,83012	-0,48043	-1,95267	-1,46617	-0,2468	-3,13831	-3,16692	1,921183	4,839928	-0,33387	-1,87058	0,440411	-15,2273	-11,9919	-9,7458	0,278704	0,46401
reactome_Carnitine_synthesis_Main_Pathway	0,128953	-0,10421	-0,16828	-0,11639	-0,60407	-0,40218	1,581486	1,80648	1,247827	0,068839	0,184661	-0,16689	0,382636	0,176512	0,304095	-1,56927	-0,58769	-0,68605	0,278905	0,464096
Ephrin-mediated_Signaling_Events_During_Cell_A	0,419455	-0,01258	0,147419	-0,83841	-2,4149	-0,83313	-3,76954	-3,82465	-3,99453	-0,42876	0,574808	-1,2747	0,027359	0,150774	0,522672	-1,07953	-0,14794	2,548749	0,280115	0,464888
mucin_core_1_and_core_2_iOi-glycosylation	-0,39728	-0,56024	-0,35323	-0,8102	0,931757	0,027726	1,584882	0,877878	1,424262	-0,16383	-1,41709	-0,46656	-0,1043	1,58282	1,163892	0,994718	0,920946	0,894908	0,279795	0,464888
NCI_S1P2_Pathway_(regulation_of_vascular_perr	-0,08538	-0,14777	-0,1423	0,110397	-0,15544	-0,55387	-0,73794	-0,67814	-0,49969	-0,62705	0,301177	0,113212	0,0605	-0,23692	0,10338	-0,64518	-0,55976	-0,99439	0,27984	0,464888
PTEN_Pathway_Cell_Survival	0,048561	0,002127	0,135256	-0,31177	-0,8707	0,16864	0,568913	0,629485	1,132855	0,167491	-1,29856	-1,234	0,032368	0,080578	-0,12015	2,498448	1,417829	1,218811	0,280575	0,464888
reactome_Dopamine_Neurotransmitter_Release_	-0,41261	-0,78673	-0,46757	-0,04881	-0,37749	-0,67987	-3,15232	-3,12109	-2,79848	-2,70866	-2,06673	-1,3275	-0,10735	-0,51643	0,181166	6,477042	6,010625	6,489581	0,280538	0,464888
reactome_Glycogen_synthesis_Main_Pathway	0,361563	0,088268	0,315987	-0,31112	-0,36728	-2,59505	-0,56143	-0,2251	-0,97384	-0,09168	0,279864	0,783218	0,395129	0,600916	0,481473	-5,77425	-4,45997	-4,39767	0,280147	0,464888
reactome_Serotonin_and_melatonin_biosynthesi	0,000649	-0,01788	0,065379	0,197153	0,015428	0,085016	-0,10055	-0,23734	0,25546	-0,78062	-0,49938	-0,06163	0,160749	-0,28429	-0,0665	1,697807	1,41008	1,748913	0,279889	0,464888
reactome_Serotonin_Neurotransmitter_Release_	-0,41261	-0,78673	-0,46757	-0,04881	-0,37749	-0,67987	-3,15232	-3,12109	-2,79848	-2,70866	-2,06673	-1,3275	-0,10735	-0,51643	0,181166	6,477042	6,010625	6,489581	0,280538	0,464888
HMGB1_signaling_through_RAGE	-0,16714	-0,65094	-0,38726	-2,52603	0,196273	-0,64065	-3,62586	-3,10881	-4,41997	-1,74578	1,057729	0,377536	1,445687	0,41989	1,70801	-2,82707	-1,84266	-0,62778	0,281042	0,465065
IL-2_Pathway_Gene_Expression_via_FYN_LYN_RF	0,135209	0,419375	0,274091	-0,49453	0,678686	-0,64605	-0,1607	-0,58786	-0,60047	0,723771	1,570661	1,4268	0,281578	-0,06204	0,159252	-0,45676	-0,38852	-0,51256	0,281279	0,465065
reactome_Synthesis_of_PIPs_at_the_Golgi_meml	-0,21173	-0,06177	-0,25483	-0,23385	-1,18229	-0,46763	-0,15568	0,292875	-0,6923	-1,84279	-1,09093	-0,65118	-1,27701	-0,97915	-0,53795	-3,03132	-1,57201	-2,19886	0,280881	0,465065
TRAF_Pathway_Direct_Antimicrobial_Response_a	-0,15982	0,142709	-0,00725	-0,52223	-1,23458	0,074156	0,253187	0,17279	0,162715	0,121758	-0,71155	-0,35121	0,262979	0,682513	0,501078	-0,95359	-1,01972	-0,88141	0,281269	0,465065
reactome_Acetylcholine_Neurotransmitter_Relea	-0,26921	-0,47001	-0,32752	-0,08121	-0,82432	-1,71586	-2,56986	-2,53742	-2,44995	-2,54628	-1,9534	-2,02357	0,352367	-0,0632	0,19959	6,064433	5,456639	5,599845	0,281491	0,46517
BRCA1_Pathway_G1_S_arrest	0,134437	0,02553	-0,03551	0,239466	0,617266	-0,29119	-0,15043	0,111182	-0,51563	0,582869	0,298096	0,317843	0,003729	0,1215	-0,03219	-1,16034	-0,62109	-0,97235	0,282678	0,466884
reactome_Pre_NOTCH_Processing_in_Golgi_Mair	-0,23236	-0,22956	-0,08776	0,412447	1,347662	1,129078	0,285667	0,378728	0,477069	0,641132	-0,44118	-0,75253	0,390565	-0,22131	0,00992	1,319431	-0,24119	1,10953	0,283606	0,467958
reactome_Regulation_of_actin_dynamics_for_ph	-0,23996	-0,81392	-0,73549	-1,91658	-2,29013	-3,18376	-2,57315	-2,30117	-3,12195	0,830862	-1,34302	2,154883	1,257272	1,434178	1,854898	-9,27231	-6,80087	-3,47471	0,283629	0,467958
biocarta_phospholipids_as_signalling_intermedia	0,007474	-0,12786	-0,1183	-0,0285	-0,3642	-0,75626	-0,71273	-0,55043	-0,87588	-0,30069	0,479541	0,633608	-0,13288	-0,28829	-0,06691	-0,5494	-0,55955	-0,16066	0,284908	0,468919
biocarta_phospholipids_as_signalling_intermedia	0,007474	-0,12786	-0,1183	-0,0285	-0,3642	-0,75626	-0,71273	-0,55043	-0,87588	-0,30069	0,479541	0,633608	-0,13288	-0,28829	-0,06691	-0,5494	-0,55955	-0,16066	0,284908	0,468919
NCI_Endothelins_Pathway_(regulation_of_vascula	0,007474	-0,12786	-0,1183	-0,0285	-0,3642	-0,75626	-0,71273	-0,55043	-0,87588	-0,30069	0,479541	0,633608	-0,13288	-0,28829	-0,06691	-0,5494	-0,55955	-0,16066	0,284908	0,468919
reactome_Activation_of_C3_and_C5_Main_Pathv	0,083176	0,115839	0,080377	0,286374	-0,06349	0,501196	0,132126	0,268502	0,245969	-0,13629	-0,94878	-0,00074	-0,34674	-0,39922	-0,40575	-0,71157	-0,91612	-0,38417	0,284964	0,468919
reactome_Sema4D_induced_cell_migration_and_	-0,19092	-0,33638	-0,55112	-1,13004	-0,6847	-0,79869	-1,40818	-1,18305	-1,77864	-0,7756	-0,50359	-0,81917	0,218713	0,379284	-0,15796	-1,31404	-1,12219	-0,49216	0,284689	0,468919
reactome_Xenobiotics_Main_Pathway	-0,23311	0,089887	-0,2034	2,196565	1,371452	0,352524	-0,48673	-1,15443	0,814815	1,650942	2,363944	3,332677	0,256839	0,870624	0,536482	4,080054	2,207422	1,099272	0,286476	0,471158
biocarta_regulation_of_spermatogenesis_by_crer	-0,02777	0,149752	0,095427	-0,57153	-0,83628	-0,67885	0,162244	-0,05429	0,063615	0,92708	0,02729	-0,40757	0,249704	0,164607	0,525649	0,028921	0,193189	-0,01302	0,286925	0,471399
PTEN_Pathway_Differentiation	-1,156	-0,4243	-0,45137	-1,03249	-1,06306	0,065697	7,161949	7,068319	7,791915	-1,75448	-4,76164	-5,57122	0,372096	-0,52302	-0,28179	11,8021	12,59512	11,25553	0,286855	0,471399
NCI_Caspase_Cascade_in_Apoptosis_Pathway_(p	0,139088	0,008821	-0,09293	-0,02395	0,027895	-0,31447	-0,58577	-0,49379	-0,77997	0,129112	0,544645	0,413886	0,010566	-0,08799	-0,08503	-2,25188	-1,82039	-1,36167	0,28895	0,474475
reactome_EPH_ephrin_mediated_repulsion_of_c	-0,40204	-0,41789	-0,59306	0,056506	-1,12147	-2,15742	-4,59594	-4,92682	-5,44433	0,730889	3,871373	2,952077	0,62747	1,279691	1,676488	-0,76916	1,788951	1,566502	0,289524	0,475167
reactome_Glutathione_synthesis_and_recycling_	0,007447	-0,01355	-0,0649	-0,30781	-0,11847	0,051238	-0,25352	-0,14291	-0,45469	0,135483	0,606114	0,609441	0,405282	0,573662	0,29806	-1,1246	-0,49601	-0,61493	0,289853	0,475456
NCI_E_cadherin_signaling_in_the_nascent_adher	0,100422	-0,21079	0,092962	-1,94696	0,716387	-2,20127	-0,98096	-0,70545	-1,42687	-0,51549	0,202941	1,33816	-0,63803	0,19125	-0,064	-8,83174	-5,95068	-4,98941	0,290121	0,475645
reactome_Early_Phase_of_HIV_Life_Cycle_Main_	0,041973	0,069889	0,055435	-0,2653	-0,13534	-0,27591	0,707349	0,772752	0,673576	-0,64805	0,474291	0,808483	-0,1178	-0,15361	0,034379	-0,3161	-0,10182	-0,24004	0,290405	0,475861
reactome_RIG_I_MDA5_mediated_induction_of_	0,199698	0,097613	0,083919	0,466055	-0,22934	0,019606	0,087974	0,337145	0,046744	0,76025	0,248148	0,231088	-0,19371	0,26422	0,363016	-1,63242	-0,84597	-0,8954	0,291071	0,476701
biocarta_mcalpain_and_friends_in_cell_motility_	0,003573	-0,35548	-0,40051	-1,41565	-2,90665	-3,01844	-0,19011	0,162281	-0,55667	-0,78019	0,60176	-1,35707	0,635745	-0,23566	-0,11295	-0,88367	-1,00062	0,459895	0,292333	0,47834
KEGG_Chronic_myeloid_leukemia_Main_Pathway	-0,49959	-0,80518	-1,10227	-2,44301	-0,56219	-1,13517	-4,88094	-4,14094	-6,14252	-1,00057	0,236764	2,242505	0,540783	-0,10264	1,06213	-6,81175	-3,97764	-2,18297	0,292405	0,47834
KEGG_Systemic_lupus_erythematosus_Main_Patl	0,102335	0,15673	0,072799	0,151532	-0,16308	-0,03352	-0,90672	-0,85406	-0,403	0,191443	-1,16504	-0,33154	0,222316	0,386274	-0,56907	1,913177	1,188176	1,237152	0,292532	0,47834
biocarta_west_nile_virus_Main_Pathway	0,226082	0,136343	0,070985	0,031133	-0,3558	-0,01962	-0,31506	-0,06103	-0,64596	0,934215	-0,29664	-0,48084	-0,03042	-0,25335	-0,41096	-2,46986	-1,88815	-1,72894	0,29305	0,478434
biocarta_west_nile_virus_Pathway_(apoptosis)	0,226082	0,136343	0,070985	0,031133	-0,3558	-0,01962	-0,31506	-0,06103	-0,64596	0,934215	-0,29664	-0,48084	-0,03042	-0,25335	-0,41096	-2,46986	-1,88815	-1,72894	0,29305	0,478434
NCI_FGF_signaling_Main_Pathway	0,037413	0,342523	0,37531	0,532451	-0,00455	1,05817	0,724841	0,51864	0,958111	0,974599	0,496494	-0,43745	0,26105	0,261206	0,012317	1,943916	1,438369	0,883592	0,292945	0,478434
biocarta_caspase_cascade_in_apoptosis_Pathway	-0,30269	0,20348	0,032075	0,025489	-0,45753	0,262375	0,924878	0,848578	1,203096	0,439995	0,465893	0,745349	0,169439	0,255522	0,432211	0,765664	0,37659	0,013787	0,293521	0,478952
reactome_Signaling_by_activated_point_mutants	-0,46232	-0,23342	-0,13583	-0,46435	-0,26639	-0,3357	-0,61554	-0,96482	-0,26193	1,020705	-0,35001	0,243025	0,073011	-0,17034	-0,27415	-0,05054	0,220295	-0,62357	0,293861	0,479255
NCI_Plexin_D1_Signaling_Main_Pathway	-0,14463	-0,26274	-0,40349	-0,22325	-1,51432	-0,65961	-2,59871	-2,36879	-3,13163	0,741138	0,492553	-0,55541	0,828481	1,252509	0,864491	-1,3355	-1,10847	-1,75324	0,29586	0,482263
ERK_Signaling_Pathway_Cell_Survival	0,165241	0,038883	-0,11778	-0,36205	-1,01993	-1,00381	-0,88472	-0,67307	-1,40156	-1,2767	0,427938	-0,65744	0,195535	0,154247	0,244553	-1,67989	-1,85151	-1,4385	0,296745	0,483453
KEGG_Inflammatory_mediator_regulation_of_TR	-0,57762	-1,22069	-0,56708	0,562797	1,140986	-0,14366	-0,5218	-0,41214	0,320443	2,203844	1,765332	0,261256	0,610572	0,960437	2,577796	4,831529	5,677502	6,520169	0,297214	0,483657
reactome_Downstream_signal_transduction_Mai	0,076702	-0,26197	-0,30633	-0,99606	0,690591	-1,3886	-0,84147	-0,52325	-0,91668	0,537759	0,181136	0,739236	0,189692	-0,32439	0,419349	-4,37422	-3,0608	-2,47657	0,297336	0,483657
reactome_LDL_endocytosis_Main_Pathway	0,061875	0,224368	0,333683	-0,29466	-0,12394	-0,28339	0,126712	-0,24262	0,325087	0,563047	0,059436	-0,23975	0,417936	0,533705	-0,15613	0,490655	0,279984	0,073421	0,297304	0,483657
4-hydroxybenzoate_biosynthesis	-0,03148	0,024319	0,006746	0,162311	-0,1479	0,138878	-0,00184	0,043466	0,230683	-0,1641	0,422144	0,383445	-0,0098	0,204529	-0,11389	0,446824	-0,0355	0,263747	0,297819	0,484182
KEGG_Regulation_of_actin_cytoskeleton_Main_P	0,860403	1,324296	0,781025	3,819722	-0,82	3,781448	4,807455	3,603306	5,025406	-7,2285	-3,48823	-7,74845	1,157171	-0,84331	-0,9794	4,705852	3,804575	-0,97416	0,29797	0,484182
NCI_FAS_CD95_signaling_Pathway_(release_of_c	-0,00961	0,022427	-0,07184	-0,0391	0,169882	0,44299	-0,53138	-0,42456	-0,66934	-0,34582	-0,0097	0,307692	-0,16611	-0,11657	-0,31019	-0,94925	-0,57915	-0,02875	0,298263	0,484405
biocarta_pten_dependent_cell_cycle_arrest_and_	0,042359	0,010872	0,000608	-0,65897	-0,30227	-0,63251	0,145503	0,007345	-0,02527	-0,37342	-0,7441	-0,32692	0,496357	0,238983	0,479772	-0,66569	-0,41569	-0,51792	0,298481	0,484507
pyridoxal_5-phosphate_salvage	-0,09119	-0,07215	-0,09926	-0,1776	0,141665	0,274622	0,059666	0,003538	-0,08118	0,562199	0,072658	0,908197	-0,0885	0,057769	0,033233	0,155572	0,017931	0,190686	0,298776	0,484732
CDC42_Pathway	-0,14062	-0,50963	-0,28551	-1,97085	-2,33872	-1,65545	-2,78177	-2,28683	-3,45539	-4,60232	0,210854	-0,04743	0,27149	0,430657	0,900114	-5,48601	-2,63933	-1,91493	0,299511	0,485673
reactome_Acyl_chain_remodelling_of_PC_Main_	-0,14416	0,032399	-0,19782	-0,26325	-0,85193	-1,14695	-1,38937	-1,25755	-0,84554	-0,58923	-1,19052	-0,61627	-0,13926	0,034696	0,002818	-1,78016	-0,74815	-1,00141	0,300677	0,487055
reactome_Asparagine_N_linked_glycosylation_M	0,253085	0,162865	0,137742	-0,12743	-0,50502	0,535549	0,322281	0,323237	0,234748	0,646324	0,534925	0,491241	-0,11144	-0,07449	-0,00397	-2,87058	-2,62549	-2,26813	0,300662	0,487055
biocarta_rho_cell_motility_signaling_Pathway_(a	-0,14797	-0,14661	-0,16798	-0,27352	-0,60676	-0,59642	-0,632	-0,77172	-0,68305	0,350143	0,023874	0,087026	0,046059	-0,0969	-0,16494	-0,52239	-0,35088	-0,29977	0,301014	0,487168
NCI_EPHA2_forward_signaling_Main_Pathway	-0,18447	-0,05132	-0,24029	-0,54286	0,437142	-0,84042	-1,2525	-1,23031	-1,54582	0,547959	0,769326	0,954232	0,519227	0,393842	0,152666	-2,53124	-1,88666	-1,97114	0,301442	0,487168
reactome_Beta_oxidation_of_palmitoyl_CoA_to_	0,073	0,014935	-0,02741	-0,42707	-0,25967	-0,44764	0,253905	0,316866	0,068965	-0,32143	-0,23858	0,35789	0,17422	-0,0144	0,075063	-1,4041	-1,05123	-0,97241	0,301137	0,487168
reactome_IRAK2_mediated_activation_of_TAK1_	0,169334	-0,02947	-0,02552	-0,93981	-1,24448	-1,21578	-0,04765	0,075573	-0,46701	-0,90259	0,098613	-0,2806	0,184096	0,301451	0,197663	-3,35641	-2,71169	-2,43654	0,301528	0,487168
reactome_IRAK2_mediated_activation_of_TAK1_	0,169334	-0,02947	-0,02552	-0,93981	-1,24448	-1,21578	-0,04765	0,075573	-0,46701	-0,90259	0,098613	-0,2806	0,184096	0,301451	0,197663	-3,35641	-2,71169	-2,43654	0,301528	0,487168
KEGG_Mucin_type_O_Glycan_biosynthesis_Main	-0,65941	-0,65482	-0,42406	-2,30844	-0,25697	-0,89631	0,930873	0,412586	0,614189	-0,6936	-1,64997	-2,37414	-0,91095	1,598752	1,115548	-1,35033	-0,83958	-0,19611	0,30249	0,488216
NCI_FAS_CD95_signaling_Pathway_(neuron_proj	0,082205	0,108153	0,007104	-0,41501	-0,0299	0,240969	-0,34701	-0,23621	-0,46175	-0,24056	-0,10327	-0,0471	-0,20628	-0,14166	-0,39693	-1,70677	-1,17345	-0,63804	0,302385	0,488216
KEGG_Butirosin_and_neomycin_biosynthesis_Ma	-0,02894	0,044679	0,069429	-1,23948	-0,52977	-0,00054	-0,37847	-0,71947	-0,35856	-0,46615	0,226302	0,586197	0,379324	0,111825	0,397658	0,335881	0,124118	0,593798	0,30378	0,489536
reactome_2_LTR_circle_formation_Main_Pathwa	-0,01426	-0,17469	-0,11069	-0,19273	-0,42249	-0,61002	-0,85329	-0,6354	-1,09291	-0,09874	0,428832	-0,21982	-0,39877	-0,28899	-0,1852	-2,84088	-2,20027	-1,82097	0,30377	0,489536
STAT3_Pathway_Anti-Apoptosis	-0,04247	-0,11515	-0,07179	0,099435	0,551333	-0,1149	-0,32823	-0,37378	-0,26767	0,58752	0,202328	0,147986	-0,05481	-0,00482	0,141539	-0,39562	-0,3268	-0,49494	0,303568	0,489536
NCI_CXCR4_mediated_signaling_events_Pathway	0,053194	-0,14815	-0,06275	-0,57005	-0,32087	-0,21555	-0,19888	-0,10573	-0,5005	-0,08693	-0,17174	-0,24723	0,14652	0,334429	0,207214	0,430214	0,556274	0,978916	0,30495	0,490147
NCI_Syndecan_1_mediated_signaling_events_Pat	-0,68002	-0,75047	-0,22677	-1,73949	-2,26175	-2,10081	-0,27736	-0,97508	0,93213	-2,12646	-1,45408	-0,21403	0,201483	0,440331	0,760274	2,941996	2,306135	0,822496	0,305103	0,490147
NCI_Syndecan_1_mediated_signaling_events_Pat	-0,68002	-0,75047	-0,22677	-1,73949	-2,26175	-2,10081	-0,27736	-0,97508	0,93213	-2,12646	-1,45408	-0,21403	0,201483	0,440331	0,760274	2,941996	2,306135	0,822496	0,305103	0,490147
NCI_Syndecan_1_mediated_signaling_events_Pat	-0,68002	-0,75047	-0,22677	-1,73949	-2,26175	-2,10081	-0,27736	-0,97508	0,93213	-2,12646	-1,45408	-0,21403	0,201483	0,440331	0,760274	2,941996	2,306135	0,822496	0,305103	0,490147
Nitric_Oxide_Pathway_in_Skeletal_Muscle	-0,02909	0,028891	-0,1872	-2,52931	-1,23311	-1,83566	-1,20618	-1,50767	-0,01152	-1,31191	-1,30663	-0,40437	1,417043	0,358673	0,870955	2,178168	1,866833	2,698667	0,304808	0,490147
p53_Signaling_Pathway_Inhibition_of_IGF1R_mT	0,0085	-0,02724	-0,02963	0,332899	-0,00847	0,104167	0,080666	0,07828	0,040808	0,308876	0,180739	0,071281	0,209676	0,107608	0,200128	0,179443	0,132705	-0,00295	0,304677	0,490147
reactome_GAB1_signalosome_Main_Pathway	-0,00672	-0,17069	-0,32267	-0,75634	-0,55527	-0,70064	-0,21691	-0,14787	-0,54045	-0,84198	0,448345	-0,12797	0,330833	0,310617	0,757844	-1,87688	-0,88639	-0,47302	0,305581	0,490663
reactome_Vitamin_B2_riboflavin_metabolism_M	-0,2811	-0,25977	-0,03964	-1,21944	-1,3915	-1,7332	-0,20637	-0,30859	-0,39507	0,29086	0,146642	1,307357	-0,07505	0,205105	0,152152	-0,88362	-1,25158	-0,88333	0,305942	0,490988
biocarta_role_of_pi3k_subunit_p85_in_regulatior	0,138639	-0,08251	0,046881	-0,29347	-0,21265	-0,07085	-0,02155	0,049204	-0,28645	-0,40051	-0,42557	0,10977	0,232888	0,471398	0,28351	1,128417	1,243252	1,487196	0,306187	0,491129
Interleukin-8-induced_Cell_Adhesion_and_Migrat	0,156198	-0,2635	-0,06861	1,310124	0,597945	-0,07508	-1,53503	-1,55517	-1,39757	0,36924	0,656848	-0,17245	0,309279	0,378924	0,395305	0,251907	0,612372	0,65955	0,306422	0,491254
KEGG_Glycosaminoglycan_biosynthesis_chondroi	0,01078	-0,30876	-0,27544	0,51472	-0,56434	-0,23421	-0,8018	-0,81856	-1,20504	-2,73511	-1,97927	0,072758	-0,18802	0,005052	0,552021	-0,32277	1,447443	-0,06456	0,307378	0,492533
reactome_Prolactin_receptor_signaling_Main_Pa	-0,03162	-0,02046	0,036751	-0,06669	-0,48669	-0,33247	0,353448	0,230324	0,289404	1,349352	-0,56188	0,056154	-0,33377	-0,37509	-0,09689	-0,78332	-0,47882	-0,03254	0,30778	0,492923
NCI_ALK1_signaling_events_Pathway_(endothelia	-0,09101	0,008694	-0,06847	-0,36684	-0,52506	-0,33987	-0,97165	-0,98944	-1,3085	-1,35696	-0,28406	-0,23551	0,03482	0,04279	0,158461	-1,07408	-0,53186	0,170062	0,307968	0,492971
reactome_Inositol_transporters_Main_Pathway	0,055352	0,162541	0,161342	0,85381	-0,24649	0,686433	0,12522	0,075403	0,630924	-1,30467	-0,98181	-1,44363	0,263588	-0,18548	-0,20064	0,900186	0,353102	0,554068	0,308412	0,493402
reactome_Synthesis_of_Dolichyl_phosphate_Mai	-0,11002	-0,16644	-0,16963	-0,08514	0,002279	-0,40959	0,181034	0,211631	0,029175	0,051105	-0,24132	-0,32411	-0,28063	-0,35621	-0,03293	0,234172	-0,13081	-0,00718	0,308554	0,493402
biocarta_cd40l_signaling_Main_Pathway	0,332514	0,204061	0,198488	-0,06262	-0,53206	0,205487	-0,07771	-0,11606	-0,05522	0,144772	-0,0871	-0,15708	0,30251	-0,06582	0,30285	-0,83515	-1,10692	-0,50241	0,309419	0,493517
biocarta_cd40l_signaling_Pathway_(Pathway_pro	0,332514	0,204061	0,198488	-0,06262	-0,53206	0,205487	-0,07771	-0,11606	-0,05522	0,144772	-0,0871	-0,15708	0,30251	-0,06582	0,30285	-0,83515	-1,10692	-0,50241	0,309419	0,493517

Hedgehog_Signaling_in_Mammals_Pathway	-0,56765	-0,48424	-0,82566	1,427425	2,656011	0,768336	-2,4931	-2,3254	-2,14687	-1,97917	-0,35082	-0,65872	-0,41663	-0,1894	-0,14455	1,968801	0,497826	2,2019	0,309202	0,493517
KEGG_HIF_1_signaling_Main_Pathway	-0,3172	-0,24051	-0,48236	-0,3521	-0,70825	-4,01562	0,245735	0,748948	-0,17071	1,020039	0,861351	-0,66994	-0,16045	-1,17697	0,380453	-2,45735	-0,29358	0,639407	0,309344	0,493517
NCI_Signaling_events_mediated_by_focal_adhesi	0,164928	0,133189	0,223932	-0,27701	-0,24224	0,086641	0,110191	0,3217	-0,20755	-0,26419	0,409434	0,072365	0,535425	0,498159	0,503684	0,652878	1,124066	1,392377	0,308867	0,493517
NCI_Nongenotropic_Androgen_signaling_Pathwa	0,075399	-0,05421	-0,15748	-0,38217	-0,50378	-0,18066	0,346054	0,510091	0,071123	-0,17519	-0,21235	-0,11379	-0,26752	-0,07	-0,07051	-1,55781	-1,21855	-0,78039	0,309761	0,493689
reactome_NGF_independant_TRKA_activation_M	-0,12341	-0,21365	0,036732	0,576998	0,695613	1,212204	0,370761	0,324468	0,532795	0,001223	-0,11437	0,302162	-0,0009	0,186489	0,335674	0,628115	0,992784	0,79518	0,309843	0,493689
ILK_Signaling_Pathway_Induced_Cell_Proliferatio	0,392306	-0,00251	0,084378	0,019335	0,095667	0,173713	0,051005	0,407552	-0,11519	-0,42732	0,456155	-0,2983	0,040155	0,037113	0,020004	-0,23771	-0,52108	0,00298	0,310351	0,494245
NCI_Arf6_trafficking_events_Pathway_(cell_adhe	-0,0158	-0,05935	-0,16771	0,291924	-1,19631	-0,18188	-2,86131	-2,74524	-3,36333	1,033019	0,707563	0,419997	0,398078	0,484287	0,05406	-2,29426	-2,42351	-2,56613	0,31105	0,495105
KEGG_Endometrial_cancer_Main_Pathway	-0,40294	-0,53552	-0,75429	-0,90954	-0,40556	-0,68227	-0,06666	0,191565	-1,08023	-0,45439	0,317533	2,546377	-0,45979	-0,32595	0,384777	-6,17547	-3,73469	-2,76088	0,312262	0,49678
fatty_acid_alpha-oxidation	0,023905	-0,09368	-0,00463	-0,16577	0,143476	-0,03162	-0,03905	0,10322	-0,02449	-0,15114	0,509658	0,166248	-0,02853	-0,24201	-0,19119	-1,31051	-1,06489	-0,9242	0,313221	0,497589
NCI_LPA_receptor_mediated_events_Main_Pathw	0,006675	-0,60778	-0,77036	-1,03342	-0,57226	-2,30463	-1,30749	-1,5048	-1,71093	0,104642	0,198705	-0,63142	-0,62264	-2,36049	-0,91967	-9,49864	-6,9404	-5,90316	0,313182	0,497589
reactome_SRP_dependent_cotranslational_prote	3,365634	0,97318	0,420392	-7,74917	-9,6999	-12,1988	7,91839	9,517691	4,355825	-6,68275	6,455631	4,47922	-2,20359	-1,6545	1,066834	-54,3925	-45,8768	-38,4068	0,31325	0,497589
reactome_Signaling_by_activated_point_mutants	-0,26068	-0,2933	-0,10628	-1,01886	-0,91214	-1,38903	-0,71136	-0,85245	-0,29669	-0,17825	-0,38081	0,400721	0,125594	0,044209	0,11678	-0,30605	-0,03724	-0,51882	0,314037	0,498587
KEGG_N_Glycan_biosynthesis_Main_Pathway	0,347782	-0,54532	-0,33512	0,22987	0,138778	-0,96148	-0,72087	-0,28878	-1,97373	1,13527	4,322744	0,971612	-0,85837	-1,23975	-0,81112	-10,9236	-9,38899	-8,86425	0,314336	0,498806
biocarta_activation_of_camp_dependent_protein	-0,7449	-0,07253	-0,05372	-1,33239	0,233989	0,062567	-1,50585	-2,3629	-0,576	3,486206	1,984049	-0,4859	-0,25786	0,885777	0,682171	1,225777	-0,25691	0,622839	0,314797	0,499284
reactome_G2_M_DNA_damage_checkpoint_Mair	0,121618	-0,01516	0,006325	0,673749	0,695222	0,544615	-0,00645	0,259647	-0,23043	0,473049	0,711471	0,116676	-0,14384	-0,40816	-0,39448	-1,43609	-0,8676	-0,62055	0,315102	0,499513
reactome_Inhibition_of_TSC_complex_formation	-0,05994	-0,0202	-0,03131	-0,38832	-0,4357	-0,35673	-0,2478	-0,22428	-0,27606	-0,40118	0,80185	-0,07019	0,134633	-0,05736	0,040007	-0,05389	-0,0449	-0,30251	0,316388	0,501297
coenzyme_A_biosynthesis	-0,01037	-0,05346	0,126027	0,429761	-0,4986	-0,28671	0,141639	0,099565	0,153484	-0,1456	0,054537	0,082971	0,513039	-0,01385	-0,14742	-0,47322	-0,63723	-0,86667	0,316784	0,501669
biocarta_nitric_oxide_signaling_Main_Pathway	0,026718	0,236712	0,244941	2,03746	1,631886	1,543789	0,804172	0,934465	1,089546	1,936881	0,87677	-0,43434	0,200394	-0,16164	-0,18588	4,049948	3,96057	3,530626	0,317449	0,501702
NCI_BARD1_signaling_events_Pathway_(DNA_dar	0,100595	0,03242	0,035579	0,352745	0,434231	0,377147	-0,05115	0,157846	-0,22585	0,075248	0,595761	0,27604	-0,26625	-0,23772	-0,14453	-1,1216	-0,78082	-0,58915	0,317319	0,501702
NCI_IL8_and_CXCR1_mediated_signaling_events_	0,00839	0,125834	0,000869	0,074982	0,124376	0,581567	-0,88918	-1,12174	-0,65727	0,446759	-0,00283	-0,52991	-0,31532	-0,30811	-0,18697	0,51059	0,764176	0,414559	0,317339	0,501702
NCI_Syndecan_1_mediated_signaling_events_Ma	-0,8644	-0,93593	-0,42243	-2,79158	-3,19797	-2,78047	-2,17369	-2,82094	-0,85652	-1,64686	-0,82812	0,460758	0,405889	0,571733	0,7976	1,529276	0,816667	-0,27749	0,317432	0,501702
Calcium2+_Signaling	0,130041	-0,1774	-0,26311	-0,11632	-0,2373	-0,44783	-0,90933	-0,72137	-1,60697	-2,10011	-0,43813	-2,19635	-0,26143	-0,69539	-0,02669	-0,80686	-0,19822	0,329568	0,31764	0,501748
biocarta_stress_induction_of_hsp_regulation_Pat	-0,04192	-0,07132	-0,01569	-0,23771	-0,08025	0,238032	-0,28922	-0,22182	0,118678	-0,03205	0,092266	0,084422	0,178505	-0,04416	-0,07046	0,027	0,345963	0,503356	0,319287	0,503839
biocarta_stress_induction_of_hsp_regulation_Pat	-0,04192	-0,07132	-0,01569	-0,23771	-0,08025	0,238032	-0,28922	-0,22182	0,118678	-0,03205	0,092266	0,084422	0,178505	-0,04416	-0,07046	0,027	0,345963	0,503356	0,319287	0,503839
serotonin_and_melatonin_biosynthesis	0,14127	-0,09342	0,218107	-0,10536	-0,86514	-0,41063	-1,5502	-1,75154	-1,2724	-0,28509	-0,78545	-0,22345	0,425408	0,169112	0,366967	1,096476	0,865006	1,331281	0,320027	0,504752
Akt_Signaling_Pathway_Elevation_of_Glucose_Im	-0,0916	-0,20838	0,373081	-1,37555	0,371393	-1,26673	0,22418	0,094386	0,404121	0,009285	1,327149	-0,21552	0,579088	0,540815	0,396363	0,101344	0,837496	0,770875	0,320518	0,505271
biocarta_rho_cell_motility_signaling_Main_Pathw	-0,40114	-0,24246	-0,07455	-1,92825	-2,67177	-1,88872	-1,52902	-2,00399	-1,49705	-1,08247	0,430004	0,81104	0,666056	0,274179	0,578796	0,510198	-0,49554	-0,66214	0,321188	0,50607
guanosine_nucleotides_ide_novoi_biosynthesis	0,201724	-0,09312	-0,11618	-0,58498	-0,65034	-0,5689	0,538811	0,772315	-0,06654	1,623036	2,545126	1,406566	0,760666	0,841203	1,138071	-5,53242	-4,39349	-3,99977	0,321571	0,506161
NCI_Signaling_events_mediated_by_VEGFR1_and	0,380317	-0,94037	-1,11399	-1,85657	-0,13023	-0,9346	-7,06076	-6,23406	-8,29599	3,452502	0,680358	0,947683	0,121396	-0,14787	0,65311	-8,10775	-6,52827	-4,76348	0,321484	0,506161
reactome_DNA_Damage_Telomere_Stress_Induc	0,078741	-0,39104	-0,80598	1,126794	-0,1836	-1,10663	3,94949	4,733369	2,702025	2,593194	1,031856	0,537785	-0,26083	-0,57018	0,927169	-5,59477	-2,59878	-3,47013	0,322189	0,506878
reactome_Activation_of_the_phototransduction_	-0,08967	-0,08376	0,054018	0,567705	1,192073	0,303157	0,368664	0,045518	0,983851	1,5515	0,883893	-0,77515	-0,41311	-0,68481	-0,68313	1,393168	0,059045	1,292396	0,322652	0,507351
biocarta_activation_of_csk_by_camp_dependent_	-0,60337	-0,32982	-0,19899	-0,74075	0,098041	-0,20838	-1,90313	-2,55872	-1,82544	2,485592	1,323415	-0,92439	0,595499	1,90365	1,324825	-0,19007	-1,47307	-0,98343	0,322947	0,507558
GDP-L-fucose_biosynthesis_II_from_L-fucose	0,032652	-0,02017	-0,05243	0,035463	-0,24522	0,354709	0,182349	0,266712	0,122655	0,320098	-0,00788	-0,10762	0,249022	0,054936	0,097951	-0,4142	-0,05287	0,131099	0,323836	0,508698
reactome_betaKlotho_mediated_ligand_binding_	-0,09221	-0,07457	-0,03871	-0,19057	-0,08129	0,152497	-0,09775	-0,19307	0,27709	-0,16055	-0,01875	-0,30941	-0,05321	0,323335	-0,19556	0,8404	0,615652	0,709469	0,324243	0,509081
biocarta_pkc_catalyzed_phosphorylation_of_inhil	-0,07539	-0,44999	-0,07028	1,39011	0,618437	0,230157	-1,30939	-1,77041	-1,59846	-0,77393	0,179018	0,052172	-0,14883	-0,69371	0,021236	0,069619	-0,22853	0,421931	0,324749	0,509486
PTEN_Pathway_DNA_Repair	-0,7196	-0,20027	-0,29384	0,067175	-0,27899	1,396797	7,84556	7,750374	8,072527	-1,85657	-5,25083	-2,96589	0,34206	0,043987	0,113845	12,42158	12,72394	11,46969	0,324828	0,509486
biocarta_multiple_antiapoptotic_pathways_from_	0,115403	-0,14737	-0,15555	-0,16241	-0,10779	-0,51012	-1,39919	-1,10934	-1,45161	0,186868	-0,27443	0,137829	-0,01086	-0,13354	-0,12967	-1,73217	-1,20897	-0,67269	0,325543	0,509838
KEGG_Small_cell_lung_cancer_Main_Pathway	-0,34267	-0,55884	-0,36836	-2,866	-1,82858	-1,13774	-3,36564	-3,26586	-4,05838	3,56742	-0,31738	2,949326	-0,84905	-0,40019	-0,0223	-2,55151	-2,69468	-2,38067	0,325312	0,509838
KEGG_Synaptic_vesicle_cycle_Main_Pathway	-0,15787	-0,06377	-0,2143	0,103358	-0,10534	-0,12682	-1,68341	-1,69035	-1,6536	-1,01363	-0,55886	-0,52007	0,264753	0,364945	0,13576	2,844159	2,470979	3,849544	0,325508	0,509838
CDK5_Pathway_in_Central_Nervous_System	-0,82828	-0,82496	-0,98977	-1,0565	-0,41783	-0,61336	-4,68759	-4,25803	-4,6595	-0,7959	0,770099	2,348741	-0,60169	-0,19543	0,398251	-4,46857	-2,69502	-2,75166	0,326667	0,511084
reactome_ATP_sensitive_Potassium_channels_M	0,027806	0,054611	0,239098	-0,45599	0,449891	-0,07429	0,606794	0,746934	0,725309	0,629343	-0,00462	-0,12989	0,404036	0,075985	0,306731	0,780411	0,613201	0,514053	0,326597	0,511084
NCI_Stabilization_and_expansion_of_the_E_cadh	-0,08899	0,153502	0,047202	0,441354	0,044749	-0,308	-1,06847	-1,22477	-1,11941	-0,10961	0,841395	0,300878	-0,30823	0,115344	-0,07324	-2,23788	-1,06118	-1,05222	0,326831	0,511084
reactome_GP1b_IX_V_activation_signalling_Main	-0,13589	-0,15022	-0,07192	-0,23314	-0,6291	-0,27212	-0,25487	-0,51303	0,183211	0,581474	0,440991	-0,04263	0,246299	-0,13157	0,11948	-0,27427	-0,31978	0,13327	0,327126	0,511288
NCI_CXCR4_mediated_signaling_events_Pathway	-0,03145	-0,05789	-0,06576	-0,03331	-0,26203	-0,26062	0,045073	0,035593	0,048906	-0,24123	-0,17729	0,025834	-0,14597	-0,09257	-0,16758	-0,29038	-0,11813	-0,01275	0,328114	0,512575
NCI_LPA_receptor_mediated_events_Pathway_(c	0,415805	-0,03885	-0,10601	0,399073	-0,7121	0,207287	0,82116	0,6938	0,547692	0,087771	0,950694	0,005463	-0,56192	-1,2997	-1,02626	-5,18402	-4,74507	-3,86886	0,328282	0,51258
wybutosine_biosynthesis	0,09889	0,123971	0,007504	0,020834	0,061703	-0,89894	0,35715	0,597239	0,073657	-0,40989	-0,12757	0,51323	0,282178	-0,01142	0,245793	-1,40483	-0,95187	-0,7663	0,32846	0,512602
reactome_PI_3K_cascade_Main_Pathway	-0,66069	-0,43157	-0,28567	-1,06988	0,240262	0,027715	-1,12085	-1,50467	-0,03255	0,071379	-1,21873	-0,20433	-0,33896	-0,08613	-0,25285	-0,84042	-0,45706	-0,27086	0,329182	0,513471
biocarta_erk1_erk2_mapk_signaling_Main_Pathw	-0,2811	-0,42432	-0,52227	-1,52961	-1,45072	-1,83473	-1,44792	-1,52242	-1,4856	0,245443	1,571622	0,750421	0,452519	-0,10951	0,745137	-1,10351	-0,64332	-0,24463	0,330135	0,514701
catecholamine_biosynthesis	-0,12405	-0,00092	-0,01465	0,142131	-0,23413	0,048971	-0,19018	-0,03366	0,429079	-0,80911	-0,35603	-1,06788	-0,02075	-0,20078	-0,44994	1,228522	1,500726	1,887488	0,331223	0,515295
NCI_EPO_signaling_Pathway_(cell_proliferation)	0,15748	0,146824	0,064917	-0,30541	-0,31753	0,049426	-0,09628	-0,08871	-0,2912	-0,47464	0,212756	-0,30337	-0,15021	-0,12242	-0,011	-1,13186	-0,64633	-0,97928	0,331344	0,515295
NCI_Signaling_events_mediated_by_focal_adhesi	0,081542	0,077688	0,03105	-0,30065	-0,34319	-0,06612	0,406729	0,633525	0,134457	-0,52614	0,021304	0,171174	0,283427	0,220353	0,315155	-0,84903	-0,17097	-0,1764	0,331068	0,515295
purine_nucleotides_degradation	0,01413	-0,09076	-0,11976	0,485732	-0,01889	-1,15232	0,961115	1,096422	0,690549	0,16711	0,104568	-0,05613	0,448712	0,208972	0,429308	-0,76574	-0,84461	-0,47244	0,330946	0,515295
reactome_Catecholamine_biosynthesis_Main_Pat	-0,12405	-0,00092	-0,01465	0,142131	-0,23413	0,048971	-0,19018	-0,03366	0,429079	-0,80911	-0,35603	-1,06788	-0,02075	-0,20078	-0,44994	1,228522	1,500726	1,887488	0,331223	0,515295
NCI_Angiopoietin_receptor_Tie2_mediated_signa	0,057119	-0,16926	-0,25531	-0,85526	-0,89966	-0,59007	-2,28469	-2,42403	-2,39471	1,144713	-0,1523	-1,10622	0,147099	-0,30719	-0,06326	-0,8373	-0,44984	0,493468	0,331511	0,515298
reactome_Cleavage_of_the_damaged_pyrimidine	-0,09404	-0,06315	-0,03581	0,020732	-0,31104	0,289485	0,12667	-0,0124	0,11043	0,399125	0,128886	0,341407	-0,02963	0,046923	-0,0863	-1,47688	-0,90318	-0,39659	0,332939	0,517001
reactome_Recognition_and_association_of_DNA_	-0,09404	-0,06315	-0,03581	0,020732	-0,31104	0,289485	0,12667	-0,0124	0,11043	0,399125	0,128886	0,341407	-0,02963	0,046923	-0,0863	-1,47688	-0,90318	-0,39659	0,332939	0,517001
NCI_Regulation_of_nuclear_SMAD2_3_signaling_	-0,05959	-0,10127	-0,1192	0,261332	-0,11971	-0,02485	-0,24838	-0,26581	-0,2421	-0,09801	0,281304	0,283868	0,006815	0,129559	0,115156	0,014936	-0,36014	-0,07449	0,334177	0,518408
reactome_Proton_oligopeptide_cotransporters_M	0,007366	-0,0949	-0,04464	-0,57564	-0,24118	0,042563	0,944751	1,027904	0,876739	-0,3912	0,040975	0,119062	0,116615	0,243972	-0,10849	-0,46587	-0,6871	-0,73104	0,33407	0,518408
NCI_Integrins_in_angiogenesis_Pathway_(angioge	-0,0825	-0,18281	0,046799	-2,7901	-2,74816	-2,85113	-3,2732	-3,22552	-3,39008	1,29151	-0,55314	-0,41448	0,720733	0,934377	0,759463	-0,74919	-0,22995	-0,22255	0,335216	0,519761
C20_prostanoid_biosynthesis	-0,18233	0,231876	0,022047	-1,24833	-1,01733	-1,04217	-0,37065	-0,52359	-0,12542	-0,4014	-0,02775	0,704345	0,345151	0,568652	0,794901	0,433011	0,06751	0,492951	0,336331	0,52123
reactome_Cation_coupled_Chloride_cotransporte	-0,29951	-0,17317	-0,03665	0,138573	0,32093	0,123998	-0,14893	-0,11892	0,028367	0,277588	-0,27954	1,083246	0,090769	0,071232	0,333313	0,466217	1,344056	1,385009	0,3372	0,522285
reactome_formation_of_the_beta_catenin_TCF_t	-0,0302	-0,5341	-0,74208	-0,27445	-0,7715	-3,57949	4,048434	4,874556	2,825707	1,970397	1,341214	-0,05629	0,041293	-0,64686	1,196777	-9,17791	-6,33134	-5,56384	0,337435	0,522285
reactome_Oncogene_Induced_Senescence_Main	0,161415	0,21175	0,182155	2,04201	0,270879	0,623927	0,464838	0,278264	0,974556	2,338018	2,075413	2,347418	-0,29073	-0,44607	-0,19137	-0,24767	0,446785	0,165051	0,337515	0,522285
NCI_Caspase_Cascade_in_Apoptosis_Pathway_(n	0,059968	0,045056	0,034521	-0,42324	-0,22958	-0,04127	-0,47722	-0,28986	-0,56405	0,249604	-0,02826	-0,00454	0,021529	-0,03216	-0,32834	-1,92402	-1,31609	-1,16452	0,33805	0,522849
NCI_Osteopontin_mediated_events_Main_Pathw	0,019934	-0,38245	-0,50951	-1,12249	-1,8999	-2,17963	-2,49525	-1,88537	-3,37955	-0,23627	-1,34445	-1,29498	0,148943	0,29306	0,455405	-4,02688	-2,77336	-1,76075	0,338215	0,522849
reactome_Small_interfering_RNA_siRNA_biogene	0,089711	-0,09105	-0,06929	-1,35516	-0,72098	-1,23353	0,443704	0,502819	0,051851	-1,28498	-0,60893	-0,97673	0,464919	0,609322	0,46067	-1,30321	-1,19702	-1,05394	0,338518	0,523057
KEGG_Antigen_processing_and_presentation_Ma	-0,52113	0,066436	0,517757	-3,19118	-4,17841	-1,66611	-1,41571	-2,12701	-0,26352	1,214032	1,8978	-1,20036	-0,87062	-1,95836	-0,25687	-3,63915	-4,34961	-3,56436	0,340287	0,525137
NCI_Signaling_events_mediated_by_the_Hedgeh	-0,05191	-0,07167	-0,09445	-0,22695	0,338816	-0,03129	0,011959	-0,04633	0,181578	0,550348	0,260195	-0,36697	-0,0027	-0,25251	0,025064	0,171502	0,295807	0,204817	0,340238	0,525137
reactome_Coenzyme_A_biosynthesis_Main_Path	0,035128	-0,19584	0,070542	0,359998	-0,4578	-0,42903	0,258008	0,297102	0,071125	0,151301	0,58026	-0,146	0,578062	0,229853	-0,09054	-0,5596	-0,70964	-1,11885	0,340434	0,525137
reactome_COPII_Coat_Protein_2_Mediated_Vesi	0,163766	-0,14483	0,028537	-1,25721	-0,92013	-1,01592	-1,45791	-1,20494	-1,79419	-1,11807	0,111489	-0,71365	-0,37815	0,013044	-0,13845	-3,65604	-2,542	-2,21844	0,340539	0,525137
NCI_PDGFR_alpha_signaling_Main_Pathway	-0,24781	-0,33042	-0,59422	-2,31013	-2,51918	-2,12871	-3,44292	-3,29156	-3,85001	-0,25287	-1,61748	0,059633	0,044942	-0,3551	-0,10484	-2,09248	-0,77906	-0,18213	0,341213	0,525917
NCI_Alpha4_beta1_integrin_signaling_events_Ma	-0,18556	-0,06833	-0,31574	-1,12089	-0,25154	-1,63187	-0,59354	-0,12391	-0,71635	1,10699	0,590582	1,208935	-0,8838	-0,24208	-0,55348	-2,74408	-1,31927	-1,06809	0,342581	0,526203
NCI_Arf6_trafficking_events_Pathway_(positive_r	-0,05456	-0,17294	-0,12069	-0,35969	-0,86583	0,014415	-0,1399	-0,14751	-0,23059	-0,019	-0,24283	-0,17598	0,201963	0,086316	-0,00687	-0,79392	-0,71028	-0,87939	0,342397	0,526203
NCI_N_cadherin_signaling_events_Pathway_(apo	-0,08134	-0,10135	-0,07301	-0,20876	0,437767	-0,28315	-0,5035	-0,59935	-0,7459	0,353488	0,941533	0,611949	0,120681	0,021884	0,188229	-1,96754	-1,29818	-0,96575	0,342278	0,526203
NCI_Rapid_glucocorticoid_signaling_Main_Pathw	-0,05386	-0,10083	-0,10924	-0,68993	-0,68647	-0,64078	-0,46755	-0,37695	-0,45661	-1,07799	-0,74642	-0,86927	0,240735	-0,18059	-0,02837	-0,7053	-0,6058	-0,53614	0,34201	0,526203
NCI_Rapid_glucocorticoid_signaling_Pathway_(glu	-0,05386	-0,10083	-0,10924	-0,68993	-0,68647	-0,64078	-0,46755	-0,37695	-0,45661	-1,07799	-0,74642	-0,86927	0,240735	-0,18059	-0,02837	-0,7053	-0,6058	-0,53614	0,34201	0,526203
reactome_Phospholipase_C_mediated_cascade_M	-0,65059	-0,32049	-0,10917	-0,70109	0,283311	-0,40205	-0,5255	-1,04985	0,721716	0,627359	-1,11924	-0,83509	-0,53303	-0,45569	-0,64468	0,606847	0,466698	-0,05885	0,342577	0,526203
urate_biosynthesisinosine_5-phosphate_degradat	-0,03019	-0,09664	0,028366	-0,17	-0,29803	-1,12428	0,014865	-0,03895	-0,08245	0,859219	0,591905	0,984624	0,240434	0,189628	0,299502	-0,35426	-0,11342	-0,61348	0,342145	0,526203
molybdenum_cofactor_biosynthesis	-0,0496	-0,06758	-0,10701	-0,0499	-0,03195	0,401575	0,550013	0,638302	0,280351	1,243953	0,509342	0,854982	0,326899	0,166161	0,216457	-1,41925	-1,31593	-1,01253	0,344152	0,528355
Akt_Signaling_Pathway_NF-kB_dependent_transc	-0,03302	-0,20254	0,247423	-1,35409	0,196628	-1,41291	0,329634	0,166298	0,328264	0,269996	1,236658	-0,42996	0,569573	0,712994	0,260518	-0,27811	0,801986	0,797709	0,344829	0,52841
Cytoskeleton_Reorganization	-1,09796	-1,05188	-1,02429	-4,74986	-4,8171	-3,22077	-6,80235	-6,96631	-7,12882	-0,13426	-4,17031	1,571226	0,992012	1,804627	1,547986	-9,7813	-7,59999	-5,40497	0,345036	0,52841
D-galactose_degradation_V_Leloir_pathway	-0,02458	0,049945	0,162018	-0,36385	-0,31813	-0,38964	0,63887	0,736132	0,579298	-1,78644	-0,07548	-0,39246	-0,24464	-0,15924	0,056904	-0,80857	-0,58019	-0,56258	0,345018	0,52841
KEGG_Ovarian_steroidogenesis_Main_Pathway	-0,53567	0,318087	0,103511	-0,55456	-2,37491	-2,02896	-1,37684	-1,49259	-0,65864	-1,99985	-1,45279	-0,21635	0,160605	1,344803	0,449967	1,993803	2,320753	1,938837	0,344504	0,52841
reactome_GABA_synthesis_release_reuptake_and	-0,40992	-0,24621	-0,37497	-0,57565	-0,27782	-0,81216	-1,56501	-1,48924	-1,55032	-1,80508	-1,78826	-1,19824	0,126933	0,114507	0,265218	3,778929	3,72355	4,317915	0,344833	0,52841
KEGG_Histidine_metabolism_Main_Pathway	-0,20112	-0,30848	-0,07071	-1,37998	-0,9162	-0,39947	-0,7388	-1,25804	-0,02215	-0,73366	-1,20493	0,30565	0,914021	0,307219	0,326118	1,341082	1,460546	2,227899	0,34667	0,530391
reactome_Nonsense_Mediated_Decay_NMD_enh	3,28858	0,616226	0,391713	-9,47393	-11,013	-13,267	8,279847	10,1308	4,622431	-5,2684	4,853203	2,563655	-1,16325	-0,86292	1,673355	-49,6248	-41,72	-35,9503	0,346611	0,530391
KEGG_Other_glycan_degradation_Main_Pathway	0,256543	0,312059	0,173775	-1,24633	-1,90341	-1,22981	-1,43942	-1,23252	-1,70569	0,536667	-1,24353	1,192965	-0,10111	-0,62093	0,326535	-1,23318	-1,47295	-1,4786	0,347531	0,531448
biocarta_rac1_cell_motility_signaling_Pathway_(a	-0,33939	-0,1752	-0,03919	-0,6331	-0,59654	0,680961	-0,95116	-1,35862	-0,85329	-0,42968	0,210118	0,661934	0,227125	0,264735	0,221154	2,265884	0,26178	0,921033	0,347961	0,531844
NCI_Direct_p53_effectors_Main_Pathway	-0,03012	0,034037	-0,04512	1,001422	0,667809	0,451775	-0,47746	-0,62758	-0,67041	-0,03254	0,349449	0,017327	-0,30784	-0,12502	-0,08899	0,136362	0,15042	-0,18415	0,348952	0,532575
NCI_Thromboxane_A2_receptor_signaling_Pathw	-0,1987	-0,1149	0,0125	-0,64531	-1,06199	-1,06288	-0,78478	-1,09208	-0,38638	-1,38285	-0,59252	-0,61515	-0,81802	-0,46778	-0,07102	-1,14142	-0,40704	0,361873	0,348646	0,532575
p38_Signaling_Pathway_Translation	0,090264	-0,01773	-0,21349	-0,16676	-0,16886	-0,24512	-0,27554	-0,33953	-0,32978	0,067501	0,318777	-0,02055	0,10498	0,250595	0,306687	-0,65232	-0,17797	-0,49795	0,348914	0,532575
Akt_Signaling_Pathway_Induction_of_Chromatin_	-0,00345	-0,1567	0,301261	-1,19442	0,472632	-1,36479	0,168405	0,141973	0,343453	0,492288	1,186026	-0,56272	0,55514	0,563619	0,176526	-0,27849	0,908676	0,654251	0,349201	0,532694
Akt_Signaling_Pathway_Tumor_Supression	-0,04761	-0,15395	0,270499	-1,33909	0,196047	-1,21965	-0,2066	-0,29841	0,032135	0,115633	1,093403	-0,3686	0,681252	0,601863	0,31431	0,021471	0,789592	0,804747	0,349798	0,533343
KEGG_African_trypanosomiasis_Main_Pathway	0,155723	-0,15776	0,071293	-0,19451	0,262671	-1,14384	-0,45874	-0,30617	-0,46178	-0,79917	-0,32813	-1,12396	0,069961	0,204415	0,686532	1,84251	2,064464	2,596421	0,350542	0,533758
NCI_amb2_Integrin_signaling_Pathway_(leukocyt	-0,08811	-0,28561	-0,136	0,52422	-0,28689	-0,10154	-0,50785	-0,71657	0,054571	-0,76381	-0,14739	-0,42312	0,765538	0,209843	0,882268	1,081146	0,471662	0,84023	0,350288	0,533758
NCI_Regulation_of_Androgen_receptor_activity_I	-0,15408	-0,07071	-0,26782	0,272874	-0,27632	0,693717	0,20908	0,359611	-0,42934	-0,98599	-0,59308	-0,63299	0,004003	0,328389	0,302909	0,007808	1,077244	1,165461	0,350584	0,533758
reactome_Synthesis_of_dolichyl_phosphate_man	0,005056	-0,05986	0,019309	0,216764	-0,27521	-0,448	0,2413	0,223865	0,185978	-0,29074	0,483729	-0,05158	0,095333	0,016334	0,081569	-0,96166	-0,61071	-0,54087	0,350891	0,533965
NCI_Alpha_synuclein_signaling_Pathway_(neuron	-0,16891	-0,25262	-0,00289	0,03734	1,652863	0,47345	-2,48043	-2,66156	-2,22166	-0,70657	-0,39863	-0,09357	-0,16982	0,256148	0,602205	1,507669	0,008051	1,236863	0,351104	0,534027
biocarta_nerve_growth_factor_pathway_ngf__M	-0,24667	-0,41835	-0,45923	-0,17201	-0,55323	-0,35946	-0,73208	-0,52456	-1,09745	-0,39738	0,544564	0,750872	0,145338	-0,43818	0,149273	-2,7012	-1,16072	-0,90248	0,351759	0,534763
creatine_biosynthesis	0,036106	0,009506	0,010513	0,209704	0,120941	0,1564	-1,02044	-0,94652	-1,0919	0,525804	0,059501	0,107248	-0,75023	-0,5527	-0,46938	-0,40028	-0,28292	-0,25345	0,351943	0,534782
KEGG_Epithelial_cell_signaling_in_Helicobacter_p	0,166388	-0,14257	0,021351	-0,85891	0,313806	-0,02388	-1,04953	-0,74595	-1,39776	0,538224	-1,01054	0,235538	0,547141	0,046459	0,570554	1,488873	1,038885	1,614434	0,352926	0,535688
KEGG_Lipoic_acid_metabolism_Main_Pathway	0,070546	0,115036	0,012247	0,187629	-0,51622	-0,2859	-0,33219	-0,25882	-0,57727	-0,95718	-0,569	-0,12589	-0,05793	-0,15186	-0,00978	-0,25057	-0,38427	-0,49049	0,353166	0,535688
NCI_Nectin_adhesion_Main_Pathway	0,002399	-0,05743	-0,01794	-1,31067	-0,62878	-1,67155	-1,70447	-1,53394	-2,10777	-0,194	0,16891	1,415812	0,411886	0,67229	0,920568	-4,17892	-3,19183	-3,18524	0,353227	0,535688
reactome_Scavenging_by_Class_F_Receptors_Ma	-0,07343	-0,02467	0,058334	0,177919	0,576359	0,525846	0,010616	0,019808	0,099342	0,293249	0,383114	0,550155	-0,25981	0,189475	-0,27372	-1,02576	-0,48025	-0,17598	0,352818	0,535688
biocarta_rho_cell_motility_signaling_Pathway_(ac	-0,26762	-0,16272	0,04273	-1,58534	-1,83829	-1,14049	-0,47441	-0,84442	-0,31575	-1,27462	0,559702	0,656258	0,58848	0,382397	0,71335	1,134626	0,026552	-0,31967	0,353872	0,536208
biocarta_the_igf_1_receptor_and_longevity_Path	0,061583	-0,13444	-0,04551	0,423515	0,016655	-0,25143	-1,35567	-1,28956	-1,59366	0,772586	-0,35144	0,157198	0,146737	0,031856	0,044622	-1,01645	-0,49161	0,046051	0,354235	0,536208
NCI_Caspase_Cascade_in_Apoptosis_Pathway_(JN	0,004557	0,081079	0,097354	-0,34112	0,004729	0,12651	-0,55905	-0,40096	-0,56352	0,149396	0,175164	-0,03914	-0,08704	-0,06836	-0,29356	-1,22452	-1,20357	-0,90018	0,354431	0,536208
NCI_Regulation_of_nuclear_SMAD2_3_signaling_	-0,70734	-0,54629	-0,43968	0,211802	-0,83553	0,07964	-3,44377	-4,02684	-3,07501	-1,41229	-1,80572	-1,29952	-0,3327	-0,37653	-0,01516	5,294803	3,346512	3,548116	0,354389	0,536208
reactome_Rap1_signalling_Main_Pathway	-0,1156	-0,08173	-0,09725	-0,48092	-0,18989	-1,50928	-0,43325	-0,42145	-0,66268	-2,63599	-1,24341	-0,48426	-0,2593	-0,09383	-0,22354	-1,48873	-0,95759	-0,15865	0,354357	0,536208
biocarta_aspirin_blocks_signaling_pathway_involv	0,037534	-0,32408	-0,3124	-1,10808	-1,38875	-1,83243	-0,32308	-0,09746	-0,42353	-1,1291	0,109929	0,092987	0,526618	0,335175	0,885493	-2,68941	-3,08976	-2,01961	0,355234	0,536381
ILK_Signaling_Pathway_Cell_Cycle_Proliferation	-0,53653	-0,50242	-0,2547	-0,47365	0,002531	-0,14601	-3,63616	-3,07764	-3,25981	2,54225	0,24139	3,382412	0,285167	0,455274	1,113465	-4,01753	-3,53579	-3,33607	0,354977	0,536381
ILK_Signaling_Pathway_G2_Phase_Arrest	-0,53653	-0,50242	-0,2547	-0,47365	0,002531	-0,14601	-3,63616	-3,07764	-3,25981	2,54225	0,24139	3,382412	0,285167	0,455274	1,113465	-4,01753	-3,53579	-3,33607	0,354977	0,536381
mitochondrial_L-carnitine_shuttle	-0,0116	-0,05454	-0,01501	-0,48021	-0,90115	-0,55055	-0,60934	-0,56014	-0,57438	0,063141	0,098131	-0,03292	0,350558	-0,04806	-0,24403	-0,27569	-0,51836	-0,35519	0,355212	0,536381
Akt_Signaling_Pathway_Enhancement_of_Breast_	0,004984	-0,14213	0,29804	-1,24253	0,341495	-1,33909	0,163357	0,114177	0,27597	0,289644	1,303049	-0,28904	0,671756	0,574602	0,274695	-0,22217	0,595212	0,759519	0,355938	0,537005
KEGG_Serotonergic_synapse_Main_Pathway	0,148482	0,336506	0,056955	3,381722	3,042291	1,896393	1,558087	0,674015	2,472084	-2,06921	1,361835	1,60008	-0,16668	-0,01585	-0,04066	1,357533	-0,37662	2,665174	0,356308	0,537005
NCI_CD40_CD40L_signaling_Main_Pathway	0,185324	0,090654	-0,20854	-1,82041	-1,61855	-0,55564	-1,19298	-0,52089	-1,88755	-0,54707	0,413729	0,710943	1,125259	0,646113	1,238881	-3,36603	-2,84363	-2,49099	0,356429	0,537005
reactome_p75NTR_recruits_signalling_complexes	0,159003	-0,13095	-0,00432	-0,55958	-0,33662	-1,05722	-1,42145	-1,39978	-1,69825	0,90524	0,30254	0,594766	0,648071	-0,18105	0,240995	-3,71571	-3,19916	-2,26744	0,356483	0,537005
thiosulfate_disproportionation_III_rhodanese	-0,00876	-0,0381	0,020979	-0,1981	0,195713	0,20233	0,138417	0,130078	0,128377	0,540588	0,079711	0,604878	-0,05756	-0,0554	-0,152	-0,00626	-0,17076	-0,1196	0,356509	0,537005
EGF_Pathway	-0,96763	-1,05956	-1,17006	-4,22271	-1,96404	-2,42503	-4,77341	-3,54351	-6,86154	1,988643	0,043507	-1,60861	-0,03625	0,733774	1,237301	-10,7736	-7,95349	-2,30313	0,357233	0,537835
KEGG_Taste_transduction_Main_Pathway	-0,36361	0,049571	-0,17249	-0,56901	-0,1554	-0,718	-1,4047	-1,46259	-1,07324	-1,80974	-0,40492	1,022725	-0,51637	0,238744	-0,10304	0,838247	0,097134	0,794071	0,358532	0,539161
protein_citrullination	-0,06173	0,015652	0,130054	0,840189	0,911486	0,42612	-0,11721	-0,24163	0,366732	-0,42987	-0,0297	-0,02233	0,125929	0,17676	0,105043	0,836967	0,870673	0,725347	0,358328	0,539161
reactome_Vitamins_B6_activation_to_pyridoxal_	-0,08548	-0,07695	-0,02381	-0,06986	0,623865	0,010651	0,184508	0,107142	0,201704	0,592315	0,207323	0,341195	-0,03321	0,06646	-0,18758	0,549469	-0,00887	0,392697	0,358633	0,539161
reactome_Beta_oxidation_of_pristanoyl_CoA_Ma	0,172807	0,03039	-0,02517	-1,00537	-0,4768	0,239487	-0,52196	-0,35175	-0,73647	-0,69721	-0,24147	0,451387	0,470064	0,435965	0,785511	-0,88525	-0,62013	-0,24969	0,359601	0,540355
biocarta_role_of_brca1_brca2_and_atr_in_cance	0,078466	-0,10677	-0,10325	0,3004	0,408777	0,157267	-0,45558	-0,12306	-0,77414	0,383962	0,975432	0,585976	-0,10545	-0,17584	-0,29634	-2,00392	-1,31045	-1,03811	0,35997	0,54051
NCI_IL12_mediated_signaling_events_Main_Path	0,066231	0,086905	0,310966	-3,14789	-0,90846	-1,99025	0,285254	-0,54939	0,216524	0,078287	-0,45136	0,244093	0,877798	0,417839	1,530005	0,670364	0,096331	0,79488	0,360051	0,54051
reactome_TRAF6_Mediated_Induction_of_proinf	0,227171	0,054573	0,064455	-0,32753	-0,39251	-0,56984	0,417012	0,428903	0,233663	-0,14463	0,586906	0,403653	0,094986	0,07073	0,101822	-2,52008	-2,00645	-1,75506	0,361322	0,542157
NCI_E_cadherin_signaling_in_the_nascent_adher	0,025669	0,015452	0,03647	-0,39819	-0,39114	-1,08201	-0,86732	-0,7489	-1,18154	-0,19011	1,058433	0,321951	-0,43016	-0,1018	-0,16561	-4,47849	-3,48119	-3,10434	0,361622	0,542346
EGF_Pathway_IP3_Signaling	-0,00527	-0,01366	-0,06179	-0,2401	-0,13845	-0,86375	0,946791	1,021281	0,726306	-0,37174	-0,20169	-0,76243	0,093014	-0,23202	0,146893	-0,81337	-0,72647	-0,47589	0,362057	0,542737
NCI_Atypical_NF_kappaB_Pathway_(Pathway_deg	-0,03545	-0,08537	-0,10669	-0,82582	0,366664	-0,3955	-0,47219	-0,35408	-0,66799	-0,32837	-1,22147	0,717266	0,361661	-0,07746	0,26214	-0,43593	-0,78114	-0,45643	0,362345	0,542907
KEGG_Long_term_potentiation_Main_Pathway	0,289962	-0,2282	-0,05761	1,578254	2,152269	-0,3209	-5,26475	-4,71199	-5,90855	-4,1799	0,966364	-0,80885	-0,97716	-0,52622	0,285079	-1,85153	0,494099	2,011125	0,362999	0,543357

NCI_Urokinase_type_plasminogen_activator_uPA	-0,04411	-0,04404	-0,13763	0,103906	-0,36996	0,528429	-1,20655	-1,2212	-0,95218	-1,06457	-0,51621	-0,83755	-0,01451	0,060566	0,12836	-0,05736	0,671346	0,709966	0,36316	0,543357
reactome_Arachidonate_production_from_DAG_	-0,08295	-0,12192	-0,0472	-0,05813	-0,25716	-0,63014	-0,68706	-0,64933	-0,80182	-0,17912	0,127505	-0,30482	-0,03808	0,143404	0,265942	0,487594	0,339465	0,270291	0,363168	0,543357
NCI_E_cadherin_signaling_in_the_nascent_adher	-0,06351	-0,00736	-0,02201	-0,47816	0,597167	-0,52001	0,568318	0,673637	0,613297	0,790224	0,307412	-0,07981	-0,80691	-0,45332	-0,41448	-1,07576	-0,68213	-0,02874	0,364876	0,54565
NCI_mTOR_signaling_Pathway_(regulation_of_ac	0,071162	-0,02004	-0,05338	-0,3218	-0,45646	-0,55963	-0,88198	-0,9154	-1,03198	-0,11091	-0,21356	-0,64962	0,241948	0,06361	0,662011	-0,41281	-0,27586	-0,09421	0,366148	0,547027
NCI_mTOR_signaling_Pathway_(regulation_of_pr	0,071162	-0,02004	-0,05338	-0,3218	-0,45646	-0,55963	-0,88198	-0,9154	-1,03198	-0,11091	-0,21356	-0,64962	0,241948	0,06361	0,662011	-0,41281	-0,27586	-0,09421	0,366148	0,547027
KEGG_Regulation_of_autophagy_Main_Pathway	0,382555	0,11396	-0,07304	0,288706	-0,09113	-0,15957	-1,78758	-1,64699	-2,28654	1,179024	1,916474	0,868958	0,046632	0,185986	0,331402	-4,08133	-3,04364	-2,35962	0,366735	0,547642
NCI_Regulation_of_Androgen_receptor_activity_I	0,048728	0,034852	0,004348	0,234694	-0,02089	0,173526	0,095353	0,057553	0,12989	0,459602	0,084635	0,130126	0,10557	0,030892	-0,01745	0,137486	0,233591	0,155086	0,367506	0,54853
guanosine_ribonucleotides_ide_novoi_biosynthe:	0,054482	-0,04583	-0,06683	-0,45387	-0,58294	-0,52633	-0,2038	-0,11292	-0,62458	1,734637	2,288386	1,588975	0,69554	0,721369	0,92129	-4,68782	-3,99329	-3,66203	0,368568	0,549851
KEGG_Pantothenate_and_CoA_biosynthesis_Mai	0,141919	-0,20112	0,454024	-0,43969	-2,01833	-2,93049	0,59612	0,428976	0,585322	-0,54998	0,322967	0,171228	0,312416	-0,17509	-0,79025	-0,81521	-1,84507	-1,79398	0,369375	0,550791
Ras_Pathway_RhoA_Pathway	0,087038	-0,1039	-0,20429	0,485378	0,375547	0,073823	-0,21905	-0,22909	-0,68311	0,979464	0,457591	1,257151	-0,06699	-0,00796	-0,27528	-2,00171	-1,62691	-1,35288	0,371486	0,553674
phosphatidylcholine_biosynthesis	0,098883	-0,01299	0,004056	-0,61595	-0,18682	0,110058	-0,2477	-0,14976	-0,50837	-1,26165	0,182244	0,412473	-0,58775	-0,42336	-0,33265	-2,74447	-1,56307	-1,34031	0,371727	0,553721
reactome_Class_A_1_Rhodopsin_like_receptors_	-0,7749	-0,04958	-0,13344	0,433406	-1,32948	-1,00626	0,216069	-0,34983	1,19692	1,860441	-1,54138	-0,0659	0,858395	1,023105	0,883569	3,521936	1,924725	2,41265	0,371873	0,553721
biocarta_growth_hormone_signaling_Main_Pathw	-0,31174	-0,4364	-0,23098	-0,99961	-1,28066	-2,62298	-0,27997	-0,376	-0,55272	1,716266	0,527038	-0,23148	0,778396	0,231411	1,06362	-0,99873	0,195857	0,983975	0,372894	0,554494
reactome_Toxicity_of_botulinum_toxin_type_D_I	-0,07001	0,204879	0,10533	0,392064	0,117744	-0,64227	-0,0435	-0,06565	-0,14503	-0,916	-0,99086	-0,86704	0,773904	0,517845	0,541304	0,464688	1,008819	0,897481	0,372926	0,554494
reactome_Toxicity_of_botulinum_toxin_type_F_E	-0,07001	0,204879	0,10533	0,392064	0,117744	-0,64227	-0,0435	-0,06565	-0,14503	-0,916	-0,99086	-0,86704	0,773904	0,517845	0,541304	0,464688	1,008819	0,897481	0,372926	0,554494
reactome_Hormone_sensitive_lipase_HSL_media	-0,32682	-0,33291	-0,13322	-0,19458	-0,44876	-1,37513	-2,74325	-2,49328	-2,8543	0,590901	-0,78917	0,099721	-0,53644	-0,64801	0,02959	-0,98075	-0,8711	0,184327	0,373667	0,55533
KEGG_Valine_leucine_and_isoleucine_degradatio	0,853746	0,086041	0,485523	-1,05522	-0,5393	-3,1715	3,559683	4,298119	1,189346	2,254783	4,36531	3,452267	0,395002	0,390913	0,468396	-15,6205	-12,3766	-10,7123	0,375152	0,557214
Regulation_of_Cell_Migration_by_Platelet-derive	-0,10906	-0,42335	-0,28577	-1,4228	-1,27626	-1,99549	-2,24757	-1,80401	-2,67922	-0,07065	-0,95648	0,438389	-0,25688	-0,13896	0,333458	-1,70007	-0,41662	-0,05555	0,375292	0,557214
reactome_Synthesis_of_PG_Main_Pathway	0,071508	-0,07449	-0,05941	-0,03804	-0,29903	-1,06878	-0,48146	-0,73311	-0,67362	0,649916	-0,41308	0,332368	0,60332	0,2265	0,475567	0,568077	0,516807	0,629729	0,375776	0,557667
KEGG_Bacterial_invasion_of_epithelial_cells_Mai	-0,50047	-0,8897	-1,27065	-4,93431	-4,45532	-4,45836	-3,49142	-3,15652	-4,2622	-2,12914	-1,96207	0,508228	0,40828	-0,2078	1,71173	-10,8954	-9,23869	-6,68209	0,376051	0,55781
glutamate_dependent_acid_resistance	-0,00238	0,039714	-0,05851	-0,56533	-0,08009	0,169032	-0,36702	-0,30397	-0,25168	0,342721	0,578902	-0,19772	0,114373	-0,01992	0,136747	-0,26121	0,301389	0,077607	0,378999	0,561393
NCI_IL23_mediated_signaling_events_Main_Path	0,05913	-0,08991	-0,08355	-2,22229	-0,4208	-1,24386	-0,70772	-0,44878	-0,71993	-0,44879	-1,29303	1,456747	-0,31048	-0,67694	0,451895	-0,59117	-0,65795	-1,17244	0,379008	0,561393
Role_of_Cholinergic_Receptors_in_Migration_of	0,123229	0,123559	-0,03814	-0,42613	0,356202	-0,12844	-0,062	-0,00319	-0,21161	-0,10325	-0,3276	-0,67856	0,024346	0,198828	-0,10775	0,646847	1,202015	0,779085	0,378818	0,561393
KEGG_Aminoacyl_tRNA_biosynthesis_Main_Path	0,155281	-0,05468	0,100888	-0,02643	-0,16003	-0,7618	0,037278	0,117797	-0,27445	0,708628	-0,21181	0,58101	0,025578	0,034241	0,061002	-1,95651	-1,93753	-1,84915	0,37949	0,56184
NCI_N_cadherin_signaling_events_Pathway_(myc	-0,21493	-0,03502	0,00399	0,044239	0,935038	-0,05972	-1,14444	-1,33751	-1,04583	0,895897	1,374661	0,368202	-0,00527	-0,20118	-0,07645	-1,98805	-1,46139	-1,39442	0,379794	0,562023
NCI_p73_transcription_factor_network_Pathway	-0,18742	-0,18854	-0,42417	-0,78681	-0,58868	0,31699	-1,9434	-1,44957	-2,39852	-0,11415	-1,55219	-0,01034	-0,14634	-0,2133	0,040604	-0,96827	-0,19447	-0,34911	0,380222	0,56239
KEGG_Arrhythmogenic_right_ventricular_cardion	-0,27193	-0,24255	-0,13254	-0,33666	-0,07143	-0,70727	-0,29851	-0,23728	-0,26743	0,918606	1,059231	0,69207	0,233588	0,367603	0,795888	-2,0577	-2,23208	-1,06867	0,380882	0,562718
reactome_MEK_activation_Main_Pathway	-0,0686	-0,15517	-0,17912	-0,20362	-0,22219	-0,61659	-0,70061	-0,54597	-0,95442	-0,98133	0,471339	0,311352	-0,39901	-0,36056	-0,18291	-1,85202	-1,65536	-1,32652	0,380987	0,562718
reactome_RAF_phosphorylates_MEK_Main_Path	-0,0686	-0,15517	-0,17912	-0,20362	-0,22219	-0,61659	-0,70061	-0,54597	-0,95442	-0,98133	0,471339	0,311352	-0,39901	-0,36056	-0,18291	-1,85202	-1,65536	-1,32652	0,380987	0,562718
KEGG_Phototransduction_Main_Pathway	0,09359	0,126099	-0,10776	0,719306	0,829519	0,227935	0,55792	0,426897	0,866791	1,140971	0,651854	0,168411	0,130991	0,446249	0,365373	0,300731	-1,54944	0,93866	0,381333	0,562963
reactome_Synthesis_of_pyrophosphates_in_the_	-0,18883	-0,06698	-0,05184	-0,51797	0,526159	-0,13473	-1,12233	-1,26787	-1,437	1,150576	-0,58927	-0,13943	0,076245	0,526997	0,414183	-0,06997	-0,65325	-0,03768	0,381705	0,563246
KEGG_Vibrio_cholerae_infection_Main_Pathway	-0,36643	-0,16446	-0,22314	-1,14974	-1,55911	-1,16626	-0,25876	0,027875	-0,12463	0,051012	0,100106	0,846698	0,075367	0,134454	0,15	-1,08912	-0,76337	-0,57661	0,38198	0,563385
NCI_Regulation_of_CDC42_activity_Main_Pathwa	-0,05181	-0,42491	-0,47546	-2,33711	-3,31534	-2,60448	-0,79366	-0,23876	-1,39321	-0,57599	0,602443	-0,16929	-0,42438	0,394082	0,542211	-2,67419	-1,60661	0,057882	0,383168	0,564334
NCI_Thromboxane_A2_receptor_signaling_Main_	-0,38762	-0,36974	-0,05317	-1,78066	-2,25649	-1,56179	0,077305	-0,44272	0,464392	-2,32149	-1,00904	-1,20838	-0,91215	-0,91151	-0,15046	-1,98277	-0,76905	0,130862	0,382992	0,564334
reactome_FGFR1c_ligand_binding_and_activatior	-0,24717	-0,28073	-0,06736	-0,62833	-0,53181	-1,02246	-0,7096	-0,84529	-0,24411	-0,11039	-0,4454	0,153064	-0,1361	-0,12733	-0,15344	-0,46126	-0,25919	-0,75839	0,382953	0,564334
NCI_CDC42_signaling_events_Pathway_(regulatio	-0,23807	-0,12068	-0,29413	-0,24507	-0,38039	-0,3032	-0,98531	-0,82503	-0,93904	0,19517	0,1446	-0,30513	0,056113	0,125653	-0,06955	0,167461	0,190852	-0,06755	0,383373	0,56437
ATM_Pathway_S-phase_progression	0,100775	0,057317	0,027774	0,763	0,235135	0,832794	0,266214	0,37472	-0,02276	0,087915	-0,21224	-0,35883	-0,0825	-0,19897	-0,22873	-0,43402	0,321032	0,554954	0,383808	0,564743
KEGG_Dilated_cardiomyopathy_Main_Pathway	-0,00564	-0,26446	0,044368	-0,87873	-0,95245	0,88317	-0,76364	-1,2665	-0,3621	0,338963	-0,02969	-1,7674	0,05754	0,507206	0,165462	2,123521	1,646874	1,679392	0,384172	0,564745
KEGG_Hypertrophic_cardiomyopathy_HCM__Mai	-0,00564	-0,26446	0,044368	-0,87873	-0,95245	0,88317	-0,76364	-1,2665	-0,3621	0,338963	-0,02969	-1,7674	0,05754	0,507206	0,165462	2,123521	1,646874	1,679392	0,384172	0,564745
NCI_a6b1_and_a6b4_Integrin_signaling_Pathway	0,012874	0,035557	0,04616	-0,15647	-0,19008	-0,61081	-0,72137	-0,69203	-0,80881	-0,41724	-0,00643	-0,15529	-0,10923	-0,0237	-0,03207	-1,21964	-0,87878	-0,53216	0,384362	0,564758
L-kynurenine_degradation	-0,11579	0,187798	0,182529	-0,69533	-0,48863	-0,02275	-0,30018	-0,66246	-0,16537	-1,61144	-0,85492	-1,64044	0,010379	-0,95	-0,91029	0,758084	1,515738	0,153311	0,386165	0,567074
NCI_Class_I_PI3K_signaling_events_mediated_by	0,01545	-0,00502	-0,19519	0,070059	-0,47202	-0,03337	-0,45041	-0,38266	-0,56988	0,212016	0,374681	0,097918	-0,15556	-0,25635	-0,40917	-0,4817	-0,62983	-0,16506	0,386302	0,567074
reactome_GTP_hydrolysis_and_joining_of_the_6(	3,347792	1,355295	0,816484	-8,88391	-11,3327	-13,1635	8,22366	9,915061	4,42298	-4,39742	7,461781	4,96995	-2,29631	-1,63653	1,052358	-54,695	-45,003	-38,2095	0,386987	0,567812
biocarta_skeletal_muscle_hypertrophy_is_regula	0,210451	0,159555	0,070731	-0,08929	-0,26879	-0,713	0,764382	0,886838	0,467137	0,243493	-0,0428	0,174823	-0,45254	-0,3861	0,120476	-2,88542	-2,15102	-2,06488	0,387468	0,567982
TSPANs_Influence_on_Integrin-based_Cell_Migra	-0,07261	-0,25771	-0,51288	-1,48814	-4,47459	-1,83456	-7,28826	-6,7699	-8,65574	-2,5969	0,344647	-0,61745	-0,77149	-1,39511	-0,71175	-9,71958	-7,65998	-6,20992	0,387368	0,567982
biocarta_cell_cycle_g2_m_checkpoint_Pathway_(	0,11739	0,007474	0,011975	0,416058	0,624927	0,246951	0,260642	0,479998	-0,05707	0,56299	0,635933	0,43159	-0,19765	-0,20441	-0,22649	-1,37453	-0,94019	-0,72185	0,388484	0,569204
biocarta_role_of_erbb2_in_signal_transduction_a	-0,06213	-0,34175	-0,54939	-2,28448	-1,4225	-2,36308	-0,83132	-0,7616	-1,45537	0,145579	1,508244	1,291373	0,168773	-0,33249	0,437139	-2,7232	-1,86844	-0,6643	0,388697	0,569248
reactome_Pyrimidine_catabolism_Main_Pathway	-0,42781	0,086071	-0,03041	-0,07756	0,809657	-1,22967	-0,95543	-1,05209	-0,44476	0,5113	-0,37311	0,014105	-0,62101	-0,44701	-0,00703	0,715634	0,380962	-0,62889	0,388975	0,569388
Junctional_Cell_Adhesion_in_Endothelium	-0,4014	-0,25479	-0,12888	-0,7305	-1,04375	-2,27604	-0,83043	-1,31737	-0,95165	1,379098	0,260652	-0,13443	1,838391	0,850191	2,343593	-3,27793	-1,60058	-1,36539	0,389635	0,570085
IL-10_Pathway_IL-10_Responsive_Genes_Transcri	-0,12692	-0,35259	-0,33213	-0,12913	-0,30758	-0,46915	1,219225	1,893608	0,893645	2,784676	0,883027	1,866511	0,769412	0,502844	1,112876	-4,13188	-2,29079	-2,32895	0,390965	0,571496
IL-10_Pathway_Inflammatory_Cytokine_Genes_E:	-0,12692	-0,35259	-0,33213	-0,12913	-0,30758	-0,46915	1,219225	1,893608	0,893645	2,784676	0,883027	1,866511	0,769412	0,502844	1,112876	-4,13188	-2,29079	-2,32895	0,390965	0,571496
reactome_Synthesis_of_5_eicosatetraenoic_acids	-0,10482	-0,06624	0,015334	0,336759	-0,80896	0,487633	-0,29925	-0,39287	0,032762	0,393108	0,803672	0,724844	0,096108	-0,26291	0,035329	0,0677	0,322897	-0,44618	0,391253	0,571648
reactome_eNOS_activation_Main_Pathway	0,124318	-0,07619	-0,01586	-0,90709	-0,44029	-0,35692	-1,05923	-1,0589	-1,4723	0,157357	0,176904	0,275874	-0,50263	-0,24692	-0,64497	-1,38962	-1,20687	-0,80165	0,39155	0,571814
BRCA1_Pathway_Cell_Cycle_Arrest_DNA_Repair_	0,108332	0,021866	-0,032	0,671595	0,317872	0,006742	0,33726	0,622465	-0,03104	1,027442	0,362946	0,271397	0,02627	-0,00524	0,063816	-0,92014	-0,26329	-0,74617	0,391823	0,571944
reactome_Downregulation_of_ERBB2_ERBB3_sig	0,26524	-0,12807	-0,05724	-0,33932	0,483981	-0,70329	-0,1382	-0,07642	-0,78841	0,491525	1,429025	0,963202	-0,0531	0,077172	0,010776	-3,98899	-2,893	-2,05408	0,393305	0,573839
Akt_Signaling_Pathway_Acetylation_of_proteins	-0,02743	-0,18176	0,243291	-1,16131	0,370528	-1,27001	0,061919	0,02362	0,138129	0,379252	1,338398	-0,07718	0,791479	0,614017	0,392781	-0,18282	0,552932	0,955832	0,393952	0,574245
KEGG_Non_small_cell_lung_cancer_Main_Pathw	-0,55018	-0,73063	-0,80578	-0,97047	0,178006	-1,80212	-1,08854	-0,49092	-2,03753	-0,09708	0,322567	1,187465	0,186088	-0,34071	0,974932	-4,79086	-1,8279	-0,70851	0,393863	0,574245
dermatan_sulfate_degradation_metazoa	0,049434	-2,07E-05	0,048733	-1,48106	-0,84799	-0,94445	-0,53658	-0,44702	-0,52181	1,631018	-0,3023	0,599696	-0,14254	-0,212	0,192134	-1,28543	-1,09456	-1,50358	0,395251	0,575355
NCI_PAR1_mediated_thrombin_signaling_events_	-0,09624	-0,37217	-0,17185	0,678671	0,889144	-0,38883	-0,91155	-0,70001	-0,81286	-0,16568	-1,2855	-1,64507	-0,12317	-0,10736	0,426716	0,503824	0,780169	1,473308	0,394928	0,575355
reactome_Laminin_interactions_Main_Pathway	-0,16782	-0,171	-0,11618	-0,67788	-1,84659	-1,09893	-4,40258	-4,64322	-4,75404	2,3122	1,47713	0,218339	-0,42321	-0,23054	-0,64313	-1,96635	-2,22986	-2,11989	0,395266	0,575355
reactome_Chondroitin_sulfate_biosynthesis_Mai	-0,17896	-0,39789	-0,28844	-0,20271	-0,22904	-0,393	0,030101	-0,25665	0,120877	-0,39261	-1,66069	0,360264	0,105319	0,576072	1,691165	3,381763	4,214223	3,50667	0,395787	0,575654
reactome_Lysine_catabolism_Main_Pathway	0,175685	0,057265	0,079488	-0,61755	-0,91505	-0,95368	1,030297	1,280331	0,591674	-0,04771	1,14968	1,109591	-0,2602	0,123946	-0,2155	-1,821	-1,33497	-0,64888	0,395843	0,575654
KEGG_Phenylalanine_tyrosine_and_tryptophan_t	-0,07712	0,046873	0,062889	0,192673	-0,63197	-0,28188	0,254803	0,310668	0,524261	0,322431	0,136537	1,042131	0,005649	0,254439	0,0476	-0,0388	-0,24427	-0,35682	0,397479	0,576886
reactome_binding_of_TCF_LEF_CTNNB1_to_targ	-0,17289	-0,1844	-0,127	-0,27011	-0,34905	-0,74234	-0,33521	-0,20277	-0,35703	0,649005	0,931656	0,847155	0,136458	0,373346	0,783955	-2,16146	-2,35998	-1,23581	0,397368	0,576886
reactome_Formation_of_the_ternary_complex_a	1,352488	0,684484	0,291812	-4,75616	-4,83099	-5,49404	3,981531	4,831749	2,387973	-2,53652	3,400178	2,678264	-1,14494	-1,74469	0,036927	-24,9793	-21,397	-17,8987	0,397043	0,576886
reactome_Release_of_eIF4E_Main_Pathway	0,085117	0,109203	0,022044	-0,26623	0,202968	-0,47943	0,345854	0,387841	0,057356	0,025845	-0,52043	-0,67478	0,479987	-0,13409	0,204994	-0,60619	-0,19614	0,286827	0,397616	0,576886
reactome_Scavenging_by_Class_A_Receptors_Ma	-0,20647	0,016766	0,230752	-0,16279	0,601149	0,409396	-0,20621	-0,49239	0,288508	0,567284	-0,16684	0,627199	0,058045	0,244281	-0,14736	1,103916	0,618986	0,21479	0,397343	0,576886
biocarta_insulin_signaling_Main_Pathway	-0,0023	-0,25644	-0,21634	-0,24811	-0,2355	-0,97327	-1,43196	-1,50636	-1,72861	1,026325	0,449783	-0,396	0,416118	0,264229	0,461348	-2,27608	-1,00835	-0,81353	0,398605	0,577514
KEGG_Allograft_rejection_Main_Pathway	-0,00014	0,06814	-0,05993	-0,12348	0,106456	0,059927	0,021723	0,089755	0,107456	-0,04193	-0,27879	0,067829	-0,16284	0,076734	-0,05821	-0,47756	-0,27573	0,183761	0,398597	0,577514
reactome_Ribosomal_scanning_and_start_codon	1,693714	0,990572	0,593714	-4,30094	-4,93888	-5,68948	3,648939	4,653776	1,610179	-1,69892	4,302291	3,006627	-0,886	-1,85892	0,228382	-27,754	-23,0958	-18,8006	0,398336	0,577514
KEGG_Sulfur_relay_system_Main_Pathway	-0,0825	-0,15511	-0,12838	-0,21264	0,680441	0,523628	0,403437	0,38671	0,192887	2,349812	0,496907	0,397337	0,27556	0,129042	0,044721	-1,19858	-1,5335	-1,28667	0,400718	0,580305
reactome_AKT_phosphorylates_targets_in_the_n	-0,08948	-0,18509	-0,01597	0,239168	0,457641	0,20137	0,284331	0,330652	0,137567	-0,5668	0,109258	-0,13356	0,231981	-0,14737	0,094021	0,193385	0,547282	0,60792	0,40096	0,580387
KEGG_Osteoclast_differentiation_Main_Pathway	-2,33664	-0,48855	-0,64763	0,825069	-1,18444	2,050523	-2,44489	-3,95254	-0,60984	-2,05592	-0,60598	0,471163	-0,09442	-0,6704	1,216978	6,073547	6,214303	7,466999	0,402865	0,580887
NCI_Arf6_downstream_Pathway_(liver_developm	0,006941	-0,05848	-0,0376	-0,07168	-0,39179	0,073645	-0,03055	-0,04361	-0,05806	-0,16693	-0,25344	-0,28603	0,151555	0,077987	0,016693	0,027073	-0,03148	-0,11837	0,402985	0,580887
NCI_Arf6_downstream_Pathway_(myoblast_fusic	0,006941	-0,05848	-0,0376	-0,07168	-0,39179	0,073645	-0,03055	-0,04361	-0,05806	-0,16693	-0,25344	-0,28603	0,151555	0,077987	0,016693	0,027073	-0,03148	-0,11837	0,402985	0,580887
NCI_Arf6_downstream_Pathway_(regulation_of_	0,006941	-0,05848	-0,0376	-0,07168	-0,39179	0,073645	-0,03055	-0,04361	-0,05806	-0,16693	-0,25344	-0,28603	0,151555	0,077987	0,016693	0,027073	-0,03148	-0,11837	0,402985	0,580887
NCI_Arf6_downstream_Pathway_(substrate_adhe	0,006941	-0,05848	-0,0376	-0,07168	-0,39179	0,073645	-0,03055	-0,04361	-0,05806	-0,16693	-0,25344	-0,28603	0,151555	0,077987	0,016693	0,027073	-0,03148	-0,11837	0,402985	0,580887
NCI_Arf6_trafficking_events_Pathway_(clathrin_i	0,013882	-0,11696	-0,0752	-0,14336	-0,78358	0,14729	-0,0611	-0,08722	-0,11611	-0,33387	-0,50688	-0,57205	0,303111	0,155974	0,033386	0,054147	-0,06295	-0,23674	0,402985	0,580885
NCI_Arf6_trafficking_events_Pathway_(positive_r	0,013882	-0,11696	-0,0752	-0,14336	-0,78358	0,14729	-0,0611	-0,08722	-0,11611	-0,33387	-0,50688	-0,57205	0,303111	0,155974	0,033386	0,054147	-0,06295	-0,23674	0,402985	0,580887
NCI_Arf6_trafficking_events_Pathway_(receptor_	0,013882	-0,11696	-0,0752	-0,14336	-0,78358	0,14729	-0,0611	-0,08722	-0,11611	-0,33387	-0,50688	-0,57205	0,303111	0,155974	0,033386	0,054147	-0,06295	-0,23674	0,402985	0,580887
reactome_Initiation_of_Nuclear_Envelope_Refor	-0,02834	0,003157	-0,00038	-1,0898	-0,36716	-0,16871	0,082654	0,380365	-0,15116	-0,35163	0,925366	-1,05421	-0,05044	-0,49416	0,040853	-2,42011	-1,90089	-1,48102	0,402291	0,580887
reactome_The_AIM2_inflammasome_Main_Path	-0,04249	-0,04058	-0,21416	0,057578	-0,95042	-0,88918	0,729455	0,694489	0,618259	-0,11755	-0,18024	-0,62912	-0,32497	-0,01399	0,463581	0,679842	0,075343	0,014484	0,403397	0,581212
KEGG_VEGF_signaling_Main_Pathway	-0,30689	-0,61052	-0,66077	-1,44199	-1,90929	-2,24365	-1,44233	-1,16027	-1,27909	-0,89575	0,837894	1,026645	-0,59618	-0,82956	0,371559	-3,64566	-1,00278	-0,08634	0,403778	0,581493
biocarta_endocytotic_role_of_ndk_phosphins_an	0,129653	-0,12821	0,161837	1,439735	0,665773	1,139717	-2,12555	-1,83863	-2,36119	-0,19945	1,150975	-0,28964	-0,40935	-0,71001	-0,49124	-0,84774	-0,30615	-0,56997	0,405235	0,582819
glucocorticoid_biosynthesis	-0,19976	-0,3001	-0,01891	0,492777	-0,09597	0,198431	0,123972	-0,15845	0,478501	-0,00549	-0,39181	-0,32353	-0,14029	-0,30454	-0,12051	1,384252	1,194216	0,708986	0,404921	0,582819
NCI_CXCR4_mediated_signaling_events_Pathway	-0,26865	-0,43559	-0,16965	-1,20699	0,261164	-1,09788	-0,68615	-0,94174	-0,67122	-0,94903	-1,0902	-0,92274	-0,36039	-0,15607	0,023678	0,656986	1,294682	1,073914	0,40526	0,582819
biocarta_endocytotic_role_of_ndk_phosphins_an	-0,04698	-0,21323	0,040596	0,325097	0,215246	0,526169	-2,70376	-2,51095	-2,64068	0,515669	1,220829	0,18552	-0,1211	-0,43579	-0,39557	0,137705	0,17622	-0,15554	0,406443	0,583981
Integrin-based_Cell_Migration_and_Adhesion	-0,8983	-0,47769	-0,37787	-2,63129	-2,86526	-1,85587	-2,31201	-3,15	-1,43134	0,0875	-2,55899	0,443307	-0,5847	-0,00753	-0,31336	-6,3389	-5,21163	-4,76746	0,406351	0,583981
MAPK_Signaling_Pathway_Cell_Survival	0,005097	-0,09786	-0,08708	0,040612	0,081802	0,293394	-0,79421	-0,85734	-0,95915	-0,76427	0,00214	-0,26319	0,062509	-0,03054	0,068489	0,205455	0,300626	0,479718	0,406717	0,584105
D-imyoi-inositol_145-trisphosphate_degradation	0,3356	-0,15936	-0,01612	-0,2592	-0,50062	-0,12522	-2,18341	-1,96943	-3,11596	-0,17074	-0,09156	-0,30228	-0,55121	-0,24711	-0,42423	-1,87196	-2,2549	-1,04001	0,407384	0,584524
reactome_Keratan_sulfate_degradation_Main_Pa	-0,37224	0,316035	0,180626	-0,17258	0,118318	0,131705	-0,38715	-0,7752	-0,18712	1,424129	-0,58383	-0,71214	-0,94408	-0,59149	-0,85565	-1,38955	-0,0763	0,281108	0,40729	0,584524
reactome_Signaling_by_Robo_receptor_Main_Pa	-0,2443	-0,18031	-0,20013	-0,38778	-0,29509	-0,25145	-2,30031	-2,20298	-2,18139	-0,59724	0,505044	0,230931	0,01765	0,500667	0,399389	-0,7532	-0,16172	-0,167	0,407586	0,584545
reactome_Molybdenum_cofactor_biosynthesis_N	-0,11553	-0,04393	-0,1888	0,316701	-0,29059	-0,02415	0,374994	0,448331	0,20991	0,857164	0,143698	0,543338	0,068988	-0,15179	0,224006	-1,33369	-0,73203	-0,83847	0,407872	0,584685
reactome_Activation_of_caspases_through_apop	0,148032	0,137817	0,11567	-0,03018	-0,49262	0,132338	-0,16779	0,008411	-0,45575	0,72488	-0,41417	-0,5926	-0,10823	-0,33056	-0,37725	-1,52364	-1,33193	-1,24745	0,409456	0,586686
KEGG_Platelet_activation_Main_Pathway	0,164433	0,183224	-0,04599	-0,04504	2,01693	-0,68364	4,290826	3,196434	5,475651	2,375249	1,180762	2,705133	-1,17255	-2,18556	-1,3424	-0,14898	-2,38987	-2,62154	0,410539	0,587967
KEGG_Gap_junction_Main_Pathway	-0,55523	0,293098	0,102977	1,165381	1,952321	1,206836	0,958843	0,426231	2,130191	2,847539	0,012845	0,943595	1,617529	2,585652	1,368987	9,04094	6,179746	4,854708	0,411315	0,588808
ILK_Signaling_Pathway_Apoptosis	-0,04643	-0,05563	-0,1359	0,277946	0,766395	0,11797	-0,12296	-0,35546	-0,50546	-0,24529	0,524921	1,188294	0,221785	0,084172	0,200825	-0,55742	0,168272	0,164916	0,412445	0,589976
reactome_FGFR4_ligand_binding_and_activation	-0,52595	-0,30497	-0,02531	-0,38718	-0,12373	-0,44961	-0,75936	-1,20818	0,088171	-0,04688	-0,83174	-0,41194	-0,19038	0,023049	-0,3209	0,597707	0,625539	0,122798	0,41251	0,589976
reactome_Post_transcriptional_silencing_by_sma	-0,00911	-0,05324	-0,07027	0,018189	-0,45157	-0,41322	-0,39834	-0,4009	-0,57064	-0,31419	-0,2448	-0,42304	-0,07948	0,022895	-0,00218	0,181981	0,113705	0,421729	0,41273	0,59002
Akt_Signaling_Pathway_Apoptosis	-0,03368	-0,16804	0,211271	-1,14987	0,473984	-1,19594	0,203703	0,069108	0,306978	-0,12982	1,613419	0,252805	0,716692	0,642843	0,365176	-0,31316	0,728132	0,893811	0,412996	0,590129
NCI_LKB1_signaling_events_Pathway_(tubulin_co	-0,02032	-0,11296	-0,16736	-0,14548	0,778732	-0,03459	-0,11694	-0,15653	-0,1727	0,175874	-0,42727	1,216163	-0,00303	0,326163	0,137781	0,025276	-0,58796	-0,18201	0,413515	0,590385
NCI_Osteopontin_mediated_events_Pathway_(ru	0,077511	-0,01427	-0,08996	-0,27804	-0,91119	-0,44447	-0,05392	0,134593	-0,14425	-0,16019	-0,23914	-0,63425	-0,27894	-0,32854	-0,18624	-1,64251	-1,54062	-1,36553	0,413554	0,590385
biocarta_pten_dependent_cell_cycle_arrest_and	0,048949	-0,03332	-0,13127	-0,33789	-1,0108	-0,43135	-0,08443	-0,32705	-0,55299	-0,00144	0,112282	0,650877	0,445518	0,474112	0,63143	-1,87996	-1,22707	-0,71158	0,414739	0,591265
GDP-mannose_biosynthesis	0,114795	0,097613	0,091517	0,185956	0,203507	0,248257	-0,67657	-0,48235	-0,64818	-0,76905	-0,45289	-0,20588	-0,42653	-0,25435	-0,12545	0,083093	-0,29063	-0,08948	0,414711	0,591265
melatonin_degradation_II	0,046668	-0,10228	0,071533	-0,30799	-0,07301	-0,10052	-0,22249	-0,26725	-0,2513	-0,64133	-0,7107	-0,38168	0,047987	0,108886	0,286357	0,198259	0,204053	0,152241	0,414556	0,591265
reactome_PP2A_mediated_dephosphorylation_o	0,10013	0,032478	-0,02411	-0,25792	-0,80062	-0,72504	-0,02294	-0,03356	-0,05157	1,02631	1,26598	1,013224	0,23257	0,350919	0,151279	-0,55689	-1,24184	-0,55	0,414969	0,591321
NCI_Posttranslational_regulation_of_adherens_ju	-0,28335	0,021806	0,011991	-0,80834	0,285533	-1,84464	-7,34625	-7,27354	-7,34272	-1,67069	0,712872	1,726024	-0,38904	-0,10027	-0,3432	-4,20496	-3,61482	-3,33346	0,415559	0,591892
adenosine_ribonucleotides_ide_novoi_biosynthe	-0,18293	-0,23944	-0,32435	0,501459	-0,02973	-0,2735	0,224557	0,208415	0,181599	0,423421	0,166136	-0,09439	-0,51424	-0,46616	-0,09776	-0,875	-1,37487	-0,60324	0,416267	0,592357
CD40_Pathway_Cell_Survival	0,064229	0,085999	0,033969	0,270774	0,230146	0,076514	-0,20915	-0,17567	-0,16896	0,662208	0,502484	0,40801	0,295389	0,066174	0,249027	-0,31746	-0,45033	-0,22797	0,41611	0,592357
NCI_ErbB1_downstream_signaling_Pathway_(cell	0,301512	0,011217	-0,04921	-0,86807	-1,20488	-1,81199	0,753989	1,065725	0,305877	-1,49018	0,073096	-0,88655	0,091155	0,083914	0,040147	-0,34462	-0,29454	0,100068	0,416657	0,592643
biocarta_internal_ribosome_entry_Main_Pathwa	0,284305	0,233592	0,003431	-0,22096	-0,78849	-0,60029	0,319276	0,899601	-0,39898	1,547893	-0,68549	-0,48057	-0,34226	-0,97976	-0,43551	-5,02311	-3,53824	-2,99114	0,41738	0,592842
biocarta_internal_ribosome_entry_Pathway_(trar	0,284305	0,233592	0,003431	-0,22096	-0,78849	-0,60029	0,319276	0,899601	-0,39898	1,547893	-0,68549	-0,48057	-0,34226	-0,97976	-0,43551	-5,02311	-3,53824	-2,99114	0,41738	0,592842
IGF1R_Signaling_Pathway_Apoptosis	-0,21618	-0,23766	-0,44563	1,110062	-0,27748	0,096788	-0,61221	-0,62801	-0,87598	-0,81471	0,67958	-0,23573	0,163371	-0,19397	0,319096	-0,28418	0,347726	0,93716	0,417081	0,592842
NCI_Role_of_Calcineurin_dependent_NFAT_signa	-0,0604	-0,12954	-0,09886	-0,00383	0,11339	0,374027	0,406995	0,351497	0,320803	-0,30327	-0,46734	-0,53973	0,311085	0,260712	-0,00575	0,73644	0,733418	0,38458	0,417749	0,592842
reactome_Acetylcholine_regulates_insulin_secret	0,072974	-0,14368	-0,21544	-0,21888	0,909397	0,014125	-0,98997	-0,95509	-1,10281	0,159639	-0,97312	-0,25752	-0,13259	-0,19449	-0,03526	-0,13296	-0,12179	0,470661	0,417655	0,592842
asparagine_biosynthesis	-0,00959	0,033694	-0,05722	-0,5152	-0,3273	-0,02737	1,07E-05	0,015363	0,051409	-0,26867	0,144102	0,224753	0,069904	0,048177	0,158676	-0,72813	-0,88751	-0,64982	0,418846	0,593323
CREB_Pathway	-4,58324	-3,22515	-2,87345	-1,93855	1,648762	-4,40608	-15,8602	-17,1017	-13,7504	-5,64213	-4,4427	-8,66407	5,42395	2,317148	7,012085	6,676155	4,936513	11,47608	0,418904	0,593323
GNAQ_Signaling_Events	0,192206	-0,06435	-0,13386	0,50292	0,798258	0,172743	-0,38652	-0,31999	-0,80119	0,109742	0,165767	-0,12937	-0,42424	-0,676	-0,44497	-1,12174	-1,06923	-0,28661	0,419138	0,593323
KEGG_Tight_junction_Main_Pathway	-1,59897	-0,83564	-0,97412	-2,75184	-3,88719	-2,83154	-1,87397	-3,82472	0,13888	-0,77357	-3,10271	-6,0769	0,719945	1,381723	1,260097	-1,39582	-0,69021	-2,38674	0,418687	0,593323
MAPK_Signaling_Pathway_Gene_Expression_Cell	0,389335	-0,36227	-0,25294	-0,30924	-0,56034	-0,76062	-6,17058	-5,49098	-6,44441	1,410245	3,193383	0,760671	0,807328	0,681877	0,881475	-3,30314	-3,70156	-3,59276	0,418872	0,593323
mTOR_Pathway_Ribosome_Biogenesis	0,061457	-0,04235	-0,03321	0,110927	-0,27729	-0,63348	0,597204	0,636228	0,413384	0,141331	0,590319	0,276373	0,115318	0,184896	0,350773	-1,28468	-1,04436	-0,86335	0,419299	0,593323
reactome_L13a_mediated_translational_silencing	3,214971	1,245557	0,802403	-9,46972	-12,0115	-13,5534	8,831133	10,51107	5,160193	-4,2646	7,832923	5,192348	-2,37654	-2,01705	0,989479	-54,0644	-44,3426	-38,0193	0,419421	0,593323
ketogenesis	0,108255	-0,02057	0,024602	-0,1283	-0,64052	-0,54511	0,452644	0,453575	0,35139	-0,63261	-0,2111	0,543042	0,350615	0,064831	0,430333	-0,7047	-0,90473	-0,74583	0,419679	0,593419
biocarta_hiv_1_nef_negative_effector_of_fas_an	0,207841	-0,05677	0,095531	-0,23746	0,37749	-0,11925	-1,78317	-1,39644	-1,93578	-0,1029	-1,34002	-0,19704	-0,34264	-0,53154	-0,78232	-1,47477	-1,24572	-0,3911	0,420168	0,59384
biocarta_basic_mechanism_of_action_of_ppara_	-0,26897	0,017994	-0,17118	-0,70734	-0,10993	-0,04428	-0,54288	-0,51263	-0,32274	-0,27718	-0,13883	0,324519	0,313053	0,1053	0,396956	0,624967	0,665681	0,773039	0,420603	0,594185
EGF_Pathway_Cell_Survival	-0,31278	-0,24725	-0,355	0,250015	0,54941	-0,40742	0,765936	0,88119	0,786211	0,999005	1,125737	1,167404	0,355103	0,01344	0,391513	-1,81376	-0,93767	-0,68394	0,421058	0,594559
KEGG_Salmonella_infection_Main_Pathway	-0,10757	-0,92652	-0,90738	-2,06412	-2,50363	-3,51756	-4,21331	-3,88465	-5,22109	-3,07183	0,182411	1,410143	0,053147	-1,12436	-0,06321	-7,57964	-6,50669	-5,5246	0,421672	0,594939
reactome_Tachykinin_receptors_bind_tachykinin	0,023988	0,022746	0,110541	0,078663	-0,12233	0,582073	-0,12421	0,007701	0,138348	0,13236	-0,77987	-0,00344	-0,21128	-0,27225	0,003473	0,716278	0,581529	0,808093	0,421709	0,594939
KEGG_Autoimmune_thyroid_disease_Main_Pathw	-0,24151	-0,04636	-0,10347	-0,64551	0,110981	0,606512	0,206991	0,343059	0,394058	1,453264	-0,70412	0,013955	-0,17733	0,02527	0,048657	0,457331	0,746296	0,371121	0,422767	0,595662
KEGG_Graft_versus_host_disease_Main_Pathway	-0,24151	-0,04636	-0,10347	-0,64551	0,110981	0,606512	0,206991	0,343059	0,394058	1,453264	-0,70412	0,013955	-0,17733	0,02527	0,048657	0,457331	0,746296	0,371121	0,422767	0,595662

reactome_Formation_of_a_pool_of_free_40S_su	2,894203	0,926044	0,514561	-9,57979	-11,594	-13,1742	8,520555	9,975784	5,350214	-4,62104	7,000686	4,74415	-2,26153	-1,351	0,997631	-49,2949	-41,3316	-35,7247	0,422796	0,595662
NCI_Signaling_events_mediated_by_PRL_Pathwa	-0,07318	0,013676	0,009474	-0,15592	0,008002	-0,24304	-0,66598	-0,60858	-0,57561	-0,10713	-0,1025	0,146302	-0,03172	-0,04391	0,065734	-0,02824	0,109627	-0,14251	0,423482	0,596359
Gamma-Aminobutyric_Acid_A_Receptor_Pathwa	0,062985	-0,13767	0,160171	-0,62639	-1,34716	-1,72442	-2,89695	-2,97264	-3,31216	-1,40796	1,994013	1,39065	0,712362	0,34746	0,946794	-0,71485	-0,12647	0,505283	0,424516	0,597485
Rapoport-Luebering_glycolytic_shunt	0,031439	-0,03032	0,033455	-0,15187	-0,06393	-0,09253	0,007848	0,145818	0,030979	0,468105	0,13722	0,222891	0,086041	0,052922	-0,05588	-0,94879	-0,7654	-0,8117	0,424665	0,597485
reactome_Removal_of_aminoterminal_propeptid	-0,1545	-0,01255	0,091059	-1,02933	-0,27483	0,821912	-0,1927	-0,61845	0,347173	-1,00566	0,093709	0,820425	0,233966	0,009325	0,342763	1,434079	0,993811	0,514811	0,424903	0,597549
adenine_and_adenosine_salvage_I	0,013768	-0,0544	0,007219	-0,19509	-0,06012	0,015236	-0,14044	-0,14419	-0,07971	-0,57458	-0,14041	-0,12418	0,029943	0,114372	0,104558	0,035553	0,24203	-0,03666	0,42571	0,598414
biocarta_extrinsic_prothrombin_activation_Main_	-0,20548	-0,09602	0,009637	-0,80439	-0,76119	-0,30151	0,133529	-0,18222	0,569219	-1,48255	-1,94125	-0,23066	0,70359	0,124109	-0,47833	2,93121	1,44779	1,443906	0,426269	0,59866
KEGG_Alanine_aspartate_and_glutamate_metab	0,055362	-0,36186	-0,10223	-0,97869	-2,5658	-1,28288	-1,22191	-0,79836	-2,13302	0,346978	1,186312	-0,20895	0,85019	0,290053	0,846809	-6,4471	-3,34874	-2,62921	0,426247	0,59866
Protein_kinase_A_Pathway	0,065946	0,005426	0,033105	0,395624	0,223113	-0,23546	-0,03879	-0,05436	-0,18535	0,316468	0,153323	0,188351	0,961203	0,736941	0,593843	0,941142	0,418238	0,404374	0,42704	0,599473
reactome_Glucagon_type_ligand_receptors_Main	-0,17485	-0,01966	-0,12365	1,577495	1,083278	0,823993	0,216016	-0,25982	0,475757	-1,2534	1,221715	0,333437	-0,37797	-0,46108	-0,17557	0,059787	-0,14551	0,681627	0,42746	0,599792
NCI_TNF_receptor_signaling_pathway__Main_Pa	-0,04019	-0,43424	-0,46554	-2,55336	-0,778	-3,47876	-5,99414	-5,53278	-6,29038	-1,63201	-2,36781	0,141301	0,292967	0,233979	-0,166	-4,12764	-4,79574	-2,4419	0,428018	0,600304
KEGG_Leukocyte_transendothelial_migration_Ma	-0,52342	-0,55524	-0,87074	1,63585	-2,52625	-1,98592	-4,62971	-5,20481	-4,03764	2,433498	1,832804	1,506039	0,830182	0,396355	0,656501	-4,95002	-4,37111	-4,70441	0,428461	0,600385
reactome_Signaling_by_BMP_Main_Pathway	0,029613	0,208958	-0,08182	0,058658	-0,77864	-1,44152	1,349183	0,896568	0,565042	-1,13173	-0,11287	-0,09034	-0,18726	-0,68582	-0,25007	-1,25102	-0,65501	0,306231	0,428345	0,600385
reactome_SHC_activation_Main_Pathway	0,042026	-0,04586	-0,07837	-0,1854	-0,13498	-0,56436	-0,64936	-0,72772	-0,7379	0,66457	0,610728	0,01062	0,314542	-0,03207	0,044618	-0,57998	-0,05133	-0,13235	0,429122	0,601041
biocarta_cell_cycle_g2_m_checkpoint_Main_Path	0,090888	0,183983	0,077684	1,101339	0,47085	0,276669	1,221106	1,435605	0,862766	3,325901	2,274528	1,320733	-0,44234	-0,5177	-0,15851	-1,00529	-0,32514	-0,82393	0,429759	0,601662
4-hydroxyproline_degradation	-0,12166	-0,04237	-0,06605	-0,36893	-0,12519	-0,32214	0,038055	-0,05446	0,026638	-0,36626	-0,28464	0,328637	0,326725	0,392629	-0,00673	0,418295	0,927469	0,455669	0,430912	0,603006
Akt_Signaling_Pathway_JNK_mediated_apoptosis	0,052649	-0,09002	0,384047	-1,43596	0,561956	-1,33559	0,752977	0,64739	0,96909	0,575512	1,547093	-0,59003	0,556903	0,47582	0,230175	-0,68981	0,419168	0,32663	0,43137	0,603375
reactome_Condensation_of_Prophase_Chromoso	0,431296	-0,09321	-0,54208	0,177898	-0,73661	-2,1289	7,055882	7,989708	5,713197	0,089091	-0,16741	-1,22389	-1,53333	-2,12565	-0,30552	-7,68986	-4,28187	-3,86246	0,432093	0,603863
reactome_Translation_initiation_complex_format	1,53471	0,869988	0,51438	-4,73744	-5,48579	-5,98922	4,068946	5,08046	2,086378	-2,09562	4,462772	3,005687	-0,97059	-2,18585	0,123058	-27,5481	-22,7101	-18,7244	0,432106	0,603863
Cellular_Apoptosis_Pathway	2,790215	1,47545	1,141054	6,091493	2,545134	6,550772	11,80354	12,86827	10,38309	-0,21791	4,667837	6,83155	-4,93777	-3,8809	-6,19119	-0,66312	2,555786	0,36909	0,432788	0,604545
reactome_N_glycan_trimming_in_the_ER_and_C	0,01264	-0,055	-0,0503	0,075031	-0,15952	0,031267	0,127582	0,156455	-0,09721	-0,09446	0,187632	0,334317	-0,27777	-0,24135	-0,12757	-0,87874	-0,82133	-0,96328	0,433306	0,604726
reactome_Toxicity_of_botulinum_toxin_type_G_	-0,02474	-0,06765	-0,12107	0,07897	-0,17545	-0,60256	-1,47746	-1,36562	-1,67444	-1,10998	-2,10375	-1,03882	0,23437	0,012502	-0,07037	0,337896	0,700496	0,297319	0,433161	0,604726
reactome_Vitamin_B5_pantothenate_metabolism	0,031394	0,017433	-0,08095	0,820334	-0,2801	0,630865	-0,07527	-0,13272	-0,35068	0,801832	0,423073	0,046925	-0,05958	-0,3998	-0,26556	-0,75279	-0,96718	-1,01673	0,433522	0,604756
IL-6_Pathway	-0,05109	0,45252	0,080369	-2,00434	-2,35272	-1,16929	1,071678	2,086743	0,460005	2,178661	0,344351	-0,45378	-0,23181	1,494251	1,543319	-7,41577	-4,68575	-3,51267	0,434375	0,605674
NCI_IL6_mediated_signaling_events_Pathway_(ce	0,022008	-0,01544	0,044021	-0,34275	-0,04449	-0,1444	-0,31602	-0,29005	-0,33838	-0,54486	-0,11284	-0,3704	0,100872	0,214462	0,121083	0,781724	0,63788	0,723754	0,436473	0,607784
NCI_IL6_mediated_signaling_events_Pathway_(ce	0,022008	-0,01544	0,044021	-0,34275	-0,04449	-0,1444	-0,31602	-0,29005	-0,33838	-0,54486	-0,11284	-0,3704	0,100872	0,214462	0,121083	0,781724	0,63788	0,723754	0,436473	0,607784
NCI_IL6_mediated_signaling_events_Pathway_(m	0,022008	-0,01544	0,044021	-0,34275	-0,04449	-0,1444	-0,31602	-0,29005	-0,33838	-0,54486	-0,11284	-0,3704	0,100872	0,214462	0,121083	0,781724	0,63788	0,723754	0,436473	0,607784
glutamine_biosynthesis	0,035533	0,023648	0,021375	-0,22016	0,186501	0,143478	0,133716	0,169636	0,082902	-0,09718	-0,00039	-0,08251	-0,15102	-0,12172	0,112147	-0,31218	-0,13832	-0,12145	0,436766	0,60792
reactome_Acyl_chain_remodeling_of_CL_Main_P	-0,02315	-0,07764	-0,21143	-1,11728	-1,07722	-1,16111	1,252575	1,46816	0,909999	-0,76226	-0,28642	0,060291	-0,24058	0,227236	-0,10941	-2,50008	-2,57889	-2,07089	0,437271	0,608079
reactome_TGF_beta_receptor_signaling_in_EMT_	-0,07011	-0,17598	-0,19783	-0,70288	-0,09847	-0,92001	-0,77325	-0,76935	-0,94502	0,298494	0,378606	0,969467	0,360088	0,30072	0,217885	-2,26173	-2,00673	-1,34298	0,437262	0,608079
NCI_Arf6_downstream_Pathway_(regulation_of_	-0,01033	-0,06193	-0,05218	0,003801	-0,71163	-0,95744	-0,1247	-0,03206	-0,236	0,549757	-0,75466	-0,14834	0,553332	0,561448	0,323315	-0,75852	-0,68898	-0,66458	0,43861	0,609398
NCI_Arf6_downstream_Pathway_(ruffle_organiza	-0,01033	-0,06193	-0,05218	0,003801	-0,71163	-0,95744	-0,1247	-0,03206	-0,236	0,549757	-0,75466	-0,14834	0,553332	0,561448	0,323315	-0,75852	-0,68898	-0,66458	0,43861	0,609398
Akt_Signaling_Pathway_Cell_Survival	-0,00731	0,093773	0,347331	-0,81537	-0,19414	-1,63753	-1,55115	-1,68453	-1,82829	-0,48541	1,32208	-0,88987	0,766063	0,760728	0,47662	-3,20386	-1,71438	-0,63536	0,440631	0,609926
KEGG_Lysine_biosynthesis_Main_Pathway	0,08224	0,002274	0,024672	-0,25237	-0,62043	-0,69827	0,170769	0,238461	-0,06342	-0,01683	-0,05528	-0,54514	0,249351	0,409397	0,30178	-1,02313	-0,77782	-0,35106	0,44035	0,609926
KEGG_TNF_signaling_Main_Pathway	0,249774	-0,43312	-0,56915	-2,18794	0,122807	-1,89176	-1,45044	-0,80076	-2,73549	-0,07858	-0,60076	1,645057	1,098196	1,427131	1,774061	-3,44244	-2,35937	-0,09997	0,440624	0,609926
NCI_DNA_PK_pathway_in_nonhomologous_end_	0,527227	-0,084	0,191555	-0,48954	-0,68599	-0,27124	-0,12348	-0,5884	-1,51856	-0,19199	-0,19697	-0,74361	-0,46046	-0,09947	0,438063	-2,26731	-1,57198	-0,43106	0,439743	0,609926
NCI_DNA_PK_pathway_in_nonhomologous_end_	0,527227	-0,084	0,191555	-0,48954	-0,68599	-0,27124	-0,12348	-0,5884	-1,51856	-0,19199	-0,19697	-0,74361	-0,46046	-0,09947	0,438063	-2,26731	-1,57198	-0,43106	0,439743	0,609926
reactome_EPHA_mediated_growth_cone_collaps	-0,25494	-0,24265	-0,43536	-1,19135	-1,98238	-2,0277	-2,95894	-3,33775	-3,62142	0,356181	0,594099	0,050453	0,438246	0,998192	1,124037	-0,81429	0,972603	0,480536	0,440273	0,609926
reactome_Role_of_phospholipids_in_phagocytos	-0,34943	-0,2211	-0,11524	-0,36665	-0,20787	0,077548	-0,23727	-0,5304	-0,16273	0,303809	0,034948	1,342056	0,901553	1,098972	0,449967	0,752964	0,72262	0,761556	0,440574	0,609926
reactome_Sodium_Proton_exchangers_Main_Pat	-0,21448	-0,13768	0,060747	0,414709	0,762169	-0,85989	0,134316	-0,08129	0,040041	0,396799	0,250686	0,240846	-0,10053	-0,59511	-0,06802	1,016607	-0,2313	-0,0114	0,440753	0,609926
retinoate_biosynthesis_I	-0,26481	-0,02053	-0,0879	-1,38745	-0,41169	-0,38364	-0,40275	-0,57407	-0,02807	0,549598	-0,74068	-0,46759	0,345172	0,584768	0,288723	1,483669	0,659289	0,131954	0,439598	0,609926
KEGG_Biosynthesis_of_unsaturated_fatty_acids_	0,10888	-0,13118	-0,11535	-2,33159	-2,23554	-1,66086	-1,41775	-1,05276	-1,79204	3,134615	1,89834	1,875878	-0,96881	-1,00047	-0,42046	-5,23229	-3,96772	-3,19498	0,442359	0,610251
KEGG_Pathways_in_cancer_Main_Pathway	-3,84002	-3,57316	-3,11368	-6,07854	-0,75315	-5,92076	-9,07743	-9,74981	-7,8663	-1,0785	-4,37674	1,178587	0,607064	1,144815	0,849628	-6,05261	-3,25602	-2,43802	0,441464	0,610251
NCI_E_cadherin_signaling_in_keratinocytes_Pat	0,157558	-0,05967	-0,00518	-1,57828	-0,17851	-0,82404	-1,23911	-1,48142	0,129348	-0,31326	1,198203	0,74226	-0,16091	0,512952	-2,6428	-2,88748	-1,92485	0,450886	0,617017
reactome_Signaling_by_FGFR3_mutants_Main_P	-0,43574	-0,19511	-0,08403	-0,64666	-0,12162	-0,42432	-0,66327	-1,02202	-0,26668	0,728034	-0,63956	0,008786	-0,10232	-0,29383	-0,24702	-0,22662	-0,08283	-0,68348	0,450707	0,617017
KEGG_Thyroid_cancer_Main_Pathway	-0,00058	-0,13966	-0,18851	-0,46532	-0,35145	-0,63007	-0,60745	-0,48113	-0,88006	-0,06879	0,962717	0,410717	-0,40658	-0,16897	-0,15527	-1,1048	-0,93066	-0,60867	0,451825	0,617837
NCI_IL2_signaling_events_mediated_by_PI3K_Ma	0,061143	0,100583	-0,14937	-2,02231	0,135861	-2,54586	0,405657	0,317947	-0,14047	-0,0092	-0,09471	0,193966	0,335841	-0,0282	0,648805	-5,39732	-3,46468	-2,55831	0,453224	0,619479
reactome_Beta_oxidation_of_hexanoyl_CoA_to_	0,066052	-0,05807	0,044481	-0,18845	0,175409	-0,37207	0,600081	0,759953	0,521543	0,951509	0,262676	0,720929	-0,03553	0,004427	-0,22548	-1,99857	-2,00816	-1,76902	0,453439	0,619502
reactome_Viral_mRNA_Translation_Main_Pathwa	2,85335	0,81548	0,464493	-8,96762	-10,4734	-13,0154	7,962871	9,214591	5,126508	-3,85112	6,063481	4,680678	-2,29369	-1,41337	0,71012	-44,5527	-37,7857	-32,4198	0,453731	0,619628
KEGG_Glycolysis_Gluconeogenesis_Main_Pathwa	-0,27993	0,388011	0,25738	-5,23525	-1,61638	-2,9367	0,61744	0,434748	1,921907	0,203373	0,796901	-0,06697	1,707168	1,834611	0,43817	-6,63354	-8,84332	-6,4622	0,454349	0,6202
reactome_PKA_activation_in_glucagon_signalling	-0,38461	-0,13882	-0,21474	-0,41908	0,239223	-0,44862	-2,20413	-1,9537	-2,03466	-1,07318	-1,33514	0,257234	-0,56003	0,412488	0,626993	0,03254	0,209398	0,416126	0,455163	0,621039
NCI_Aurora_A_signaling_Pathway_(negative_regu	-0,16317	0,029139	-0,05302	-0,50075	-0,46091	-0,22393	0,963152	0,892555	1,045545	-1,38021	-0,88267	-0,74022	-0,38383	-0,25077	-0,31467	0,466083	0,759129	0,450807	0,455894	0,621765
KEGG_Glycosphingolipid_biosynthesis_globo_seri	0,189263	-0,16566	-0,37276	0,570852	1,14534	0,693709	-2,91479	-2,87824	-2,60986	0,949981	0,516997	-0,26263	-0,68475	-0,24104	-0,2062	-0,88788	-1,076	0,509301	0,456114	0,621793
NCI_Aurora_A_signaling_Pathway_(centrosome_	-0,10755	-0,01728	-0,07138	-0,25129	-0,6372	-0,13635	1,074053	1,06391	0,860016	-1,32219	-0,37886	-0,79541	-0,02958	0,226694	0,116	1,045135	1,670022	1,41846	0,456593	0,622173
biocarta_melanocyte_development_and_pigment	-0,08077	-0,17477	-0,24165	-1,11891	-1,07079	-1,8539	-1,86862	-1,88755	-2,36699	-1,67503	-0,46299	-0,59644	-0,23648	-0,36056	-0,14954	-2,40368	-2,76048	-1,81515	0,457473	0,622827
NCI_Caspase_Cascade_in_Apoptosis_Pathway_(D	-0,00828	0,0701	0,058668	-0,69436	0,025145	-0,09339	-0,59118	-0,44298	-0,62014	-0,38829	0,253694	-0,51032	-0,12981	0,089688	-0,15413	-0,93885	-0,94311	-0,30363	0,457311	0,622827
Akt_Signaling_Pathway_Promotes_Adipogenesis	-0,04374	-0,17698	0,293693	-1,47722	0,362385	-1,51261	0,068004	0,056624	0,14608	-0,16996	1,218414	-0,41917	0,566186	0,54371	0,292146	-0,35966	0,361621	0,604433	0,459953	0,625202
biocarta_agrin_in_postsynaptic_differentiation_M	-0,1818	-0,71608	-0,19195	-1,56436	-2,20325	-2,82063	-3,28239	-2,88593	-3,56812	0,52949	0,645375	1,375612	0,515655	1,019727	0,854348	-1,04927	-1,63481	0,253879	0,460221	0,625202
methylglyoxal_degradation_III	-0,05487	-0,0814	-0,04799	-0,6279	-0,52513	-0,62892	-0,56493	-0,61073	-0,59539	1,34722	0,059154	0,736031	0,031675	0,215826	-0,02634	-0,93046	-0,50521	-0,88857	0,460037	0,625202
NAD_ide_novoi_biosynthesis	0,018159	0,124834	0,102729	-1,25105	0,327132	0,37995	-2,15358	-2,30383	-2,45176	-0,6709	-0,26023	-0,54134	0,241981	-0,02294	-0,5115	1,087164	1,28731	-0,5051	0,45979	0,625202
reactome_Nonsense_Mediated_Decay_NMD_ind	2,8583	0,747536	0,346489	-9,41665	-11,0589	-12,7366	7,803379	9,099096	4,722389	-4,18394	6,441837	4,584274	-2,20271	-1,78653	0,743664	-44,7941	-37,9204	-32,9349	0,460059	0,625202
fatty_acid_beta-oxidation_unsaturated_odd_num	-0,06183	-0,10739	-0,07328	0,258426	-0,21379	-0,07552	0,303032	0,256857	0,145232	0,328086	0,898934	0,750692	0,123033	0,337724	-0,02139	-0,83525	-1,04072	-1,0031	0,461306	0,62613
reactome_Toxicity_of_botulinum_toxin_type_C_	-0,11638	-0,04786	-0,11363	0,238176	0,407572	0,379076	-0,55061	-0,72698	-0,56444	-1,26245	-0,57386	-1,13169	0,023204	0,184138	0,039367	3,123373	2,993519	3,549137	0,461217	0,62613
KEGG_Glioma_Main_Pathway	-0,55227	-0,14624	-0,97669	-1,00629	-0,56337	-2,32458	-3,57706	-2,95716	-4,46807	-1,45061	-0,78712	-0,29661	0,294625	0,130099	1,8944	-3,89809	0,039096	1,548564	0,462087	0,626644
NCI_ErbB2_ErbB3_signaling_events_Pathway_(m	0,094171	-0,01211	-0,09945	-1,0635	-0,11907	-0,4639	0,168989	0,371183	-0,02588	-0,0362	-0,23389	-0,12305	-0,09181	-0,10681	0,15577	-2,19401	-1,3164	-0,74182	0,4619	0,626644
biocarta_vegf_hypoxia_and_angiogenesis_Main_	-0,22061	-0,5625	-0,43914	-1,63928	0,469265	-1,23751	-3,39649	-3,43651	-3,59067	1,412245	0,306852	-1,23791	0,879923	0,478114	0,576165	-2,14645	-1,01561	-0,57794	0,462351	0,62673
CDK5_Signaling_in_Neurons	-0,49973	-0,58375	-0,53276	-1,20102	1,098472	-0,48159	-2,84282	-2,88317	-3,37062	0,578043	2,583719	2,045678	1,249914	1,078496	1,055815	-2,14616	-1,36515	-0,39502	0,462959	0,627008
IL-2_Pathway_Actin_Reorganization	-0,13871	-0,02844	-0,07987	-0,26126	0,078675	-0,45876	-1,21915	-1,27013	-1,19907	0,318493	1,365607	2,498018	0,526299	0,622678	0,930422	-1,77715	-1,91446	-1,84936	0,462866	0,627008
KEGG_Hepatitis_B_Main_Pathway	-0,30944	-0,95265	-0,55109	-1,76534	-1,61437	-3,06928	-0,33545	0,344382	-0,97125	1,50221	1,028902	1,6466	-0,14384	-0,68663	1,336092	-2,70701	-1,96983	-1,12381	0,463289	0,627182
ILK_Signaling_Pathway_Cell_Migration_Retractio	-0,61355	-0,51157	-0,30501	-0,47498	-0,2815	-0,32821	-3,45849	-2,96715	-3,04383	2,582478	-0,09795	3,575959	0,109567	0,11884	0,977171	-3,73011	-3,35342	-3,4577	0,465426	0,629351
leukotriene_biosynthesis	-0,12082	0,015826	0,097196	0,604038	-0,63645	0,32108	0,351732	0,1253	0,512929	-0,48581	-0,20651	0,024682	0,52887	0,291805	0,665247	0,790552	0,816284	0,138108	0,465497	0,629351
NCI_Class_I_PI3K_signaling_events_mediated_by	0,174308	0,052054	-0,39532	0,754477	1,406931	1,315568	-2,38887	-2,28372	-3,38721	0,669126	1,444597	1,46735	-0,31046	0,416677	-0,32833	-0,6094	-0,58102	-0,12196	0,465332	0,629351
NCI_Alpha9_beta1_integrin_signaling_events_Ma	-1,01898	-0,44031	-0,50204	-2,88313	-0,74321	-3,2593	-2,31354	-2,44631	-2,03613	1,281485	-1,18092	0,17996	1,401862	0,721357	0,727085	0,742341	0,772857	0,123161	0,465745	0,62941
reactome_Synthesis_of_PC_Main_Pathway	0,165797	-0,13919	-0,13883	-1,251	-1,73607	-1,99927	-0,71553	-0,86105	-0,98301	-2,72576	-1,48626	-1,55538	-0,26093	-0,17617	-0,02174	-2,86676	-1,68994	-1,16815	0,465945	0,62941
biocarta_rho_selective_guanine_exchange_facto	-0,26913	-0,04873	-0,18918	0,33729	-0,06349	-0,55978	-0,74062	-0,82994	-0,87095	-0,00638	0,361741	0,180068	-0,84429	-1,23727	-0,7733	-3,32046	-2,51357	-2,47502	0,467517	0,63126
NCI_CD40_CD40L_signaling_Pathway_(regulation_	-0,02155	-0,01129	-0,04828	-0,12824	-0,83396	0,200635	-0,51579	-0,42709	-0,64006	-0,314	0,573037	-0,1484	0,166783	0,093098	0,100695	-0,70692	-0,56386	-0,80641	0,468434	0,631461
NCI_CD40_CD40L_signaling_Pathway_(regulation_	-0,02155	-0,01129	-0,04828	-0,12824	-0,83396	0,200635	-0,51579	-0,42709	-0,64006	-0,314	0,573037	-0,1484	0,166783	0,093098	0,100695	-0,70692	-0,56386	-0,80641	0,468434	0,631461
reactome_Dissolution_of_Fibrin_Clot_Main_Path	0,016376	0,136425	-0,13423	-1,48564	-1,70012	-1,04976	-5,30544	-5,02387	-5,10386	-2,39283	-0,04387	-0,62228	1,344249	0,598174	0,909664	-1,46963	-0,49945	-1,28046	0,467889	0,631461
reactome_Hydrolysis_of_LPC_Main_Pathway	0,045374	0,275356	0,172381	-0,64752	-1,19423	-1,00174	0,45701	0,249056	0,686946	-0,76095	-1,64854	-0,62889	-0,18724	-0,36387	-0,62294	-0,95885	-0,12031	-0,65843	0,468477	0,631461
biocarta_il12_and_stat4_dependent_signaling_pa	0,255705	-0,20432	0,058472	-0,93786	0,035174	0,173261	0,775373	0,802237	0,740257	-1,03261	-0,85922	-0,51515	0,044145	0,777655	1,051652	0,557225	1,114381	-0,10224	0,469887	0,632813
reactome_Eukaryotic_Translation_Termination_M	2,815915	0,855026	0,461644	-9,24697	-10,6725	-13,1251	7,870212	9,050682	4,998703	-3,70215	6,308636	4,636639	-2,20094	-1,52375	0,949639	-43,8935	-37,3767	-32,2598	0,469742	0,632813
reactome_Classical_Kir_channels_Main_Pathway	-0,05381	0,047212	0,052575	-0,42169	0,134511	0,32101	-0,09361	-0,26403	0,068148	-0,11964	0,601896	0,318174	0,246963	-0,049	0,20869	-0,21555	-0,35087	-0,76573	0,470336	0,63309
reactome_Regulation_of_Complement_cascade_	0,145165	0,367471	0,622921	-2,56938	-2,87359	-2,33437	-1,69397	-1,42693	-1,09103	-0,21698	-0,52645	0,501694	0,104394	0,156947	-0,46427	-2,40616	-3,22989	-2,17639	0,470499	0,63309
NCI_Glypican_3_network_Pathway_(embryonic_c	-0,09564	0,050286	0,116565	-0,36742	0,244294	-0,31213	0,097088	0,003265	0,054753	0,191529	-0,01316	-0,25229	0,381473	0,174696	-0,05115	0,670257	0,045506	0,440729	0,471805	0,633753
NCI_Glypican_3_network_Pathway_(kidney_deve	-0,09564	0,050286	0,116565	-0,36742	0,244294	-0,31213	0,097088	0,003265	0,054753	0,191529	-0,01316	-0,25229	0,381473	0,174696	-0,05115	0,670257	0,045506	0,440729	0,471805	0,633753
NCI_Glypican_3_network_Pathway_(skeletal_syst	-0,09564	0,050286	0,116565	-0,36742	0,244294	-0,31213	0,097088	0,003265	0,054753	0,191529	-0,01316	-0,25229	0,381473	0,174696	-0,05115	0,670257	0,045506	0,440729	0,471805	0,633753
reactome_CTLA4_inhibitory_signaling_Main_Path	0,073641	-0,25112	-0,08744	-0,48234	-0,53434	-0,16299	-0,45346	-0,53823	-1,1442	0,514065	1,038712	0,7697	0,221077	0,235155	0,095709	-3,13584	-1,68442	-1,36963	0,471378	0,633753
reactome_Initial_triggering_of_complement_Mai	-0,13397	0,082808	-0,16153	0,178132	0,20286	0,01815	-0,56955	-0,74734	-0,22441	0,42437	-0,75205	0,375152	0,440306	0,623486	0,10591	1,78825	1,165111	1,575867	0,474151	0,63663
dopamine_degradation	0,068636	-0,16555	0,059843	-0,78245	-0,33217	-0,58826	-1,21356	-1,2764	-1,24508	-0,68889	-0,45277	-0,37161	0,235022	-0,12144	0,161086	0,241754	0,1427	0,388288	0,474566	0,636638
KEGG_NOD_like_receptor_signaling_Main_Pathw	0,84705	-0,36743	-0,10866	-1,94354	-1,86382	-2,76312	-0,68627	0,373664	-1,84245	0,135087	-1,80878	-2,24176	0,226224	-0,05249	0,808026	-4,50052	-3,65317	-2,28169	0,475424	0,636638
reactome_Peptide_chain_elongation_Main_Path	2,872186	0,877569	0,446202	-9,22306	-10,6584	-13,0129	8,145455	9,28722	5,316214	-3,43894	6,752694	4,943317	-2,15478	-1,37583	0,805859	-44,2082	-37,552	-32,3806	0,474844	0,636737
reactome_Mineralocorticoid_biosynthesis_Main_	-0,35106	-0,18147	-0,02751	0,393834	0,160578	0,610189	0,20221	-0,13691	0,478188	-0,76087	-0,02989	0,096198	0,073199	-0,33269	0,087962	2,046671	1,669994	1,418407	0,476239	0,638333
BRCA1_Pathway_Transcription_Coupled_Repair	0,019517	-0,01324	-0,08129	-0,04403	0,07153	-0,37691	0,191092	0,477369	-0,1431	0,618715	-0,01455	0,102054	-0,13278	-0,13398	-0,07336	-1,42468	-0,75902	-0,94882	0,476625	0,638574
biocarta_bcr_signaling_Main_Pathway	-0,11711	-0,50485	-0,42662	0,685314	-0,53849	-0,64285	-0,72271	-0,70554	-0,9316	0,088365	0,468598	-1,20575	-1,19921	-1,91885	-0,6583	-1,55646	-0,60267	0,210764	0,477354	0,63904
NCI_Signaling_events_mediated_by_focal_adhesi	0,100812	-0,05437	0,049122	-0,179	0,406458	-0,42954	-1,85803	-1,45639	-2,30857	-0,4176	0,334054	0,544426	0,726669	0,442341	0,752874	-0,82972	0,330458	0,812965	0,477383	0,63904
reactome_Acyl_chain_remodelling_of_PS_Main_	-0,04972	0,092284	0,031767	-0,80579	-1,76859	-1,52183	0,492196	0,559474	0,929497	-1,12011	-1,3748	-0,6022	-0,29447	-0,32302	0,079884	-0,87114	-0,15523	-0,56881	0,477732	0,639233
NCI_Arf1_Pathway_(actin_filament_polymerizati	0,013368	0,059741	-0,03857	-0,08206	-0,47565	-0,10475	0,064154	0,017492	-0,11506	-0,79293	-0,16869	0,13735	-0,30375	-0,2725	-0,28321	-1,11544	-0,9805	-0,53418	0,478903	0,64025
reactome_Formation_of_annular_gap_junctions_	-0,02955	0,055892	0,031077	-0,09992	-0,22125	-0,28727	-2,22665	-2,14269	-2,23734	-0,89234	0,857861	-0,06287	-0,05827	0,00128	0,239721	-0,04764	0,166692	0,41617	0,478861	0,64025
IP3_Pathway	-1,60914	-2,22358	-1,366	1,268519	4,497615	2,175773	-7,70171	-9,55122	-5,85261	-5,29708	0,598579	-7,32458	0,927062	-0,95205	2,677923	5,31592	5,190133	9,633287	0,479925	0,641341
pentose_phosphate_pathway	0,170388	-0,13995	-0,03212	0,036373	-1,10675	-1,58817	-0,39849	-0,38781	-0,70252	0,129104	-0,0159	-0,3071	-0,12875	-0,02786	0,039056	-2,68828	-1,91688	-2,5957	0,481566	0,642982
reactome_Electric_Transmission_Across_Gap_Jur	-0,16267	-0,16065	-0,16141	-0,20393	1,085205	-0,15477	0,530466	0,273953	0,670602	-0,55324	-0,1841	0,892738	0,219192	0,157511	0,31451	0,104908	0,430303	0,353645	0,481525	0,642982
reactome_DAG_and_IP3_signaling_Main_Pathwa	0,096089	-0,06991	0,08693	0,21806	0,665528	0,232076	-0,16536	-0,22872	-0,29028	0,36162	0,201561	-0,71494	0,044071	0,037618	-0,10578	0,111858	0,431011	0,119776	0,482186	0,643535
NCI_Signaling_events_mediated_by_VEGFR1_and	0,065182	0,030212	-0,07479	0,104897	0,14993	0,440972	-1,84925	-1,63706	-1,95728	-0,46969	-0,27572	-1,07125	-0,28404	-0,37247	-0,19174	-0,215	0,01765	0,515494	0,484484	0,646325
reactome_Activation_of_IRF3_IRF7_mediated_by	0,266661	-0,03842	-0,01413	-0,61258	-0,32011	-0,69939	-0,16937	-0,01298	-0,32942	0,276926	0,449253	-0,70318	-0,26761	-0,22798	0,022724	-2,41966	-1,86277	-1,53449	0,485801	0,647804
biocarta_tumor_suppressor_arf_inhibits_ribosom	-0,11692	-0,09339	-0,11663	0,167068	-0,23515	-0,049	-0,02548	-0,02949	0,139012	-0,12623	0,172584	0,160989	-0,05458	-0,2383	-0,30572	-0,12321	-0,16571	-0,12005	0,48765	0,649991
pyrimidine_deoxyribonucleosides_degradation	-0,26459	-0,09431	-0,05784	-0,38094	0,059393	-0,33471	-0,96479	-1,05331	-0,6819	-0,40012	-0,89477	-0,50488	-0,20008	-0,42482	-0,1005	0,313484	0,718233	0,268144	0,4879	0,650046
HGF_Pathway	-0,83532	-1,38629	-1,03112	-3,98338	-2,8116	-3,4002	-8,40604	-6,8546	-9,94973	0,306034	1,260584	3,867514	0,040403	0,936765	1,921076	-11,6967	-8,11595	-5,74977	0,488589	0,650686
NCI_JNK_signaling_in_the_CD4_TCR_Main_Pathw	-0,00989	-0,0802	0,033003	-0,48423	-0,15849	-0,29254	-0,095	-0,21042	-0,3668	-0,9757	-0,21867	0,262316	0,318654	0,663666	0,941777	1,332671	1,992709	1,754255	0,489034	0,651

4-aminobutyrate_degradation	0,046683	-0,08593	-0,05855	0,278393	0,076622	-0,52924	0,680638	0,719311	0,523431	-0,0419	0,113437	-0,73935	0,438174	0,114114	0,285854	-0,04835	0,182526	0,49234	0,489625	0,6515
WNT_Pathway_NFAT_Pathway	0,050676	-0,12923	-0,22209	0,692382	0,628583	-0,04877	-0,69719	-0,38512	-0,92569	-1,4764	0,035469	-1,0485	0,239394	0,264577	0,951747	0,382367	1,267979	2,51144	0,489827	0,6515
biocarta_tumor_suppressor_arf_inhibits_ribosom	-0,11049	-0,09481	-0,22338	-0,14062	-0,4149	-0,12798	0,18829	0,282509	0,183966	-0,06569	0,232173	0,054687	-0,15527	-0,26832	-0,27382	-1,3285	-1,03712	-0,68211	0,490223	0,651684
KEGG_Central_carbon_metabolism_in_cancer_M	-0,58626	-0,47555	-0,91443	-4,2503	-2,84384	-5,20863	-4,99467	-5,50646	-5,68547	-3,17897	-0,8803	0,358913	1,825306	0,965226	1,396584	-6,86566	-6,51336	-3,13543	0,490385	0,651684
KEGG_Hippo_signaling_Main_Pathway	1,955779	1,664995	0,59274	0,506378	0,392773	1,380325	-0,4104	0,100667	-2,75471	5,583295	2,892488	1,382185	-0,27743	-1,77358	0,586105	-9,5542	-7,77442	-7,85791	0,490877	0,652061
biocarta_tnf_stress_related_signaling_Main_Path	0,233549	-0,21755	-0,33624	-0,89866	-0,84444	-2,2382	-0,49928	-0,01472	-1,07362	-0,50788	0,151444	-0,26002	0,183607	0,926618	0,570897	-2,95941	-1,3865	-2,019	0,491285	0,652325
2-oxoglutarate_decarboxylation_to_succinyl-CoA	0,102695	-0,01876	0,018236	-0,23614	-0,08247	-0,3142	0,300388	0,390048	0,183272	-0,47587	0,660612	0,687989	-0,2432	-0,11317	-0,10995	-0,47196	-0,54186	-0,53156	0,492415	0,653444
NCI_Atypical_NF_kappaB_Main_Pathway	-0,27282	-0,1618	-0,15991	-0,60029	1,254092	-0,8016	-0,58219	-0,60602	-0,59391	-0,23535	-1,58103	0,265451	0,290859	-0,06046	0,414693	0,295327	-0,04962	0,321371	0,492548	0,653444
KEGG_Complement_and_coagulation_cascades_M	0,121567	0,29015	-0,08277	2,50861	2,76315	3,692108	-0,00766	-0,39403	0,797563	0,566991	-1,40236	1,101722	-0,93094	-0,26104	-1,5316	6,400319	5,465094	5,304257	0,492938	0,653683
reactome_Integration_of_energy_metabolism_M	0,091951	-0,05818	-0,03959	-0,43449	-0,58343	-0,61321	-0,04204	0,022663	-0,20059	0,47543	1,216118	0,630704	0,012803	0,026298	0,009609	-1,14956	-0,96147	-0,73528	0,493902	0,654683
KEGG_Glutathione_metabolism_Main_Pathway	-0,55107	0,300298	0,026093	-3,29502	-2,27727	-2,9355	1,765107	1,803834	1,133167	2,98028	1,581835	4,324166	-0,20793	0,41669	0,976996	-3,95446	-4,59167	-3,06981	0,495002	0,655862
TRAF_Pathway	-0,00198	-1,30389	-0,44552	-3,35655	-0,66022	-2,98593	-5,8185	-4,50662	-5,49918	6,419423	0,334114	4,825085	0,826397	-0,30689	0,565061	-3,53912	-4,33739	-2,02046	0,495869	0,656731
RANK_Signaling_in_Osteoclasts_Pathway_Express	-0,15083	-0,44288	-0,7003	-0,40814	-2,02395	-0,55366	-1,23546	-0,79514	-1,65036	0,360675	-0,77913	-1,00227	0,681169	0,376074	1,422103	-0,75203	-1,31099	0,584683	0,496203	0,656894
biocarta_reversal_of_insulin_resistance_by_leptir	-0,09535	-0,24263	-0,29696	-2,10342	-1,19746	-2,31189	-0,83854	-0,62462	-1,20142	-1,15895	-0,70476	-0,71598	0,079805	0,095798	0,229974	-3,56977	-2,37777	-2,61832	0,497687	0,6583
biocarta_reversal_of_insulin_resistance_by_leptir	-0,09535	-0,24263	-0,29696	-2,10342	-1,19746	-2,31189	-0,83854	-0,62462	-1,20142	-1,15895	-0,70476	-0,71598	0,079805	0,095798	0,229974	-3,56977	-2,37777	-2,61832	0,497687	0,6583
reactome_Nectin_Necl_trans_heterodimerization	-0,14617	-0,01754	-0,05657	0,822964	0,470103	0,505821	-0,41409	-0,51086	-0,42489	0,974151	0,411943	1,369046	-0,19524	-0,25431	0,009751	0,398851	0,255551	0,374255	0,498984	0,659735
NCI_Stabilization_and_expansion_of_the_E_cadh	0,046529	0,102039	0,117803	-0,26563	-0,1407	-0,41754	-0,7559	-0,70353	-0,94634	0,388519	0,605784	0,933206	-0,41767	-0,05513	0,021354	-2,71876	-2,31974	-2,08441	0,49956	0,659775
reactome_ATM_mediated_phosphorylation_of_r	0,047769	-0,18543	-0,17435	-0,01047	0,609617	0,121866	0,910323	1,090237	0,485639	-0,41402	0,171274	0,360468	-0,30666	-0,44804	-0,34665	-1,01972	-0,33493	-0,33224	0,49965	0,659775
reactome_Toxicity_of_botulinum_toxin_type_A_I	-0,15435	0,104159	0,031064	0,112453	0,056152	-0,4583	-0,6348	-0,75563	-0,63371	-1,29365	-0,66638	-1,26657	0,45184	0,452798	0,32195	2,47556	2,930688	3,074054	0,499475	0,659775
fatty_acid_beta-oxidation_peroxisome	0,110519	-0,01843	0,403637	-0,97368	-0,42395	-1,03323	0,033582	0,044684	-0,42395	2,73143	2,679004	3,106168	0,377685	-0,06891	-0,26954	-3,36279	-1,86898	-2,26245	0,502719	0,662881
putrescine_biosynthesis_II	-0,11537	0,038019	-0,0873	-0,47217	0,188031	0,38661	-0,06963	-0,13637	0,027897	0,683381	0,100013	-0,00675	-0,11875	0,143326	0,098293	0,203843	0,108007	0,334671	0,503066	0,662881
reactome_FasL_CD95L_signaling_Main_Pathway	-0,00428	0,076991	0,024974	-0,29862	0,196212	0,373143	-0,28453	-0,2078	-0,09995	-0,37241	-0,24225	0,370227	0,048573	-0,14843	-0,27232	-0,72102	-0,17169	-0,0508	0,502289	0,662881
reactome_Regulation_of_signaling_by_CBL_Main	0,032354	-0,07409	-0,07265	0,784039	0,304422	0,249864	-0,4119	-0,74197	-0,53396	-0,49564	0,255703	1,07637	-0,54469	-0,23676	-0,06914	-0,36983	-0,6159	-0,62917	0,50292	0,662881
thioredoxin_pathway	0,060684	0,131967	-0,00908	0,11519	0,542134	0,280389	-0,38855	-0,12837	-0,21435	-1,47848	-0,03104	-0,11814	-0,39355	-0,99825	-0,64329	-0,66227	-0,88039	-0,54463	0,502838	0,662881
glycoaminoglycan-protein_linkage_region_biosyn	-0,23001	-0,20977	-0,27629	0,560514	0,03112	0,653289	0,709022	0,693291	0,639933	-0,83288	-0,65256	0,168804	0,149075	-0,10684	0,182781	0,954776	1,319381	0,309819	0,504163	0,664046
biocarta_chaperones_modulate_interferon_signa	-0,10075	-0,07716	-0,0538	-0,11927	-0,2591	0,061067	-0,12856	-0,07175	-0,11773	0,315452	0,508115	-0,02106	0,004917	-0,21165	0,032553	-0,29634	-0,17476	-0,20507	0,505186	0,664269
cAMP_Pathway_Cell_Proliferation	-0,05174	0,042864	0,050458	0,052476	0,736511	0,528257	-1,08243	-0,94177	-1,08579	-0,85076	0,067722	0,630973	-0,74351	-0,38257	-0,30094	-0,72556	-0,78381	-0,31671	0,504995	0,664269
KEGG_Butanoate_metabolism_Main_Pathway	0,06691	-0,06287	0,118679	-0,22424	0,549608	-0,67663	1,026753	0,940413	0,999536	1,200249	1,1014	2,222818	0,681129	0,287341	-0,37428	-2,90725	-2,98009	-2,67544	0,504862	0,664269
reactome_Synthesis_of_IP2_IP_and_Ins_in_the_c	0,242269	-0,09139	0,077735	-0,65303	-0,59805	-1,22599	-1,66758	-1,6872	-2,2112	-1,60272	-1,22927	-3,29756	-0,97653	-0,77913	-0,67992	-1,56096	-1,15189	-1,10577	0,505162	0,664269
reactome_Interferon_alpha_beta_signaling_Main	0,818073	0,212528	0,880694	-1,00672	0,393881	-2,53593	0,645153	0,587761	0,957003	1,271641	2,672988	2,748433	1,916598	1,975271	3,392083	2,038349	1,881565	-0,25107	0,505412	0,664287
lysine_degradation_II_pipecolate_pathway	0,079131	-0,07083	-0,00358	-0,63817	-1,15713	-1,32846	0,262391	0,358457	0,21235	-0,3942	0,074715	-1,0221	0,679389	0,662998	0,517611	-1,55586	-1,30959	-0,4255	0,506947	0,665754
reactome_Biotin_transport_and_metabolism_Ma	0,441915	0,14768	0,063331	-0,05471	-1,04645	-0,64925	0,752554	0,9844	0,047676	1,392635	2,189091	0,982878	0,147082	0,09736	0,316574	-4,48728	-3,75703	-4,36348	0,50717	0,665754
reactome_Ficolins_bind_to_repetitive_carbohydr	-0,08034	-0,06252	0,023146	0,585216	0,413435	0,847361	0,233322	0,056371	0,536358	-2,0718	-0,99788	0,376071	0,295989	-0,15445	-0,1843	1,67245	0,699892	0,709255	0,507109	0,665754
reactome_CD28_dependent_Vav1_Main_Pathwa	-0,16932	-0,21207	0,069832	-1,99656	-0,75154	-0,08723	-0,43435	-0,20761	-0,65733	0,783743	-1,34753	-0,22054	0,099223	0,568299	0,700224	0,363568	0,5464	0,902171	0,50826	0,666903
reactome_NF_kB_activation_through_FADD_RIP_	0,156011	-0,08465	0,04097	0,033291	-0,16772	0,500482	-0,39233	-0,13197	-0,49098	0,134626	-0,35687	0,254634	-0,04552	-0,48288	-0,27957	0,06929	0,07369	-0,01924	0,509314	0,668005
biocarta_induction_of_apoptosis_through_dr3_ai	-0,00418	-0,3078	-0,19881	-0,89795	-0,88674	-2,29705	-1,93805	-1,63733	-1,7525	0,897178	0,31851	1,725429	0,754346	0,363764	0,040366	-1,07976	-1,11431	-1,18142	0,509632	0,668142
reactome_Interconversion_of_2_oxoglutarate_ar	0,09919	-0,26002	-0,15968	0,194775	0,648799	-0,15213	0,459594	0,39362	0,320513	0,974763	-0,49914	0,712332	-0,08988	-0,18748	-0,16881	0,220079	0,28684	0,241572	0,509889	0,668197
Akt_Signaling_Pathway_Glucose_Uptake	-0,01978	-0,09067	-0,04947	-0,41573	0,280349	-0,29592	-0,87369	-0,81337	-0,94956	0,574536	0,540367	-0,05083	0,100137	-0,01001	0,088118	-0,30203	0,041425	-0,12834	0,510581	0,668823
NCI_Plexin_D1_Signaling_Pathway_(receptor_rec	-0,14248	-0,19924	-0,22922	-0,82242	-0,49236	-0,69693	-0,043	0,036915	-0,03493	-0,28889	-0,10181	-0,67346	0,252568	0,467354	0,700887	0,923622	1,279216	0,645959	0,511015	0,66911
NCI_VEGFR3_signaling_in_lymphatic_endotheliun	-0,02506	-0,06407	0,09333	-0,31619	-0,2846	0,002644	-0,79383	-0,88383	-0,81262	0,297527	0,45762	-0,11781	0,292791	0,074172	-0,02303	-1,00049	-0,52389	-0,59641	0,511306	0,669185
reactome_Regulation_of_Rheb_GTPase_activity_	-0,01031	-0,13776	-0,12734	-1,5094	-0,9151	-2,02035	-0,81248	-0,58541	-1,01945	-1,6791	0,319584	-0,7724	0,127878	0,207668	0,172058	-2,74722	-2,02121	-2,03407	0,511502	0,669185
NCI_IL2_signaling_events_mediated_by_PI3K_Pat	0,084374	0,288077	0,023408	-0,78935	0,471391	-0,92238	-0,27327	-0,44203	-0,43382	0,109064	0,33877	0,706652	0,276185	0,075946	0,349762	-2,85891	-1,24404	-0,97666	0,511822	0,669322
cardiolipin_biosynthesis	-0,03124	-0,07674	-0,06222	-0,21843	-0,30496	-0,02691	0,181481	0,119015	0,087425	-0,67601	-0,43865	-0,12469	-0,29616	-0,22066	-0,09687	-0,10708	0,052227	0,382268	0,512125	0,669411
reactome_Tryptophan_catabolism_Main_Pathwa	-0,02773	0,054479	0,209694	-0,66564	-1,18062	-0,42564	-1,79072	-2,23593	-1,72991	-1,12922	-1,15127	-1,48828	0,217054	-0,29676	-0,41269	0,467555	0,93974	-0,00256	0,512534	0,669411
reactome_Uptake_and_function_of_anthrax_toxi	0,023604	-0,05183	-0,05244	-0,24965	-0,22356	-0,47582	-0,44748	-0,38241	-0,6064	-1,39715	-0,0859	0,016515	0,059193	-0,038	0,161708	0,523084	0,309658	0,997898	0,512365	0,669411
reactome_Alternative_complement_activation_M	-0,00062	0,212696	0,265274	-0,30443	0,388558	0,867423	0,183393	-0,10381	0,504467	-0,59157	-0,45558	0,327965	-0,40613	0,162226	-0,22195	1,165917	0,449628	1,047169	0,514955	0,67229
biocarta_il_6_signaling_Main_Pathway	0,154915	-0,15975	-0,15025	0,156404	-0,53505	-0,51481	-0,45441	-0,34495	-0,43438	0,440251	0,961385	0,037849	-0,31085	-0,29015	0,150088	-2,91362	-1,58056	-1,37298	0,516251	0,673136
GSK3_Signaling_Pathway	6,658664	4,011508	2,961412	4,46253	3,825608	7,119885	13,94227	15,78894	8,591739	-2,81557	7,691076	5,817254	-5,7759	-3,82123	-3,83185	-18,3526	-14,3514	-13,0464	0,516185	0,673136
TYROBP_Signaling_in_Natural_Killer_Cells	-1,15186	-0,92138	-0,66795	-2,0038	-3,31652	-3,09637	-2,87211	-3,07865	-2,85852	-1,90433	2,478925	1,456152	-1,15927	-1,63226	0,304612	-11,6777	-9,16757	-4,98204	0,515889	0,673136
reactome_Bicarbonate_transporters_Main_Pathv	-0,02594	0,07841	0,003602	-1,01312	0,479572	0,273077	-0,70474	-0,72685	-0,58039	-0,76624	-0,37699	-0,49855	-0,14854	0,14171	-0,1414	-0,25022	-0,24631	0,036629	0,51714	0,674013
reactome_N_Glycan_antennae_elongation_Main_	-0,1821	-0,48597	-0,22827	0,782583	0,501378	0,817152	-0,87232	-0,87379	-1,16243	0,422651	-0,06694	0,039349	-0,0125	-0,60967	-0,55794	1,571332	1,446792	1,070353	0,518036	0,674899
NCI_Alpha9_beta1_integrin_signaling_events_Pat	-0,1565	-0,14316	-0,17631	-0,57347	0,429479	-0,93077	-1,76742	-1,81761	-1,86817	0,127904	-0,66069	-0,31005	0,697558	0,744002	0,763003	0,157712	0,453671	0,397993	0,519291	0,676251
NCI_CXCR4_mediated_signaling_events_Main_Pa	-0,33251	-0,21042	0,000312	-2,35757	0,970408	-1,88816	-2,65989	-3,03672	-2,87911	1,637433	1,574825	2,444466	-0,23759	-0,50263	0,804815	-4,05778	-1,41977	0,610383	0,521474	0,67881
KEGG_Focal_adhesion_Main_Pathway	-1,77832	-2,05141	-1,25906	-9,85132	-7,27988	-9,30246	-18,8203	-19,2141	-18,7404	3,217206	-1,03304	4,736308	3,595769	3,639657	3,232275	-3,43553	-2,11764	-2,3854	0,522215	0,679491
biocarta_role_of_erk5_in_neuronal_survival_Path	0,044318	-0,14509	-0,19402	-0,20183	0,36062	-0,4355	-0,7981	-0,62414	-1,19329	0,094376	0,904749	0,955697	0,079769	-0,12817	0,134779	-1,91842	-1,26612	-0,74729	0,52334	0,68067
NCI_Angiopoietin_receptor_Tie2_mediated_signa	0,086963	-0,14933	-0,19405	-0,81004	-0,36834	-0,49999	-2,45808	-2,63822	-2,55121	1,16426	-0,12691	-0,95972	0,30266	-0,20338	0,016482	-0,81537	-0,2394	0,448311	0,524912	0,68243
biocarta_no2_dependent_il_12_pathway_in_nk_c	-0,03519	-0,0086	-0,02115	-0,12667	-0,13382	-0,30866	-0,20892	-0,25306	-0,26832	-0,20688	-0,21893	-0,08124	0,178424	0,124848	0,212112	-0,05273	0,00102	-0,00515	0,525225	0,682552
superpathway_of_steroid_hormone_biosynthesis	-0,43981	-0,29536	0,161316	1,017536	-0,35918	0,329362	0,178544	-0,24109	1,123328	0,397329	-0,16482	-0,27216	-0,18806	-0,55064	0,413168	2,507993	1,218134	0,201232	0,525808	0,683025
biocarta_ifn_alpha_signaling_Main_Pathway	0,268786	-0,0552	-0,02528	-0,50346	-0,13148	-1,08266	-0,21923	-0,06089	-0,42428	0,17531	1,05619	0,8416	0,58286	0,522865	1,050039	-1,35697	-1,28835	-1,65898	0,526036	0,683036
fatty_acid_beta-oxidation	0,088404	0,009698	0,222742	-0,80679	-0,4141	-0,43663	0,691552	0,706622	0,13802	2,531883	1,965714	2,473428	0,675586	-0,2424	0,114515	-2,24435	-1,46198	-1,52338	0,526712	0,683629
reactome_Striated_Muscle_Contraction_Main_Pa	-0,49964	-0,19127	0,250984	-0,23399	-0,21482	2,806673	0,539057	-0,43984	1,372715	0,217022	-0,69916	-1,14309	-0,01183	1,142945	0,518074	5,098225	4,053364	4,566361	0,527622	0,684524
NCI_IL8_and_CXCR1_mediated_signaling_events_	-0,03649	0,015265	0,011778	-0,30692	0,147266	-0,33764	-0,08386	-0,07468	0,005693	-0,69968	-0,49498	-0,64132	-0,13026	-0,00182	-0,06283	-0,13342	-0,08054	0,245342	0,528899	0,685895
Akt_Signaling_Pathway_p73_mediated_apoptosis	0,010476	-0,07313	0,334648	-1,17415	0,421112	-1,4118	-0,36669	-0,40636	-0,27585	-0,16318	0,604726	-0,83183	0,75163	0,654818	0,237733	-0,04772	1,012366	0,954396	0,530429	0,685977
glutathione_biosynthesis	-0,01257	0,00886	-0,00168	-0,41123	-0,48552	-0,70881	-0,60321	-0,42422	-0,88843	0,611062	0,789799	0,39623	0,007433	0,191496	0,030527	-1,02439	-0,59688	-0,76369	0,529939	0,685977
L-cysteine_degradation_II	-0,02936	-0,00997	-0,00895	0,15897	0,254525	0,006242	-0,0378	-0,09333	0,033402	0,426518	-0,06005	-0,00388	0,000102	0,027742	0,019416	0,034996	0,033486	-0,06515	0,530538	0,685977
MAPK_Signaling_Pathway	-6,80919	-5,99578	-4,43045	-19,7009	-10,1841	-22,7258	-38,9731	-40,4393	-33,1089	-0,49252	-5,77168	-3,553	10,11477	6,10592	10,26762	9,550885	4,156786	13,7087	0,530698	0,685977
NCI_CDC42_signaling_events_Pathway_(cell_mot	-0,23461	-0,12508	-0,36409	-0,51982	-0,29904	-0,36606	-1,12352	-0,96996	-1,11554	0,453412	0,391312	0,100821	0,256132	0,315995	0,222323	-0,06567	0,186942	0,111469	0,529433	0,685977
NCI_RAC1_signaling_Pathway_(neuron_projectior	0,045068	-0,01957	0,053542	-0,6599	-0,35345	-0,00498	-0,24975	-0,1997	-0,41563	-0,66468	-0,68573	-0,32158	0,208199	0,237888	0,333188	0,611353	1,045518	1,187865	0,530724	0,685977
reactome_Cytosolic_sulfonation_of_small_molecu	0,038741	-0,04901	-0,12836	-0,51642	-0,04243	0,049314	0,059558	-0,13171	-0,04022	0,910464	-0,47294	0,302981	-0,5315	-0,83198	-0,35512	-1,15832	-0,41084	-0,93192	0,53054	0,685977
reactome_Proton_coupled_monocarboxylate_tra	-0,01627	-0,10182	-0,03702	-0,15407	-0,0519	-0,39734	-1,05159	-1,2385	-0,93422	-1,1035	0,470759	0,289273	0,160124	0,210093	0,078754	0,359635	0,528718	0,515728	0,529253	0,685977
ILK_Signaling_Pathway_Cell_Motility	-0,0901	-0,07853	-0,16414	-1,3353	-1,06894	-1,71404	-2,56454	-2,69402	-2,76901	-0,65437	0,55335	1,171042	0,932238	1,013571	0,756271	-2,77943	-2,00148	-2,00922	0,532325	0,687762
KEGG_PPAR_signaling_Main_Pathway	-0,25062	0,019812	-0,18967	-0,94933	-0,18089	-0,01113	0,061186	0,023857	0,025183	-0,90618	-0,05442	0,107565	-0,10491	-0,03274	-0,12474	-0,09032	0,157761	0,157922	0,533207	0,688615
reactome_Sema4D_mediated_inhibition_of_cell_	-0,13814	-0,10641	-0,17413	-1,49171	-0,89641	-0,51889	-1,18337	-1,10051	-1,08468	0,03755	0,588163	-0,29726	0,128296	0,123378	-0,07117	-0,62191	-0,88385	-0,72052	0,533638	0,688886
acetone_degradation_I_to_methylglyoxal	-0,07802	0,139541	-0,1929	-0,08696	0,196943	0,381492	0,580963	0,458638	0,652392	-1,37418	-0,56634	-1,25593	-0,07532	-0,08163	0,202854	1,024042	1,023689	0,350632	0,534961	0,690022
NCI_Beta5_beta6_beta7_and_beta8_integrin_cell	-0,16659	-0,19704	-0,23329	-0,92568	-1,13467	-1,08659	-2,1925	-1,87659	-1,87347	-0,82904	-0,48442	1,08529	0,089734	0,144984	0,567421	-0,32261	-0,27407	-0,05974	0,534851	0,690022
NCI_E_cadherin_signaling_in_keratinocytes_Pathv	-0,45935	-0,10809	-0,16992	-0,52904	0,003534	-0,49174	-0,96178	-0,99929	-0,86512	0,084758	1,243329	0,57038	-0,57466	0,166386	-0,18737	-1,19955	-0,03348	-0,79372	0,535814	0,690806
NCI_Reelin_signaling_Main_Pathway	-0,00193	0,080517	-0,22262	-0,43484	-0,06466	-0,2011	-4,42681	-3,88098	-4,70392	0,744113	0,833905	0,834696	0,557328	0,284891	1,169957	-1,5065	-0,86789	0,035541	0,536012	0,690806
NCI_PAR1_mediated_thrombin_signaling_events_	-0,11592	-0,32241	-0,08943	0,067054	0,581531	-0,59125	-0,64513	-0,52223	-0,35751	0,172268	-1,06353	-1,06527	-0,28857	-0,39409	0,211902	1,129246	1,122652	1,754473	0,536286	0,690873
KEGG_Vitamin_B6_metabolism_Main_Pathway	-0,11767	-0,08515	-0,06396	0,007542	0,973745	-0,04265	0,253476	0,277212	0,25142	0,131776	0,133459	-0,24664	0,046495	0,083863	-0,13733	0,390152	-0,15504	0,342525	0,53658	0,690966
reactome_TRP_channels_Main_Pathway	-0,31	-0,34979	0,331879	-0,83584	1,435638	0,607454	-2,34473	-2,21914	-1,303	-1,56801	-1,53322	-2,99214	0,041599	-0,52845	0,499267	2,181744	1,779272	0,589496	0,53689	0,691079
NCI_Syndecan_2_mediated_signaling_events_Pat	0,00731	-0,09731	-0,0095	0,102484	0,111427	0,16386	-0,12144	-0,2069	0,057159	-0,1587	-0,32792	-0,97035	-0,41206	-0,26575	-0,31886	0,041611	-0,00356	-0,25816	0,537351	0,691387
reactome_Cobalamin_Cbl_vitamin_B12_transport	0,095916	0,357868	0,283348	-0,4007	-1,3352	0,008265	-0,34023	-0,26388	-0,68503	0,664905	0,852529	0,88905	-0,84467	-0,37552	0,111354	-2,09074	-1,55908	-1,27418	0,541307	0,69619
NCI_Wnt_signaling_network_Pathway_(Wnt_rece	-0,92394	-0,47034	-0,26511	-0,28999	0,261627	-1,51399	-1,86735	-2,18149	-0,96868	-1,03727	0,50058	-0,87755	-0,47323	0,501403	0,271347	1,198415	1,035832	1,509195	0,542077	0,696892
KEGG_Thiamine_metabolism_Main_Pathway	0,047316	0,052564	-0,04732	0,223351	0,328043	0,142594	0,096915	0,251737	-0,12325	0,958885	0,909483	0,15558	-0,29538	-0,1649	-0,30959	0,001531	-0,14571	-0,83909	0,542322	0,69692
HGF_Pathway_Gene_Expression_via_JUN_STAT3_	-0,27251	-0,36667	-0,53554	-1,38345	-1,73917	-1,08985	-2,58445	-2,2137	-3,17138	-1,0588	0,267704	1,095077	0,319217	-0,07028	0,527091	-3,1186	-2,70586	-1,8462	0,543133	0,697674
biocarta_multiple_antiapoptotic_pathways_from_	0,150085	-0,01062	-0,03922	0,2297	0,364195	-0,29795	-0,89046	-0,60374	-0,91282	0,177465	-0,59912	0,070672	-0,06126	-0,0177	-0,11797	-0,29985	-0,30041	0,240544	0,544154	0,697834
biocarta_multiple_antiapoptotic_pathways_from_	0,150085	-0,01062	-0,03922	0,2297	0,364195	-0,29795	-0,89046	-0,60374	-0,91282	0,177465	-0,59912	0,070672	-0,06126	-0,0177	-0,11797	-0,29985	-0,30041	0,240544	0,544154	0,697834
Chemokine_Pathway_Cell_Activation	0,126553	-0,25089	-0,21403	-0,39348	-1,44821	-0,72037	-1,14318	-1,10396	-0,73382	-1,79936	-0,78239	-1,68101	-0,16558	0,196932	-0,24258	-1,08837	0,447666	-0,86538	0,543782	0,697834
reactome_Circadian_Clock_Main_Pathway	0,272658	0,023935	-0,10119	-1,14974	-1,9191	-2,79461	-0,3496	0,068307	-1,2395	-1,13993	-0,00848	-0,67003	-0,10506	-0,32197	0,437699	-5,2289	-4,26629	-2,36281	0,543809	0,697834
biocarta_fc_epsilon_receptor_i_signaling_in_mas	0,089319	0,042131	-0,08861	-0,77735	-0,04641	-1,44628	-0,70801	-0,62217	-0,90399	-0,28072	-0,2438	0,120677	-0,10152	-1,12439	0,289433	-3,27741	-2,46699	-1,54682	0,544391	0,697851
reactome_SIRT1_negatively_regulates_rRNA_Exp	0,112262	-0,13199	-0,53596	0,232124	-1,10536	-1,9621	4,864889	5,608581	3,61705	1,973936	1,423116	0,383223	-0,65657	-1,21539	0,716988	-6,73746	-4,3354	-4,22498	0,544628	0,697868
KEGG_Tuberculosis_Main_Pathway	0,738374	-0,15176	0,243137	-1,4688	-1,22372	-0,19369	1,697072	1,914979	0,939088	-2,26424	0,780718	-2,87378	-1,20508	-1,14856	-0,62786	-1,84042	-0,26323	-0,44834	0,545056	0,697873
oxidative_ethanol_degradation_III	0,310576	0,068512	0,171321	-0,14807	0,012842	0,493874	0,474391	0,640637	0,418878	0,695225	0,019823	1,168154	0,530492	0,418193	0,553093	-0,20112	-0,1488	-0,43758	0,545081	0,697873
phospholipases	-0,42106	-0,48221	-0,53412	0,647803	-0,66427	-1,40773	-0,79751	-1,06742	0,080436	-1,79269	-1,98147	-1,44688	0,044876	-0,15439	0,243279	2,781852	3,909422	2,458585	0,545713	0,698397
NCI_mTOR_signaling_Pathway_(lipid_biosyntheti	0,019004	-0,02369	-0,11951	-0,37763	-0,30763	-1,05425	-1,383	-1,42505	-1,79102	0,423961	-0,28639	-0,82702	0,329653	0,428697	0,375142	-0,61008	-0,94169	-0,12819	0,546709	0,699383
IGF1R_Signaling_Pathway_Glucose_Uptake	-0,19253	-0,03483	0,050324	-0,12722	-0,02075	-0,1444	0,144104	0,014571	0,221553	-0,02879	0,112733	-0,03478	0,097185	-0,1674	0,08283	0,058595	0,202037	-0,19246	0,548368	0,700642
NCI_Aurora_A_signaling_Pathway_(protein_catab	-0,12239	0,06361	-0,0438	-0,27608	-0,24587	-0,01397	1,374186	1,365023	1,397794	-1,68614	-1,35694	-1,49714	-0,44579	-0,21551	-0,39831	-0,04305	0,50202	0,201158	0,548164	0,700642
reactome_CD28_co_stimulation_Main_Pathway	-0,1728	-0,04707	0,033848	-1,03865	-0,35919	0,399728	-0,24493	-0,23308	-0,25414	0,757896	-0,72029	0,157298	-0,17046	0,214693	0,346905	0,080656	0,441678	0,029054	0,5482	0,700642
reactome_Inflammasomes_Main_Pathway	-0,09059	0,01795	-0,04654	-0,74901	-0,1439	-0,36268	-0,34352	-0,29826	-0,40019	-0,14707	-0,86074	0,170219	0,237103	0,01174	0,158787	-0,36564	-0,64664	-0,34328	0,548648	0,700713
reactome_Urea_cycle_Main_Pathway	0,038715	-0,05321	0,006953	0,011189	-0,34286	0,236647	0,426244	0,283152	0,409322	0,330522	0,474984	0,575795	0,104274	0,227605	-0,45008	-0,8109	-1,84326	-0,60658	0,549695	0,701763
tetrapyrrole_biosynthesis	-0,01261	-0,04675	-0,01487	0,159781	-0,21104	-0,0578	-0,33908	-0,35091	-0,25876	-0,30027	0,097185	0,461935	0,040635	-0,12789	0,272638	0,39875	0,151579	-0,02437	0,550068	0,70195
NCI_AP_1_transcription_factor_network_Main_P	-0,57564	-0,45207	-0,54739	-0,17178	0,521236	0,072572	-1,90538	-1,86051	-1,99232	-0,61603	-0,19671	-0,47768	-0,01212	-0,01323	-0,01754	0,722719	0,466357	0,973175	0,550553	0,702282
NCI_Stabilization_and_expansion_of_the_E_cadh	0,145354	0,05927	0,042419	-0,47989	-0,04759	-0,1858	-0,46104	-0,56694	-0,69605	0,369308	1,424549	1,646949	-0,73661	-0,31212	-0,0762	-2,06939	-1,10016	-0,98368	0,550922	0,702314
sulfite_oxidation	0,053219	0,040356	0,043536	0,23213	-0,18973	-0,07933	-0,07681	-0,00314	-0,18341	-0,79353	0,017656	-0,04629	-0,21906	-0,07125	0,028201	-0,11462	-0,02048	-0,24816	0,551029	0,702314
NCI_Alpha_synuclein_signaling_Pathway_(cell_de	-0,12522	-0,11284	0,080341	-0,0915	1,345872	0,319723	-1,3152	-1,50722	-1,18274	-0,58592	-0,14615	0,405755	0,110497	0,384995	0,895916	1,583574	0,216852	1,352638	0,552596	0,703735
NCI_Alpha_synuclein_signaling_Pathway_(protea	-0,12522	-0,11284	0,080341	-0,0915	1,345872	0,319723	-1,3152	-1,50722	-1,18274	-0,58592	-0,14615	0,405755	0,110497	0,384995	0,895916	1,583574	0,216852	1,352638	0,552596	0,703735
Circadian_Pathway	0,033012	0,157175	0,224939	0,270108	0,056276	1,411918	1,830898	1,69453	1,815529	0,307109	0,446123	-0,67334	-0,31274	-0,05603	-0,36537	-0,04985	-0,24004	-0,3344	0,552938	0,703883
reactome_Vpr_mediated_induction_of_apoptosis	0,030739	-0,0445	-0,05382	-0,12053	-0,60972	-0,13499	0,259015	0,266472	0,206225	-0,20248	0,262166	0,000704	-0,17196	-0,07141	-0,0096	-0,96415	-1,00053	-0,6788	0,554723	0,705867
biocarta_bcr_signaling_Pathway_(re_entry_into_	-0,09567	-0,2069	-0,12807	0,820755	-0,11815	-0,33892	0,420768	0,716903	0,402915	-0,16982	-0,27838	-1,40734	-0,67272	-0,93831	-0,13308	0,296807	0,651785	1,12275	0,555687	0,705868
biocarta_pten_dependent_cell_cycle_arrest_and_	-0,00794	-0,06417	-0,0714	0,514585	-0,68799	0,244645	-0,36519	-0,54534	-0,55541	0,417118	0,769224	0,770029	-0,10054	0,15947	0,037723	-0,32138	-0,24716	0,185686	0,555317	0,705868
ILK_Signaling_Pathway_Loss_of_Occludin_Barrier	-0,23222	-0,33807	-0,08242	-1,19718	0,200851	-0,60831	-5,29841	-5,20163	-5,01564	1,317789	1,1357	2,304244	1,0691	0,819431	1,145962	-1,74911	-1,95805	-1,72387	0,555857	0,705868
NCI_Caspase_Cascade_in_Apoptosis_Main_Pathw	-0,22961	-0,58544	-0,52401	-2,98965	-1,79742	-4,13196	-4,04965	-3,26528	-3,77345	1,929254	2,170691	2,091363	0,654528	0,809838	-0,1334	-4,67977	-3,35868	-1,86082	0,555549	0,705868
reactome_WNT5A_dependent_internalization_of	-0,32363	-0,15881	-0,0783	-0,28483	0,164572	-1,26116	-0,27408	-0,45025	-0,47893	-1,4607	1,451295	1,761217	0,259273	0,343364	0,686147	0,215904	0,236498	0,531122	0,555498	0,705868
NCI_Presenilin_action_in_Notch_and_Wnt_signal	0,173865	0,338494	0,225487	0,391247	-0,1663	0,673762	0,199963	0,295214	0,317644	-0,02394	-0,10418	0,4862	-0,36091	0,076708	-0,09435	0,377554	-0,28142	0,599556	0,556432	0,70631
KEGG_MAPK_signaling_Main_Pathway	-1,29431	-1,37332	-1,07585	-10,5534	-7,19018	-6,23279	-8,70662	-9,38525	-8,51113	-9,32744	-7,91834	-7,56081	2,86193	2,75307	3,623761	7,260361	8,54255	9,857581	0,557193	0,7067
KEGG_Melanogenesis_Main_Pathway	-0,03045	-0,11996	-0,25527	-1,40445	-1,25169	-1,88518	-1,50943	-1,44423	-2,03002	-1,40015	-0,12754	-0,62049	-0,47384	-0,36084	-0,33729	-2,95032	-3,53079	-2,39364	0,557055	0,7067
lanosterol_biosynthesis	0,045093	0,01903	0,027197	-0,18336	0,006773	-0,11782	0,111393	0,175703	0,1163	-0,42456	-0,15565	-0,38882	0,119702	0,10405	-0,00318	0,216951	0,131959	-0,06039	0,55752	0,706827
KEGG_Amyotrophic_lateral_sclerosis_ALS__Main	0,188967	0,241347	0,061302	-0,31787	-0,80757	0,369591	1,476796	1,564133	1,469347	0,745047	0,37571	0,942721	-0,32956	-0,22054	-0,20217	-1,28732	-3,43437	-3,23956	0,560468	0,709699
Ras_Pathway_Golgi_Trafficking_and_Vesicle_Forr	-0,04734	-0,17106	-0,23306	0,289294	-0,01818	-0,49477	-0,90232	-0,62403	-1,26823	1,953054	0,275691	0,530795	0,022302	0,264117	0,008392	-1,88347	-1,42158	-1,06363	0,560076	0,709699
sphingomyelin_metabolismceramide_salvage	0,109473	0,114223	0,097865	-0,0359	-1,37118	-1,11288	0,066749	0,135165	-0,4066	0,875276	0,846784	-1,08615	-0,19151	-0,48624	-0,52874	-1,34412	-1,71172	-1,36499	0,560254	0,709699
NCI_Thromboxane_A2_receptor_signaling_Pathw	-0,10044	0,026869	0,096643	-0,63683	-0,35528	-0,76875	-0,44352	-0,54896	-0,16621	-0,96365	-0,78624	-0,67265	-0,02245	0,101092	0,147152	0,485909	0,163055	0,813241	0,561863	0,71026
NCI_Thromboxane_A2_receptor_signaling_Pathw	-0,10044	0,026869	0,096643	-0,63683	-0,35528	-0,76875	-0,44352	-0,54896	-0,16621	-0,96365	-0,78624	-0,67265	-0,02245	0,101092	0,147152	0,485909	0,163055	0,813241	0,561863	0,71026
putrescine_degradation_III	0,061893	-0,07787	-0,0522	-1,22271	-1,05841	-1,02796	-2,50257	-2,73359	-2,50899	-0,97049	-0,61706	-0,55747	0,742575	0,68832	0,667191	-0,66491	-1,04915	-0,23904	0,562052	0,71026
reactome_Amino_acid_synthesis_and_interconve	0,030041	-0,0538	-0,04195	-1,06149	-0,97288	-0,27345	0,493638	0,536903	0,410594	0,0168	1,104488	0,549008	-0,05495	0,050253	0,553746	-2,89245	-2,20966	-1,97478	0,561574	0,71026
SMAD_Signaling_Network_Pathway	-3,40599	-2,91836	-3,09192	-1,24288	0,339776	-0,34527	-4,4869	-4,71336	-2,38428	-1,06106	-2,55566	-2,23944	3,241719	1,094502	0,762119	14,68339	9,655538	10,14249	0,561183	0,71026
methionine_salvage	-0,02751	-0,07472	-0,01356	-0,81251	-1,04963	-0,60769	0,067135	-0,02063	0,125021	-0,56411	0,009744	-0,38304	0,02687	0,093009	0,201629	-0,3008	-0,34286	-0,27693	0,563724	0,712085
EGF_Pathway_Cell_Motility	0,022495	-0,02195	-0,03526	-0,65134	-0,22453	-0,7423	0,998872	1,117799	0,733035	-0,64571	-0,4313	-0,68695	0,068225	-0,17744	0,066938	-0,69593	-0,66427	-0,27385	0,564835	0,71291
reactome_Galactose_catabolism_Main_Pathway	0,002309	0,043821	0,157133	-0,48258	-0,39508	-0,38227	0,224604	0,145304	0,283585	-0,91083	-0,63197	-0,64812	-0,10033	-0,17271	0,247808	0,333003	0,543848	0,257785	0,564677	0,71291
biocarta_trefoil_factors_initiate_mucosal_healing	0,033625	-0,33434	-0,18325	-0,71313	-1,33749	-1,01907	-0,67364	-0,52337	-0,79147	0,38819	1,088928	0,335651	-0,28976	-0,76191	0,367466	-1,70554	-1,59634	-1,47274	0,565341	0,713259
cAMP_Pathway_Protein_Retention	-0,0823	-0,03471	-0,05244	-0,57923	-0,16536	-0,88174	-0,56166	-0,32133	-0,7163	-0,38443	1,181281	1,414707	-0,99906	-0,67873	-0,31303	-2,36464	-1,83053	-0,95386	0,566747	0,714449

NCI_E_cadherin_signaling_in_keratinocytes_Pathv	-0,2101	-0,11165	-0,01058	-0,57267	0,367669	-0,39233	-0,76246	-0,8871	-0,7066	0,489965	1,070728	1,478191	-0,37771	-0,09242	-0,02892	-1,42184	-0,44795	-0,73577	0,566525	0,714449
NCI_IL12_signaling_mediated_by_STAT4_Main_P	-0,03077	-0,15026	-0,06016	-0,21357	-0,41977	-0,34918	-0,17228	0,000308	0,045785	-0,26019	-0,36873	0,319004	0,22214	0,301024	0,54371	0,897342	0,815618	0,133477	0,566972	0,714449
NCI_Endothelins_Pathway_(positive_regulation_c	0,08545	-0,07805	0,017792	0,220558	0,215286	-0,77727	-1,25436	-1,22944	-1,26633	0,842868	0,541276	-0,21952	0,130219	-0,1256	0,077267	-1,29112	-1,00046	-0,76956	0,567991	0,715155
reactome_Non_integrin_membrane_ECM_intera	-0,07972	-0,27098	-0,12093	-0,65209	-1,30174	-0,52099	0,091229	0,135554	0,040982	0,069512	-0,19865	0,024861	0,565183	0,64525	0,670564	0,616318	0,448522	0,579727	0,567923	0,715155
ILK_Signaling_Pathway	-6,6067	-4,46728	-3,93024	-17,7311	-9,43578	-16,5014	-31,7865	-34,6889	-25,3722	3,38641	-3,11181	-2,02187	11,26782	6,651601	8,319292	9,31385	3,064893	9,184732	0,568664	0,715713
VEGF_Pathway_Prostaglandin_Production	-0,08809	0,011665	-0,01527	-0,69154	-1,63612	-1,93216	-0,51491	-0,18207	-1,0759	-1,40808	0,75616	-0,47847	0,489696	0,302632	0,594423	-2,41173	-1,80394	-1,95385	0,569164	0,716053
EGF_Pathway_Gene_Expression_via_FOS_NFKB2	-0,35344	-0,44357	-0,43388	-2,60524	-1,12132	-2,25224	0,182414	0,869818	-0,33811	-1,10907	-1,13605	-2,55151	1,029517	0,210422	1,510094	-2,07872	-1,48749	0,62182	0,570859	0,717315
KEGG_Endocrine_and_other_factor_regulated_ca	-0,48461	-0,16071	-0,32105	0,112244	0,121033	-0,5726	-1,24447	-1,22334	-0,30112	-0,70959	-0,30633	0,091988	0,453821	1,533083	2,195516	2,891312	3,815199	5,552473	0,570823	0,717315
reactome_Opsins_Main_Pathway	-0,07485	-0,06437	0,16185	0,201878	0,489832	-0,28921	0,235494	0,310568	0,323701	-0,3103	0,156076	0,159837	0,54711	-0,25791	-0,05162	0,773695	0,439273	0,733501	0,570402	0,717315
NCI_IL2_mediated_signaling_events_Pathway_(G	0,001975	-0,15791	-0,11854	0,084717	-0,10877	-0,98764	-0,14173	-0,01583	-0,32408	0,167003	0,571919	0,710291	-0,24718	-0,25327	-0,05487	-0,74114	-0,96332	-0,54992	0,571537	0,717878
reactome_SEMA3A_Plexin_repulsion_signaling_b	-0,23663	-0,22316	-0,40657	-1,01407	-0,40902	0,002169	-3,22768	-3,14785	-3,53294	-1,36864	-1,32835	-0,8183	0,212693	0,141529	-0,14479	0,259315	-0,25636	0,955606	0,571786	0,717901
reactome_Nef_Mediated_CD8_Down_regulation_	-0,01655	0,004823	0,086913	-0,24109	0,076905	0,150318	-0,43352	-0,34691	-0,50339	-0,33911	0,543028	0,185269	0,453234	0,097474	0,74801	0,073434	0,328903	0,204041	0,572024	0,717911
NCI_CXCR3_mediated_signaling_events_Pathway	-0,35463	0,009199	0,217329	0,645857	0,392863	0,321194	-0,41843	-0,38111	-0,35462	1,327388	1,205994	-0,55052	-0,88128	-0,51554	0,262354	0,098197	0,941902	-0,73482	0,574488	0,718846
NCI_ErbB4_signaling_events_Pathway_(glial_cell_	-0,12579	0,03155	-0,05414	-1,22851	-1,25276	-1,85777	0,403185	0,478235	0,059656	-0,64784	-0,90357	-0,39226	0,579783	0,678195	0,55126	0,064025	0,218835	0,651453	0,573499	0,718846
NCI_Regulation_of_Telomerase_Pathway_(cell_cy	0,080179	0,185551	-0,02584	-0,15458	-0,25476	-0,48008	-0,14736	-0,25418	-0,29718	0,693182	-0,18268	0,003971	0,040173	-0,08015	-0,11577	-0,37143	-0,68577	-0,46915	0,575695	0,718846
p53_Signaling_Pathway_Breast_Cancer	0,002122	-0,20328	-0,12711	0,796829	0,160383	-0,3396	-1,02425	-0,55086	-1,83541	1,82264	2,077625	1,697306	0,76595	0,386345	0,62748	-1,70681	-1,21608	-1,44052	0,575769	0,718846
p53_Signaling_Pathway_Cell_Cycle_Arrest	0,002122	-0,20328	-0,12711	0,796829	0,160383	-0,3396	-1,02425	-0,55086	-1,83541	1,82264	2,077625	1,697306	0,76595	0,386345	0,62748	-1,70681	-1,21608	-1,44052	0,575769	0,718846
p53_Signaling_Pathway_Cell_Growth_Accumulati	0,002122	-0,20328	-0,12711	0,796829	0,160383	-0,3396	-1,02425	-0,55086	-1,83541	1,82264	2,077625	1,697306	0,76595	0,386345	0,62748	-1,70681	-1,21608	-1,44052	0,575769	0,718846
p53_Signaling_Pathway_DNA_Repair	0,002122	-0,20328	-0,12711	0,796829	0,160383	-0,3396	-1,02425	-0,55086	-1,83541	1,82264	2,077625	1,697306	0,76595	0,386345	0,62748	-1,70681	-1,21608	-1,44052	0,575769	0,718846
p53_Signaling_Pathway_Exosome_Mediated_Sec	0,002122	-0,20328	-0,12711	0,796829	0,160383	-0,3396	-1,02425	-0,55086	-1,83541	1,82264	2,077625	1,697306	0,76595	0,386345	0,62748	-1,70681	-1,21608	-1,44052	0,575769	0,718846
p53_Signaling_Pathway_Inhibition_of_Angiogene	0,002122	-0,20328	-0,12711	0,796829	0,160383	-0,3396	-1,02425	-0,55086	-1,83541	1,82264	2,077625	1,697306	0,76595	0,386345	0,62748	-1,70681	-1,21608	-1,44052	0,575769	0,718846
reactome_ERK_MAPK_targets_Main_Pathway	0,09277	-0,35681	-0,44569	-0,5266	-1,3082	-0,85738	-1,26869	-0,98074	-1,95583	-0,84599	1,957383	-0,09502	0,066556	0,464889	0,660029	-1,93413	-2,17476	-1,12732	0,57368	0,718846
reactome_misspliced_LRP5_mutants_have_enhar	0,190521	-0,13085	-0,05117	0,472073	0,012177	-0,31287	-0,14771	0,022438	-0,10248	-2,14929	-0,7593	-1,75717	-0,25006	-0,05546	0,278271	0,45874	0,969476	1,321524	0,575644	0,718846
reactome_RNF_mutants_show_enhanced_WNT_	0,190521	-0,13085	-0,05117	0,472073	0,012177	-0,31287	-0,14771	0,022438	-0,10248	-2,14929	-0,7593	-1,75717	-0,25006	-0,05546	0,278271	0,45874	0,969476	1,321524	0,575644	0,718846
reactome_Signaling_by_Wnt_Main_Pathway	0,190521	-0,13085	-0,05117	0,472073	0,012177	-0,31287	-0,14771	0,022438	-0,10248	-2,14929	-0,7593	-1,75717	-0,25006	-0,05546	0,278271	0,45874	0,969476	1,321524	0,575644	0,718846
biocarta_acetylation_and_deacetylation_of_rela_	-0,10468	-0,13516	-0,14711	-0,39339	-0,16126	-0,04411	-1,11731	-1,00837	-1,31034	0,27248	-0,2199	0,515599	0,46843	-0,10523	0,379057	-0,26653	-0,49828	-0,07677	0,576433	0,719387
reactome_DAP12_signaling_Main_Pathway	-0,30597	-0,06196	-0,2894	-1,50081	-0,69478	-1,96725	-1,11387	-1,1735	-1,4582	2,273034	1,616573	0,508717	-1,75158	-1,36318	-1,3203	-5,22638	-3,6494	-3,25867	0,577828	0,720839
biocarta_phospholipids_as_signalling_intermedia	-0,17575	-0,81604	-0,4106	-1,73282	-2,63773	-3,11599	-3,25043	-2,56458	-3,79108	1,724508	0,784993	0,317319	-1,03921	-0,89445	0,032565	-5,31548	-3,94495	-1,64963	0,578076	0,72086
reactome_repression_of_WNT_target_genes_Ma	0,092634	0,037102	0,06006	0,111388	0,001623	0,085244	-0,30822	-0,1352	-0,4659	0,855936	-0,30997	0,495147	0,047121	0,107436	0,057178	-1,00934	-0,16098	-0,37294	0,578332	0,72089
Akt_Signaling_Pathway_Regulation_of_Cyclic_Nu	0,03285	-0,13522	0,277562	-1,18523	0,296293	-1,31873	0,223522	0,305831	0,298558	0,412262	1,57892	-0,58313	0,786143	0,737605	0,397562	-0,55701	0,297924	0,56635	0,578807	0,721194
cAMP_Pathway_Fatty_Acid_Metabolism	-0,40134	-0,45668	-0,3442	-0,27384	0,613656	0,373362	-2,20137	-2,77717	-0,62905	-2,99808	-1,78094	-0,32298	-1,37048	-0,69206	-1,01785	2,323833	2,548205	1,400272	0,57982	0,721522
NCI_Signaling_events_mediated_by_HDAC_Class_	-0,30115	-0,29057	-0,42453	0,396478	0,107342	-1,59728	-0,40159	-0,39104	-0,10779	-1,19696	-1,51011	-1,05764	0,159597	-0,45041	-0,18614	0,23913	-0,07147	0,37831	0,579369	0,721522
reactome_Packaging_Of_Telomere_Ends_Main_F	0,041543	-0,23419	-0,63588	0,730533	-0,53646	-1,52154	4,17154	4,789957	3,298244	2,37921	0,567706	0,0033	-0,46565	-0,74546	0,73349	-4,23832	-1,76844	-2,58864	0,579932	0,721522
triacylglycerol_biosynthesis	-0,31117	-0,17237	-0,18134	-1,14691	-0,7208	-1,67885	0,227849	0,474193	-0,06018	-0,81093	-0,82197	-0,35151	0,380314	0,012637	0,881088	-1,31923	-1,09877	-0,54537	0,579997	0,721522
biocarta_ceramide_signaling_Main_Pathway	0,484218	0,008226	0,241854	-0,64034	-1,4499	-1,63268	-2,18455	-1,69242	-2,77607	-1,86405	-0,23836	-0,34441	-0,78281	-0,43295	-0,35015	-2,06974	-1,53009	-0,67421	0,580781	0,722208
reactome_Nef_and_signal_transduction_Main_Pa	-0,01516	-0,04381	-0,05525	-1,62949	-0,90713	-1,2102	-0,20857	-0,11401	-0,32558	-0,89451	-0,45286	-0,88274	0,044523	0,384788	0,643805	-0,98828	-1,81421	-1,0829	0,581942	0,723363
KEGG_Sulfur_metabolism_Main_Pathway	0,303686	0,123135	0,127019	-0,16877	-0,45571	0,05091	-0,59363	-0,36623	-0,91402	-0,29146	0,30647	-0,28448	-0,81269	-0,69649	-0,5156	-2,52596	-2,07514	-1,57465	0,582623	0,723736
reactome_Ephrin_signaling_Main_Pathway	-0,08487	-0,40147	-0,24121	-0,10947	-0,59033	0,310859	-2,37117	-2,12841	-2,83379	-0,94166	-0,42146	-1,56919	-0,08023	0,525514	0,25023	0,055744	0,716998	1,597073	0,582707	0,723736
mTOR_Pathway_Actin_Organization	-0,21352	-0,12433	-0,22878	-0,87575	-0,27336	0,37921	-7,16485	-7,10534	-7,59781	0,556813	-0,71394	0,805018	0,976283	-0,03465	0,338213	-0,90519	-1,03775	0,843084	0,58356	0,724507
NCI_Presenilin_action_in_Notch_and_Wnt_signal	0,072218	0,064022	-0,19912	0,180393	0,101917	-0,29322	-0,36989	-0,18036	-0,81853	2,330054	0,15607	1,193423	0,018677	0,489525	0,772425	-0,13131	-0,81446	1,005226	0,584255	0,724792
RhoA_Signaling	-0,26652	-0,32941	-0,35845	-2,21552	-2,48599	-3,18419	-1,50152	-1,71298	-2,64315	-0,78631	1,48865	-0,21711	-0,42387	0,169144	0,610761	-3,49639	-1,91549	-1,39451	0,584077	0,724792
reactome_ERKs_are_inactivated_Main_Pathway	-0,11358	-0,40233	-0,32886	-1,09537	-1,50919	-1,90569	-1,05773	-0,81275	-1,28064	0,901543	1,366179	1,120367	0,134184	0,161399	0,438118	-2,06219	-1,61082	-1,58102	0,584902	0,725306
reactome_TRIF_mediated_programmed_cell_dea	-0,0925	-0,0836	-0,05622	0,237008	-0,01763	0,090419	-0,53105	-0,41319	-0,5745	-0,32615	-0,09172	0,762234	-0,28002	-0,162	-0,17363	-0,49812	-0,29598	-0,53089	0,58523	0,725425
biocarta_induction_of_apoptosis_through_dr3_a	-0,0416	-0,35721	-0,24597	-0,7861	-0,33879	-1,88061	-2,20821	-1,91316	-2,06611	1,016292	0,378549	1,979603	1,006518	0,507961	0,062252	-0,89224	-0,67032	-0,6774	0,585773	0,72552
reactome_MyD88_Mal_cascade_initiated_on_pla	0,225235	0,308246	0,001814	0,90144	0,764847	-1,28474	-1,06669	-0,99141	-1,44688	0,416136	0,564065	0,384135	0,167627	-0,17561	-0,11154	-1,62972	-1,19618	-0,79819	0,585633	0,72552
biocarta_regulation_of_spermatogenesis_by_cre	-0,10063	0,33821	0,341554	-0,01853	-0,5411	-0,31128	0,505026	-0,02029	0,479145	1,190171	-0,01195	-1,13284	0,366995	0,629635	0,481249	-0,36162	-0,69688	-0,1161	0,588405	0,726788
GSK3_Signaling_Pathway_Glycogen_Synthesis	0,332835	0,246492	0,087324	-1,48413	0,088079	-0,12315	-0,61078	-0,72249	-0,70644	1,012069	-1,23454	1,961885	-0,76726	-0,28391	-0,0692	-1,72807	-1,62317	-0,62062	0,58843	0,726788
ICOS_signaling_in_T-helper_cells	-0,05646	-0,03408	-0,1776	0,239178	0,385195	0,975253	0,618834	0,738456	0,621826	0,511318	-1,27622	-0,94382	-0,5282	-0,40421	-0,21001	-0,40959	0,808399	0,617737	0,587807	0,726788
KEGG_Type_II_diabetes_mellitus_Main_Pathway	-0,09658	-0,06624	0,075041	-0,13302	0,029899	0,072355	0,060823	-0,01979	0,128151	-0,28036	0,0241	0,073519	-0,09267	-0,03379	0,121668	0,323515	0,242283	0,011356	0,587854	0,726788
NCI_Angiopoietin_receptor_Tie2_mediated_signa	-0,02195	-0,20557	-0,04775	-0,78016	-1,10427	-1,42578	-0,73047	-0,72639	-0,8822	0,506138	-1,08136	-0,82567	0,414195	0,209478	0,39813	-0,31174	0,274696	0,198478	0,587151	0,726788
reactome_ERK1_activation_Main_Pathway	0,084899	-0,05694	-0,03793	-0,10243	-0,17158	-0,42081	0,309786	0,376958	0,178564	-0,27589	-0,00453	-0,31997	-0,15765	-0,19285	-0,2334	-0,65644	-0,25601	0,061823	0,588188	0,726788
reactome_Histamine_receptors_Main_Pathway	-0,09202	0,029784	0,032379	0,083464	1,261815	0,71129	-0,59444	-0,89081	-0,25068	0,941689	-0,51569	0,947631	0,242115	-0,11693	0,032006	1,228687	1,455225	0,855624	0,588354	0,726788
ERK_Signaling_Pathway_EGFR_signaling	0,038768	0,151666	0,003391	-0,57761	-0,47682	-0,26006	-0,10303	-0,04044	-0,25171	-0,64443	0,592379	-0,07162	-0,37183	-0,27607	-0,45076	-1,01445	-1,16796	-0,50156	0,588763	0,726911
NCI_Degradation_of_beta_catenin_Main_Pathwa	0,319817	0,11254	-0,03519	-0,80404	-1,16917	-1,827	-0,75924	-0,70487	-1,20048	1,14288	0,271799	0,631653	-0,17535	0,226513	0,066899	-2,13073	-2,25681	-1,00548	0,589145	0,727095
KEGG_Acute_myeloid_leukemia_Main_Pathway	-0,70195	-0,7746	-0,96133	-0,3433	-0,4065	-0,42701	-1,40265	-0,9154	-2,24704	3,02203	0,607853	1,280801	-0,31439	-0,32401	0,353509	-3,92795	-2,58349	-1,67564	0,591707	0,729968
NCI_Signaling_events_mediated_by_PTP1B_Main	0,222073	-0,12914	-0,08981	-0,23094	-0,18758	-0,61975	-1,24409	-1,53492	-1,63624	1,450363	0,341945	-0,11309	0,506333	-0,10361	0,327007	-2,06414	-0,95579	-0,95637	0,592032	0,730079
dermatan_sulfate_biosynthesis	-0,16495	-0,1156	-0,1349	-0,48528	-0,63427	-0,31682	-0,19411	-0,18654	-0,56601	-0,8297	-0,78525	0,020735	0,22013	-0,12947	0,562778	0,648677	1,38094	0,254013	0,592452	0,730308
biocarta_control_of_skeletal_myogenesis_by_hda	-0,14363	-0,11365	-0,04491	1,27218	0,857224	0,618958	-0,43189	-0,46212	-0,2788	-0,97738	-0,34239	-0,53023	-0,2418	-0,66167	-0,42998	0,638652	0,563198	1,154911	0,595694	0,731848
biocarta_role_of_pi3k_subunit_p85_in_regulatior	0,102255	-0,21545	-0,17763	-1,52439	-2,17242	-1,03971	-1,48686	-1,20364	-2,19182	-1,5153	-0,25191	-0,28117	-0,37061	-0,121	-0,01803	-2,64518	-1,12298	0,59046	0,596342	0,731848
KEGG_Fc_epsilon_RI_signaling_Main_Pathway	0,120282	-0,31556	-0,44767	-0,87037	-0,97512	-2,78357	-1,02668	-0,89837	-1,67845	-0,83852	0,21305	0,644648	-0,22575	-0,53917	-0,10419	-5,94141	-3,40879	-2,30367	0,595051	0,731848
NCI_Stabilization_and_expansion_of_the_E_cadh	-0,02284	0,022053	0,01243	-0,36335	-0,12465	-0,19493	-0,37972	-0,53371	-0,47982	0,445497	1,078515	1,441922	-0,54381	0,0135	-0,05367	-1,44396	-0,94919	-0,86486	0,594967	0,731848
PPAR_Pathway_Adipocyte_Differentiation_Gluco	-0,11217	-0,03409	-0,06328	-1,21365	-0,90789	-0,59493	-0,4998	-0,47926	-0,7574	-0,42666	-0,12037	-0,73777	0,235449	0,151407	0,147823	-0,52835	-0,19495	0,323947	0,596755	0,731848
PPAR_Pathway_Fatty_Acid_Metabolism_Lipid_Hc	-0,11217	-0,03409	-0,06328	-1,21365	-0,90789	-0,59493	-0,4998	-0,47926	-0,7574	-0,42666	-0,12037	-0,73777	0,235449	0,151407	0,147823	-0,52835	-0,19495	0,323947	0,596755	0,731848
PPAR_Pathway_Peroxisome_Proliferation_Hepat	-0,11217	-0,03409	-0,06328	-1,21365	-0,90789	-0,59493	-0,4998	-0,47926	-0,7574	-0,42666	-0,12037	-0,73777	0,235449	0,151407	0,147823	-0,52835	-0,19495	0,323947	0,596755	0,731848
reactome_AUF1_hnRNP_D0_destabilizes_mRNA_	-0,05809	-0,18918	-0,07455	-0,74459	-0,64774	-0,03299	0,736293	0,767338	0,514654	-0,11118	0,57437	0,714834	-0,31454	-0,44046	-0,09898	-3,01234	-1,78043	-1,60796	0,595694	0,731848
reactome_G_alpha_z_signalling_events_Main_Pa	-0,48253	-0,03807	-0,27415	-0,34699	0,74233	-0,21202	-1,98812	-2,40412	-1,6037	-1,67064	1,298867	-0,44971	-0,13805	0,272347	0,233818	1,25063	1,110271	2,2401	0,595088	0,731848
reactome_Glucocorticoid_biosynthesis_Main_Pat	-0,46619	-0,31323	-0,08803	0,309593	0,107943	0,378483	0,364691	-0,38431	0,7792	-0,32758	-0,6421	-0,54746	0,0838	-0,23955	-0,18816	2,491162	1,402977	1,050727	0,596144	0,731848
reactome_MyD88_cascade_initiated_on_plasma_	0,072749	0,120764	-0,05649	0,625968	0,17481	-0,43325	-1,21365	-1,23446	-1,45017	0,807725	0,792297	1,014848	0,048954	0,069496	0,057597	-1,48884	-1,07976	-1,01365	0,595224	0,731848
reactome_Vitamin_D_calciferol_metabolism_Mai	-0,12021	0,013386	-0,01234	-0,90178	0,123749	0,833211	0,345054	0,094278	0,302127	0,989183	0,932163	0,480181	0,55888	0,099776	0,165991	-0,80854	-0,79209	-1,06522	0,595249	0,731848
reactome_XAV939_inhibits_tankyrase_stabilizing_	-0,16402	0,112043	0,018184	-0,53539	0,074445	0,624066	-0,01143	-0,08169	-0,20726	1,988395	0,851386	1,912982	0,035258	-0,28109	-0,44822	-0,86931	-1,11512	-1,34253	0,594833	0,731848
IL-10_Pathway	-0,46506	-0,51914	-0,55391	-1,43953	-2,02243	-1,36499	-0,38135	0,537288	-0,41515	2,751876	0,393037	2,355642	1,036187	0,502261	1,043667	-5,58911	-3,73516	-3,28847	0,598215	0,733349
NCI_amb2_Integrin_signaling_Pathway_(dendritic	-0,10322	-0,25283	-0,18097	0,459804	-0,19471	-0,04631	-0,75655	-0,89194	-0,1807	-0,8108	0,105535	-0,18359	0,632699	0,111276	0,669499	1,329784	0,526958	0,756613	0,59952	0,734082
NCI_amb2_Integrin_signaling_Pathway_(phagocy	-0,10322	-0,25283	-0,18097	0,459804	-0,19471	-0,04631	-0,75655	-0,89194	-0,1807	-0,8108	0,105535	-0,18359	0,632699	0,111276	0,669499	1,329784	0,526958	0,756613	0,59952	0,734082
superpathway_of_geranylgeranyldiphosphate_bic	0,119481	0,100859	0,005285	0,256494	0,285611	0,845046	1,430916	1,28988	1,253142	0,164926	1,573479	1,510718	-0,35154	-0,45725	-0,46418	-3,15878	-1,3719	-1,43069	0,599212	0,734082
biocarta_toll_like_receptor_Main_Pathway	0,000578	-0,19993	-0,3563	-0,14361	-0,38775	-1,17096	-1,8653	-1,31642	-2,77909	1,148704	2,284496	0,888183	-0,19984	-0,01709	0,06991	-1,19118	-1,54581	-1,51027	0,600129	0,734539
reactome_Assembly_of_the_RAD50_MRE11_NBS	-0,02719	-0,09797	-0,11876	-0,33289	0,174612	-0,05047	0,120886	0,221118	-0,09142	-0,13187	0,184518	0,206236	-0,13988	-0,08073	-0,1268	-0,69978	-0,39078	-0,31884	0,600754	0,735015
biocarta_trka_receptor_signaling_Main_Pathway	-0,07619	-0,29805	-0,19431	-1,0353	-0,03599	-0,61885	-0,81282	-0,92399	-0,88258	0,612222	0,581992	0,172387	0,499889	-0,13696	0,407426	-2,46304	-0,96213	-0,66264	0,601268	0,735067
pyrimidine_ribonucleosides_salvage_I	0,027957	-0,13295	-0,0864	-0,54125	0,083428	-0,70883	0,131422	0,184594	0,264344	-0,21441	-0,30348	-0,16112	0,010655	0,030763	-0,00344	-0,37435	-0,49232	-0,74268	0,601122	0,735067
NCI_Aurora_A_signaling_Pathway_(spindle_assen	0,010393	-0,02553	-0,11284	-0,40772	-1,09381	-0,27588	0,723694	0,77366	0,508713	-1,63525	-1,0715	-0,91282	-0,34179	-0,2825	-0,37031	-0,83142	-0,07549	-0,61058	0,603462	0,73746
KEGG_PI3K_Akt_signaling_Main_Pathway	0,903518	1,408698	0,755591	6,415484	3,120953	1,826702	8,985998	10,05889	7,824782	-4,68062	5,556302	5,138673	-1,36945	-2,18781	-1,16347	-4,85825	-1,9889	-1,0699	0,604434	0,738358
reactome_Methylation_Main_Pathway	0,177006	0,07942	0,05706	-1,27033	-0,36652	-0,07353	-0,17547	-0,21827	-0,33326	-0,17603	-0,30183	0,607188	-0,07372	0,116739	-0,41413	-1,10583	-0,73603	-0,45762	0,606002	0,739983
serine_biosynthesis_phosphorylated_route	0,10635	0,043867	-0,01706	-0,99295	-0,87853	-0,33853	0,88537	1,007443	0,737263	-0,84968	0,415628	-0,27751	-0,31842	-0,39517	-0,49435	-2,45627	-2,13322	-1,77656	0,607035	0,740954
reactome_Propionyl_CoA_catabolism_Main_Path	0,197085	0,153282	0,042013	-0,88839	-1,25624	-0,69842	0,077122	0,201654	-0,51224	-0,1286	0,416597	0,525404	-0,07544	-0,10379	0,176685	-3,02548	-2,46385	-2,53704	0,607789	0,741584
NCI_Signaling_events_mediated_by_HDAC_Class_	-0,10189	-0,02404	-0,10813	-0,16396	-0,46445	-0,80815	0,169584	0,121996	0,149004	-0,52442	-0,41794	-0,49036	-0,10209	-0,4047	-0,46095	-1,0722	-1,00876	-0,6289	0,608391	0,742028
KEGG_Estrogen_signaling_Main_Pathway	-0,67063	-1,40838	-1,25425	-1,49428	1,216415	-2,13454	-4,96283	-4,82801	-5,59107	-2,50643	-0,71173	3,893521	1,459349	1,462583	2,462428	-1,38453	2,039504	1,617455	0,609544	0,742987
reactome_Transferrin_endocytosis_and_recycling	-0,15407	-0,16245	0,056988	-1,89747	-3,61719	-1,35904	-2,80615	-2,52741	-3,42508	0,549733	-0,02397	-0,32687	-1,49501	-1,57666	-1,16968	-5,67071	-3,27664	-3,76335	0,609655	0,742987
reactome_Glucagon_like_Peptide_1_GLP1_regula	-0,23826	-0,26953	-0,06781	1,486627	1,522813	-0,09443	-1,49185	-1,91101	-1,50852	-3,65566	-0,02429	0,301606	-1,80678	-1,32216	-0,58501	-0,29847	-0,71805	0,455937	0,611982	0,745531
ornithine_ide_novo_i_biosynthesis	0,021305	0,038576	2,83E-05	0,051572	-0,274	0,038291	0,317695	0,346438	0,220845	0,3194	-0,11095	0,2538	-0,23859	-0,24823	-0,1216	-0,74603	-0,93301	-0,84082	0,612625	0,746023
NCI_Integrin_linked_kinase_signaling_Pathway_(a	-0,16813	-0,12788	-0,18112	0,279525	-0,01161	0,23963	-1,50354	-1,63746	-1,59857	0,571307	1,316377	0,784298	0,60695	0,520881	0,7095	-0,13021	0,062771	0,422598	0,613484	0,746213
NCI_Integrin_linked_kinase_signaling_Pathway_(a	-0,16813	-0,12788	-0,18112	0,279525	-0,01161	0,23963	-1,50354	-1,63746	-1,59857	0,571307	1,316377	0,784298	0,60695	0,520881	0,7095	-0,13021	0,062771	0,422598	0,613484	0,746213
reactome_Transport_of_vitamins_nucleosides_ar	0,047759	0,041881	-0,08647	0,830308	-0,53049	0,387082	-0,5206	-0,48844	-0,76684	0,147398	0,669818	0,771357	-0,12242	-0,30613	-0,14149	-0,87467	-0,91144	-1,03854	0,613499	0,746213
NCI_HIV_1_Nef_Negative_effector_of_Fas_and_T	0,197989	0,057376	0,075736	-0,41523	-0,18193	-0,3332	-2,03799	-1,67331	-2,41422	1,126003	-1,19702	1,07902	-0,2808	-0,40385	-0,46921	-3,52053	-2,27606	-1,12061	0,613913	0,746424
KEGG_Choline_metabolism_in_cancer_Main_Pat	-0,38013	-0,83727	-0,85264	-1,22574	-1,36514	-2,66288	-1,74523	-1,02986	-3,18278	1,127822	-1,04017	2,448732	-0,38275	0,044546	0,500631	-6,97075	-3,58576	-2,31353	0,614391	0,746714
reactome_Activation_of_BMF_and_translocation_	0,03816	-0,03733	0,031562	0,12168	0,065031	0,009952	0,510299	0,481818	0,428289	-0,21732	0,0715	0,365355	0,176773	0,029741	0,209588	0,093436	0,392862	0,6059	0,61522	0,74743
iron-sulfur_cluster_biosynthesis	0,018233	0,044879	0,046721	-0,2951	-0,01356	0,109831	0,17018	0,199675	0,123228	0,387674	0,268449	0,268077	0,047097	-0,01233	0,086323	-0,10434	-0,27014	-0,31497	0,618945	0,748835
NCI_Netrin_mediated_signaling_events_Main_Pa	-0,16516	-0,62376	-0,48195	-2,27116	-1,56762	-2,15897	-4,16173	-3,62181	-4,61884	-1,0811	0,09414	-0,61821	0,964528	1,088004	1,104127	-2,5715	-1,79356	-0,5181	0,618334	0,748835
p53_Signaling_Pathway_Apoptosis	-0,12097	-0,25987	-0,3291	0,675005	0,240903	-0,03672	-0,76997	-0,20524	-1,52969	1,45799	2,156935	1,828928	0,911538	0,093616	0,491185	-1,57327	-0,96049	-0,79357	0,619261	0,748835
p53_Signaling_Pathway_Cancer	-0,12097	-0,25987	-0,3291	0,675005	0,240903	-0,03672	-0,76997	-0,20524	-1,52969	1,45799	2,156935	1,828928	0,911538	0,093616	0,491185	-1,57327	-0,96049	-0,79357	0,619261	0,748835
p53_Signaling_Pathway_Gene_Expression_Anti-A	-0,12097	-0,25987	-0,3291	0,675005	0,240903	-0,03672	-0,76997	-0,20524	-1,52969	1,45799	2,156935	1,828928	0,911538	0,093616	0,491185	-1,57327	-0,96049	-0,79357	0,619261	0,748835
p53_Signaling_Pathway_Gene_Expression_Cell_C	-0,12097	-0,25987	-0,3291	0,675005	0,240903	-0,03672	-0,76997	-0,20524	-1,52969	1,45799	2,156935	1,828928	0,911538	0,093616	0,491185	-1,57327	-0,96049	-0,79357	0,619261	0,748835
p53_Signaling_Pathway_Gene_Expression_Cell_F	-0,12097	-0,25987	-0,3291	0,675005	0,240903	-0,03672	-0,76997	-0,20524	-1,52969	1,45799	2,156935	1,828928	0,911538	0,093616	0,491185	-1,57327	-0,96049	-0,79357	0,619261	0,748835
p53_Signaling_Pathway_Gene_Expression_Cell_S	-0,12097	-0,25987	-0,3291	0,675005	0,240903	-0,03672	-0,76997	-0,20524	-1,52969	1,45799	2,156935	1,828928	0,911538	0,093616	0,491185	-1,57327	-0,96049	-0,79357	0,619261	0,748835
p53_Signaling_Pathway_Gene_Expression_DNA_I	-0,12097	-0,25987	-0,3291	0,675005	0,240903	-0,03672	-0,76997	-0,20524	-1,52969	1,45799	2,156935	1,828928	0,911538	0,093616	0,491185	-1,57327	-0,96049	-0,79357	0,619261	0,748835
p53_Signaling_Pathway_Gene_Expression_ECM_	-0,12097	-0,25987	-0,3291	0,675005	0,240903	-0,03672	-0,76997	-0,20524	-1,52969	1,45799	2,156935	1,828928	0,911538	0,093616	0,491185	-1,57327	-0,96049	-0,79357	0,619261	0,748835
p53_Signaling_Pathway_Gene_Expression_Infecti	-0,12097	-0,25987	-0,3291	0,675005	0,240903	-0,03672	-0,76997	-0,20524	-1,52969	1,45799	2,156935	1,828928	0,911538	0,093616	0,491185	-1,57327	-0,96049	-0,79357	0,619261	0,748835
p53_Signaling_Pathway_Normal_Cell_Cycle_Prog	-0,12097	-0,25987	-0,3291	0,675005	0,240903	-0,03672	-0,76997	-0,20524	-1,52969	1,45799	2,156935	1,828928	0,911538	0,093616	0,491185	-1,57327	-0,96049	-0,79357	0,619261	0,748835
biocarta_igf_1_signaling_Main_Pathway	0,081745	-0,1099	-0,03551	-0,24891	-1,17102	-1,12514	-1,88762	-1,82302	-1,92951	1,449183	0,163455	0,533335	0,227117	0,138767	0,291861	-1,86318	-1,03586	-0,12658	0,620733	0,749873
bupropion_degradation	-0,22725	0,341009	-0,03943	-0,59861	-0,05733	0,270805	0,054206	-0,13491	0,174936	-0,6212	-0,02832	0,167719	-0,31758	0,309383	-0,15952	0,593361	0,345999	-0,47191	0,621306	0,749873
NCI_Angiopoietin_receptor_Tie2_mediated_signa	0,126555	0,02643	-0,03029	-0,61686	0,4029	0,253471	0,124611	0,087487	-0,02781	-0,10579	0,402222	0,15015	0,01664	0,06247	-0,02559	-0,66151	-0,26027	-0,31607	0,621365	0,749873
NCI_Signaling_events_mediated_by_HDAC_Class_	-0,01501	-0,04324	-0,08943	-0,83259	-0,13196	-0,37609	-0,90687	-0,78106	-1,08019	0,151723	-0,73731	0,602405	0,351627	-0,11988	0,291343	-0,53315	-1,04024	-0,65473	0,621025	0,749873
reactome_Gamma_carboxylation_of_protein_pre	-0,1029	-0,02214	0,057169	-0,99251	-0,1723	0,884897	-0,1628	-0,53607	0,340477	-1,24761	0,302466	0,866376	0,096148	-0,14699	0,263805	0,846238	0,687906	0,029835	0,621218	0,749873
reactome_Lysosome_Vesicle_Biogenesis_Main_P	-0,15957	-0,17057	-0,25617	-1,85685	-1,76032	-1,50496	-3,77928	-3,68393	-4,20299	-0,84786	-0,39357	1,079804	0,123561	-0,37562	0,601563	-1,8098	-1,52536	-0,71456	0,621564	0,749873
KEGG_Bile_secretion_Main_Pathway	-0,04951	0,056088	-0,00359	-0,60121	0,260221	-0,10856	0,199697	0,126927	0,300976	-0,34431	-0,29597	0,311321	0,179134	-0,19894	-0,1353	-0,30183	0,252301	0,155338	0,621928	0,750022
NCI_Visual_signal_transduction_Rods_Main_Path	0,0537	0,107962	0,05808	0,401493	1,21075	-0,32744	0,346178	-0,13122	0,87937	0,610781	0,612098	-0,2069	0,430313	0,013337	-0,11942	1,276829	0,025382	1,170902	0,625002	0,753436
NCI_ErbB1_downstream_signaling_Main_Pathwa	0,631368	0,14712	0,065424	-1,17105	-4,89524	-5,88666	1,021056	1,720683	-0,28347	-4,84118	-0,78003	-2,69191	-1,08336	-1,33775	-0,6615	-9,20137	-7,45673	-5,22215	0,626503	0,754955
FCGR3A-mediated_phagocytosis	0,123452	-0,32047	-0,21579	-1,14019	-1,34981	-0,03574	-0,17677	-0,51641	-0,20892	-1,03827	-0,87302	1,129178	-0,40414	-0,29906	0,12831	-2,20053	-1,97513	-1,17059	0,628616	0,756747
NCI_ErbB4_signaling_events_Pathway_(cell_proli	-0,12918	-0,00534	-0,05438	-1,54786	-1,06766	-1,83468	-0,11035	-0,06957	-0,31378	-0,50257	-0,12917	-0,07916	0,403666	0,218138	0,303317	-1,02859	-0,81913	-0,55792	0,628674	0,756747
reactome_SHC1_events_in_ERBB2_signaling_Mai	-0,11087	-0,21637	-0,14222	-1,4687	-1,05355	-1,96068	-0,12535	-0,10821	-0,44927	-0,82004	0,089199	-1,07408	0,257898	0,284016	0,284193	-2,30437	-1,80769	-1,34273	0,62872	0,756747
reactome_HDL_mediated_lipid_transport_Main_	-0,48609	0,117345	0,201087	0,29372	0,093602	0,374168	-1,51125	-1,86894	-0,93565	0,314767	-0,19539	-0,55529	0,781159	0,290891	0,446902	1,478986	0,903365	0,576357	0,629342	0,757204
biocarta_granzyme_a_mediated_apoptosis_Pathv	-0,15006	0,026963	0,21395	0,624463	0,773234	0,222188	-0,07884	-0,03025	0,501357	-0,33381	-0,07338	-0,04189	0,22008	0,203487	0,341278	0,868308	1,259078	0,866549	0,629813	0,757478
Development_of_Immune_Synapse	-1,19219	0,19307	-0,52738	0,822653	0,121287	1,901947	-4,31057	-5,19811	-2,41675	1,035908	2,60669	5,013015	-0,56288	-0,0715	0,79804	-4,97823	-1,17913	-0,86818	0,631374	0,759062
biocarta_atm_signaling_Pathway_(Pathway_prote	0,063316	-0,05724	-0,03537	0,352679	0,283079	0,185196	-0,25538	-0,03484	-0,37661	0,53346	0,791998	0,324583	-0,08712	-0,26299	-0,30675	-1,09471	-0,72244	-0,59974	0,632394	0,759288
KEGG_Lysosome_Main_Pathway	-0,26072	-0,81567	-0,18491	-6,72753	-7,17717	-7,58512	-13,6725	-12,4997	-16,2526	-0,23693	-0,15331	3,162606	-0,2209	-0,8067	2,810525	-17,1295	-16,2436	-11,7391	0,632537	0,759288
NCI_Plexin_D1_Signaling_Pathway_(endothelial_	-0,0366	-0,204	-0,19421	-0,71451	-1,17038	-0,34519	0,500958	0,508574	0,326376	-0,21859	-0,00034	-0,34327	0,497132	0,502308	0,492657	0,479707	0,680207	0,170551	0,632148	0,759288
reactome_L1CAM_interactions_Main_Pathway	-0,14755	-0,27037	-0,25975	-0,37425	-0,48211	-0,05053	-0,34197	-0,16486	-0,68787	-0,22271	-1,30032	-0,96798	0,167482	0,366364	0,806757	1,564441	1,970484	2,43823	0,632411	0,759288
NCI_Glypican_1_network_Main_Pathway	-0,37168	0,083386	-0,42749	-1,37851	-2,2462	-1,84847	-3,37006	-3,59445	-3,37601	1,921494	-0,38732	-1,08291	0,348007	0,721759	0,686515	-1,2736	-0,48897	-0,73246	0,634561	0,761188
reactome_activated_TAK1_mediates_p38_MAPK_	0,075652	-0,19273	-0,23964	-0,31997	-0,13144	-0,54312	0,257204	0,357891	0,100897	0,746484	1,093075	0,75603	0,345656	0,413473	0,492571	-0,27123	-0,10953	-0,35969	0,634609	0,761188
Akt_Signaling_Pathway_Splicing_Regulation	-0,01036	-0,06515	-0,05249	0,048582	0,319241	-0,08904	-0,19425	-0,22173	-0,42724	0,248612	0,716443	0,029435	-0,19419	-0,07012	-0,18546	-0,34402	-0,15202	-0,07418	0,636923	0,76367
ethanol_degradation_IV	0,163077	0,100466	0,004052	-0,45362	-0,27125	-0,13975	0,24922	0,463226	0,130241	0,679079	0,012996	0,97398	0,252878	0,31762	0,389839	-0,95345	-1,16984	-0,91205	0,637359	0,763899
NCI_IL2_signaling_events_mediated_by_PI3K_Pat	0,032444	0,266721	0,038576	-1,51132	0,408819	-0,88735	-0,17425	-0,43271	-0,33262	0,444528	0,207798	0,26183	0,29214	-0,03843	0,302991	-2,76029	-1,54882	-1,20469	0,639288	0,765917

ATM_Pathway_G2-Mitosis_progression	-0,14143	-0,1532	-0,14744	1,517868	1,030313	0,389133	-1,75301	-1,6652	-1,61431	2,414585	1,348778	1,456244	0,049684	0,144801	0,479996	0,888822	1,18879	0,194081	0,641086	0,767185
ATM_Pathway_G2_M_Checkpoint_Arrest	-0,14143	-0,1532	-0,14744	1,517868	1,030313	0,389133	-1,75301	-1,6652	-1,61431	2,414585	1,348778	1,456244	0,049684	0,144801	0,479996	0,888822	1,18879	0,194081	0,641086	0,767185
creatine-phosphate_biosynthesis	-0,07592	-0,04037	-0,00182	-0,79679	-0,9846	0,00175	1,158539	1,046613	1,088639	1,047636	0,363681	0,947942	0,341329	-0,04658	-0,13726	-0,89121	-0,93529	-1,35544	0,640727	0,767185
KEGG_Folate_biosynthesis_Main_Pathway	-0,03504	0,154461	0,001925	-0,39116	-0,6948	-0,19589	-0,15407	-0,04538	-0,16594	-0,22541	1,060093	0,872169	-0,16413	0,592612	0,127774	-0,71903	-0,46505	-0,56951	0,644074	0,770465
NCI_Reelin_signaling_Pathway_(neuron_migratio	0,071471	-0,01266	-0,04667	-0,02703	-0,38355	-0,49669	-1,42522	-1,14206	-1,2431	0,019856	-0,30748	0,027564	0,123789	0,103357	0,362692	-0,09542	0,170662	0,328978	0,644752	0,77098
NCI_LPA4_mediated_signaling_events_Main_Path	-0,29152	-0,02049	-0,06641	0,071972	0,32204	-0,34585	-0,99557	-0,83255	-0,77553	-0,58322	-1,13041	-0,26843	0,181562	0,862068	0,749897	2,54674	2,405011	2,368286	0,647548	0,773433
NCI_LPA4_mediated_signaling_events_Pathway_(	-0,29152	-0,02049	-0,06641	0,071972	0,32204	-0,34585	-0,99557	-0,83255	-0,77553	-0,58322	-1,13041	-0,26843	0,181562	0,862068	0,749897	2,54674	2,405011	2,368286	0,647548	0,773433
reactome_Collagen_degradation_Main_Pathway	-0,42963	-0,43309	0,165241	1,3589	0,203122	-0,08237	-0,2299	-0,52498	1,146786	1,635829	-1,1508	0,385073	0,530806	0,215097	0,930539	2,152457	2,677391	1,847025	0,647091	0,773433
NCI_Alpha_synuclein_signaling_Pathway_(Pathwa	-0,1394	-0,09969	0,087681	0,100565	1,288571	0,24604	-2,21618	-2,42447	-2,08687	-0,49115	-0,1388	0,380054	-0,17229	0,145773	0,566616	1,384877	-0,09648	1,130243	0,64929	0,774622
NCI_Alpha_synuclein_signaling_Pathway_(Pathwa	-0,1394	-0,09969	0,087681	0,100565	1,288571	0,24604	-2,21618	-2,42447	-2,08687	-0,49115	-0,1388	0,380054	-0,17229	0,145773	0,566616	1,384877	-0,09648	1,130243	0,64929	0,774622
NCI_Alpha_synuclein_signaling_Pathway_(protein	-0,1394	-0,09969	0,087681	0,100565	1,288571	0,24604	-2,21618	-2,42447	-2,08687	-0,49115	-0,1388	0,380054	-0,17229	0,145773	0,566616	1,384877	-0,09648	1,130243	0,64929	0,774622
Akt_Signaling_Pathway_Blocks_Apoptosis	0,042467	-0,2004	0,307535	-1,03957	0,427406	-1,05887	0,151723	0,187766	0,203401	-0,23171	0,754639	-0,76517	0,581636	0,43284	0,278976	-0,50113	0,742826	0,849065	0,650165	0,775281
biocarta_how_progesterone_initiates_the_oocyte	0,185359	0,215507	0,103343	-0,50505	-0,15329	-0,94379	0,751147	1,009617	0,660383	-0,37713	-1,50695	-0,8694	0,678072	0,420938	0,070425	0,668497	1,51382	1,07293	0,65034	0,775281
NCI_Regulation_of_p38_alpha_and_p38_beta_Ma	0,490508	0,071219	0,27767	-1,68837	0,496169	-1,12172	-1,72385	-1,5651	-2,17653	0,089978	0,577388	-0,12536	0,119159	0,512155	0,392995	-0,54741	-1,02556	0,039872	0,653629	0,778309
NCI_Regulation_of_p38_alpha_and_p38_beta_Pa	0,490508	0,071219	0,27767	-1,68837	0,496169	-1,12172	-1,72385	-1,5651	-2,17653	0,089978	0,577388	-0,12536	0,119159	0,512155	0,392995	-0,54741	-1,02556	0,039872	0,653629	0,778309
reactome_Interleukin_6_signaling_Main_Pathway	-0,01719	-0,14862	-0,13891	-0,09244	-0,2938	-0,48087	0,107618	0,212395	-0,01123	0,22528	0,135397	0,10915	-0,40559	-0,0637	0,013285	-1,05984	-0,34274	-0,11872	0,653495	0,778309
reactome_RNA_Polymerase_I_Promoter_Opening	-0,16023	-0,09018	0,257994	0,143084	1,05729	2,116324	-4,6993	-5,20299	-4,00013	-2,43427	-1,17302	-0,56502	0,850852	1,530515	-0,26564	4,245818	2,190188	2,642373	0,654412	0,778943
PAK_Pathway_Paxillin_Disassembly	-0,00595	-0,00091	-0,0675	-0,32016	-0,09011	-0,10058	-0,38866	-0,42866	-0,37471	-0,09233	0,201579	-0,14361	0,17678	0,103616	0,161831	0,115181	0,128023	0,133647	0,654895	0,77922
KEGG_GnRH_signaling_Main_Pathway	-0,29051	-0,60498	-0,55173	-0,99549	-0,31802	-1,9822	-3,70691	-3,21992	-3,97602	-1,97988	-0,07063	0,209252	0,117852	1,181146	1,743826	0,875195	2,231674	2,904072	0,655506	0,77965
biocarta_inactivation_of_gsk3_by_akt_causes_acc	0,150445	-0,08444	0,021749	0,192948	0,640465	0,673415	-0,20874	-0,09905	-0,34908	0,929104	0,027778	-0,60835	-0,01173	-0,25085	-0,02587	0,197131	0,145632	0,217505	0,658711	0,78131
Glucocorticoid_Receptor_Signaling_Pathway_Cell	0,015086	-0,02246	0,000653	-0,18793	0,010497	-0,01472	0,148924	0,139977	0,110387	0,205251	0,460311	0,477355	0,145552	-0,01083	0,163797	-0,46513	-0,48264	-0,61816	0,658514	0,78131
NCI_Class_I_PI3K_signaling_events_Main_Pathwa	-0,16977	-0,2842	-0,34276	-0,60022	0,955766	0,805914	-0,21727	-0,73844	-0,12168	0,89424	0,181102	0,182325	-1,16496	0,172593	0,535147	-0,90404	-0,85382	0,701162	0,657708	0,78131
NCI_E_cadherin_signaling_in_keratinocytes_Path	-0,08746	0,00102	-0,03453	-1,10548	0,302682	-0,87929	-0,31122	-0,30242	-0,52452	0,431666	1,344513	0,858801	-0,33138	-0,16461	0,160898	-3,64749	-2,13259	-2,2252	0,657582	0,78131
NCI_Stabilization_and_expansion_of_the_E_cadh	0,042034	0,038893	0,081915	-0,26173	0,15063	-0,09565	-0,30554	-0,37779	-0,44533	0,919836	1,079115	1,324073	-0,53035	-0,09676	-0,09094	-1,456	-0,95935	-1,04645	0,65916	0,78131
NCI_Stabilization_and_expansion_of_the_E_cadh	0,042034	0,038893	0,081915	-0,26173	0,15063	-0,09565	-0,30554	-0,37779	-0,44533	0,919836	1,079115	1,324073	-0,53035	-0,09676	-0,09094	-1,456	-0,95935	-1,04645	0,65916	0,78131
NCI_Stabilization_and_expansion_of_the_E_cadh	0,042034	0,038893	0,081915	-0,26173	0,15063	-0,09565	-0,30554	-0,37779	-0,44533	0,919836	1,079115	1,324073	-0,53035	-0,09676	-0,09094	-1,456	-0,95935	-1,04645	0,65916	0,78131
RALA_Signaling	-0,17663	-0,09612	-0,3622	-0,61489	-1,65138	-0,45326	-2,00643	-1,88484	-1,88028	-1,09322	-0,27721	0,07469	0,36014	-0,18902	0,034731	-1,19089	-0,93426	-1,06835	0,65898	0,78131
reactome_Ras_activation_uopn_Ca2_infux_throu	-0,02905	-0,19704	-0,01545	1,772709	2,742755	1,883849	-1,85605	-1,78315	-1,4899	0,934039	1,181812	-0,13223	-0,00733	0,182231	0,043282	3,847313	4,046399	3,444712	0,657799	0,78131
reactome_Golgi_Associated_Vesicle_Biogenesis_I	0,490626	-0,55548	-0,65726	-6,16462	-5,65341	-6,07449	-6,17903	-5,12281	-7,94645	-2,27531	-0,27778	-0,13359	0,808434	0,45965	1,962898	-11,5718	-8,22799	-7,64524	0,659704	0,781658
NCI_EGFR_dependent_Endothelin_signaling_ever	0,10803	-0,10589	0,050345	0,15601	-0,24635	-1,25095	-0,75048	-0,6392	-0,7412	0,104541	0,378275	-0,03673	0,260091	-0,13539	0,293258	-1,36341	-1,09572	-0,68895	0,660098	0,781827
NCI_Signaling_mediated_by_p38_gamma_and_p3	0,113967	0,008823	0,160216	1,309145	0,067557	0,15895	1,360954	1,489119	1,500493	0,055126	0,298236	-0,59784	0,948876	0,413268	0,545319	1,306968	1,569818	1,016212	0,660805	0,782367
KEGG_Circadian_entrainment_Main_Pathway	-0,63463	-0,02027	-0,46619	0,56178	2,064485	2,666342	-0,84748	-0,99003	-0,77201	-0,19494	-1,65156	-1,89703	-0,04406	0,627807	0,10747	6,339836	7,072296	5,720112	0,661084	0,7824
Akt_Signaling_Pathway_Neuroprotection	-0,01089	-0,09007	0,327066	-1,06167	0,458897	-0,80731	0,26046	0,221235	0,420016	-0,1756	1,202918	-0,5944	0,577724	0,497764	0,173458	-0,34869	0,230342	0,613337	0,664259	0,784667
cAMP_Pathway_Cell_Growth	-0,28274	-0,40278	-0,13324	0,276512	1,871633	-0,09296	-1,64566	-1,70334	-1,09533	-2,5057	-0,03058	0,102002	-0,68172	-0,54892	-0,31247	1,233788	-0,18208	0,894786	0,664254	0,784667
hypusine_biosynthesis	-0,07766	-0,09681	-0,07429	0,136979	-0,21727	-0,3788	-0,12866	-0,06963	-0,01233	0,65034	0,619679	0,247385	-0,20977	-0,24136	0,060474	-0,85588	-0,40151	-0,53628	0,663938	0,784667
reactome_Heme_degradation_Main_Pathway	0,1209	-0,03414	-0,00465	0,533306	-0,23641	-0,46479	-0,42178	-0,3023	-0,21197	-0,38963	-0,08351	-0,86482	-0,13294	0,080876	-0,23997	0,039112	-0,15266	-0,0223	0,663783	0,784667
tetrahydrobiopterin_ide_novoi_biosynthesis	-0,01607	0,147394	0,057656	-0,17147	0,086952	-0,27705	0,226381	0,369326	0,19737	-0,63331	-0,13861	0,164376	-0,35219	-0,31848	-0,32786	-0,80429	-0,78921	-0,64634	0,663924	0,784667
KEGG_Signaling_pathways_regulating_pluripoten	-0,0055	-0,00333	-0,01942	-0,15758	-0,02478	-0,05418	-0,05148	-0,0556	-0,06812	0,177642	0,131609	0,131716	-0,07693	-0,01568	-0,01301	-0,54525	-0,30586	-0,44361	0,665268	0,784965
NCI_ErbB1_downstream_signaling_Pathway_(cyto	0,111326	-0,0297	-0,11013	-0,54516	-0,27783	-0,72715	0,047487	0,204876	-0,24314	-0,08118	-0,24947	0,182802	-0,09717	0,111649	-0,05542	-1,36123	-1,06298	-0,6764	0,665078	0,784965
reactome_Activation_of_RAS_in_B_cells_Main_Pa	0,076115	0,146972	0,10154	-0,82742	-0,06003	0,090373	-0,02013	0,055937	-0,45296	-0,16358	0,215684	-0,19371	-0,1359	0,223594	-0,03615	-0,34356	-0,19099	-0,10431	0,665112	0,784965
GDP-L-fucose_biosynthesis_I_from_GDP-D-mann	0,053111	0,009863	-0,05041	-0,09333	-0,06623	-0,03119	-0,19107	-0,10537	-0,20912	-0,14039	0,173225	0,456497	0,287346	0,1864	0,249065	0,0924	0,16263	0,426278	0,6665	0,785824
JNK_Pathway_Insulin_Signaling	0,513047	0,801891	0,473553	-0,36818	0,866123	0,24712	0,866352	0,905288	0,771186	-1,67109	0,32716	-0,76008	-0,015	0,215741	-0,25098	0,768216	0,561657	1,457457	0,666332	0,785824
cysteine_biosynthesishomocysteine_degradation_	-0,00524	0,099065	0,060662	-0,05257	0,106041	-0,11204	0,039667	-0,04712	0,096783	0,124149	0,092248	0,19006	0,127156	-0,11806	0,15367	0,00968	-0,20834	0,045876	0,6681	0,786275
hydrogen_sulfide_biosynthesis_trans-sulfuration	-0,00524	0,099065	0,060662	-0,05257	0,106041	-0,11204	0,039667	-0,04712	0,096783	0,124149	0,092248	0,19006	0,127156	-0,11806	0,15367	0,00968	-0,20834	0,045876	0,6681	0,786275
reactome_S6K1_signalling_Main_Pathway	0,158078	-0,04144	0,008077	0,196989	-0,5491	-0,18307	0,525058	0,623131	0,229109	0,218667	0,90094	0,822012	-0,1003	-0,13639	0,043416	-1,55046	-1,66439	-1,32158	0,667143	0,786275
spermine_and_spermidine_degradation_I	-0,17675	0,000529	-0,06505	-0,2967	-0,04651	-0,21895	-2,31755	-2,46347	-2,18519	0,48735	0,863341	0,436085	0,245014	0,229922	0,066855	-0,56284	-0,33461	0,012556	0,668107	0,786275
thymine_degradation	-0,18604	-0,04558	0,025133	0,233882	0,059214	-0,40632	-0,35181	-0,42879	-0,19603	0,533587	0,223052	0,44001	-0,41732	-0,09978	-0,29765	-0,04572	-0,52279	-1,04449	0,668397	0,786275
uracil_degradation	-0,18604	-0,04558	0,025133	0,233882	0,059214	-0,40632	-0,35181	-0,42879	-0,19603	0,533587	0,223052	0,44001	-0,41732	-0,09978	-0,29765	-0,04572	-0,52279	-1,04449	0,668397	0,786275
NCI_ErbB2_ErbB3_signaling_events_Main_Pathw	0,37911	-0,1735	-0,25077	-1,14032	-0,99895	-1,74333	-0,49685	0,146172	-1,61032	0,820119	1,836081	2,623452	-0,23413	0,13365	0,702081	-7,05716	-4,315	-3,21649	0,669191	0,786912
NCI_Arf1_Main_Pathway	0,048163	0,001444	-0,08774	-0,64197	-0,5221	-0,76317	-0,52878	-0,24929	-1,0183	0,025151	0,773649	-0,03942	-0,11604	-0,16871	0,313182	-1,87898	-1,79418	-1,27178	0,672276	0,790242
reactome_CREB_phosphorylation_through_the_a	-0,08272	-0,13222	-0,02427	2,074062	2,835999	1,883905	-1,91919	-1,79291	-1,81087	0,548206	0,882649	-0,82618	0,232187	0,100113	0,182179	2,074652	2,958331	2,935554	0,672629	0,790359
imyoi-inositol_ide_novoi_biosynthesis	-0,06423	-0,25692	-0,06812	0,273616	-0,1857	0,256085	-0,56582	-0,65155	-0,63045	0,56971	-0,20504	0,187067	-0,03783	-0,0239	0,167985	0,499064	-0,02345	0,5576	0,672939	0,790424
NCI_Angiopoietin_receptor_Tie2_mediated_signa	0,237245	-0,43379	-0,58636	-3,57903	-1,84635	-2,95914	-3,39847	-3,6221	-4,33646	3,361427	0,962978	0,65818	1,640264	0,577309	1,80373	-3,97185	-1,66115	-0,52022	0,673283	0,790488
NCI_FGF_signaling_Pathway_(negative_regulation	-0,07922	0,103758	0,109137	0,426986	0,194936	0,972266	-0,30806	-0,39025	-0,21437	0,075438	0,548929	-0,00409	-0,10329	0,01302	-0,20968	0,277481	0,230623	-0,07149	0,673501	0,790488
KEGG_Phosphatidylinositol_signaling_system_Ma	-0,14138	-0,8693	-0,64088	-1,28375	-2,47155	-0,33724	0,817202	1,604585	-1,07539	-1,06156	-0,50748	-1,8291	-1,548	-0,48706	0,994871	-3,20337	-1,66864	-0,07451	0,674115	0,790693
NCI_Osteopontin_mediated_events_Pathway_(bo	-0,10126	-0,2443	-0,19139	-1,22752	-1,52152	-2,11627	-2,95916	-2,7175	-3,18526	0,304314	-0,67567	-1,64743	0,060393	0,27055	0,393286	-0,74799	-0,7957	-0,2417	0,674183	0,790693
KEGG_Dorso_ventral_axis_formation_Main_Path	0,126884	-0,03154	-0,01515	-0,13235	-0,49905	-0,1882	-0,42101	-0,33914	-0,68757	0,054157	0,277446	-0,19339	-0,22802	-0,13777	0,011514	-1,37614	-0,78757	-0,55313	0,675495	0,791934
Akt_Signaling_Pathway_Protein_Synthesis	0,200316	0,011308	0,327031	-0,09975	0,664729	-0,85991	0,03206	0,131229	-0,03281	0,606013	0,513182	-0,49646	0,81765	0,702581	0,450794	-0,31336	0,88029	2,223067	0,677417	0,793508
NCI_CDC42_signaling_events_Pathway_(cell_proli	0,034485	-0,02639	-0,02537	-0,03724	-0,16202	-0,05084	0,066141	0,099048	0,013085	-0,11786	-0,13597	-0,00374	-0,08341	-0,01999	-0,05121	-0,17626	-0,17357	-0,10917	0,677895	0,793508
NCI_CDC42_signaling_events_Pathway_(cytokines	0,034485	-0,02639	-0,02537	-0,03724	-0,16202	-0,05084	0,066141	0,099048	0,013085	-0,11786	-0,13597	-0,00374	-0,08341	-0,01999	-0,05121	-0,17626	-0,17357	-0,10917	0,677895	0,793508
Outside-in_Signaling_through_Integrins	-0,23377	-0,58142	-0,38712	-3,00515	-2,01073	-2,64539	-7,01447	-6,96242	-7,82835	-0,62844	1,384553	2,164952	0,353093	0,462549	1,153351	-6,46695	-4,41228	-3,9995	0,677345	0,793508
reactome_Recruitment_of_repair_and_signaling_	0,039249	-0,14497	-0,14141	9,51E-06	0,583977	0,01355	0,533412	0,69132	0,149381	0,073971	0,381228	0,315473	-0,16659	-0,43531	-0,24923	-0,88916	-0,4058	-0,34993	0,678112	0,793508
reactome_TRAF6_mediated_NF_kB_activation_M	0,161516	-0,13083	-0,02129	0,11933	-0,04574	-0,18625	-1,27105	-1,18107	-1,3987	2,331921	-0,28709	0,334443	0,281847	-0,77871	-0,01489	-1,8825	-2,05129	-1,17697	0,679247	0,794538
biocarta_bone_remodeling_Main_Pathway	-0,23778	-0,15848	-0,07622	0,687137	0,378092	0,828381	0,207685	-0,01314	0,26867	0,047168	-1,07096	-0,99578	-0,32878	-0,56353	-0,2154	1,988186	1,543415	1,884815	0,680008	0,795129
retinol_biosynthesis	0,232426	0,064468	0,021231	-0,62604	0,396306	0,827708	-0,30263	-0,48569	0,022451	0,150351	-0,0344	-1,37503	0,894417	0,185309	-0,15027	1,34993	2,07944	1,380432	0,680286	0,795156
biocarta_cystic_fibrosis_transmembrane_conduct	-0,48651	-0,14304	-0,35482	-0,46968	0,808622	0,328583	-0,95723	-1,13269	-0,60258	1,10712	1,141748	-0,33542	-0,88817	-0,38995	0,143955	-0,80335	-1,86307	-0,46358	0,681933	0,796783
KEGG_Herpes_simplex_infection_Main_Pathway	-0,12542	0,548605	0,095782	2,22517	-0,37237	0,207078	1,561692	1,55434	1,963256	-0,43663	1,08758	-0,57707	-1,11612	0,111723	-1,12093	-1,52799	2,14198	1,048687	0,683099	0,797845
Growth_Hormone_Signaling_Pathway_Protein_Sy	0,06594	0,011138	-0,07469	0,013084	0,18663	-0,04195	0,200907	0,377048	0,087968	0,553012	-0,15171	0,827705	0,848777	0,970598	0,777782	0,67932	0,4169	0,936612	0,685219	0,799722
reactome_Transport_and_synthesis_of_PAPS_Ma	0,040459	0,098981	0,065098	-0,07335	-0,8176	0,486342	-1,12685	-1,01743	-1,10337	0,366092	0,00788	0,009477	0,231874	0,18196	-0,1432	-0,50913	-0,88746	-0,22996	0,685123	0,799722
HGF_Pathway_Cell_Adhesion_and_Cell_Migration	-0,10006	-0,20466	-0,01002	-0,793	-0,41004	-0,57728	-4,86856	-4,84189	-4,63182	0,629992	1,039549	1,559827	0,090003	0,403849	0,362563	-0,83942	-1,04136	-1,03878	0,686342	0,800133
NCI_Cellular_roles_of_Anthrax_toxin_Pathway_(r	-0,289	-0,13968	-0,13078	-0,45108	-0,25234	-0,47108	-0,26925	-0,00868	-0,00438	0,968965	-0,13378	-0,15392	0,272741	0,547058	0,163476	-0,80552	-0,37877	-0,08935	0,686105	0,800133
NCI_Endothelins_Pathway_(arachidonic_acid_sec	-0,1358	-0,13062	-0,20786	-0,45502	-1,35171	-1,3906	0,268364	0,410597	0,157931	-1,07097	-0,79104	0,583013	-0,3539	-0,58762	-0,43481	-1,19749	-1,62958	-1,14664	0,686232	0,800133
reactome_Nef_mediated_downregulation_of_MH	-0,0799	-0,14445	-0,18239	-1,34684	0,091973	-0,82992	-1,99916	-1,79629	-2,22824	-0,28566	-0,19051	0,643412	0,293604	0,244326	0,682552	-0,94416	-0,77083	-0,67063	0,687335	0,800991
reactome_Synthesis_of_Ketone_Bodies_Main_Pa	0,05096	0,042718	0,077365	-0,05299	-0,29106	-0,26025	0,257767	0,12893	0,218093	-0,24144	-0,25273	0,583218	0,236515	-0,1919	0,239092	-0,21756	-0,72664	-0,3741	0,687864	0,801307
reactome_POU5F1_OCT4_SOX2_NANOG_repress	-0,00679	0,101538	-0,02956	-0,35896	0,114664	0,185167	0,210539	0,238089	0,182951	0,2801	0,338425	0,515298	-0,06265	0,021585	0,098347	-0,32294	-0,20213	-0,22057	0,68816	0,801352
chondroitin_sulfate_biosynthesis_late_stages	-0,01224	-0,28595	-0,13186	-0,84529	-0,39149	-1,54359	-1,78779	-2,1675	-1,82958	-0,21249	-1,37869	-0,69917	-0,33424	0,188642	0,853907	-1,03551	0,08089	-0,84449	0,68917	0,801359
NCI_a4b7_Integrin_signaling_Main_Pathway	-0,06144	-0,17018	-0,08384	-0,36228	0,299694	0,18848	-0,48228	-0,41816	-0,30709	0,485371	0,515727	0,544876	0,304598	0,017928	0,076219	-0,33333	-0,3257	-0,39116	0,688631	0,801359
NCI_a6b1_and_a6b4_Integrin_signaling_Pathway	0,013057	-0,18003	-0,07385	-0,60834	-1,26071	-1,66322	-0,93017	-0,84696	-1,14381	-0,20025	-0,06873	-0,70863	0,049084	-0,48096	-0,10452	-2,9868	-1,95016	-1,5944	0,689966	0,801359
NCI_a6b1_and_a6b4_Integrin_signaling_Pathway	0,013057	-0,18003	-0,07385	-0,60834	-1,26071	-1,66322	-0,93017	-0,84696	-1,14381	-0,20025	-0,06873	-0,70863	0,049084	-0,48096	-0,10452	-2,9868	-1,95016	-1,5944	0,689966	0,801359
NCI_a6b1_and_a6b4_Integrin_signaling_Pathway	0,013057	-0,18003	-0,07385	-0,60834	-1,26071	-1,66322	-0,93017	-0,84696	-1,14381	-0,20025	-0,06873	-0,70863	0,049084	-0,48096	-0,10452	-2,9868	-1,95016	-1,5944	0,689966	0,801359
NCI_RAC1_signaling_Pathway_(Pathway_positive_	0,047618	-0,03806	0,07004	-0,10764	0,019599	0,118782	-0,45687	-0,38193	-0,56786	-0,37149	-0,51407	0,020418	0,270375	0,280426	0,300016	0,785213	0,90572	1,185855	0,689412	0,801359
reactome_Fatty_Acids_bound_to_GPR40_FFAR1_	0,017544	-0,11565	-0,12987	0,193006	1,40185	0,729881	-0,36871	-0,48108	-0,40742	-0,21193	-0,18001	0,111891	-0,14153	-0,08903	0,146223	0,704771	0,881515	1,175828	0,688983	0,801359
reactome_Tie2_Signaling_Main_Pathway	0,033045	-0,28771	-0,30136	-1,85045	-0,53557	-1,4173	-2,03518	-2,17445	-2,00468	-0,42219	0,496809	-0,45764	0,550404	0,353268	0,62059	-2,93122	-1,08228	-0,4608	0,690957	0,802211
NCI_IL2_mediated_signaling_events_Main_Pathw	-0,10749	0,005537	-0,17166	-2,69457	-0,60757	-4,19743	-2,28364	-2,09796	-2,82257	1,184507	0,588006	0,466538	-0,29829	-0,34479	-0,12582	-4,29308	-3,5806	-2,96235	0,691304	0,802314
lysine_degradation_I_saccharopine_pathway	0,077887	0,051412	0,074482	-0,63167	-0,83945	-0,98434	0,789201	0,88538	0,492822	-0,35618	-0,11591	-0,74561	0,129506	-0,00816	0,216377	-1,58011	-1,03713	-0,59326	0,693732	0,80481
NCI_Class_IB_PI3K_non_lipid_kinase_events_Mai	0,014677	-0,00469	0,019553	0,637381	-0,48684	0,250892	-0,21616	-0,18199	-0,25494	0,05936	0,681069	-0,12346	-0,27341	0,18847	0,146891	-0,77219	0,019218	0,057446	0,69423	0,80481
NCI_Signaling_events_mediated_by_focal_adhesi	0,068584	0,030098	-0,12269	-1,29127	-0,57988	-2,01938	0,062725	0,277374	-0,2034	-0,78836	-0,94582	-0,26956	0,639655	0,202977	0,655259	-1,77442	-0,98025	-0,6144	0,694005	0,80481
NCI_Caspase_Cascade_in_Apoptosis_Pathway_(ap	0,022587	-0,0133	0,068509	-0,66387	-0,22565	-0,0878	-0,25638	-0,08753	-0,32742	0,386226	0,396397	-0,04277	0,057138	0,080234	-0,16096	-1,21152	-1,15136	-0,81164	0,695179	0,805311
reactome_RIP_mediated_NFkB_activation_via_ZB	-0,19158	-0,22916	-0,21065	-0,66993	-0,30999	-0,62116	-1,65046	-1,66976	-1,62679	2,769223	0,203866	1,839371	-0,03475	-0,78	-0,23516	-1,87992	-2,33669	-1,82689	0,695099	0,805311
biocarta_hiv_1_nef_negative_effector_of_fas_and	0,258924	-0,08831	0,040379	-0,51408	-0,05132	-0,93111	-2,08311	-1,76887	-2,31756	0,41344	-1,3641	1,066243	-0,71968	-0,47335	-0,74312	-2,3439	-1,48269	-0,26659	0,696797	0,806396
KEGG_Oocyte_meiosis_Main_Pathway	0,311256	0,495531	0,237033	-2,10518	-1,29919	-2,20767	0,295319	0,771241	-0,15272	0,565769	-0,4363	-1,49579	-0,91626	-1,56936	-1,10742	-6,40937	-6,44894	-4,29947	0,696396	0,806396
mTOR_Pathway_Cap-dependent_Translation	0,154388	0,130473	0,185726	-0,32292	-0,46605	-1,35353	0,388723	0,473112	0,035043	0,968993	0,877726	0,312827	0,603728	-0,16731	0,383374	-1,69503	-0,85577	-0,62255	0,696892	0,806396
reactome_IRS_related_events_triggered_by_IGF1	-0,15275	0,003249	-0,03466	1,268637	1,166299	-0,0581	-1,10529	-1,22893	-1,09364	0,664233	-0,36783	-0,267	0,23822	0,118672	0,053137	1,029673	0,804911	1,35104	0,697554	0,806863
Non-Junctional_Cell_Adhesion_in_Endothelium	-0,08039	-0,16593	-0,34536	-2,58503	-1,70634	-2,18916	-4,38423	-4,80971	-4,969	2,260448	1,860156	1,089632	0,171684	0,551038	1,182432	-5,59956	-4,85206	-5,4616	0,698152	0,807255
VEGF_Pathway_Nitric_Oxide_Production	0,145465	0,01551	0,061043	-0,41278	0,476526	0,373543	-0,01147	-0,13445	-0,10147	0,064295	0,398304	0,011234	-0,09245	0,02985	-0,16901	-0,01668	0,063679	0,090717	0,699009	0,807946
reactome_Cross_presentation_of_particulate_exo	-0,16101	0,113613	-0,08247	-0,16683	-0,40341	0,049418	-0,36759	-0,56211	-0,31631	-0,6143	-0,41851	-1,34027	-0,09393	-0,62878	-0,83885	0,128957	0,134282	-0,1349	0,699869	0,80864
KEGG_Cocaine_addiction_Main_Pathway	0,132022	0,227135	-0,03379	-2,7617	-2,33729	-3,32333	1,670752	2,097529	0,741324	-0,04677	-1,03404	0,918106	0,484419	0,588241	0,949246	-1,88035	-2,47207	-2,21241	0,700638	0,809228
NCI_Atypical_NF_kappaB_Pathway_(positive_regu	-0,23543	-0,11047	-0,13918	-0,95652	0,893951	-0,64479	-0,27188	-0,33302	-0,28146	-0,15419	-1,16547	0,821324	0,292548	-0,03131	0,281592	-0,023	-0,46185	0,058462	0,701	0,809346
reactome_Serine_biosynthesis_Main_Pathway	0,010274	-0,04244	-0,06202	-0,813	-0,5468	-0,30031	0,84924	0,891685	0,74047	-0,74934	0,228129	-0,18664	-0,32003	-0,54026	-0,38684	-1,76433	-1,44995	-1,26329	0,701638	0,809783
cAMP_Pathway_Cytokine_Production	-0,1099	-0,52931	-0,20582	0,79481	2,082135	0,132852	-2,06662	-2,04897	-1,35076	-1,79024	0,226429	-0,15904	-0,45907	-0,73109	-0,41666	1,669574	0,109783	0,990968	0,702157	0,810081
Erythropoietin_Pathway_Cell_Cycle_Progression	0,138075	0,061735	-0,01643	-0,27287	-0,34848	-0,49049	-0,51414	-0,3004	-0,83546	-0,69006	0,717796	0,458388	0,259714	0,31966	0,443136	-0,33501	-0,02744	0,315999	0,702497	0,810174
alpha-tocopherol_degradation	0,110961	0,164921	0,0384	0,277669	0,227663	-0,29169	-0,13442	-0,41691	-0,09481	0,312564	-0,14807	-0,0717	0,200699	0,389011	0,280197	0,055499	0,305017	0,444978	0,704498	0,81188
NCI_CDC42_signaling_events_Pathway_(RNA_spli	-0,2374	-0,19547	-0,31416	-0,17693	-0,26885	-0,67441	-1,06444	-0,90576	-1,07001	0,500862	0,710286	0,040304	0,417868	0,499782	0,514983	0,212811	0,402139	0,177637	0,704477	0,81188
3-phosphoinositide_degradation	0,198736	0,110766	0,074149	-0,47444	-0,95784	0,627882	-2,48906	-2,10548	-3,4492	-1,96224	-0,25567	-2,58364	-1,25137	-0,90174	-0,87113	-1,61759	-0,97068	-0,24958	0,706987	0,814168
KEGG_Glycerophospholipid_metabolism_Main_Pa	-0,46438	-1,01798	-0,92608	-0,23436	-3,17777	-3,00719	-0,31801	-0,13874	-0,60573	-0,87869	-1,65713	-4,3228	-0,25867	0,282993	0,848297	-1,23811	2,983811	2,740612	0,707492	0,814168
NCI_CDC42_signaling_events_Pathway_(establish	0,056152	-0,03018	0,005752	0,088369	-0,26537	-0,28381	0,097131	0,129906	0,021382	0,020442	-0,0622	-0,0229	0,028689	0,183644	0,145928	-0,15904	-0,10091	-0,17366	0,707529	0,814168
NCI_Thromboxane_A2_receptor_signaling_Pathw	-0,10058	-0,11598	-0,05146	-0,80773	-0,83928	-1,11077	-0,9876	-1,09027	-0,80048	-1,26471	-0,47453	-0,84185	-0,48041	-0,58752	-0,17583	-1,45199	-0,86832	-0,05307	0,70713	0,814168
NCI_N_cadherin_signaling_events_Pathway_(oste	-0,03287	-0,06854	-0,0768	0,174393	0,266418	-0,00588	-0,46587	-0,53953	-0,68124	0,4537	1,334448	0,639594	-0,35019	-0,00124	-0,09221	-1,25412	-1,20813	-0,85144	0,708194	0,814632
NCI_Urokinase_type_plasminogen_activator_uPA	-0,92359	-0,5049	-0,77205	-0,81233	-2,82738	-1,01787	-4,06274	-4,79209	-3,30264	0,810059	-1,67999	-0,50008	1,013704	-0,00157	0,799263	0,072318	1,419043	0,082046	0,710676	0,817185
NCI_TCR_signaling_in_naive_CD4_T_cells_Main_F	-0,43416	-0,41865	-0,27041	-0,01538	0,172818	0,13059	-2,14807	-2,34968	-2,37456	2,775832	1,18316	-2,15538	0,051305	0,468226	0,907321	0,875825	0,982282	1,774481	0,710962	0,817212
biocarta_signal_transduction_through_il1r_Main_	0,519407	-0,21925	-0,05953	1,161895	-0,19238	-0,28572	-2,22343	-1,80018	-2,81663	1,280675	0,717776	1,756536	0,229215	0,635166	0,491677	-0,68795	-0,53392	-1,01733	0,711325	0,817329
reactome_VEGFR2_mediated_vascular_permeabi	0,190452	-0,40476	0,113437	-1,91971	-0,08269	-0,67382	-1,58582	-1,51393	-2,40843	0,910443	1,32513	2,023652	0,279708	1,140723	0,431215	-2,13847	-1,07251	-0,14025	0,71202	0,817826
Angiotensin-PTK2B_Pathway	-0,0944	-0,28044	-0,33845	0,22029	0,346727	-0,04349	0,247018	0,318777	-0,03884	-0,94246	-0,45864	-1,54756	0,147331	0,205694	0,447729	1,103814	1,337246	1,893228	0,712584	0,818172
mTOR_Pathway	-5,37923	-3,60788	-3,08908	-6,8274	-8,19708	-11,5468	-18,1836	-20,6945	-14,9519	2,863065	-7,44712	-5,15774	6,358581	6,059313	6,3251	-1,88443	-1,66397	1,134112	0,71328	0,818669
reactome_Activation_of_Rac_Main_Pathway	0,117528	-0,15993	0,036188	-0,45942	0,363838	-0,81896	-3,21328	-2,7124	-3,38857	-0,23755	0,463017	-0,28105	0,14327	0,953931	0,597396	0,224652	0,371486	1,791936	0,715448	0,820854
biocarta_tnfr2_signaling_Main_Pathway	0,044862	0,100511	-0,03665	-0,44999	-0,25601	0,574634	-0,27599	-0,29755	-0,38958	0,774625	0,193976	0,460695	-0,12939	-0,03738	-0,08417	-0,75515	-0,81021	0,196415	0,716723	0,821088
biocarta_tnfr2_signaling_Pathway_(Pathway_pro	0,044862	0,100511	-0,03665	-0,44999	-0,25601	0,574634	-0,27599	-0,29755	-0,38958	0,774625	0,193976	0,460695	-0,12939	-0,03738	-0,08417	-0,75515	-0,81021	0,196415	0,716723	0,821088
biocarta_vegf_hypoxia_and_angiogenesis_Pathwa	-0,15401	-0,17083	-0,29494	-0,84601	0,335248	-0,5969	-1,55584	-1,69861	-1,3345	0,156263	-0,02569	-1,05787	1,025865	0,699604	0,616683	0,849065	0,700911	0,625065	0,719424	0,821088
CD40_Pathway_Gene_Expression_Cell_Adhesion_	0,03197	0,215123	0,038755	-0,47108	-1,50559	0,721141	0,790143	0,820357	0,646762	0,625141	-0,88613	-1,5588	0,182746	0,459468	0,538573	-0,02853	-0,21227	0,299072	0,719639	0,821088
CD40_Pathway_Gene_Expression_Cell_Survival_v	0,03197	0,215123	0,038755	-0,47108	-1,50559	0,721141	0,790143	0,820357	0,646762	0,625141	-0,88613	-1,5588	0,182746	0,459468	0,538573	-0,02853	-0,21227	0,299072	0,719639	0,821088
CD40_Pathway_Gene_Expression_Co-Stimulatory	0,03197	0,215123	0,038755	-0,47108	-1,50559	0,721141	0,790143	0,820357	0,646762	0,625141	-0,88613	-1,5588	0,182746	0,459468	0,538573	-0,02853	-0,21227	0,299072	0,719639	0,821088
CD40_Pathway_Gene_Expression_COX2_and_Pro	0,03197	0,215123	0,038755	-0,47108	-1,50559	0,721141	0,790143	0,820357	0,646762	0,625141	-0,88613	-1,5588	0,182746	0,459468	0,538573	-0,02853	-0,21227	0,299072	0,719639	0,821088
CD40_Pathway_Gene_Expression_Immunoglobul	0,03197	0,215123	0,038755	-0,47108	-1,50559	0,721141	0,790143	0,820357	0,646762	0,625141	-0,88613	-1,5588	0,182746	0,459468	0,538573	-0,02853	-0,21227	0,299072	0,719639	0,821088
CD40_Pathway_Gene_Expression_Pro-Inflamator	0,03197	0,215123	0,038755	-0,47108	-1,50559	0,721141	0,790143	0,820357	0,646762	0,625141	-0,88613	-1,5588	0,182746	0,459468	0,538573	-0,02853	-0,21227	0,299072	0,719639	0,821088
CD40_Pathway_Gene_Expression_Procoagulant_	0,03197	0,215123	0,038755	-0,47108	-1,50559	0,721141	0,790143	0,820357	0,646762	0,625141	-0,88613	-1,5588	0,182746	0,459468	0,538573	-0,02853	-0,21227	0,299072	0,719639	0,821088
EGF_Pathway_Actin_Cytoskeletal_Rearrangemen	-0,10416	-0,09905	-0,02765	-0,80825	-0,65399	-0,36997	0,099263	0,076999	-0,0848	2,114335	0,145608	0,628383	-0,27631	0,067147	-0,37995	-1,63361	-2,30435	-0,87283	0,72017	0,821088
NCI_Calcineurin_regulated_NFAT_dependent_tra	-0,31042	-0,50676	-0,5526	-0,00228	0,320334	-0,00674	0,0823	0,046973	-0,03323	0,129705	0,267382	0,608392	-0,19622	-0,11905	0,494088	0,06844	0,35889	0,100264	0,717133	0,821088
NCI_FoxO_family_signaling_Main_Pathway	-0,09323	-0,02281	-0,04524	-0,62367	-0,29133	-2,22423	-0,20021	-0,33777	0,03526	0,148995	0,445831	0,645469	0,041685	-0,23477	0,220525	-2,72148	-2,35813	-2,63605	0,716747	0,821088
Ras_Pathway_Receptor_Endocytosis	-0,2199	-0,19804	-0,31061	-1,03124	-0,14519	-0,64142	-0,5256	-0,61523	-0,67626	-0,26746	0,324698	0,022748	-0,14771	0,178688	-0,16356	-1,333	-0,87217	-0,88656	0,720325	0,821088
reactome_A_tetrasaccharide_linker_sequence_is_	-0,85514	-0,7157	-0,77187	-0,46563	-0,33331	0,125348	2,16867	2,097217	1,986028	-1,6074	-1,31012	-0,55104	1,152604	0,540055	1,048696	4,962438	3,613504	3,273486	0,720396	0,821088

reactome_Advanced_glycosylation_endproduct_r	-0,06974	-0,05943	-0,05495	0,318279	-0,60086	-0,28211	0,097499	-0,17751	0,151317	1,523405	0,800348	0,359734	-0,15601	-0,20837	-0,07643	-1,2743	-0,90417	-0,77731	0,718975	0,821088
reactome_AMPK_inhibits_chREBP_transcriptiona	-0,06028	-0,12002	-0,18852	-1,00629	-0,58701	-0,67239	-0,53218	-0,44092	-0,61761	-0,88871	-0,40013	-0,36349	0,142861	0,372195	0,148834	-0,44781	-0,32073	-0,43085	0,719845	0,821088
reactome_Insulin_receptor_recycling_Main_Path	0,038957	-0,11996	0,068761	-0,82335	-2,33212	-0,30715	-2,09303	-1,80947	-2,69838	0,412393	0,651492	-0,51464	-1,04329	-1,18084	-0,78339	-5,36808	-3,78257	-3,80107	0,716967	0,821088
NCI_a6b1_and_a6b4_Integrin_signaling_Main_Pa	-0,157	-0,51936	-0,30887	-2,52674	-2,71416	-2,54386	-5,11531	-5,09031	-5,25568	0,030414	0,653704	-1,46563	0,342358	-0,35605	-0,23213	-5,52628	-4,49512	-3,32726	0,72095	0,821309
reactome_Glucagon_signaling_in_metabolic_regu	-0,37873	-0,03712	-0,36089	1,320538	1,055535	-0,04981	-0,89746	-0,90782	-0,63108	-2,35705	0,102227	0,067775	-0,07414	0,614413	0,741208	1,916502	2,160693	2,44825	0,721118	0,821309
biocarta_gamma_aminobutyric_acid_receptor_lif	-0,26295	-0,08046	0,098219	0,218023	-1,69772	-0,3936	-2,8622	-2,77626	-2,91737	1,160271	1,015951	0,29421	1,724359	2,025195	2,325329	2,300205	1,936975	2,269111	0,72218	0,821637
KEGG_Prion_diseases_Main_Pathway	-0,1772	-0,32005	-0,39471	0,262783	-2,47439	-2,05171	-0,03694	0,332876	-0,71367	-1,20881	0,487001	0,772493	-0,53327	-0,45616	0,059348	-3,70576	-3,17922	-2,3798	0,722197	0,821637
reactome_Glycolysis_Main_Pathway	0,179548	0,075107	-0,03177	-0,894	-1,05239	-0,92521	0,196567	0,128939	0,037195	0,675501	0,914522	0,37744	0,038029	0,072455	0,092109	-2,73575	-3,0925	-2,24689	0,721776	0,821637
biocarta_adp_ribosylation_factor_Main_Pathway	0,342366	0,141153	-0,08452	0,264235	-0,49699	-1,60123	0,752157	1,280063	-0,01466	-0,70547	0,552808	0,181432	-0,86155	-0,44433	-0,52669	-4,74455	-1,68375	-1,49452	0,723206	0,822185
phosphatidylserine_biosynthesis_I	0,022513	0,020636	-0,00942	-0,07348	-0,07067	-0,25004	0,252051	0,262768	0,181557	-0,27626	-0,14062	-0,1288	0,014842	-0,03186	0,000765	-0,12262	-0,05214	-0,09409	0,723049	0,822185
NCI_FAS_CD95_signaling_Pathway_(necroptosis)	0,02568	0,077007	0,016692	-0,43776	-0,07619	0,111091	-0,51545	-0,39027	-0,47366	-0,31943	-0,21295	0,929218	-0,04458	-0,09436	-0,22207	-1,22395	-0,62247	-0,50395	0,724084	0,822883
NCI_mTOR_signaling_Pathway_(translational_init	0,06304	0,129779	-0,04068	-0,80599	-0,16323	-1,17513	-0,84858	-0,87938	-1,33065	0,72279	-0,59451	-1,4459	0,71991	0,158057	0,388583	-0,88282	-0,59813	0,12977	0,724392	0,822932
STATs_Pathway_Regulation_by_GH	-0,48867	-0,7729	-0,66868	-1,45308	-0,01751	-2,59032	-0,51043	-0,26102	-0,85204	-0,28828	-0,03963	1,822704	1,390517	1,081882	1,01701	0,660645	0,83686	0,438042	0,724876	0,823182
ErbB_Family_Pathway	-0,68005	-1,18405	-0,70723	-3,99501	-3,10337	-6,49706	-3,3504	-1,83611	-4,59729	0,976119	1,491541	-0,36243	1,718835	2,102147	3,348799	-8,14404	-5,73351	-1,59344	0,726112	0,824285
biocarta_fmlp_induced_chemokine_gene_expres	0,015834	0,023712	0,166557	-0,05253	0,149314	0,095703	-0,048	-0,23717	0,09301	-0,02687	-0,11804	-1,10921	-0,06833	0,097321	0,236028	0,839767	0,40603	1,207919	0,727328	0,824875
ceramide_ide_novoi_biosynthesis	-0,00382	-0,24344	-0,02205	-0,08023	0,012686	-0,62308	-0,46629	-0,36571	-0,63616	-0,16776	0,350446	0,304571	-0,17937	-0,21772	-0,11108	-0,94654	-0,92268	-0,75473	0,727074	0,824875
Smooth_Muscle_Tone_Regulation_by_G-protein_	-0,15166	-0,44308	-0,4381	0,644871	0,122215	-2,65985	-2,81529	-2,77845	-2,6499	0,991771	0,185085	-1,25507	1,070744	1,497024	1,739816	2,050609	1,922729	3,428133	0,727426	0,824875
biocarta_rho_cell_motility_signaling_Pathway_(fc	0,000382	0,105301	0,0446	-0,59083	-1,0145	-1,02479	0,138335	0,321118	0,135755	0,180718	0,531248	0,145567	-0,13276	0,025431	0,322979	-1,25487	-1,19487	-1,13347	0,728028	0,825258
KEGG_Collecting_duct_acid_secretion_Main_Path	-0,06617	-0,0848	0,203788	-0,89641	-2,06786	0,109084	-2,60848	-2,63484	-2,66785	0,037814	0,995173	-0,41138	-0,38974	-0,31319	0,238837	-3,2911	-2,01982	-2,19819	0,728446	0,825431
PPAR_Pathway	-0,98858	-0,55793	-0,45327	-0,96771	1,266576	0,569361	-4,00892	-3,36841	-3,31707	2,843563	1,380626	3,503514	0,338351	0,704179	1,458655	-1,08297	-1,11379	-0,69088	0,729013	0,825772
NCI_E_cadherin_signaling_in_keratinocytes_Main	-0,48524	-0,0652	-0,33472	-1,3599	-0,07798	-1,42214	-0,1437	-0,04162	-0,18379	0,383977	2,245058	-0,0216	-0,66391	-0,26747	-0,15465	-4,81444	-2,40677	-2,77297	0,72977	0,826329
NCI_Signaling_events_mediated_by_the_Hedgeh	-0,25091	-0,19791	-0,42223	-1,58455	0,940367	0,868365	-0,37206	-0,31658	-0,35183	1,541237	-0,35098	-0,97552	0,206115	-0,37044	-0,2414	-0,10447	-0,55365	-0,39125	0,730241	0,826563
cAMP_Pathway_Oncogenesis	-0,08089	0,095983	0,078622	0,33823	0,693452	0,325036	-0,88976	-0,77071	-0,84619	-1,3343	0,038143	0,33571	-1,20778	-0,70498	-0,42892	-0,76247	-0,58743	-0,36044	0,733309	0,828737
KEGG_Synthesis_and_degradation_of_ketone_bo	0,128581	-0,07716	0,010742	-0,48698	-0,85407	-0,82263	0,360288	0,370566	0,066549	-0,47683	0,729651	1,24045	0,376153	0,019835	0,274342	-2,19642	-1,54339	-1,42524	0,732984	0,828737
NCI_ErbB4_signaling_events_Pathway_(axon_gui	-0,09731	-0,02734	-0,00517	-1,383	-1,11557	-1,54596	-0,43818	-0,38673	-0,5451	-0,52284	-0,05211	0,078272	0,297799	0,216449	0,276556	-1,37497	-0,87819	-0,72195	0,733758	0,828737
NCI_ErbB4_signaling_events_Pathway_(heart_de	-0,09731	-0,02734	-0,00517	-1,383	-1,11557	-1,54596	-0,43818	-0,38673	-0,5451	-0,52284	-0,05211	0,078272	0,297799	0,216449	0,276556	-1,37497	-0,87819	-0,72195	0,733758	0,828737
NCI_S1P2_Pathway_(chemotaxis)	-0,06386	-0,00139	0,029866	0,131809	0,20976	-0,25902	-0,22063	-0,21359	0,044073	-1,06449	0,750567	0,573501	-0,078	-0,41938	-0,28841	0,114384	-0,06667	-0,66112	0,732475	0,828737
reactome_TWIK_related_potassium_channel_TRE	-0,04755	-0,00847	-0,00408	0,348333	0,86946	0,591153	-0,56697	-0,68245	-0,46906	-0,15829	0,465686	0,108022	-0,52033	-0,48119	-0,47754	-0,31671	-0,11615	-0,28188	0,733389	0,828737
NCI_IL2_mediated_signaling_events_Pathway_(na	0,004304	0,177106	-0,0382	-1,42313	-0,35734	-1,9232	-0,87467	-1,02976	-1,06858	-0,03955	0,829533	1,504752	0,373641	-0,10445	0,498032	-2,6149	-1,60423	-0,82548	0,734426	0,829191
reactome_GRB2_SOS_provides_linkage_to_MAPI	-0,29775	-0,24387	-0,13338	-0,74441	-0,27891	-0,21187	-0,43571	-0,53077	-0,5152	-0,62947	0,416613	-1,19847	-0,18477	-0,23555	-0,86767	-0,54791	-1,05697	-0,73501	0,73479	0,829301
reactome_Signalling_to_STAT3_Main_Pathway	-0,06805	-0,07886	-0,03323	-0,1445	0,004993	0,042381	-0,09276	-0,27684	0,040435	0,454325	0,023098	0,759847	0,203824	0,013969	0,150173	-0,22724	-0,03667	-0,02622	0,735491	0,829791
NCI_Integrins_in_angiogenesis_Main_Pathway	-0,63277	-0,98436	-0,50323	-4,80032	-3,45333	-4,69953	-6,04332	-5,97811	-5,75881	-0,091	-2,19983	-0,42643	1,284363	1,188226	1,437962	-2,24867	-1,51915	-0,80744	0,73581	0,829851
reactome_Regulation_of_HSF1_mediated_heat_s	-0,19898	-0,39821	-0,23516	-1,1867	-0,27012	0,26279	0,446434	0,777973	0,380995	1,547476	1,65372	0,923506	0,0836	-0,0634	0,110525	-2,57179	-2,66107	-1,43986	0,738335	0,832397
proline_degradation	-0,08116	-0,04555	-0,04238	-0,16216	-0,15151	-0,34132	-0,03807	0,012356	0,006883	-0,11334	-0,5937	0,37793	0,215745	0,398188	-0,02475	0,089913	0,562618	0,226872	0,739418	0,833316
cAMP_Pathway_Chemotaxis	-0,24351	-0,44838	-0,15113	0,5377	1,912932	0,034995	-1,67501	-1,75035	-1,08533	-1,83718	0,47182	0,520456	-0,43006	-0,39197	-0,11689	1,553615	0,03817	0,944577	0,740681	0,833835
NCI_Glypican_3_network_Main_Pathway	-0,07141	0,038	0,044213	-0,57729	0,409596	-0,46408	0,384093	0,431901	0,267654	0,23274	0,62272	-0,72629	0,362106	-0,22825	-0,16188	-0,19672	-0,21072	-0,08502	0,740212	0,833835
reactome_Amine_compound_SLC_transporters_I	0,159356	0,115364	0,163629	-0,96706	0,115482	-1,461	-0,29963	-0,60278	0,033077	-2,02611	-1,16514	-0,38293	0,71029	0,082549	0,484332	-0,3051	-0,12889	0,665002	0,740583	0,833835
SPP1_Pathway_in_Osteoclasts	-0,04729	-0,45837	-0,45202	-3,57659	-4,24408	-4,47736	-2,47457	-2,01752	-3,36777	-1,60018	-0,46565	-0,66607	0,122179	0,182146	0,969515	-7,69488	-5,59825	-3,74093	0,741276	0,834204
reactome_Platelet_sensitization_by_LDL_Main_P	0,021822	-0,08596	-0,14178	-0,50564	-1,16555	-1,47246	0,500484	0,414144	0,398519	-0,69051	2,169465	0,843394	-0,13892	0,078666	0,187539	-3,74521	-2,6599	-2,28877	0,742739	0,835548
reactome_Synthesis_of_PA_Main_Pathway	-0,10457	-0,09382	-0,14632	-0,95305	-1,66605	-2,35514	0,556279	0,815911	0,562952	-1,33422	-0,88145	0,418116	0,181214	-0,37422	0,44798	-2,47904	-2,38497	-1,79445	0,743425	0,836018
KEGG_beta_Alanine_metabolism_Main_Pathway	-0,47099	-0,07176	-0,32965	-0,5708	-1,38017	-1,3241	0,328671	-0,11003	0,910215	-0,03169	1,164004	1,207848	0,605765	1,016168	0,255168	-1,21424	-0,65793	-0,21037	0,74497	0,836849
reactome_Assembly_Of_The_HIV_Virion_Main_P	0,125846	0,014024	0,037694	-0,71888	-0,48315	-0,78461	0,746576	0,804156	0,603071	-0,08419	0,268247	0,473191	-0,1607	-0,14634	-0,05636	-2,33745	-1,77269	-1,34805	0,744522	0,836849
reactome_Type_II_Na_Pi_cotransporters_Main_F	-0,18332	0,020821	0,105642	0,168133	-0,06145	-0,05924	0,023131	0,07609	0,335366	-0,76267	-0,60777	-0,14764	0,015863	0,121924	-0,00703	1,203534	0,408903	0,89176	0,744719	0,836849
biocarta_phosphorylation_of_mek1_by_cdk5_p35	-0,30321	-0,56152	-0,40513	-0,84582	-0,19776	-1,23032	-1,56477	-1,47698	-1,58586	0,074188	1,10373	0,9193	0,22417	-0,70109	-0,15131	-1,63514	-1,58634	-0,81274	0,747347	0,839217
reactome_Facilitative_Na_independent_glucose_	0,00113	-0,03029	0,06143	-0,67799	0,389993	-0,23814	-1,72745	-1,80869	-1,44779	-1,62305	0,4236	-0,20708	-0,03505	-0,04605	0,476702	0,400887	0,136183	-0,14338	0,748563	0,840278
reactome_Processing_of_DNA_double_strand_br	-0,07345	-0,07483	-0,05611	0,049657	0,000548	0,078454	0,82757	0,989168	0,983224	1,025115	0,268934	0,485579	0,003975	0,141409	0,171824	-0,41714	-0,54547	-0,45232	0,749184	0,840673
biocarta_egf_signaling_Main_Pathway	-0,18204	-0,31458	-0,36657	-1,0842	-0,96661	-2,34126	-0,23392	-0,16867	-0,3736	0,836721	-0,13951	-0,32246	0,156761	-0,40624	0,292794	-3,30902	-2,55853	-1,92319	0,75056	0,841807
NCI_Signaling_events_mediated_by_Hepatocyte_	-0,08385	-0,27485	-0,25612	-1,56432	-1,3434	-1,06622	-1,69984	-1,58037	-1,88802	0,351277	0,616503	0,666057	0,359513	0,107604	0,143458	-3,13342	-2,38836	-2,28052	0,750735	0,841807
reactome_Synthesis_of_epoxy_EET_and_dihydro	-0,21726	-0,17981	0,031675	1,081493	0,940937	1,061	-0,74877	-0,90962	-0,41109	2,656515	1,183463	2,037351	-0,22069	0,324584	0,32872	1,220382	-0,66481	0,458095	0,752867	0,843894
lipoate_salvage	-0,03597	0,035822	-0,04159	0,013115	-0,10266	0,107089	-0,26345	-0,14369	-0,35552	0,066726	0,066834	0,053456	0,000161	-0,0995	0,088214	0,14402	0,06088	-0,19886	0,753899	0,844747
KEGG_Fc_gamma_R_mediated_phagocytosis_Ma	-0,65502	-1,01243	-0,57165	-0,94508	-3,65195	-3,37473	-1,03498	-0,70917	-1,38395	-0,4521	-1,05491	1,606354	-0,05852	1,211301	1,580599	-5,30287	-2,98631	-0,64079	0,755753	0,846519
reactome_Axonal_growth_inhibition_RHOA_activ	-0,13377	-0,01396	-0,01968	-0,36066	0,165289	0,845482	-0,8931	-1,08696	-0,85195	-0,49368	-0,26318	-0,58432	0,529027	0,464901	0,453312	1,414431	1,201702	1,154157	0,756136	0,846644
3-phosphoinositide_biosynthesis	-0,58881	-0,32081	-0,63262	-0,38093	-1,40755	-0,79118	1,861611	2,107157	1,790175	2,412401	0,088602	2,128979	-0,46423	0,01784	0,485799	-4,1654	-2,54676	-2,36346	0,757202	0,847228
reactome_FGFR3b_ligand_binding_and_activatio	-0,18442	-0,21347	-0,08061	-1,01215	-0,61602	-0,76747	-0,69546	-0,87525	-0,57112	0,216721	-0,66585	0,239576	0,429887	0,16305	0,238845	-0,25812	-0,3337	-0,29495	0,757146	0,847228
KEGG_Bladder_cancer_Main_Pathway	0,102017	-0,17058	-0,30144	-0,57697	-0,44323	-0,42235	-0,31585	0,051962	-0,83658	-0,48638	1,009541	-0,19305	-0,49037	-0,07205	-0,33841	-1,0968	-1,05889	-0,67613	0,757763	0,847552
biocarta_transcriptional_activation_of_dbpb_fror	-0,02238	-0,01373	-0,02358	-0,10555	-0,09114	-0,11729	0,085349	0,084555	0,117083	0,034545	0,010675	0,053479	0,01399	0,035347	0,035093	-0,23341	-0,18274	-0,09283	0,758661	0,847947
reactome_Clathrin_derived_vesicle_budding_Mai	0,044594	0,081366	-0,02315	0,08206	-0,3336	0,038905	-0,30822	-0,28113	-0,50802	0,0532	0,802116	0,852983	0,199229	-0,14726	0,259618	-0,58705	-0,39467	-0,15844	0,758403	0,847947
NCI_RAC1_signaling_Pathway_(regulation_of_cell	0,169681	-0,03584	0,00601	-0,28842	0,042316	-0,01137	0,217043	0,347654	-0,08455	0,056628	0,753788	0,194183	0,515569	0,904322	0,689117	0,527425	1,040284	0,709745	0,761236	0,85052
NCI_Lissencephaly_gene_LIS1_in_neuronal_migra	0,054349	-0,03779	0,024936	-0,18768	-0,3232	-0,32107	-0,13317	-0,11072	-0,29822	0,216316	0,215261	0,158112	0,121334	0,234139	0,189949	-0,64136	-0,48659	-0,34788	0,763478	0,852719
NCI_Arf6_trafficking_events_Pathway_(regulatior	0,096939	0,140267	0,186099	-0,08985	0,187275	-0,09832	-0,42419	-0,50107	-0,631	0,53083	0,974862	1,106187	-0,41078	-0,08218	0,105848	-1,96459	-1,3464	-1,35331	0,764885	0,853984
NCI_a6b1_and_a6b4_Integrin_signaling_Pathway	-0,02318	-0,01409	0,055382	-1,55E-06	0,291316	-0,1335	-0,10371	-0,09395	0,026613	-0,11265	-0,01171	-0,25477	0,081822	-0,01757	0,061079	0,277651	0,397635	0,367475	0,766791	0,855806
KEGG_Parkinson_s_disease_Main_Pathway	-0,10837	-0,28767	-0,17386	-0,12416	-0,83569	-0,26395	-0,53196	-0,3984	-0,64954	-0,4514	-0,33592	-0,57839	-0,06088	-0,007	0,126559	-0,07169	0,02075	-0,18205	0,768002	0,85685
Interferon_Pathway_Translation	0,073801	0,049913	-0,04478	-0,36825	-0,09907	-1,17584	0,724554	0,7476	0,482762	0,633076	0,187195	-0,37527	0,527842	-0,05343	0,346257	-1,32304	-0,7239	-0,56014	0,768842	0,857174
reactome_Nuclear_Receptor_transcription_Main_	-0,85416	-0,5417	-0,52646	-3,32316	-1,58513	-1,30376	-0,96117	-1,51766	-1,22459	-1,32861	0,289046	-0,10603	0,228142	-0,72938	0,836348	-1,3901	-1,12455	-1,48158	0,768782	0,857174
biocarta_influence_of_ras_and_rho_proteins_on_	0,146767	-0,10012	-0,00086	0,068202	0,560543	0,579336	-0,16898	-0,07424	-0,34393	0,860586	0,095828	-0,4915	0,003686	-0,21824	0,015904	0,165784	0,146635	0,212183	0,770097	0,858265
chondroitin_sulfate_biosynthesis	-0,25336	-0,34889	-0,37399	-0,70295	-0,57619	-0,78609	-0,54091	-0,84979	-0,59767	-0,50864	-1,56549	-1,04923	0,049793	0,089107	0,741825	0,390498	1,118755	-0,12538	0,772572	0,860716
reactome_POU5F1_OCT4_SOX2_NANOG_activate	-0,21524	-0,06947	-0,22593	0,026894	0,315555	0,704777	0,130478	-0,09026	0,291244	2,230652	0,113048	0,25444	-0,38706	-0,08232	0,176367	-1,02283	0,292435	-1,18491	0,772867	0,860737
KEGG_Prolactin_signaling_Main_Pathway	-0,24216	-0,43153	-0,77299	-1,74814	-2,11199	-2,29881	-1,93312	-1,67709	-2,86512	0,953509	1,083021	1,674187	0,049393	-0,71678	-0,00213	-7,48999	-4,42349	-3,49493	0,775868	0,863462
NCI_Urokinase_type_plasminogen_activator_uPA	-0,08202	-0,07057	-0,13595	0,004915	-0,44675	0,426548	-1,22081	-1,19789	-0,94012	-0,93699	-0,42789	-0,74467	-0,02891	-0,0217	0,083951	0,203077	0,859763	0,752569	0,775698	0,863462
NCI_CXCR3_mediated_signaling_events_Pathway	-0,17174	-0,11076	-0,0128	0,71396	0,224097	-0,71387	0,667061	0,946462	0,301277	1,721789	2,198138	0,129436	-0,80395	-0,34961	0,425948	-1,10489	-0,34533	-1,79843	0,778851	0,865882
NCI_IL2_signaling_events_mediated_by_PI3K_Pat	-0,08949	0,321329	0,069221	-1,5613	-0,10615	-1,26327	-0,21011	-0,57093	-0,19113	0,405502	0,37052	1,068995	0,693864	0,166625	0,537647	-1,84583	-0,79861	-0,55867	0,778877	0,865882
NCI_IL2_signaling_events_mediated_by_PI3K_Pat	-0,08949	0,321329	0,069221	-1,5613	-0,10615	-1,26327	-0,21011	-0,57093	-0,19113	0,405502	0,37052	1,068995	0,693864	0,166625	0,537647	-1,84583	-0,79861	-0,55867	0,778877	0,865882
Akt_Signaling_Pathway_Cell_Cycle	-0,00603	0,074159	0,009465	0,423964	0,3716	0,195509	-0,62221	-0,69195	-0,5973	0,129934	-0,13802	-0,13913	-0,40342	-0,32731	-0,2479	0,12929	0,301966	0,067444	0,782991	0,870145
biocarta_caspase_cascade_in_apoptosis_Main_Pa	-0,22087	0,060045	-0,18449	0,778711	-0,36042	0,038041	0,694112	0,561408	0,859735	0,539162	0,864395	1,884317	-0,19828	-0,50129	0,020509	-1,54455	-1,16425	-1,65828	0,783917	0,870261
KEGG_B_cell_receptor_signaling_Main_Pathway	-0,3352	-0,78253	-0,55029	-2,33804	1,047348	-0,72254	-0,55043	-0,30263	-1,59754	1,611441	-0,82628	2,344789	-0,53702	-2,07475	-0,53551	-3,25783	-2,37039	-1,62408	0,783622	0,870261
reactome_Golgi_Cisternae_Pericentriolar_Stack_I	0,078909	-0,00226	0,024611	-0,30703	-0,67866	-0,51093	-0,46358	-0,33932	-0,63376	0,018313	0,111623	-0,24725	0,016675	-0,07777	-0,03713	-1,30843	-0,89512	-0,69429	0,784213	0,870261
sorbitol_degradation_I	-0,01899	0,02965	0,001747	-0,35646	-0,14639	-0,39701	-0,11051	-0,10036	-0,09864	-0,33183	-0,2805	-0,23263	-0,00011	0,018512	0,043468	-0,02429	-0,06751	-0,09944	0,783972	0,870261
7-3-amino-3-carboxypropyl-wyosine_biosynthesis	0,042495	0,107864	-0,00709	-0,12522	-0,12684	-0,63854	0,191124	0,4133	-0,0022	0,321854	0,014549	0,350646	0,311461	0,122988	0,301852	-0,85503	-0,47396	-0,22167	0,784881	0,870383
reactome_Transcriptional_regulation_of_pluripot	-0,27072	-0,24605	-0,0327	-0,17938	0,031358	0,392706	0,297451	0,175309	0,627133	-0,64957	-0,30711	-0,9047	0,130264	-0,21887	0,542489	0,972154	1,115027	0,692339	0,784603	0,870383
NAD_salvage	0,028463	-0,01853	-0,06533	-0,70417	0,176965	-0,12432	-0,91071	-1,10183	-1,0768	0,260428	0,807511	0,648899	-0,1809	0,262839	-0,0849	-0,83798	-0,61005	-0,60832	0,785548	0,870813
NCI_PDGFR_beta_signaling_Pathway_(receptor_r	-0,06542	-0,09299	-0,19791	-0,34354	-1,35549	-0,64468	0,207321	0,126868	-0,05952	0,359031	-0,03017	0,439849	-0,15835	0,146526	0,575635	-2,45765	-1,20662	-0,42899	0,786545	0,871607
reactome_SMAD2_3_MH2_Domain_Mutants_in_	-0,10485	-0,08544	-0,10529	-0,39089	-0,7069	-0,22676	-0,70132	-0,75524	-0,93298	-0,53421	-0,15364	0,020774	-0,01729	0,130433	-0,02436	-0,59651	-0,54476	-0,11709	0,789042	0,873752
reactome_SMAD2_3_Phosphorylation_Motif_Mu	-0,10485	-0,08544	-0,10529	-0,39089	-0,7069	-0,22676	-0,70132	-0,75524	-0,93298	-0,53421	-0,15364	0,020774	-0,01729	0,130433	-0,02436	-0,59651	-0,54476	-0,11709	0,789042	0,873752
reactome_VEGFR2_mediated_cell_proliferation_I	0,024685	-0,22237	0,020503	0,022414	0,787338	-0,26763	-1,13658	-1,16242	-1,33192	1,353448	1,339581	-0,78112	0,256763	0,389433	0,151708	-0,90711	0,546369	-0,11723	0,789586	0,874045
biocarta_chrebp_regulation_by_carbohydrates_a	-0,57452	-0,14992	-0,11419	-3,02932	-0,54687	-1,71106	-1,91461	-2,40771	-1,34853	2,22607	1,288682	-1,92021	-0,05945	1,118593	0,59139	-1,46465	-1,96959	-0,85286	0,7908	0,875077
HA_syntesis_pathway	0,132692	-0,19166	-0,31891	-0,38443	-1,53376	-0,70792	1,489998	1,927283	0,774109	-2,25851	-0,66548	-1,41318	-0,07834	0,200785	0,489792	0,241128	0,928488	1,715043	0,792436	0,876576
L-cysteine_degradation_I	0,004702	0,048966	0,066817	-0,29364	-0,15734	-0,1948	-0,22136	-0,23934	-0,32152	-0,01422	-0,11315	-0,23852	-0,05636	-0,02371	0,0207	-0,22827	-0,41528	-0,35132	0,795296	0,879249
NCI_ErbB1_downstream_signaling_Pathway_(tigh	0,211777	0,153655	0,22279	0,180142	-0,14241	0,221893	1,357776	1,276495	1,319965	-0,15971	-0,79855	-0,3469	0,020852	-0,17828	-0,13529	0,277103	0,111889	-0,04737	0,795417	0,879249
NCI_Signaling_events_mediated_by_the_Hedgeh	-0,21999	-0,22406	-0,23594	-1,29502	0,374926	0,555693	-0,48589	-0,42742	-0,45272	0,476107	0,19178	-1,61239	0,346441	-0,30877	-0,27344	0,958965	0,427876	0,211884	0,797233	0,880944
reactome_Caspase_mediated_cleavage_of_cytosl	-0,05443	-0,0559	-0,10017	-0,42001	-0,75116	-0,51816	-0,05668	0,062726	-0,28558	0,072297	0,152463	-0,17574	0,615675	0,009563	0,314024	-0,4072	-0,58469	-0,52982	0,797772	0,881227
iSi-methyl-5-thioadenosine_degradation	0,017109	-0,01645	0,020692	0,320094	-0,06031	-0,03429	0,02282	0,003117	0,147942	-0,15807	0,041364	-0,11263	0,194239	0,046676	0,065481	0,258119	0,400126	0,151727	0,798341	0,881543
KEGG_Galactose_metabolism_Main_Pathway	-0,18231	0,145248	0,216765	-2,13667	-1,44335	-1,12437	0,779127	0,537664	0,893576	-2,66091	0,572736	0,765145	-0,3792	-0,32878	0,367442	-1,52947	-2,45608	-1,14892	0,804461	0,887986
KEGG_FoxO_signaling_Main_Pathway	0,246845	0,364073	0,334285	0,227425	-0,91498	-0,20759	-0,69257	-0,76773	0,866596	-1,75655	-0,00594	-1,726	0,441371	0,324034	-0,09013	2,414675	1,409636	-0,49436	0,805032	0,888302
Ras_Pathway_Increased_T-cell_Adhesion	-0,1523	-0,25277	0,026478	0,023839	0,10884	-0,12392	-4,4323	-4,40303	-4,14227	0,891884	0,859209	1,798244	0,416068	0,786249	0,70561	-0,12117	-0,37762	-0,46374	0,807516	0,890727
biocarta_srebp_control_of_lipid_synthesis_Main_	0,031673	-0,0763	-0,24017	-0,54276	0,152732	-0,45059	-0,78822	-0,79503	-0,9165	-0,58055	-0,39682	0,313815	-0,30247	-0,27564	-0,28494	-0,30178	-0,81511	-0,16483	0,810642	0,892653
biocarta_srebp_control_of_lipid_synthesis_Pathw	0,031673	-0,0763	-0,24017	-0,54276	0,152732	-0,45059	-0,78822	-0,79503	-0,9165	-0,58055	-0,39682	0,313815	-0,30247	-0,27564	-0,28494	-0,30178	-0,81511	-0,16483	0,810642	0,892653
cAMP_Pathway_eNOS_Signaling_Cardiovascular_	0,009669	0,009138	0,045503	-0,60416	0,724255	0,421156	-0,85829	-0,8237	-0,88568	-1,4606	0,423826	0,499652	-0,8577	-0,40925	-0,35761	-0,7815	-0,72483	-0,37206	0,810695	0,892653
NCI_E_cadherin_signaling_in_keratinocytes_Path	-0,02375	0,018107	0,018742	-1,0049	0,351004	-0,38283	-0,19201	-0,20922	-0,44427	0,82864	1,492763	1,037149	-0,47822	-0,0777	0,086957	-3,06956	-1,93037	-1,85428	0,810472	0,892653
reactome_FCERI_mediated_Ca_2_mobilization_M	0,111725	0,13666	0,184116	1,822939	0,770118	-0,47483	-0,32943	-0,91419	-0,48534	1,072145	1,074416	0,618802	-0,4108	-0,2366	0,615748	-0,07278	-0,37413	-0,54799	0,809735	0,892653
IGF1R_Signaling_Pathway_Glycogen_Synthesis	-0,01133	0,020682	-0,02477	0,840061	0,897618	0,589422	-0,20427	-0,26554	-0,48638	0,365904	0,690664	0,220338	-0,33139	-0,27358	-0,22183	-0,11017	0,29202	0,242284	0,812348	0,893861
reactome_Synthesis_of_PIPs_at_the_plasma_me	-0,3188	-0,49217	-0,34387	-1,83477	-3,05124	0,109138	0,157929	0,721988	-0,85297	-0,82643	-0,45091	-1,023	-1,23905	-0,14746	0,060689	-3,54896	-2,32713	-1,21192	0,812366	0,893861
reactome_WNT_ligand_biogenesis_and_traffickin	-1,09105	-0,62087	-0,54071	0,207892	-0,22881	-0,67733	-0,42416	-0,81237	0,91082	-0,00814	0,630769	0,91497	0,627001	1,660696	0,056924	0,934198	1,615709	1,639655	0,815219	0,896683
ethanol_degradation_II	0,209386	0,094804	0,04715	-0,40686	-0,25299	-0,29165	0,21165	0,366636	0,124104	0,879206	0,204922	1,013135	0,448273	0,636634	0,610439	-0,18173	-0,38208	-0,22128	0,816248	0,897498
reactome_PRC2_methylates_histones_and_DNA_	0,23479	-0,09415	-0,49984	-0,49897	-1,29473	-2,5658	6,495576	7,158977	5,729418	0,92551	0,44083	0,152155	-0,79209	-1,33049	0,168396	-5,94675	-3,17964	-3,33361	0,818724	0,899903
NCI_Signaling_events_mediated_by_Hepatocyte_	-0,34682	-0,35054	-0,55492	-0,92801	-1,01887	-0,36882	-1,19233	-1,16821	-1,33627	-0,69631	1,301925	1,311597	-0,02469	-0,05979	-0,04052	-1,95737	-0,84096	-0,70374	0,819707	0,900666
reactome_Common_Pathway_Main_Pathway	-0,31735	0,071847	-0,10212	-0,34479	-0,32312	0,888275	0,369929	-0,22897	1,261479	-1,53734	0,368806	-1,69759	0,315076	-1,09169	-0,21338	2,229427	0,587457	0,231898	0,820707	0,901128
reactome_Vitamin_B1_thiamin_metabolism_Mai	0,039026	-0,11404	-0,00217	-0,03994	0,166026	0,15254	-0,15889	0,121371	-0,38102	0,518956	0,148391	-0,62488	-0,39936	-0,28075	-0,31634	-0,28784	-0,23748	-0,87224	0,820701	0,901128
reactome_Trafficking_and_processing_of_endosc	-0,04606	0,046855	0,190308	-1,34685	-0,36364	-0,69449	-0,58573	-0,4661	-0,56927	1,74193	0,930755	0,053657	-0,05264	-0,08835	0,031255	-1,23662	-2,76459	-1,64337	0,822143	0,902387
NCI_Presenilin_action_in_Notch_and_Wnt_signal	0,249042	0,342398	0,073669	0,578186	0,426466	0,143638	-0,10206	0,046046	-0,46312	2,460843	0,379509	0,911243	-0,18131	0,330865	0,644032	-0,28003	-1,35757	0,886216	0,823019	0,902395
NCI_Signaling_events_mediated_by_PTP1B_Path	0,133986	-0,06016	-0,02416	-0,49381	-0,58795	0,084291	-0,08144	-0,18045	-0,21367	0,570661	0,0839	0,304865	0,256196	-0,08761	0,214303	-0,96887	-0,77973	-0,65277	0,822935	0,902395
NCI_Signaling_events_mediated_by_PTP1B_Path	0,133986	-0,06016	-0,02416	-0,49381	-0,58795	0,084291	-0,08144	-0,18045	-0,21367	0,570661	0,0839	0,304865	0,256196	-0,08761	0,214303	-0,96887	-0,77973	-0,65277	0,822935	0,902395
Cellular_Apoptosis_Pathway_Gene_Expression_F	-0,03495	0,072162	0,004074	-0,45581	-1,41836	0,309454	0,219578	0,291068	0,122737	0,990819	-0,86351	-1,18703	0,156655	0,394692	0,56218	-0,15537	-0,32863	0,069305	0,823967	0,903117
flavin_biosynthesis	-0,09046	-0,09838	-0,06436	-0,08613	-0,24002	0,117405	-0,30733	-0,31407	-0,40337	0,172052	0,202713	0,477453	-0,10195	0,051309	-0,02537	-0,46361	-0,41624	-0,3852	0,825632	0,904392
IGF1R_Signaling_Pathway_Cell_Migration	-0,13962	-0,24627	-0,03875	1,481553	1,077135	0,279714	-4,81002	-4,73085	-4,37755	3,766466	0,946871	3,738128	0,192122	0,377139	0,550661	-0,04141	-0,63693	-0,16653	0,825833	0,904392
reactome_p38MAPK_events_Main_Pathway	0,067379	-0,2425	-0,26651	-0,21254	0,165473	-0,47343	-1,03183	-0,88565	-0,92986	0,619034	0,737144	-0,11557	0,131226	-0,0698	-0,04233	-0,76042	-0,42503	-0,77538	0,826001	0,904392
reactome_Adenylate_cyclase_activating_Main_Pa	-0,336	-0,16886	-0,13943	-0,34818	0,230477	-0,32336	-1,22582	-1,02838	-1,05639	-1,31218	-1,53928	-0,63786	0,386491	0,715323	0,66737	2,70494	2,361146	2,227872	0,826808	0,904957
KEGG_Lysine_degradation_Main_Pathway	0,253386	-0,09907	0,063502	-0,81344	-0,38382	-0,91123	2,460305	2,723945	1,945904	-0,16148	0,851652	1,691741	-0,48716	-0,03296	-0,70428	-3,13501	-2,9367	-2,72775	0,831227	0,906926
KEGG_Melanoma_Main_Pathway	-0,92782	-1,04551	-0,69326	-3,73591	-1,79038	-2,85638	-5,1359	-5,33557	-4,74778	0,327242	-1,71596	0,884558	-0,54421	-0,49877	1,038645	-3,84512	-2,55143	-1,31695	0,830368	0,906926
NCI_ErbB4_signaling_events_Pathway_(apoptosis	-0,11866	-0,03071	-0,06564	-2,29772	-1,51279	-1,80108	-1,15243	-0,93233	-1,4377	-0,40428	0,258196	0,532125	0,464601	0,408701	0,531435	-3,34275	-2,61178	-1,91688	0,831141	0,906926
NCI_ErbB4_signaling_events_Pathway_(chemota	-0,11866	-0,03071	-0,06564	-2,29772	-1,51279	-1,80108	-1,15243	-0,93233	-1,4377	-0,40428	0,258196	0,532125	0,464601	0,408701	0,531435	-3,34275	-2,61178	-1,91688	0,831141	0,906926
NCI_ErbB4_signaling_events_Pathway_(dendrite_	-0,11866	-0,03071	-0,06564	-2,29772	-1,51279	-1,80108	-1,15243	-0,93233	-1,4377	-0,40428	0,258196	0,532125	0,464601	0,408701	0,531435	-3,34275	-2,61178	-1,91688	0,831141	0,906926
NCI_Signaling_events_mediated_by_the_Hedgeh	-0,08048	-0,14189	-0,15352	-0,19549	0,75279	0,270792	0,643338	0,593802	0,906111	0,454187	0,186474	-0,17328	0,284134	-0,20632	0,171569	0,654191	0,691757	0,556644	0,829956	0,906926
NCI_Signaling_events_mediated_by_the_Hedgeh	-0,08048	-0,14189	-0,15352	-0,19549	0,75279	0,270792	0,643338	0,593802	0,906111	0,454187	0,186474	-0,17328	0,284134	-0,20632	0,171569	0,654191	0,691757	0,556644	0,829956	0,906926
NCI_Signaling_events_mediated_by_the_Hedgeh	-0,08048	-0,14189	-0,15352	-0,19549	0,75279	0,270792	0,643338	0,593802	0,906111	0,454187	0,186474	-0,17328	0,284134	-0,20632	0,171569	0,654191	0,691757	0,556644	0,829956	0,906926
NCI_Signaling_events_mediated_by_the_Hedgeh	-0,08048	-0,14189	-0,15352	-0,19549	0,75279	0,270792	0,643338	0,593802	0,906111	0,454187	0,186474	-0,17328	0,284134	-0,20632	0,171569	0,654191	0,691757	0,556644	0,829956	0,906926
JAK-STAT_Pathway_IFN-Inducible_Gene_Expressi	1,263748	0,691978	0,842343	-2,02615	-0,41847	-3,69964	1,265183	1,231506	1,443085	0,839362	3,24694	3,159787	2,501933	3,223972	4,949021	0,350036	0,26458	-1,00423	0,832168	0,907548
reactome_Signalling_to_p38_via_RIT_and_RIN_M	0,076719	0,020457	0,008704	-0,13798	0,204622	0,662801	0,001449	-0,23368	-0,24271	0,259358	-0,22587	0,520411	-0,75206	-0,54354	-0,34169	-0,84015	-0,34821	-0,34545	0,832844	0,907548
reactome_Zinc_influx_into_cells_by_the_SLC39_g	0,131492	0,300433	0,255615	0,013031	-0,32727	-0,70147	-0,83161	-0,72976	-0,8205	-0,84843	-0,98388	-0,54031	0,199871	-0,14986	0,417596	-0,14798	0,177067	0,374278	0,832963	0,907548
TRH_Pathway	-0,72844	-1,10546	-1,11673	0,432353	1,154308	-0,11032	-5,92603	-5,68607	-5,6967	-3,56669	0,716353	2,446896	-1,26687	-0,79138	0,100873	-1,40483	0,509673	2,124752	0,832485	0,907548
NCI_Class_I_PI3K_signaling_events_mediated_by_	0,108629	0,003293	-0,07478	-0,82111	1,047783	0,2444	-1,64215	-1,43346	-2,29695	1,315506	0,843734	1,348525	0,36559	0,804599	0,373196	-0,39001	-0,41244	-0,40803	0,834009	0,90837

NCI_Urokinase_type_plasminogen_activator_uPA	-0,05898	0,015708	-0,12297	-0,09096	-0,64619	0,027092	-0,22214	-0,31212	0,154417	-0,99998	-0,19462	-0,48052	0,139544	0,16933	0,158066	0,477789	0,809448	0,627054	0,834425	0,908505
NCI_RAC1_signaling_Pathway_(actin_filament_pc	0,063279	0,00323	0,079434	-0,29189	-0,1616	-0,14298	-0,45484	-0,33054	-0,548	-0,74363	-0,73093	-0,25798	0,237356	0,311051	0,396073	0,362321	0,532263	0,808035	0,835014	0,908829
biocarta_downregulated_of_mta_3_in_er_negati	-0,01287	-0,01672	-0,00415	0,144158	0,343619	-0,00224	-0,04184	-0,07682	0,075717	-0,09447	0,140158	0,136113	-0,05499	-0,03189	0,021188	0,20608	0,099765	0,042187	0,836366	0,90903
mineralocorticoid_biosynthesis	-0,30813	-0,33934	-0,07046	0,345847	0,032754	0,522375	0,237727	-0,17295	0,309221	-1,05382	-0,49804	-0,54562	0,066077	-0,21254	0,136148	1,545793	1,123296	0,884823	0,836125	0,90903
NCI_FOXA1_transcription_factor_network_Main_	-0,01287	-0,01672	-0,00415	0,144158	0,343619	-0,00224	-0,04184	-0,07682	0,075717	-0,09447	0,140158	0,136113	-0,05499	-0,03189	0,021188	0,20608	0,099765	0,042187	0,836366	0,90903
reactome_Transport_of_glucose_and_other_suga	0,106645	0,109102	0,288001	-1,37176	-0,09122	-1,33401	0,332406	-0,05533	0,580357	-2,67049	-1,16973	-0,3464	1,149048	0,38912	0,501235	0,064829	0,338154	1,084986	0,836022	0,90903
NCI_p38_signaling_mediated_by_MAPKAP_kinase	0,024655	-0,17634	-0,1839	-0,29605	-0,17717	-0,14751	-0,23887	-0,14096	-0,27784	0,32183	0,456181	0,009466	-0,01222	-0,01342	0,104321	-0,60146	-0,39706	-0,5202	0,836828	0,909215
NCI_IL8_and_CXCR2_mediated_signaling_events_	-0,02964	-0,02284	-0,11888	-0,36359	-0,16641	-0,27816	-1,76235	-1,89262	-1,54059	-0,04419	0,371452	-0,89505	-0,31203	-0,55475	-0,16168	-1,79194	-0,29088	-0,35152	0,837809	0,909963
NCI_Signaling_events_mediated_by_HDAC_Class_	-0,07956	-0,07269	-0,18435	-0,43226	-0,60575	-1,04672	0,263993	0,154464	0,135992	-0,71537	-0,69025	-0,86877	-0,12454	-0,38712	-0,24509	-0,75673	-0,30201	-0,27325	0,838235	0,910108
NCI_Atypical_NF_kappaB_Pathway_(Pathway_de	-0,28735	-0,18178	-0,09944	-0,40678	1,274634	-0,75812	-0,71745	-0,81696	-0,6216	-0,18611	-1,66818	0,057686	0,241156	-0,13612	0,300758	1,18822	0,514605	0,700717	0,838684	0,910179
NCI_Thromboxane_A2_receptor_signaling_Pathw	-0,12316	-0,15843	0,110933	-0,95988	-1,20676	-1,32609	-0,80234	-1,09551	-0,51583	-1,44741	-0,91292	-1,15671	-0,18532	-0,27739	0,140201	-1,24995	-0,31118	0,129971	0,839088	0,910179
pentose_phosphate_pathway_non-oxidative_bra	0,214191	-0,01777	0,033068	-0,34373	-0,81581	-0,96738	-0,13608	-0,1085	-0,34028	0,087073	0,060713	0,025063	-0,35466	-0,13332	-0,28856	-1,94349	-0,96575	-1,30221	0,839177	0,910179
cAMP_Pathway_Glycolisis	-0,03227	-0,22095	0,218373	-0,71955	0,038358	-0,70171	-0,54385	-0,33621	-0,73112	0,176078	0,888172	0,734154	0,683032	-0,15012	0,610101	-1,3121	-0,55301	-0,90029	0,840405	0,911194
NCI_Signaling_events_mediated_by_Hepatocyte_	-0,00927	0,043068	-0,08874	-1,12177	-0,13768	-0,87652	-0,22343	-0,28548	-0,4956	0,373629	-0,18539	-1,10781	0,545802	0,15526	0,383749	-0,29304	0,04771	0,226648	0,84113	0,911663
mTOR_Pathway_Lipid_Synthesis	-0,25921	-0,19724	-0,27022	-1,36806	-0,31328	-0,08066	-8,01777	-7,98177	-8,5004	0,18094	-1,33199	0,338362	1,118002	0,248003	0,291632	-0,31609	-0,39259	1,338145	0,842691	0,913038
biocarta_atm_signaling_Pathway_(apoptosis)	-0,07535	-0,09075	-0,14862	0,393	0,164828	0,176677	0,016469	0,292849	-0,07842	0,555362	0,936377	0,336189	-0,0638	-0,29083	-0,31369	-0,84299	-0,56062	-0,40653	0,844379	0,914397
KEGG_Riboflavin_metabolism_Main_Pathway	-0,09601	-0,09268	-0,09028	0,129557	-0,46125	-0,08123	-0,22956	-0,26145	-0,27655	0,091668	0,335379	0,393468	-0,14635	0,070683	-0,06415	-0,43514	-0,69188	-0,51356	0,844533	0,914397
reactome_Signaling_by_Leptin_Main_Pathway	-0,21068	-0,29532	-0,14515	0,156739	-0,06404	-0,51706	-0,5152	-0,55641	-0,70291	1,513187	-0,3267	0,119315	-0,0282	-0,15947	0,227561	-0,86442	-0,43132	-0,37929	0,84486	0,914433
WNT_Pathway	-3,68536	-3,28683	-3,09148	4,613817	6,618059	3,024167	-2,46078	-2,43005	0,434518	-4,5336	-0,69366	-3,70242	1,24081	1,851925	2,520866	14,84183	11,75973	16,64233	0,845946	0,915291
reactome_HDACs_deacetylate_histones_Main_Pa	0,321038	-0,15716	-0,40994	0,460577	-0,74765	-1,54811	4,367265	5,242562	3,749942	3,640896	2,300217	0,562169	-1,2378	-1,1368	-0,13063	-4,83045	-2,99579	-3,22619	0,846529	0,915603
glutathione_redox_reactions_II	-0,05037	-0,04542	-0,05594	-0,32114	-0,23029	-0,28803	0,023644	0,043899	0,008563	-0,30464	-0,14186	0,210358	-0,02103	0,063805	0,00304	-0,4151	-0,10196	-0,12673	0,847413	0,916241
KEGG_Cytosolic_DNA_sensing_Main_Pathway	0,158783	-0,42405	-0,18999	-0,52017	0,469468	0,0983	-1,20154	-0,88366	-1,27598	0,283276	-1,37978	-0,40943	-0,09088	-0,50975	0,162964	0,981199	0,168468	0,725112	0,847724	0,916259
NCI_p38_signaling_mediated_by_MAPKAP_kinase	-0,0116	-0,14408	-0,16649	-0,37725	0,010501	-0,02836	-0,20274	-0,15245	-0,16659	0,320681	0,468678	0,193411	0,115222	-0,02685	0,151774	-0,40664	-0,30879	-0,40747	0,848394	0,916665
reactome_APOBEC3G_mediated_resistance_to_H	-0,1483	-0,16509	-0,09864	-0,64767	-0,4255	-0,75165	0,204753	0,306585	0,162155	0,046949	0,151836	-0,34689	-0,03958	-0,16611	0,019723	-0,75019	-0,83729	-0,56275	0,849935	0,917694
reactome_Integration_of_provirus_Main_Pathwa	-0,1483	-0,16509	-0,09864	-0,64767	-0,4255	-0,75165	0,204753	0,306585	0,162155	0,046949	0,151836	-0,34689	-0,03958	-0,16611	0,019723	-0,75019	-0,83729	-0,56275	0,849935	0,917694
biocarta_bioactive_peptide_induced_signaling_M	-0,15787	-0,35406	-0,44284	-0,08041	-1,02778	0,174959	-0,08818	0,062287	-0,51167	0,965296	1,206316	-1,40354	0,125953	0,246197	0,789047	-0,49233	-0,2169	0,613083	0,85251	0,918432
biocarta_regulation_of_eif_4e_and_p70s6_kinase	-0,06669	-0,38165	-0,23521	-0,12045	-0,5726	-0,64682	0,022233	-0,04457	-0,31093	0,940377	0,526378	0,387621	1,64937	1,917852	1,930284	1,463134	0,880471	1,731186	0,8527	0,918432
mevalonate_pathway	0,014319	0,108854	0,013176	0,133268	0,333908	0,603594	0,906654	0,774459	0,936874	0,265034	1,345779	1,428372	-0,28195	-0,31611	-0,48173	-1,54158	-0,45509	-0,58651	0,852135	0,918432
NCI_E_cadherin_signaling_in_the_nascent_adher	0,025417	0,055876	0,086533	-0,14635	0,123969	-0,03431	-0,48798	-0,51758	-0,56601	1,099188	1,34299	1,30521	-0,60064	-0,22157	-0,14083	-1,87616	-1,38751	-1,45158	0,852501	0,918432
pregnenolone_biosynthesis	-0,01932	-0,01661	-0,00562	-0,07817	-0,30578	-0,11611	-0,02379	0,018367	0,133079	-0,1206	-0,38661	-0,19584	0,029252	0,037679	0,110689	0,241887	-0,14017	0,015947	0,852058	0,918432
reactome_Bile_salt_and_organic_anion_SLC_tran	-0,05271	-0,00626	0,124372	-0,4047	-0,2067	0,126992	0,632032	0,547453	0,54728	-0,64438	-0,00459	0,036535	0,438759	0,306571	0,016903	0,369924	0,467043	0,419984	0,852144	0,918432
reactome_GPVI_mediated_activation_cascade_M	-0,71801	-0,29286	-0,58294	-0,3948	-0,18668	-0,89811	-0,35052	-0,84892	-0,45008	0,829182	0,636195	1,367317	-0,68816	-0,1201	-0,24471	-2,85905	-1,93494	-1,04662	0,852666	0,918432
reactome_Spry_regulation_of_FGF_signaling_Ma	0,08333	-0,02744	-0,0676	-0,16824	-0,17559	-0,43722	-0,30983	-0,33545	-0,40037	0,565636	0,487008	0,846083	0,094606	0,105596	0,305687	-1,51442	-0,81823	-0,42572	0,852978	0,918432
anandamide_degradation	-0,08931	-0,11441	0,040674	0,034271	0,052184	-0,3886	0,220988	0,258454	0,397757	0,047317	-0,73651	-0,47914	-0,12444	-0,19182	0,112597	0,357048	0,0711	0,387536	0,85342	0,918591
reactome_Nicotinate_metabolism_Main_Pathwa	0,012948	-0,06745	-0,01256	-0,85694	0,951447	0,684824	-0,60489	-0,91879	-0,75683	0,288486	-0,44736	-0,10522	0,188674	0,617978	-0,15362	0,713926	0,83246	0,41398	0,854657	0,919605
reactome_Fc_epsilon_receptor_FCERI_signaling_	0,127386	0,437516	0,307599	0,016717	0,186479	-0,01994	-0,04607	-0,32391	-0,05326	-0,39872	-0,6638	-0,72508	-0,49101	-0,5091	-0,29675	0,01306	-0,23776	-0,22932	0,855268	0,919945
biocarta_no2_dependent_il_12_pathway_in_nk_	0,122843	-0,19151	0,116637	-0,82368	-0,04673	-0,12251	-0,13493	-0,23674	0,014226	-1,06249	-1,48715	-0,52651	0,09563	0,479636	0,977169	1,219229	1,459041	0,512581	0,856554	0,92101
NCI_E_cadherin_signaling_in_the_nascent_adher	0,021708	0,008808	0,059203	-0,08162	0,185619	0,142222	-0,7118	-0,77928	-0,91265	1,205602	1,188868	1,304007	-0,49803	-0,08496	-0,02109	-2,08547	-1,56428	-1,40673	0,857302	0,921496
NCI_Signaling_events_mediated_by_Hepatocyte_	-0,00029	-0,01992	-0,00412	-0,16914	0,416538	0,135763	-0,28281	-0,37431	-0,49492	0,49189	0,854449	0,569802	0,194117	-0,01366	0,097	-0,72059	-0,2356	-0,24228	0,857869	0,921787
biocarta_multi_step_regulation_of_transcription_	0,062687	-0,07315	-0,22532	-1,4345	-0,52553	-0,75043	-1,37758	-1,44574	-1,93225	0,731264	1,316553	1,498922	0,795237	0,681673	0,941006	-1,62261	-2,35067	-0,72504	0,860125	0,92248
biocarta_regulation_of_splicing_through_sam68_	-0,05223	-0,26625	-0,26959	-0,09454	-0,16385	-1,08618	-0,46652	-0,22013	-0,58553	-0,89391	0,64283	0,653215	-0,34374	-0,46202	-0,20177	-1,81169	-1,31437	-0,72818	0,859409	0,92248
biocarta_stress_induction_of_hsp_regulation_Ma	-0,23029	-0,41629	-0,20261	0,779981	0,719384	0,696582	-0,26831	-0,48718	0,213689	-0,5926	1,064571	0,978919	0,749336	0,449168	0,437363	1,797971	1,611521	1,217339	0,860116	0,92248
ErbB_Family_Pathway_Anti-Apoptosis	-0,00127	0,070457	-0,00592	-0,0203	0,580891	0,196458	-0,45747	-0,62704	-0,67795	0,647546	0,409956	0,171168	-0,08927	-0,05138	-0,03111	-0,20193	0,123254	-0,11245	0,859742	0,92248
NCI_Signaling_events_mediated_by_the_Hedgeh	-0,02007	-0,00705	-0,10142	0,065988	0,046029	0,620293	-0,00818	-0,01183	0,09322	0,699443	0,415416	0,028802	0,11893	0,055238	0,210236	0,367078	-0,03465	0,119833	0,85941	0,92248
reactome_Mitochondrial_iron_sulfur_cluster_bio	0,031717	-0,17911	-0,0358	-0,12227	-0,13393	-0,1517	-0,26947	-0,26602	-0,43243	0,396582	0,725469	0,67141	0,06372	-0,2018	-0,03972	-1,0624	-0,69074	-1,11853	0,86029	0,92248
KEGG_Staphylococcus_aureus_infection_Main_Pa	0,019861	0,305216	-0,0134	2,995639	2,365149	2,297129	-1,73183	-1,64947	-0,92015	0,615197	-1,30871	1,209053	-0,59965	0,258781	-0,991	3,051586	1,723864	2,543905	0,861873	0,922591
NADH_repair	-0,00481	-0,04186	0,005591	-0,18301	-0,3075	-0,20593	-0,11128	-0,11287	-0,10905	0,429339	0,072798	0,300454	0,014461	-0,02584	-0,03146	-0,65309	-0,57169	-0,42697	0,861231	0,922591
NCI_Calcium_signaling_in_the_CD4_TCR_Main_Pa	-0,25855	-0,40522	-0,5415	-0,04859	0,027952	-0,5066	-0,55072	-0,63941	-0,731	0,393019	0,930937	1,248895	-0,35299	0,036474	0,657103	-0,41686	-0,35838	-0,42851	0,86161	0,922591
NCI_Downstream_signaling_in_naive_CD8_T_cell	0,052177	0,204755	0,007616	0,071627	-0,12719	-0,22746	0,042058	-0,10231	-0,07634	0,509453	-0,33109	0,11021	-0,0707	-0,13663	-0,15968	-0,26712	-0,61804	-0,44227	0,862467	0,922591
reactome_regulation_of_FZD_by_ubiquitination_	0,290473	-0,26384	0,076364	-0,76596	-0,4679	-1,29602	1,830994	1,841739	1,840529	-1,11391	-0,43486	-1,19485	-0,40616	0,277511	0,056549	-0,96729	-0,14462	0,336318	0,86178	0,922591
thyroid_hormone_metabolism_I_via_deiodinatio	-0,02543	-0,03064	0,042007	-0,35295	-0,1471	-0,59455	0,017946	-0,01854	0,236143	0,836237	0,591147	-0,009	0,36237	0,022205	-0,06937	-0,65987	-0,82003	-0,72327	0,862394	0,922591
thyronamine_and_iodothyronamine_metabolism	-0,02543	-0,03064	0,042007	-0,35295	-0,1471	-0,59455	0,017946	-0,01854	0,236143	0,836237	0,591147	-0,009	0,36237	0,022205	-0,06937	-0,65987	-0,82003	-0,72327	0,862394	0,922591
NCI_Signaling_events_mediated_by_Stem_cell_fa	-0,04767	-0,01317	-0,01956	-0,67178	-0,7556	-0,76721	-1,2754	-1,2068	-1,44584	0,328748	0,393421	0,413747	-0,03144	0,003681	-0,03984	-1,58094	-2,03109	-1,39327	0,864944	0,924723
reactome_Metabolism_of_folate_and_pterines_	0,214381	-0,02987	-0,10593	-0,26699	-0,28669	-0,74452	0,972677	1,162522	0,860921	0,070564	0,329813	-0,39386	-0,02192	-0,22608	0,029309	-0,70056	-1,05703	-0,3122	0,865054	0,924723
biocarta_cell_to_cell_adhesion_signaling_Main_P	-0,20696	-0,16182	-0,28248	-0,35244	0,440899	-0,87919	-0,82389	-0,99788	-0,64293	0,059062	0,631505	-0,17774	0,610101	0,308594	0,657459	0,444983	0,669368	0,643832	0,867985	0,926099
biocarta_cell_to_cell_adhesion_signaling_Pathwa	-0,20696	-0,16182	-0,28248	-0,35244	0,440899	-0,87919	-0,82389	-0,99788	-0,64293	0,059062	0,631505	-0,17774	0,610101	0,308594	0,657459	0,444983	0,669368	0,643832	0,867985	0,926099
biocarta_cell_to_cell_adhesion_signaling_Pathwa	-0,20696	-0,16182	-0,28248	-0,35244	0,440899	-0,87919	-0,82389	-0,99788	-0,64293	0,059062	0,631505	-0,17774	0,610101	0,308594	0,657459	0,444983	0,669368	0,643832	0,867985	0,926099
NCI_Signaling_mediated_by_p38_gamma_and_p	0,039816	-0,05833	-0,01475	0,214351	0,010142	-0,37124	0,608504	0,681935	0,568165	0,041331	0,018328	0,050307	0,369939	0,095569	0,156272	0,548713	0,064632	0,206142	0,868422	0,926099
reactome_Hydroxycarboxylic_acid_binding_recep	0,041029	0,030095	-0,1522	-0,07719	0,379037	-0,30649	0,13901	0,001408	0,021973	1,065468	0,279751	-0,74215	-0,07154	0,293325	0,007854	-0,27004	0,350662	0,031442	0,868218	0,926099
reactome_Interleukin_1_processing_Main_Pathw	-0,02581	-0,23288	-0,17358	-1,19416	-0,69141	-0,93886	0,195519	0,274248	0,083037	-0,03416	-1,47121	0,167677	0,246358	0,139988	0,094165	-0,23415	-1,21797	-0,78864	0,866764	0,926099
reactome_SHC_mediated_cascade_Main_Pathwa	-0,61597	-0,40971	-0,18761	-1,23741	0,192162	-0,4122	-0,63821	-1,10832	0,469511	-0,36974	-1,15424	-1,14264	-0,6924	-0,27611	-0,68572	-0,20265	-0,19986	-0,74423	0,868351	0,926099
reactome_Keratan_sulfate_biosynthesis_Main_Pa	-0,56028	-0,1162	-0,30718	0,786861	0,967266	1,373699	-2,1824	-3,14013	-2,15602	2,679075	0,353248	-0,65508	-1,289	-0,6226	-0,61077	-1,11073	0,73436	0,767011	0,869353	0,926775
KEGG_Toll_like_receptor_signaling_Main_Pathwa	0,402006	-0,15433	-0,00688	-2,42271	0,466499	-1,4831	-4,50219	-3,84913	-5,27457	2,725713	2,005098	-2,25705	1,972656	0,543859	0,599539	0,743583	-1,43064	-1,23475	0,86982	0,926955
reactome_Class_II_GLUTs_Main_Pathway	-0,23425	0,035856	0,170725	1,080839	0,308635	0,425639	0,313674	-0,04202	0,499808	0,426356	0,490207	0,228555	0,130954	-0,20157	-0,13514	0,532507	0,212126	0,882627	0,870673	0,927229
reactome_Smooth_Muscle_Contraction_Main_Pa	-0,2571	-0,13411	-0,46801	-1,11506	-2,8753	-0,51223	-4,11128	-4,48974	-4,33023	-1,67688	-0,17204	0,209564	0,720396	0,664136	0,627341	-1,45427	-0,30475	-0,42049	0,870491	0,927229
Ras_Pathway_Cell-Cell_Junctions	-0,05038	-0,05571	0,134929	-1,43799	1,174406	-0,20418	-0,47096	-0,77925	-0,69222	-1,65236	-1,59412	-1,23126	-0,25554	1,183953	0,241644	1,003672	1,808088	1,792153	0,870998	0,927259
biocarta_il_2_receptor_beta_chain_in_t_cell_acti	0,189159	-0,00883	-0,23829	-0,73623	0,774331	-1,89813	0,026021	0,197636	-0,82319	2,112505	1,583624	2,476244	0,252449	-0,66036	-0,15104	-4,63711	-3,15543	-3,35131	0,871301	0,927264
KEGG_Toxoplasmosis_Main_Pathway	0,302015	-0,6492	-0,20426	-1,27827	-0,55477	-0,01821	-3,24768	-2,35471	-4,563	3,633309	2,839294	0,935551	0,560633	-1,06439	-0,13247	-2,58915	-4,04298	-2,62655	0,872358	0,928073
biotin-carboxyl_carrier_protein_assembly	0,045102	0,015617	-0,009	-0,2345	-0,39531	-0,18718	0,180918	0,188095	0,078614	-0,06158	0,251145	-0,09335	-0,00796	0,014339	0,03562	-0,42937	-0,34157	-0,38731	0,87271	0,92813
reactome_Interaction_With_The_Zona_Pellucida_	-0,60334	-0,26292	-0,30834	0,527798	-0,27864	0,796704	-0,35811	-0,38136	0,124563	1,411474	0,502288	0,411758	0,297248	0,589784	0,015374	1,461613	0,450783	0,279631	0,874214	0,929411
reactome_Binding_and_entry_of_HIV_virion_Mai	-0,12356	0,028845	0,066827	-0,58568	-0,37672	-0,64745	-0,14766	-0,13729	-0,09928	0,740666	0,402325	0,165053	0,398429	0,395569	0,672217	-0,33224	-0,44363	-0,3307	0,875928	0,930916
reactome_Nef_Mediated_CD4_Down_regulation_	-0,14467	-0,10252	0,041247	-0,77706	-0,10884	-0,46811	-0,27376	-0,23072	-0,42189	-0,71682	0,793024	0,599746	0,32854	0,020775	0,585947	-0,58542	-0,21276	0,074885	0,876624	0,931338
NCI_LKB1_signaling_events_Pathway_(Pathway_	0,043281	-0,01136	0,071647	0,086085	0,358827	0,025743	-0,04656	-0,0826	-0,06706	0,474034	0,362742	0,199078	-0,15757	-0,00637	0,017373	-0,13481	-0,18014	-0,18935	0,87701	0,93143
NCI_CDC42_signaling_events_Pathway_(actin_fila	0,013883	-0,04103	-0,01058	-0,21981	0,12043	0,117132	-0,31374	-0,24642	-0,38937	-0,00275	-0,07675	0,03016	0,420713	0,38358	0,355269	0,745649	0,398892	0,511006	0,878443	0,932635
reactome_Activation_of_the_mRNA_upon_bindir	0,228883	0,209107	0,233641	-0,29875	-0,34017	-0,53131	-0,06014	0,073475	-0,46586	1,089354	0,909003	0,286854	0,351088	-0,51544	0,025162	-2,26897	-1,25951	-0,84856	0,879562	0,933504
acetyl-CoA_biosynthesis_from_citrate	0,013074	-0,04256	0,007746	-0,08651	-0,25113	0,019459	0,160921	0,17087	0,137084	0,389886	0,468668	0,179644	0,106905	0,036593	0,05349	-0,34117	-0,31035	-0,38613	0,880462	0,934141
biocarta_angiotensin_ii_mediated_activation_of_	-0,11041	-0,48974	-0,40226	-0,66751	-0,87462	-1,4458	-1,30571	-0,73348	-1,8048	-1,62248	-0,10647	0,106639	0,148851	-0,21075	0,549867	-1,09618	-0,17605	0,642714	0,884514	0,935121
biocarta_mechanism_of_acetaminophen_activity_	-0,0146	-0,18976	-0,07307	-0,51416	-0,2642	-0,14129	-0,00925	0,001731	-0,14646	-0,29669	-0,18518	0,132017	0,207878	0,045529	-0,05837	-0,10471	-0,06751	-0,14021	0,886489	0,935121
biocarta_mechanism_of_acetaminophen_activity_	-0,0146	-0,18976	-0,07307	-0,51416	-0,2642	-0,14129	-0,00925	0,001731	-0,14646	-0,29669	-0,18518	0,132017	0,207878	0,045529	-0,05837	-0,10471	-0,06751	-0,14021	0,886489	0,935121
biocarta_mechanism_of_acetaminophen_activity_	-0,0146	-0,18976	-0,07307	-0,51416	-0,2642	-0,14129	-0,00925	0,001731	-0,14646	-0,29669	-0,18518	0,132017	0,207878	0,045529	-0,05837	-0,10471	-0,06751	-0,14021	0,886489	0,935121
biocarta_mechanism_of_acetaminophen_activity_	-0,0146	-0,18976	-0,07307	-0,51416	-0,2642	-0,14129	-0,00925	0,001731	-0,14646	-0,29669	-0,18518	0,132017	0,207878	0,045529	-0,05837	-0,10471	-0,06751	-0,14021	0,886489	0,935121
biocarta_mechanism_of_acetaminophen_activity_	-0,0146	-0,18976	-0,07307	-0,51416	-0,2642	-0,14129	-0,00925	0,001731	-0,14646	-0,29669	-0,18518	0,132017	0,207878	0,045529	-0,05837	-0,10471	-0,06751	-0,14021	0,886489	0,935121
D-glucuronate_degradation	0,052183	-0,00068	-0,00104	-0,27965	-0,10316	-0,03091	0,264994	0,217601	0,201304	0,132664	0,126934	-0,03808	0,04287	0,008273	-0,01865	-0,17426	-0,43015	-0,13718	0,882728	0,935121
IGF1R_Signaling_Pathway_Cell_Proliferation_Dife	0,031949	-0,21753	-0,3871	-0,36809	-0,15964	-0,91535	-0,21286	0,121501	-0,47099	-0,95481	1,121851	0,313309	0,197534	0,233709	0,486338	-0,68928	-0,59419	0,195494	0,884904	0,935121
NCI_BCR_signaling_Pathway_(cytokine_secretion	0,072848	0,088266	-0,0741	0,085078	0,106327	-0,65939	0,170706	0,161928	0,088354	-0,88368	0,370267	-0,68032	-0,28954	-0,38135	-0,10917	-0,64313	-0,63431	0,242576	0,881806	0,935121
NCI_Notch_signaling_Pathway_(Bergmann_glial_	0,20945	0,296338	-0,00916	-1,80865	-0,17944	-0,31342	-1,83153	-1,47168	-2,57343	0,240791	0,978878	0,751672	-0,44752	0,148306	-0,6003	-3,95532	-1,34274	-1,19666	0,884603	0,935121
reactome_Adenosine_P1_receptors_Main_Pathw	-0,02526	-0,03906	-0,00851	0,68115	-0,11903	0,111048	-0,24773	-0,35203	-0,1233	-0,48419	-0,47348	-0,36543	0,056267	-0,15806	-0,06786	1,049538	0,917407	0,492558	0,883945	0,935121
reactome_Basigin_interactions_Main_Pathway	-0,3303	-0,18936	-0,35459	-0,20793	-0,1727	-0,36135	-2,57943	-2,61879	-2,20212	0,076556	-0,43265	0,514157	0,579024	0,425065	0,607215	0,608824	0,866602	0,882952	0,884709	0,935121
reactome_Depolymerisation_of_the_Nuclear_Lar	-0,02256	-0,13168	-0,19155	-1,00016	-0,03124	-0,97548	0,033077	0,23812	-0,26707	-0,13108	0,047299	-1,96951	-0,01556	-0,20343	0,210393	-0,64746	-0,3899	0,00692	0,882885	0,935121
reactome_Digestion_of_dietary_carbohydrate_M	-0,45443	-0,38807	-0,38018	-2,23658	-2,83175	-2,80363	0,95386	0,927856	1,112595	0,073711	-0,00743	-0,88842	0,261776	0,130697	0,245016	-2,80751	-3,63017	-3,45934	0,884857	0,935121
reactome_Iron_uptake_and_transport_Main_Pat	0,081775	-0,16641	-0,18555	-1,85501	-0,98617	-2,5423	-1,01303	-0,75391	-1,41173	0,982601	0,747818	-0,1716	0,416029	0,94362	0,634669	-2,97324	-1,96268	-2,46949	0,882321	0,935121
reactome_O_linked_glycosylation_of_mucins_Ma	-1,18049	-0,21026	-0,28229	-1,45266	0,224578	-0,74186	1,188496	-0,81832	1,592914	0,263675	-2,83033	-4,10102	-0,9442	2,060707	0,462542	2,981342	3,346714	2,764921	0,88527	0,935121
reactome_Sodium_Calcium_exchangers_Main_Pa	-0,128	-0,38063	0,030112	0,624734	0,621817	0,786145	0,295656	0,100508	0,497505	-1,79417	-0,77959	-1,14942	-1,11949	-0,47167	-0,54354	2,121144	0,939178	1,712989	0,884286	0,935121
NCI_BMP_receptor_signaling_Main_Pathway	0,067149	-0,13077	-0,06555	-0,56906	-0,28483	-1,75639	-1,06605	-0,64125	-1,98653	-1,96513	1,03413	-0,19686	1,184717	-0,7242	0,91814	-0,40895	-1,0301	0,155955	0,887061	0,935408
NCI_CD40_CD40L_signaling_Pathway_(proteasom	-0,12515	-0,04759	-0,07843	-0,01959	-0,23049	0,493297	-0,17981	-0,18705	-0,17902	0,110927	-0,01624	-0,12868	-0,23895	-0,16821	-0,08459	-0,03937	0,043514	0,059827	0,88938	0,937536
biocarta_mcalpain_and_friends_in_cell_motility_	-0,01794	-0,05098	-0,16151	-0,48607	-0,60097	-0,81125	-1,11875	-1,00188	-1,31163	-0,09021	0,009005	-0,56333	0,096186	-0,15277	-0,33415	-1,28034	-0,90804	-0,11236	0,890087	0,937678
KEGG_Rap1_signaling_Main_Pathway	-2,13021	-2,49566	-1,67701	-3,26638	-1,53996	-6,33005	-11,6097	-12,3885	-10,8392	-0,76256	-3,22115	-1,59282	1,434536	2,256732	5,325761	0,875702	4,723583	5,675412	0,89047	0,937678
reactome_SHC_related_events_triggered_by_IGF	0,071944	0,018494	0,009333	0,513829	0,719907	0,08194	-0,5869	-0,51046	-0,63923	0,560994	-0,438	0,403855	0,104078	0,088048	-0,04796	0,507862	0,449022	0,830234	0,890719	0,937678
reactome_Signaling_by_Type_1_Insulin_like_Gro	0,071944	0,018494	0,009333	0,513829	0,719907	0,08194	-0,5869	-0,51046	-0,63923	0,560994	-0,438	0,403855	0,104078	0,088048	-0,04796	0,507862	0,449022	0,830234	0,890719	0,937678
NCI_Endothelins_Main_Pathway	-0,26623	-0,95097	-0,55098	-1,95238	-0,74646	-4,94333	-3,66516	-2,9436	-3,80338	-2,63795	-1,38083	-2,52592	-0,48649	-0,12001	0,832715	-0,83146	-1,38184	0,543712	0,891542	0,938228
mTOR_Pathway_Inflammation_Stress_Resistance	-0,10335	0,182237	0,042026	-0,83301	-1,73076	-0,08673	0,355609	0,282316	0,212461	0,501239	-0,56306	-0,28069	0,193556	0,616478	0,429395	-0,81914	-0,88231	-0,69586	0,892345	0,938756
reactome_Costimulation_by_the_CD28_family_M	-0,36399	-0,04325	0,096637	-0,60733	0,268168	0,799089	0,123157	0,069739	0,409083	-0,03293	0,725203	1,026376	0,002205	0,111252	0,358773	-0,91145	-0,20629	0,761836	0,892834	0,938953
PAK_Pathway_Contractility_Stress_Fibres_and_F	-0,51525	0,461046	0,252376	-2,02738	0,225927	-0,48131	-3,09579	-3,59882	-1,88432	-0,457	-2,58924	-2,31746	1,393258	3,00451	2,009758	3,515872	1,176291	5,274051	0,893466	0,9393
biocarta_il_2_receptor_beta_chain_in_t_cell_acti	0,022121	-0,11951	-0,18126	-0,39829	0,131766	-0,55464	-0,33501	-0,18034	-0,71888	0,236217	1,025295	0,974646	0,053906	-0,11906	0,15366	-1,4672	-0,87901	-0,35595	0,895206	0,939792
KEGG_Arginine_and_proline_metabolism_Main_	-0,45105	-0,16631	-0,2295	-3,39895	-2,28853	-3,68174	-0,55125	-0,96627	-1,16437	-1,38313	-2,42807	0,084094	0,633615	0,358425	0,341047	-4,97167	-2,49061	-1,21487	0,895269	0,939792
NCI_Signaling_events_mediated_by_Stem_cell_fa	0,007292	-0,05705	0,114145	-1,1892	-0,27154	-0,75305	-1,07193	-0,8797	-1,10589	0,66259	-0,13828	0,158836	-0,72462	-0,91379	-0,83575	-2,77418	-3,08118	-2,53943	0,896303	0,939792
NCI_Signaling_events_mediated_by_Stem_cell_fa	0,007292	-0,05705	0,114145	-1,1892	-0,27154	-0,75305	-1,07193	-0,8797	-1,10589	0,66259	-0,13828	0,158836	-0,72462	-0,91379	-0,83575	-2,77418	-3,08118	-2,53943	0,896303	0,939792
NCI_Signaling_events_regulated_by_Ret_tyrosine	-0,23394	-0,052	-0,21711	-0,32644	-0,49178	-0,0978	-2,74209	-3,04494	-2,79004	1,24185	-0,15554	0,230591	-0,01946	-0,21654	-0,07124	-1,14776	-0,77894	-0,4393	0,896188	0,939792
reactome_Abnormal_metabolism_in_phenylketo	-0,04408	-0,05107	-0,01362	-0,1674	0,003969	-0,1917	-0,1672	-0,26091	-0,13513	0,176482	0,056449	0,381203	0,054204	0,126682	0,085514	-0,19616	-0,1753	-0,16629	0,8949	0,939792
reactome_Synthesis_of_16_20_hydroxyeicosatet	-0,10286	0,399271	0,220413	0,923193	0,752488	-0,00764	-0,52241	-0,55226	0,074718	2,442587	-0,1866	1,33556	0,367391	0,895262	1,189268	0,902987	0,235401	0,541868	0,896347	0,939792
reactome_The_NLRP1_inflammasome_Main_Patl	-0,01698	-0,06358	-0,05214	0,090895	0,428118	0,257368	-0,38924	-0,26363	-0,29007	0,079721	0,368171	0,032992	0,005031	0,143573	0,001007	0,331005	0,511831	0,081741	0,895903	0,939792
acetate_conversion_to_acetyl-CoA	0,10078	0,063881	0,016315	0,01372	-0,04538	0,053406	-0,52361	-0,40125	-0,58642	0,963104	-0,21079	0,554045	0,257163	0,296311	0,363854	0,177196	-0,08577	-0,00349	0,896664	0,939808
thyroid_hormone_biosynthesis	-0,01447	-0,08025	-0,12353	0,104846	-0,32674	0,081336	0,13139	0,073403	0,217879	0,046939	-0,34943	0,045595	0,126655	-0,00158	0,195536	0,455828	0,238763	0,178303	0,897802	0,940684
reactome_Phagosomal_maturation_early_endoso	-0,251	-0,24411	-0,05148	-1,52609	-1,95506	-0,13803	-2,03306	-2,36768	-2,06257	-0,79872	0,665251	-2,4111	-1,03377	-1,37046	-1,46753	-3,33492	-2,53759	-2,9217	0,898527	0,941127
NCI_CXCR3_mediated_signaling_events_Pathway	-0,17922	0,0171	0,105503	0,742462	0,588297	0,042392	1,37979	1,496892	1,177159	2,022476	1,718597	-0,50417	-0,67107	-0,06132	0,492861	-0,55549	0,214221	-1,63778	0,900322	0,94269
NCI_Angiopoietin_receptor_Tie2_mediated_signa	0,140378	-0,06042	-0,05341	0,061304	-0,30722	-0,2483	-0,87081	-0,797	-0,79779	0,722545	-0,16144	-0,40341	0,256901	0,190718	0,318801	0,266876	-0,13387	0,265279	0,901843	0,943302
noradrenaline_and_adrenaline_degradation	-0,24602	-0,2623	-0,22963	-1,64291	-0,54795	-1,18177	-0,40175	-0,19922	0,253218	-0,21891	-1,25617	-2,12979	0,747641	0,441626	0,253503	0,971582	0,81683	0,233817	0,90196	0,943302
reactome_Beta_oxidation_of_butanoyl_CoA_to_	0,001767	-0,05251	0,089026	-0,09336	0,102199	-0,3199	0,384287	0,479253	0,489002	0,953759	0,436443	0,66234	-0,04573	0,018351	-0,19475	-0,74803	-0,9297	-0,9412	0,902118	0,943302
reactome_MyD88_dependent_cascade_initiated_	0,078646	0,021833	-0,03128	0,244961	-0,05122	-0,51069	-1,21877	-1,27079	-1,52377	0,59755	0,692745	0,662309	-0,00182	-0,0579	-0,05695	-0,79972	-0,83129	-0,77596	0,901696	0,943302
KEGG_Glucagon_signaling_Main_Pathway	-0,05549	-0,62387	0,203192	-2,60044	-0,64789	-2,04279	-0,8442	-0,75877	-0,92233	-1,88921	-0,60866	-0,18055	1,433403	0,699081	1,885674	0,514761	0,798425	0,854637	0,903065	0,943664
reactome_Fibronectin_matrix_formation_Main_F	-0,0898	-0,08563	-0,11997	-0,69242	-0,64986	-0,68096	-0,26462	-0,33736	-0,49341	0,198065	-0,1114	0,008566	-0,032	0,01701	-0,04242	-1,17062	-0,68549	-0,82759	0,90307	0,943664
KEGG_ErbB_signaling_Main_Pathway	-1,24748	-1,01785	-0,94772	-3,43932	-3,45732	-4,86098	-3,20449	-2,33061	-4,19026	-0,30736	0,982907	-0,15669	0,97062	0,41365	2,885403	-5,52079	-1,91503	0,476514	0,904091	0,944414
reactome_Acyl_chain_remodelling_of_PE_Main_	-0,1199	0,173172	-0,07697	-0,80899	-1,2712	-1,54417	-0,39162	-0,21193	-0,0223	-0,61058	-1,53361	-0,81697	-0,10215	0,0484	-0,0244	-1,59119	-0,43174	-0,92848	0,905785	0,945867
KEGG_Prostate_cancer_Main_Pathway	-0,52441	-0,85895	-0,98641	-3,45263	-0,73949	-3,86274	-2,03313	-0,97266	-3,61402	0,888388	-0,83831	3,440903	-0,58949	-0,70974	1,657643	-6,62762	-4,13212	-0,53745	0,906397	0,94611
NCI_VEGFR3_signaling_in_lymphatic_endotheliun	0,051295	0,000466	-0,00961	-0,43517	-0,39938	-0,34965	-0,16569	-0,14703	-0,34268	0,588598	1,322103	1,285885	0,313336	0,5687	-0,24606	-1,29961	-1,13632	-1,21584	0,906626	0,94611
Nicotine_Influence_on_Glutamatergic_Neurons	-0,09146	-0,21437	-0,33199	3,424196	4,419831	1,603664	-4,07078	-3,83188	-3,87409	-2,45499	0,268643	-0,18859	-0,44187	-1,2033	0,38918	3,571386	3,913351	6,434157	0,907716	0,946614
reactome_WNT5A_dependent_internalization_of	-0,1726	-0,06369	0,047465	-0,54571	-0,04124	-1,29491	-0,71767	-0,78786	-0,98642	-2,35768	0,637859	0,870378	0,681706	0,544149	0,273646	-0,30735	0,155951	0,154048	0,907434	0,946614
Akt_Signaling_Pathway_Survival_Genes	0,191963	-0,13856	0,383282	-1,0942	0,87219	-1,0333	-0,21642	-0,16491	-0,24938	-0,02964	1,714503	-0,04558	0,671647	0,469239	0,422092	-1,10299	-0,17835	0,116817	0,910753	0,949146
reactome_Neurotransmitter_Clearance_In_The_	0,165823	0,103317	0,162247	-0,14626	0,038957	-0,19173	-0,81776	-0,82826	-0,88284	-0,34608	0,472979	-0,45728	0,27608	0,132663	-0,18795	0,105314	0,028254	-0,49128	0,910512	0,949146
reactome_Downstream_TCR_signaling_Main_Pat	-0,90208	0,019483	-0,38964	-0,72651	0,730474	1,907354	-1,06116	-1,48803	-0,54778	0,33391	-0,58235	1,827826	1,040878	0,229091	0,541475	1,300077	0,973889	2,473104	0,912445	0,950591

reactome_Activation_of_Matrix_Metalloproteina	-0,1144	0,113781	0,193415	0,642028	-1,14577	-0,13686	-0,45337	-0,42735	0,059036	0,836556	0,221891	1,1704	0,631053	0,13841	1,438985	0,302709	0,6027	-0,57766	0,914203	0,952103
NCI_Syndecan_2_mediated_signaling_events_Pat	0,015193	-0,07952	-0,01752	0,187942	0,237099	0,180139	0,033965	-0,0872	0,21701	0,126195	-0,20804	-0,61351	-0,29027	-0,18446	-0,26039	0,446748	0,174722	-0,17587	0,915905	0,9532
NCI_Syndecan_2_mediated_signaling_events_Pat	0,015193	-0,07952	-0,01752	0,187942	0,237099	0,180139	0,033965	-0,0872	0,21701	0,126195	-0,20804	-0,61351	-0,29027	-0,18446	-0,26039	0,446748	0,174722	-0,17587	0,915905	0,9532
reactome_Acyl_chain_remodelling_of_PG_Main_	-0,04067	0,053882	-0,00922	-0,76314	-1,38803	-0,33191	0,661044	0,553666	0,850431	-1,96561	-0,93655	-0,44876	-0,62833	-0,78338	-0,26012	-1,54752	-0,79407	-0,8194	0,916479	0,9532
TGF-Beta_Pathway_Post_Transciptional_G1_Arre	0,070795	-0,27213	-0,13396	-0,79212	-1,15443	-0,49444	-0,76337	-0,59584	-1,04581	-0,01812	0,774824	1,19253	0,068456	-0,19778	0,042478	-2,35066	-1,71769	-0,92563	0,916191	0,9532
NCI_VEGFR3_signaling_in_lymphatic_endotheliun	-0,03865	0,062417	0,211397	0,214098	0,311813	0,381927	-0,06124	-0,17705	0,065944	0,058639	0,076209	-0,10741	0,09731	0,10017	-0,07183	0,679967	0,519256	0,192119	0,91769	0,953778
NCI_VEGFR3_signaling_in_lymphatic_endotheliun	-0,03865	0,062417	0,211397	0,214098	0,311813	0,381927	-0,06124	-0,17705	0,065944	0,058639	0,076209	-0,10741	0,09731	0,10017	-0,07183	0,679967	0,519256	0,192119	0,91769	0,953778
sulfate_activation_for_sulfonation	0,051808	0,097095	0,079716	0,073412	-0,25477	0,035975	-0,2213	-0,15969	-0,1684	-0,23564	0,042648	0,057248	-0,07277	-0,05323	-0,13761	-0,17785	-0,43262	-0,17016	0,917954	0,953778
NCI_LKB1_signaling_events_Pathway_(establishm	-0,14593	-0,16749	-0,21395	-0,68334	-0,34797	-0,90671	-0,36675	-0,28097	-0,55882	-0,40905	-0,0688	0,372676	0,004374	0,381298	0,134794	-0,49065	-0,71392	-0,50209	0,920352	0,95595
guanine_and_guanosine_salvage	-0,07376	-0,09084	0,008833	-0,2102	0,030001	-0,13297	0,257356	0,244149	0,213475	-0,26342	0,228658	0,058831	-0,10657	0,096276	0,058726	-0,14738	-0,06153	-0,11785	0,922094	0,957122
KEGG_Fatty_acid_degradation_Main_Pathway	0,226049	0,105946	0,44101	-1,53529	-0,77948	-1,39588	0,610828	0,920987	-0,33323	2,440012	0,48657	2,552019	0,694439	0,762147	0,286789	-4,46427	-1,83836	-2,12464	0,92187	0,957122
guanosine_nucleotides_degradation	0,026803	-0,0736	0,008859	0,121584	0,252805	0,14201	0,314507	0,306445	0,084587	1,049262	0,403258	0,735635	-0,00563	0,120452	0,157518	-0,32905	-0,03334	-0,45408	0,922513	0,957238
Cellular_Apoptosis_Pathway_Gene_Expression_B	-0,10472	-0,12522	-0,18298	0,830244	-0,14824	0,252012	0,213073	0,376234	0,222469	0,388461	0,057049	0,166144	-0,02694	-0,27025	-0,30841	0,11542	0,520821	-0,16739	0,923119	0,957547
NCI_Glypican_3_network_Pathway_(apoptosis)	-0,00941	0,113971	0,159205	-0,21512	0,086724	-0,24464	0,381474	0,423163	0,177796	0,349763	0,372852	0,044618	0,442532	0,127329	-0,01998	-0,20041	-0,46451	0,017804	0,923965	0,958106
epoxysqualene_biosynthesis	0,068269	0,036463	0,039558	-0,07696	-0,02949	-0,02164	0,61675	0,590738	0,545264	0,02062	0,445409	0,171686	-0,12696	-0,16997	-0,16244	-0,72878	-0,48821	-0,33891	0,92548	0,959038
reactome_Meiotic_synapsis_Main_Pathway	0,279874	-0,27951	-0,51813	0,591157	-1,14542	-1,3274	6,577364	7,517456	4,837864	4,094719	3,557405	1,915765	-1,00373	-1,12554	0,269798	-7,97856	-3,61265	-4,2445	0,925184	0,959038
biocarta_regulation_of_pgc_1a_Main_Pathway	0,102301	-0,13466	-0,1467	0,398444	-1,21511	0,061202	-0,942	-0,67567	-1,48424	-1,64187	0,080413	-2,18364	-0,31964	-0,4479	-0,2706	0,392464	0,529443	1,476783	0,926143	0,959406
biocarta_d4gdi_signaling_Main_Pathway	-0,13655	-0,24979	-0,38688	-0,42991	-0,86328	-0,91706	-0,1302	0,061652	-0,49421	0,369696	-0,77225	0,580337	-0,9124	-0,73833	-0,10225	-2,06987	-1,75783	-1,81351	0,928427	0,960495
biocarta_d4gdi_signaling_Pathway_(apoptosis)	-0,13655	-0,24979	-0,38688	-0,42991	-0,86328	-0,91706	-0,1302	0,061652	-0,49421	0,369696	-0,77225	0,580337	-0,9124	-0,73833	-0,10225	-2,06987	-1,75783	-1,81351	0,928427	0,960495
NCI_Arf6_downstream_Pathway_(Tumor_Cell_In	0,034943	-0,07768	-0,07106	-0,29789	-0,51936	-0,17898	-0,21997	-0,19548	-0,27889	0,016795	-0,10502	-0,39227	0,262425	0,13447	0,060598	-0,07724	-0,09921	-0,14525	0,927741	0,960495
TNF_Signaling_Pathway_Apoptosis	0,231775	0,056277	-0,05598	0,084716	-0,88131	-1,87717	-0,11763	0,242162	-0,6983	1,192325	-0,75304	0,685261	-0,60827	-0,2497	-0,66569	-3,06066	-2,15278	-2,93154	0,92819	0,960495
NCI_Syndecan_4_mediated_signaling_events_Ma	-0,27043	-0,47386	-0,24748	-0,53641	-0,6342	-0,80567	-2,66666	-2,50248	-2,74619	0,395424	-0,22529	-0,59606	-0,00803	-0,17292	0,615623	-1,14797	0,175525	0,296071	0,929221	0,960997
NCI_Glypican_2_network_Main_Pathway	-0,03293	-0,03777	-0,14424	0,225289	-0,83601	-0,15649	-0,76954	-0,87372	-0,79776	-0,07596	0,05534	-0,16432	0,067743	0,104947	0,001252	-0,17558	-0,13317	-0,31351	0,930093	0,96158
NCI_Signaling_events_regulated_by_Ret_tyrosine	-0,31406	-0,37507	-0,47709	-0,23662	-0,68157	-1,86213	-5,31837	-5,49153	-5,25917	0,750308	0,153815	0,124591	0,677448	0,175391	0,612873	-2,17464	-1,871	-0,30369	0,930937	0,962133
taurine_biosynthesis	0,057423	0,145837	0,14854	-0,41702	-0,25717	-0,43288	-0,32885	-0,32351	-0,34612	0,192402	0,249822	0,263245	-0,0761	-0,00319	0,383104	-0,41845	-1,05188	-0,63536	0,932434	0,963361
Mitochondrial_Apoptosis_Pathway	0,393986	0,507367	0,395177	1,996858	1,584402	2,461139	3,011804	2,667953	3,249295	1,607313	1,084147	3,142028	0,721147	-0,40159	-0,84948	1,271096	0,587418	-0,22459	0,932929	0,963377
NCI_Trk_receptor_signaling_mediated_by_PI3K_a	-0,33694	-0,51292	-0,4302	-1,46159	0,710488	1,094262	-0,92991	-1,1612	-1,20144	0,398026	0,85667	0,3532	-0,42407	-0,92046	-0,49881	-1,3392	-0,39936	-1,19738	0,933841	0,963377
reactome_CYP2E1_reactions_Main_Pathway	-0,09951	0,372131	0,024105	2,02903	1,92962	1,185407	-0,00468	-0,18106	1,285821	-1,0206	0,053463	0,566714	-0,68509	0,290732	-0,59841	3,279401	1,806286	0,747966	0,933267	0,963377
reactome_Elastic_fibre_formation_Main_Pathwa	-0,2465	0,227465	0,214852	0,367048	0,532269	0,13761	-3,25516	-3,38606	-2,66433	0,813304	-1,69112	-0,48877	0,64097	0,816672	0,656072	2,425219	1,421693	1,717374	0,933483	0,963377
reactome_WNT_mediated_activation_of_DVL_Ma	-0,11478	-0,08196	-0,15567	-0,86909	-1,10162	-0,82132	0,682283	0,744589	0,705696	-0,41941	0,479125	-0,12872	0,12982	0,019443	0,277932	-1,16897	-0,93857	-0,78937	0,933996	0,963377
biocarta_how_progesterone_initiates_the_oocyte	0,179289	0,30102	0,078358	-0,84067	-0,63234	-1,08641	0,93388	0,872598	0,918995	-0,16541	-1,98754	-0,95267	0,289182	-0,06013	0,176998	-0,24344	-0,55522	0,268409	0,935856	0,964976
reactome_Sulfur_amino_acid_metabolism_Main_	0,02752	0,065341	0,020078	-0,9341	-1,45428	-0,59226	-0,12352	0,037275	-0,69874	0,115218	0,708533	0,734147	0,117044	0,176169	0,166452	-1,78616	-1,54679	-1,79859	0,937238	0,96608
D-myoi-inositol_134-trisphosphate_biosynthesis	0,099339	-0,07308	0,04185	-0,43462	-0,66297	0,40637	-1,03744	-0,92911	-1,60317	0,622741	0,148752	-0,17217	-0,54862	-0,27601	-0,95004	-1,0348	-1,32474	-1,77595	0,938489	0,966154
KEGG_Adipocytokine_signaling_Main_Pathway	-0,12875	0,166185	-0,04485	3,655221	1,372456	2,558706	0,470606	0,044139	0,669924	1,687365	0,711252	0,333331	-0,0775	0,000544	-0,79593	3,246552	1,511596	2,015158	0,938676	0,966154
NCI_a6b1_and_a6b4_Integrin_signaling_Pathway	-0,02632	-0,03701	-0,07459	-0,72754	-0,37559	-0,88855	-0,86264	-0,83137	-0,93286	-0,02229	0,56872	0,134478	0,055935	-0,11951	0,073231	-1,99239	-1,05551	-0,68415	0,938785	0,966154
NCI_CXCR3_mediated_signaling_events_Pathway	-0,31542	0,110228	0,270496	0,846743	0,399907	0,160263	0,868141	0,885925	0,866192	1,490215	0,96273	-0,75388	-0,92347	-0,51279	0,073737	-0,34739	0,223871	-1,30285	0,938612	0,966154
NCI_Hedgehog_signaling_events_mediated_by_G	-0,03447	0,022676	-0,10341	0,782754	0,781249	0,624185	0,962577	0,799681	0,776879	0,044654	0,316342	0,480154	-0,30229	-0,1203	-0,27446	0,467049	0,424861	0,452875	0,93917	0,966154
NCI_Hedgehog_signaling_events_mediated_by_G	-0,03447	0,022676	-0,10341	0,782754	0,781249	0,624185	0,962577	0,799681	0,776879	0,044654	0,316342	0,480154	-0,30229	-0,1203	-0,27446	0,467049	0,424861	0,452875	0,93917	0,966154
reactome_Toxicity_of_botulinum_toxin_type_B_(	-0,03362	-0,08119	-0,14179	0,183363	-0,09812	-0,49502	-1,30243	-1,15031	-1,32281	-0,96416	-2,05714	-0,71707	-0,0252	-0,2464	-0,25489	0,830422	0,88165	0,649148	0,940452	0,967154
reactome_Formation_of_apoptosome_Main_Path	0,14071	0,145676	0,138001	-0,26195	-0,32668	-0,19564	-0,04895	0,126226	-0,21723	0,220412	-0,25778	-0,69633	-0,16455	-0,20723	-0,25305	-0,70634	-0,70668	-0,48583	0,941575	0,967989
reactome_Effects_of_PIP2_hydrolysis_Main_Path	-0,06645	-0,17042	-0,1597	-0,86714	-0,30289	0,582294	0,164377	0,333903	0,223137	0,592638	-0,2924	-2,07067	1,197065	0,551283	0,544032	1,614749	1,40195	2,050199	0,942283	0,968078
TGF-beta_Signaling_in_Epithelial-mesenchymal_t	-0,45694	-1,05971	-1,2043	-3,01858	-3,22574	-3,29071	-4,48744	-3,83158	-5,82186	1,91311	0,788396	3,935415	1,146433	0,44787	0,682856	-6,30737	-5,40294	-4,05109	0,942077	0,968078
reactome_Signaling_by_EGFR_Main_Pathway	0,107548	-0,033	0,12552	-0,48456	-0,15379	-0,36176	0,177488	0,300037	-0,11114	0,346115	0,026299	0,229943	0,393222	0,30876	0,461336	-0,54286	-0,14851	0,257756	0,944073	0,969596
Integrins_Function_in_Carcinoma_Progression	-0,39781	-0,80622	-0,50149	-4,37189	-2,76775	-5,41028	-5,34919	-5,49098	-6,35731	1,84237	0,354576	-1,77821	1,718266	1,263301	1,555904	-5,81188	-3,61705	-2,2674	0,945511	0,97064
reactome_Triglyceride_Biosynthesis_Main_Pathw	0,093147	-0,04408	-0,12907	-0,50153	-1,19995	-1,22227	0,22851	0,393152	-0,1773	-0,33377	0,200323	-0,24131	-0,04636	-0,26868	0,552223	-1,60138	-1,21846	-0,97604	0,945712	0,97064
CD40_Pathway	0,802245	0,20526	0,287146	-1,77875	-2,46662	-0,39652	0,029776	0,695173	-0,46264	1,417741	0,323801	-0,32942	1,420496	0,559557	2,011603	-1,76083	-1,46838	0,047898	0,946354	0,970971
NCI_Arf6_signaling_events_Main_Pathway	-0,0566	-0,73322	-0,34105	-4,10407	-1,50804	-2,32167	-3,28752	-3,42428	-3,1527	-2,78065	-1,978	-0,9315	0,512037	0,259246	0,158528	-0,39933	-1,52663	-2,26555	0,946658	0,970971
TRH_Influence_on_Cytoskeleton	-0,25591	-0,36732	-0,28093	-0,28579	0,710923	-0,26424	-2,9044	-2,61332	-2,75673	-1,1941	-1,23616	-0,02527	-0,00748	0,536419	0,92972	1,02609	1,931589	2,542742	0,948942	0,972993
HGF_Pathway_IP3_Pathway	-0,26633	-0,14076	-0,18136	-1,10171	-1,24111	-1,02816	-1,51653	-1,45033	-1,40029	0,634818	0,934823	0,77743	0,351574	0,157003	0,108121	-1,80503	-1,87331	-1,93355	0,950768	0,973213
KEGG_Nicotinate_and_nicotinamide_metabolism	0,486712	0,10054	-0,18618	-2,71305	1,109235	-3,47211	-0,31374	-0,58843	-0,21134	-0,61695	-0,07674	-0,23357	0,523592	0,464856	0,02274	-1,7739	-2,32605	-0,97911	0,950098	0,973213
NCI_p38_signaling_mediated_by_MAPKAP_kinase	-0,05499	-0,26977	-0,28927	-0,16298	-0,11311	0,057267	-0,25705	-0,18609	-0,29248	0,651891	0,536816	0,486384	0,188551	-0,03174	0,300723	-0,37943	-0,05059	-0,18605	0,950243	0,973213
reactome_Sema3A_PAK_dependent_Axon_repuls	-0,00981	-0,2486	-0,03998	-1,86289	-0,87467	-1,23631	-4,04151	-3,74734	-4,62239	-1,39628	-1,56592	-1,10819	0,50487	0,505879	0,113271	-0,49359	-0,64044	1,516814	0,951031	0,973213
reactome_Signal_regulatory_protein_SIRP_family	-0,0862	0,005588	0,159617	0,193338	-0,0993	-0,59605	0,291927	0,292135	0,348303	-1,49302	0,064931	-0,26109	-0,42223	0,040395	0,049505	0,044691	0,007756	0,43218	0,950416	0,973213
reactome_STAT6_mediated_induction_of_chemo	-0,04491	-0,00949	-0,0264	-0,08576	-0,25803	-0,19917	-0,33585	-0,39193	-0,37282	-0,17341	0,038661	-0,38026	0,14349	-0,04185	-0,02098	-0,15804	-0,31541	0,323882	0,950085	0,973213
Intermediate_Filaments_in_Epithelial_Cells	-0,09996	0,041916	0,193386	-0,98645	-1,30917	-0,76502	0,430802	0,12728	0,159102	-0,82066	0,309829	0,383385	-0,03825	-0,62119	-0,00991	-2,61776	-1,85174	-0,91471	0,953673	0,975543
NAD_biosynthesis_from_2-amino-3-carboxymuco	0,0331	-0,04089	-0,02936	-0,77763	1,014613	0,577081	-0,70608	-0,99392	-0,7738	0,50351	0,034861	0,213264	0,06693	0,471063	-0,14626	0,440583	0,485965	0,024435	0,95456	0,975543
reactome_PKA_mediated_phosphorylation_of_ke	-0,06757	0,005477	-0,04128	0,132167	-0,27285	-0,51668	-0,11355	-0,13088	0,029708	0,719379	-0,18446	0,711484	-0,10019	0,123918	0,237683	-0,2425	-1,0386	-0,34354	0,954348	0,975543
reactome_Regulation_of_IFNA_signaling_Main_P	0,694853	0,052651	0,908498	-0,33472	1,397184	-1,80116	0,117469	-0,20559	0,732445	0,225292	2,921908	3,252069	2,010868	2,527371	4,01524	1,596835	1,315919	0,256758	0,954466	0,975543
biocarta_pkc_catalyzed_phosphorylation_of_inhil	-0,0856	-0,14988	-0,11364	0,730861	0,527152	0,438514	0,080599	-0,14703	-0,06787	0,909845	-0,17051	0,472327	-0,29924	-0,58962	-0,21247	0,211313	-0,07894	-0,23054	0,956252	0,976701
NCI_Cellular_roles_of_Anthrax_toxin_Main_Path	-0,29683	-0,54659	-0,25611	-0,95008	-0,66393	-1,333	-0,45135	-0,1439	0,03804	3,086007	-0,79161	-0,37922	0,305874	0,756288	-0,20944	-1,44309	-1,18777	-1,61012	0,956321	0,976701
NCI_Noncanonical_Wnt_signaling_Main_Pathway	0,013673	0,050264	0,010032	-0,58808	-0,32949	-0,35433	0,139603	0,101694	0,023653	-0,12898	0,225499	-0,65714	0,23756	0,331873	0,247864	-0,4175	0,107423	0,204947	0,958759	0,97887
HIF1Alpha_Pathway	-0,11755	-0,35063	-0,15362	-0,22069	1,496106	-0,39593	-0,6601	-0,47761	-0,50051	-0,34151	0,497694	2,297943	1,088743	0,606271	0,326774	0,868654	0,176552	1,058537	0,959449	0,979254
biocarta_bone_remodeling_Pathway_(Pathway_p	0,177694	-0,05239	0,109934	0,06423	-0,11313	0,305335	0,011182	-0,00398	0,115473	-0,04219	-0,50498	-0,75616	0,302917	-0,2157	0,353209	0,88443	0,739119	0,396713	0,960156	0,979654
NCI_CXCR4_mediated_signaling_events_Pathway	-0,22653	-0,11828	-0,11108	-1,57829	1,097829	-0,67147	-1,95526	-1,98109	-2,08387	-0,09829	1,028489	1,018391	-0,1791	-0,085	0,843478	-3,5608	-0,67062	1,491917	0,960558	0,979744
NCI_Ephrin_B_reverse_signaling_Pathway_(endo	0,006377	-0,0395	-0,04087	0,155215	-0,06783	0,299004	-0,03162	0,075578	-0,05361	0,425186	0,38762	0,462184	-0,18986	-0,15048	0,032278	-0,73954	-0,19372	-0,08436	0,961143	0,98002
biocarta_regulation_of_splicing_through_sam68_	-0,04904	-0,2368	-0,23635	-0,026	-0,18226	-0,87486	-0,28076	-0,09791	-0,43434	-0,7238	0,593165	0,606338	-0,29571	-0,35858	-0,16043	-1,47422	-0,98677	-0,23225	0,963973	0,981432
biocarta_regulation_of_splicing_through_sam68_	-0,04904	-0,2368	-0,23635	-0,026	-0,18226	-0,87486	-0,28076	-0,09791	-0,43434	-0,7238	0,593165	0,606338	-0,29571	-0,35858	-0,16043	-1,47422	-0,98677	-0,23225	0,963973	0,981432
gluconeogenesis	-0,22719	-0,19895	-0,10612	-0,1053	-0,66432	-1,45342	-0,19958	-0,26579	-0,18557	-0,78042	0,836459	-0,15306	-0,19978	0,016224	0,121857	-0,37887	-1,40395	-0,88294	0,964103	0,981432
proline_biosynthesis	0,004049	-0,05792	-0,01332	0,115628	0,290289	-0,13314	0,669614	0,717282	0,631073	-0,0366	-0,44954	0,497923	-0,30917	-0,56291	-0,24602	-0,52877	-0,2023	-0,55265	0,963061	0,981432
reactome_TAK1_activates_NFkB_by_phosphoryla	0,007948	-0,20993	-0,05395	-0,26973	-0,0682	-0,26773	-1,29755	-1,2986	-1,41761	2,151638	-0,56818	0,52673	0,005793	-0,96098	-0,06998	-1,64845	-1,82507	-1,00546	0,963824	0,981432
KEGG_Thyroid_hormone_signaling_Main_Pathwa	-0,71135	-0,51509	-0,62492	-0,96967	-0,3231	-2,94894	-2,5273	-1,9317	-3,60147	1,149205	1,749552	2,895544	0,613248	2,814308	-4,44348	-0,61945	0,979475	0,96457	0,981586	
Erythropoietin_Pathway_Gene_Expression_Neurc	-0,18002	0,260763	0,053454	-1,07634	-1,14899	-0,14992	0,245956	0,183307	0,423809	0,568332	-0,67295	-0,36742	0,251168	0,44865	0,30899	-0,66218	-0,52597	-0,65764	0,964971	0,981674
NCI_Notch_signaling_Pathway_(oligodendrocyte_	0,17366	0,31308	0,011809	-1,94851	-0,15447	-0,31891	-1,61854	-1,13976	-2,21461	0,107165	0,945037	0,029294	-0,50205	-0,01678	-0,64365	-4,28435	-1,67071	-1,24079	0,965519	0,98191
biocarta_brca1_dependent_ub_ligase_activity_M	0,248444	0,053838	0,013721	0,190353	0,857593	-0,16923	0,874399	0,990748	0,468736	0,18548	0,567665	-0,2892	-0,35813	-0,39307	-0,1579	-0,14868	-0,4293	-0,16376	0,966892	0,982754
reactome_Activation_of_PPARGC1A_PGC_1alpha	-0,01287	-0,27783	-0,26	-1,992	-1,21055	-2,70734	-0,6523	-0,38306	-0,94651	-1,16448	0,180341	-0,65543	0,179664	0,011559	0,153736	-2,66871	-2,08085	-1,63896	0,96698	0,982754
biocarta_the_41bb_dependent_immune_respons	0,294628	0,478805	0,062884	-0,99451	-0,47765	-1,14266	-0,24819	-0,40751	-0,39953	0,10642	-1,21016	0,72605	0,616688	0,582082	0,616251	-0,40428	-0,30624	-0,79843	0,967867	0,983335
biocarta_stat3_signaling_Main_Pathway	-0,02164	-0,12843	-0,075	-0,65575	0,022413	-0,73272	0,143238	0,346872	0,048336	0,182703	0,214823	-0,03307	-0,12588	-0,04573	0,417652	-1,21977	-0,33738	-0,18596	0,970991	0,985721
carnosine_biosynthesis	-0,06959	-0,07798	-0,0078	-0,21848	-0,14161	0,004705	-0,00295	-0,03203	0,179698	-0,12247	-0,04811	-0,03813	0,135531	0,202191	0,11411	0,084653	0,21825	0,179423	0,971165	0,985721
homocarnosine_biosynthesis	-0,06959	-0,07798	-0,0078	-0,21848	-0,14161	0,004705	-0,00295	-0,03203	0,179698	-0,12247	-0,04811	-0,03813	0,135531	0,202191	0,11411	0,084653	0,21825	0,179423	0,971165	0,985721
asparagine_degradation	-0,18705	-0,05536	-0,10965	-0,30044	0,341046	-0,01971	0,377991	0,296454	0,444634	-0,11228	-0,57855	0,240975	-0,13379	-0,20152	0,189214	0,416694	-0,10977	0,26178	0,971922	0,985847
NCI_EPO_signaling_Pathway_(elevation_of_cytos	0,024847	0,104402	0,101726	-0,14687	-0,17484	-0,05786	-0,16285	-0,20018	-0,31035	-0,2674	-0,09175	-0,44393	-0,49241	-0,52361	-0,38488	-0,97277	-0,4432	-0,63523	0,97185	0,985847
KEGG_Cell_adhesion_molecules_CAMs__Main_P	-0,30679	-0,42391	-0,23721	1,832612	1,985776	0,488277	-1,85975	-2,08875	-1,12313	1,59418	0,211073	1,693361	0,971246	0,546628	0,842421	2,969947	2,396445	2,259599	0,973578	0,986563
KEGG_Hedgehog_signaling_Main_Pathway	-0,02997	0,118618	0,137592	0,970126	2,014951	2,207379	2,188738	1,756074	2,481558	-0,56712	-1,96703	-1,10659	-0,28225	0,175846	-0,31345	4,210386	3,519727	2,579736	0,973061	0,986563
reactome_FGFR2b_ligand_binding_and_activatio	-0,14596	-0,09329	-0,16619	-0,3829	0,539372	0,119941	-0,26284	-0,29289	-0,03213	0,682828	-1,25252	0,139363	-0,14145	-0,45776	-0,02789	0,300799	-0,16894	0,10766	0,973397	0,986563
KEGG_Axon_guidance_Main_Pathway	0,420335	0,66287	0,929053	-1,13135	-4,53376	-3,33172	0,820634	0,960475	0,284091	0,155846	0,025829	-0,59321	-0,66359	-1,12094	-0,70992	-7,2254	-4,81881	-6,43369	0,975649	0,988238
NCI_ErbB4_signaling_events_Main_Pathway	-0,32819	-0,21876	-0,3352	-3,45184	-2,89838	-2,24904	-0,76454	-0,75446	-1,26493	0,442553	0,11316	0,361333	0,245676	0,140668	0,494842	-5,04118	-3,80649	-2,493	0,976183	0,988238
reactome_Synthesis_of_GDP_mannose_Main_Pa	0,08258	0,087708	0,074462	0,477713	0,208085	0,279123	-0,37324	-0,20905	-0,30167	-0,52391	-0,40977	-0,3513	-0,41222	-0,33361	-0,12574	0,501357	0,358612	0,336502	0,976182	0,988238
biocarta_lissencephaly_gene_lis1_in_neuronal_m	0,029444	0,034675	-0,00396	-0,73928	-1,53854	-0,32988	-0,1093	-0,05812	-0,3666	0,478425	0,227544	0,470782	0,174945	0,366496	0,350658	-1,3089	-1,02156	-1,38059	0,97825	0,989425
ERK_Signaling_Pathway_Translation	0,024113	0,100887	-0,14722	-0,43786	-0,28535	-0,31929	0,204473	0,150963	0,126164	-0,80448	0,587821	0,049351	-0,25085	-0,17915	-0,28886	-1,17448	-1,11836	-0,21098	0,978308	0,989425
reactome_IRS_activation_Main_Pathway	0,01443	-0,06728	-0,05155	0,49333	0,23686	-0,52988	-0,77644	-0,94326	-0,9352	1,015073	-0,36569	-0,96174	0,183375	0,261929	0,18598	0,607602	0,420952	0,670342	0,978153	0,989425
biocarta_g_protein_signaling_through_tubby_pro	-0,21711	-0,13999	-0,09446	0,123964	1,314031	0,670475	-0,24599	-0,49388	-0,12754	0,915043	0,302978	-0,80297	-0,15238	0,289575	0,302289	1,531157	0,322674	1,920755	0,979798	0,98995
Cellular_Apoptosis_Pathway_Depolarization	0,03205	0,113157	0,005226	0,505627	-0,12593	0,493697	0,416678	0,441865	0,354458	0,517096	-0,02148	-0,09843	0,127128	0,211714	0,11927	0,584687	0,774338	0,123438	0,980222	0,98995
Chemokine_Pathway_Internalization_Degradatior	-0,00969	-0,34304	-0,14025	-0,15845	-0,09895	0,081383	-1,00347	-1,14768	-0,97531	-0,58112	-0,15065	-0,33544	-0,33531	-0,50969	-0,34014	-0,44982	0,486041	-0,55052	0,979598	0,98995
GABA_shunt	0,044306	-0,04622	-0,11706	-0,28694	-0,00347	-0,36021	0,313616	0,415341	0,271749	0,300822	0,692338	-0,93707	0,552547	0,094195	0,4226	-0,30956	0,483914	0,569947	0,979521	0,98995
methylglyoxal_degradation_VI	-0,03319	-0,11033	-0,04313	0,160439	0,211542	0,09724	0,005883	0,020238	0,032729	0,044279	0,09738	-0,01646	-0,00191	0,152651	-0,02988	0,407441	0,408202	0,147749	0,980416	0,98995
reactome_Glycosphingolipid_metabolism_Main_I	-0,40964	-0,48681	-0,02914	-0,2825	-1,07218	-1,12118	-5,55753	-4,72364	-5,34113	1,591497	0,045996	1,879735	-0,68779	-1,17679	0,244329	-2,24458	-3,76954	-2,8701	0,981167	0,990168
reactome_Synthesis_of_12_eicosatetraenoic_acic	-0,10201	-0,15998	-0,10902	0,548093	0,377057	0,679177	-0,08709	-0,32012	0,059484	0,092688	0,438914	0,682349	-0,00243	-0,22202	-0,05156	0,575656	0,366319	0,168191	0,981267	0,990168
acyl-CoA_hydrolysis	0,092326	0,104646	0,02515	-0,11657	-0,06859	-0,11968	-1,13337	-1,11821	-1,2547	-0,3548	0,505079	-0,15648	-0,20756	-0,14312	-0,05588	-0,22337	-0,83109	-0,25427	0,982358	0,990894
NCI_Validated_transcriptional_targets_of_deltaN	0,313138	-0,01297	0,204298	-0,14384	0,07736	-0,24269	0,470524	0,361748	0,503813	0,386872	0,956776	0,226101	0,23055	0,552449	0,730907	-0,16197	-0,30871	0,163803	0,982623	0,990894
biocarta_il_7_signal_transduction_Main_Pathway	-0,06265	-0,04672	-0,10523	-0,39609	0,642933	0,206549	0,056738	0,112585	-0,13959	0,747508	0,201206	1,575801	0,252681	0,178494	0,508479	-0,24807	-0,10051	0,173928	0,983628	0,991586
NCI_Wnt_signaling_network_Main_Pathway	-0,97037	-0,66132	-0,33522	0,267786	0,071332	-1,5489	-1,66385	-1,90616	-0,54644	-2,78545	-0,94159	-1,56484	-0,93499	0,261465	0,164658	1,119019	1,250827	2,424856	0,984052	0,991693
NCI_Ceramide_signaling_Main_Pathway	0,071716	-0,54768	-0,22183	-0,85502	-0,08054	-1,32406	-3,41366	-2,96465	-3,94028	-2,77238	-0,63929	3,026571	-0,44826	-0,41134	-0,27125	-1,87802	-1,72581	-0,49931	0,984929	0,991935
reactome_Unblocking_of_NMDA_receptor_glutai	-0,13707	-0,09529	-0,0166	2,028428	2,71313	1,854021	-2,02825	-1,93196	-1,85608	1,314716	1,010402	-0,59063	0,17143	0,150568	0,154184	2,687146	3,246715	3,310922	0,984711	0,991935
L-serine_degradation	-0,05395	-0,06273	-0,04574	-0,02682	0,056502	-0,06386	-0,22188	-0,22772	-0,25287	-0,21267	0,047587	-0,05965	0,074775	0,135821	0,197787	0,339588	0,265046	0,314682	0,986251	0,992945
NCI_Endothelins_Pathway_(cAMP_biosynthetic_p	-0,19234	-0,24665	-0,18794	-0,37801	-0,62867	-1,85419	-2,63482	-2,30303	-2,51322	-0,49019	-0,64281	-0,32714	0,972228	1,076067	1,284795	0,91315	0,456373	1,511394	0,986929	0,992985
reactome_HSF1_activation_Main_Pathway	-0,12156	-0,05689	-0,12855	-0,68683	-0,48141	-0,15593	1,023921	1,198888	1,073257	0,555355	0,247292	0,183096	0,09144	-0,18504	-0,04745	-1,14914	-0,76625	-0,99594	0,986824	0,992985
NCI_Arf6_downstream_Pathway_(actin_filament_	0,023558	-0,07546	-0,04222	-0,18706	-0,36513	0,012308	0,151889	0,096177	0,062623	-0,34629	-0,51731	-0,26716	0,22184	0,202798	0,066586	0,447237	0,396682	0,286762	0,987367	0,993106
1D-imyoi-inositol_hexakisphosphate_biosynthesis	0,0289	-0,19052	-0,04979	-0,39228	-0,42462	0,495781	-1,31197	-1,27251	-1,90917	1,456699	0,178427	-0,09736	-0,28805	0,122679	-0,78301	-1,03356	-1,14157	-1,0112	0,988204	0,993626
NCI_ErbB2_ErbB3_signaling_events_Pathway_(he	0,143909	-0,07851	-0,02687	-0,1152	-0,07687	-0,66189	-0,59562	-0,48172	-0,87668	0,567443	0,973238	0,887422	0,11577	0,234141	0,214049	-1,30634	-1,0161	-0,61591	0,989414	0,993881
NCI_ErbB2_ErbB3_signaling_events_Pathway_(m	0,143909	-0,07851	-0,02687	-0,1152	-0,07687	-0,66189	-0,59562	-0,48172	-0,87668	0,567443	0,973238	0,887422	0,11577	0,234141	0,214049	-1,30634	-1,0161	-0,61591	0,989414	0,993881
NCI_ErbB2_ErbB3_signaling_events_Pathway_(ne	0,143909	-0,07851	-0,02687	-0,1152	-0,07687	-0,66189	-0,59562	-0,48172	-0,87668	0,567443	0,973238	0,887422	0,11577	0,234141	0,214049	-1,30634	-1,0161	-0,61591	0,989414	0,993881
reactome_Hexose_uptake_Main_Pathway	0,06702	0,126892	0,120883	0,474192	-0,81796	-0,03322	0,388037	0,30372	1,059473	-1,35672	-0,29618	-0,82524	0,203111	0,028706	-0,00088	0,99916	0,482695	0,431092	0,990786	0,994938
KEGG_Inflammatory_bowel_disease_IBD__Main_	-0,046	-0,31475	-0,18413	-1,88025	0,172371	-0,08911	-1,45256	-1,37169	-1,42587	1,18488	-0,8571	1,156209	0,226441	-0,66523	0,216114	-1,01092	-1,69846	-1,30797	0,991131	0,994964
NCI_Alpha4_beta1_integrin_signaling_events_Pat	0,008081	-0,12858	-0,14161	-0,52811	-0,94558	-0,39841	-0,8763	-0,66451	-1,03405	0,549407	0,509751	0,826326	-0,42673	-0,00505	-0,2381	-2,41088	-1,69487	-1,3667	0,992148	0,99554
NCI_Class_IB_PI3K_non_lipid_kinase_events_Path	-0,07086	0,001065	0,002224	0,223478	-0,46504	0,111787	0,224278	0,207324	0,348193	0,22125	0,202478	-0,32903	-0,22508	0,226034	0,108009	-0,31768	0,175488	0,071997	0,992344	0,99554
reactome_HS_GAG_degradation_Main_Pathway	-0,38685	-0,17624	-0,27962	-0,36603	-0,68981	-0,15186	0,512096	0,247602	0,49684	-1,15449	-0,42799	-1,5206	0,54853	-0,11856	-0,15737	1,646714	0,400605	0,475398	0,99497	0,997853
biocarta_il_7_signal_transduction_Pathway_(cell_	-0,00631	0,180577	0,039349	-0,92668	0,053686	-0,34233	0,484717	0,516633	0,674298	-0,41761	0,17625	0,29385	0,305783	0,355848	0,577647	-0,27389	0,027985	0,006061	0,99654	0,998784
NCI_Class_I_PI3K_signaling_events_Pathway_(act	-0,07927	0,071653	-0,10765	-0,24108	-0,10482	-0,06898	0,303519	0,277083	0,123498	0,585046	0,707789	0,110008	-0,9281	0,106482	0,002167	-1,95982	-0,87332	-0,31873	0,996397	0,998784
reactome_Regulation_of_gene_expression_in_en	-0,03565	0,115786	0,010587	0,334446	0,036727	0,007012	0,003534	-0,08526	0,16579	0,02197	-0,19045	0,047516	-0,05514	0,225776	0,063933	0,432968	0,087159	0,421944	0,99733	0,999254
NCI_Circadian_rhythm_Main_Pathway	0,10336	0,086481	0,071425	0,646715	0,69634	-0,15441	0,545963	0,735957	0,322307	0,279055	0,631457	0,190671	-0,29819	-0,23995	0,021657	-0,36246	-0,20266	0,21312	0,999083	0,999404
NCI_Circadian_rhythm_Pathway_(chromatin_moc	0,10336	0,086481	0,071425	0,646715	0,69634	-0,15441	0,545963	0,735957	0,322307	0,279055	0,631457	0,190671	-0,29819	-0,23995	0,021657	-0,36246	-0,20266	0,21312	0,999083	0,999404
NCI_Circadian_rhythm_Pathway_(S_phase_of_mi	0,10336	0,086481	0,071425	0,646715	0,69634	-0,15441	0,545963	0,735957	0,322307	0,279055	0,631457	0,190671	-0,29819	-0,23995	0,021657	-0,36246	-0,20266	0,21312	0,999083	0,999404
NCI_ErbB1_downstream_signaling_Pathway_(cell	0,064515	0,135104	0,074536	-0,51627	-0,48765	-0,89093	-0,62129	-0,56396	-0,73758	-0,95979	-0,27471	-0,73117	0,034426	0,102865	0,151688	-0,36673	-0,39486	0,048033	0,998513	0,999404
reactome_Ceramide_signalling_Main_Pathway	-0,15672	-0,06915	0,010013	0,241426	0,388173	0,060582	-0,25718	-0,32307	-0,16227	0,762032	0,282954	0,100244	0,350108	0,063681	0,314156	0,476277	0,295291	0,42369	0,998055	0,999404
NCI_EPHB_forward_signaling_Main_Pathway	-0,18775	-0,33926	-0,40159	0,934522	0,111925	0,668282	-1,76163	-1,66931	-1,73563	0,743204	0,07886	-0,41669	-0,38598	-0,1421	0,018016	0,52251	0,850664	0,956388	0,999795	0,999795
alanine_biosynthesisdegradation	0	0	0	0	0	0	0	0	0	0	0	0	0	0	0	0	0	0	NA	NA
aspartate_biosynthesis	0	0	0	0	0	0	0	0	0	0	0	0	0	0	0	0	0	0	NA	NA
fructose_26-bisphosphate_synthesisdephosphory	0	0	0	0	0	0	0	0	0	0	0	0	0	0	0	0	0	0	NA	NA
glycerol-3-phosphate_shuttle	0	0	0	0	0	0	0	0	0	0	0	0	0	0	0	0	0	0	NA	NA
glycine_cleavage	0	0	0	0	0	0	0	0	0	0	0	0	0	0	0	0	0	0	NA	NA

glycineserine_biosynthesis	0	0	0	0	0	0	0	0	0	0	0	0	0	0	0	0	0	0	NA	NA
ILK_Signaling_Pathway_Regulation_of_Intermedi	0	0	0	0	0	0	0	0	0	0	0	0	0	0	0	0	0	0	NA	NA
lactate_fermentation_reoxidation_of_cytosolic_N	0	0	0	0	0	0	0	0	0	0	0	0	0	0	0	0	0	0	NA	NA
p38_Signaling_Pathway_Apoptosis	0	0	0	0	0	0	0	0	0	0	0	0	0	0	0	0	0	0	NA	NA
reactome_Formation_of_ATP_by_chemiosmotic_	0	0	0	0	0	0	0	0	0	0	0	0	0	0	0	0	0	0	NA	NA
reactome_XBP1_S_activates_chaperone_genes_N	0	0	0	0	0	0	0	0	0	0	0	0	0	0	0	0	0	0	NA	NA
